# Chemical tools for BAZ2A/B biology: discovery of dual and BAZ2B-selective PROTAC degraders

**DOI:** 10.1039/d6md00244g

**Published:** 2026-08-20

**Authors:** Iván Cheng-Sánchez, Katherine A. Gosselé, Leonardo Palaferri, Michael Krummhaar, Eleen Laul, Alessio Ciulli, Cristina Nevado

**Affiliations:** a Department of Chemistry, University of Zurich Winterthurerstrasse 190 CH-8057 Zurich Switzerland; b Centre for Targeted Protein Degradation, School of Life Sciences, University of Dundee 1 James Lindsay Place DD1 5JJ Dundee UK a.ciulli@dundee.ac.uk

## Abstract

Bromodomain adjacent to zinc finger 2A and 2B (BAZ2A/B) are regulatory subunits of imitation switch (ISWI) chromatin remodelling complexes. Despite their implication in diverse pathologies, BAZ2A/B remain poorly understood, largely due to the lack of chemical probes capable of discriminating between these homologues or targeting their multiple domains. To address this challenge, we recently disclosed two first-in-class PROTACs: dBAZ2, which degrades both BAZ2A and BAZ2B, and the BAZ2B-selective degrader dBAZ2B. Here, we describe the medicinal chemistry campaign that led to their discovery. In addition, we report here an optimized synthesis of the BAZ2A/B bromodomain (BRD) ligand BAZ2-ICR enabling two different attachment points for linker incorporation and demonstrate that BAZ2A/B degradation is more readily achieved through recruitment of VHL than CRBN. We also identify a third degrader, 8, which employs an alternative linkage vector to BAZ2-ICR compared to dBAZ2 and dBAZ2B, adding to the structural diversity of BAZ2 PROTACs. Cooperativity assays and the BAZ2B-BRD:8:VHL-EloC-EloB co-crystal structure reflect the ability of 8 to degrade both BAZ2A and BAZ2B. In contrast, dBAZ2B achieves selectivity through preferential ternary complex formation with BAZ2B. Finally, we demonstrate the utility of dBAZ2/dBAZ2B as chemical probes: dBAZ2 induces expression of BAZ2A-regulated genes more strongly than dBAZ2B or BAZ2-ICR, and the BAZ2B selectivity of dBAZ2B is maintained across multiple cell lines. Collectively, this work delivers new chemical tools to interrogate BAZ2A/B biology and establishes a platform for developing heterobifunctional molecules targeting these understudied yet disease-relevant proteins.

## Abstract 2

Systematic exploration of attachment vectors, linkers and E3 ligase ligands yields a suite of BAZ2 PROTACs, including dual degrader dBAZ2 to the BAZ2B-selective dBAZ2B, with ternary complex formation driving selectivity.

## Graphical abstract

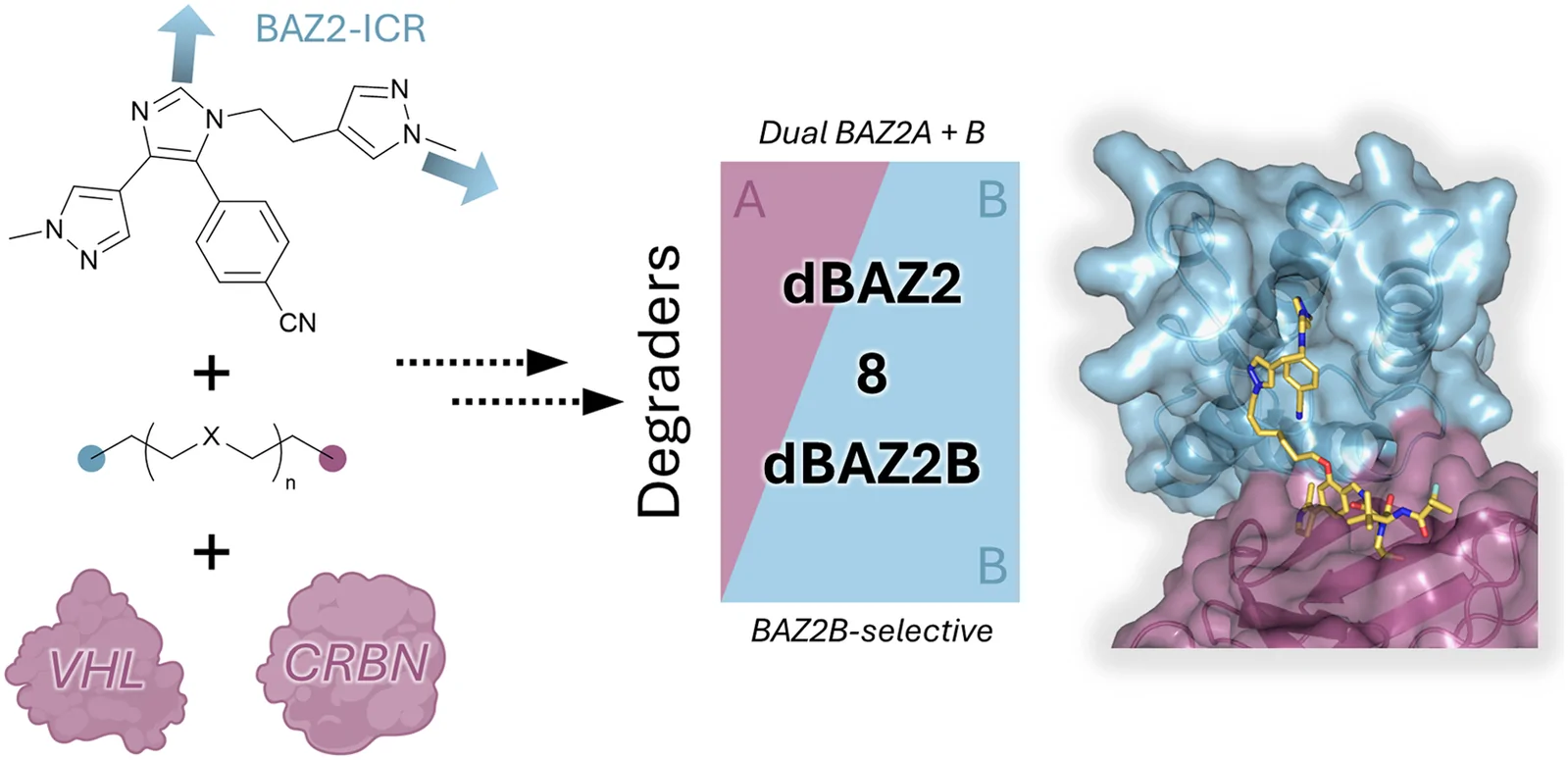

## Introduction

Bromodomain adjacent to zinc finger 2A and 2B (BAZ2A/B) are homologous proteins which directly bind DNA and RNA through their MBD/TAM (methyl-CpG-binding), DDT (DNA binding homeobox and different transcription factors), and PHD (plant homeodomain) domains, and read histone acetylation marks through their bromodomain (BRD).^1^ Both proteins act as regulatory subunits of imitation switch (ISWI) chromatin remodelling complexes, associating with SMARCA1 or SMARCA5 to form the nucleolar remodelling complex 1 and 5 (NoRC-1/5) with BAZ2A, or BRF-1/5 with BAZ2B.^2^ By governing chromatin structure, BAZ2A/B regulate DNA-templated processes such as replication and transcription, with the better studied NoRC particularly associated with the repression of ribosomal DNA (rDNA) transcription and the organization of subnuclear compartments.^3–7^

Both BAZ2A and BAZ2B have been implicated in a wide range of pathologies, including the suppression of liver regeneration and multiple cancers.^8,9^ Nevertheless, despite their structural similarity, numerous studies indicate that BAZ2A and BAZ2B play distinct roles in disease. BAZ2A has been most extensively investigated in prostate cancer (PCa), where its overexpression correlates with poor clinical outcomes,^10^ and it plays an essential role in cancer development, sustaining tumour growth by repressing genes frequently silenced in metastatic PCa through a mechanism dependent on its RNA-binding MBD/TAM domain.^11–13^ BAZ2A has also been shown to play a role in other malignancies, including hepatocellular carcinoma^14,15^ and cervical cancer.^16^ In contrast, the role of BAZ2B in cancer is comparatively underexplored. Still, BAZ2B has been linked to oncogene expression and colony formation in HAP-1 cells,^3^ and the BAZ2B-containing 2q24 locus has been shown to drive childhood hepatoblastoma.^17^ Functional studies directly interrogating BAZ2B function remain limited, with most current knowledge derived from population genetics studies which have associated BAZ2B with asthma,^18^ cardiovascular disease,^19,20^ neurodevelopmental disorders^21–23^ and COVID-19.^24^

Medicinal chemistry campaigns targeting the acetyl–lysine binding BRD have included early fragment screening efforts,^25,26^ and structure-based designs leading to two BAZ2A/B inhibitors, namely BAZ2-ICR and GSK2801.^27,28^ Although these inhibitors show good selectivity over other BRD-containing proteins, achieving selectivity between BAZ2A and BAZ2B remains challenging due to their high similarity (BRD sequence identity = 63%). Moreover, these inhibitors target only the BRD, leaving other critical domains of these large, multifunctional proteins unaffected and thereby limiting their therapeutic potential. To overcome these limitations, we turned to proteolysis targeting chimeras (PROTACs), an emerging therapeutic modality that enables degradation of a protein of interest (POI) by hijacking the endogenous ubiquitin proteasome system (UPS).^29,30^ These heterobifunctional small molecules simultaneously engage the POI and an E3 ligase to form a ternary complex, triggering the poly-ubiquitination and subsequent proteasomal degradation of the POI. In this manner, PROTACs transform a domain-specific binder into a compound capable of inhibiting an entire multi-domain protein. Furthermore, through isoform-specific protein–protein interactions at the ternary complex interface, PROTACs often display enhanced selectivity in comparison to their parent ligands.^31–37^

Recently, we reported two first-in-class PROTACs: dBAZ2, which degrades BAZ2A and BAZ2B, and dBAZ2B, which selectively degrades BAZ2B.^38^ Both PROTACs are derived from BAZ2-ICR and differ only in their attachment point to the von Hippel–Lindau (VHL) E3 ligase ligand VH101. Here, we outline the medicinal chemistry campaign that yielded these first-in-class PROTACs for BAZ2A/B. We describe an optimized synthesis of the BAZ2A/B BRD inhibitor BAZ2-ICR tailored for linker attachment and reveal that BAZ2A/B degradation is more readily achieved through recruitment of VHL than CRBN. We also introduce a third degrader, 8, which employs an alternative exit vector from BAZ2-ICR. Interestingly, while 8 forms ternary complexes with BAZ2A and BAZ2B with equal efficiency, our study suggests that dBAZ2B achieves selectivity through preferential ternary complex formation with BAZ2B.

## Results and discussion

### Design of BAZ2A/B-targeting PROTAC libraries

**Fig. 1:**
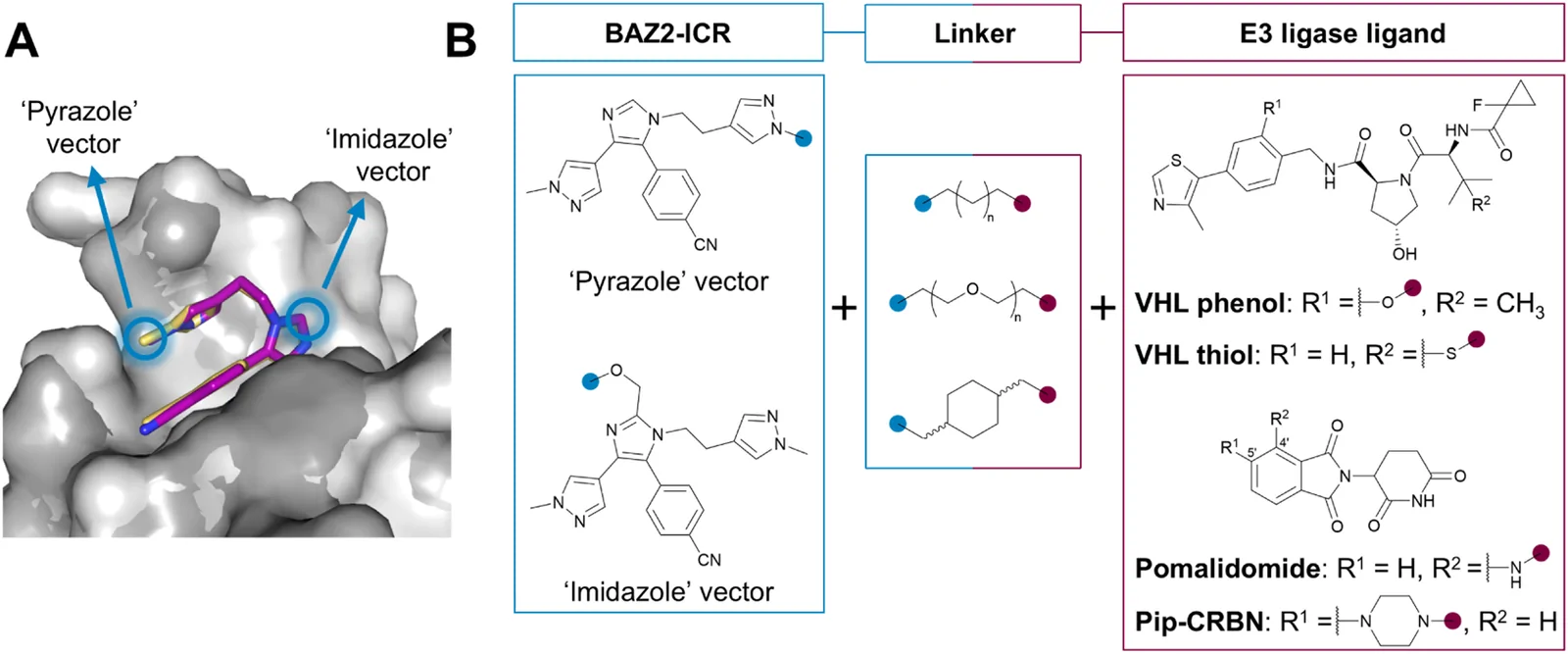
Design of BAZ2A/B-targeting PROTACs. A) Crystal structure of BAZ2-ICR in complex with BAZ2A (PDB: 7BL8,^40^ protein surface pale grey, BAZ2-ICR purple) overlaid with the crystal structure of a BAZ2-ICR analogue in complex with BAZ2B (PDB: 4XUB,^27^ protein surface dark grey, BAZ2-ICR analogue gold). Selected conjugation positions for linker attachment are highlighted. B) General structure and overview of BAZ2A/B-targeting PROTACs synthesized and characterized in this work. **VHL phenol** and **VHL thiol** are derived from VH101,^41^**pip-CRBN** = pomalidomide 5′-piperazine derivative.

BAZ2-ICR, a cell active and potent binder of the BAZ2A/B-BRDs (reported *K*_d_ values: 109 nM [BAZ2A] and 170 nM [BAZ2B]) was chosen as the BAZ2A/B-binding moiety of our PROTACs.^27^ This compound exhibits strong selectivity over other BRD-containing proteins as well as a broad panel of receptors and ion channels.^27,39^ Examination of the crystal structure of BAZ2-ICR bound to BAZ2A (PDB: 7BL8)^40^ and of a close analogue of BAZ2-ICR (7 from Drouin *et al.*) bound to BAZ2B (PDB: 4XUB),^27^ allowed us to identify two possible vectors for linker attachment, namely the methylated pyrazole (hereafter ‘pyrazole vector’) and the C2 position of the central imidazole (hereafter ‘imidazole vector’, Fig. 1A). These positions were exploited to generate structurally diverse BAZ2A/B degrader libraries by conjugating BAZ2-ICR to established ligands of the VHL and Cereblon (CRBN) E3 ligases using a panel of flexible alkylic and polyethylene glycol (PEG) or semi-rigid 1,4-cyclohexane linkers (Fig. 1B).

### Synthesis and characterization of ‘pyrazole–VHL’ PROTACs

**Scheme 1:**
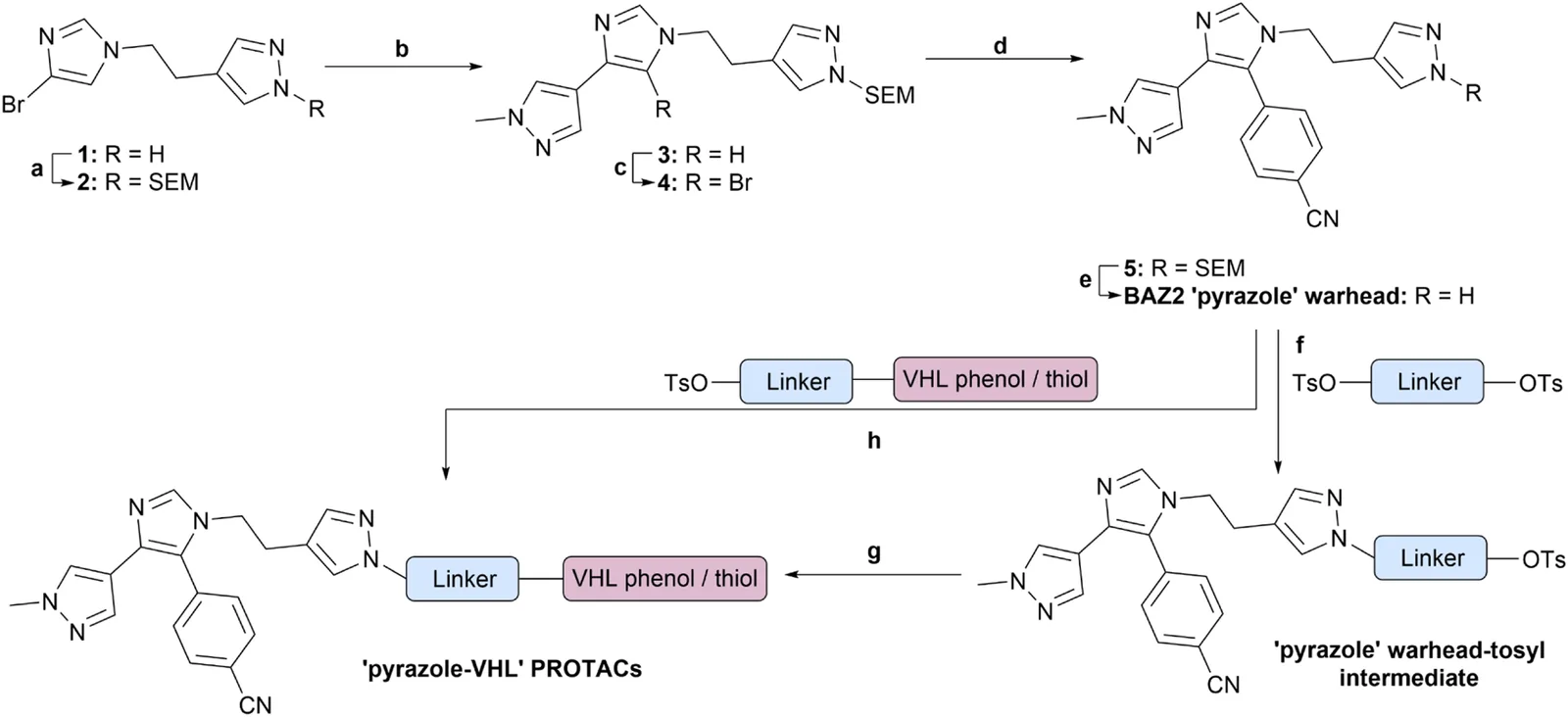
Preparation of a library of ‘pyrazole–VHL’ PROTACs. a. NaH (1.5 equiv.), SEM-Cl (1.4 equiv.), THF (0.5 M), 0 to 20 °C, 14 h, 74%; b. 1-Methylpyrazole-4-boronic acid pinacol ester (1.5 equiv.), Pd(dppf)Cl_2_ (10 mol%), cesium carbonate (3.0 equiv.), 5 : 1 ethanol/water (0.15 M), 120 °C, 4 h, 91% yield; c. *N*-bromosuccinimide (1.0 equiv.), DMF (0.1 M), 0 to 20 °C, 15 h, 71% yield; d. 4-Cyanophenylboronic acid pinacol ester (1.5 equiv.), Pd(dppf)Cl_2_ (10 mol%), cesium carbonate (3.0 equiv.), 5 : 1 1,4-dioxane/water (0.1 M), 120 °C, 4 h, 69% yield; e. TFA/DCM, 20 °C, 4 h, 79% yield; f. Ditosyl-linker (3.0 equiv.), NaH (1.3 equiv.), DMF (0.1 M) 0 to 20 °C, 3 h; g. **VHL phenol** (1.2 equiv.), K_2_CO_3_ (2.0 equiv.), DMF (0.1 M), 80 °C, 4 h/**VHL thiol** (1.25 equiv.), DBU (1.5 equiv.), THF (0.1 M), 22 °C, 12 h; h. **VHL phenol**/**thiol-linker-tosyl intermediate** (1.4 equiv.), NaH (1.3 equiv.), DMF (0.1 M), 0 to 20 °C, 3 h. For detailed synthetic methods and compound characterization, see SI.

First BAZ2-ICR was conjugated through the ‘pyrazole vector’ to two derivatives of the VHL binder VH101 (**VHL phenol** and **VHL thiol**, Fig. 1B).^41^ As detailed in Scheme 1, to access these PROTACs a suitable derivative of BAZ2-ICR was prepared from the corresponding 4-(2-(4-bromo-1*H*-imidazol-1-yl)ethyl)-1*H*-pyrazole.^38^*N*-protection with a SEM group followed by a Pd-catalysed Suzuki cross-coupling reaction with 1-methylpyrazole-4-boronic acid pinacol ester installed the *N*-methyl pyrazole group to produce intermediate 3 in 67% yield over two steps. Selective bromination of the imidazole ring with *N*-bromosuccinimide followed by a second Suzuki coupling with 4-cyanophenylboronic acid pinacol ester delivered 5 in 69% yield. Deprotection of the SEM group under acidic conditions furnished the key intermediate **BAZ2 ‘pyrazole’ warhead** in 79% yield. This compound was subsequently conjugated to a ditosylated linker generating a **‘pyrazole’ warhead-tosyl intermediate** to which the VHL ligand was subsequently appended *via* an S_N_2 reaction with the thiol or phenol moiety. Alternatively, direct incorporation of a tosylated VHL ligand-linker derivative was also implemented in certain cases. In total, 20 compounds were prepared using this strategy as summarized in Table 1. Compounds 6–10 and 16–20 featured flexible alkylic linkers containing 5 to 9 methylene units between the *N*-pyrazole of the warhead and the phenolic (6–10) or thiol (16–20) moieties of the VHL ligand. Similarly, a set of molecules encompassing 1–3 or 5 PEG units were prepared (11–14 and 21–24), again leveraging the two available attachment vectors of VH101, as well as a **VHL thiol** derivative 25 featuring a longer linker composed of 6 PEG units. Finally, 15, a **VHL phenol** derivative bearing a semi-rigid *trans* 1,4-disubstituted cyclohexyl linker, was also synthesized using the same protocol.

**Table 1:** BAZ2A and BAZ2B degradation activity of the ‘pyrazole–VHL’ PROTAC library. Quantification of BAZ2A and BAZ2B by western blot after 16 h treatment of PC3 cells with 2.5 μM ‘pyrazole–VHL’ PROTACs (images in Fig. S1). Protein levels were normalized to Vinculin as a loading control and the percentage remaining calculated relative to DMSO-treated cells run on the same gel. Table entries where >50% protein was degraded are highlighted in grey. n.s. = not synthesized. Mean degradation shown ± standard deviation, calculated from at least two replicates from independent experiments

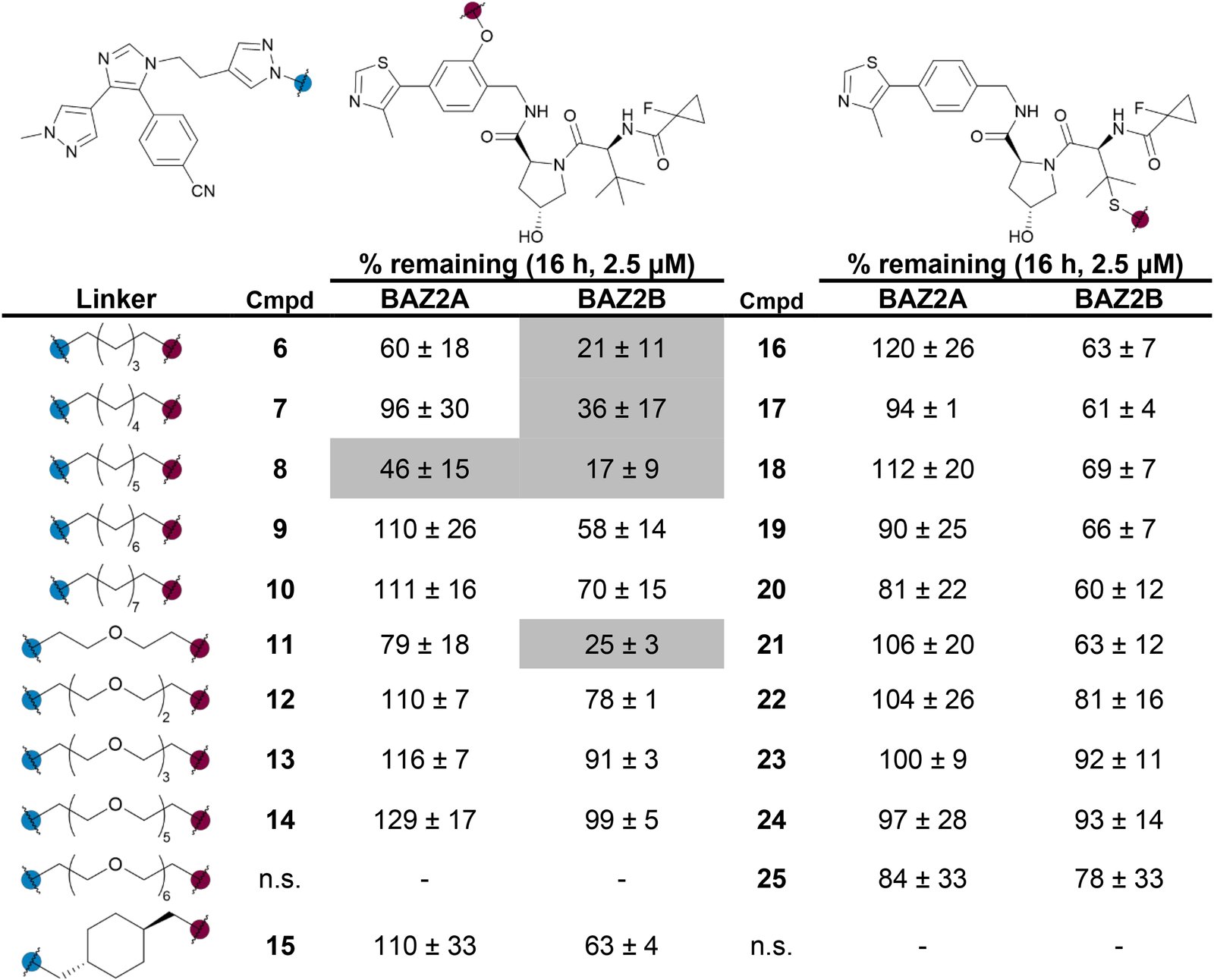

To evaluate the degradation activity of this PROTAC library, we selected the PCa cell line PC3, as BAZ2A is implicated in PCa and we found PC3 cells to have robust and quantifiable expression of the BAZ2A and BAZ2B proteins. Compounds were screened by western blotting of BAZ2A and BAZ2B following a 16 h treatment with 2.5 μM PROTACs (Table 1).

**Fig. 2:**
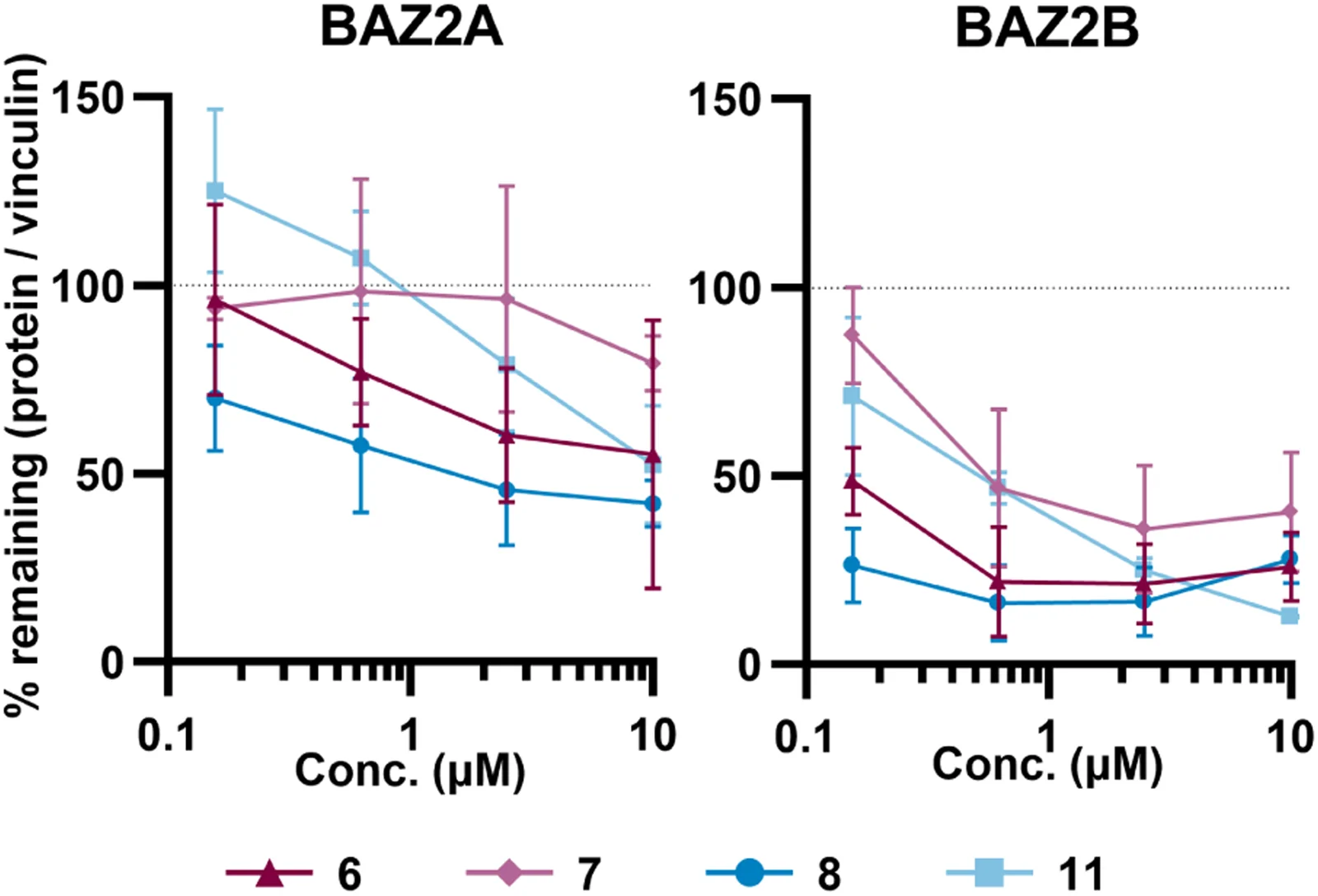
Dose response analysis of lead ‘pyrazole–VHL’ PROTACs. Quantification of BAZ2A and BAZ2B by western blot after 16 h treatment of PC3 cells with lead ‘pyrazole–VHL’ PROTACs (images in Fig. S2). Percentage remaining calculated as for Table 1. Mean degradation shown ± standard deviation, calculated from at least two replicates from independent experiments.

From this library, four PROTACs (6, 7, 8 and 11) exhibited moderate to strong degradation of BAZ2B (>60% degraded), all featuring flexible linkers (5–7 methylene or 1 PEG unit(s)) conjugated through the phenolic linkage to VH101. Interestingly, all compounds showed a preference for degrading BAZ2B over BAZ2A, with 8 being the only compound inducing over 50% degradation of BAZ2A, while also being the strongest degrader of BAZ2B (>80%). Given that PROTACs often show the characteristic “hook effect”, where degradation efficiency is reduced at high PROTAC concentrations due to formation of binary rather than ternary complexes, these four promising candidates were re-screened across a wider range of doses (Fig. 2). In line with the initial screening, 8 was the most potent degrader of both BAZ2A and BAZ2B, showing stronger degradation of BAZ2B (16% remaining protein at 0.6–2.5 μM), compared to BAZ2A (42% remaining protein at 10 μM).

### Analysis of the BAZ2B-BRD:8:VCB ternary complex

**Fig. 3:**
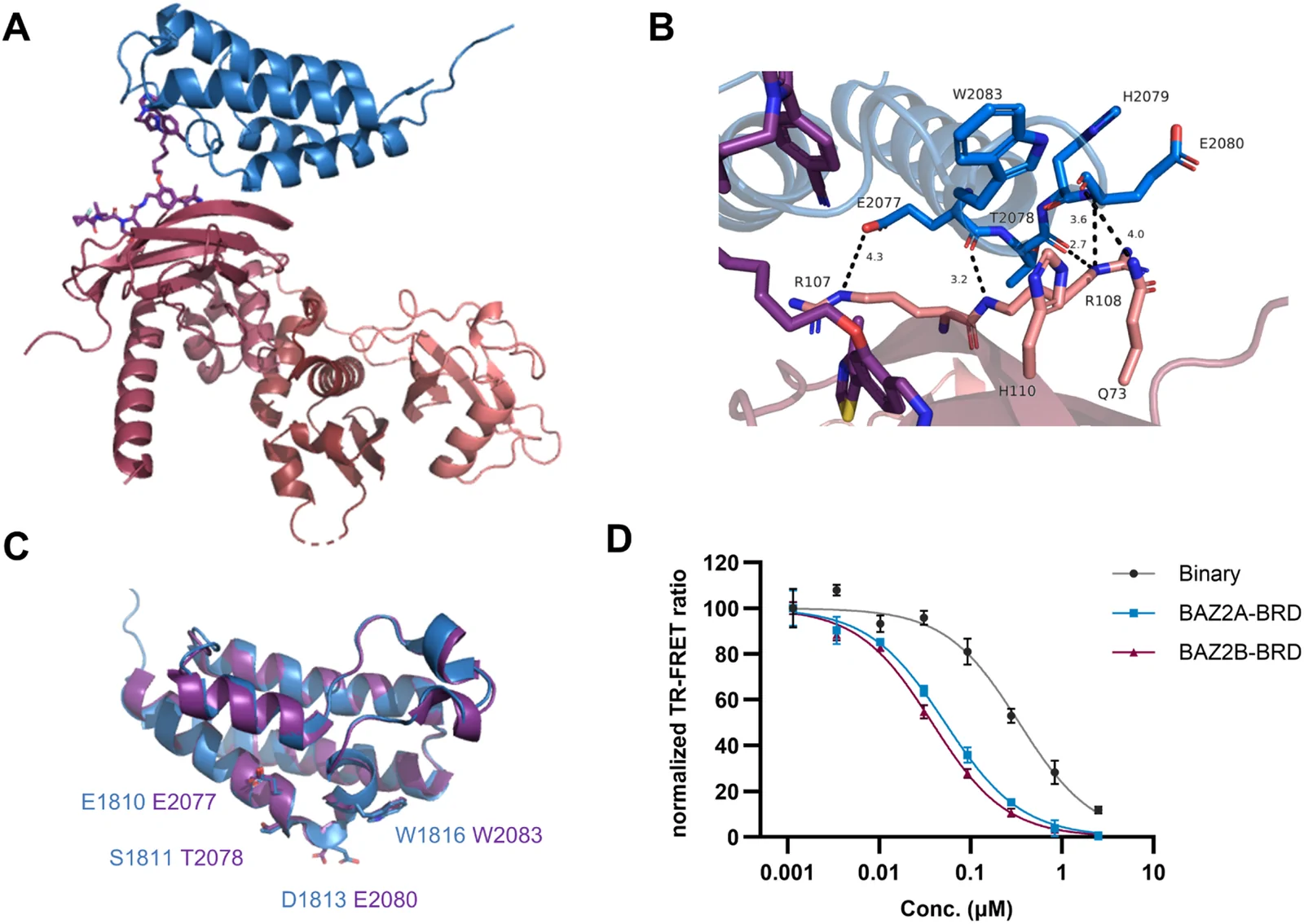
Structure of the BAZ2B-BRD:8:VCB complex. A) The overall shape of the ternary complex. VCB subcomplex (maroon) and BAZ2B-BRD (blue) are shown as cartoon representations, with compound 8 as a stick model (PDB ID: 29HO). B) Residues within bonding distance between BAZ2B-BRD and VCB are shown in stick representation with interatomic distances indicated. C) Alignment of BAZ2A-BRD (blue, PDB ID: 7BL8)^40^ with BAZ2B-BRD (purple, PDB ID: 4XUB).^27^ Residues implicated in VCB binding to BAZ2B and their corresponding amino acids in BAZ2A are highlighted. D) Concentration dependent TR-FRET traces of displacement of a VHL-probe by compound 8 either in a binary complex or in the presence of BAZ2A-BRD or BAZ2B-BRD. Shown are representative traces of a single experiment with two replicates. Data points show the mean ± SD.

As the most active degrader, we selected 8 for further biophysical characterization. Successful crystallization of the BAZ2B-BRD:8:VCB (VHL–ElonginB–ElonginC) ternary complex (Fig. 3A, S3 and Table S1, PDB ID: 29HO) revealed that the BAZ2B-BRD adopts a markedly different orientation relative to VHL than that observed with, for example, BRD4^BD2^ (Fig. S4). As the BAZ2B-BRD points away from the E2 binding site of the CRL2 complex, this orientation may represent one of comparatively low target ubiquitinability.^42^ Detailed inspection of the ternary complex structure revealed several amino acids involved in protein–protein contacts (Fig. 3B). Beyond an H110–W2083 and potential R108–E2077 interaction, most contacts involve the BAZ2B peptide backbone rather than the side-chains (R108–T2078, R107–H2079, R107–E2080). Furthermore, all of the identified amino acids contributing to the VHL:BAZ2B-BRD binding interface are highly conserved between BAZ2A and BAZ2B (Fig. 3C), which is consistent with 8's ability to degrade both BAZ2A and BAZ2B. In TR-FRET displacement binding assays, 8 showed very similar ternary binding affinities with VHL whether in the presence of the BAZ2A- or BAZ2B-BRDs, with both displaying positive cooperativity (*α* ≈ 3, Fig. 3D). Together, our ternary complex structural and biophysical data with compound 8 and the BAZ2A- and BAZ2B-BRDs cannot alone explain the observed preferential degradation of BAZ2B over BAZ2A, suggesting alternative orientations may exist in solution or with the full-length endogenous protein in the cellular environment.

### Synthesis and characterization of ‘pyrazole–CRBN’ PROTACs

**Scheme 2:**
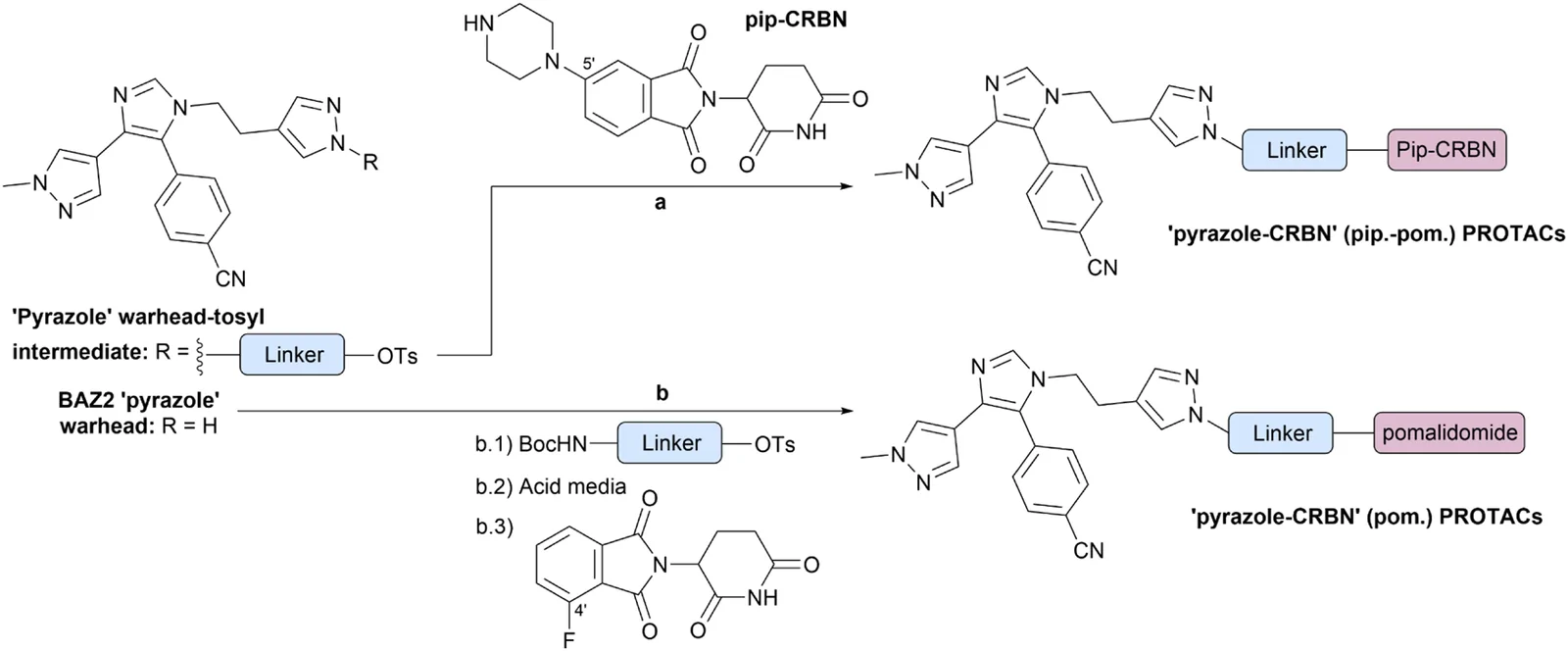
Preparation of a library of ‘pyrazole–CRBN’ PROTACs. a. Pomalidomide 5′-piperazine (**pip-CRBN**) (1.5 equiv.), Et_3_N (3.0 equiv.), DMF (0.1 M), 80 °C, 15 h; b. 1) *N*-Boc-tosyl linker (1.4 equiv.), NaH (1.3 equiv.), DMF (0.1 M), 0 to 20 °C, 3 h; 2) TFA (100 equiv.) or HCl in 1,4-dioxane (4 M, 40 equiv.), DCM (0.1 M), 23 °C, 1–2.5 h; 3) 4-fluorothalidomide (1.2–1.55 equiv.), DIPEA (6.0 equiv.), DMSO (0.2 M), 120 °C, 15 h. For detailed synthetic methods and compound characterization, see SI.

Given that each E3 ligase offers unique surface properties at the ternary complex interface and ubiquitinates distinct positions on the target protein, PROTACs recruiting different E3 ligases frequently exhibit different degradation efficiency and homologue selectivity. Hence, a second library of ‘pyrazole’ linked PROTACs was synthesized using the same *N*-attachment point on BAZ2-ICR but recruiting a different E3 ligase. Inspired by the widespread success of CRBN-recruiting PROTACs, particularly in clinical development,^43^ we employed derivatives of the CRBN ligand pomalidomide for this library (Scheme 2, structures in Table 2). Firstly, a pomalidomide 5′-piperazine derivative (**pip-CRBN**) underwent S_N_2 reactions with a diverse panel of **‘pyrazole’ warhead-tosyl intermediates** to produce PROTACs bearing flexible alkylic linkers with 6, 7, 9 and 10 methylene (26–29) or 2 PEG (30) units between the *N*-pyrazole of the warhead and the piperazine moiety. In parallel, a second batch of PROTACs was prepared in which the linker was directly attached at the C4-position of the pomalidomide phthalimide ring through the aniline vector and in the absence of the piperazine appendage (31–35). Their synthesis started from the **BAZ2 ‘pyrazole’ warhead**, which was reacted under S_N_2 conditions with bifunctional linkers bearing an *N*-Boc-protected amine at one terminus and a tosylate leaving group at the other. The resulting linker-appended pyrazoles were subsequently deprotected under acidic conditions (TFA or HCl in 1,4-dioxane) to afford the corresponding free amines as TFA or hydrochloride salts, respectively. A final S_N_Ar reaction with 4-fluorothalidomide afforded PROTACs bearing linkers with 5 or 8 methylene (31, 32) or 1, 3 and 5 PEG (33–35) units between the *N*-pyrazole of the warhead and the pomalidomide ligand.

**Table 2:** BAZ2A and BAZ2B degradation activity of the ‘pyrazole–CRBN’ PROTAC library. Quantification of BAZ2A and BAZ2B by western blot after 16 h treatment of PC3 cells with 0.63 μM ‘pyrazole–CRBN’ PROTACs (images in Fig. S5). Protein levels were normalized to Vinculin as a loading control and the percentage remaining calculated relative to DMSO-treated cells run on the same gel. Mean degradation shown ± standard deviation, calculated from at least two replicates from independent experiments

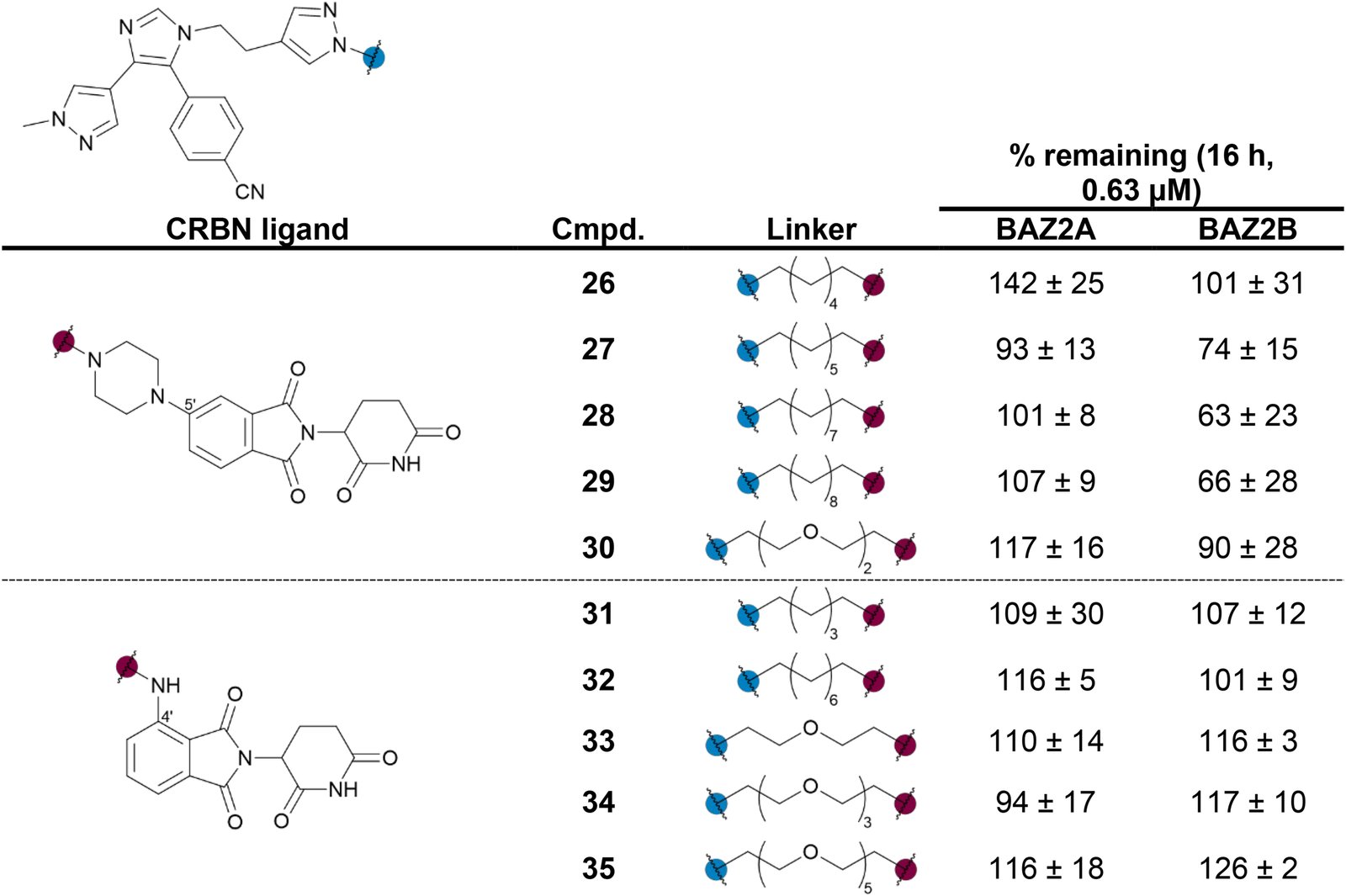

This new library was screened by western blotting of BAZ2A and BAZ2B following a 16 h treatment of PC3 cells, using a lower dose (0.63 μM) than for the VHL library in order to identify PROTACs more potent than 8 (Table 2). Unfortunately, no meaningful degradation of BAZ2A or BAZ2B was observed with any ‘pyrazole–CRBN’ PROTACs under these conditions.

### Synthesis and characterization of ‘imidazole’ PROTACs

**Scheme 3:**
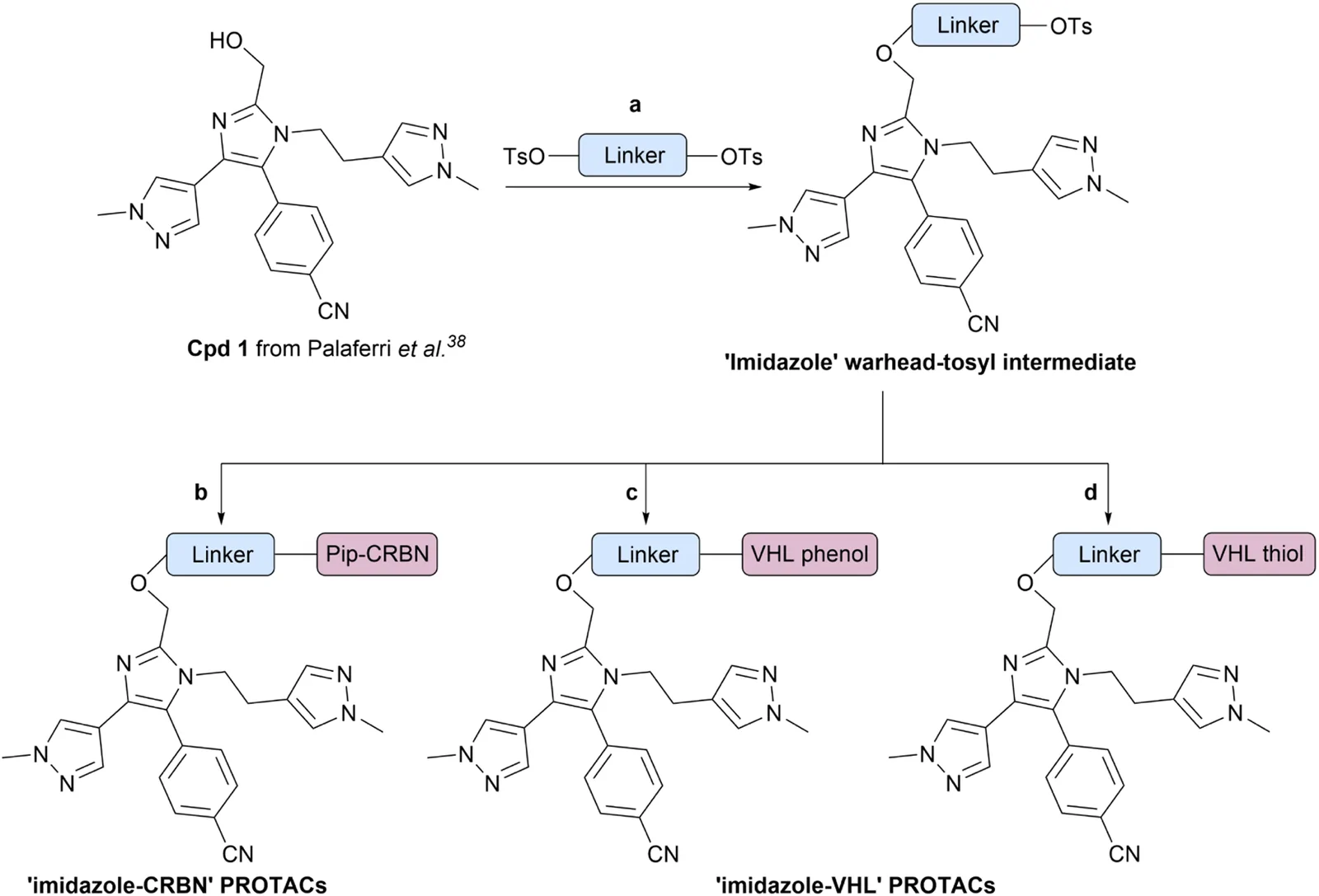
Preparation of a library of ‘imidazole’ PROTACs. a. Ditosyl linker (3.0 equiv.), NaH (1.3 equiv.), DMF (0.1 M), 0 to 20 °C, 3 h; b. Pomalidomide 5′-piperazine (**pip-CRBN**) (1.5 equiv.), NEt_3_ (3.0 equiv.), DMF (0.1 M), 80 °C, 5 h; c. **VHL phenol** (1.2 equiv.), K_2_CO_3_ (2.0 equiv.), DMF (0.1 M), 80 °C, 3–6 h; d. **VHL thiol** (1.35 equiv.), DBU (1.5 equiv.), THF (0.1 M), 22 °C, 15 h. For detailed synthetic methods and compound characterization, see SI.

To expand the ternary complex structural space accessed by our PROTAC libraries, we explored an alternative vector position on BAZ2-ICR, namely the central imidazole ring (Fig. 1).^38^ Previously, we reported that the high affinity of BAZ2-ICR was maintained upon linker attachment at this position and we developed a synthetic route to efficiently deliver BAZ2-ICR with a hydroxymethyl handle installed on the central imidazole to facilitate addition of the linker at this site (Palaferri *et al.* compound 1).^38^ As shown in Scheme 3, to generate a diverse library of ‘imidazole’ PROTACs a panel of ditosyl linkers were first incorporated on the hydroxymethyl handle to afford **‘imidazole’ warhead-tosyl intermediates***via* nucleophilic displacement. Subsequently, these intermediates were subjected to S_N_2 reactions with E3 ligase ligands binding either CRBN (**pip-CRBN**, Fig. 1B) or VHL (**VHL thiol** and **VHL phenol**, Fig. 1B), displacing the tosylate leaving group to deliver the corresponding PROTACs in a straightforward manner. This PROTAC series featured linkers connecting the oxygen atom of the hydroxymethyl handle of the BAZ2-ICR warhead to the corresponding E3 ligand (Table 3). Specifically, PROTACs bearing alkyl linkers of 5 or 8 methylene units (36, 37, 39, 40, 44 and 45) or PEG1 linkers (38, 41 and 46) were synthesized for the CRBN- and both VHL-series. In addition, VHL-recruiting PROTACs incorporating a *trans*- (dBAZ2B and dBAZ2) or *cis*-1,4-disubstituted cyclohexyl linker (42 and 47) were prepared. Finally, a PROTAC bearing a shorter linker composed of 4 methylene units attached to the **VHL thiol** ligand (43) was also synthesized.

**Table 3:** BAZ2A and BAZ2B degradation activity of the ‘imidazole’ PROTAC library. Quantification of BAZ2A and BAZ2B by western blot after 16 h treatment of PC3 cells with 0.25 μM ‘imidazole’ PROTACs (images in Fig. S6). Protein levels were normalized to Vinculin as a loading control and the percentage remaining calculated relative to DMSO-treated cells run on the same gel. Table entries where >50% protein was degraded are highlighted in grey. Mean degradation shown ± standard deviation, calculated from at least two replicates from independent experiments

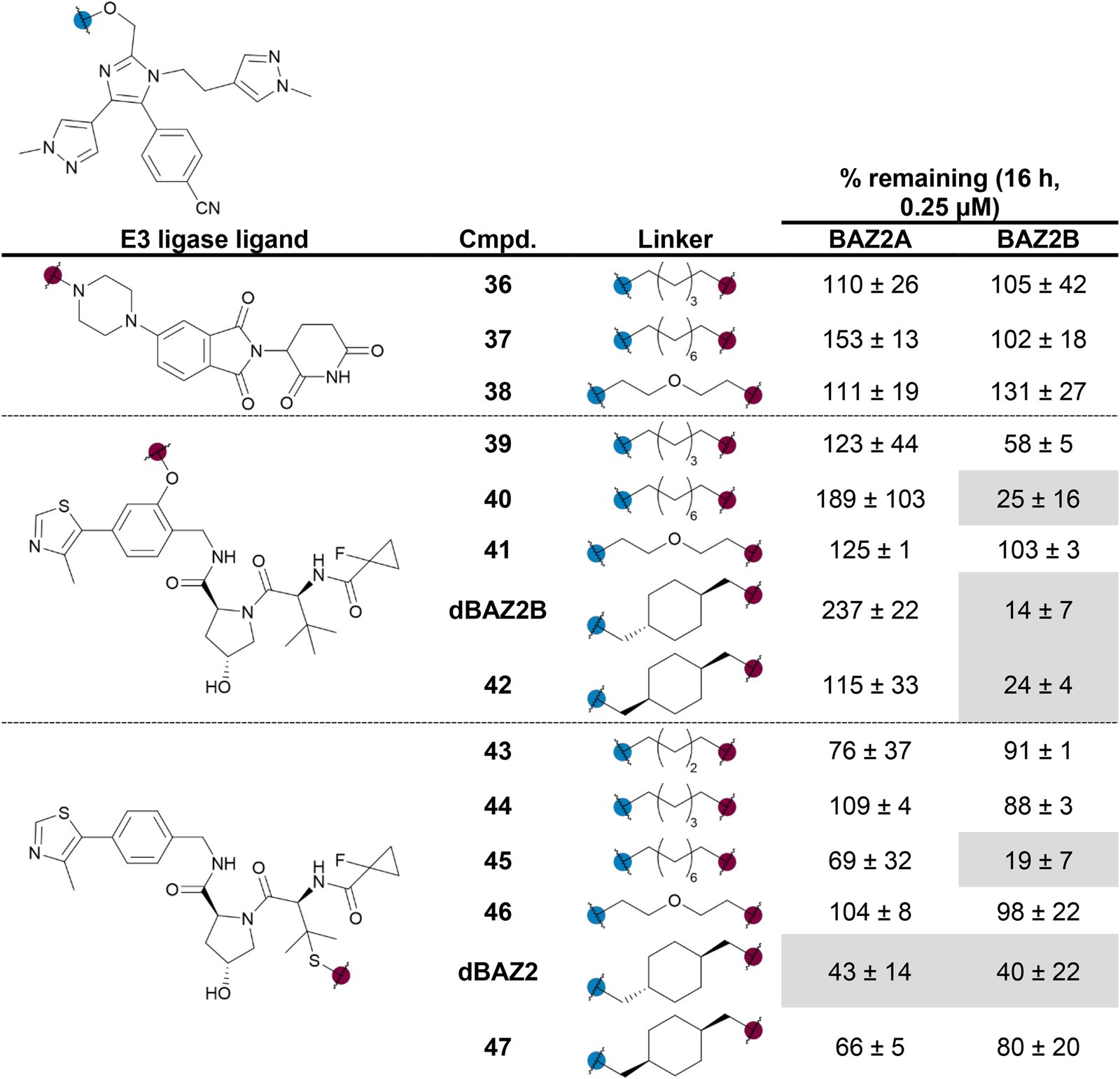

**Fig. 4:**
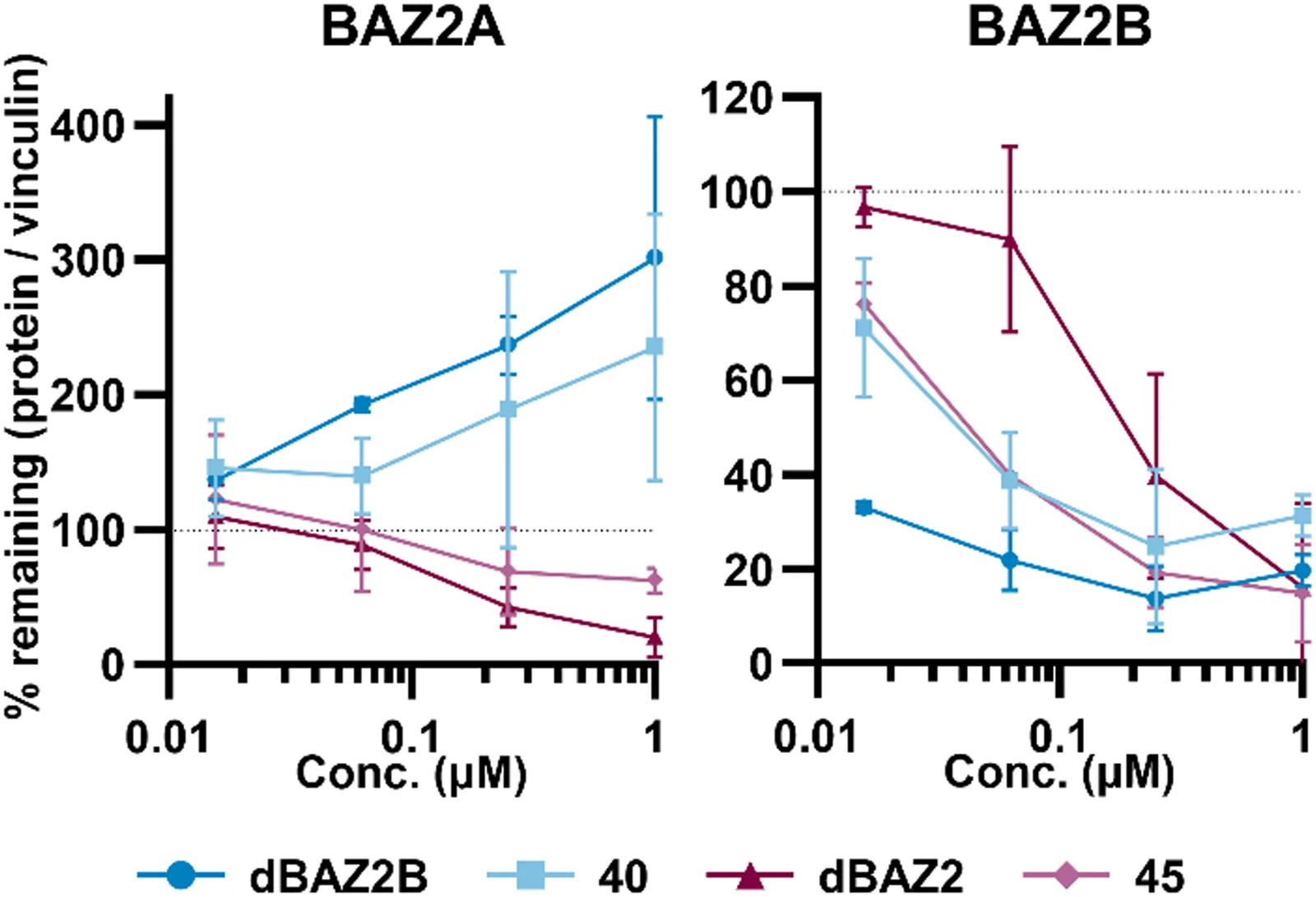
Dose response analysis of lead ‘imidazole’ PROTACs. Quantification of BAZ2A and BAZ2B by western blot after 16 h treatment of PC3 cells with lead ‘imidazole’ PROTACs (images in Fig. S7). Percentage remaining calculated as for Table 3. Mean degradation shown ± standard deviation, calculated from at least two replicates from independent experiments.

This library of ‘imidazole’ PROTACs was screened for BAZ2A and BAZ2B degradation activity, again by treating PC3 cells for 16 h, using an even lower concentration (0.25 μM) to identify only highly potent degraders (Table 3). Promisingly, the imidazole vector in combination with VHL delivered a number of potent degraders (>50% degradation, highlighted in grey in Table 3). Of particular interest, several compounds featuring the phenolic linkage to VH101 showed remarkable selectivity for BAZ2B over BAZ2A. From this initial screening, two selective BAZ2B degraders (40 and dBAZ2B), and two compounds showing degradation of both BAZ2A and BAZ2B (45 and dBAZ2) were chosen for further characterization across a wider concentration range (Fig. 4).

Our characterization revealed dBAZ2 as a moderately potent but dual degrader of both BAZ2A and BAZ2B, and dBAZ2B as a highly potent and selective degrader of BAZ2B. In our previous communication (Palaferri *et al.*)^38^ their degradation activity was fully characterized: dBAZ2 induces BAZ2A/B degradation with a *D*_max_ ≥ 97%, BAZ2A_DC_50_ = 180 nM, BAZ2B_DC_50_ = 250 nM, while dBAZ2B selectively degrades BAZ2B with a *D*_max_ ≥ 97% and DC_50_ = 19 nM. Furthermore, we demonstrated that both compounds induce almost maximal degradation within 2 h which is maintained for at least 3 days. Finally, the mechanism was determined to function through the expected UPS- and VHL-mediated degradation pathway, by comparing the degraders' activity to their respective negative control structural analogues and blocking the activity with a proteasome inhibitor or competitive binders (Fig. S8A and B).

**Fig. 5:**
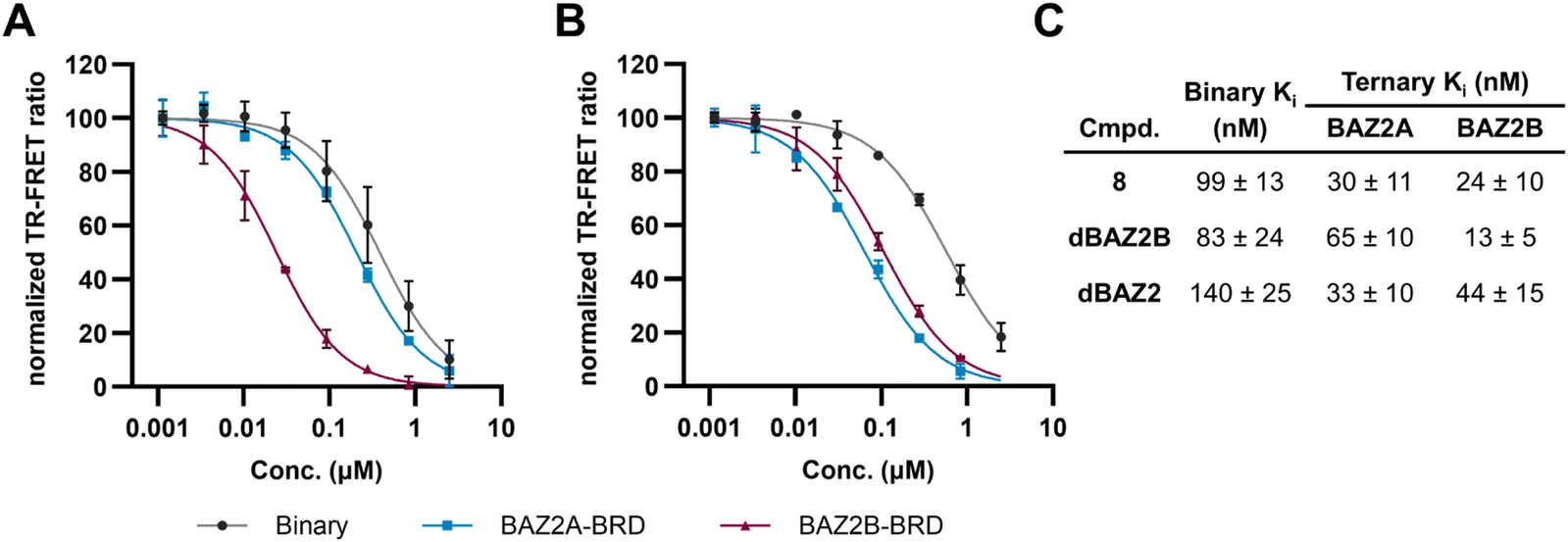
Displacement TR-FRET. Concentration dependent TR-FRET traces of displacement of a VHL-probe by dBAZ2B (A) or dBAZ2 (B) in a binary complex or in the presence of BAZ2A-BRD or BAZ2B-BRD. Shown are representative traces of a single experiment with two replicates. Data points show the arithmetic mean ± SD. C) Affinities of select compounds to VCB either in the presence or absence of BAZ2A/B-BRDs. Values represent the arithmetic mean ± standard error of the mean (SEM) of independent triplicates.

The observed selectivity of dBAZ2B corresponds well with its significantly higher affinity to VCB in the presence of the BAZ2B-BRD compared to with BAZ2A-BRD or to the binary complex alone (Fig. 5A and C), indicating that ternary complex formation contributes to BAZ2B-selective degradation by dBAZ2B. This is in contrast to the dual degrader dBAZ2 which showed similarly improved affinity for VCB in the presence of the BAZ2A- and BAZ2B-BRDs (Fig. 5B and C).

### Exploring the elevation of BAZ2A by dBAZ2B

**Fig. 6:**
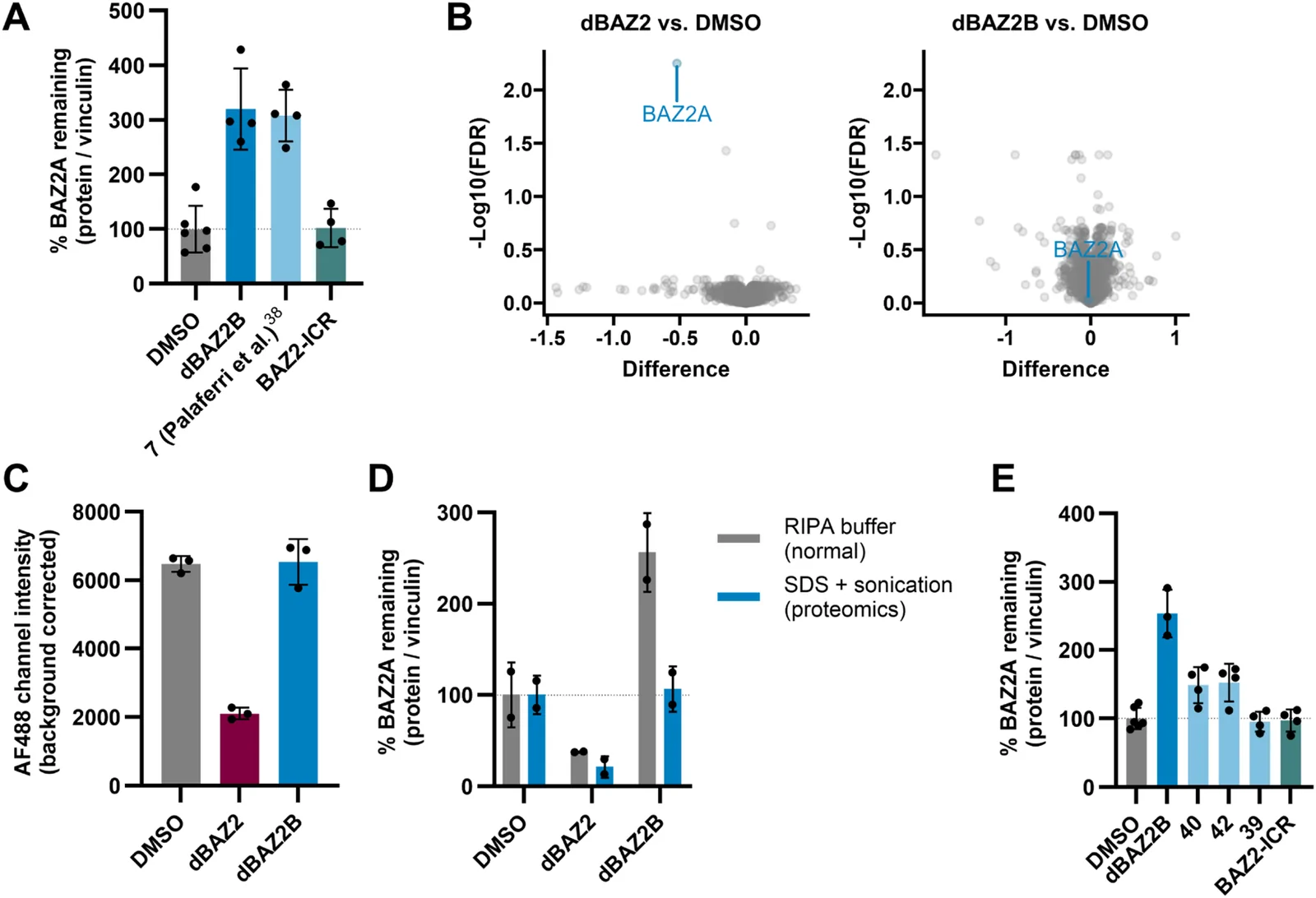
Characterization of the observed elevation in BAZ2A with dBAZ2B. A) Quantification of BAZ2A by western blot after 4 h treatment of MM1S cells with 0.25 μM compounds (images in Fig. S9). For all western blots, BAZ2A protein levels were normalized first to vinculin as a loading control and then the percentage remaining calculated relative to DMSO-treated cells run on the same gel. B) Quantification of the global proteome of MM1S cells treated for 4 h with 1 μM dBAZ2 or 0.25 μM dBAZ2B compared to DMSO-treated cells, BAZ2A highlighted. C) Mean fluorescence staining intensity measured by flow cytometry (one point per sample) of BAZ2A-stained MM1S cells treated for 4 h with 1 μM dBAZ2 or 0.25 μM dBAZ2B, background signal calculated from cells stained with no primary BAZ2A antibody and subtracted from anti-BAZ2A stained cells. D) Quantification of BAZ2A by western blot using different cell lysis protocols (details in Materials and methods) after 4 h treatment of MM1S cells with 1 μM dBAZ2 or 0.25 μM dBAZ2B (images in Fig. S13). E) Quantification of BAZ2A by western blot after 4 h treatment of MM1S cells with 0.25 μM compounds (images in Fig. S14).

Characterization of dBAZ2B by western blotting showed that BAZ2A was not only spared from degradation but was consistently elevated (Table 3, Fig. 4 and Palaferri *et al.*).^38^ Interestingly, its negative control analogue (compound 7 from Palaferri *et al.*),^38^ bearing an inverted stereocenter on the hydroxyproline moiety of the VHL ligand to prevent BAZ2B degradation, also increased BAZ2A, whereas BAZ2-ICR did not affect BAZ2A levels (MM1S cells Fig. 6A, PC3 cells Fig. S8C). This indicates that elevated BAZ2A is not a downstream compensatory consequence of BAZ2B loss, but that the linker and (in)active VHL ligand have additional effects beyond BRD inhibition. Furthermore, dBAZ2B and its negative analogue do not induce expression of *baz2a* mRNA (Fig. S8D), and elevated BAZ2A was also observed with an alternative antibody (Fig. S8E), ruling out regulation of gene expression or epitope specific effects. However, BAZ2A was not elevated in global proteomics after dBAZ2B treatment (MM1S cells Fig. 6B, PC3 cells Palaferri *et al.*),^38^ which employed an alternative method for cell lysis, or by *in situ* staining of fixed cells (flow cytometry with MM1S cells Fig. 6C, immunofluorescence with PC3 cells Fig. S8F). Indeed, while the selective degradation of BAZ2B by dBAZ2B was maintained, elevated BAZ2A could no longer be observed by western blotting of lysates produced using the harsher lysis method employed for proteomics (Fig. 6D, details of lysis protocols in Materials and methods in SI). Finally, this effect was particularly pronounced for dBAZ2B as the most similar PROTACs from our medicinal chemistry campaign, featuring an inverted linker stereochemistry or flexible linker, did not significantly elevate BAZ2A (MM1S cells Fig. 6E, PC3 cells Fig. S8G). Together, these results suggest that the 1,4-*trans*-cyclohexane linker in combination with the VHL ligand in dBAZ2B (and in its negative control) engages a region of BAZ2A beyond the BAZ2-ICR binding site, influencing how readily the protein enters solution upon cell lysis. This may involve changes in tertiary structure, potentially in intrinsically disordered regions, or alterations in interactions with other proteins, DNA, or RNA. While intriguing, these possibilities remain speculative and fall beyond the scope of our study.

### Biological characterization of dBAZ2 and dBAZ2B

**Fig. 7:**
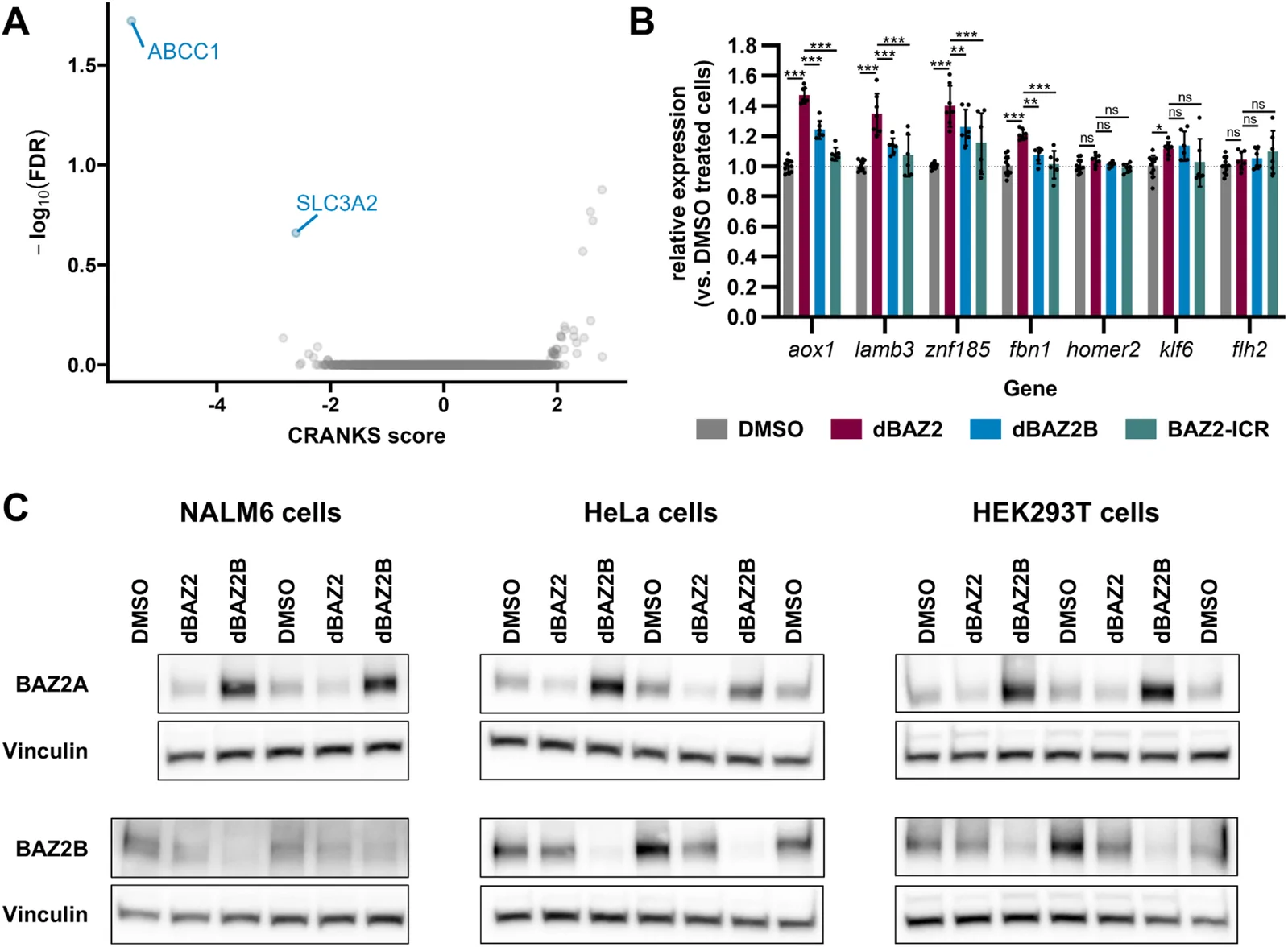
Further biological characterization of dBAZ2 and dBAZ2B. A) CRISPR chemogenomic screening of dBAZ2. Volcano plot showing CRANKS scores and false discovery rate (FDR) obtained from the CRISPR screen in which NALM6 cells were treated for 8 days with 1 μM dBAZ2. Negative scores indicate depleted sgRNAs and positive scores enriched sgRNAs. Proteins mediating resistance to dBAZ2 which have previously been shown to be involved in PROTAC efflux are highlighted in blue. B) Effects of PROTACs and BAZ2-ICR on BAZ2A-regulated genes. mRNA expression after 4 day treatment of PC3 cells with 1 μM dBAZ2/BAZ2-ICR or 0.25 μM dBAZ2B. Gene expression was quantified by RT-qPCR, normalized to *hprt* and then to cells treated with DMSO in the same experimental run. Data from at least three independent biological replicates. Significance calculated *vs.*dBAZ2 treated cells using two-way ANOVA and adjusted using Dunnett's multiple comparisons test: adjusted *p*-value < 0.001 = ***; < 0.01 = **; < 0.05 = *; > 0.05 = ns. C) The activity and selectivity of dBAZ2 and dBAZ2B is maintained across a panel of cell lines. BAZ2A and BAZ2B western blot images after 4 h treatment of the named cell lines with 1 μM dBAZ2 or 0.25 μM dBAZ2B. Vinculin included for comparison as a loading control.

To identify genetic determinants of sensitivity and resistance to dBAZ2, a genome-wide CRISPR/Cas9 knockout chemogenomic screen was performed in NALM6 cells using the ChemoGenix platform at the University of Montreal.^44^ No genes were significantly enriched (−log_10_(FDR) > 1), likely as dBAZ2 did not have an anti-proliferative effect under the experimental conditions used. Notably, however, the top two scoring genes mediating resistance were the ABC transporter ABCC1 and the membrane transporter component SLC3A2 (Fig. 7A). Recently, these two proteins were demonstrated to mediate the export of a metabolite of the BET-targeting PROTAC MZ1, contributing to resistance to MZ1 and similarly to other PROTACs in human cancer cells.^45^ Together, these findings suggest that dBAZ2 might be substantially effluxed, although this does not preclude robust protein degradation.

To validate their use as tool compounds, we also explored the effect of dBAZ2 and dBAZ2B on gene expression. BAZ2A has been shown to regulate transcription in cell line models of PCa,^10,12^ and, consistent with this, dBAZ2 significantly upregulated five of seven tested genes which were previously identified as BAZ2A-regulated (Fig. 7B). BAZ2A degradation likely significantly contributed to this upregulation, as dual BAZ2A and BAZ2B degradation by dBAZ2 had greater effects than either BAZ2B-selective degradation by dBAZ2B or treatment with the BAZ2A/B-BRD inhibitor BAZ2-ICR. These results emphasize that, for biological studies of BAZ2A or BAZ2B, effective chemical tools must target the full-length protein rather than a single functional domain and achieve selectivity between these two close homologues.

Previously, we showed that dBAZ2 is a dual degrader of both BAZ2A and BAZ2B, and dBAZ2B a selective BAZ2B degrader in both the PCa cell line PC3 and the multiple myeloma MM1S cell line.^38^ Additional screening of these compounds confirmed that this specificity is maintained across a wide range of cellular backgrounds, including acute lymphoblastic leukaemia NALM6 cells, as well as the widely used HeLa and HEK293T cell lines, highlighting the wide applicability of these compounds as chemical tools (Fig. 7C).

## Conclusions

Until recently, functional studies of BAZ2A and BAZ2B were limited by a lack of tool compounds that are either homologue-selective or target functional domains beyond the BRD. We addressed this gap with two first-in-class degraders derived from the BAZ2A/B-BRD inhibitor BAZ2-ICR: dBAZ2, a dual BAZ2A/B degrader, and dBAZ2B, a BAZ2B-selective PROTAC. Here, we detail the medicinal chemistry campaign behind these compounds, exploring two linker attachment positions on BAZ2-ICR (‘pyrazole’ and ‘imidazole’) and recruitment of VHL and CRBN E3 ligases. The synthetic routes we developed to access conjugatable BAZ2-ICR derivatives provide a foundation for future campaigns to create efflux-resistant dBAZ2 analogues, the first BAZ2A-selective degraders, or heterobifunctionals for induced proximity modalities beyond degradation.

All active PROTACs recruited VHL, indicating that achieving CRBN-driven degradation of BAZ2A/B may require broader exploration of chemical space, while the inactivity of the CRBN-recruiting compounds may reflect several challenging factors in degrader development, such as ternary complex formation, cellular permeability or compound stability. Notably, a phenolic linkage to the VHL ligand was required when using the ‘pyrazole’ vector, yielding a third degrader, 8. Compound 8 displayed similar cooperativity with BAZ2A and BAZ2B, likely stemming from structural features in the BAZ2B-BRD:8:VCB interface being conserved with BAZ2A, despite 8 slightly favouring BAZ2B degradation in our initial screening, suggesting that BAZ2B is intrinsically more amenable to downstream degradation. Additionally, the unusual orientation of the BAZ2B-BRD relative to VHL in the ternary complex, where the BRD is pointing away from the E2-ubiquitin catalytic site, suggests that target protein ubiquitination may also occur beyond the BRD.

Both dBAZ2 and dBAZ2B use the ‘imidazole’ vector of BAZ2-ICR but differ in their VHL ligand linkage. Like 8, dBAZ2B employs a phenolic linkage which thus appears to generally favour BAZ2B. However, in contrast to 8, dBAZ2B shows preferential ternary complex formation with BAZ2B, likely contributing to its enhanced selectivity. Interestingly, while dBAZ2B does not degrade BAZ2A, it nonetheless elicited effects on this protein beyond BRD inhibition. Altered protein solubilization in western blot analysis indicates that dBAZ2B affects BAZ2A structure or interactions, highlighting that these large molecules can produce unexpected functional outcomes.

Collectively, dBAZ2 and dBAZ2B are powerful, versatile chemical tools for probing BAZ2 biology. The selective modulation of BAZ2A-regulated genes by dBAZ2—but not by dBAZ2B or BAZ2-ICR—underscores the importance of whole-protein targeting and homologue selectivity, which conventional BRD inhibitors cannot achieve. Their consistent activity across diverse cell lines demonstrates their broad utility, providing a toolkit for future studies to uncover the biological roles of these understudied proteins and their contributions to disease.

## Experimental section

See SI for synthetic routes and chemical characterization of all PROTACs and all experimental methods for the biological characterization of PROTAC activity.

### Synthesis and characterization of compound 8

**BAZ2 ‘pyrazole’ warhead** (Scheme 1, 30 mg, 0.0874 mmol, 1.0 equiv.) and NaH (1.3 equiv.) were suspended in dry DMF (0.1 M) and the resulting suspension stirred at 0 °C for 1 hour. Then, a solution of ditosyl linker heptane-1,7-diyl bis(4-methylbenzenesulfonate)^46^ (3.0 equiv.) was added and the reaction mixture stirred for 3 hours and allowed to warm up to 20 °C. After this time, the reaction was quenched with water, extracted with DCM and the organic layer washed with brine, dried over anhydrous MgSO_4_, filtered and the solvent removed under reduced pressure. The crude product was purified by flash chromatography (1% to 8% MeOH in DCM) to afford the corresponding ‘pyrazole’ warhead-tosyl intermediate S2 (39 mg, 73%, white solid). ^1^H NMR (400 MHz, CD_2_Cl_2_) *δ* 7.78–7.73 (m, 4H), 7.44 (s, 1H), 7.43–7.39 (m, 2H), 7.38–7.36 (m, 1H), 7.36–7.34 (m, 1H), 7.32 (s, 1H), 7.25 (d, *J* = 0.7 Hz, 1H), 7.02 (s, 1H), 6.92 (s, 1H), 4.00–3.90 (m, 6H), 3.78 (s, 3H), 2.65 (t, *J* = 7.0 Hz, 2H), 2.44 (s, 3H), 1.75–1.67 (m, 2H), 1.63–1.55 (m, 2H), 1.29–1.16 (m, 6H). ^13^C NMR (101 MHz, CD_2_Cl_2_) *δ* 145.31, 138.51, 138.02, 136.79, 135.92, 133.41, 133.14, 131.62, 130.21, 128.13, 127.90, 127.46, 126.11, 125.19, 118.84, 117.06, 116.27, 112.58, 71.11, 52.31, 46.93, 39.11, 30.61, 29.01, 28.72, 26.63, 26.29, 25.50, 21.73. HRMS (ESI): *m*/*z* calcd for C_33_H_38_N_7_O_3_S [M + H]^+^: 612.27530; found 612.27528.

‘Pyrazole’ warhead-tosyl intermediate S2 (13 mg, 0.0212 mmol, 1.0 equiv.), **VHL phenol**^47^ (13.6 mg, 0.0255 mmol, 1.2 equiv.), and K_2_CO_3_ (2.0 equiv.) were dissolved in dry DMF (0.1 M). The reaction mixture was stirred at 80 °C for 4 h. After this time, the organic solvent was removed under reduced pressure and the resulting residue triturated with Et_2_O. The crude mixture was purified by preparative TLC (2 : 2 : 1 EtOAc : DCM : MeOH) followed by trituration with Et_2_O and pentane to afford the corresponding VHL phenol-based ‘pyrazole’ PROTAC 8 (5 mg, 24%, white solid). ^1^H NMR (500 MHz, CD_2_Cl_2_) *δ* 8.66 (s, 1H), 7.77 (d, *J* = 8.2 Hz, 2H), 7.45–7.38 (m, 3H), 7.36 (s, 1H), 7.33 (d, *J* = 7.7 Hz, 1H), 7.29–7.23 (m, 2H), 7.03–6.99 (m, 2H), 6.96 (d, *J* = 7.7 Hz, 1H), 6.91 (d, *J* = 8.0 Hz, 2H), 4.63 (t, *J* = 7.6 Hz, 1H), 4.57 (d, *J* = 8.9 Hz, 1H), 4.55–4.45 (m, 2H), 4.36 (dd, *J* = 14.8, 5.2 Hz, 1H), 4.07–3.95 (m, 4H), 3.94 (t, *J* = 6.8 Hz, 2H), 3.86 (d, *J* = 11.0 Hz, 1H), 3.77 (s, 3H), 3.65 (dd, *J* = 11.0, 4.2 Hz, 1H), 2.63 (t, *J* = 6.8 Hz, 2H), 2.50 (s, 3H), 2.41 (ddd, *J* = 12.6, 7.4, 4.9 Hz, 1H), 2.07–1.98 (m, 1H), 1.85–1.71 (m, 4H), 1.54–1.44 (m, 2H), 1.45–1.33 (m, 2H), 1.34–1.18 (m, 6H), 0.93 (s, 9H). ^13^C NMR (126 MHz, CD_2_Cl_2_) *δ* 171.22, 170.85, 170.00 (d, *J* = 20.2 Hz), 157.34, 150.48, 148.99, 138.54, 138.23, 136.86, 135.83, 133.38, 133.22, 132.73, 132.16, 131.70, 129.89, 128.13, 127.68, 126.83, 125.30, 121.63, 118.84, 116.87, 116.34, 112.77, 112.49, 78.72 (d, *J* = 232.2 Hz), 70.44, 68.50, 59.09, 57.62, 56.82, 52.48, 47.02, 39.20, 39.14, 36.81, 35.86, 30.78, 29.46, 29.17, 26.89, 26.44, 26.13, 16.38, 13.88 (d, *J* = 10.3 Hz), 13.70 (d, *J* = 10.2 Hz). ^19^F NMR (471 MHz, CD_2_Cl_2_) *δ* −197.95. HRMS (ESI): *m*/*z* calcd for C_52_H_63_FN_11_O_5_S [M + H]^+^: 972.47068; found 972.47067.

### PROTAC screening by western blot in PC3 cells

PC3 cells were cultured in RPMI 1640 (Gibco 21875034) supplemented with 10% FBS (Gibco A5256701) and penicillin/streptomycin (Gibco 15140). Cells were maintained in a humidified incubator with 5% CO_2_ at 37 °C. The identity of the PC3 cells was authenticated by STR profiling (performed by Microsynth).

For PROTAC screening by western blotting (Tables 1–3, Fig. 2 and 4), 2.4 × 10^5^ PC3 cells per well on 6 well plates were plated 24 h prior to compound treatment. Cells were treated with compounds for 16 h at a final concentration of 0.1% DMSO. Following treatment, cells were washed in ice cold PBS and then lysed on ice with RIPA buffer (50 mM tris pH 7.5, 2 mM EDTA, 150 mM NaCl, 0.5% sodium deoxycholate, 1% Triton X-100) supplemented with cOmplete™ protease inhibitor cocktail (Roche) by scraping and resuspension by pipetting, before the lysates were clarified by centrifugation. Protein concentrations of the cell lysates were determined using the Pierce™ BCA Protein Assay Kit (Thermo Scientific, 23227).

Equal amounts of protein (20–30 μg) were separated on AccuPAGE Bis-Tris 4–12% gradient gels (Bio Bench AP-GPB-41215) and transferred to PVDF membranes. The membranes were blocked with 5% milk (Merck Millipore 115363) in PBS-T and proteins were stained with the following antibodies diluted in 5% milk at 4 °C overnight: for BAZ2A, anti-BAZ2A/TIP5 [EPR25419-10] (Abcam ab290639) diluted 1 : 1000; for BAZ2B, anti-BAZ2B (Atlas Antibodies HPA059292) diluted 1 : 500; for vinculin, used as a loading control, anti-vinculin [42H89L44] (Invitrogen 700062) diluted 1 : 1000. For detection, membranes were incubated for 1 hour at room temperature with HRP-conjugated secondary antibody (Southern Biotech, anti-rabbit 6415-05, 1 : 5000 dilution), and were imaged using SuperSignal™ West Pico PLUS Chemiluminescent Substrate (Thermo Scientific 34580) and the Vilber Fusion-FX7 imager. Bands were quantified using ImageJ software and protein levels were normalized to protein from DMSO-treated cells treated in parallel and run on the same gel.

## Author contributions

(I. C.-S. and K. A. G.) contributed equally as lead authors. (L. P. and M. K.) contributed equally. I. C.-S. and L. P. synthesized the compounds, and I. C.-S., L. P. and E. L. performed the chemical characterization. K. A. G. and L. P. performed the cellular assays, and M. K. solved the cocrystal structure and performed the biophysical assays. I. C.-S., K. A. G., L. P. and C. N. conceived the work, I. C.-S., K. A. G., M. K., L. P. and C. N. designed and interpreted experiments. I. C.-S., K. A. G., L. P. and M. K. performed the data curation, analysis and visualization and prepared the original draft. All authors reviewed and edited the manuscript. A. C. and C. N. supervised the project and provided resources. All authors read and agreed to the final version of the manuscript.

## Conflicts of interest

A. C. is a scientific founder and shareholder of Amphista Therapeutics, a company that is developing targeted protein degradation therapeutic platforms, and is on the Scientific Advisory Board of ProtOS Bio and TRIMTECH Therapeutics. The Ciulli Laboratory receives or has received sponsored research support from Almirall, Amgen, Amphista Therapeutics, Boehringer Ingelheim, Eisai, Merck KGaA, Nurix Therapeutics, Ono Pharmaceutical, and Tocris-Biotechne. All other authors have no conflicts of interest to declare.

## Supplementary Material

MD-017-D6MD00244G-s001

## Data Availability

Atomic coordinates and structure factors for the BAZ2B-BRD:8:VCB complex structure have been deposited in the Protein Data Bank (PDB) under accession number 29HO. Supplementary information (SI): full synthetic procedures and characterization (NMR, HRMS) for all PROTACs and intermediates, and additional biological, biophysical and structural data (Fig. S1–S14, Table S1) supporting the main text. See DOI: https://doi.org/10.1039/d6md00244g.
