# Supplementary material for: Chemical tools for BAZ2A/B biology: discovery of dual and BAZ2B-selective PROTAC degraders
Source: RSC Med Chem. 2026 Aug 20. Online ahead of print. doi: 10.1039/d6md00244g (PMC13492239; doi:10.1039/d6md00244g)
Supplement: Supplementary file 1 [file MD-OLF-D6MD00244G-s001.pdf]

Supplementary Information (SI) for RSC Medicinal Chemistry.
This journal is © The Royal Society of Chemistry 2026

Chemical Tools for BAZ2A/B Biology: Discovery of Dual and BAZ2B-Selective PROTAC Degraders

Iván Cheng-Sánchez,[a, b †] *Katherine A. Gosselé,*[a †] *Leonardo Palaferri,*[a $]

Michael Krummhaar,[c $] *Eleen Laul,*[a] *Alessio Ciulli,*[*c] *Cristina Nevado*[*a]

[a] Department of Chemistry, University of Zurich, Winterthurerstrasse 190, CH-8057 Zurich, Switzerland

[b] Current address: Department of Organic Chemistry, Faculty of Sciences, University of Málaga, 29071 Málaga, Spain

[c] Centre for Targeted Protein Degradation, School of Life Sciences, University of Dundee, 1 James Lindsay Place, DD1 5JJ, Dundee, UK

[†] Contributed equally as lead authors

[$] Contributed equally

*Corresponding Authors

Supporting Information

Table of Contents

Supplementary Figures and Tables 2

Materials and Methods 20

General synthetic procedures 28

Experimental Procedures and Compound Characterization 34

^{1}H, ^{13}C and ^{19}F NMR spectra 77

LCMS Spectra 168

References 189

Supplementary Figures and Tables

Notes regarding images from western blot experiments (Fig. S1-2, S5-7, S9-11, S13-15):

Where compounds from different figures were measured on the same gel, DMSO lanes are shown multiple times across different figures. Membranes are numbered to clarify where repeated data is shown. In the cases where a measurement was included in both the initial screening and dose response quantifications, the image is either duplicated and shown in both figures or the figure refers to another where the image is shown.

In all figures, lanes from the same membrane and stained for the same protein are shown in a solid box. Grouped are the same membrane stained for different proteins (either by cutting the membrane or stripping and re-blotting). Dashed lines indicate where lanes not relevant to the figure have been cropped out.

Membrane 1:

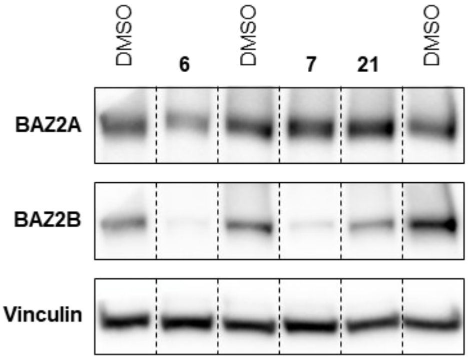

Membrane 2:

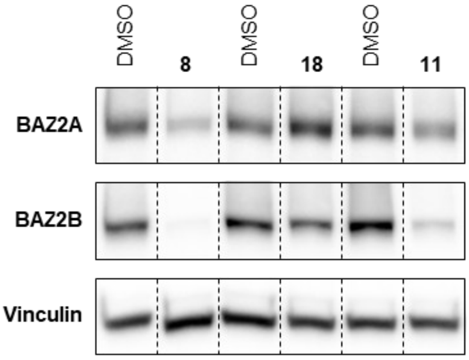

Membrane 3:

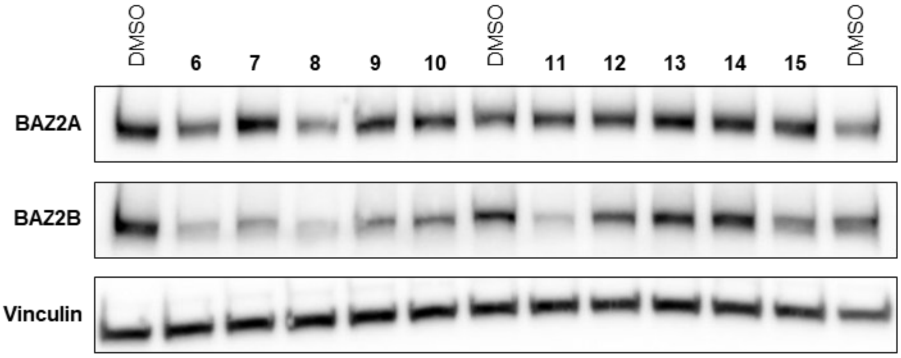

Membrane 4:

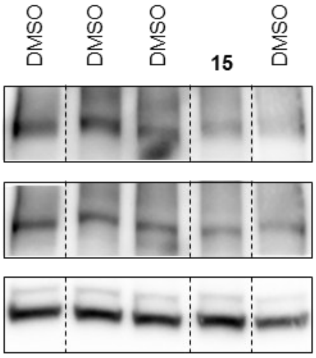

Membrane 5:

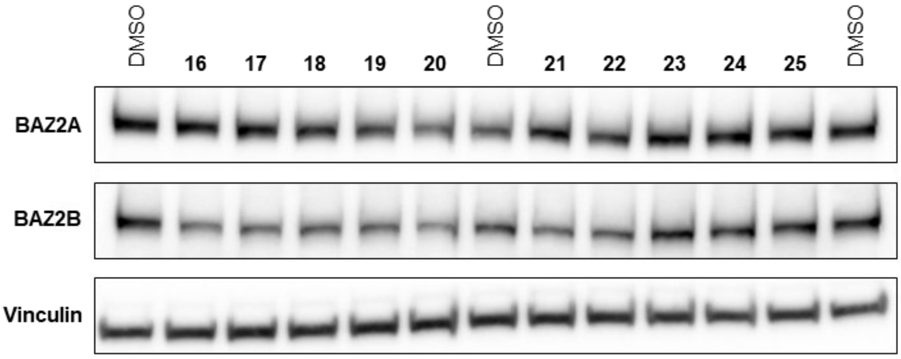

Membrane 6:

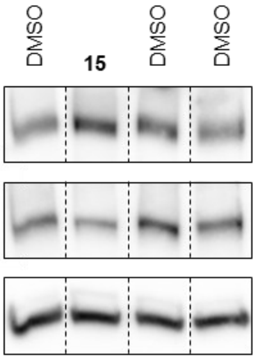

Membrane 7:

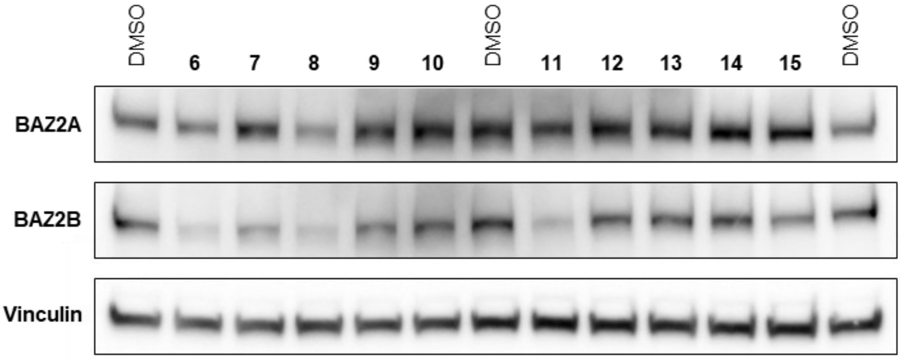

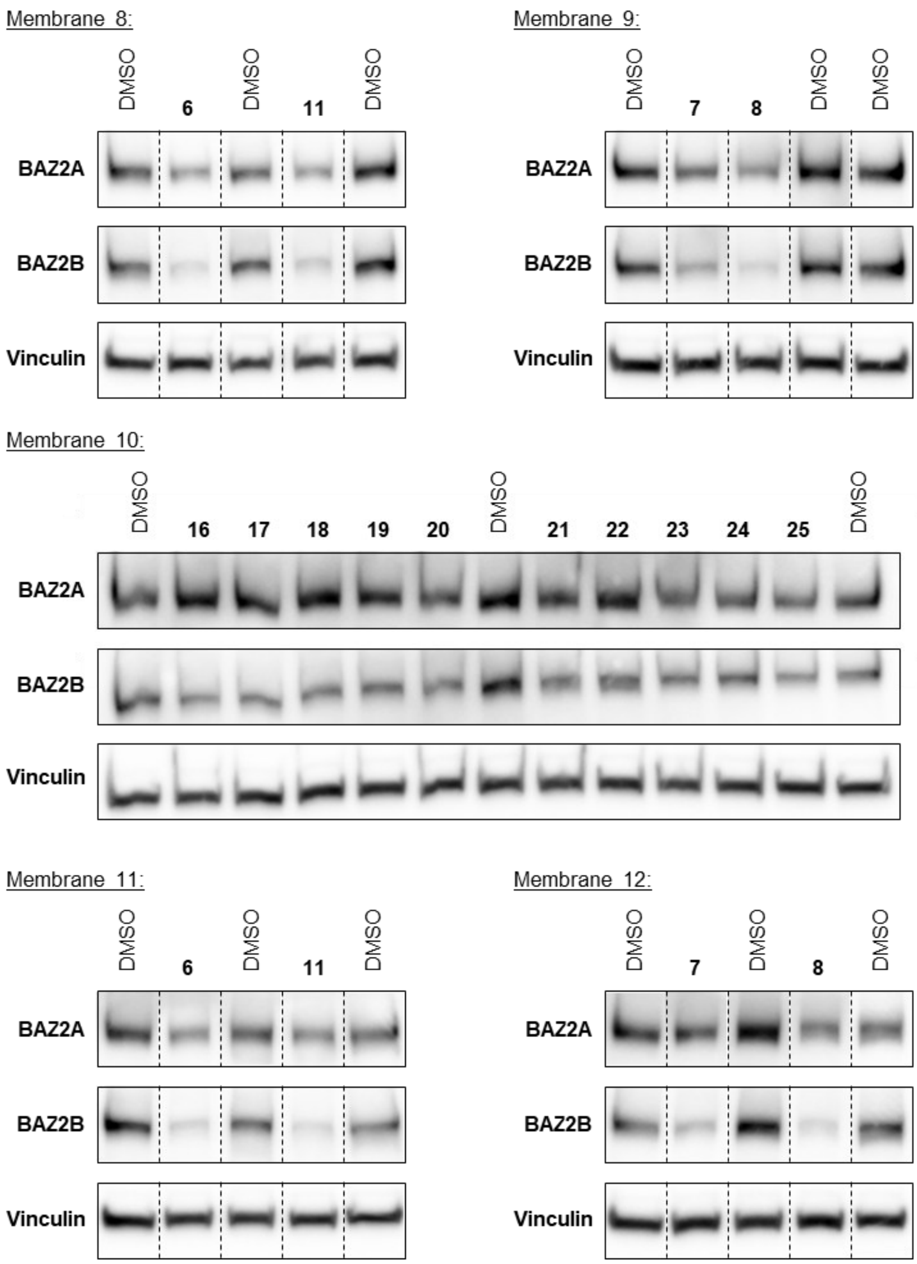

Fig. S1. [continued from previous page] Images of western blots used for quantification in Table 1: 'pyrazole-VHL' PROTAC screening.

Membrane 1:

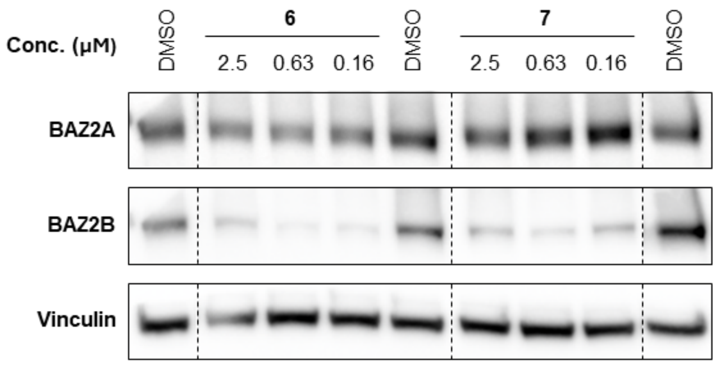

Also included replicates at 2.5 µM for compounds **6**, **7**, **8** and **11** from membranes 3 and 7 (see Fig. S1)

Membrane 2:

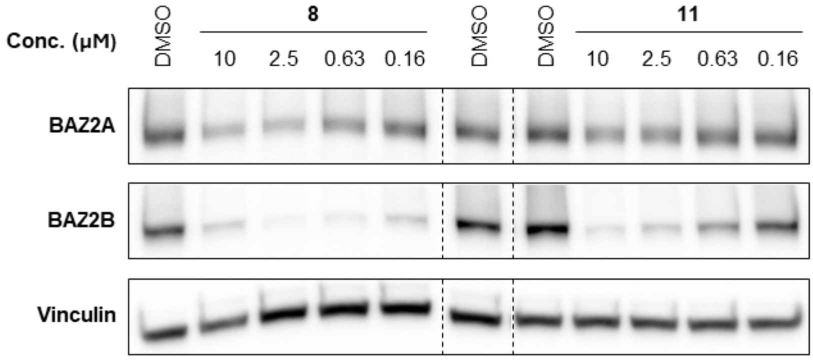

Membrane 8:

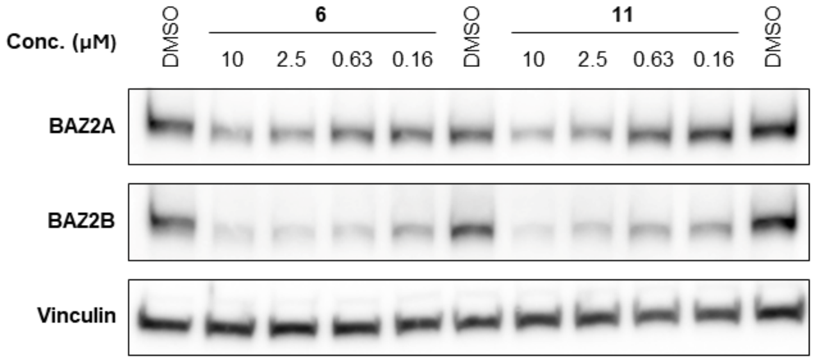

Membrane 9:

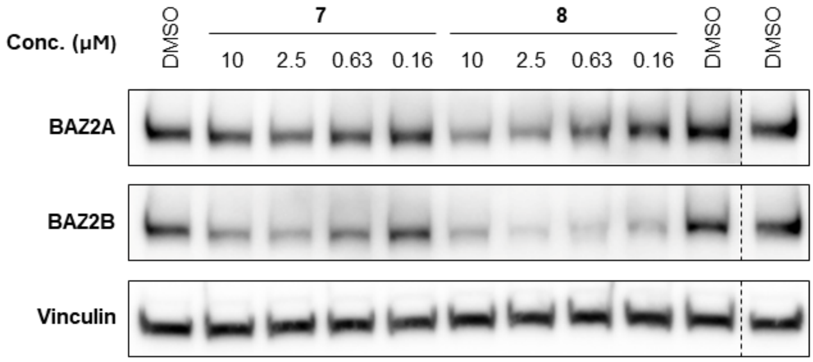

Membrane 11:

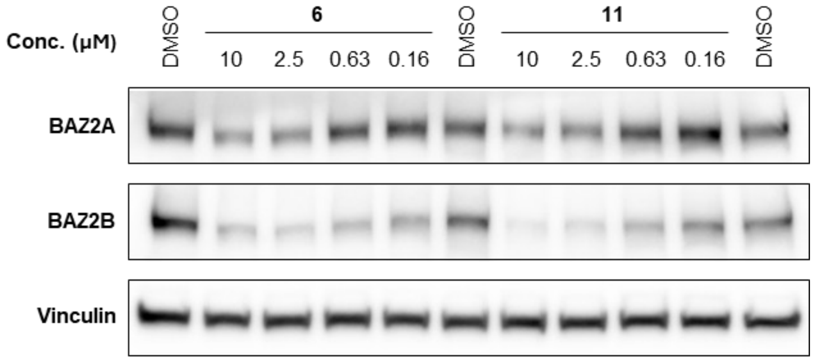

Membrane 12:

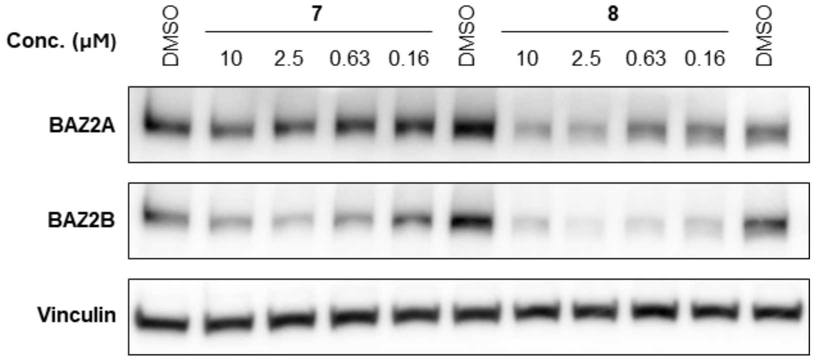

Fig. S2. [continued from previous page] Images of western blots used for quantification in Fig. 2: ‘pyrazole-VHL’ PROTAC dose response.

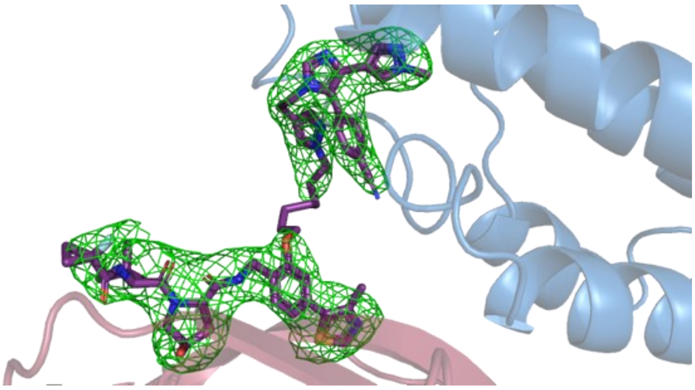

Fig. S3. Structure of the BAZ2B-BRD:**8**:VCB ternary complex at the ligand binding site, showing the mFo-DFc omit map at 3σ.

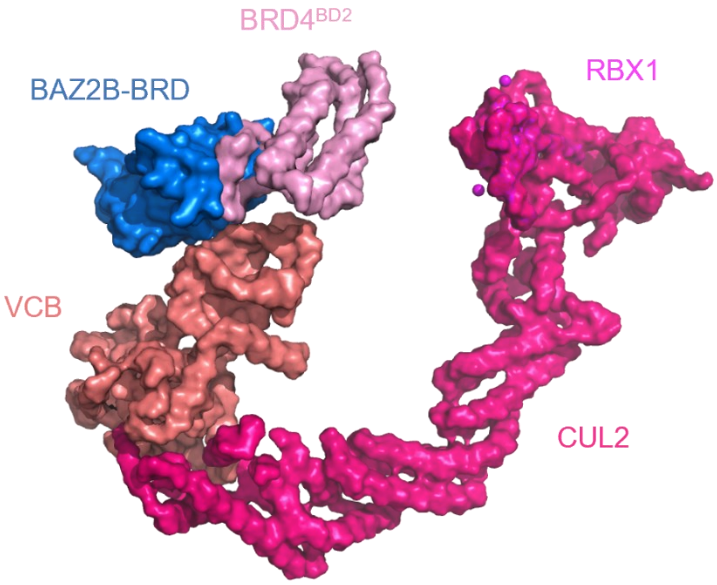

Fig. S4. BAZ2B-BRD:VCB complex induced by compound **8** aligned with a $BRD4^{BD2}$:$CRL2^{VHL}$ complex (reds and maroons, PDB ID: 8RWZ[1]) showing the difference in orientation of the bromodomains with respect to the RBX1 active site.

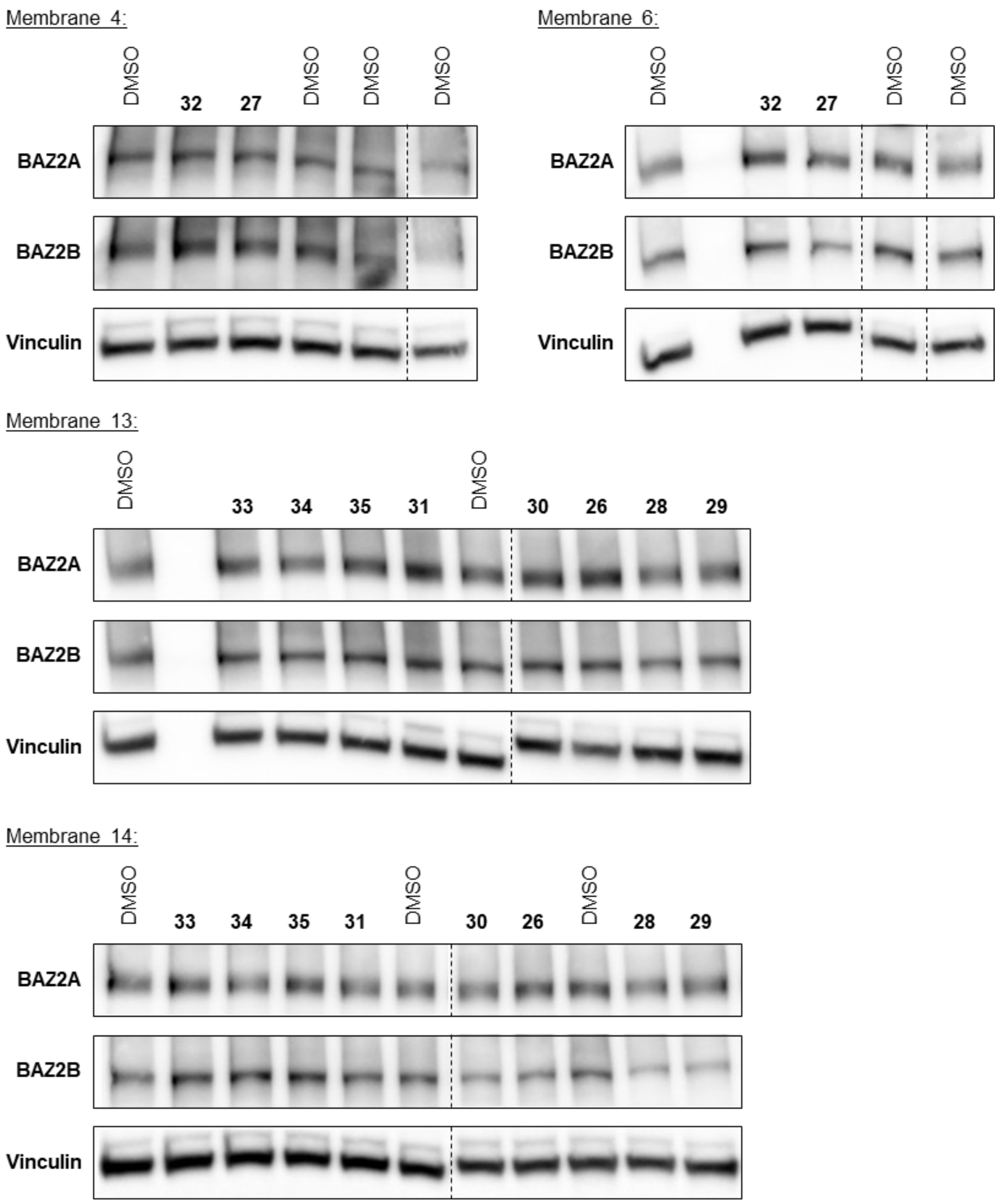

Fig. S5. Images of western blots used for quantification in Table 2: ‘pyrazole-CRBN’ PROTAC screening.

Membrane 6:

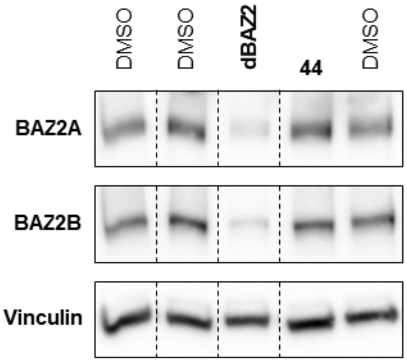

Membrane 9:

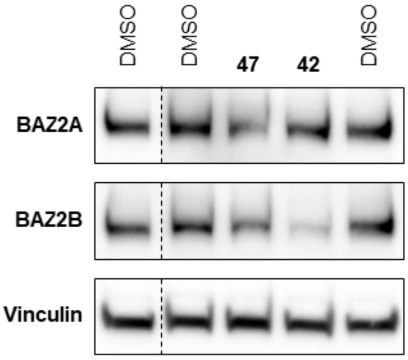

Membrane 15:

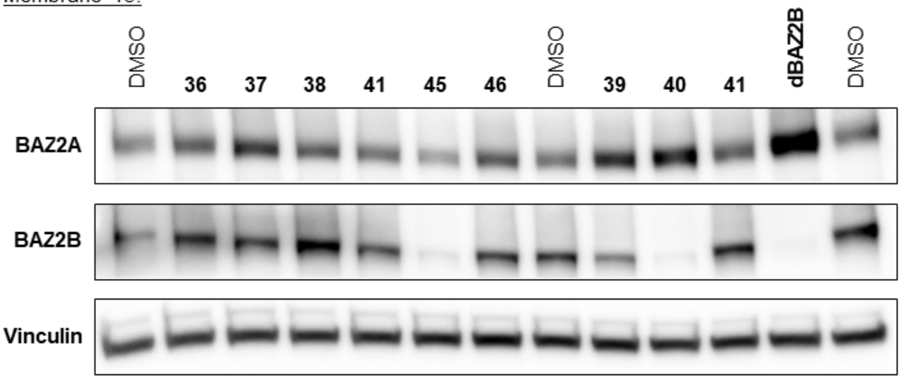

Membrane 16:

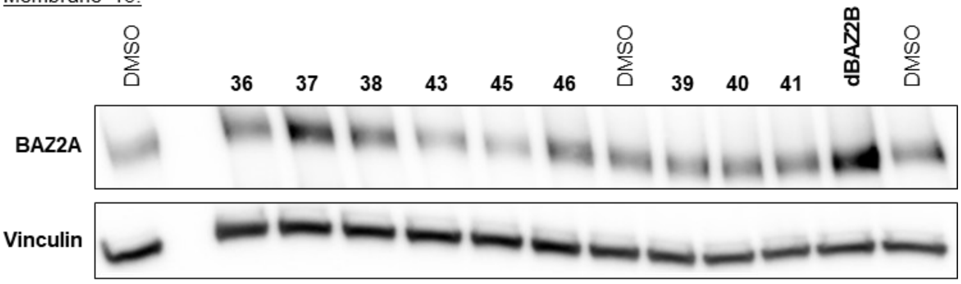

Membrane 17:

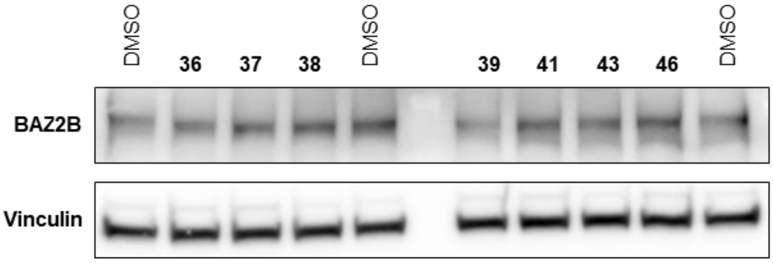

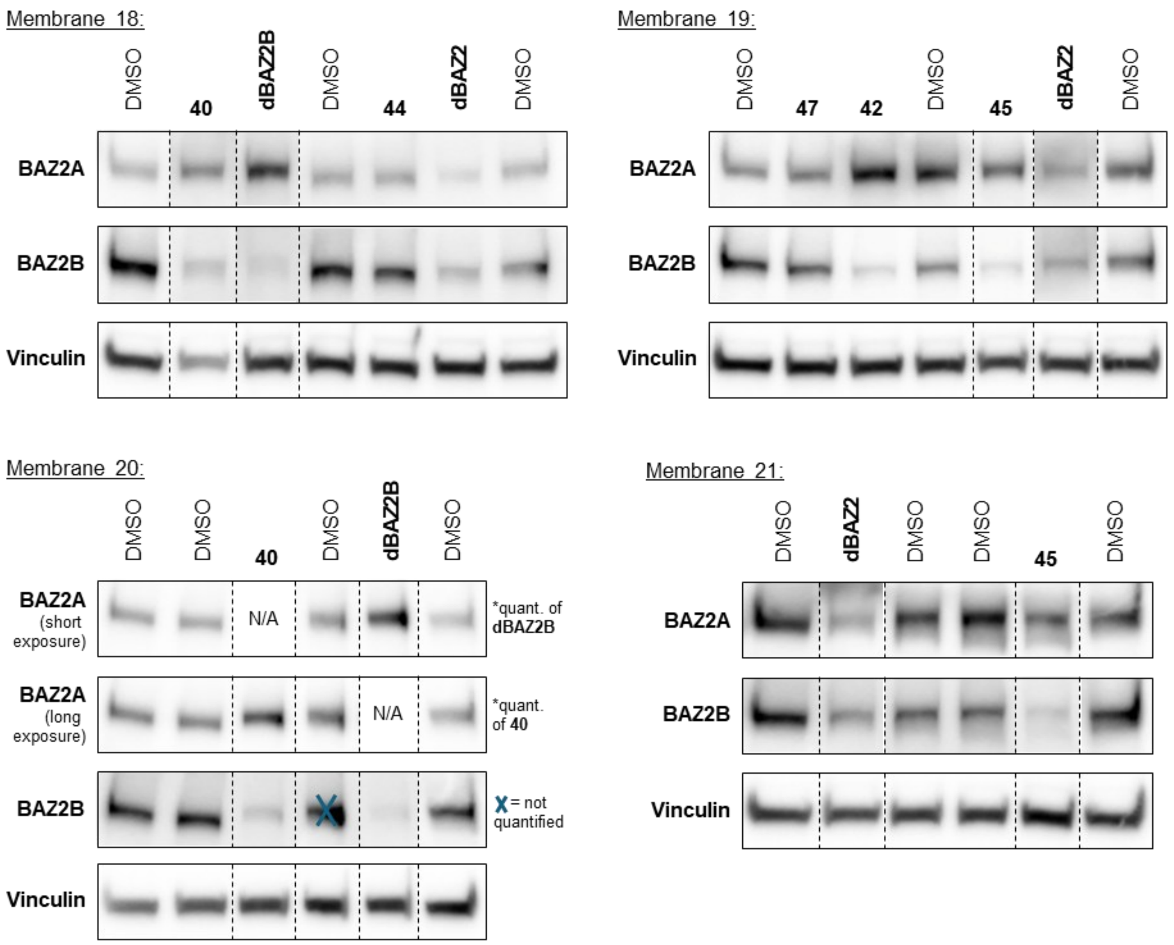

Fig. S6. [continued from previous page] Images of western blots used for quantification in Table 3: ‘imidazole’ PROTAC screening.

Membrane 6:

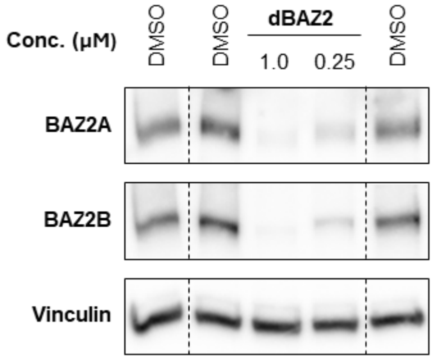

Also included replicates at 0.25 µM for compounds **40**, **45** and **dBAZ2B** from membranes 15 (BAZ2A and BAZ2B) and 16 (BAZ2A only, see Fig. S6)

Membrane 18:

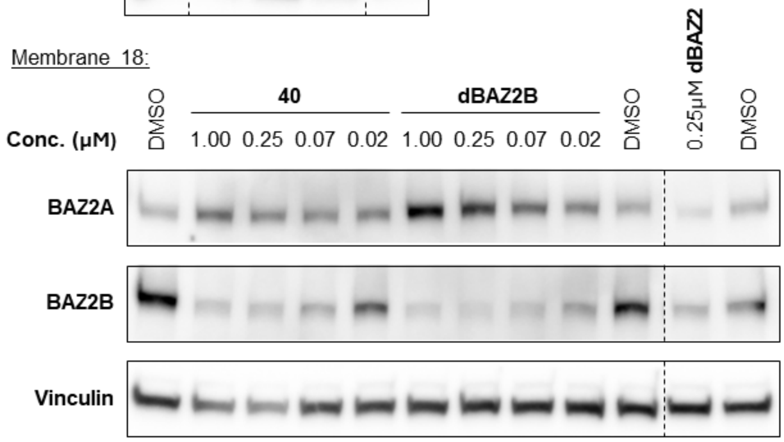

Membrane 19:

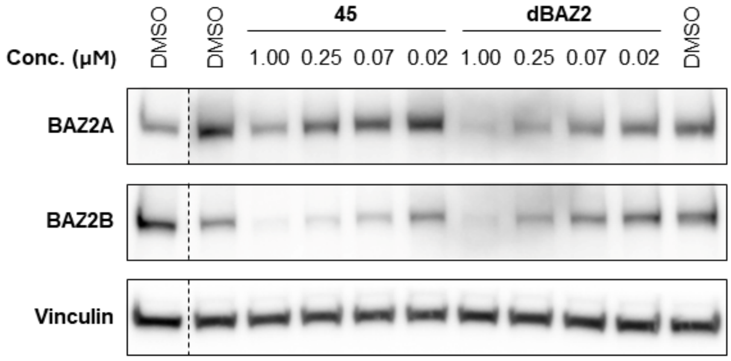

Membrane 21:

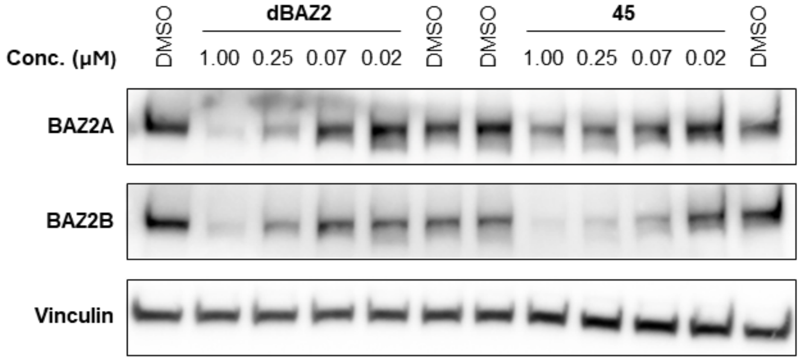

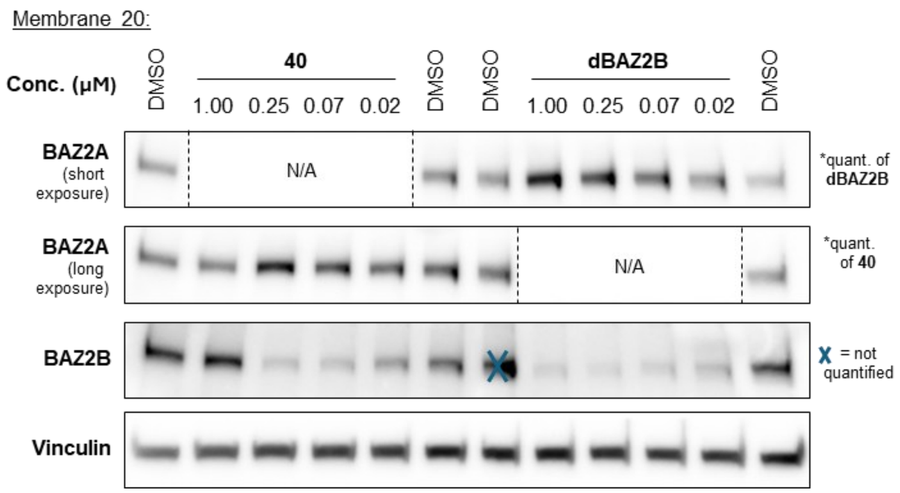

Fig. S7. [continued from previous page] Images of western blots used for quantification in Fig. 4: 'imidazole' PROTAC dose response.

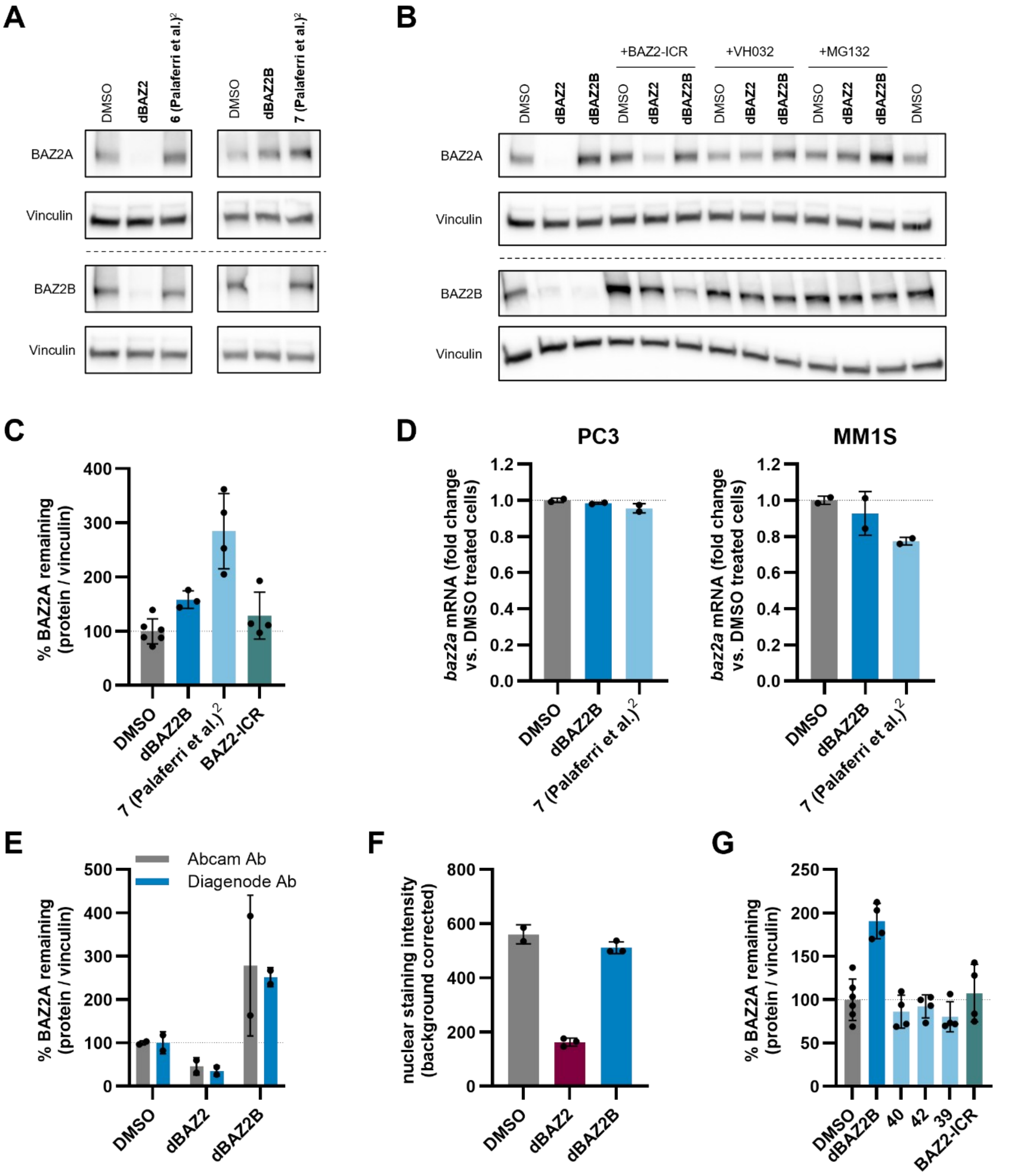

Fig. S8. Characterization of the degradation mechanism of **dBAZ2** and **dBAZ2B** and the observed elevation in BAZ2A with **dBAZ2B**. A) Western blot of BAZ2A and BAZ2B in PC3 cells after 4 h treatment with **dBAZ2** (1 µM), **dBAZ2B** (0.25 µM), or their negative controls **6** (1 µM) and **7** (0.25 µM), respectively; adapted from Palaferri *et al.*[2] B) Western blot of BAZ2A/B in PC3 cells after 1 h pretreatment with BAZ2-ICR (10 µM), VH032 (20 µM), or MG132 (10 µM) followed by 4 h treatment with **dBAZ2** (1 µM) or **dBAZ2B** (0.25 µM); reproduced from Palaferri *et al.*[2] C) Quantification of BAZ2A by western blot after 48 h treatment of PC3 cells with 0.25 µM compounds (images in Fig. S10).[2] For all western blots, BAZ2A protein levels were normalized first to Vinculin as a loading control and then the percentage remaining calculated relative to DMSO-treated cells run on the same

membrane. D) *baz2a* mRNA expression after 48 h (PC3) or 4 h (MM1S) treatment with 0.25 µM compounds. Gene expression was quantified by RT-qPCR, normalized to *hprt* and then to cells treated with DMSO in the same experimental run. E) Quantification of BAZ2A by western blot after 4 h treatment of MM1S cells with 1 µM **dBAZ2** or 0.25 µM **dBAZ2B** (images in Fig. S11). Abcam Ab = anti-BAZ2A/TIP5 [EPR25419-10] (Abcam ab290639) which was used for all other characterization, Diagenode Ab = anti-TIP5 (Diagenode C15310090). F) Mean nuclear fluorescence staining intensity after immunofluorescent staining of BAZ2A (one data point per well) in PC3 cells treated for 48 h with 1 µM **dBAZ2** or 0.25 µM **dBAZ2B**. DAPI staining was used to identify nuclear regions, background signal was calculated from cells stained with no primary BAZ2A antibody and subtracted from anti-BAZ2A stained cells (representative images in Fig. S12). G) Quantification of BAZ2A by western blot after 48 h treatment of PC3 cells with 0.25 µM compounds (images in Fig. S15), quantification as for C. Figures A and B adapted with permission from Palaferri *et al., ACS Med. Chem. Lett.* **2025**, *16* (2), 234–240. Copyright 2026 American Chemical Society.

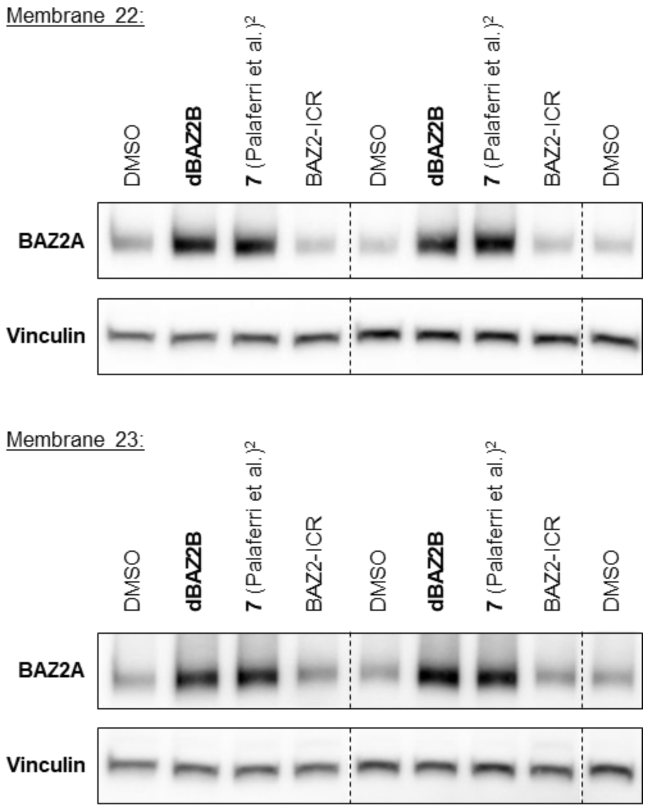

Fig. S9. Images of western blots used for quantification in Fig. 6A.

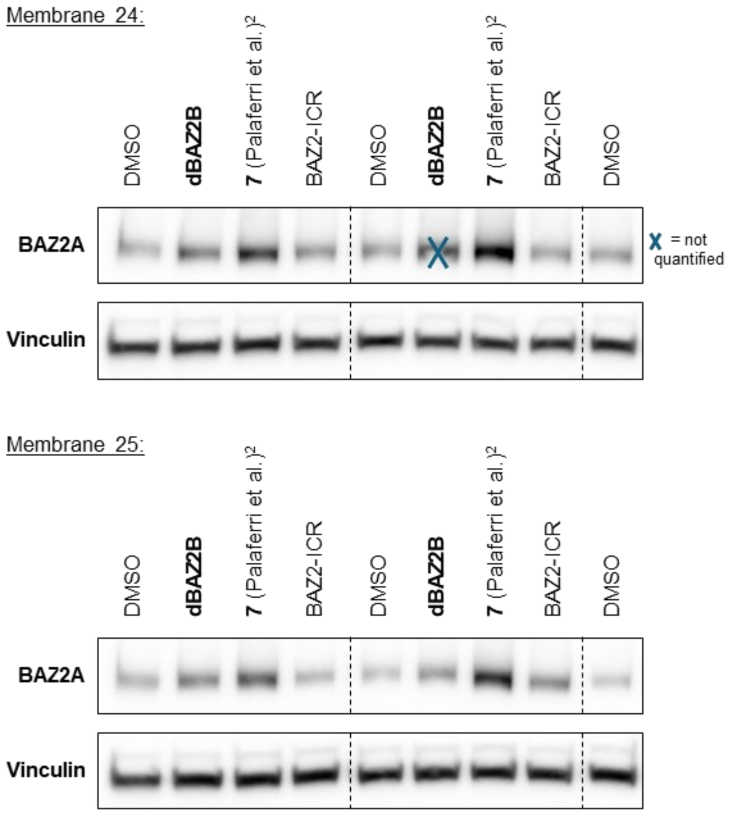

Fig. S10. Images of western blots used for quantification in Fig. S8C.

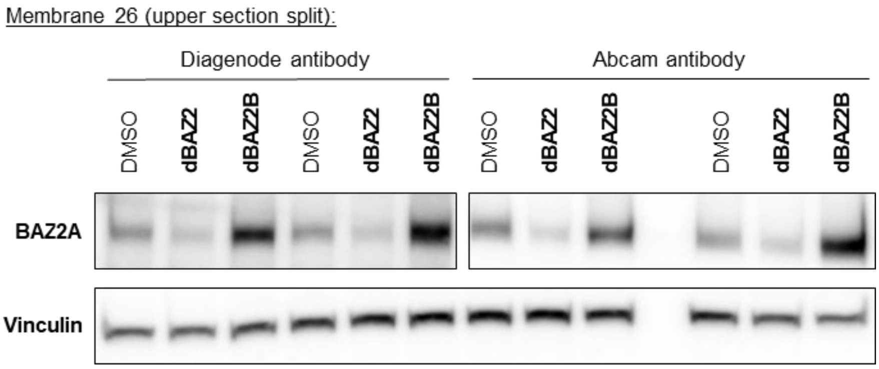

Fig. S11. Images of western blots used for quantification in Fig. S8E.

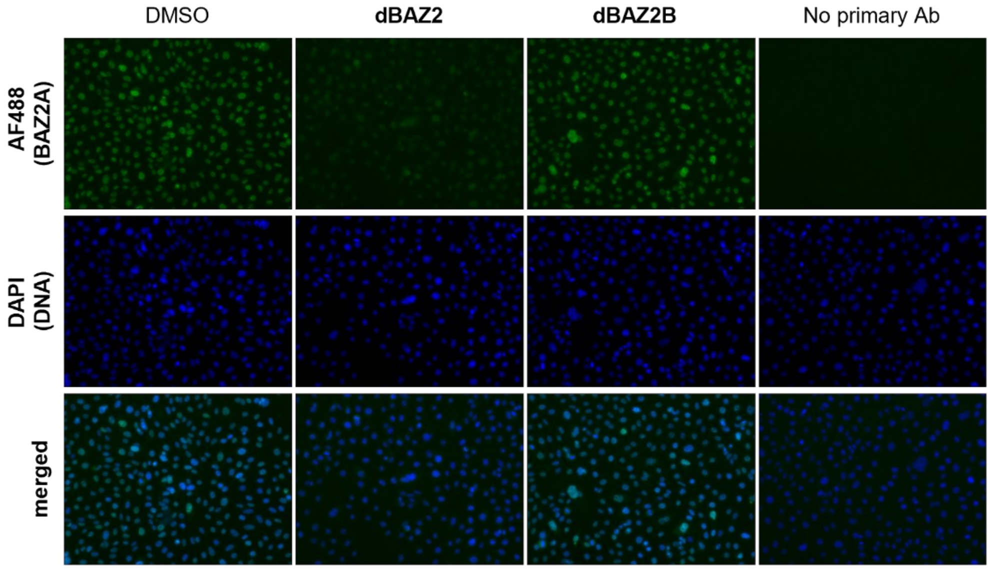

Fig. S12. Representative images of microscopy images used for quantification in Fig. S8F.

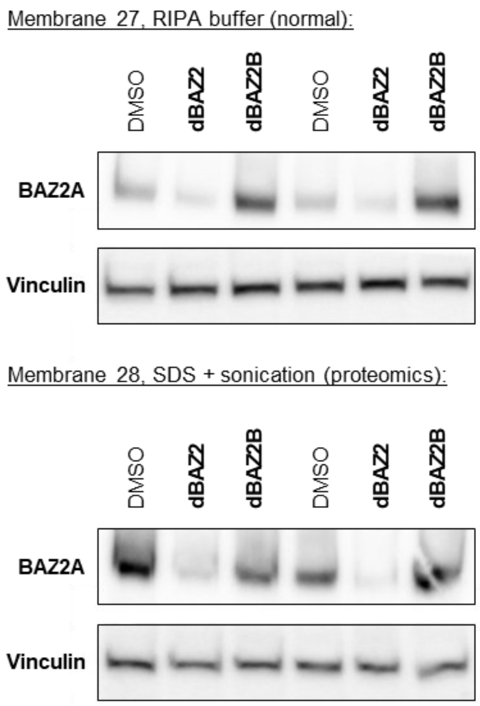

Fig. S13. Images of western blots used for quantification in Fig. 6D.

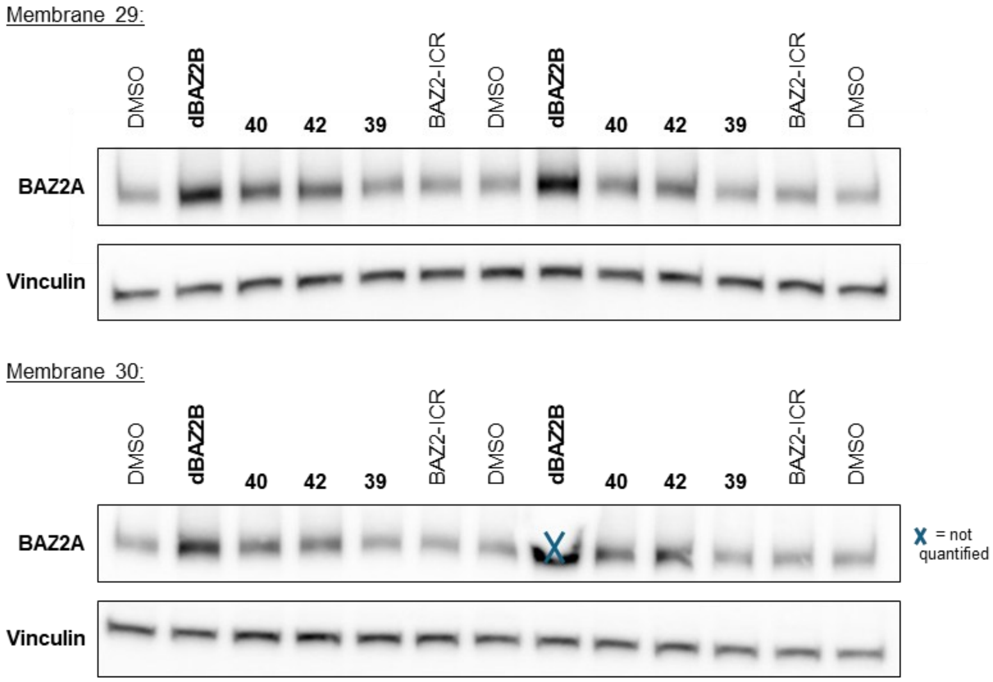

Fig. S14. Images of western blots used for quantification in Fig. 6E.

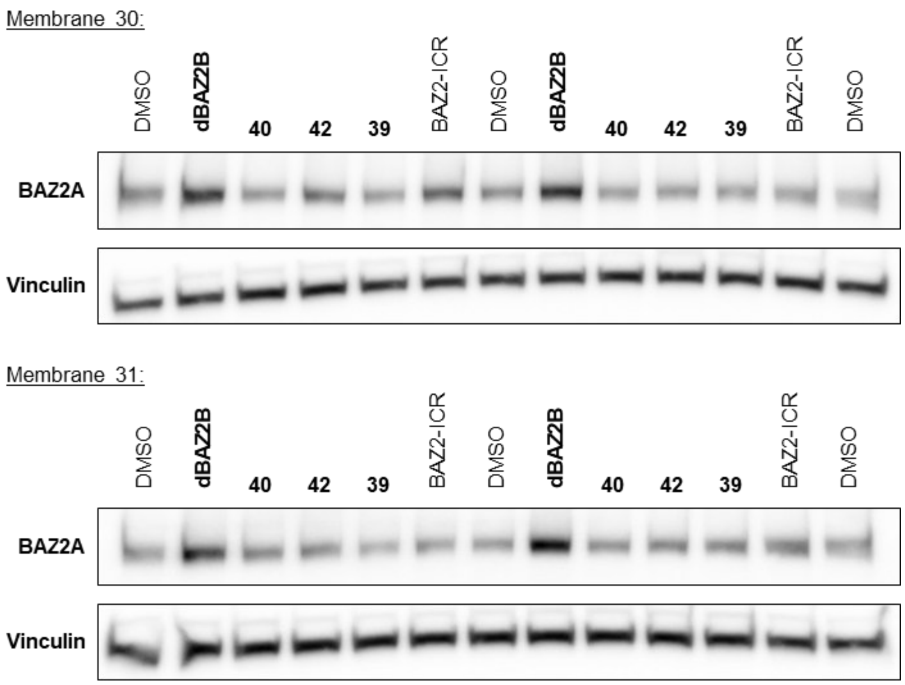

Fig. S15. Images of western blots used for quantification in Fig. S8G.

Table S1. Data collection, merging and refinement statistics for the BAZ2B-BRD:**8**:VCB ternary complex (Fig. 3).

	PDB: 29HO
**Data collection and merging**	
Wavelength	0.9537
Resolution range	74.77 - 2.45 (2.54 - 2.45)
Space group	P 2_1 2_1 2_1
Unit cell	
a, b, c	60.65, 73.355, 149.531
α, β, γ	90 90 90
Total reflections	327257 (33249)
Unique reflections	25239 (2470)
Multiplicity	13.0 (13.5)
Completeness (%)	99.92 (99.88)
Mean I/sigma(I)	15.87 (0.89)
Wilson B-factor	84.04
R-merge	0.06154 (2.206)
R-meas	0.0644 (2.292)
R-pim	0.01836 (0.6189)
CC1/2	0.966 (0.855)
CC*	0.991 (0.96)
**Refinement**	
R-work	0.2395
R-free	0.2843
RMS(bonds)	0.009
RMS(angles)	1.79
**Geometry**	
Ramachandran plot	
favored (%)	95.74
allowed (%)	4.04
outliers (%)	0.22
Rotamer outliers (%)	3.34
Clashscore	1.19
Average B-factor	115.49
macromolecules	115.86
ligands	107
solvent	99.63

Materials and Methods

Reagents

BAZ2-ICR was purchased from MedChem Express (HY-19336). All other compounds were synthesized in house, see following sections for details on synthetic procedures and chemical characterization.

Cell culture

PC3 and NALM6 cells were cultured in RPMI 1640 (Gibco 21875034), MM1S cells in RPMI 1640 ATCC modification (Gibco A1049101), and HeLa and HEK293T cells in DMEM (Gibco 41966029). All cell culture media were supplemented with 10 % FBS (Gibco A5256701), and all media except that for the NALM6 cells was additionally supplemented with penicillin/streptomycin (Gibco 15140). Cells were maintained in a humidified incubator with 5 % CO_2 at 37 °C. The identity of PC3 and HEK293T cells were authenticated by STR profiling (performed by Microsynth), MM1S cells were recently purchased from ATCC, and NALM6 cells were a gift from ChemoGenix, Université de Montréal.

Western blotting

For determination of BAZ2A and BAZ2B protein levels by western blotting, cells were plated on 6 well plates at the following densities: for 48 h compound treatment of PC3 cells, 5-7x10^4 cells/well were plated 72 h prior to treatment; for 16 h compound treatment of PC3 cells, 2.4x10^5 cells/well were plated 24 h prior to treatment; for MM1S cells either 6x10^5 cells/well or 8x10^5 cells/well were plated 72 h or 48 h prior to treatment respectively; for NALM6 cells 8x10^5 cells/well were plated 48 h prior to treatment; for HeLa cells 2x10^5 cells/well were plated 48 h prior to treatment; for HEK293T cells 6x10^5 cells/well were plated 24 h prior to treatment.

Cells were treated with compounds as described at a final concentration of 0.1 % DMSO, unless otherwise stated. Following treatment, cells were washed in ice cold PBS and lysed on ice with RIPA buffer (50 mM tris pH 7.5, 2 mM EDTA, 150 mM NaCl, 0.5 % sodium deoxycholate, 1 % Triton X-100) supplemented with cOmplete™ protease inhibitor cocktail (Roche) by scraping and resuspension by pipetting, before the lysates were clarified by centrifugation. Protein concentrations were then determined using the Pierce™ BCA Protein Assay Kit (Thermo Scientific, 23227).

Alternatively, a harsher lysis method was used where stated, mimicking preparation for proteomic analysis. Cells were washed on ice before being snap frozen in liquid nitrogen. Thawed cell pellets were lysed in 4 % SDS/Tris-HCl, resuspended by pipetting, treated for 2 x 1 min with High Intensity Focused Ultrasound (HIFU), and boiled at 95 °C for 10 min. Lysates were centrifuged at 20,000 x g for 10 min, resuspended again by pipetting, treated for an additional minute with HIFU and finally freeze-thawed a final time in liquid nitrogen to give a homogeneous non-viscous solution. Protein concentrations were determined using the Lunatic UV/Vis polychromatic spectrophotometer (Unchained Labs).

In all cases, equal amounts of protein (normally 20-30 µg but 50 µg for staining with anti-BAZ2A from Diagenode) were separated on AccuPAGE Bis-Tris 4-12 % gradient gels (Bio Bench AP-GPB-41215) and transferred to PVDF membranes. The membranes were

blocked with 5 % milk (Merck Millipore 115363) in PBS-T and proteins were stained with the following antibodies diluted in 5 % milk at 4 °C overnight: for BAZ2A, anti-BAZ2A/TIP5 [EPR25419-10] (Abcam ab290639) diluted 1:1000 was generally used, alternatively anti-TIP5 (Diagenode C15310090) diluted 1:1000 was used where stated; for BAZ2B, anti-BAZ2B (Atlas Antibodies HPA059292) diluted 1:500; for vinculin, used as a loading control, anti-vinculin [42H89L44] (Invitrogen 700062) diluted 1:1000. For detection, membranes were incubated for 1 hour at room temperature with HRP-conjugated secondary antibody (Southern Biotech, anti-rabbit 6415-05, 1:5000 dilution), and were imaged using SuperSignal™ West Pico PLUS Chemiluminescent Substrate (Thermo Scientific 34580) and the Vilber Fusion-FX7 imager. Bands were quantified using ImageJ software and protein levels were normalized firstly to Vinculin as a loading control and then to protein from DMSO-treated cells treated in parallel and run on the same membrane.

Protein expression and purification

Expression and purification of VCB: VCB (His-tagged VHL 54-213, EloB 1-104, EloC 17-112) was expressed and purified as previously described.[3] Briefly, *E. coli* BL21 (DE3) harbouring a plasmid for the expression of His-VCB and a plasmid for the bicistronic expression for EloB and EloC were grown in LB to an OD_{600} of 0.6 before induction using 0.25 mM IPTG and expression at 18 °C for 16 hours. Bacterial cells were lysed using a OneShot Cell Disruptor (Constant Systems) at 30 kpsi, 4 °C. Cell debris was removed by centrifugation. The cell lysate was loaded onto a HisTrap HP column (Cytiva) and the VCB complex eluted using a gradient of 50-250 mM Imidazole. Fractions containing VCB were pooled, combined with His-tagged TEV protease in a 1:100 ratio and dialyzed overnight against 20 mM HEPES pH 8.0, 150 mM NaCl. The resulting mixture was flowed through a HisTrap HP column to remove the protease and tagged proteins. The protein was subsequently polished using a HiTrapQ ion-exchange column (Cytiva), eluting with a gradient of 0-500 mM NaCl. Eluted VCB was concentrated and loaded onto a HiLoad Superdex S75 16/600pg size exclusion column (Cytiva) for final purification.

Expression and purification of His-VCB was performed as described for untagged VCB, omitting the addition of TEV protease during the dialysis step and the second pass through a HisTrap HP column.

Expression and purification of BAZ2B-BRD: BAZ2B-BRD (2054-2168) was purified as previously described.[4] Briefly, BAZ2B-BRD was expressed from a pNIC-Bsa4 plasmid as a His-tagged construct bearing a TEV cleavage site in *E. coli* BL21(DE3). Terrific broth medium was inoculated with an overnight culture grown in LB and incubated shaking at 37 °C until an OD_{600} of 1.8. Expression was induced by the addition of 0.1 mM IPTG. Cells were incubated for 16 h at 16 °C before harvesting.

Bacterial cells were lysed using a OneShot Cell Disruptor (Constant Systems) at 30 kpsi, 4 °C. Cell debris was removed by centrifugation and the cell lysate was loaded onto a HisTrap HP column (Cytiva). The protein of interest was eluted using a gradient of 50-500 mM Imidazole. Fractions containing BAZ2B-BRD were pooled, combined with His-tagged TEV protease in a 1:100 ratio and dialyzed overnight against 20 mM HEPES pH 8.0, 150 mM NaCl. The resulting mixture was loaded onto a HisTrap HP column, collecting the flow-through. The protein was subsequently concentrated and loaded onto a HiLoad

Superdex S75 16/600 pg size exclusion column (Cytiva) for final purification. Fractions containing monomeric BAZ2B-BRD were pooled, concentrated to 6.9 mM, snap frozen in liquid nitrogen and stored at -80 °C until further use.

Expression and purification of BAZ2A-BRD: BAZ2A-BRD (1796-1901) was purified as previously described.[4] Briefly, BAZ2A-BRD was expressed from a pCri11b plasmid as a His-SUMO-tagged construct in *E. coli* BL21(DE3). Terrific broth medium was inoculated with an overnight culture grown in LB and incubated shaking at 37 °C until an OD_{600} of 1.8. Expression was induced by the addition of 0.1 mM IPTG. Cells were incubated for 16 h at 16 °C before harvesting.

Bacterial cells were lysed using a OneShot Cell Disruptor (Constant Systems) at 30 kpsi, 4 °C. Cell debris was removed by centrifugation and the cell lysate was loaded onto a HisTrap HP column (Cytiva). The protein of interest was eluted using a gradient of 50-500 mM Imidazole. Fractions containing BAZ2A-BRD were pooled, combined with His-tagged Ulp1 in a 1:1000 ratio and dialyzed overnight against 20 mM HEPES pH 8.0, 150 mM NaCl. The resulting mixture was loaded onto a HisTrap HP column, collecting the flow-through. The protein was subsequently concentrated and loaded onto a HiLoad Superdex S75 16/600 pg size exclusion column (Cytiva) for final purification. Fractions containing monomeric BAZ2A-BRD were pooled, concentrated to 3.7 mM, snap frozen in liquid nitrogen and stored at -80 °C until further use.

Isolation of the BAZ2B-BRD:8:VCB ternary complex

BAZ2B-BRD was incubated with VCB and PROTAC **8** in a molar ratio of 2:1:1.1 and incubated on ice for 1 h. The ternary complex was subsequently isolated by size exclusion using a HiLoad Superdex S75 16/600 pg column, equilibrated with 20 mM HEPES pH 8.0, 200 mM NaCl, 1 mM TCEP, 5 % glycerol. Eluting ternary complex was pooled, concentrated to 10 mg/ml and snap-frozen in liquid nitrogen.

Crystallography

Crystals of the BAZ2B-BRD:**8**:VCB ternary complex were grown in 16-20 % PEG8000, 150-200 mM $Ca(OAc)_2$, 100 mM sodium cacodylate pH 6.5 at 293 K. Crystals usually appeared after a single day and reached their final size after 3 days. Crystals were snap-frozen in liquid nitrogen without cryoprotectant.

Diffraction data were collected at Diamond Light Source beamline i04 with a wavelength of 0.9537 Å, oscillation of 0.1° and exposure of 0.0101 s. A full rotation was collected. Scaling and merging was performed using the Diamond Light Source pipeline, using xia2/DIALS.[5–8] AIMLESS[9] was used for data reduction. MR-Phaser[10] was used for molecular replacement using PDB ID 4XUB[11] and 8P0F[12] as search models for BAZ2B and VCB, respectively. Schrodinger Maestro (Schrödinger, Inc.) was used to relax the ligand structure and phenix.eLBOW[13] was used to generate geometric restraints for the ligand based on the resulting model. The model was iteratively refined using REFMAC5[14] and Coot,[15] until refinement statistics converged. In order to determine whether further resolution shells should be included we performed paired refinement using PAIREF.[16]

TR-FRET

TR-FRET was measured with a 3-fold dilution series of compounds at a concentration range of 1.1 nM - 2.5 μM and His-VCB at a concentration of 4 nM. BAZ2-BRDs were supplemented at a concentration of 2.5 μM unless otherwise specified. 0.5 nM LANCE anti-6His EU-antibody (Revvity) was used as a FRET donor and 200 nM Celt-050 (Celtarys) used as FRET acceptor. Assay components were combined in a buffer consisting of 20 mM HEPES pH 8.0, 150 mM NaCl, 0.5 mg/ml BSA, 0.5 mM TCEP, 0.005 % Tween-20 and incubated for 16 h at 4 °C before equilibration to RT for subsequent measurement of the TR-FRET rate. DMSO without compound and assay mixture lacking His-VCB were used as positive and negative controls, respectively. FRET signals were measured using a PHERAstar *FSX* plate reader (BMG Labtech) equipped with an HTRF 337-665-620 optical module after 60 μs filtering time, with 400 μs integration time. Experiments were performed as a series of 3 independent replicates with 2 technical replicates each. Measured signal was normalized between the controls, and non-linear curve fitting was performed using GraphPad Prism (GraphPad Software) in order to determine the IC_{50} and, subsequently, K_i.

RT-qPCR

For analysis of the expression of BAZ2A-regulated genes, PC3 cells were plated on 12 well plates at a density of $2x10^4$ cells/well 24 h prior to compound treatment. For analysis of *baz2a* expression, $5x10^4$ PC3 cells or $8x10^5$ MM1S cells were plated per well on 6 well plates 72 h prior to treatment. Cells were treated as described at a final concentration of 0.1 % DMSO. RNA was extracted using the NucleoSpin® RNA kit (Macherey-Nagel 740955) and 250 or 500 ng used for cDNA synthesis with the High-Capacity cDNA Reverse Transcription kit (Applied Biosystems 4368814). RT-qPCR reactions were performed using *Power*SYBR™ green PCR Master Mix (Applied Biosystems 4367659) and a LightCycler® 480 (Roche). cDNA was denatured for 10 min at 95 °C followed by 40 cycles of 15 s at 95 °C and 1 min at 60 °C. Cp values were determined as the maximum of the second derivative of the amplification curve and target gene expression was compared to that of *hprt* from the same sample using the $2^{-\Delta Cp}$ method. Gene expression was then normalized to the average of the DMSO-treated samples from the same experimental run. Significance was calculated for all compounds vs. **dBAZ2** for each gene by two-way ANOVA, correcting p-values using Dunnett's multiple comparisons test.

The primer sequences used were:

Gene Name	Gene ID	Forward Primer (5'-3')	Reverse Primer (5'-3')
aox1[17]	NM_001159.4	CCGACAAGCCCAGCGACAGG	GTGGCTGGACCAACGCCTCC
baz2a[18]	NM_013449.4	AAGATGTGTGGCTACAATGG	TCTGCACCCATCAGCTCCG
fbn1[17]	NM_000138.5	GCCTCAACACCCGTGGGAGC	GCACTCGTCCTGGTTGGGGC
flh2	NM_001450.4	GGAGTTGGGGAGACTGGTTGC	GCAATGGTGGCAGTCAAAGC
homer2[17]	NM_004839.4	TTGACGCAGAGCGCAGCCAA	TGCAGTGCTGTGGTCAGCCG
hprt	NM_000194.3	CCCTGGCGTCGTGATTAGTG	TCGAGCAAGACGTTCAGTCC
klf6[17]	NM_001300.6	ACCTCCGACCCCATTGGCGA	AAGTCCCGCTGCGCACCTTC
lamb3	NM_000228.3	CCCATGAATGCCAAAGGTGC	CTGACACCGCTCACAGTTCT
znf185[17]	NM_001178106.1	AGCAGCCCCAGCGGATCTGA	GTCCTGCCAGGCCTCTCCGA

Proteomics

Sample digestion and clean up: Treated cells were washed in PBS and cell pellets were then lysed in 4 % SDS/Tris-HCl, treated with High Intensity Focused Ultrasound (HIFU) for 1 min at an ultrasonic amplitude of 80 % and boiled at 95 °C for 10 min. Ten units of Benzonase were added after diluting SDS to 1 % followed by centrifugation at 20000 x g for 10 min. The protein concentration was estimated using the Lunatic UV/Vis polychromatic spectrophotometer (Unchained Labs). For each sample, 10 µg of proteins were reduced with 2 mM TCEP (tris(2-carboxyethyl)phosphine) and alkylated with 15 mM chloroacetamide at 30 °C for 30 min. Samples were processed using the single-pot solid-phase enhanced sample preparation (SP3). The SP3 protein purification, digest and peptide clean-up were performed using a KingFisher Flex System (Thermo Fisher Scientific) and Carboxylate-Modified Magnetic Particles (GE Life Sciences; GE65152105050250, GE45152105050250).[19,20] Beads were conditioned following the manufacturer's instructions, consisting of 3 washes with water at a concentration of 1 µg/µl. Samples were diluted with 100 % ethanol to a final concentration of 50 % ethanol. The beads, wash solutions and samples were loaded into 96 deep well- or micro-plates and transferred to the KingFisher. The following steps were carried out on the robot: collection of beads from the last wash, protein binding to beads, washing of beads in wash solutions 1-3 (80 % ethanol), protein digestion (overnight at 37 °C with a trypsin:protein ratio of 1:50 in 50 mM Triethylammonium bicarbonate (TEAB)) and peptide elution from the magnetic beads using MilliQ water. The digest solution and water elution were combined and dried to completeness.

TMT labelling and peptide fractionation: 50 µg TMTpro 18-plex reagent (Thermo Fisher Scientific) was dissolved in 5 µl of anhydrous acetonitrile (Sigma-Aldrich) and added to 10 µg peptides in 15 µl of 50 mM TEAB, pH 8.5. The solution was gently mixed and incubated for 60 min at room temperature. The reaction was quenched by adding 1.2 µl of 5 % hydroxylamine (Thermo Fisher Scientific). The combined TMT sample was created by mixing equal amounts of each TMT channel together. Labeled peptides were offline pre-fractionated using high pH reverse phase chromatography. Peptides were separated on an XBridge Peptide BEH C18 column (130 Å, 3.5 µm, 4.6 mm X 250 mm, Waters) using a 72 min linear gradient from 5-40 % acetonitrile / 9 mM NH_4HCO_2. Every minute a new fraction was collected and concatenated into 12 final fractions.

LC-MS/MS analysis: Mass spectrometry analysis was performed on an Orbitrap Exploris 480 mass spectrometer (Thermo Fisher Scientific) equipped with a Nanospray Flex Ion Source (Thermo Fisher Scientific) and coupled to an M-Class UPLC (Waters). Solvent composition at the two channels was 0.1 % formic acid for channel A and 0.1 % formic acid, 99.9% acetonitrile for channel B. Column temperature was 50 °C. Peptides were loaded on a commercial nanoEase MZ Symmetry C18 Trap Column (100 Å, 5 µm, 180 µm x 20 mm, Waters) connected to a nanoEase MZ C18 HSS T3 Column (100 Å, 1.8 µm, 75 µm x 250 mm, Waters). Peptides were eluted at a flow rate of 300 nL/min. After a 3 min initial hold at 5 % B, a gradient from 5 to 22 % B in 80 min and 22 to 32 % B in additional 10 min was applied. The column was cleaned after the run by increasing to 95 % B and holding 95 % B for 10 min prior to re-establishing loading condition for another 10 min. The mass spectrometer was operated in data-dependent mode (DDA) with a maximum cycle time of 3 s, with spray voltage set to 2.6 kV, funnel RF level at 40 % and heated

capillary temperature of 275 °C. Full-scan MS spectra (350−1’500 m/z) were acquired at a resolution of 120’000 at 200 m/z after accumulation to a target value of 3’000’000 or for a maximum injection time of 45 ms. Precursors with an intensity above 5’000 were selected for MS/MS. Ions were isolated using a quadrupole mass filter with 0.7 m/z isolation window and fragmented by higher-energy collisional dissociation (HCD) using a normalized collision energy of 32 %. HCD spectra were acquired at a resolution of 30’000 with turboTMT on and maximum injection time was set to Auto. The normalized automatic gain control (AGC) was set to 100 %. Charge state screening was enabled such that singly, unassigned and charge states higher than six were rejected. Precursor masses previously selected for MS/MS measurement were excluded from further selection for 20 s, and the exclusion window was set at 10 ppm. The samples were acquired using internal lock mass calibration on m/z 371.1012 and 445.1200.
The mass spectrometry proteomics data were handled using the local laboratory information management system (LIMS).[21]

Data analysis: The acquired shotgun MS data were processed for identification and quantification using Fragpipe 19.0 (Philosopher 4.8.1).[22] Spectra were searched against a Uniprot *Homo sapiens* reference proteome (reviewed canonical version from 2023-03-30, concatenated to its reversed decoyed fasta database and common protein contaminants) using MSFragger 3.6 and Percolator. Proteins were identified using at least one peptide. TMT modification on peptide N-termini and Lysine side chains as well as carbamidomethylation of cysteine were set as fixed modifications, while methionine oxidation was set as variable. Enzyme specificity was set to trypsin/P allowing a minimal peptide length of 7 amino acids and a maximum of two missed cleavages. Reporter ion intensities were extracted with 20 ppm integration tolerance. For peptide and protein quantification the co-isolation filter was set to 50 %. The R package prolfqua[23] was used to analyze the differential expression and to determine group differences, confidence intervals, and false discovery rates for all quantifiable proteins. Starting with the psm.tsv file generated by FragPipe, which does report the peptide spectrum match abundances (psm) for each TMT label, we filtered the psm's by purity_threshold = 0.5, PeptideProphetProb = 0.9. Next, we determined protein abundances by aggregating the psm abundances to peptidoform abundances and then employed the Tukeys-Median Polish to estimate protein abundances. Furthermore, before fitting the linear models, we transformed the protein abundances using the variance stabilizing normalization.[24] To analyze the differential expression, data were fitted to a linear model that explains the observed protein abundances using the R function *lm* for each protein, with samples grouped by compound treatment. Differences between PROTAC treated and DMSO-treated samples were computed, and a null hypothesis significance test was performed. Variance shrinkage was performed[25] to increase the power of the analysis, and finally the false discovery rate (FDR) was computed using the Benjamini-Hochberg procedure.[26]

Immunofluorescence

For immunofluorescence studies, 5x10^3 PC3 cells/well were plated on a µClear 96 well plate (Greiner 655090) 48 h prior to cell treatment. Cells were treated with the stated compounds at a final concentration of 0.1 % DMSO. Prior to immunostaining, cells were washed with PBS (also between subsequent steps), fixed with 4 % PFA for 30 min and permeabilized with 0.25 % Triton X-100. Cells were blocked with 2 % BSA for 1 h, stained

with anti-BAZ2A antibody (Abcam ab290639, diluted 1:500 in blocking buffer, 1 h at room temperature), then stained with DAPI (final concentration of 0.8 μg/ml) and AF488 conjugated secondary antibody (donkey anti-rabbit, BioLegend 406416 diluted 1:250) in blocking buffer for 1 h at room temperature. Images (76 per well) were obtained using an Evos M7000 at 20x magnification using GFP and DAPI light cubes.

ImageJ was used for quantification. Briefly, nuclei were identified using the DAPI channel image by subtracting the background, thresholding using the Huang algorithm, running the watershed algorithm, and selecting particles with a size of 200 – infinity pixels. The mean signal intensity of the GFP channel (AF488) image for each nuclear area was calculated. Nuclei with an area greater than $3x10^6$ or a roundness of less than 0.35 were excluded from further analysis (25 nuclei removed, leaving 199384 across all wells and conditions). A z-score for AF488 staining intensity of each nucleus was calculated on a per well basis and nuclei with a z-score greater than 10 were also excluded from the analysis (a further 65 nuclei removed). The mean AF488 staining of all nuclei in a well was calculated and plotted as a single data point. Background signal was calculated from a well treated in an analogous fashion but without primary antibody and subtracted from the mean signal of stained wells.

Flow cytometry

For flow cytometry experiments, $6x10^5$ MM1S cells/well were plated on 6 well plates 72 h prior to treatment with the stated compounds at a final concentration of 0.1 % DMSO. Cells were collected on ice and washed with cold PBS. To identify dead cells, cells were stained with Zombie Violet™ (423113 BioLegend) diluted 1:500 in PBS for 15 min at room temperature. Cells were washed in cold PBS + 2 % FBS (used for all further washes), then fixed with 4 % PFA for 30 min at room temperature, before washing again. Cells were blocked with 2 % BSA, washed and stained with anti-BAZ2A/TIP5 [EPR25419-10] (Abcam ab290639) diluted 1:500 in blocking buffer for 1 h at room temperature. After washing, cells were stained with AF488 donkey anti-rabbit secondary antibody (BioLegend 406416) diluted 1:250 in blocking buffer for 1 h at room temperature. Finally, cells were washed and resuspended in FACS buffer (PBS + 2 % FBS + 0.5 mM EDTA).

Cells were analyzed using a FACSymphony and FlowJo™ v10 software (BD Biosciences). Gating was performed based on FSC-A vs. SSC-A (to identify cells), FSC-A vs. FSC-H (to select single cells) and Zombie Violet staining intensity (to exclude dead cells [excitation 405 nm, emission 450/50 nm]). AF488 staining intensity was measured using an excitation of 488 nm and emission of 530/30 nm. The geometric mean AF488 staining intensity of live single cells was calculated for each well and plotted as a single data point. Background signal was calculated from cells stained in an analogous fashion but without primary antibody and subtracted from the mean signal of stained cells.

Chemogenomic screening

A genome-wide pooled CRISPR/Cas9 knockout screen was performed in NALM6 cells by the ChemoGenix platform at the University of Montreal (https://chemogenix.iric.ca/) as described previously.[27] Briefly, a NALM6 clone stably expressing doxycycline-inducible Cas9 was infected with lentiviruses encoding the sgRNA EKO library (>278K sgRNAs, 10 sgRNAs per gene) at a low multiplicity of infection followed by blasticidin selection. After 1 day of recovery from thawing, gene knockout was induced by expansion for 7 days in

medium with 2 µg/ml doxycycline. Cells were separated into T75 bottles, each containing 70 ml cells at $4x10^5$ cells/ml ($28x10^6$ total cells, 100 cells per sgRNA) and treated with either DMSO or 1 µM **dBAZ2**. Cells were cultured for 8 days during which growth was monitored every two days by counting, and cells diluted back to $4x10^5$ cells/ml when their concentration exceeded $8x10^5$ cells/ml using DMSO/compound containing medium. Following treatment, cells were harvested, genomic DNA extracted and sgRNA sequences amplified by PCR. Next generation sequencing of sgRNA sequences was performed using an Illumina NextSeq 2000, and sgRNA frequencies used to compute statistically robust CRANKs scores for each gene.

Data analysis

Data were analyzed and graphs prepared using R v. 4.5.1 and GraphPad Prism v. 10.5.0. Bars and error bars show mean ± standard deviation, individual data points are shown as dots to indicate the n number. Protein structures were analyzed and figures generated using PyMOL v. 3.0.0 Open-Source.

Characterization of compounds

NMR spectra were recorded on AV 300, AV2 400 or AV2 500 MHz Bruker spectrometers. The spectra are calibrated to the residual ^1H and ^{13}C signals of the solvents. Chemical shifts are reported in ppm with the resonance resulting from incomplete deuteration of the solvent as the internal standard. Multiplicities are abbreviated as follows: singlet (s), doublet (d), triplet (t), quartet (q), doublet-doublet (dd), quintet (quint), multiplet (m), and broad (b). High resolution electrospray ionization mass spectrometry, HRMS (ESI), was performed on a Dionex Ultimate 3000 UHPLC system (Thermo Fisher Scientific, Germering, Germany) connected to a QExactive MS with a heated ESI source (Thermo Fisher Scientific, Bremen, Germany); mass calibration to <2 ppm accuracy with Pierce® ESI calibration solutions. (Thermo Fisher Scientific, Rockford, USA). LC-MS analysis of small molecules (≤ ca. 2000 Da) was carried out by a less than 5 min HPLC gradient elution based on H2O + 0.02 % TFA (mobile phase A1) and ACN + 0.02 % TFA (mobile phase B1).

Synthetic and purification methods

Unless otherwise stated, reactions were carried out under a nitrogen atmosphere using standard Schlenk-techniques. All reagents were used as received unless otherwise noted. Solvents were purchased in the best quality available, and when indicated degassed by purging thoroughly with nitrogen or dried over activated molecular sieves of appropriate size. Reactions were monitored by thin layer chromatography (TLC) using Merck TLC silica gel 60 F254 and using UV light (254 nm) as visualizing agent and acidic ceric ammonium molybdate/ phosphomolybdic acid or potassium permanganate solutions and heat as developing agents. Column chromatography was performed over silica gel (230-400 mesh) under nitrogen pressure or using a Smart Flash W-Prep 2XY flash chromatography system using Yamazen Universal Premium Columns. All biologically tested compounds are >95 % pure by LC-MS. Reversed-phase HPLC purification was performed on a Shimadzu Nexera system, equipped with an Agilent Zorbax 300SB-C18 semipreparative column (9.4 x 250 mm 5-micron) using 20 to 90 % acetonitrile in water buffered with 0.1 % trifluoroacetic acid as eluent (3.5 mL/min).

General synthetic procedures

Synthesis of ligand-linker intermediates

General procedure 1: synthesis of BAZ2 'pyrazole' warhead-tosyl intermediates

BAZ2 'pyrazole' warhead (1.0 equiv) and NaH (1.3 equiv) were suspended in dry DMF (0.1 M) and the resulting suspension stirred at 0 °C for 1 hour. Then, a solution of ditosyl linker (3.0 equiv) was added and the reaction mixture stirred for 3 hours and allowed to warm up to 20 °C. After this time, the reaction was quenched with water, extracted with DCM and the organic layer washed with brine, dried over anhydrous $MgSO_4$, filtered and the solvent removed under reduced pressure. The crude product was purified by flash chromatography (MeOH:DCM) to afford the corresponding **BAZ2 'pyrazole' warhead-tosyl intermediate**.

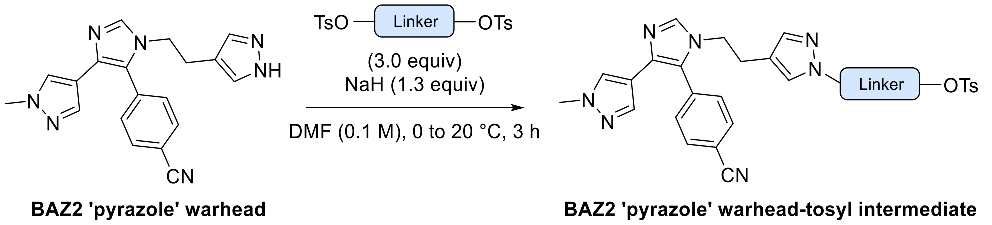

General procedure 2: synthesis of BAZ2 'imidazole' warhead-tosyl intermediates

BAZ2 'imidazole' warhead [cpd 1 from Palaferri *et al.*][2] (1.0 equiv) and NaH (1.3 equiv) were suspended in dry DMF (0.1 M) and the resulting suspension stirred at 0 °C for 1 hour. Then, a solution of ditosyl linker (3.0 equiv) was added and the reaction mixture stirred for 3 hours and allowed to warm up to 20 °C. After this time, the reaction was quenched with water, extracted with DCM and the organic layer washed with brine, dried over anhydrous $MgSO_4$, filtered and the solvent removed under reduced pressure. The crude product was purified by flash chromatography (MeOH:DCM) to afford the corresponding **BAZ2 'imidazole' warhead-tosyl intermediate**.

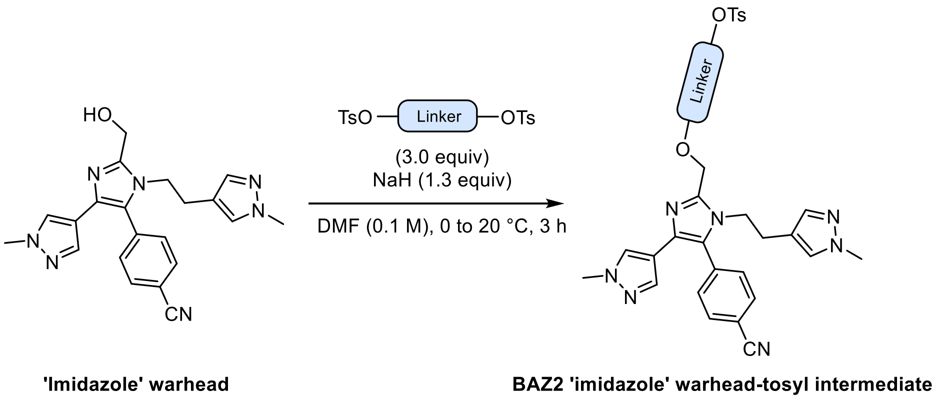

General procedure 3: synthesis of VHL phenol-tosyl intermediates

VHL phenol[28] (1.0 equiv), distosyl linker (3.0 equiv) and K_2CO_3 (2.0 equiv) were dissolved in dry DMF (0.1 M) and the resulting reaction mixture stirred at 80 °C for 2 hour. After this time, the reaction was quenched with water, extracted with DCM(x4) and the organic layer washed with brine, dried over anhydrous $MgSO_4$, filtered and the solvent removed under reduced pressure. The crude product was purified by flash chromatography (MeOH:DCM) to afford the corresponding **VHL phenol-tosyl intermediate**.

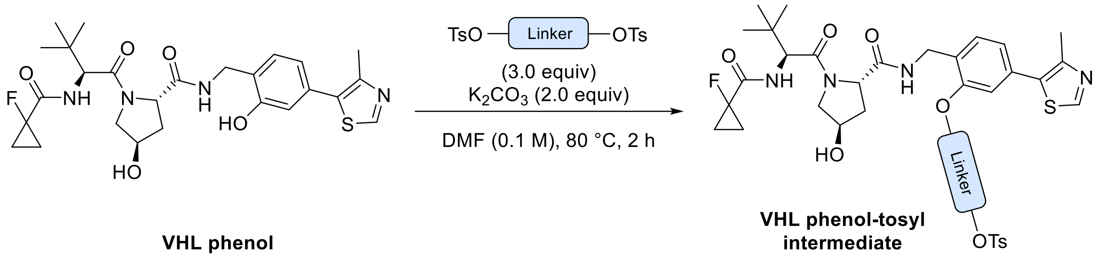

General procedure 4: synthesis of VHL thiol-tosyl intermediates

VHL thiol[29] (1.0 equiv) and distosyl linker (3.0 equiv) were dissolved in dry THF (0.1 M) and DBU (1.5 equiv) was added. Then, the reaction mixture was stirred at 22°C for 15 hour. After this time, the reaction was quenched with water, extracted with DCM and the organic layer washed with brine, dried over anhydrous $MgSO_4$, filtered and the solvent removed under reduced pressure. The crude product was purified by flash chromatography (MeOH:DCM) to afford the corresponding **VHL thiol-tosyl intermediate**.

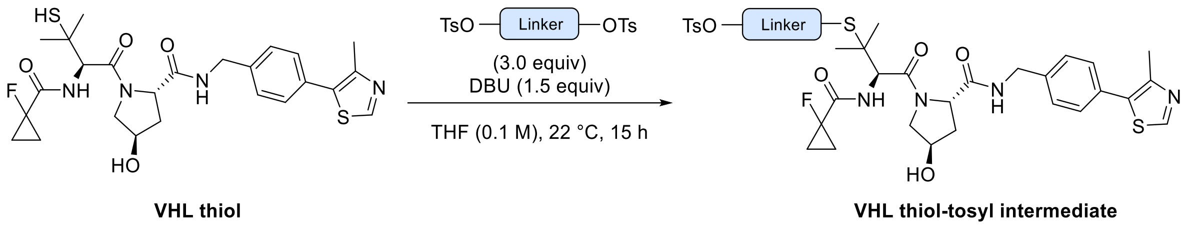

Synthesis of PROTACs

General procedure 5: synthesis of VHL phenol-based 'pyrazole' PROTACs from BAZ2 'pyrazole' warhead-tosyl intermediate and VHL phenol

BAZ2 'pyrazole' warhead-tosyl intermediate (1.0 equiv), **VHL phenol**[28] (1.2 equiv), K_2CO_3 (2.0 equiv) were dissolved in dry DMF (0.1 M). The reaction mixture was stirred at 80 ºC for 4 h. After this time, the organic solvent was removed under reduced pressure and the resulting residue triturated with Et_2O. The crude mixture was purified by preparative TLC (MeOH:DCM or MeOH:DCM:EtOAC) followed by trituration with Et_2O and pentane or by flash column chromatography to afford the corresponding **VHL phenol-based 'pyrazole' PROTAC**.

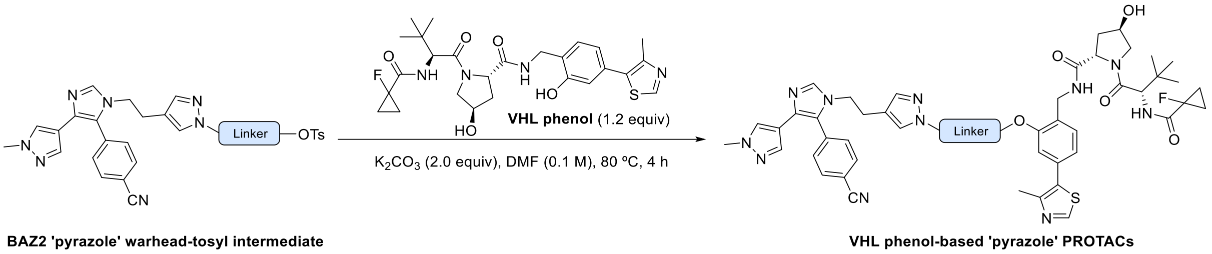

General procedure 6: synthesis of VHL thiol-based 'pyrazole' PROTACs from BAZ2 'pyrazole' warhead-tosyl intermediate and VHL thiol

BAZ2 'pyrazole' warhead-tosyl intermediate (1.0 equiv) and **VHL thiol**[29] (1.25 equiv) were dissolved in dry THF (0.1 M). Then, DBU (1.5 equiv) was added and the reaction mixture was stirred at 22 ºC for 12 h. After this time, the organic solvent was removed under reduced pressure and the resulting residue purified by preparative TLC followed by trituration with Et_2O and pentane or by or flash column chromatography to afford the corresponding **VHL thiol-based 'pyrazole' PROTAC**.

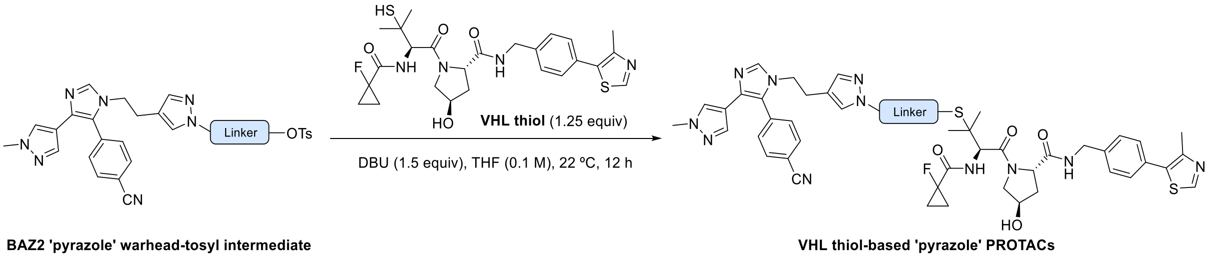

General procedure 7: synthesis of VHL phenol-based 'pyrazole' PROTACs from BAZ2 'pyrazole' warhead and VHL phenol-tosyl intermediate

BAZ2 'pyrazole' warhead (1.0 equiv) and NaH (1.3 equiv) were suspended in dry DMF (0.1 M) and the resulting suspension stirred at 0 °C for 1 hour. Then, a solution of **VHL phenol-tosyl intermediate** (1.4 equiv) was added and the reaction mixture stirred for 3 hours and allowed to warm up to 20 °C. After this time, the reaction was quenched with water, extracted with DCM and the organic layer washed with brine, dried over anhydrous $MgSO_4$, filtered and the solvent removed under reduced pressure. The crude product was purified by preparative TLC followed by trituration with Et_2O and pentane or by or flash

column chromatography to afford the corresponding **VHL phenol-based 'pyrazole' PROTAC**.

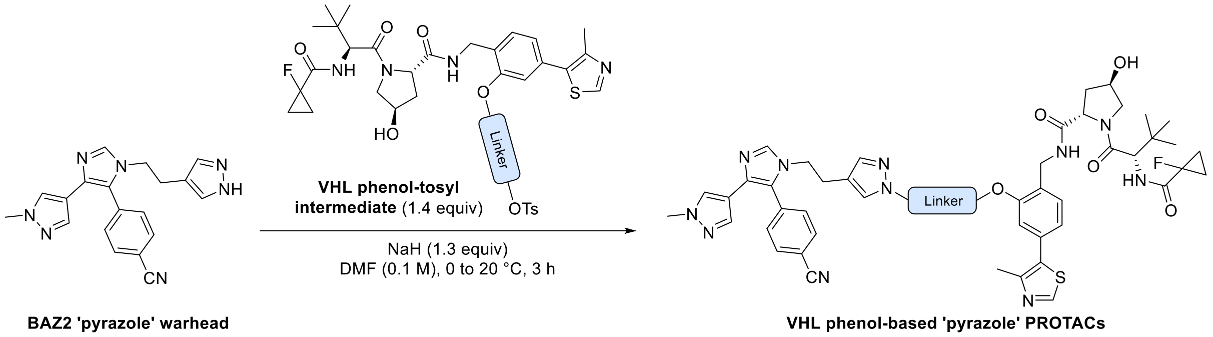

General procedure 8: synthesis of VHL thiol-based 'pyrazole' PROTACs from BAZ2 'pyrazole' warhead and VHL thiol-tosyl intermediate

BAZ2 'pyrazole' warhead (1.0 equiv) and NaH (1.3 equiv) were suspended in dry DMF (0.1 M) and the resulting suspension stirred at 0 °C for 1 hour. Then, a solution of **VHL thiol-tosyl intermediate** (1.4 equiv) was added and the reaction mixture stirred for 3 hours and allowed to warm up to 20 °C. After this time, the reaction was quenched with water, extracted with DCM and the organic layer washed with brine, dried over anhydrous $MgSO_4$, filtered and the solvent removed under reduced pressure. The crude product was purified by preparative TLC followed by trituration with Et_2O and pentane or by flash column chromatography to afford the corresponding **VHL thiol-based 'pyrazole' PROTAC**.

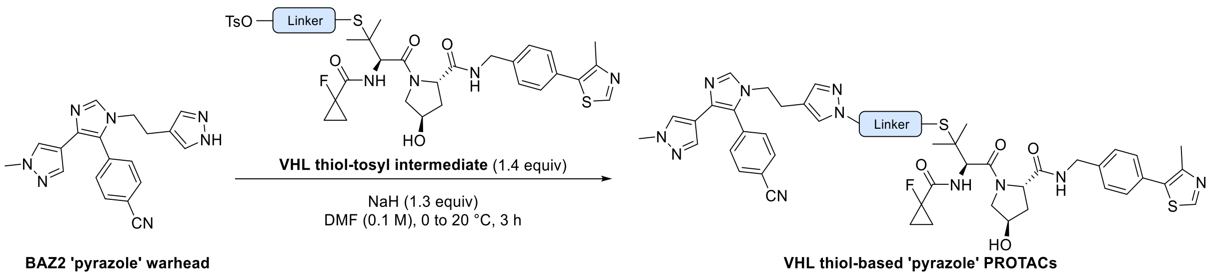

General procedure 9: synthesis of piperazine thalidomide-based 'pyrazole' PROTACs from BAZ2 'pyrazole' warhead-tosyl intermediate and pip-CRBN

BAZ2 'pyrazole' warhead-tosyl intermediate (1.0 equiv) and **pip-CRBN**[29] (1.5 equiv) were dissolved in DMF (0.1 M). Then, Et_3N (3.0 equiv) was added and the resulting reaction mixture stirred at 80 °C for 15 h. After this time, the organic solvent was removed under reduced pressure and the resulting residue was triturated with Et_2O to obtain a crude product which was purified by preparative TLC (MeOH:DCM or MeOH:DCM:EtOAC) followed by trituration with Et_2O and pentane to afford the corresponding **piperazine thalidomide-based 'pyrazole' PROTAC**.

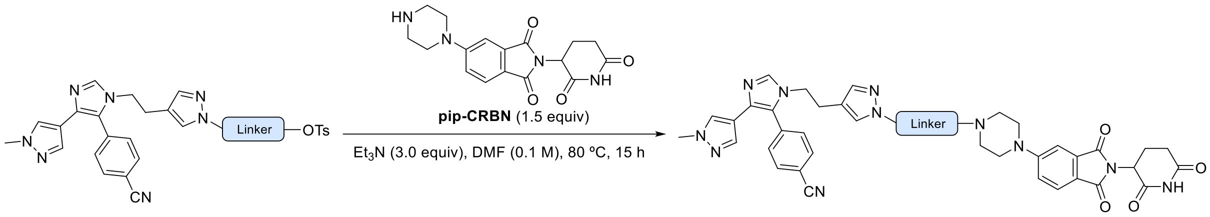

BAZ2 'pyrazole' warhead-tosyl intermediate

Piperazine thalidomide-based 'pyrazole' PROTACs

<u>General procedure 10</u>: synthesis of thalidomide-based PROTACs from BAZ2 'pyrazole' warhead

BAZ2 'pyrazole' warhead (1.0 equiv) and NaH (1.3 equiv) were suspended in dry DMF (0.1 M) and the resulting suspension stirred at 0 °C for 1 hour. Then, a solution of *N*-Boc-tosyl linker (1.4 equiv) was added and the reaction mixture stirred for 3 hours and allowed to warm up to 20 °C. After this time, the reaction was quenched with water, extracted with DCM and the organic layer washed with brine, dried over anhydrous $MgSO_4$, filtered and the solvent removed under reduced pressure to obtain the corresponding crude *N*-Boc intermediate which was used in the next step without further purification.

N-Boc intermediate obtained above (1.0 equiv) was dissolved in DCM (0.1 M) and TFA (100 equiv, protocol A) or a solution of HCl in 1,4-dioxane (4 M, 40 equiv, protocol B) was added. The reaction mixture was stirred at 20 ºC for 1-2.5 hours. After this time, excess of solvent was removed under reduced pressure to afford the corresponding TFA or hydrochloric salt of the amine which was triturated with ether and used in the next step without further purification.

Amine salt obtained above (1.0 equiv), 4-fluorothalidomide[30] (1.2-1.55 equiv) and DIPEA (6.0 equiv) were dissolved in DMSO (0.2 M) and the reaction mixture was stirred at 120 ºC for 15 hours. After this time, the solvent was removed under reduced pressure and the resulting residue purified by reversed phase HPLC to afford the corresponding **thalidomide-based 'pyrazole' PROTACs**.

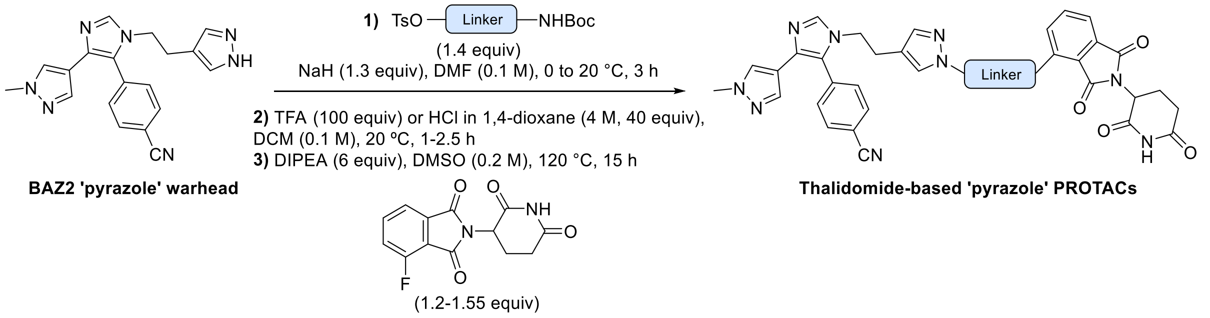

<u>General procedure 11:</u> synthesis of piperazine thalidomide-based PROTACs from BAZ2 'imidazole' warhead-tosyl intermediate and pip-CRBN

BAZ2 'imidazole' warhead-tosyl intermediate (1.0 equiv), **pip-CRBN**[29] (1.5 equiv) and NEt_3 (3.0 equiv) were dissolved in dry DMF (0.1 M) and the reaction mixture heated at 80 ºC for 5 h. After this time, the organic solvent was removed under reduced pressure and the resulting crude was purified by preparative TLC (MeOH: DCM) followed by trituration

in Et_2O and pentane to afford the corresponding **piperazine thalidomide-based 'imidazole' PROTACs.**

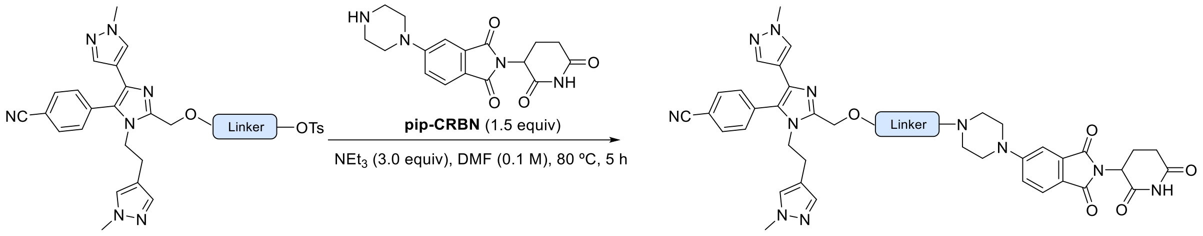

BAZ2 'imidazole' warhead-tosyl intermediate   Piperazine thalidomide-based 'imidazole' PROTACs

<u>General procedure 12:</u> synthesis of VHL phenol-based PROTACs from BAZ2 'imidazole' warhead-tosyl intermediate and VHL phenol

BAZ2 'imidazole' warhead-tosyl intermediate (1.0 equiv), **VHL phenol**[28] (1.2 equiv) and K_2CO_3 (2.0 equiv) were suspended in dry DMF (0.1 M) and the resulting suspension heated at 80 ºC for 3-6 h. After this time, the K_2CO_3 was filtered off and the organic solvent removed under reduced pressure. The crude product was purified by preparative TLC (MeOH:DCM or MeOH:DCM:EtOAc) followed by trituration with Et_2O and pentane to afford the corresponding **VHL phenol-based 'imidazole' PROTACs**.

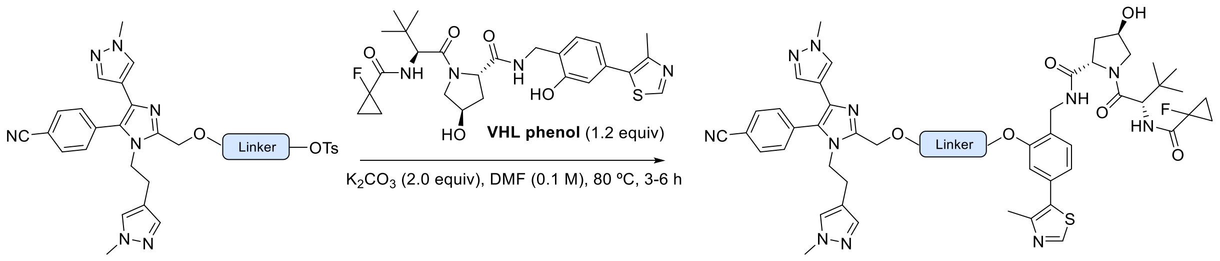

BAZ2 'imidazole' warhead-tosyl intermediate   VHL phenol-based 'imidazole' PROTACs

<u>General procedure 13:</u> synthesis of VHL thiol-based PROTACs from BAZ2 'imidazole' warhead-tosyl intermediate and VHL thiol

BAZ2 'imidazole' warhead-tosyl intermediate (1.0 equiv) and **VHL thiol**[29] (1.35 equiv) were dissolved in dry THF (0.1 M). DBU (1.5 equiv) was added and the reaction mixture stirred at 22 ºC for 15 hours. After this time, the organic solvent was removed under reduced pressure and the resulting crude product was purified by preparative TLC (MeOH:DCM or MeOH:DCM:EtOAc) followed by trituration with Et_2O and pentane to afford the corresponding **VHL thiol-based 'imidazole' PROTACs**.

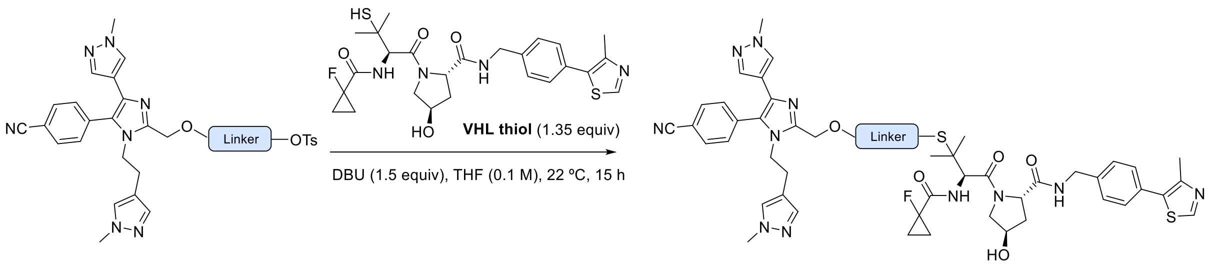

BAZ2 'imidazole' warhead-tosyl intermediate   VHL thiol-based 'imidazole' PROTACs

Experimental Procedures and Compound Characterization

Synthesis of BAZ2 'pyrazole' warhead

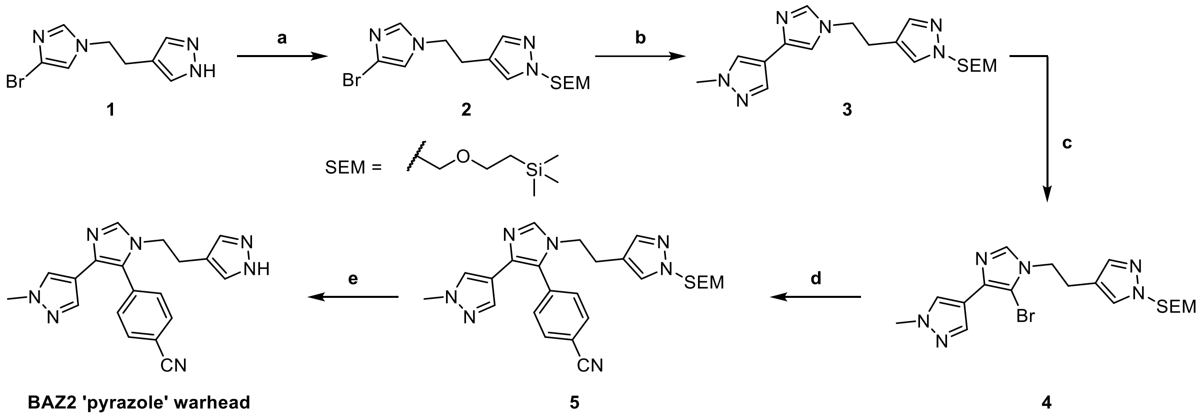

Scheme S1: a. NaH (1.5 equiv), SEM-Cl (1.4 equiv), THF, 0 to 20 °C, 14 h, 74%; **b**. 1-methylpyrazole-4-boronic acid pinacol ester (1.5 equiv), Pd(dppf)Cl_2 (10 mol%), cesium carbonate (3.0 equiv), 5:1 ethanol/water, 120 °C, 4 h, 91% yield; **c**. *N*-bromosuccinimide (1.0 equiv), DMF, 0 to 20 °C, 15 h, 71% yield; **d**. 4-cyanophenylboronic acid pinacol ester (1.5 equiv), Pd(dppf)Cl_2 (10 mol%), cesium carbonate (3.0 equiv), 5:1 1,4-dioxane/water, 120 °C, 4 h, 69% yield; **e**. TFA/DCM, 20 °C, 4 h, 79% yield.

Compound 2

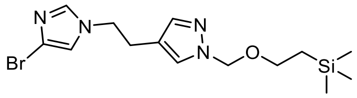

4-(2-(4-bromo-1*H*-imidazol-1-yl)ethyl)-1*H*-pyrazole [cpd S2 from Palaferri *et al.*][2] (2.65 g, 11.0 mmol, 1.0 equiv) and sodium hydride (60 % dispersion in mineral oil, 0.659 g, 16.5 mmol, 1.5 equiv) were flushed with nitrogen and suspended in dry THF (22 mL, 0.5 M) under nitrogen atmosphere and the mixture stirred at 0 °C for 30 minutes. Then SEM-chloride (2.72 mL, 15.4 mmol, 1.4 equiv) was added and the reaction mixture stirred for 14 hours, allowing it to warm to 20 °C. After this time, the reaction mixture was diluted with THF, filtered over cotton and concentrated under reduced pressure. The crude product was purified by flash chromatography (2% to 20% MeOH in DCM) to afford compound **2** as a colourless oil (3.02 g, 8.14 mmol, 74% yield). ^{1}H NMR (400 MHz, CD_2Cl_2) δ 7.27 (s, 1H), 7.24 (s, 1H), 7.21 (d, *J* = 1.6 Hz, 1H), 4.06 (t, *J* = 7.0 Hz, 2H), 3.55 – 3.46 (m, 2H), 2.92 (t, *J* = 6.4 Hz, 2H), 0.93 – 0.83 (m, 2H), -0.02 (s, 9H). ^{13}C NMR (126 MHz, CD_2Cl_2) δ 139.66, 137.16, 128.56, 118.41, 117.90, 115.32, 80.56, 66.94, 49.13, 26.57, 18.06, -1.41. HRMS (ESI): m/z calcd for $C_{14}H_{24}ON_4BrSi$ [M + H]$^+$: 371.08973; found 371.08981.

Compound 3

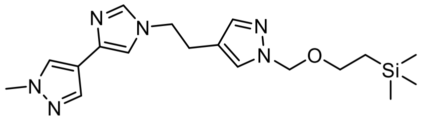

Compound **2** (1.65 g, 4.44 mmol, 1.0 equiv), 1-methylpyrazole-4-boronic acid pinacol ester (1.39 g, 6.66 mmol, 1.5 equiv), Pd(dppf)Cl_2 (0.325 g, 0.444 mmol, 10 mol%) and cesium carbonate (4.34 g, 13.3 mmol, 3.0 equiv) were flushed with nitrogen and dissolved in a degassed mixture of ethanol/water (5:1, 30 mL, 0.15 M). The reaction mixture was stirred at 120 °C for 4 hours. After this time, the mixture was allowed to cool 20 °C and diluted with ethyl acetate. Then, anhydrous magnesium sulphate was added and the mixture filtered over celite and concentrated under reduced pressure. The crude product was purified by flash chromatography (0% to 5% methanol in DCM) to afford compound **3** as a colourless oil (1.50 g, 4.03 mmol, 91% yield). ^{1}H NMR (400 MHz, CD_2Cl_2) δ 7.62 (s, 1H), 7.61 (s, 1H), 7.31 (d, *J* = 1.4 Hz, 1H), 7.27 (s, 1H), 7.24 (s, 1H), 6.97 (d, *J* = 1.3 Hz, 1H), 5.31 (s, 2H), 4.07 (t, *J* = 7.0 Hz, 2H), 3.87 (s, 3H), 3.54 – 3.46 (m, 2H), 2.95 (t, *J* = 7.1 Hz, 2H), 0.92 – 0.81 (m, 2H), -0.03 (s, 9H). ^{13}C NMR (126 MHz, CD_2Cl_2) δ 139.77, 137.26, 136.39, 135.72, 128.55, 126.94, 118.34, 117.89, 113.56, 80.52, 66.91, 48.56, 39.18, 26.72, 18.05, -1.41. HRMS (ESI): m/z calcd for $C_{18}H_{29}ON_6Si$ $[M + H]^+$: 373.21666; found 373.21680.

Compound 4

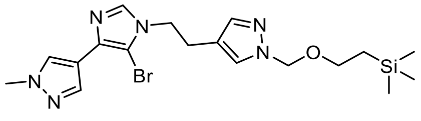

Compound **3** (212 mg, 0.569 mmol, 1.0 equv) was dissolved in dry DMF (5.7 mL, 0.1 M) at 0 °C. Then, a solution of *N*-bromosuccimide (101 mg, 0.569 mmol 1.0 equiv) in dry DMF (5.7 mL, 0.1 M) was added dropwise and the reaction mixture stirred for 15 hours, allowing it to warm to 20 °C. After this time, the reaction mixture was diluted with ice-cold water, extracted with ethyl acetate and the combined organic layers washed with a 5% LiCl aq. solution, and brine. The crude product was purified by flash chromatography (1% to 10% methanol in ethyl acetate) to afford compound **4** as a colourless oil (220 mg, 0.403 mmol, 71% yield). ^{1}H NMR (500 MHz, CD_2Cl_2) δ 7.90 (d, *J* = 0.8 Hz, 1H), 7.84 (d, *J* = 0.7 Hz, 1H), 7.38 (s, 1H), 7.29 (d, *J* = 0.7 Hz, 1H), 7.28 (d, *J* = 0.8 Hz, 1H), 5.32 (s, 2H), 4.09 (t, *J* = 7.2 Hz, 2H), 3.90 (s, 3H), 3.54 – 3.47 (m, 2H), 2.95 (t, *J* = 7.2 Hz, 2H), 0.91 – 0.83 (m, 2H), -0.02 (s, 9H). ^{13}C NMR (126 MHz, CD_2Cl_2) δ 139.84, 137.83, 136.94, 133.87, 128.67, 127.57, 117.86, 116.32, 98.12, 80.52, 66.90, 47.72, 39.25, 25.89, 18.05, -1.40. HRMS (ESI): m/z calcd for $C_{18}H_{28}ON_6SiBr$ $[M + H]^+$: 451.12718; found 451.12737.

Compound 5

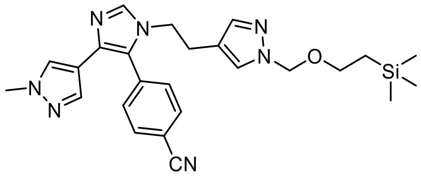

Compound **4** (1.23 g, 2.72 mmol, 1.0 equiv), 4-cyanophenylboronic acid pinacol ester (934 mg, 4.08 mmol, 1.5 equiv), Pd(dppf)Cl_2 (199 mg, 0.272 mmol, 10 mol%) and cesium carbonate (2.66 g, 8.15 mmol, 3.0 equiv) were flushed with nitrogen and dissolved in a degassed mixture of 1,4-dioxane/water (5:1, 12 mL, 0.1 M). The reaction mixture was stirred at 120 °C for 4 hours. After this time, the mixture was allowed to cool to 20 °C and diluted with DCM. Then, anhydrous magnesium sulphate was added and the mixture filtered over celite and concentrated under reduced pressure. The crude product was purified by flash chromatography (2% to 10% ethanol in DCM) to afford compound **5** as an off white solid (884 mg, 1.87 mmol, 69% yield). ^{1}H NMR (500 MHz, CD_2Cl_2) δ 7.77 (d, *J* = 8.3 Hz, 2H), 7.45 (s, 1H), 7.42 (d, *J* = 8.3 Hz, 2H), 7.33 (s, 1H), 7.26 (s, 1H), 7.13 (s, 1H), 7.09 (s, 1H), 5.28 (s, 2H), 3.96 (t, *J* = 7.1 Hz, 2H), 3.78 (s, 3H), 3.52 – 3.46 (m, 2H), 2.68 (t, *J* = 7.1 Hz, 2H), 0.89 – 0.83 (m, 2H), -0.04 (s, 9H). Cyclohexane impurity: 1.43 ppm. ^{13}C NMR (126 MHz, CD_2Cl_2) δ 139.68, 138.00, 136.83, 135.92, 133.57, 133.21, 131.65, 128.46, 127.51, 125.17, 118.83, 117.79, 117.07, 112.69, 80.48, 66.98, 46.78, 39.14, 26.28, 18.04, -1.43. Cyclohexane impurity: 27.30 ppm. HRMS (ESI): m/z calcd for $C_{25}H_{32}ON_7Si$ $[M + H]^+$: 474.24321; found 474.24290.

BAZ2 ‘pyrazole’ warhead

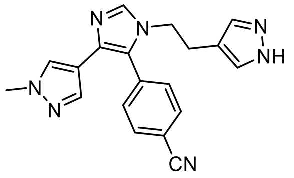

Compound **5** (957 mg, 2.02 mmol, 1.0 equiv) was dissolved in dry DCM under nitrogen atmosphere. Trifluoroacetic acid (6.5 mL, 87.5 mmol, 43 equiv) was then added and the reaction mixture stirred at 20 °C for 4 hours. After this time the solvent was flushed with nitrogen and the residue taken up in a solution of sat. aq. sodium carbonate, extracted with DCM and the combined organic layers washed with brine and concentrated under reduced pressure. The crude product was purified by flash chromatography (2% to 15% methanol in DCM) to afford **BAZ2 ‘pyrazole’ warhead** as a white solid (547 mg, 1.59 mmol, 79% yield). ^{1}H NMR (500 MHz, CD_2Cl_2) δ 7.79 – 7.73 (m, 2H), 7.45 (s, 1H), 7.44 – 7.37 (m, 2H), 7.33 (d, *J* = 0.8 Hz, 1H), 7.27 (d, *J* = 0.8 Hz, 1H), 7.14 (s, 2H), 3.97 (t, *J* = 6.9 Hz, 2H), 3.78 (s, 3H), 2.70 (t, *J* = 6.9 Hz, 2H). ^{13}C NMR (126 MHz, CD_2Cl_2) δ 138.11, 136.87, 135.86, 133.46, 133.22, 131.67, 127.56, 125.31, 118.85, 116.99, 116.12, 112.72, 46.93, 39.14, 26.13. HRMS (ESI): m/z calcd for $C_{19}H_{18}N_7$ $[M + H]^+$: 344.16182; found 344.16159.

Synthesis of BAZ2 'pyrazole' warhead-tosyl intermediates

Compound S1

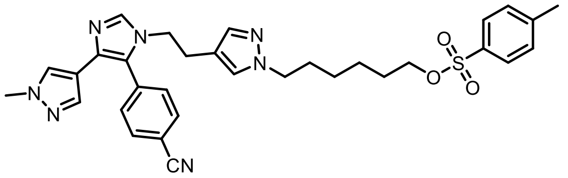

S1 (37 mg, 71%, white solid) was prepared from **BAZ2 'pyrazole' warhead** (30 mg, 0.0874 mmol, 1.0 equiv) and ditosyl linker hexane-1,6-diyl bis(4-methylbenzenesulfonate)[31] according to general procedure 1 and purified with flash column chromatography (1% to 12% MeOH in DCM). ^{1}H NMR (400 MHz, CD_2Cl_2) δ 7.78 – 7.73 (m, 4H), 7.43 (s, 1H), 7.43 – 7.41 (m, 1H), 7.41 – 7.39 (m, 1H), 7.38 – 7.37 (m, 1H), 7.36 – 7.35 (m, 1H), 7.33 (s, 1H), 7.25 (d, *J* = 0.8 Hz, 1H), 7.02 (s, 1H), 6.91 (s, 1H), 3.99 – 3.90 (m, 6H), 3.78 (s, 3H), 2.64 (t, *J* = 7.0 Hz, 2H), 2.44 (s, 3H), 1.71 (p, *J* = 7.3 Hz, 2H), 1.59 (dt, *J* = 14.5, 6.5 Hz, 2H), 1.33 – 1.24 (m, 2H), 1.23 – 1.11 (m, 2H). ^{13}C NMR (101 MHz, CD_2Cl_2) δ 145.33, 138.55, 138.04, 136.77, 135.92, 133.42, 133.38, 133.14, 131.61, 130.22, 128.12, 127.92, 127.46, 125.16, 118.84, 117.06, 116.30, 112.58, 70.99, 52.18, 46.92, 39.11, 30.51, 28.96, 26.25, 26.22, 25.23, 21.73. HRMS (ESI): m/z calcd for $C_{32}H_{36}N_7O_3S$ $[M + H]^+$: 598.25912; found 598.25911.

Compound S2

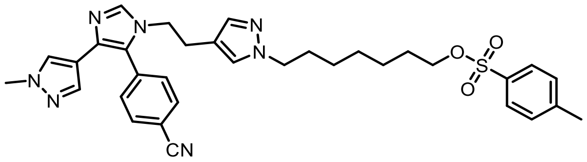

S2 (39 mg, 73%, white solid) was prepared from **BAZ2 'pyrazole' warhead** (30 mg, 0.0874 mmol, 1.0 equiv) and ditosyl linker heptane-1,7-diyl bis(4-methylbenzenesulfonate)[32] according to general procedure 1 and purified with flash column chromatography (1% to 8% MeOH in DCM). ^{1}H NMR (400 MHz, CD_2Cl_2) δ 7.78 – 7.73 (m, 4H), 7.44 (s, 1H), 7.43 – 7.39 (m, 2H), 7.38 – 7.36 (m, 1H), 7.36 – 7.34 (m, 1H), 7.32 (s, 1H), 7.25 (d, *J* = 0.7 Hz, 1H), 7.02 (s, 1H), 6.92 (s, 1H), 4.00 – 3.90 (m, 6H), 3.78 (s, 3H), 2.65 (t, *J* = 7.0 Hz, 2H), 2.44 (s, 3H), 1.75 – 1.67 (m, 2H), 1.63 – 1.55 (m, 2H), 1.29 – 1.16 (m, 6H). ^{13}C NMR (101 MHz, CD_2Cl_2) δ 145.31, 138.51, 138.02, 136.79, 135.92, 133.41, 133.14, 131.62, 130.21, 128.13, 127.90, 127.46, 126.11, 125.19, 118.84, 117.06, 116.27, 112.58, 71.11, 52.31, 46.93, 39.11, 30.61, 29.01, 28.72, 26.63, 26.29, 25.50, 21.73. HRMS (ESI): m/z calcd for $C_{33}H_{38}N_7O_3S$ $[M + H]^+$: 612.27530; found 612.27528.

Compound S3

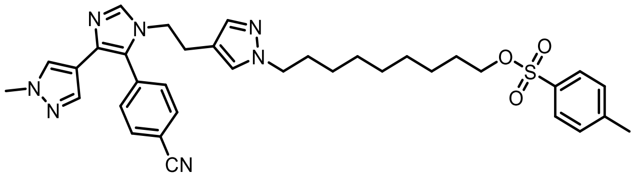

S3 (34 mg, 61%, white solid) was prepared from **BAZ2 'pyrazole' warhead** (30 mg, 0.0874 mmol, 1.0 equiv) and ditosyl linker nonane-1,9-diyl bis(4-methylbenzenesulfonate)[31] according to general procedure 1 and purified with flash column chromatography (1% to 4% MeOH in DCM). ^{1}H NMR (400 MHz, CD_2Cl_2) δ 7.77 (d, *J* = 1.9 Hz, 2H), 7.75 (d, *J* = 2.0 Hz, 2H), 7.44 (s, 1H), 7.41 (s, 1H), 7.39 (s, 1H), 7.37 (s, 1H), 7.35 (s, 1H), 7.32 (s, 1H), 7.25 (s, 1H), 7.02 (s, 1H), 6.93 (s, 1H), 4.01 – 3.90 (m, 7H), 3.78 (s, 3H), 2.65 (t, *J* = 7.0 Hz, 2H), 2.44 (s, 3H), 1.79 – 1.65 (m, 4H), 1.60 (p, *J* = 6.6 Hz, 2H), 1.31 – 1.15 (m, 7H). ^{13}C NMR (126 MHz, CD_2Cl_2) δ 145.30, 138.52, 138.03, 136.85, 135.98, 133.57, 133.49, 133.16, 131.68, 130.23, 128.17, 127.88, 127.50, 125.26, 118.86, 117.13, 116.29, 112.65, 71.25, 52.48, 46.98, 39.14, 30.80, 29.56, 29.32, 29.18, 29.16, 26.88, 26.37, 25.65, 21.76. HRMS (ESI): m/z calcd for $C_{35}H_{42}N_7O_3S$ [M + H]$^+$: 640.30615; found 640.30615.

Compound S4

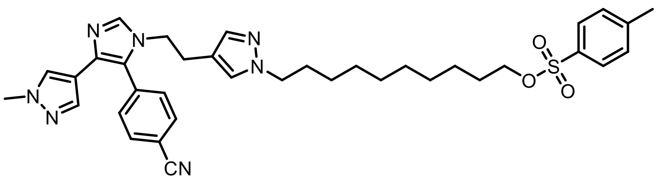

S4 (35 mg, 61%, white solid) was prepared from **BAZ2 'pyrazole' warhead** (30 mg, 0.0874 mmol, 1.0 equiv) and ditosyl linker decane-1,10-diyl bis(4-methylbenzenesulfonate)[33] according to general procedure 1 and purified with flash column chromatography (1% to 4% MeOH in DCM). ^{1}H NMR (400 MHz, CD_2Cl_2) δ 7.77 (d, *J* = 1.9 Hz, 2H), 7.75 (d, *J* = 1.8 Hz, 2H), 7.45 (s, 1H), 7.41 (s, 1H), 7.39 (s, 1H), 7.37 (s, 1H), 7.35 (s, 1H), 7.32 (s, 1H), 7.25 (d, *J* = 0.8 Hz, 1H), 7.02 (s, 1H), 6.92 (s, 1H), 4.02 – 3.89 (m, 7H), 3.78 (s, 3H), 2.65 (t, *J* = 7.0 Hz, 2H), 2.44 (s, 3H), 1.74 (q, *J* = 7.2 Hz, 2H), 1.59 (q, *J* = 7.0 Hz, 4H), 1.22 (d, *J* = 11.8 Hz, 13H). ^{13}C NMR (126 MHz, CD_2Cl_2) δ 144.89, 138.10, 137.62, 136.45, 135.57, 133.17, 133.08, 132.75, 131.27, 129.82, 127.76, 127.47, 127.10, 124.86, 118.46, 116.72, 115.88, 112.25, 70.87, 52.10, 46.58, 38.73, 30.43, 29.26, 29.25, 29.03, 28.83, 28.76, 26.53, 25.97, 25.28, 21.35. HRMS (ESI): m/z calcd for $C_{36}H_{44}N_7O_3S$ [M + H]$^+$: 654.32209; found 654.32206.

Compound S5

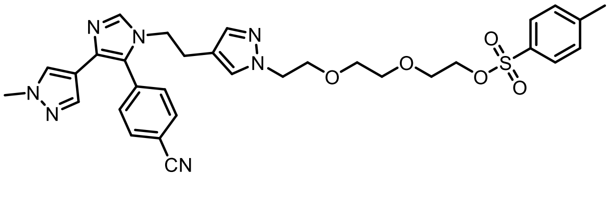

S5 (48 mg 87%, colourless oil) was prepared from **BAZ2 'pyrazole' warhead** (30 mg, 0.0874 mmol, 1.0 equiv) and ditosyl linker (ethane-1,2-diylbis(oxy))bis(ethane-2,1-diyl) bis(4-methylbenzenesulfonate)[34] according to general procedure 1 and purified with flash column chromatography (3% to 10% MeOH in DCM). ^{1}H NMR (500 MHz, CD_2Cl_2) δ 7.78 – 7.74 (m, 4H), 7.46 (s, 1H), 7.41 (d, *J* = 8.1 Hz, 2H), 7.36 (d, *J* = 8.1 Hz, 2H), 7.33 (s, 1H), 7.25 (s, 1H), 7.08 (s, 1H), 7.03 (s, 1H), 4.16 (t, *J* = 5.3 Hz, 2H), 4.10 – 4.06 (m, 2H), 3.95

(t, J = 7.0 Hz, 2H), 3.78 (s, 3H), 3.73 (t, J = 5.3 Hz, 2H), 3.59 – 3.57 (m, 2H), 3.50 – 3.44 (m, 4H), 2.66 (t, J = 7.0 Hz, 2H), 2.44 (s, 3H). ^{13}C NMR (126 MHz, CD_2Cl_2) δ 145.55, 138.87, 138.06, 136.81, 135.94, 133.36, 133.23, 133.16, 131.70, 130.28, 129.04, 128.22, 127.49, 125.23, 118.87, 117.10, 116.54, 112.63, 70.90, 70.74, 70.17, 69.85, 68.94, 52.43, 46.92, 39.12, 26.30, 21.77. HRMS (ESI): m/z calcd for $C_{32}H_{36}N_7O_5S$ $[M + H]^+$: 630.24931; found 630.24962.

Compound S6

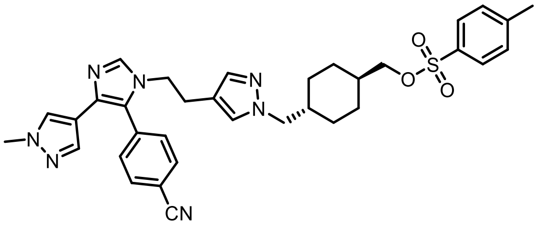

S6 (30 mg, 55%, white solid) was prepared from **BAZ2 'pyrazole' warhead** (30 mg, 0.0874 mmol, 1.0 equiv) and ditosyl linker ((1r,4r)-cyclohexane-1,4-diyl)bis(methylene) bis(4-methylbenzenesulfonate)[2] according to general procedure 1 and purified with flash column chromatography (1% to 7% MeOH in DCM). ^1H NMR (500 MHz, CD_2Cl_2) δ 7.78 – 7.72 (m, 4H), 7.44 – 7.39 (m, 3H), 7.36 (dd, J = 8.6, 0.8 Hz, 2H), 7.32 (d, J = 0.9 Hz, 1H), 7.25 (d, J = 0.9 Hz, 1H), 7.02 (d, J = 0.8 Hz, 1H), 6.87 (d, J = 0.8 Hz, 1H), 3.93 (t, J = 7.0 Hz, 2H), 3.81 – 3.76 (m, 7H), 2.63 (t, J = 7.0 Hz, 2H), 2.44 (s, 3H), 1.73 – 1.51 (m, 6H; overlapping with water), 0.95 – 0.82 (m, 4H). ^{13}C NMR (126 MHz, CD_2Cl_2) δ 145.3, 138.6, 138.1, 136.8, 136.0, 133.5, 133.4, 133.2, 131.7, 130.2, 128.6, 128.2, 127.5, 125.2, 118.9, 117.1, 116.2, 112.7, 75.5, 58.5, 47.0, 39.1, 39.0, 37.5, 29.9, 28.7, 26.2, 21.8. HRMS (ESI): m/z calcd for $C_{34}H_{38}N_7O_5S$ $[M + H]^+$: 624.27514; found 624.27535.

Synthesis of BAZ2 'imidazole' warhead-tosyl intermediates

Compound S7

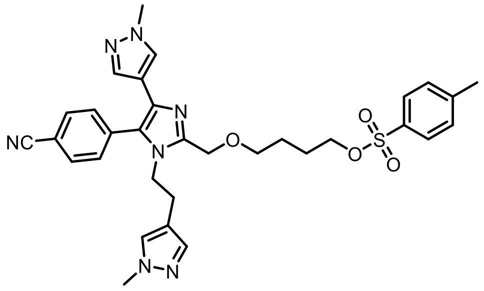

S7 (39 mg, 70%, white solid) was prepared from **BAZ2 'imidazole' warhead** [cpd 1 from Palaferri *et al.*][2] (35 mg, 0.0903 mmol, 1.0 equiv) and ditosyl linker butane-1,4-diyl bis(4-methylbenzenesulfonate)[35] according to general procedure 2 and purified with flash column chromatography (0.2% to 1% MeOH in DCM). ^{1}H NMR (400 MHz, CD_2Cl_2) δ 7.79 – 7.72 (m, 4H), 7.43 – 7.41 (m, 1H), 7.41 – 7.39 (m, 1H), 7.37 – 7.35 (m, 1H), 7.35 – 7.33 (m, 1H), 7.29 (s, 1H), 7.22 (d, *J* = 0.8 Hz, 1H), 6.93 (s, 1H), 6.87 (s, 1H), 4.46 (s, 2H), 4.00 (q, *J* = 6.8 Hz, 4H), 3.77 (s, 3H), 3.76 (s, 3H), 3.50 (t, *J* = 6.1 Hz, 2H), 2.59 (t, *J* = 7.4 Hz, 2H), 2.43 (s, 3H), 1.77 – 1.67 (m, 2H), 1.67 – 1.56 (m, 4H). ^{13}C NMR (126 MHz, CD_2Cl_2) δ 145.40, 138.73, 136.87, 136.15, 133.41, 133.14, 132.07, 131.77, 130.34, 130.27, 128.86, 128.80, 128.14, 127.49, 126.57, 118.85, 116.88, 112.73, 70.87, 70.06, 65.57, 45.86, 39.12, 39.07, 26.22, 26.08, 25.95, 21.74. HRMS (ESI): m/z calcd for $C_{32}H_{36}N_7O_4S$ [M + H]$^+$: 614.25384; found 614.25384.

Compound S8

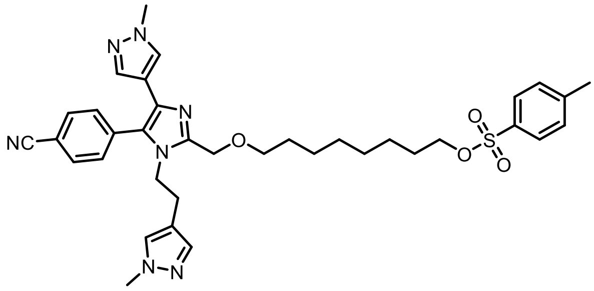

S8 (28 mg, 54%, white solid) was prepared from **BAZ2 'imidazole' warhead** [cpd 1 from Palaferri *et al.*][2] (30 mg, 0.0774 mmol, 1.0 equiv) and ditosyl linker octane-1,8-diyl bis(4-methylbenzenesulfonate)[36] according to general procedure 2 and purified with flash column chromatography (1% to 7% MeOH in DCM). ^{1}H NMR (500 MHz, CD_2Cl_2) δ 7.79 – 7.73 (m, 4H), 7.40 (d, *J* = 8.3 Hz, 2H), 7.36 (d, *J* = 8.3 Hz, 2H), 7.29 (s, 1H), 7.22 (s, 1H), 6.95 (s, 1H), 6.86 (s, 1H), 4.49 (s, 2H), 4.03 – 3.99 (m, 2H), 3.97 (t, *J* = 6.6 Hz, 2H), 3.77 (s, 3H), 3.75 (s, 3H), 3.51 (t, *J* = 6.6 Hz, 2H), 2.61 (t, *J* = 7.5 Hz, 2H), 2.44 (s, 3H), 1.66 – 1.51 (m, 4H), 1.39 – 1.17 (m, 8H). ^{13}C NMR (126 MHz, CD_2Cl_2) δ 145.7, 145.3, 138.8, 136.9, 136.2, 133.6, 133.1, 132.0, 131.8, 130.2, 128.8, 128.2, 127.5, 126.5, 118.9, 117.0, 116.9, 112.7, 71.2, 71.2, 65.6, 45.9, 39.1, 39.1, 30.0, 29.6, 29.2, 29.2, 26.4, 26.0, 25.7, 21.8. HRMS (ESI): m/z calcd for $C_{36}H_{44}N_7O_4S$ [M + H]$^+$: 670.31700; found 670.31661.

Compound S9

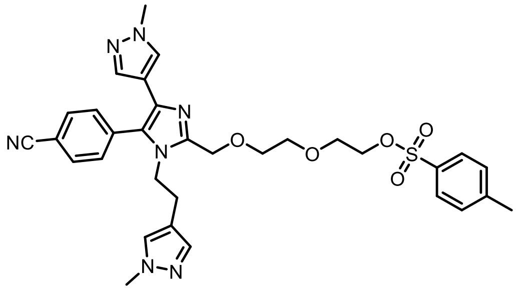

S9 (40 mg, 75%, white solid) was prepared from **BAZ2 'imidazole' warhead** [cpd 1 from Palaferri *et al.*][2] (33 mg, 0.0852 mmol, 1.0 equiv) and distoyl linker oxybis(ethane-2,1-diyl) bis(4-methylbenzenesulfonate)[37] according to general procedure 2 and purified by flash column chromatography (2% to 8% MeOH in DCM). ^{1}H NMR (400 MHz, CD_2Cl_2) δ 7.79 – 7.73 (m, 4H), 7.41 (s, 1H), 7.39 (s, 1H), 7.36 (s, 1H), 7.34 (s, 1H), 7.29 (s, 1H), 7.22 (s, 1H), 6.94 (s, 1H), 6.89 (s, 1H), 4.53 (s, 2H), 4.11 – 4.07 (m, 2H), 4.07 – 3.99 (m, 2H), 3.78 (s, 3H), 3.75 (s, 3H), 3.68 – 3.59 (m, 4H), 3.62 – 3.55 (m, 2H), 2.61 (t, *J* = 7.4 Hz, 2H), 2.42 (s, 3H). ^{13}C NMR (126 MHz, CD_2Cl_2) δ 145.56, 145.26, 138.82, 136.89, 136.14, 133.25, 133.14, 132.05, 131.80, 130.30, 128.22, 127.52, 126.68, 118.87, 117.01, 116.87, 112.72, 70.90, 69.92, 69.80, 68.99, 65.78, 45.89, 39.14, 39.06, 25.89, 21.77. HRMS (ESI): m/z calcd for $C_{32}H_{36}N_7O_5S$ $[M + H]^+$: 630.24962; found 630.24978.

Compound S10

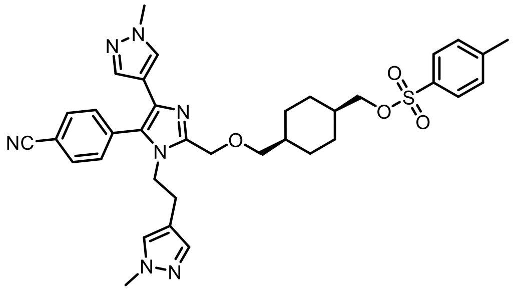

S10 (40 mg, 52%, white solid) was prepared from **BAZ2 'imidazole' warhead** [cpd 1 from Palaferri *et al.*][2] (45 mg, 0.116 mmol, 1.0 equiv) and ditosyl linker ((1s,4s)-cyclohexane-1,4-diyl)bis(methylene) bis(4-methylbenzenesulfonate)[38] according to general procedure 2 and purified by flash column chromatography (0.2% to 1% MeOH in DCM). ^{1}H NMR (400 MHz, CD_2Cl_2) δ 7.79 – 7.72 (m, 4H), 7.42 – 7.34 (m, 4H), 7.29 (s, 1H), 7.22 (d, *J* = 0.8 Hz, 1H), 6.93 (d, *J* = 0.9 Hz, 1H), 6.86 (s, 1H), 4.47 (s, 2H), 4.03 – 3.97 (m, 2H), 3.89 (d, *J* = 7.1 Hz, 2H), 3.78 (s, 3H), 3.75 (s, 3H), 3.39 (d, *J* = 7.0 Hz, 2H), 2.66 – 2.56 (m, 2H), 2.44 (s, 3H), 1.89 – 1.74 (m, 2H), 1.53 – 1.41 (m, 4H), 1.38 – 1.28 (m, 4H). ^{13}C NMR (126 MHz, CD_2Cl_2) δ 145.59, 145.35, 138.76, 136.88, 136.18, 133.48, 133.15, 132.03, 131.78, 130.26, 128.77, 128.14, 127.51, 126.59, 118.87, 116.95, 116.90, 112.73, 74.04, 73.71, 65.83, 45.86, 39.14, 39.08, 35.62, 35.20, 25.96, 25.70, 25.29, 21.77. HRMS (ESI): m/z calcd for $C_{36}H_{42}N_7O_4S$ $[M + H]^+$: 668.30135; found 668.30169.

Synthesis of VHL phenol-tosyl intermediates

Compound S11

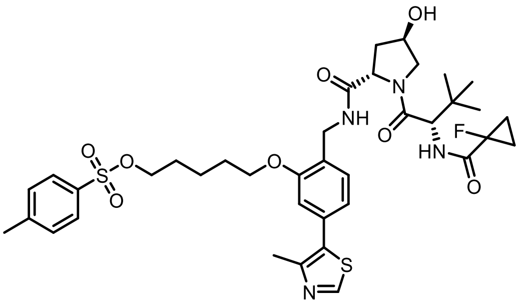

S11 (42 mg, 72%, white solid) was prepared from **VHL phenol**[28] (40 mg, 0.0751 mmol, 1.0 equiv) and ditosyl linker pentane-1,5-diyl bis(4-methylbenzenesulfonate)[39] according to general procedure 3 and purified by flash column chromatography (2% to 5% MeOH in DCM). ^{1}H NMR (500 MHz, CD_2Cl_2) δ 8.66 (s, 1H), 7.79 – 7.73 (m, 2H), 7.38 – 7.30 (m, 3H), 7.13 (t, *J* = 6.1 Hz, 1H), 7.03 – 6.98 (m, 1H), 6.96 (dd, *J* = 7.6, 1.6 Hz, 1H), 6.87 (s, 1H), 4.67 (t, *J* = 7.7 Hz, 1H), 4.55 – 4.44 (m, 3H), 4.34 (dd, *J* = 15.0, 5.2 Hz, 1H), 4.05 (t, *J* = 6.4 Hz, 2H), 4.00 – 3.94 (m, 2H), 3.92 (d, *J* = 11.3 Hz, 1H), 3.61 (dd, *J* = 11.2, 4.0 Hz, 1H), 2.69 (bs, 1H), 2.50 (s, 3H), 2.47 (s, 1H), 2.42 (s, 3H), 2.09 – 2.01 (m, 1H), 1.81 (p, *J* = 6.6 Hz, 2H), 1.73 (p, *J* = 6.7 Hz, 2H), 1.58 – 1.49 (m, 2H), 1.36 – 1.22 (m, 4H), 0.92 (s, 9H). ^{13}C NMR (126 MHz, CD_2Cl_2) δ 171.32, 170.77, 170.30 (d, *J* = 20.3 Hz), 157.12, 150.51, 148.98, 145.40, 133.48, 132.67, 132.13, 130.28, 129.58, 128.18, 126.86, 121.74, 112.41, 78.71 (d, *J* = 232.4 Hz), 71.04, 70.66, 68.17, 66.07, 58.88, 57.75, 56.89, 38.97, 36.43, 35.63, 28.99, 26.43, 22.59, 21.75, 16.38, 13.92 (d, *J* = 10.0 Hz), 13.80 (d, *J* = 10.5 Hz). HRMS (ESI): m/z calcd for $C_{38}H_{50}FN_4O_8S_2$ $[M + H]^+$: 773.30394; found 773.30415.

Compound S12

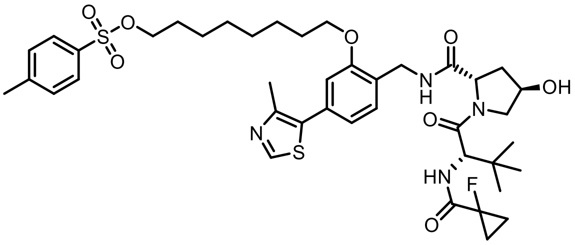

S12 (30 mg, 49%, white solid) was prepared from **VHL phenol**[28] (40 mg, 0.0751 mmol, 1.0 equiv) and ditosyl linker octane-1,8-diyl bis(4-methylbenzenesulfonate)[36] according to general procedure 3 and purified by flash column chromatography (1% to 5% MeOH in DCM). ^{1}H NMR (500 MHz, CD_2Cl_2) δ 8.66 (s, 1H), 7.81 – 7.70 (m, 2H), 7.39 – 7.34 (m, 2H), 7.32 (d, *J* = 7.8 Hz, 1H), 7.14 – 7.09 (m, 1H), 6.99 – 6.94 (m, 2H), 6.90 (d, *J* = 1.6 Hz, 1H), 4.68 (t, *J* = 7.7 Hz, 1H), 4.54 – 4.44 (m, 3H), 4.36 (dd, *J* = 14.9, 5.2 Hz, 1H), 4.00 (dt, *J* = 8.9, 6.5 Hz, 4H), 3.93 (dt, *J* = 11.3, 2.1 Hz, 1H), 3.61 (dd, *J* = 11.2, 4.1 Hz, 1H), 2.56 – 2.47 (m, 4H), 2.44 (s, 3H), 2.33 (d, *J* = 4.5 Hz, 1H), 2.04 (dddd, *J* = 13.5, 8.3, 2.9, 1.7 Hz, 1H), 1.83 (dq, *J* = 9.1, 6.4 Hz, 2H), 1.63 (dq, *J* = 8.1, 6.6 Hz, 2H), 1.51 – 1.42 (m, 2H), 1.40 – 1.22 (m, 10H), 0.92 (s, 9H). ^{13}C NMR (126 MHz, CD_2Cl_2) δ 171.4, 170.6, 170.3 (d, *J* = 20.4 Hz), 157.3, 150.5, 149.0, 145.3, 133.6, 132.7, 132.2, 130.3, 129.6, 128.2, 126.9, 121.6, 112.5, 78.7

(d, J = 231.9 Hz), 71.3, 70.7, 68.6, 58.8, 57.8, 56.8, 39.0, 36.3, 35.5, 29.6, 29.6, 29.3, 29.2, 26.5, 26.4, 25.7, 21.8, 16.4, 13.91 (d, J = 10.5 Hz), 13.83 (d, J = 10.0 Hz). HRMS (ESI): m/z calcd for $C_{41}H_{56}N_4O_8S_2F$ $[M + H]^+$: 815.35181; found 815.35155.

Compound S13

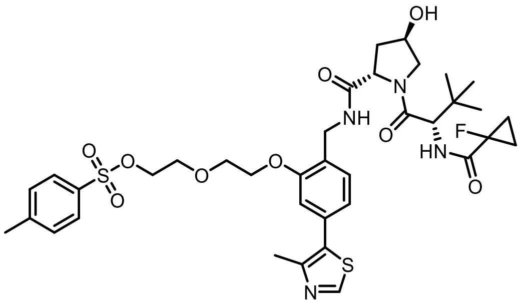

S13 (48 mg, 82%, white solid) was prepared from **VHL phenol**[28] (40 mg, 0.0751 mmol, 1.0 equiv) and ditosyl linker oxybis(ethane-2,1-diyl) bis(4-methylbenzenesulfonate)[37] according to general procedure 3 and purified by flash column chromatography (2% to 6% MeOH in DCM). ^{1}H NMR (500 MHz, CD_2Cl_2) δ 8.66 (s, 1H), 7.76 (d, J = 8.0 Hz, 2H), 7.34 (dd, J = 8.0, 3.1 Hz, 3H), 7.15 (t, J = 6.1 Hz, 1H), 7.02 – 6.96 (m, 2H), 6.91 (s, 1H), 4.62 (t, J = 7.9 Hz, 1H), 4.55 – 4.43 (m, 3H), 4.37 (dd, J = 14.9, 5.5 Hz, 1H), 4.17 (t, J = 4.7 Hz, 2H), 4.16 – 4.10 (m, 1H), 3.92 (d, J = 11.1 Hz, 1H), 3.88 – 3.81 (m, 1H), 3.79 – 3.75 (m, 1H), 3.63 (dd, J = 11.2, 3.9 Hz, 1H), 2.50 (s, 3H), 2.46 – 2.35 (m, 3H), 2.12 – 2.02 (m, 1H), 1.32 – 1.23 (m, 4H), 0.92 (s, 9H). ^{13}C NMR (126 MHz, CD_2Cl_2) δ 171.13, 170.91, 170.19 (d, J = 20.4 Hz), 157.07, 150.55, 149.07, 145.59, 133.22, 132.76, 131.98, 130.29, 129.90, 128.27, 127.36, 122.28, 113.14, 78.71 (d, J = 231.9 Hz), 70.73, 70.12, 69.95, 69.29, 68.37, 58.97, 57.73, 56.94, 39.16, 36.77, 35.69, 26.45, 21.76, 16.39, 13.88 (d, J = 10.0 Hz), 13.76 (d, J = 10.5 Hz). HRMS (ESI): m/z calcd for $C_{37}H_{48}FN_4O_9S_2$ $[M + H]^+$: 775.28332; found 775.28332.

Compound S14

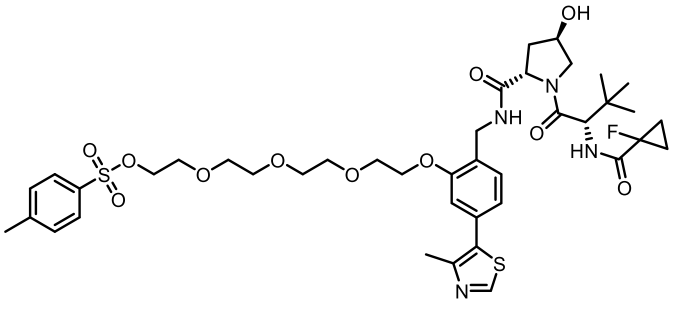

S14 (44 mg, 68%, white solid) was prepared from **VHL phenol**[28] (40 mg, 0.0751 mmol, 1.0 equiv) and ditosyl linker ((oxybis(ethane-2,1-diyl))bis(oxy))bis(ethane-2,1-diyl) bis(4-methylbenzenesulfonate)[40] according to general procedure 3 and purified by flash column chromatography (2% to 6% MeOH in DCM). ^{1}H NMR (500 MHz, CD_2Cl_2) δ 8.66 (s, 1H), 7.77 (d, J = 8.0 Hz, 1H), 7.35 (dd, J = 13.2, 7.8 Hz, 2H), 7.21 (t, J = 6.1 Hz, 1H), 7.03 – 6.97 (m, 2H), 6.95 (s, 1H), 4.61 (t, J = 7.9 Hz, 1H), 4.54 (d, J = 8.9 Hz, 1H), 4.51 – 4.46 (m, 1H), 4.43 (dd, J = 14.8, 6.1 Hz, 2H), 4.27 – 4.19 (m, 1H), 4.22 – 4.14 (m, 1H), 4.11 (t, J = 4.6 Hz, 2H), 3.95 – 3.84 (m, 3H), 3.71 (q, J = 4.8 Hz, 2H), 3.66 – 3.60 (m, 4H), 3.58 – 3.51 (m,

4H), 2.60 (d, *J* = 4.1 Hz, 1H), 2.50 (s, 3H), 2.43 (s, 3H), 2.30 (ddd, *J* = 13.0, 8.0, 4.9 Hz, 1H), 2.12 – 2.04 (m, 1H), 1.34 – 1.22 (m, 4H), 0.94 (s, 9H). ^{13}C NMR (126 MHz, CD_2Cl_2) δ 171.00, 170.95, 170.11 (d, *J* = 20.4 Hz), 157.27, 150.53, 149.05, 145.54, 133.27, 132.75, 132.01, 130.29, 129.95, 128.27, 127.47, 122.20, 113.33, 78.69 (d, *J* = 231.9 Hz), 71.09, 71.05, 70.89, 70.73, 70.67, 69.98, 69.90, 68.99, 68.55, 59.02, 57.70, 56.98, 39.37, 37.01, 35.80, 26.46, 21.78, 16.39, 13.88 (d, *J* = 10.0 Hz), 13.73 (d, *J* = 10.5 Hz). HRMS (ESI): m/z calcd for $C_{41}H_{56}FN_4O_{11}S_2$ $[M + H]^+$: 863.33708; found 863.33711.

Compound S15

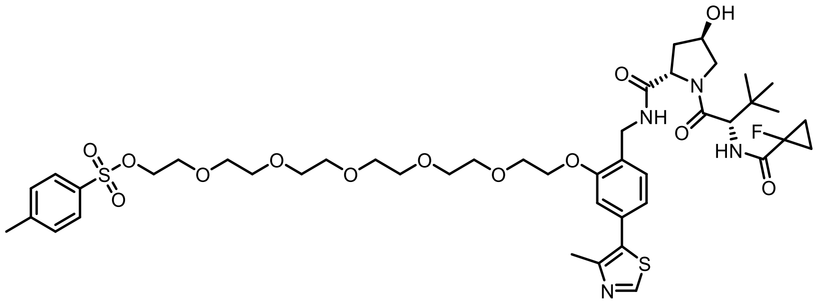

S15 (50 mg, 70%, white foam) was prepared from **VHL phenol**[28] (40 mg, 0.0751 mmol, 1.0 equiv) and ditosyl linker 3,6,9,12,15-pentaoxaheptadecane-1,17-diyl bis(4-methylbenzenesulfonate)[41] according to general procedure 3 and purified by flash column chromatography (2% to 6% MeOH in DCM). ^{1}H NMR (500 MHz, CD_2Cl_2) δ 8.58 (s, 1H), 7.69 (d, *J* = 8.0 Hz, 2H), 7.27 (dd, *J* = 16.3, 7.8 Hz, 3H), 7.15 (t, *J* = 6.2 Hz, 1H), 6.95 – 6.89 (m, 2H), 6.87 (s, 1H), 4.52 (t, *J* = 7.9 Hz, 1H), 4.47 (d, *J* = 8.9 Hz, 1H), 4.43 – 4.38 (m, 1H), 4.35 (dd, *J* = 12.9, 6.1 Hz, 2H), 4.15 (ddd, *J* = 9.4, 5.3, 3.5 Hz, 1H), 4.10 (ddd, *J* = 10.2, 6.1, 3.6 Hz, 1H), 4.05 – 4.01 (m, 2H), 3.86 – 3.77 (m, 3H), 3.67 – 3.62 (m, 2H), 3.59 – 3.53 (m, 5H), 3.53 – 3.43 (m, 11H), 2.64 (d, *J* = 4.1 Hz, 1H), 2.42 (s, 3H), 2.36 (s, 3H), 2.21 (ddt, *J* = 12.9, 8.0, 3.5 Hz, 1H), 2.04 – 1.98 (m, 1H), 1.25 – 1.13 (m, 4H), 0.86 (s, 9H). ^{13}C NMR (126 MHz, CD_2Cl_2) δ 171.05, 170.90, 170.02 (d, *J* = 20.2 Hz), 157.30, 150.52, 149.04, 145.51, 133.31, 132.75, 132.02, 130.29, 130.00, 128.27, 127.50, 122.20, 113.33, 78.69 (d, *J* = 232.4 Hz), 71.12, 71.02, 70.91, 70.87, 70.83, 70.81, 70.75, 70.64, 69.96, 69.90, 68.99, 68.54, 59.09, 57.68, 56.98, 39.40, 37.11, 35.89, 26.47, 22.74, 21.78, 16.40, 13.87 (d, *J* = 10.5 Hz), 13.71 (d, *J* = 10.5 Hz). HRMS (ESI): m/z calcd for $C_{45}H_{64}FN_4O_{13}S_2$ $[M + H]^+$: 951.38872; found 951.38870.

Synthesis of VHL thiol-tosyl intermediates

Compound S16

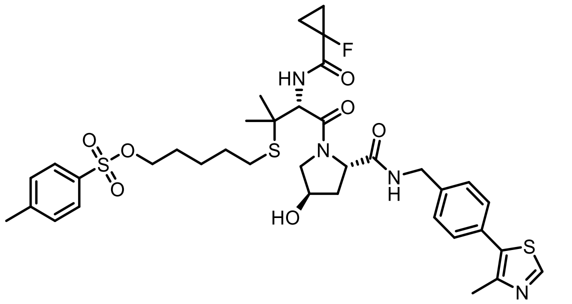

S16 (48 mg, 66%, white solid) was prepared from **VHL thiol**[29] (50 mg, 0.0938 mmol, 1.0 equiv) and pentane-1,5-diyl bis(4-methylbenzenesulfonate)[39] according to general procedure 4 and purified by flash column chromatography (1% to 4% MeOH in DCM). ^{1}H NMR (400 MHz, CD_2Cl_2) δ 8.66 (s, 1H), 7.77 – 7.71 (m, 2H), 7.43 – 7.27 (m, 7H), 7.20 (dd, *J* = 8.4, 3.5 Hz, 1H), 4.78 (dd, *J* = 8.3, 1.1 Hz, 1H), 4.68 (t, *J* = 8.1 Hz, 1H), 4.51 (p, *J* = 2.1 Hz, 1H), 4.42 (d, *J* = 6.2 Hz, 2H), 3.97 – 3.90 (m, 3H), 3.75 (dd, *J* = 11.1, 4.1 Hz, 1H), 2.86 (d, *J* = 4.6 Hz, 1H), 2.50 – 2.40 (m, 8H), 2.37 – 2.29 (m, 1H), 2.27 – 2.16 (m, 1H), 1.62 – 1.52 (m, 2H), 1.44 – 1.24 (m, 14H). ^{13}C NMR (126 MHz, CD_2Cl_2) δ 171.2, 170.4 (d, *J* = 20.4 Hz), 170.2, 150.5, 149.0, 145.4, 138.8, 133.4, 131.9, 131.3, 130.3, 129.7, 128.2, 128.2, 78.6 (d, *J* = 232.4 Hz), 70.8, 70.5, 59.6, 57.0, 56.3, 48.5, 43.1, 37.5, 29.2, 28.8, 28.3, 26.0, 25.3, 25.3, 21.8, 16.3, 14.1 (d, *J* = 7.5 Hz), 14.0 (d, *J* = 7.5 Hz). HRMS (ESI): m/z calcd for $C_{37}H_{48}N_4O_7S_3F$ $[M + H]^+$: 775.26635; found 775.26691.

Compound S17

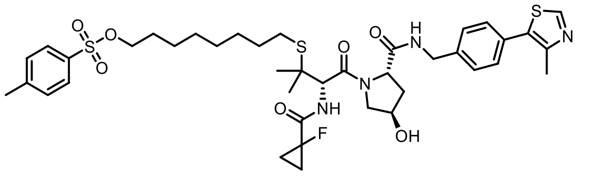

S17 (29 mg, 76%, white solid) was prepared from **VHL thiol**[29] (25 mg, 0.0468 mmol, 1.0 equiv) and octane-1,8-diyl bis(4-methylbenzenesulfonate)[36] according to general procedure 4 and purified by flash column chromatography (1% to 4% MeOH in DCM). ^{1}H NMR (500 MHz, CD_2Cl_2) δ 8.65 (s, 1H), 7.78 – 7.72 (m, 2H), 7.44 – 7.29 (m, 7H), 7.19 (d, *J* = 5.6 Hz, 1H), 4.79 (d, *J* = 8.1 Hz, 1H), 4.70 (t, *J* = 8.0 Hz, 1H), 4.50 (s, 1H), 4.43 (d, *J* = 6.3 Hz, 2H), 4.00 – 3.93 (m, 3H), 3.75 (dd, *J* = 11.1, 4.0 Hz, 1H), 2.65 (s, 1H), 2.55 – 2.39 (m, 8H), 2.39 – 2.30 (m, 1H), 2.27 – 2.19 (m, 1H), 1.64 – 1.55 (m, 4H), 1.45 – 1.36 (m, 2H), 1.36 – 1.12 (m, 16H). ^{13}C NMR (126 MHz, CD_2Cl_2) δ 171.1, 170.4 (d, *J* = 20.6 Hz), 170.2, 150.5, 148.9, 145.3, 138.8, 133.5, 131.9, 131.3, 130.2, 129.7, 128.2, 128.1, 78.6 (d, *J* = 232.4 Hz), 71.2, 70.5, 59.5, 57.0, 56.3, 48.3, 43.1, 37.6, 29.7, 29.4, 29.3, 29.1, 28.5, 26.1, 25.6, 25.1, 21.8, 16.3, 14.1 (d, *J* = 4.0 Hz), 14.0 (d, *J* = 4.5 Hz). HRMS (ESI): m/z calcd for $C_{40}H_{54}N_4O_7S_3F$ $[M + H]^+$: 817.31332; found 817.31328.

Compound S18

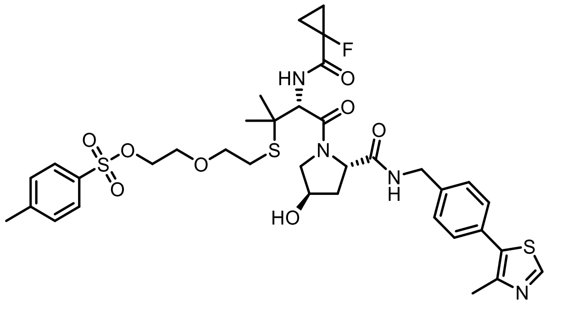

S18 (53 mg, 73%, white solid) was prepared from **VHL thiol**[29] (50 mg, 0.0935 mmol, 1.0 equiv) and oxybis(ethane-2,1-diyl) bis(4-methylbenzenesulfonate)[37]according to general procedure 4 and purified by flash column chromatography (1% to 5% EtOH in DCM). ^{1}H NMR (400 MHz, CD_2Cl_2) δ 8.66 (s, 1H), 7.75 (dq, *J* = 8.3, 2.0 Hz, 2H), 7.42 – 7.31 (m, 6H), 7.28 (t, *J* = 5.9 Hz, 1H), 7.19 (dd, *J* = 8.3, 3.0 Hz, 1H), 4.82 (dd, *J* = 8.4, 1.0 Hz, 1H), 4.67 (t, *J* = 8.1 Hz, 1H), 4.52 (s, 1H), 4.41 (d, *J* = 5.8 Hz, 2H), 4.04 (td, *J* = 4.1, 3.0 Hz, 2H), 3.92 (dt, *J* = 11.1, 2.0 Hz, 1H), 3.82 (dd, *J* = 11.0, 4.1 Hz, 1H), 3.54 (td, *J* = 4.1, 1.6 Hz, 2H), 3.51 – 3.39 (m, 2H), 2.84 (d, *J* = 4.3 Hz, 1H), 2.68 (h, *J* = 6.1 Hz, 2H), 2.48 (s, 3H), 2.43 (s, 3H), 2.32 (ddd, *J* = 12.7, 7.9, 4.7 Hz, 1H), 2.25 – 2.16 (m, 1H), 1.37 – 1.24 (m, 10H). ^{13}C NMR (126 MHz, CD_2Cl_2) δ 171.2, 170.4 (d, *J* = 20.4 Hz), 170.1, 150.5, 149.0, 145.7, 138.9, 133.2, 131.9, 131.3, 130.3, 129.7, 128.3, 128.2, 78.6 (d, *J* = 232.4 Hz), 70.7, 70.5, 69.6, 68.8, 59.7, 57.0, 56.3, 48.5, 43.2, 37.5, 28.6, 26.0, 25.4, 21.8, 14.06 (d, *J* = 9.5 Hz), 13.99 (d, *J* = 9.5 Hz). HRMS (ESI): m/z calcd for $C_{36}H_{46}N_4O_8S_3F$ [M + H]$^+$: 777.24563; found 777.24587.

Compound S19

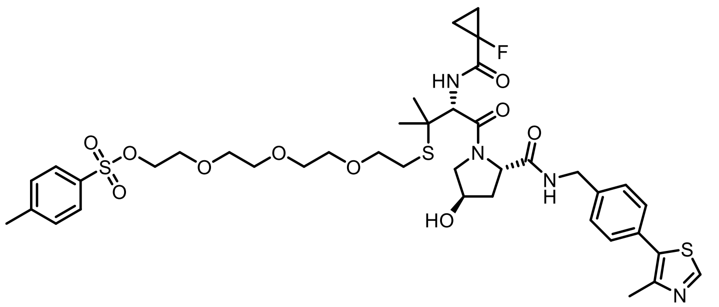

S19 (56 mg, 69%, white solid) was prepared from **VHL thiol**[29] (50 mg, 0.0935 mmol, 1.0 equiv) and ((oxybis(ethane-2,1-diyl))bis(oxy))bis(ethane-2,1-diyl) bis(4-methylbenzenesulfonate)[40] according to general procedure 4 and purified by flash column chromatography (2% to 7% EtOH in DCM). ^{1}H NMR (400 MHz, CD_2Cl_2) δ 8.66 (s, 1H), 7.81 – 7.74 (m, 2H), 7.42 – 7.29 (m, 7H), 7.18 (dd, *J* = 8.3, 3.2 Hz, 1H), 4.84 (dd, *J* = 8.5, 1.1 Hz, 1H), 4.67 (t, *J* = 7.9 Hz, 1H), 4.50 (q, *J* = 3.3 Hz, 1H), 4.50 – 4.35 (m, 2H), 4.14 – 4.08 (m, 2H), 3.93 – 3.88 (m, 1H), 3.84 (dd, *J* = 11.1, 4.3 Hz, 1H), 3.66 – 3.60 (m, 2H), 3.59 – 3.42 (m, 10H), 2.82 (d, *J* = 4.7 Hz, 1H), 2.83 – 2.66 (m, 2H), 2.49 (s, 3H), 2.44 (s, 3H), 2.35 – 2.27 (m, 1H), 2.24 – 2.16 (m, 1H), 1.36 – 1.23 (m, 10H). ^{13}C NMR (126 MHz, CD_2Cl_2) δ 171.2, 170.3 (d, *J* = 20.4 Hz), 170.1, 150.5, 149.0, 145.5, 138.9, 133.3, 131.9, 131.3, 130.3, 129.7, 128.3, 128.3, 78.6 (d, *J* = 232.4 Hz), 71.0, 70.8, 70.7, 70.7, 70.5, 70.4, 69.9, 69.0, 59.6, 56.9, 56.2, 48.4, 43.1, 37.5, 28.9, 26.1, 25.3, 21.8, 16.3, 14.1 (d, *J* = 9.0 Hz), 14.0 (d,

J = 8.5 Hz). HRMS (ESI): m/z calcd for $C_{40}H_{54}N_4O_{10}S_3F$ [M + H]$^+$: 865.29806; found 865.29810.

Compound S20

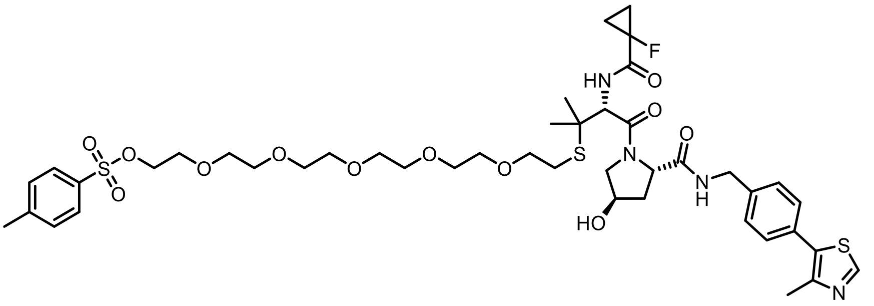

S20 (61 mg, 68%, yellow solid) was prepared from **VHL thiol**[29] (50 mg, 0.0935 mmol, 1.0 equiv) and 3,6,9,12,15-pentaoxaheptadecane-1,17-diyl bis(4-methylbenzenesulfonate)[41] according to general procedure 4 and purified by flash column chromatography (2% to 6% MeOH in DCM). ^{1}H NMR (500 MHz, CD_2Cl_2) δ 8.66 (s, 1H), 7.81 – 7.75 (m, 2H), 7.44 – 7.32 (m, 7H), 7.20 – 7.15 (m, 1H), 4.86 (dd, J = 8.5, 1.2 Hz, 1H), 4.67 (t, J = 7.9 Hz, 1H), 4.52 (q, J = 4.1 Hz, 1H), 4.42 (qd, J = 15.2, 6.1 Hz, 2H), 4.15 – 4.10 (m, 2H), 3.88 (d, J = 3.5 Hz, 2H), 3.66 – 3.63 (m, 2H), 3.61 – 3.48 (m, 18H), 2.94 (d, J = 4.3 Hz, 1H), 2.82 – 2.70 (m, 2H), 2.49 (s, 3H), 2.44 (s, 3H), 2.32 (ddd, J = 13.4, 7.4, 4.8 Hz, 1H), 2.20 (ddd, J = 13.4, 8.3, 3.4 Hz, 1H), 1.35 – 1.26 (m, 10H). ^{13}C NMR (126 MHz, CD_2Cl_2) δ 171.30, 170.2 (d, J = 20.5 Hz), 170.14, 150.5, 149.0, 145.5, 138.9, 133.3, 131.9, 131.3, 130.3, 129.7, 128.4, 128.3, 78.6 (d, J = 231.9 Hz), 71.0, 70.9, 70.9, 70.9, 70.9, 70.8, 70.8, 70.7, 70.4, 70.2, 69.9, 69.0, 59.7, 56.8, 56.1, 48.4, 43.1, 37.6, 28.9, 26.1, 25.1, 21.8, 14.04 (d, J = 10.5 Hz), 13.96 (d, J = 10.5 Hz). HRMS (ESI): m/z calcd for $C_{44}H_{62}N_4O_{12}S_3F$ [M + H]$^+$: 953.35049; found 953.35046.

Compound S21

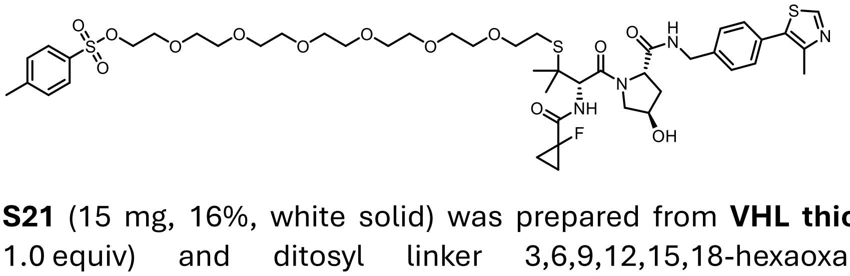

S21 (15 mg, 16%, white solid) was prepared from **VHL thiol**[29] (50 mg, 0.0935 mmol, 1.0 equiv) and ditosyl linker 3,6,9,12,15,18-hexaoxaicosane-1,20-diyl bis(4-methylbenzenesulfonate)[41] according to general procedure 4 and purified by flash column chromatography (1% to 7% MeOH in DCM). ^{1}H NMR (400 MHz, CD_2Cl_2) δ 8.66 (s, 1H), 7.78 (d, J = 8.4 Hz, 2H), 7.42 – 7.32 (m, 7H), 7.16 (dd, J = 8.7, 3.4 Hz, 1H), 4.87 (d, J = 7.2 Hz, 1H), 4.66 (t, J = 7.8 Hz, 1H), 4.52 (t, J = 4.0 Hz, 1H), 4.42 (qd, J = 15.1, 6.0 Hz, 2H), 4.16 – 4.08 (m, 2H), 3.88 (d, J = 3.7 Hz, 2H), 3.67 – 3.62 (m, 2H), 3.62 – 3.42 (m, 22H), 2.77 (ddt, J = 16.9, 12.4, 6.1 Hz, 2H), 2.49 (s, 3H), 2.44 (s, 3H), 2.32 (ddd, J = 13.3, 7.4, 4.9 Hz, 1H), 2.20 (ddd, J = 12.9, 8.3, 3.4 Hz, 1H), 1.37 – 1.23 (m, 10H). ^{13}C NMR (126 MHz, CD_2Cl_2) δ 171.30, 170.23 (d, J = 20.9 Hz), 170.16, 150.48, 148.98, 145.50, 138.90, 133.33, 131.92, 131.31, 130.28, 129.74, 128.37, 128.28, 78.63 (d, J = 232.4 Hz), 71.04, 70.93, 70.91,

70.90, 70.88, 70.85, 70.79, 70.75, 70.68, 70.35, 70.21, 69.91, 68.99, 59.75, 56.83, 56.10, 48.34, 43.11, 37.61, 28.97, 26.19, 25.05, 21.78, 16.34, 14.05 (d, *J* = 10.5 Hz), 13.96 (d, *J* = 10.5 Hz). HRMS (ESI): m/z calcd for $C_{46}H_{65}FN_4O_{13}S_3$ $[M + H]^+$: 997.37589; found 997.37589.

Synthesis of 'pyrazole-VHL' PROTACs

Compound 6

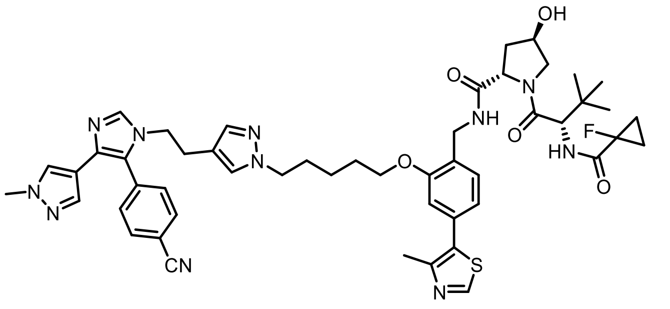

6 (10.5 mg, 48%, white solid) was prepared from **BAZ2 'pyrazole' warhead** (8 mg, 0.0233 mmol, 1.0 equiv) and VHL phenol-tosyl intermediate **S11** according to general procedure 7 and purified by preparative TLC (9% MeOH in DCM) followed by trituration with Et_2O. ^{1}H NMR (500 MHz, CD_2Cl_2) δ 8.66 (s, 1H), 7.77 (d, *J* = 7.8 Hz, 2H), 7.44 – 7.38 (m, 3H), 7.37 (s, 1H), 7.35 – 7.30 (m, 2H), 7.24 (s, 1H), 7.04 (s, 1H), 7.03 – 6.99 (m, 1H), 6.97 (d, *J* = 7.7 Hz, 1H), 6.94 (s, 1H), 6.89 (s, 1H), 4.63 (t, *J* = 7.6 Hz, 1H), 4.57 (d, *J* = 8.9 Hz, 1H), 4.54 – 4.50 (m, 1H), 4.48 (d, *J* = 6.4 Hz, 1H), 4.45 (d, *J* = 6.6 Hz, 1H), 4.35 (dd, *J* = 14.7, 5.3 Hz, 1H), 4.05 (t, *J* = 7.0 Hz, 2H), 4.03 – 3.97 (m, 2H), 3.94 (t, *J* = 6.7 Hz, 2H), 3.85 (d, *J* = 10.9 Hz, 1H), 3.77 (s, 3H), 3.65 (dd, *J* = 11.0, 4.3 Hz, 1H), 2.63 (t, *J* = 6.9 Hz, 2H), 2.50 (s, 3H), 2.40 (dt, *J* = 12.8, 6.0 Hz, 2H), 2.06 – 1.98 (m, 2H), 1.91 – 1.78 (m, 4H), 1.36 – 1.17 (m, 4H), 0.93 (s, 9H). ^{13}C NMR (126 MHz, CD_2Cl_2) δ 170.83, 170.43, 169.56 (d, *J* = 20.4 Hz), 156.87, 150.08, 148.60, 138.27, 137.80, 136.44, 135.37, 132.82, 132.38, 131.69, 131.30, 129.63, 127.78, 127.31, 126.42, 124.87, 121.31, 118.42, 116.37, 115.98, 112.39, 112.07, 78.31 (d, *J* = 231.9 Hz), 70.02, 67.87, 58.68, 57.24, 56.34, 51.90, 46.65, 38.79, 38.73, 36.42, 35.42, 30.22, 28.66, 26.05, 25.67, 23.45, 15.98, 13.46 (d, *J* = 10.5 Hz), 13.27 (d, *J* = 10.5 Hz). ^{19}F NMR (471 MHz, CD2Cl2) δ -197.95. HRMS (ESI): m/z calcd for $C_{50}H_{59}FN_{11}O_5S$ $[M + H]^+$: 944.43892; found 944.43894.

Compound 7

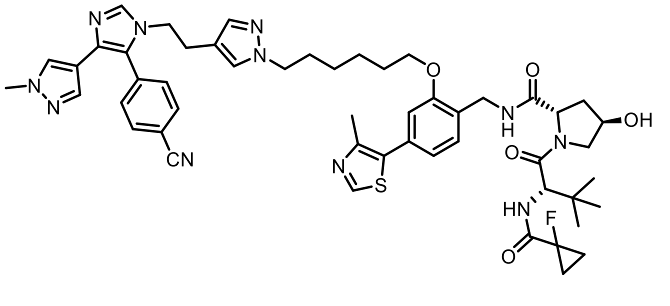

7 (3.74 mg, 19%, white solid) was prepared from BAZ2 'pyrazole' warhead-tosyl intermediate **S1** (12.6 mg, 0.0211 mmol, 1.0 equiv) and **VHL phenol**[28] according to general procedure 5 and purified by preparative TLC (2:2:1 EtOAc: DCM:MeOH) followed by trituration with Et_2O and pentane. ^{1}H NMR (500 MHz, CD_2Cl_2) δ 8.66 (s, 1H), 7.77 (d, *J* = 7.9 Hz, 2H), 7.41 (d, *J* = 7.9 Hz, 2H), 7.37 (s, 2H), 7.33 (d, *J* = 7.8 Hz, 1H), 7.30 (t, *J* = 5.9 Hz, 1H), 7.23 (s, 1H), 7.03 – 6.93 (m, 4H), 6.89 (s, 1H), 4.62 (t, *J* = 7.7 Hz, 1H), 4.57 (d, *J* = 8.9 Hz, 1H), 4.54 – 4.49 (m, 1H), 4.47 (d, *J* = 6.5 Hz, 1H), 4.37 (dd, *J* = 14.6, 5.1 Hz, 1H), 4.05 –

3.97 (m, 3H), 3.97 – 3.91 (m, 2H), 3.86 (d, *J* = 11.1 Hz, 1H), 3.77 (s, 3H), 3.65 (dd, *J* = 11.0, 4.2 Hz, 1H), 2.65 – 2.57 (m, 2H), 2.50 (s, 3H), 2.40 (dt, *J* = 12.9, 6.4 Hz, 1H), 2.06 – 1.98 (m, 1H), 1.81 (p, *J* = 6.5 Hz, 4H), 1.36 – 1.22 (m, 8H), 0.93 (s, 9H). ^{13}C NMR (126 MHz, CD_2Cl_2) δ 171.20, 170.85, 169.97 (d, *J* = 20.4 Hz), 157.34, 150.50, 148.99, 138.65, 138.55, 138.20, 136.84, 135.76, 133.24, 132.78, 132.15, 131.71, 130.07, 128.22, 127.74, 126.83, 125.26, 121.68, 118.82, 116.73, 116.29, 112.81, 112.47, 78.72 (d, *J* = 232.4 Hz), 70.39, 68.29, 59.12, 57.62, 56.79, 52.27, 47.08, 39.14, 36.92, 35.88, 30.78, 29.47, 29.42, 26.54, 26.45, 26.12, 26.06, 16.38, 13.87 (d, *J* = 10.0 Hz), 13.69 (d, *J* = 10.0 Hz). ^{19}F NMR (377 MHz, CD_2Cl_2) δ -197.95. HRMS (ESI): m/z calcd for $C_{51}H_{61}FN_{11}O_5S$ $[M + H]^+$: 958.45626; found 958.45614.

Compound 8

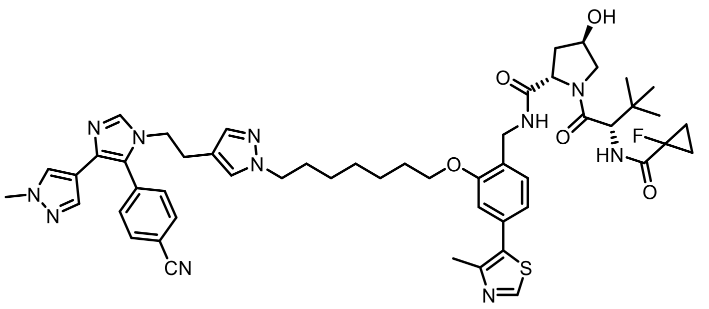

8 (5 mg, 24%, white solid) was prepared from BAZ2 'pyrazole' warhead-tosyl intermediate **S2** (13 mg, 0.0212 mmol, 1.0 equiv) and **VHL phenol**[28] (13.6 mg, 0.0255 mmol, 1.2 equiv) according to general procedure 5 and purified by preparative TLC (2:2:1 EtOAc:DCM:MeOH) followed by trituration with Et2O and pentane. ^{1}H NMR (500 MHz, CD_2Cl_2) δ 8.66 (s, 1H), 7.77 (d, *J* = 8.2 Hz, 2H), 7.45 – 7.38 (m, 3H), 7.36 (s, 1H), 7.33 (d, *J* = 7.7 Hz, 1H), 7.29 – 7.23 (m, 2H), 7.03 – 6.99 (m, 2H), 6.96 (d, *J* = 7.7 Hz, 1H), 6.91 (d, *J* = 8.0 Hz, 2H), 4.63 (t, *J* = 7.6 Hz, 1H), 4.57 (d, *J* = 8.9 Hz, 1H), 4.55 – 4.45 (m, 2H), 4.36 (dd, *J* = 14.8, 5.2 Hz, 1H), 4.07 – 3.95 (m, 4H), 3.94 (t, *J* = 6.8 Hz, 2H), 3.86 (d, *J* = 11.0 Hz, 1H), 3.77 (s, 3H), 3.65 (dd, *J* = 11.0, 4.2 Hz, 1H), 2.63 (t, *J* = 6.8 Hz, 2H), 2.50 (s, 3H), 2.41 (ddd, *J* = 12.6, 7.4, 4.9 Hz, 1H), 2.07 – 1.98 (m, 1H), 1.85 – 1.71 (m, 4H), 1.54 – 1.44 (m, 2H), 1.45 – 1.33 (m, 2H), 1.34 – 1.18 (m, 6H), 0.93 (s, 9H). ^{13}C NMR (126 MHz, CD_2Cl_2) δ 171.22, 170.85, 170.00 (d, *J* = 20.2 Hz), 157.34, 150.48, 148.99, 138.54, 138.23, 136.86, 135.83, 133.38, 133.22, 132.73, 132.16, 131.70, 129.89, 128.13, 127.68, 126.83, 125.30, 121.63, 118.84, 116.87, 116.34, 112.77, 112.49, 78.72 (d, *J* = 232.2 Hz), 70.44, 68.50, 59.09, 57.62, 56.82, 52.48, 47.02, 39.20, 39.14, 36.81, 35.86, 30.78, 29.46, 29.17, 26.89, 26.44, 26.13, 16.38, 13.88 (d, *J* = 10.3 Hz), 13.70 (d, *J* = 10.2 Hz). ^{19}F NMR (471 MHz, CD_2Cl_2) δ -197.95. HRMS (ESI): m/z calcd for $C_{52}H_{63}FN_{11}O_5S$ $[M + H]^+$: 972.47068; found 972.47067.

Compound 9

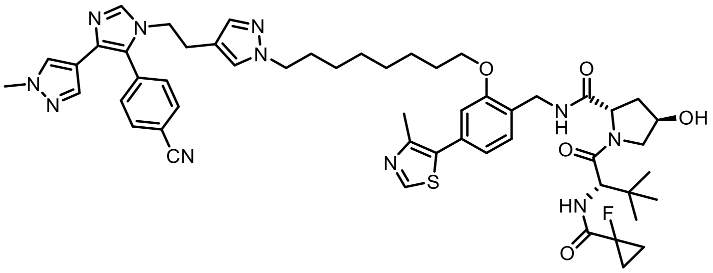

9 (20 mg, 87%, white solid) was prepared from **BAZ2 'pyrazole' warhead** (8 mg, 0.0233 mmol, 1.0 equiv) and VHL phenol-tosyl intermediate **S12** according to general procedure 7 and purified by flash column chromatography (2% to 12% MeOH in DCM). ^{1}H NMR (500 MHz, CD_2Cl_2) δ 8.66 (s, 1H), 7.79 – 7.73 (m, 2H), 7.43 – 7.39 (m, 2H), 7.38 (s, 1H), 7.37 (s, 1H), 7.36 – 7.31 (m, 2H), 7.23 (s, 1H), 7.04 – 6.99 (m, 2H), 6.95 (dd, *J* = 7.7, 1.8 Hz, 1H), 6.92 (s, 1H), 6.90 (d, *J* = 1.9 Hz, 1H), 4.64 (t, *J* = 7.7 Hz, 1H), 4.58 (d, *J* = 9.2 Hz, 1H), 4.54 – 4.51 (m, 1H), 4.48 (dd, *J* = 14.8, 6.5 Hz, 1H), 4.38 (dd, *J* = 14.8, 5.2 Hz, 1H), 4.05 – 3.89 (m, 7H), 3.85 (dt, *J* = 11.1, 2.1 Hz, 1H), 3.78 (s, 3H), 3.64 (dd, *J* = 11.0, 4.2 Hz, 1H), 2.67 – 2.56 (m, 2H), 2.49 (s, 3H), 2.41 (ddd, *J* = 12.6, 7.5, 4.7 Hz, 1H), 2.07 – 2.01 (m, 1H), 1.86 – 1.79 (m, 2H), 1.75 (p, *J* = 7.5 Hz, 2H), 1.49 (p, *J* = 7.2 Hz, 2H), 1.40 – 1.19 (m, 10H), 0.93 (s, 9H). ^{13}C NMR (126 MHz, CD_2Cl_2) δ 171.16, 170.93, 169.89 (d, *J* = 20.4 Hz), 157.35, 150.46, 148.97, 138.59, 138.21, 136.85, 135.83, 133.43, 133.22, 132.68, 132.17, 131.69, 129.76, 128.12, 127.73, 126.79, 125.24, 121.61, 118.83, 116.84, 116.26, 112.75, 112.44, 78.71 (d, *J* = 231.9 Hz), 70.38, 68.54, 59.16, 57.60, 56.92, 52.46, 47.07, 39.21, 39.14, 36.92, 35.99, 30.86, 29.49, 29.42, 29.35, 26.70, 26.47, 26.45, 26.10, 16.38, 13.87 (d, *J* = 10.5 Hz), 13.67 (d, *J* = 10.0 Hz). HRMS (ESI): m/z calcd for $C_{53}H_{65}N_{11}O_5SF$ $[M + H]^+$: 986.48694; found 986.48695.

Compound 10

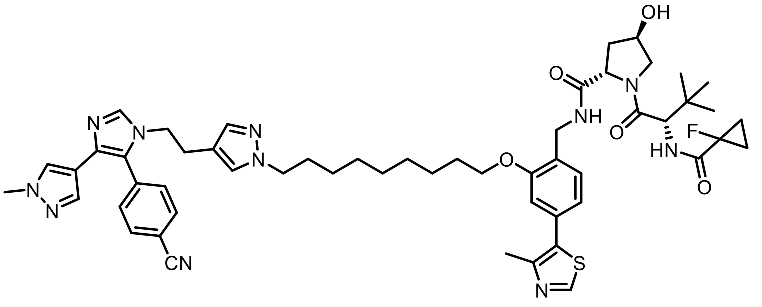

10 (4.24 mg, 21%, white solid) was prepared from BAZ2 'pyrazole' warhead-tosyl intermediate **S3** (12.8 mg, 0.0200 mmol, 1.0 equiv) and **VHL phenol**[28] according to general procedure 5 and purified by preparative TLC (2:2:1 EtOAc:DCM:MeOH) followed by trituration with Et_2O and pentane. ^{1}H NMR (500 MHz, CD_2Cl_2) δ 8.70 (d, *J* = 1.4 Hz, 1H), 7.81 (d, *J* = 8.2 Hz, 2H), 7.48 – 7.42 (m, 3H), 7.40 – 7.35 (m, 2H), 7.29 (s, 1H), 7.26 (t, *J* = 6.3 Hz, 1H), 7.08 – 7.02 (m, 2H), 7.00 (d, *J* = 7.7 Hz, 1H), 6.96 (d, *J* = 7.6 Hz, 2H), 4.68 (t, *J* = 7.7 Hz, 1H), 4.61 (d, *J* = 8.9 Hz, 1H), 4.58 – 4.53 (m, 2H), 4.52 (d, *J* = 6.6 Hz, 1H), 4.41 (dd, *J* = 14.9, 5.2 Hz, 1H), 4.10 – 4.04 (m, 2H), 3.99 (dt, *J* = 13.5, 7.1 Hz, 4H), 3.91 (d, *J* = 11.1 Hz, 1H), 3.82 (s, 3H), 3.67 (dd, *J* = 11.0, 4.2 Hz, 1H), 2.68 (t, *J* = 6.9 Hz, 2H), 2.54 (s, 3H), 2.52

– 2.43 (m, 1H), 2.11 – 2.03 (m, 1H), 1.87 (p, *J* = 6.6 Hz, 2H), 1.78 (p, *J* = 7.4 Hz, 2H), 1.53 (q, *J* = 6.9 Hz, 2H), 1.42 – 1.25 (m, 10H), 0.96 (s, 9H). ^{13}C NMR (126 MHz, CD_2Cl_2) δ 171.21, 170.86, 170.03 (d, *J* = 20.4 Hz), 157.35, 150.47, 148.97, 138.55, 138.16, 136.87, 135.84, 133.38, 133.21, 132.68, 132.18, 131.70, 129.64, 128.09, 127.64, 126.83, 125.30, 121.61, 118.84, 116.90, 116.27, 112.75, 112.47, 78.70 (d, *J* = 232.4 Hz), 70.42, 68.62, 59.07, 57.62, 56.89, 52.52, 47.03, 39.15, 36.70, 35.86, 30.81, 29.70, 29.60, 29.57, 29.30, 26.92, 26.54, 26.44, 26.19, 16.38, 13.88 (d, *J* = 10.0 Hz), 13.72 (d, *J* = 10.5 Hz). 1 signal belonging to aliphatic carbon is not listed as it overlaps with the solvent peak. ^{19}F NMR (377 MHz, CD_2Cl_2) δ -197.97. HRMS (ESI): m/z calcd for $C_{54}H_{67}FN_{11}O_5S$ [M + H]$^+$: 1000.50195; found 1000.50195.

Compound 11

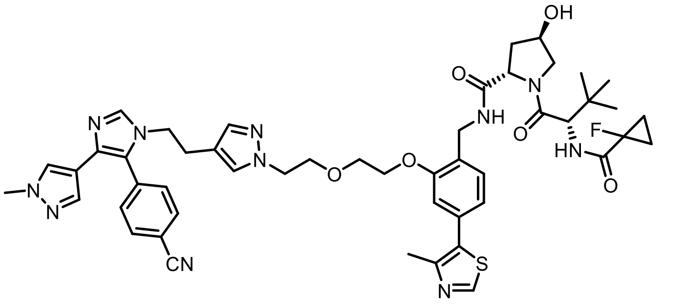

11 (12 mg, 54 %, white solid) was prepared from **BAZ2 'pyrazole' warhead** (8 mg, 0.0233 mmol, 1.0 equiv) and VHL phenol-tosyl intermediate **S13** according to general procedure 7 and purified by preparative TLC (7% MeOH in DCM) followed by trituration with Et_2O and pentane. ^{1}H NMR (500 MHz, CD_2Cl_2) δ 8.66 (s, 1H), 7.75 (d, *J* = 8.7 Hz, 2H), 7.55 (t, *J* = 6.0 Hz, 1H), 7.40 (d, *J* = 8.6 Hz, 2H), 7.35 (d, *J* = 8.6 Hz, 3H), 7.23 (s, 1H), 7.04 – 6.97 (m, 4H), 6.88 (s, 1H), 4.61 – 4.53 (m, 2H), 4.50 (s, 1H), 4.42 (t, *J* = 5.7 Hz, 2H), 4.28 – 4.16 (m, 2H), 4.15 – 4.09 (m, 1H), 4.08 – 4.02 (m, 1H), 3.97 – 3.80 (m, 7H), 3.76 (s, 3H), 3.67 (dd, *J* = 11.0, 4.0 Hz, 1H), 2.66 – 2.52 (m, 2H), 2.49 (s, 3H), 2.30 – 2.21 (m, 1H), 2.08 – 2.01 (m, 1H), 1.33 – 1.20 (m, 4H), 0.97 (s, 9H). ^{13}C NMR (126 MHz, CD_2Cl_2) δ 170.77, 170.52, 169.40 (d, *J* = 20.4 Hz), 156.75, 150.14, 148.66, 138.61, 137.89, 136.40, 135.33, 132.88, 132.81, 132.37, 131.54, 131.33, 129.77, 128.79, 127.27, 126.91, 124.84, 121.80, 118.41, 116.39, 116.14, 112.53, 112.40, 78.32 (d, *J* = 232.4 Hz), 70.09, 69.94, 69.47, 68.17, 58.91, 57.24, 56.58, 51.94, 46.58, 38.86, 38.73, 37.02, 35.64, 26.09, 25.60, 15.99, 13.45 (d, *J* = 10.5 Hz), 13.20 (d, *J* = 10.5 Hz). ^{19}F NMR (471 MHz, CD_2Cl_2) δ -197.90. HRMS (ESI): m/z calcd for $C_{49}H_{57}FN_{11}O_6S$ [M + H]$^+$: 946.41831; found 946.41838.

Compound 12

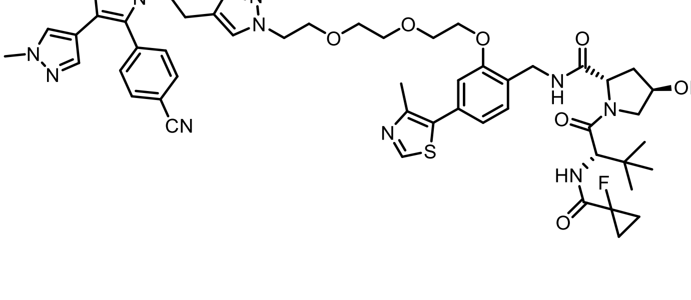

12 (6 mg, 32%, white solid) was prepared from BAZ2 'pyrazole' warhead-tosyl intermediate **S5** (12 mg, 0.0191 mmol, 1.0 equiv) and **VHL phenol**[28] according to general procedure 5 and purified by preparative TLC (10% MeOH in DCM) followed by trituration with Et_2O and pentane. ^{1}H NMR (400 MHz, CD_2Cl_2) δ 8.66 (s, 1H), 7.79 – 7.74 (m, 2H), 7.59 (t, *J* = 6.0 Hz, 1H), 7.42 – 7.37 (m, 3H), 7.36 – 7.33 (m, 2H), 7.22 (s, 1H), 7.06 – 7.02 (m, 2H), 7.01 – 6.96 (m, 2H), 6.94 – 6.89 (m, 1H), 4.62 – 4.54 (m, 2H), 4.49 (s, 1H), 4.42 (d, *J* = 6.0 Hz, 2H), 4.24 – 4.08 (m, 5H), 3.97 – 3.87 (m, 2H), 3.84 – 3.79 (m, 2H), 3.77 (s, 3H), 3.76 – 3.70 (m, 2H), 3.66 (dd, *J* = 11.0, 3.9 Hz, 1H), 3.63 – 3.58 (m, 2H), 3.56 – 3.51 (m, 2H), 2.68 – 2.52 (m, 2H), 2.49 (s, 3H), 2.19 (ddd, *J* = 12.9, 8.3, 4.5 Hz, 1H), 2.11 – 2.04 (m, 1H), 1.31 – 1.22 (m, 4H), 0.97 (s, 9H). ^{13}C NMR (126 MHz, CD_2Cl_2) δ 171.38, 170.78, 169.70 (d, *J* = 19.9 Hz), 157.27, 150.52, 149.05, 138.98, 138.29, 136.82, 135.72, 133.23, 132.70, 131.99, 131.76, 130.15, 129.31, 127.74, 127.66, 125.26, 122.26, 118.82, 116.73, 116.43, 113.38, 112.83, 78.72 (d, *J* = 232.4 Hz), 71.00, 70.78, 70.48, 70.14, 69.95, 68.52, 59.32, 57.61, 57.15, 52.37, 47.03, 39.39, 39.15, 37.64, 36.20, 30.09, 26.50, 16.41, 13.85 (d, *J* = 10.0 Hz), 13.57 (d, *J* = 10.5 Hz). ^{19}F NMR (377 MHz, CD_2Cl_2) δ -197.88. HRMS (ESI): m/z calcd for $C_{51}H_{61}FN_{11}O_7S$ [M + H]$^+$: 990.44500; found 990.44500.

Compound 13

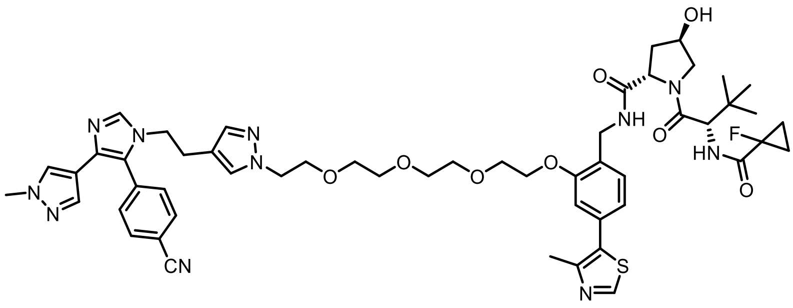

13 (13 mg, 54%, white solid) was prepared from **BAZ2 'pyrazole' warhead** (8 mg, 0.0233 mmol, 1.0 equiv) and VHL phenol-tosyl intermediate **S14** according to general procedure 7 and purified by preparative TLC (10% MeOH in DCM) followed by trituration with Et_2O and pentane. ^{1}H NMR (500 MHz, CD_2Cl_2) δ 8.70 (s, 1H), 7.80 (d, *J* = 8.5 Hz, 2H), 7.51 (t, *J* = 6.1 Hz, 1H), 7.48 (s, 1H), 7.44 (d, *J* = 7.8 Hz, 2H), 7.41 – 7.37 (m, 2H), 7.27 (s, 1H), 7.09 – 7.01 (m, 4H), 6.98 (s, 1H), 4.62 (t, *J* = 9.1 Hz, 2H), 4.55 – 4.50 (m, 2H), 4.47 (t, *J* = 6.1 Hz, 2H), 4.29 – 4.23 (m, 1H), 4.23 – 4.14 (m, 2H), 4.01 – 3.96 (m, 2H), 3.94 – 3.86 (m, 2H), 3.81 (s, 3H), 3.78 – 3.66 (m, 5H), 3.64 (dt, *J* = 9.9, 5.0 Hz, 2H), 3.57 – 3.47 (m, 4H), 2.66 (q, *J* = 7.1 Hz, 2H), 2.54 (s, 3H), 2.30 – 2.22 (m, 1H), 2.18 – 2.09 (m, 1H), 1.39 – 1.20 (m, 4H), 1.00 (s, 9H). ^{13}C NMR (126 MHz, CD_2Cl_2) δ 170.95, 170.37, 169.36 (d, *J* = 19.9 Hz), 156.85, 150.10, 148.64, 138.51, 137.89, 136.44, 135.34, 132.83, 132.82, 132.29, 131.59, 131.31, 129.57, 128.75, 127.30, 127.24, 124.86, 121.85, 118.42, 116.33, 116.01, 112.95, 112.40, 78.29 (d, *J* = 232.4 Hz), 70.80, 70.46, 70.37, 70.35, 70.00, 69.72, 69.61, 68.16, 58.91, 57.17, 56.74, 52.06, 46.60, 38.96, 38.74, 37.09, 35.71, 26.07, 25.66, 15.99, 13.44 (d, *J* = 10.3 Hz), 13.21 (d, *J* = 10.4 Hz). ^{19}F NMR (471 MHz, CD_2Cl_2) δ -197.90. HRMS (ESI): m/z calcd for $C_{53}H_{65}FN_{11}O_8S$ [M + H]$^+$: 1034.47048; found 1034.47042.

Compound 14

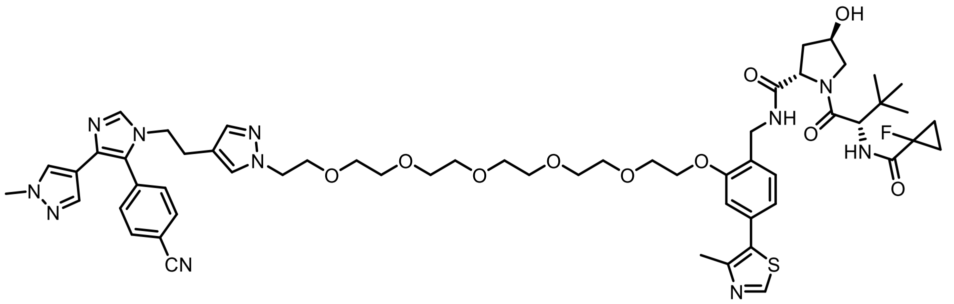

14 (14 mg, 54%, white solid) was prepared from **BAZ2 'pyrazole' warhead** (8 mg, 0.0233 mmol, 1.0 equiv) and VHL phenol-tosyl intermediate **S15** according to general procedure 7 and purified by preparative TLC (10% MeOH in DCM) followed by trituration with Et_2O and pentane. ^1H NMR (500 MHz, CD_2Cl_2) δ 8.65 (s, 1H), 7.77 (d, *J* = 8.6 Hz, 2H), 7.47 (s, 2H), 7.41 (d, *J* = 7.9 Hz, 2H), 7.39 – 7.34 (m, 2H), 7.23 (s, 1H), 7.10 (s, 1H), 7.06 – 7.01 (m, 1H), 7.01 (s, 1H), 6.99 (d, *J* = 7.7 Hz, 1H), 6.94 (s, 1H), 4.63 – 4.57 (m, 2H), 4.53 – 4.47 (m, 1H), 4.45 (d, *J* = 6.4 Hz, 1H), 4.40 (dd, *J* = 15.0, 5.7 Hz, 2H), 4.25 – 4.13 (m, 4H), 3.95 (t, *J* = 7.0 Hz, 2H), 3.92 – 3.81 (m, 3H), 3.78 (s, 3H), 3.76 – 3.69 (m, 4H), 3.68 – 3.61 (m, 3H), 3.60 – 3.47 (m, 11H), 2.65 (td, *J* = 7.1, 3.9 Hz, 2H), 2.50 (s, 3H), 2.26 (ddd, *J* = 13.0, 8.2, 4.7 Hz, 1H), 2.14 – 2.05 (m, 1H), 1.35 – 1.20 (m, 4H), 0.96 (s, 9H). ^{13}C NMR (126 MHz, CD_2Cl_2) δ 170.92, 170.41, 169.40 (d, *J* = 20.4 Hz), 156.81, 150.09, 148.62, 138.46, 137.78, 136.42, 135.42, 132.91, 132.82, 132.23, 131.63, 131.31, 129.47, 128.77, 127.31, 127.18, 124.88, 121.78, 118.44, 116.46, 116.06, 112.84, 112.34, 78.29 (d, *J* = 232.4 Hz), 70.75, 70.48, 70.46, 70.44, 70.42, 70.33, 70.05, 69.72, 69.57, 68.15, 65.66, 58.87, 57.19, 56.66, 52.03, 46.57, 38.88, 38.72, 36.97, 35.71, 26.07, 25.83, 15.99, 15.09, 13.45 (d, *J* = 10.0 Hz), 13.24 (d, *J* = 10.0 Hz). ^{19}F NMR (377 MHz, CD_2Cl_2) δ -197.91. HRMS (ESI): m/z calcd for $C_{57}H_{73}FN_{11}O_{10}S$ $[M + H]^+$: 1122.52293; found 1122.52280.

Compound 15

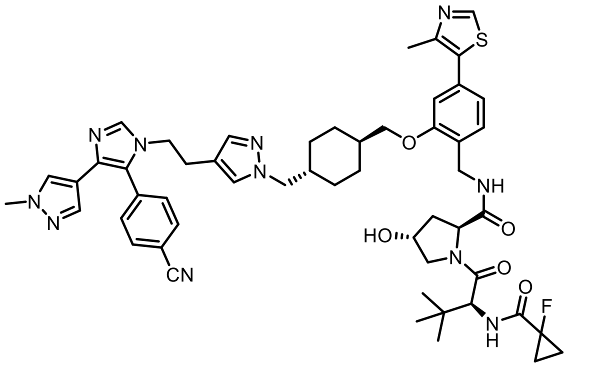

15 (12 mg, 51%, white solid) was prepared from BAZ2 'pyrazole' warhead-tosyl intermediate **S6** (15 mg, 0.0240 mmol, 1.0 equiv) and **VHL phenol**[28] according to general procedure 5 and purified by preparative TLC (1:7:7 MeOH:DCM:EtOAC) followed by trituration with Et_2O. ^1H NMR (500 MHz, CD_2Cl_2) δ 8.66 (s, 1H), 7.81 – 7.74 (m, 2H), 7.46 – 7.40 (m, 2H), 7.41 (s, 1H), 7.36 – 7.30 (m, 2H), 7.26 (d, *J* = 0.8 Hz, 1H), 7.13 (t, *J* = 6.0 Hz, 1H), 7.04 (d, *J* = 0.8 Hz, 1H), 7.01 (dd, *J* = 8.9, 2.8 Hz, 1H), 6.96 (dd, *J* = 7.6, 1.7 Hz, 1H), 6.92 (s, 1H), 6.88 (d, *J* = 1.7 Hz, 1H), 4.65 (t, *J* = 7.6 Hz, 1H), 4.56 (dd, *J* = 8.9, 1.3 Hz, 1H),

4.53 – 4.46 (m, 2H), 4.34 (dd, J = 15.0, 5.0 Hz, 1H), 3.96 (td, J = 6.8, 2.2 Hz, 2H), 3.88 – 3.78 (m, 5H), 3.76 (s, 3H), 3.64 (dd, J = 11.0, 4.3 Hz, 1H), 2.64 (t, J = 6.8 Hz, 2H), 2.50 (s, 3H), 2.43 (ddd, J = 13.4, 7.2, 4.9 Hz, 1H), 2.08 – 1.99 (m, 1H), 1.89 (t, J = 10.8 Hz, 2H), 1.85 – 1.75 (m, 2H), 1.62 (d, J = 12.4 Hz, 2H), 1.33 – 1.21 (m, 4H), 1.19 – 1.08 (m, 2H), 1.07 – 0.96 (m, 2H), 0.91 (s, 9H). (-O*H* not detected due to exchange). ^{13}C NMR (126 MHz, CD_2Cl_2) δ 171.25, 170.77, 170.03 (d, J = 20.1 Hz), 157.33, 150.49, 148.98, 138.75, 138.15, 136.87, 135.85, 133.42, 133.24, 132.77, 132.12, 131.69, 129.82, 128.78, 127.63, 126.74, 125.25, 121.64, 118.84, 116.88, 116.07, 112.75, 112.41, 78.70 (d, J = 232.4 Hz), 73.54, 70.36, 59.06, 58.65, 57.63, 56.77, 47.04, 39.14, 39.12, 39.11, 38.06, 36.71, 35.82, 30.33, 30.31, 29.44, 29.40, 26.44, 26.06, 16.37, 13.88 (d, J = 10.5 Hz), 13.74 (d, J = 10.0 Hz). HRMS (ESI): m/z calcd for $C_{53}H_{63}N_{11}O_5SF$ [M + H]$^+$: 984.47129; found 984.47183.

Compound 16

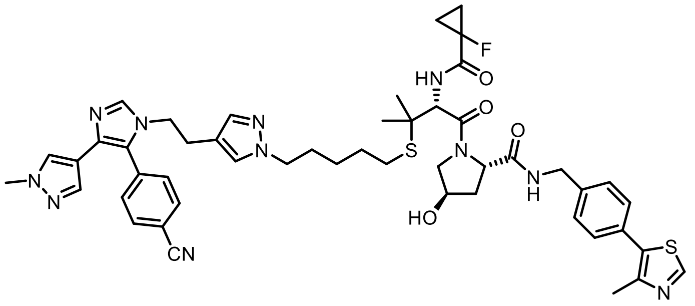

16 (19 mg, 86%, white solid) was prepared from **BAZ2 'pyrazole' warhead** (8 mg, 0.0233 mmol, 1.0 equiv) and VHL thiol-tosyl intermediate **S16** according to general procedure 8 and purified by flash column chromatography (2-10% MeOH in DCM). ^{1}H NMR (500 MHz, CD_2Cl_2) δ 8.65 (s, 1H), 7.79 – 7.75 (m, 2H), 7.45 – 7.37 (m, 5H), 7.34 (m, 4H), 7.25 (s, 1H), 7.18 (dd, J = 8.4, 2.7 Hz, 1H), 7.01 (d, J = 0.8 Hz, 1H), 6.92 (s, 1H), 4.87 (d, J = 7.8 Hz, 1H), 4.71 – 4.66 (m, 1H), 4.52 (t, J = 3.8 Hz, 1H), 4.48 – 4.37 (m, 2H), 3.98 – 3.91 (m, 4H), 3.86 (dd, J = 10.7, 2.3 Hz, 1H), 3.83 – 3.74 (m, 4H), 2.67 – 2.58 (m, 2H), 2.49 (d, J = 7.0 Hz, 5H), 2.41 – 2.34 (m, 1H), 2.18 (ddd, J = 13.0, 8.5, 4.0 Hz, 1H), 1.72 (p, J = 6.9 Hz, 2H, overlapping with water), 1.49 – 1.41 (m, 2H), 1.34 – 1.22 (m, 12H). (-O*H* not detected due to exchange). ^{13}C NMR (126 MHz, CD_2Cl_2) δ 171.23, 170.20 (d, J = 20.6 Hz), 170.12, 150.51, 149.01, 138.83, 138.68, 138.27, 136.91, 135.80, 133.39, 133.26, 131.89, 131.70, 131.36, 129.77, 128.34, 128.07, 127.66, 125.29, 118.83, 116.79, 116.45, 112.82, 78.64 (d, J = 231.9 Hz), 69.96, 59.66, 56.83, 55.96, 52.11, 48.50, 47.08, 43.19, 39.17, 37.25, 30.32, 29.00, 28.47, 26.30, 26.04, 25.99, 25.20, 16.35, 14.03 (d, J = 9.0 Hz), 13.95 (d, J = 9.5 Hz). ^{19}F NMR (376 MHz, CD_2Cl_2) δ -198.21. HRMS (ESI): m/z calcd for $C_{49}H_{57}N_{11}O_4S_2F$ [M + H]$^+$: 946.40150; found 946.40089.

Compound 17

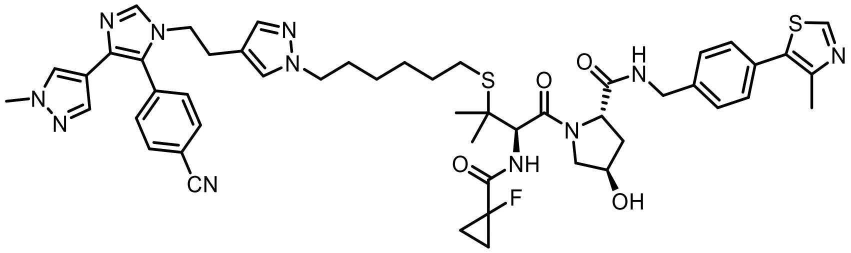

17 (8.68 mg, 54%, white solid) was prepared from BAZ2 'pyrazole' warhead-tosyl intermediate **S1** (10 mg, 0.0167 mmol, 1.0 equiv) and **VHL thiol**[29] according to general procedure 6 and purified by preparative TLC (1:2:2 MeOH:DCM:EtOAc) followed by trituration with Et_2O and pentane. ^1H NMR (500 MHz, CD_2Cl_2) δ 8.66 (s, 1H), 7.77 (d, *J* = 7.9 Hz, 2H), 7.46 – 7.32 (m, 9H), 7.26 (s, 1H), 7.19 – 7.13 (m, 1H), 7.04 (s, 1H), 6.89 (s, 1H), 4.88 (d, *J* = 8.5 Hz, 1H), 4.69 (t, *J* = 7.4 Hz, 1H), 4.53 (p, *J* = 4.6 Hz, 1H), 4.43 (d, *J* = 5.9 Hz, 2H), 3.95 (t, *J* = 6.8 Hz, 4H), 3.87 – 3.80 (m, 2H), 3.78 (s, 3H), 2.64 (t, *J* = 6.7 Hz, 2H), 2.54 – 2.45 (m, 5H), 2.39 (dt, *J* = 12.1, 5.7 Hz, 1H), 2.21 – 2.16 (m, 1H), 1.70 (p, *J* = 7.3 Hz, 2H), 1.46 – 1.37 (m, 2H), 1.34 – 1.25 (m, 11H), 1.22 – 1.15 (m, 2H). ^{13}C NMR (126 MHz, CD_2Cl_2) δ 171.18, 170.20 (d, *J* = 20.4 Hz), 170.12, 150.50, 149.00, 138.80, 138.66, 138.21, 136.90, 135.77, 133.30, 133.25, 131.89, 131.70, 131.35, 129.76, 128.35, 128.16, 127.63, 125.31, 118.82, 116.79, 116.37, 112.80, 78.64 (d, *J* = 232.4 Hz), 69.84, 59.65, 56.54, 55.98, 52.21, 48.34, 47.03, 43.16, 39.18, 37.31, 30.57, 29.17, 28.75, 28.61, 26.24, 26.21, 26.07, 24.90, 16.35, 14.05 (d, *J* = 6.5 Hz), 13.96 (d, *J* = 6.5 Hz). ^{19}F NMR (377 MHz, CD_2Cl_2) δ -198.25. HRMS (ESI): m/z calcd for $C_{50}H_{59}FN_{11}O_4S_2$ $[M + H]^+$: 960.41631; found 960.41632.

Compound 18

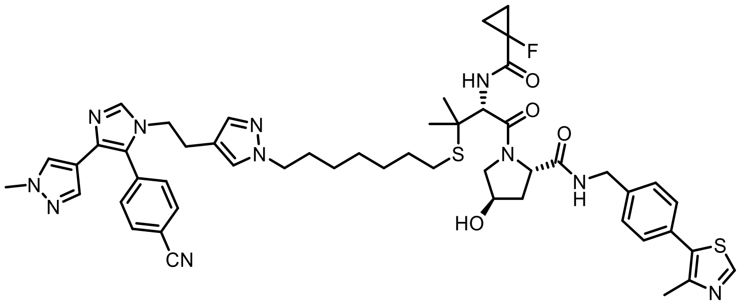

18 (7.68 mg, 40%, white solid) was prepared from BAZ2 'pyrazole' warhead-tosyl intermediate **S2** (11 mg, 0.0196 mmol, 1.0 equiv) and **VHL thiol**[29] according to general procedure 6 and purified by preparative TLC (1:2:2 MeOH:DCM:EtOAc) followed by trituration with Et_2O and pentane. ^1H NMR (500 MHz, CD_2Cl_2) δ 8.65 (s, 1H), 7.77 (d, *J* = 6.9 Hz, 2H), 7.45 (s, 1H), 7.42 – 7.37 (m, 5H), 7.35 – 7.32 (m, 3H), 7.26 (s, 1H), 7.19 – 7.15 (m, 1H), 7.03 (s, 1H), 6.92 (s, 1H), 4.85 (d, *J* = 8.5 Hz, 1H), 4.69 (t, *J* = 7.6 Hz, 1H), 4.51 (p, *J* = 4.2 Hz, 1H), 4.43 (d, *J* = 6.0 Hz, 2H), 3.95 (q, *J* = 7.3 Hz, 4H), 3.89 – 3.85 (m, 1H), 3.81 (d, *J* = 4.6 Hz, 1H), 3.78 (s, 3H), 2.65 (t, *J* = 6.8 Hz, 2H), 2.55 – 2.43 (m, 5H), 2.37 (dt, *J* = 12.3, 6.0 Hz, 1H), 2.22 – 2.15 (m, 1H), 1.72 (p, *J* = 7.3 Hz, 2H), 1.41 (td, *J* = 13.9, 6.9 Hz, 2H), 1.35 – 1.15 (m, 16H). ^{13}C NMR (126 MHz, CD_2Cl_2) δ 171.21, 170.23 (d, *J* = 20.4 Hz), 170.15, 150.50, 148.99, 138.82, 138.59, 138.12, 136.89, 135.78, 133.29, 133.23, 131.90, 131.70, 131.34, 129.75, 128.31, 128.07, 127.62, 125.33, 118.84, 116.83, 116.32, 112.78, 78.64

(d, J = 231.9 Hz), 70.00, 59.64, 56.67, 56.12, 52.38, 48.39, 47.03, 43.15, 39.17, 37.44, 30.64, 29.43, 29.12, 28.86, 28.59, 26.60, 26.18, 26.14, 25.03, 16.35, 14.04 (d, J = 6.0 Hz), 13.96 (d, J = 6.5 Hz). ^{19}F NMR (377 MHz, CD_2Cl_2) δ -198.20. HRMS (ESI): m/z calcd for $C_{51}H_{61}FN_{11}O_4S_2$ [M + H]$^+$: 974.43308; found 974.43314.

Compound 19

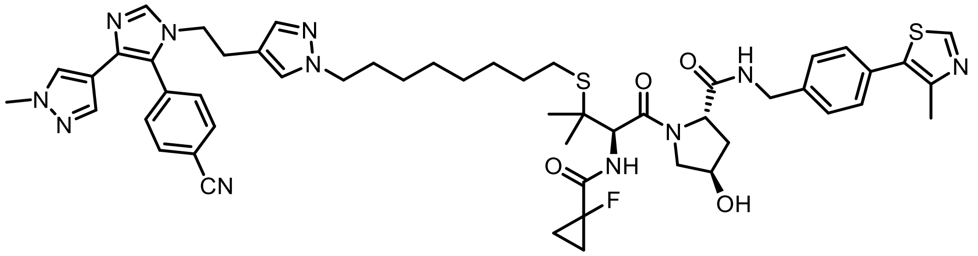

19 (20 mg, 87%, white solid) was prepared from **BAZ2 'pyrazole' warhead** (8 mg, 0.0233 mmol, 1.0 equiv) and VHL thiol-tosyl intermediate **S17** according to general procedure 8 and purified by flash column chromatography (2-10% MeOH in DCM). ^{1}H NMR (400 MHz, CD_2Cl_2) δ 8.65 (s, 1H), 7.79 – 7.74 (m, 2H), 7.48 – 7.43 (m, 2H), 7.42 – 7.37 (m, 4H), 7.35 – 7.31 (m, 3H), 7.26 (d, J = 0.9 Hz, 1H), 7.19 (dd, J = 8.7, 3.4 Hz, 1H), 7.02 (d, J = 0.9 Hz, 1H), 6.92 (d, J = 0.8 Hz, 1H), 4.85 (dd, J = 8.6, 1.2 Hz, 1H), 4.69 (t, J = 7.8 Hz, 1H), 4.54 – 4.47 (m, 1H), 4.42 (d, J = 6.0 Hz, 2H), 4.07 (s, 1H), 3.99 – 3.91 (m, 4H), 3.88 (ddd, J = 11.2, 3.3, 1.5 Hz, 1H), 3.78 (s, 4H), 2.64 (t, J = 6.9 Hz, 2H), 2.55 – 2.42 (m, 5H), 2.32 (ddd, J = 13.3, 7.2, 4.8 Hz, 1H), 2.20 (dddd, J = 13.4, 8.4, 3.7, 1.4 Hz, 1H), 1.77 – 1.66 (m, 2H), 1.46 – 1.36 (m, 2H), 1.35 – 1.14 (m, 18H). ^{13}C NMR (126 MHz, CD_2Cl_2) δ 171.33, 170.19 (d, J = 20.4 Hz), 170.16, 150.50, 148.95, 138.83, 138.56, 138.13, 136.87, 135.84, 133.36, 133.20, 131.90, 131.68, 131.29, 129.72, 128.25, 128.02, 127.59, 125.31, 118.84, 116.92, 116.31, 112.72, 78.63 (d, J = 232.4 Hz), 70.06, 59.72, 56.85, 56.12, 52.44, 48.52, 47.00, 43.11, 39.15, 37.65, 30.70, 29.56, 29.26, 29.19, 29.14, 28.59, 26.72, 26.24, 26.10, 25.06, 16.34, 14.03 (d, J = 10.5 Hz), 13.94 (d, J = 10.5 Hz). HRMS (ESI): m/z calcd for $C_{52}H_{63}N_{11}O_4S_2F$ [M + H]$^+$: 988.44845; found 988.44769.

Compound 20

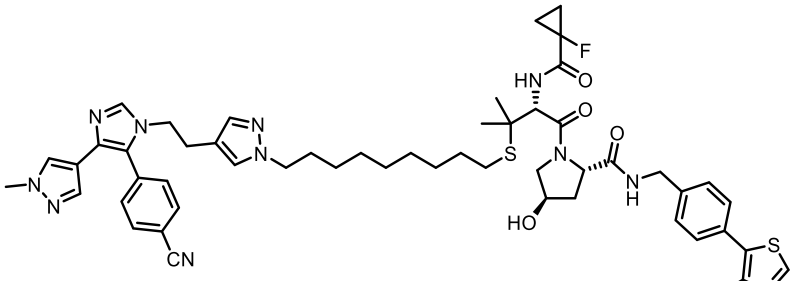

20 (9.5 mg, 51%, white solid) was prepared from BAZ2 'pyrazole' warhead-tosyl intermediate **S3** (12 mg, 0.0188 mmol, 1.0 equiv) and **VHL thiol**[29] according to general procedure 6 and purified by preparative TLC (1:2:2 MeOH:DCM:EtOAc) followed by trituration with Et_2O and pentane. ^{1}H NMR (500 MHz, CD_2Cl_2) δ 8.65 (s, 1H), 7.77 (d, J = 8.4 Hz, 2H), 7.47 (s, 1H), 7.43 – 7.36 (m, 5H), 7.36 – 7.32 (m, 3H), 7.25 (s, 1H), 7.20 – 7.13 (m, 1H), 7.03 (s, 1H), 6.93 (s, 1H), 4.85 (d, J = 8.4 Hz, 1H), 4.70 (t, J = 7.7 Hz, 1H), 4.55 –

4.49 (m, 1H), 4.43 (d, *J* = 5.9 Hz, 2H), 3.95 (dt, *J* = 13.1, 7.0 Hz, 4H), 3.94 – 3.88 (m, 1H), 3.80 (d, *J* = 4.5 Hz, 1H), 3.78 (s, 3H), 2.66 (t, *J* = 6.9 Hz, 2H), 2.57 – 2.43 (m, 5H), 2.41 – 2.31 (m, 1H), 2.25 – 2.16 (m, 1H), 1.73 (p, *J* = 7.2 Hz, 2H), 1.42 (p, *J* = 7.6 Hz, 2H), 1.35 – 1.19 (m, 14H). ^{13}C NMR (126 MHz, CD_2Cl_2) δ 171.21, 170.27 (d, *J* = 20.4 Hz), 170.25, 150.49, 148.99, 138.81, 138.54, 138.04, 136.89, 135.77, 133.22, 131.90, 131.71, 131.35, 129.75, 128.39, 128.29, 128.01, 127.62, 125.34, 118.83, 116.80, 116.27, 112.79, 78.64 (d, *J* = 231.9 Hz), 70.14, 59.63, 56.80, 56.16, 52.46, 48.40, 47.05, 43.14, 39.17, 37.58, 30.77, 29.60, 29.47, 29.31, 29.22, 28.62, 26.80, 26.25, 26.20, 24.93, 16.35, 14.05 (d, *J* = 6.0 Hz), 13.97 (d, *J* = 5.5 Hz). ^{19}F NMR (377 MHz, CD_2Cl_2) δ -198.21. HRMS (ESI): m/z calcd for $C_{53}H_{65}FN_{11}O_4S_2$ [M + H]$^+$: 1002.46397; found 1002.46336.

Compound 21

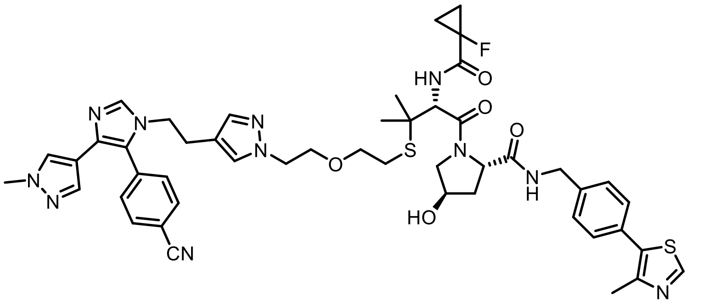

21 (14 mg, 63%, white solid) was prepared from **BAZ2 'pyrazole' warhead** (8 mg, 0.0233 mmol, 1.0 equiv) and VHL thiol-tosyl intermediate **S18** according to general procedure 8 and purified by preparative TLC (10% MeOH in DCM). ^{1}H NMR (500 MHz, CD_2Cl_2) δ 8.65 (s, 1H), 7.79 – 7.74 (m, 2H), 7.55 (s, 1H), 7.42 – 7.37 (m, 4H), 7.36 – 7.33 (m, 3H), 7.29 (t, *J* = 6.0 Hz, 1H), 7.25 (d, *J* = 0.9 Hz, 1H), 7.22 (dd, *J* = 8.7, 3.4 Hz, 1H), 7.09 (d, *J* = 0.6 Hz, 1H), 7.01 (d, *J* = 0.9 Hz, 1H), 4.88 (dd, *J* = 8.8, 1.1 Hz, 1H), 4.67 (t, *J* = 7.6 Hz, 1H), 4.53 – 4.49 (m, 1H), 4.45 (dd, *J* = 15.2, 6.0 Hz, 1H), 4.39 (dd, *J* = 15.2, 5.9 Hz, 1H), 4.12 (td, *J* = 5.1, 1.8 Hz, 2H), 3.95 (td, *J* = 6.9, 1.8 Hz, 2H), 3.87 – 3.83 (m, 1H), 3.80 (dd, *J* = 10.8, 4.3 Hz, 1H), 3.78 (s, 3H), 3.66 (t, *J* = 5.2 Hz, 2H), 3.48 (dt, *J* = 9.9, 6.3 Hz, 1H), 3.40 (dt, *J* = 9.9, 6.5 Hz, 1H), 2.73 – 2.60 (m, 4H), 2.48 (s, 3H), 2.35 (ddd, *J* = 13.1, 7.1, 5.0 Hz, 1H), 2.23 – 2.14 (m, 1H), 1.36 – 1.24 (m, 10H), (-O*H* not detected due to exchange). ^{13}C NMR (126 MHz, CD_2Cl_2) δ 171.35, 170.17 (d, *J* = 20.6 Hz), 169.96, 150.53, 149.00, 138.87, 138.84, 138.28, 136.86, 135.66, 133.25, 133.11, 131.87, 131.74, 131.34, 129.76, 129.20, 128.29, 127.68, 125.40, 118.82, 116.66, 116.66, 112.86, 78.65 (d, *J* = 232.4 Hz), 70.55, 69.99, 69.95, 59.77, 57.07, 56.10, 52.31, 48.71, 47.02, 43.22, 39.17, 37.22, 28.80, 26.14, 25.69, 25.65, 16.33, 14.03 (d, *J* = 10.0 Hz), 13.95 (d, *J* = 10.5 Hz). ^{19}F NMR (376 MHz, CD_2Cl_2) δ -198.20. HRMS (ESI): m/z calcd for $C_{48}H_{55}N_{11}O_5S_2F$ [M + H]$^+$: 948.38076; found 948.37908.

Compound 22

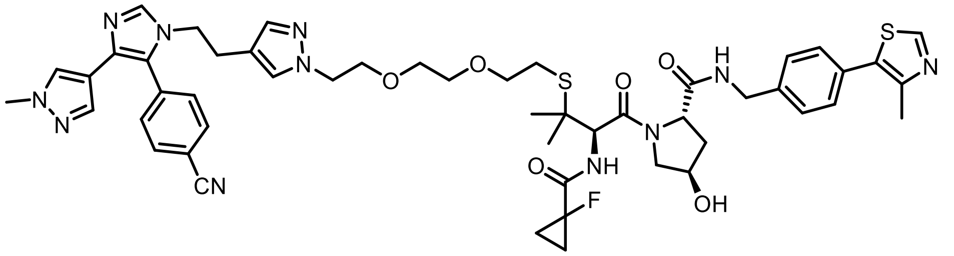

22 (13 mg, 59%, white solid) was prepared from BAZ2 'pyrazole' warhead-tosyl intermediate **S5** (14 mg, 0.0222 mmol, 1.0 equiv) and **VHL thiol**[29] according to general procedure 6 and purified by Flash Column Chromatography (2-10% MeOH in DCM). ^{1}H NMR (500 MHz, CD_2Cl_2) δ 8.65 (s, 1H), 7.79 – 7.73 (m, 2H), 7.58 (s, 1H), 7.42 – 7.37 (m, 4H), 7.37 – 7.29 (m, 4H), 7.25 (s, 1H), 7.20 (dd, *J* = 8.9, 3.3 Hz, 1H), 7.07 (s, 1H), 7.04 (s, 1H), 4.92 (dd, *J* = 8.8, 1.2 Hz, 1H), 4.67 (t, *J* = 7.6 Hz, 1H), 4.52 (p, *J* = 4.0 Hz, 1H), 4.43 (d, *J* = 6.2 Hz, 2H), 4.15 (t, *J* = 5.2 Hz, 2H), 3.95 (hept, *J* = 6.9 Hz, 2H), 3.89 – 3.81 (m, 2H), 3.78 (s, 3H), 3.71 (t, *J* = 5.2 Hz, 2H), 3.52 (dt, *J* = 9.9, 6.2 Hz, 1H), 3.49 – 3.40 (m, 5H), 2.78 – 2.63 (m, 4H), 2.49 (s, 3H), 2.34 (ddd, *J* = 13.0, 7.1, 4.9 Hz, 1H), 2.24 – 2.15 (m, 1H), 1.37 – 1.24 (m, 10H), (-O*H* not detected due to exchange). ^{13}C NMR (126 MHz, CD_2Cl_2) δ 171.39, 170.12 (d, *J* = 20.4 Hz), 169.98, 150.49, 148.99, 138.88, 138.22, 136.86, 135.67, 133.25, 133.22, 133.08, 131.89, 131.75, 131.31, 129.74, 129.20, 128.30, 127.61, 125.43, 118.83, 116.73, 116.55, 112.81, 78.63 (d, *J* = 232.4 Hz), 70.75, 70.60, 70.48, 70.09, 69.84, 59.86, 57.01, 55.86, 52.43, 48.69, 46.94, 43.17, 39.17, 37.37, 28.99, 26.29, 25.80, 25.32, 16.34, 14.04 (d, *J* = 10.0 Hz), 13.94 (d, *J* = 10.5 Hz). HRMS (ESI): m/z calcd for $C_{50}H_{59}N_{11}O_6S_2F$ [M + H]$^+$: 992.40697; found 992.40731.

Compound 23

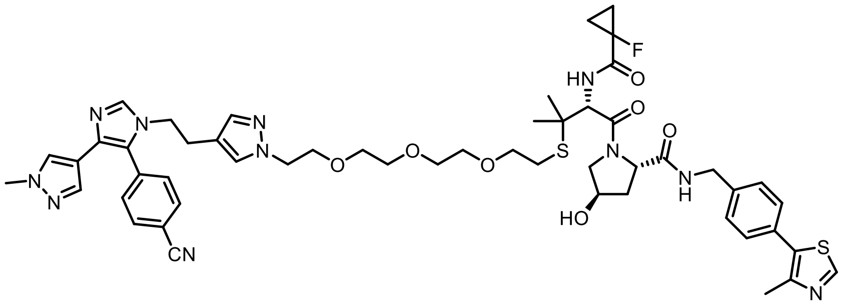

23 (18 mg, 73%, white solid) was prepared from **BAZ2 'pyrazole' warhead** (8 mg, 0.0233 mmol, 1.0 equiv) and VHL thiol-tosyl intermediate **S19** according to general procedure 8 and purified by preparative TLC (8% MeOH in DCM). ^{1}H NMR (500 MHz, CD_2Cl_2) δ 8.65 (s, 1H), 7.80 – 7.74 (m, 2H), 7.57 (s, 1H), 7.44 – 7.33 (m, 9H), 7.24 (s, 1H), 7.19 (dd, *J* = 8.8, 3.3 Hz, 1H), 7.10 (s, 1H), 7.04 (s, 1H), 4.91 (dd, *J* = 8.8, 1.2 Hz, 1H), 4.67 (t, *J* = 7.7 Hz, 1H), 4.53 (p, *J* = 4.0 Hz, 1H), 4.43 (dd, *J* = 6.0, 3.5 Hz, 1H), 4.17 (t, *J* = 5.2 Hz, 2H), 3.96 (t, *J* = 7.0 Hz, 2H), 3.89 (dd, *J* = 10.8, 4.3 Hz, 1H), 3.86 – 3.80 (m, 1H), 3.78 (s, 3H), 3.74 (t, *J* = 5.3 Hz, 2H), 3.57 (dt, *J* = 9.9, 6.2 Hz, 1H), 3.52 – 3.42 (m, 9H), 2.75 (td, *J* = 6.4, 4.9 Hz, 2H), 2.68 (t, *J* = 7.0 Hz, 2H), 2.49 (s, 3H), 2.30 (ddd, *J* = 12.4, 7.3, 4.8 Hz, 1H), 2.26 – 2.15 (m, 1H), 1.35 – 1.26 (m, 10H). ^{13}C NMR (126 MHz, CD_2Cl_2) δ 171.51, 170.13 (d, *J* = 20.6 Hz), 169.99, 150.51, 148.96, 138.92, 138.88, 138.07, 136.86, 135.59, 133.24,

132.95, 131.92, 131.75, 131.27, 129.72, 129.15, 128.34, 127.72, 125.38, 118.82, 116.53, 116.47, 112.86, 78.62 (d, *J* = 232.4 Hz), 70.88, 70.77, 70.74, 70.43, 70.18, 69.90, 59.92, 57.03, 55.89, 52.43, 48.64, 47.00, 43.11, 39.16, 37.61, 28.93, 26.22, 25.97, 25.20, 16.33, 14.04 (d, *J* = 10.5 Hz), 13.93 (d, *J* = 10.5 Hz). ^{19}F NMR (376 MHz, CD_2Cl_2) δ -198.16. HRMS (ESI): m/z calcd for $C_{52}H_{63}N_{11}O_7S_2F$ $[M + H]^+$: 1036.43433; found 1036.43374.

Compound 24

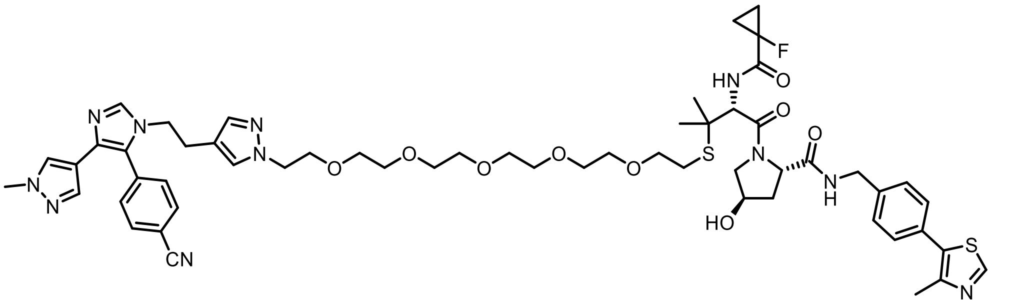

24 (16 mg, 69%, white solid) was prepared from **BAZ2 'pyrazole' warhead** (7 mg, 0.0204 mmol, 1.0 equiv) and VHL thiol-tosyl intermediate **S20** according to general procedure 8 and purified by preparative TLC (8% MeOH in DCM). ^{1}H NMR (500 MHz, CD_2Cl_2) δ 8.66 (s, 1H), 7.79 – 7.75 (m, 2H), 7.51 (s, 1H), 7.46 – 7.38 (m, 5H), 7.35 (dd, *J* = 6.2, 2.2 Hz, 3H), 7.24 (s, 1H), 7.17 (dd, *J* = 8.7, 3.3 Hz, 1H), 7.11 (s, 1H), 7.03 (s, 1H), 4.91 (dd, *J* = 8.8, 1.2 Hz, 1H), 4.66 (t, *J* = 7.8 Hz, 1H), 4.53 (p, *J* = 4.2 Hz, 1H), 4.46 (dd, *J* = 15.2, 6.1 Hz, 1H), 4.40 (dd, *J* = 15.2, 6.0 Hz, 1H), 4.17 (t, *J* = 5.3 Hz, 2H), 3.95 (t, *J* = 7.2 Hz, 2H), 3.90 (dd, *J* = 10.7, 4.4 Hz, 1H), 3.84 (ddd, *J* = 11.0, 3.0, 1.3 Hz, 1H), 3.78 (s, 3H), 3.75 (t, *J* = 5.3 Hz, 2H), 3.60 – 3.42 (m, 18H), 2.83 – 2.70 (m, 2H), 2.67 (t, *J* = 7.1 Hz, 2H), 2.49 (s, 3H), 2.29 (ddd, *J* = 12.1, 7.4, 4.8 Hz, 1H), 2.20 (dddd, *J* = 13.2, 9.7, 3.7, 1.4 Hz, 1H), 1.37 – 1.23 (m, 10H), (-O*H* not detected due to exchange). ^{13}C NMR (126 MHz, CD_2Cl_2) δ 171.45, 170.07 (d, *J* = 20.4 Hz), 170.06, 150.48, 148.96, 138.94, 138.86, 138.13, 136.85, 135.82, 133.26, 133.21, 131.92, 131.73, 131.27, 129.72, 129.15, 128.35, 127.63, 125.32, 118.85, 116.90, 116.47, 112.73, 78.62 (d, *J* = 232.4 Hz), 70.91, 70.88, 70.85, 70.76, 70.40, 70.16, 69.97, 59.90, 56.98, 55.92, 52.43, 48.56, 46.95, 43.09, 39.15, 37.72, 28.92, 26.30, 26.12, 25.03, 16.34, 14.03 (d, *J* = 10.5 Hz), 13.91 (d, *J* = 10.5 Hz). ^{19}F NMR (376 MHz, CD_2Cl_2) δ -198.15. HRMS (ESI): m/z calcd for $C_{56}H_{71}N_{11}O_9S_2F$ $[M + H]^+$: 1124.48562; found 1124.48563.

Compound 25

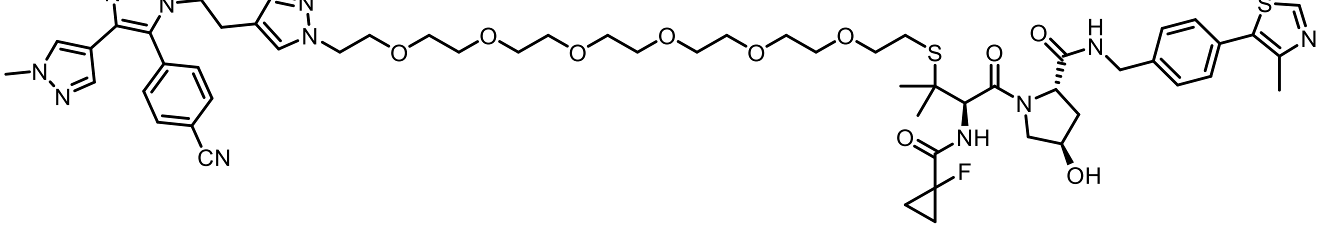

25 (9.3 mg, 34%, white solid) was prepared from **BAZ2 ‘pyrazole’ warhead** (8 mg, 0.0233 mmol, 1.0 equiv) and VHL thiol-tosyl intermediate **S21** according to general procedure 8 and purified by preparative TLC (9% MeOH:DCM) followed by trituration with Et_2O and pentane. ^1H NMR (500 MHz, CD_2Cl_2) δ 8.66 (s, 1H), 7.77 (d, *J* = 6.8 Hz, 2H), 7.49 (s, 1H), 7.44 – 7.37 (m, 5H), 7.37 – 7.33 (m, 3H), 7.24 (s, 1H), 7.19 – 7.14 (m, 1H), 7.11 (s, 1H), 7.03 (s, 1H), 4.91 (d, *J* = 8.6 Hz, 1H), 4.66 (t, *J* = 7.8 Hz, 1H), 4.56 – 4.50 (m, 1H), 4.47 (dd, *J* = 15.1, 6.2 Hz, 1H), 4.40 (dd, *J* = 15.1, 5.9 Hz, 1H), 4.18 (t, *J* = 5.3 Hz, 2H), 3.95 (t, *J* = 7.1 Hz, 2H), 3.90 (dd, *J* = 10.9, 4.3 Hz, 1H), 3.88 – 3.83 (m, 1H), 3.78 (s, 3H), 3.75 (t, *J* = 5.3 Hz, 2H), 3.62 – 3.48 (m, 16H), 2.76 (qt, *J* = 12.3, 6.5 Hz, 2H), 2.67 (t, *J* = 7.0 Hz, 2H), 2.49 (s, 3H), 2.35 – 2.27 (m, 1H), 2.24 – 2.16 (m, 1H), 1.36 – 1.23 (m, 10H). ^{13}C NMR (126 MHz, CD_2Cl_2) δ 171.00, 169.69 (d, *J* = 20.5 Hz), 169.70, 150.07, 148.57, 138.53, 138.46, 137.72, 136.44, 135.46, 132.91, 132.80, 131.51, 131.32, 130.89, 129.32, 128.73, 127.97, 127.21, 124.90, 118.46, 116.54, 116.08, 112.32, 78.22 (d, *J* = 232.4 Hz), 70.50, 70.47, 70.44, 70.34, 69.95, 69.76, 69.60, 59.45, 56.52, 55.53, 52.04, 48.08, 46.55, 42.69, 38.74, 37.27, 28.55, 25.92, 25.78, 24.56, 15.93, 13.63 (d, *J* = 10.0 Hz), 13.52 (d, *J* = 10.0 Hz). 5 signals belonging to aliphatic carbons are not listed as they overlap with the solvent peak. ^{19}F NMR (377 MHz, CD_2Cl_2) δ -198.18. HRMS (ESI): m/z calcd for $C_{58}H_{75}FN_{11}O_{10}S_2$ $[M + H]^+$: 1168.51062; found 1168.51062

Synthesis of 'pyrazole-CRBN' PROTACs

Compound 26

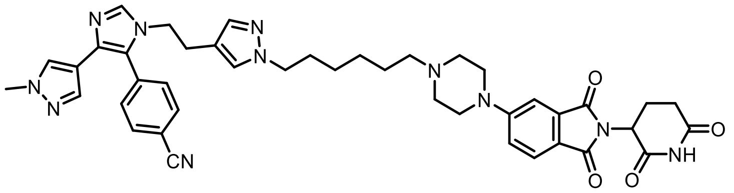

26 (4 mg, 49%, yellow solid) was prepared from BAZ2 'pyrazole' warhead-tosyl intermediate **S1** (6.3 mg, 0.0105 mmol, 1.0 equiv) and **pip-CRBN**[29] according to general procedure 9 and purified by preparative TLC (10% MeOH in DCM) followed by trituration with Et_2O and pentane. ^{1}H NMR (500 MHz, CD_2Cl_2) δ 8.11 (s, 1H), 7.80 (d, *J* = 8.1 Hz, 1H), 7.71 (d, *J* = 8.5 Hz, 1H), 7.49 (s, 1H), 7.48 – 7.41 (m, 2H), 7.37 (s, 1H), 7.32 (d, *J* = 2.3 Hz, 1H), 7.30 (s, 1H), 7.12 (dd, *J* = 8.7, 2.3 Hz, 1H), 7.07 (s, 1H), 6.97 (s, 1H), 4.96 (dd, *J* = 12.2, 5.1 Hz, 1H), 4.03 (t, *J* = 7.2 Hz, 2H), 3.98 (t, *J* = 7.1 Hz, 2H), 3.82 (s, 3H), 3.45 (t, *J* = 5.1 Hz, 4H), 2.92 – 2.74 (m, 4H), 2.70 (t, *J* = 7.0 Hz, 2H), 2.59 (t, *J* = 5.1 Hz, 4H), 2.39 (t, *J* = 7.5 Hz, 2H), 2.19 – 2.13 (m, 1H), 1.81 (p, *J* = 7.5 Hz, 2H), 1.55 – 1.48 (m, 2H), 1.43 – 1.26 (m, 4H). ^{13}C NMR (126 MHz, CD_2Cl_2) δ 170.94, 168.48, 167.86, 167.16, 155.60, 138.15, 137.64, 136.46, 135.56, 134.26, 133.07, 132.76, 131.28, 127.51, 127.12, 125.03, 124.86, 119.17, 118.46, 117.68, 116.69, 115.91, 112.26, 108.22, 58.20, 52.62, 52.05, 49.16, 47.51, 46.58, 38.74, 31.43, 30.41, 26.91, 26.59, 26.48, 25.95, 22.67. HRMS (ESI): m/z calcd for $C_{42}H_{46}N_{11}O_4$ $[M + H]^+$: 768.37207; found 768.37216.

Compound 27

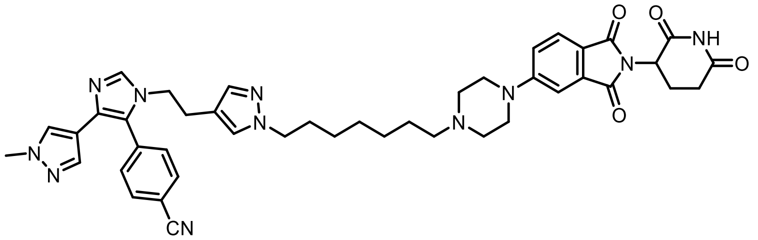

27 (4.49 mg, 58%, yellow solid) was prepared from BAZ2 'pyrazole' warhead-tosyl intermediate **S2** (6.1 mg, 0.01 mmol, 1.0 equiv) and **pip-CRBN**[29] according to general procedure 9 and purified by preparative TLC (10% MeOH in DCM) followed by trituration with Et_2O and pentane. ^{1}H NMR (500 MHz, CD_2Cl_2) δ 8.10 (s, 1H), 7.76 (d, *J* = 8.0 Hz, 1H), 7.67 (d, *J* = 8.5 Hz, 1H), 7.45 (s, 1H), 7.44 – 7.36 (m, 2H), 7.33 (s, 1H), 7.28 (d, *J* = 2.3 Hz, 1H), 7.26 (s, 1H), 7.10 – 7.05 (m, 1H), 7.03 (s, 1H), 6.93 (s, 1H), 4.92 (dd, *J* = 12.2, 5.2 Hz, 1H), 3.98 (t, *J* = 7.1 Hz, 2H), 3.94 (t, *J* = 7.1 Hz, 2H), 3.78 (s, 3H), 3.41 (t, *J* = 5.1 Hz, 4H), 2.87 – 2.70 (m, 4H), 2.66 (t, *J* = 7.1 Hz, 2H), 2.55 (t, *J* = 5.1 Hz, 4H), 2.35 (t, *J* = 7.5 Hz, 2H), 2.16 – 2.09 (m, 1H), 1.76 (p, *J* = 7.5 Hz, 2H), 1.47 (p, *J* = 6.9 Hz, 2H), 1.37 – 1.18 (m, 5H). ^{13}C NMR (126 MHz, CD_2Cl_2) δ 171.37, 168.90, 168.28, 167.57, 156.04, 138.54, 138.04, 136.86, 135.97, 134.68, 133.17, 131.75, 131.69, 127.91, 127.53, 125.43, 125.27, 119.56, 118.87, 118.07, 117.11, 116.31, 112.67, 108.61, 62.74, 58.72, 53.07, 52.50, 49.56, 47.94, 46.99, 39.15, 33.04, 31.85, 30.81, 30.66, 29.42, 29.03, 27.65, 27.11, 26.94, 26.69, 26.37, 23.08. HRMS (ESI): m/z calcd for $C_{43}H_{48}N_{11}O_4$ $[M + H]^+$: 782.38853; found 782.38773.

Compound 28

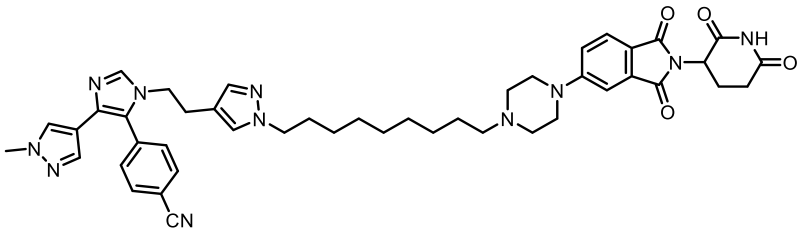

28 (4.9 mg, 55%, yellow solid) was prepared from BAZ2 'pyrazole' warhead-tosyl intermediate **S3** (7.1 mg, 0.0111 mmol, 1.0 equiv) and **pip-CRBN**[29] according to general procedure 9 and purified by preparative TLC (10% MeOH in DCM) followed by trituration with Et_2O and pentane. ^1H NMR (500 MHz, CD_2Cl_2) δ 8.13 (s, 1H), 7.77 (s, 1H), 7.75 (s, 1H), 7.67 (d, *J* = 8.5 Hz, 1H), 7.46 (s, 1H), 7.40 (d, *J* = 8.4 Hz, 1H), 7.34 (s, 1H), 7.28 (d, *J* = 2.3 Hz, 1H), 7.25 (s, 1H), 7.08 (dd, *J* = 8.7, 2.4 Hz, 1H), 7.02 (s, 1H), 6.93 (s, 1H), 4.92 (dd, *J* = 12.2, 5.1 Hz, 1H), 3.97 (t, *J* = 7.2 Hz, 2H), 3.93 (t, *J* = 7.0 Hz, 2H), 3.78 (s, 3H), 3.42 (t, *J* = 5.1 Hz, 4H), 2.88 – 2.70 (m, 4H), 2.66 (t, *J* = 7.0 Hz, 2H), 2.56 (t, *J* = 5.2 Hz, 4H), 2.36 (t, *J* = 7.6 Hz, 2H), 2.17 – 2.08 (m, 1H), 1.75 (p, *J* = 7.4 Hz, 2H), 1.53 – 1.44 (m, 2H), 1.34 – 1.21 (m, 9H). ^{13}C NMR (126 MHz, CD_2Cl_2) δ 171.38, 168.91, 168.28, 167.57, 156.02, 138.51, 138.04, 136.85, 135.96, 134.67, 133.46, 133.17, 131.69, 127.88, 127.56, 125.43, 125.28, 119.58, 118.86, 118.08, 117.09, 116.29, 112.68, 108.62, 58.81, 53.05, 52.52, 49.57, 47.91, 46.99, 39.15, 31.85, 30.86, 29.85, 29.81, 29.48, 27.79, 27.15, 26.97, 26.38, 23.08. HRMS (ESI): m/z calcd for $C_{45}H_{52}N_{11}O_4$ $[M + H]^+$: 810.41983; found 810.42068.

Compound 29

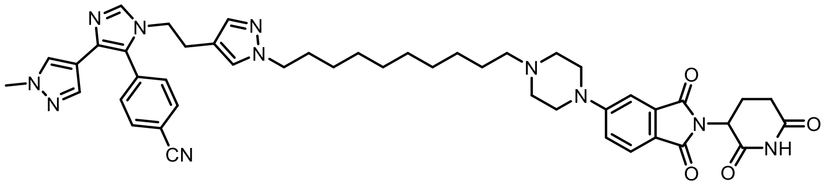

29 (3.37 mg, 41%, yellow solid) was prepared from BAZ2 'pyrazole' warhead-tosyl intermediate **S4** (6.6 mg, 0.0101 mmol, 1.0 equiv) and **pip-CRBN**[29] according to general procedure 9 and purified by preparative TLC (10% MeOH in DCM) followed by trituration with Et_2O and pentane. ^1H NMR (500 MHz, CD_2Cl_2) δ 8.11 (s, 1H), 7.77 (s, 1H), 7.75 (s, 1H), 7.67 (d, *J* = 8.5 Hz, 1H), 7.46 (s, 1H), 7.40 (d, *J* = 7.9 Hz, 1H), 7.34 (s, 1H), 7.28 (d, *J* = 2.3 Hz, 1H), 7.25 (s, 1H), 7.08 (dd, *J* = 8.4, 2.3 Hz, 1H), 7.02 (s, 1H), 6.93 (s, 1H), 4.92 (dd, *J* = 12.3, 5.2 Hz, 1H), 3.97 (t, *J* = 7.2 Hz, 2H), 3.93 (t, *J* = 7.1 Hz, 2H), 3.78 (s, 3H), 3.42 (t, *J* = 5.0 Hz, 4H), 2.87 – 2.70 (m, 4H), 2.66 (t, *J* = 7.0 Hz, 2H), 2.60 – 2.53 (m, 4H), 2.36 (s, 2H), 2.17 – 2.09 (m, 1H), 1.75 (p, *J* = 7.3 Hz, 2H), 1.53 – 1.44 (m, 2H), 1.37 – 1.17 (m, 11H). ^{13}C NMR (126 MHz, CD_2Cl_2) δ 170.95, 168.48, 167.86, 167.16, 155.60, 138.10, 137.63, 136.44, 135.55, 134.26, 133.05, 132.76, 131.28, 127.47, 127.14, 125.03, 124.86, 119.13, 118.45, 117.68, 116.69, 115.87, 112.26, 108.22, 58.41, 52.63, 52.12, 49.16, 47.49, 46.58, 38.73, 31.44, 30.45, 29.50, 29.48, 29.11, 27.41, 26.57, 25.97, 22.67. 2 signals belonging to aliphatic carbons are not listed as it overlaps with the solvent peak. HRMS (ESI): m/z calcd for $C_{46}H_{54}N_{11}O_4$ $[M + H]^+$: 824.43548; found 824.43683.

Compound 30

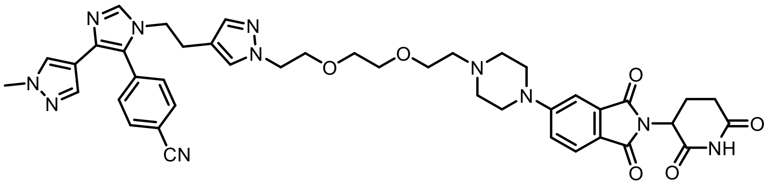

30 (5.7 mg, 98%, yellow solid) was prepared from BAZ2 'pyrazole' warhead-tosyl intermediate **S5** (11 mg, 0.0175 mmol, 1.0 equiv) and **pip-CRBN**[29] according to general procedure 9 and purified by preparative TLC (10% MeOH in DCM) followed by trituration with Et_2O. ^1H NMR (500 MHz, CD_2Cl_2) δ 8.23 (s, 1H), 7.78 – 7.74 (m, 2H), 7.67 (d, *J* = 8.5 Hz, 1H), 7.47 (s, 1H), 7.43 – 7.39 (m, 2H), 7.34 (s, 1H), 7.28 (d, *J* = 2.4 Hz, 1H), 7.25 (s, 1H), 7.10 – 7.06 (m, 2H), 7.05 (s, 1H), 4.97 – 4.88 (m, 1H), 4.17 (t, *J* = 5.4 Hz, 2H), 3.94 (t, *J* = 7.0 Hz, 2H), 3.79 – 3.74 (m, 5H), 3.58 (t, *J* = 5.5 Hz, 2H), 3.52 (s, 4H), 3.43 (t, *J* = 5.0 Hz, 4H), 2.90 – 2.58 (m, 11H), 2.16 – 2.09 (m, 1H). ^{13}C NMR (126 MHz, CD_2Cl_2) δ 171.42, 168.94, 168.25, 167.55, 155.89, 138.91, 138.06, 136.86, 135.93, 134.69, 133.40, 133.20, 131.73, 128.95, 127.57, 125.47, 125.27, 119.84, 118.87, 118.25, 117.06, 116.55, 112.71, 108.75, 70.87, 70.70, 70.19, 69.06, 58.05, 53.33, 52.48, 49.60, 47.76, 46.95, 39.15, 31.86, 26.38, 23.09. HRMS (ESI): m/z calcd for $C_{42}H_{46}N_{11}O_6$ [M + H]$^+$: 800.36270; found 800.36263.

Compound 31

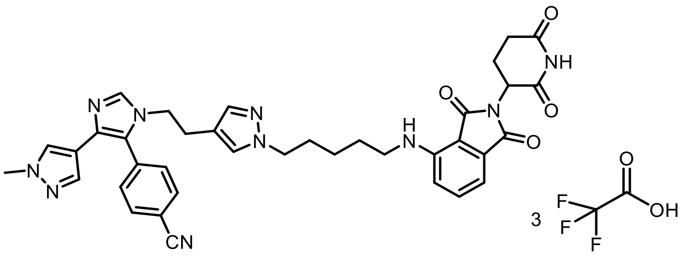

31 (8.6 mg, 14% over 3 steps, yellow solid) was prepared according to general procedure 10. Step 1: from **BAZ2 'pyrazole' warhead** (20 mg, 0.0582 mmol, 1.0 equiv) and 5-((tert-butoxycarbonyl)amino)pentyl 4-methylbenzenesulfonate[42] to obtain the corresponding 'pyrazole' warhead-*N*-Boc intermediate. Step 2: from crude 'pyrazole' warhead-*N*-Boc intermediate (26 mg, ~0.0488 mmol, 1.0 equiv) following protocol A to give the corresponding TFA salt of the amine. Step 3: from crude 'pyrazole' warhead-amine intermediate (19.6 mg, ~0.0254 mmol, 1.0 equiv) and 4-fluorothalidomide[30] (1.5 equiv) to obtain **31**. ^1H NMR (500 MHz, CD_2Cl_2) δ 8.77 (s, 1H), 8.43 (s, 1H), 7.92 – 7.86 (m, 2H), 7.74 (s, 1H), 7.50 (t, *J* = 8.7, 7.1 Hz, 1H), 7.48 – 7.43 (m, 2H), 7.21 (s, 1H), 7.18 (s, 1H), 7.13 (s, 1H), 7.05 (d, *J* = 7.1 Hz, 1H), 6.89 (d, *J* = 8.7 Hz, 1H), 4.95 – 4.87 (m, 1H), 4.09 (dt, *J* = 11.4, 7.1 Hz, 4H), 3.86 (s, 3H), 3.25 (td, *J* = 6.7, 1.3 Hz, 2H), 2.89 – 2.67 (m, 5H), 2.12 (ddd, *J* = 8.3, 6.2, 3.8 Hz, 1H), 1.84 (p, *J* = 7.2 Hz, 2H), 1.68 – 1.58 (m, 2H), 1.37 – 1.28 (m, 2H). (One -N*H* not detected due to exchange). ^{13}C NMR (126 MHz, CD_2Cl_2) δ 171.55, 169.86, 169.32, 167.94, 147.34, 137.99, 136.78, 136.49, 135.40, 134.04, 132.89, 132.01, 130.76, 129.68, 129.34, 126.48, 126.12, 117.99, 117.07, 115.46, 115.23, 111.45, 110.27, 108.91, 52.22, 49.32, 48.78, 42.53, 39.53, 31.81, 30.13, 28.86, 25.36, 23.97, 23.06. HRMS (ESI): m/z calcd for $C_{37}H_{37}N_{10}O_4$ [M + H]$^+$: 685.29938; found 685.29978.

Compound 32

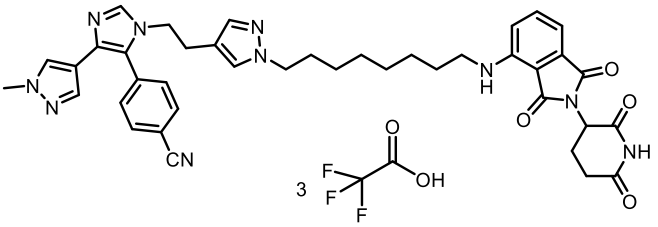

32 (9.6 mg, 15% over 3 steps, yellow solid) was prepared according to general procedure 10. Step 1: from **BAZ2 'pyrazole' warhead** (20 mg, 0.0582 mmol, 1.0 equiv) and 8-((tert-butoxycarbonyl)amino)octyl 4-methylbenzenesulfonate[43] to obtain the corresponding 'pyrazole' warhead-*N*-Boc intermediate. Step 2: from crude 'pyrazole' warhead-*N*-Boc intermediate (26 mg, ~0.0450 mmol, 1.0 equiv) following protocol B to give the corresponding hydrochloric salt of the amine. Step 3: from crude 'pyrazole' warhead-amine intermediate (40.4 mg, ~0.0696 mmol, 1.55 equiv) and 4-fluorothalidomide[30] (1.25 equiv) to obtain **32**. ^{1}H NMR (500 MHz, CD_2Cl_2) δ 8.76 (s, 2H), 8.35 (s, 0H), 7.92 – 7.88 (m, 2H), 7.74 (s, 1H), 7.51 (dd, *J* = 8.6, 7.0 Hz, 1H), 7.47 – 7.43 (m, 2H), 7.23 – 7.20 (m, 2H), 7.17 (s, 1H), 7.05 (d, *J* = 7.0 Hz, 1H), 6.92 (d, *J* = 8.6 Hz, 1H), 4.96 – 4.84 (m, 1H), 4.11 (t, *J* = 7.1 Hz, 2H), 4.07 (t, *J* = 7.2 Hz, 2H), 3.87 (s, 3H), 3.26 (t, *J* = 7.0 Hz, 2H), 2.89 – 2.68 (m, 5H), 2.19 – 2.09 (m, 1H), 1.77 (p, *J* = 7.5 Hz, 2H), 1.63 (p, *J* = 7.0 Hz, 2H), 1.42 – 1.35 (m, 2H), 1.34 – 1.27 (m, 4H), 1.23 (dd, *J* = 8.4, 5.6 Hz, 2H). (One -N*H* not detected due to exchange). ^{13}C NMR (126 MHz, CD_2Cl_2) δ 171.60, 169.91, 169.14, 168.00, 147.47, 137.74, 136.80, 136.45, 135.33, 134.06, 132.91, 132.01, 130.69, 129.70, 129.39, 126.50, 126.19, 117.98, 117.11, 115.50, 115.11, 111.35, 110.22, 108.86, 52.50, 49.32, 48.80, 42.98, 39.53, 31.80, 30.53, 29.43, 29.32, 29.13, 27.04, 26.61, 25.44, 23.10. HRMS (ESI): m/z calcd for $C_{40}H_{43}N_{10}O_4$ $[M + H]^+$: 727.34633; found 727.34662.

Compound 33

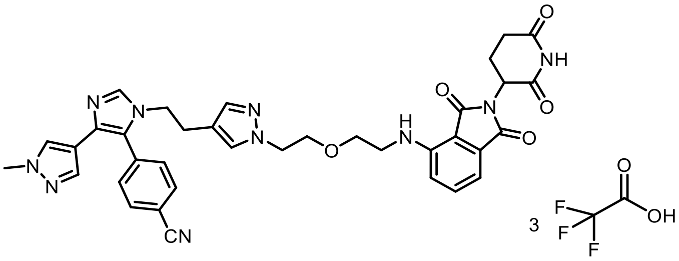

33 (13 mg, 22% over 3 steps, yellow solid) was prepared according to general procedure 10. Step 1: from **BAZ2 'pyrazole' warhead** (20 mg, 0.0582 mmol, 1.0 equiv) and 2-(2-((tert-butoxycarbonyl)amino)ethoxy)ethyl 4-methylbenzenesulfonate[44] to obtain the corresponding 'pyrazole' warhead-*N*-Boc intermediate. Step 2: from crude 'pyrazole' warhead-*N*-Boc intermediate (25 mg, ~0.0471 mmol, 1.0 equiv) following protocol A to give the corresponding TFA salt of the amine. Step 3: from crude 'pyrazole' warhead-amine intermediate (36 mg, ~0.0471 mmol, 1.0 equiv) and 4-fluorothalidomide[30] (1.2 equiv) to obtain **33**. ^{1}H NMR (500 MHz, CD_2Cl_2) δ 9.28 (s, 1H), 8.77 (s, 1H), 7.82 (dt, *J* = 7.9, 1.6 Hz, 2H), 7.73 (s, 1H), 7.48 (dd, *J* = 8.5, 7.1 Hz, 1H), 7.39 (dt, *J* = 7.9, 1.7 Hz, 2H), 7.16 (s, 1H), 7.15 (s, 1H), 7.06 (d, *J* = 6.9 Hz, 1H), 7.02 (s, 1H), 6.89 (d, *J* = 8.5 Hz, 1H), 4.97 – 4.89 (m, 1H), 4.26 – 4.18 (m, 2H), 3.95 (hept, *J* = 7.4 Hz, 2H), 3.83 (s, 3H), 3.82 – 3.76 (m,

2H), 3.59 (ddd, *J* = 9.9, 6.4, 3.6 Hz, 1H), 3.54 (ddd, *J* = 10.1, 6.2, 3.6 Hz, 1H), 3.32 (tdt, *J* = 13.9, 6.4, 3.6 Hz, 2H), 2.88 – 2.60 (m, 5H), 2.14 – 2.09 (m, 1H). (One -N*H* not detected due to exchange). ^{13}C NMR (126 MHz, CD_2Cl_2) δ 171.70, 170.08, 169.91, 167.92, 147.30, 138.51, 136.77, 136.42, 135.48, 133.82, 132.82, 132.09, 131.11, 130.28, 129.49, 126.32, 125.96, 118.10, 117.43, 115.20, 115.11, 111.66, 110.42, 109.18, 70.00, 69.68, 52.69, 49.45, 48.58, 42.51, 39.53, 31.90, 25.07, 22.97. HRMS (ESI): m/z calcd for $C_{36}H_{35}N_{10}O_5$ [M + H]$^+$: 687.27864; found 687.27837.

Compound 34

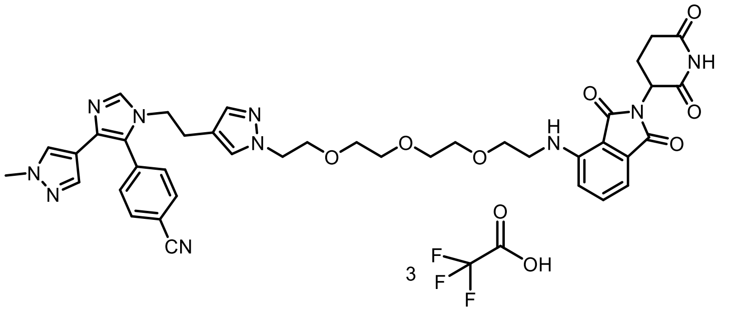

34 (9 mg, 14% over 3 steps, yellow solid) was prepared according to general procedure 10. Step 1: from **BAZ2 ‘pyrazole’ warhead** (20 mg, 0.0582 mmol, 1.0 equiv) and 2,2-dimethyl-4-oxo-3,8,11,14-tetraoxa-5-azahexadecan-16-yl 4-methylbenzenesulfonate[45] to obtain the corresponding ‘pyrazole’ warhead-*N*-Boc intermediate. Step 2: from crude ‘pyrazole’ warhead-*N*-Boc intermediate (18 mg, ~0.0291 mmol, 1.0 equiv) following protocol B to give the corresponding hydrochloric salt of the amine. Step 3: from crude ‘pyrazole’ warhead-amine intermediate (18 mg, ~0.0291 mmol, 1.0 equiv) and 4-fluorothalidomide[30] (1.2 equiv) to obtain **34**. ^{1}H NMR (500 MHz, CD_2Cl_2) δ 8.89 (s, 1H), 8.62 (s, 1H), 7.90 – 7.83 (m, 2H), 7.73 (s, 1H), 7.50 (t, *J* = 8.6, 7.0 Hz, 1H), 7.46 – 7.42 (m, 2H), 7.21 (s, 1H), 7.19 (s, 1H), 7.11 (s, 1H), 7.06 (d, *J* = 7.0 Hz, 1H), 6.93 (d, *J* = 8.6 Hz, 1H), 4.94 – 4.87 (m, 1H), 4.22 (td, *J* = 4.9, 3.2 Hz, 2H), 4.08 (t, *J* = 6.8 Hz, 2H), 3.84 (s, 3H), 3.76 (t, *J* = 5.1 Hz, 2H), 3.70 (t, *J* = 5.2 Hz, 2H), 3.65 – 3.59 (m, 4H), 3.59 – 3.53 (m, 4H), 3.43 (t, *J* = 5.2 Hz, 2H), 2.85 – 2.68 (m, 5H), 2.15 – 2.07 (m, 1H). (One -N*H* not detected due to exchange). ^{13}C NMR (126 MHz, CD_2Cl_2) δ 171.72, 169.84, 169.44, 167.91, 147.20, 138.65, 136.87, 136.48, 135.53, 133.98, 132.90, 132.04, 130.99, 130.04, 129.56, 126.56, 125.94, 118.09, 117.28, 115.26, 115.10, 111.76, 110.70, 109.06, 70.81, 70.64, 70.59, 70.55, 70.01, 69.64, 52.31, 49.38, 48.80, 42.74, 39.54, 31.84, 25.35, 23.10. HRMS (ESI): m/z calcd for $C_{40}H_{43}N_{10}O_7$ [M + H]$^+$: 775.33107; found 775.33032.

Compound 35

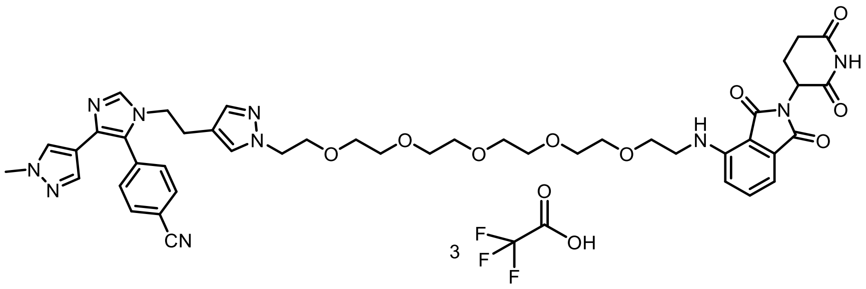

35 (9.4 mg, 27%, yellow solid) was prepared according to general procedure 10. Step 1: from **BAZ2 'pyrazole' warhead** (20 mg, 0.0582 mmol, 1.0 equiv) and 2,2-dimethyl-4-oxo-3,8,11,14,17,20-hexaoxa-5-azadocosan-22-yl 4-methylbenzenesulfonate[46] to obtain the corresponding 'pyrazole' warhead-*N*-Boc intermediate. Step 2: from crude 'pyrazole' warhead-*N*-Boc intermediate (28 mg, ~0.0382, 1.0 equiv) following protocol A to give the corresponding TFA salt of the amine. Step 3: from crude 'pyrazole' warhead-amine intermediate (28 mg, ~0.0291 mmol, 1.0 equiv) and 4-fluorothalidomide[30] (1.5 equiv) to obtain **35**. ^{1}H NMR (400 MHz, CD_2Cl_2) δ 8.79 (s, 1H), 8.62 (s, 1H), 7.89 – 7.84 (m, 2H), 7.74 (d, *J* = 0.8 Hz, 1H), 7.50 (dd, *J* = 8.5, 7.1 Hz, 1H), 7.47 – 7.41 (m, 2H), 7.25 (s, 1H), 7.18 (d, *J* = 0.8 Hz, 1H), 7.12 (s, 1H), 7.07 (dd, *J* = 7.1, 0.6 Hz, 1H), 6.93 (dd, *J* = 8.5, 0.6 Hz, 1H), 4.94 – 4.86 (m, 1H), 4.27 – 4.19 (m, 2H), 4.09 (t, *J* = 6.9 Hz, 2H), 3.84 (s, 3H), 3.78 – 3.71 (m, 2H), 3.71 – 3.66 (m, 2H), 3.64 – 3.50 (m, 16H), 3.43 (t, *J* = 5.3 Hz, 2H), 2.88 – 2.69 (m, 5H), 2.16 – 2.08 (m, 1H), (One -N*H* not detected due to exchange). ^{13}C NMR (126 MHz, CD_2Cl_2) δ 171.69, 169.80, 169.35, 167.97, 147.22, 138.80, 136.87, 136.45, 135.61, 133.91, 132.92, 132.10, 131.32, 129.77, 129.47, 126.76, 125.84, 118.15, 117.25, 115.53, 115.09, 111.68, 110.64, 109.40, 70.89, 70.74, 70.65, 70.58, 70.52, 70.47, 70.12, 69.62, 52.40, 49.40, 48.75, 42.70, 39.55, 31.87, 25.46, 23.10. (4 signals missing due to overlapping). HRMS (ESI): m/z calcd for $C_{44}H_{51}N_{10}O_9$ $[M + H]^+$: 863.38350; found 863.38269.

Synthesis of 'imidazole' PROTACs

Compound 36

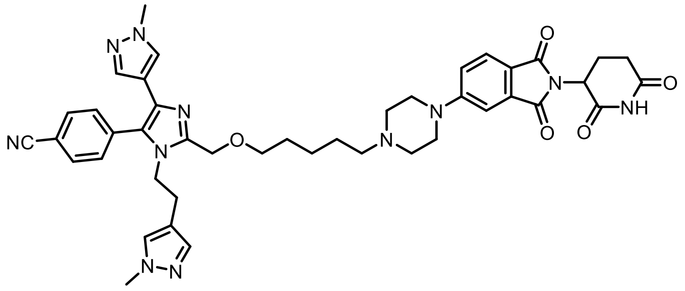

BAZ2 'imidazole' warhead [cpd 1 from Palaferri *et al.*][2] (5.5 mg, 0.0143 mmol, 1.0 equiv) and NaH 60 % dispersion in mineral oil (0.7 mg, 0.0186 mmol, 1.3 equiv) were suspended in dry DMF (0.15 mL) and the resulting suspension stirred at 0 °C for 1 hour. Then, a solution of ditosyl linker pentane-1,5-diyl bis(4-methylbenzenesulfonate)[39] (18 mg, 0.043 mmol, 3.0 equiv) was added and the reaction mixture stirred for 3 hours and allowed to warm up to 20 °C. After this time, the reaction was quenched with water, extracted with DCM and the organic layer washed with brine, dried over anhydrous $MgSO_4$, filtered and the solvent removed under reduced pressure to afford the corresponding BAZ2 'imidazole' warhead-tosyl intermediate which was used in the next step without further purification. BAZ2 'imidazole' warhead-tosyl intermediate obtained above (9 mg, ~0.0143 mmol, 1.0 equiv), **pip-CRBN**[29] (7.36 mg, 0.0215 mmol, 1.5 equiv) and NEt_3 (0.006 mL, 0.043 mmol, 3.0 equiv) were dissolved in dry DMF (0.14 mL) and the reaction mixture heated at 80 ºC for 4 h. After this time, the organic solvent was removed under reduced pressure and the resulting crude was purified by preparative TLC (10% MeOH in DCM) followed by trituration in Et_2O and pentane to afford **36** (4.0 mg, 35% over 2 steps, yellow solid). ^1H NMR (400 MHz, CD_2Cl_2) δ 8.04 (s, 1H), 7.83 – 7.77 (m, 2H), 7.70 (d, J = 8.6 Hz, 1H), 7.47 – 7.42 (m, 2H), 7.32 (s, 1H), 7.30 (d, J = 2.4 Hz, 1H), 7.26 (d, J = 0.8 Hz, 1H), 7.10 (dd, J = 8.6, 2.4 Hz, 1H), 7.00 (d, J = 0.8 Hz, 1H), 6.90 (s, 1H), 4.96 (dd, J = 12.1, 5.4 Hz, 1H), 4.54 (s, 2H), 4.09 – 4.01 (m, 2H), 3.81 (s, 3H), 3.79 (s, 3H), 3.59 (t, J = 6.5 Hz, 2H), 3.42 (t, J = 5.2 Hz, 4H), 2.93 – 2.76 (m, 3H), 2.70 – 2.64 (m, 2H), 2.60 – 2.55 (m, 4H), 2.41 – 2.36 (m, 2H), 1.73 – 1.64 (m, 2H), 1.49 – 1.41 (m, 3H). ^{13}C NMR (126 MHz, CD_2Cl_2) δ 170.93, 168.46, 167.85, 167.14, 155.60, 145.22, 138.41, 136.48, 135.81, 134.26, 132.74, 131.64, 131.38, 128.37, 127.06, 126.12, 125.02, 119.18, 118.46, 117.67, 116.59, 116.53, 112.33, 108.20, 70.68, 65.20, 58.19, 52.64, 49.15, 47.48, 45.50, 38.73, 38.68, 31.43, 29.54, 26.57, 25.60, 24.05, 22.67. HRMS (ESI): m/z calcd for $C_{43}H_{48}N_{11}O_5$ $[M + H]^+$: 798.38344, found 798.38371.

Compound 37

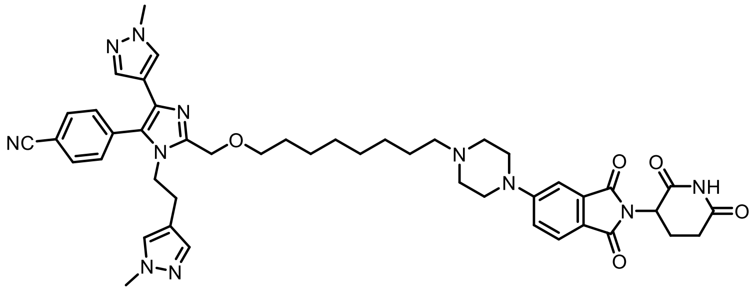

37 (4.4 mg, 27%, yellow solid) was prepared from BAZ2 'imidazole' warhead-tosyl intermediate **S8** (13 mg, 0.0194 mmol, 1.0 equiv) and **pip-CRBN**[29] according to general procedure 11 and purified by preparative TLC (10% MeOH in DCM) followed by trituration in Et_2O and pentane. ^1H NMR (400 MHz, CD_2Cl_2) δ 8.08 (s, 1H), 7.83 – 7.79 (m, 2H), 7.70 (d, *J* = 8.5 Hz, 1H), 7.47 – 7.42 (m, 2H), 7.36 – 7.29 (m, 2H), 7.26 (dd, *J* = 2.3, 0.8 Hz, 1H), 7.11 (dd, *J* = 8.6, 2.4 Hz, 1H), 7.00 (d, *J* = 0.8 Hz, 1H), 6.90 (s, 1H), 5.00 – 4.51 (m, 1H), 4.54 (s, 2H), 4.08 – 4.00 (m, 2H), 3.81 (s, 3H), 3.79 (s, 3H), 3.57 (t, *J* = 6.6 Hz, 2H), 3.48 – 3.42 (m, 4H), 2.92 – 2.76 (m, 3H), 2.70 – 2.62 (m, 2H), 2.61 – 2.54 (m, 4H), 2.42 – 2.34 (m, 2H), 1.69 – 1.54 (m, 7H), 1.56 – 1.46 (m, 2H). ^{13}C NMR (126 MHz, CD_2Cl_2) δ 170.93, 168.47, 167.87, 167.16, 155.63, 145.27, 138.40, 136.47, 135.84, 134.26, 132.73, 131.62, 131.39, 128.37, 127.07, 126.12, 125.02, 119.14, 118.47, 117.64, 116.61, 116.55, 112.31, 108.20, 70.83, 65.22, 58.40, 52.66, 49.15, 47.52, 45.50, 38.73, 38.67, 31.43, 29.68, 29.48, 29.41, 27.39, 26.80, 26.15, 25.58, 22.67. HRMS (ESI): m/z calcd for $C_{46}H_{54}N_{11}O_5$ $[M + H]^+$: 840.43039, found 840.43096.

Compound 38

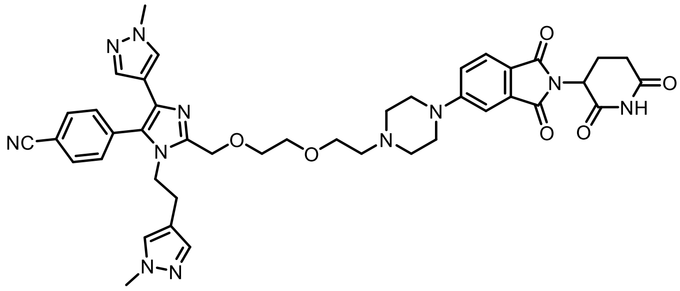

38 (3.0 mg, 25%, yellow solid) was prepared from BAZ2 'imidazole' warhead-tosyl intermediate **S9** (9.5 mg, 0.0151 mmol, 1.0 equiv) and **pip-CRBN**[29] according to general procedure 11 and purified by preparative TLC (2:2:1 EtOAc:DCM:MeOH) followed by trituration in Et_2O and pentane. ^1H NMR (500 MHz, CD_2Cl_2) δ 8.06 (s, 1H), 7.82 – 7.77 (m, 2H), 7.69 (dd, J = 8.5, 1.0 Hz, 1H), 7.46 – 7.41 (m, 2H), 7.32 (s, 1H), 7.29 (d, J = 2.3 Hz, 1H), 7.27 (s, 1H), 7.09 (dd, J = 8.6, 2.3 Hz, 1H), 7.02 (s, 1H), 6.92 (s, 1H), 4.96 (dd, J = 12.3, 5.1 Hz, 1H), 4.61 (s, 2H), 4.06 (dd, J = 8.1, 6.8 Hz, 2H), 3.81 (s, 3H), 3.79 (s, 3H), 3.78 – 3.72 (m, 2H), 3.69 – 3.61 (m, 4H), 3.42 (t, J = 5.2 Hz, 2H), 2.91 – 2.74 (m, 3H), 2.70 – 2.58 (m, 6H), 2.17 – 2.13 (m, 1H), 1.39 – 1.25 (m, 4H). ^{13}C NMR (126 MHz, CD_2Cl_2) δ 170.92, 168.46, 167.84, 167.14, 155.57, 144.90, 138.49, 136.49, 135.75, 134.25, 132.74, 131.71, 131.38, 128.46, 127.08, 126.22, 125.02, 119.24, 118.46, 117.71, 116.59, 116.48, 112.35, 108.21,

70.23, 69.74, 68.93, 65.32, 57.68, 49.16, 47.45, 45.54, 38.68, 31.43, 29.69, 29.06, 25.59, 22.66. HRMS (ESI): m/z calcd for $C_{42}H_{46}N_{11}O_6$ [M + H]+: 800.3633; found 800.36407.

Compound 39

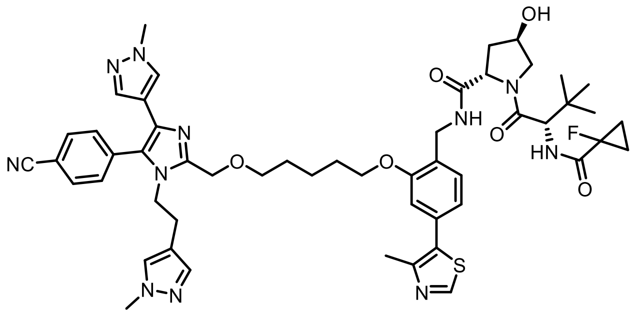

BAZ2 'imidazole' warhead [cpd 1 from Palaferri *et al.*][2] (5 mg, 0.0127 mmol, 1.0 equiv) and NaH 60 % dispersion in mineral oil (1.0 mg, 0.017 mmol, 1.3 equiv) were suspended in dry DMF (0.13 mL) and the resulting suspension stirred at 0 °C for 1 hour. Then, a solution of ditosyl linker pentane-1,5-diyl bis(4-methylbenzenesulfonate)[39] (16 mg, 0.038 mmol, 3.0 equiv) was added and the reaction mixture stirred for 3 hours and allowed to warm up to 20 °C. After this time, the reaction was quenched with water, extracted with DCM and the organic layer washed with brine, dried over anhydrous $MgSO_4$, filtered and the solvent removed under reduced pressure to afford the corresponding BAZ2 'imidazole' warhead-tosyl intermediate which was used in the next step without further purification. BAZ2 'imidazole' warhead-tosyl intermediate obtained above (8 mg, ~0.0127 mmol, 1.0 equiv), **VHL phenol**[28] (8.15 mg, 0.015 mmol, 1.2 equiv) and K_2CO_3 (3.52 mg, 0.0255 mmol, 2.0 equiv) were suspended in dry DMF (0.1 mL) and the resulting suspension heated at 80 °C for 3 h. After this time, the K_2CO_3 was filtered off and the organic solvent removed under reduced pressure. The crude product was purified by preparative TLC (1:2:2 MeOH:DCM:EtOAc) followed by trituration with Et_2O and pentane to afford **39** (7 mg, 56% over 2 steps, white solid). ^1H NMR (500 MHz, CD_2Cl_2) δ 8.66 (s, 1H), 7.76 (d, *J* = 8.3 Hz, 2H), 7.41 (d, *J* = 8.2 Hz, 2H), 7.34 (s, 1H), 7.32 (d, *J* = 7.6 Hz, 1H), 7.19 (s, 1H), 7.11 (t, *J* = 6.0 Hz, 1H), 7.01 – 6.98 (m, 1H), 6.96 (d, *J* = 9.5 Hz, 2H), 6.87 (d, *J* = 11.2 Hz, 2H), 4.62 – 4.55 (m, 2H), 4.55 – 4.45 (m, 4H), 4.32 (dd, *J* = 14.5, 4.9 Hz, 1H), 4.06 – 3.94 (m, 4H), 3.81 (d, *J* = 11.0 Hz, 1H), 3.77 (s, 3H), 3.73 (s, 3H), 3.64 (dd, *J* = 10.9, 4.4 Hz, 1H), 3.59 (t, *J* = 6.5 Hz, 2H), 2.61 (t, *J* = 7.4 Hz, 2H), 2.49 (s, 3H), 2.39 (dt, *J* = 12.5, 5.7 Hz, 1H), 2.04 – 1.97 (m, 1H), 1.89 – 1.81 (m, 2H), 1.71 (p, *J* = 7.0 Hz, 2H), 1.35 – 1.20 (m, 6H), 0.91 (s, 9H). ^{13}C NMR (126 MHz, CD_2Cl_2) δ 171.21, 170.67, 169.82 (d, *J* = 20.4 Hz), 157.38, 150.51, 149.01, 145.64, 138.79, 136.93, 135.94, 133.19, 132.89, 132.11, 131.84, 131.82, 130.24, 128.88, 127.77, 126.70, 126.65, 121.69, 118.82, 116.90, 116.44, 112.88, 112.46, 78.72 (d, *J* = 231.9 Hz), 71.21, 70.20, 68.39, 65.38, 59.13, 57.52, 56.63, 45.97, 39.22, 39.16, 39.04, 36.76, 35.97, 29.91, 29.39, 26.43, 25.93, 23.49, 16.39, 13.86 (d, *J* = 10.0 Hz), 13.67 (d, *J* = 10.0 Hz). ^{19}F NMR (377 MHz, CD_2Cl_2) δ -197.97. HRMS (ESI): m/z calcd for $C_{52}H_{63}FN_{11}O_6S$ $[M + H]^+$: 988.46514; found 988.46476.

Compound 40

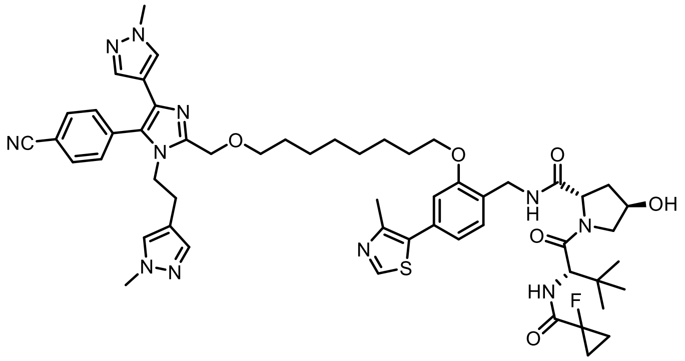

40 (8 mg, 52%, white solid) was prepared from BAZ2 'imidazole' warhead-tosyl intermediate **S8** (10 mg, 0.0149 mmol, 1.0 equiv) and **VHL phenol**[28] according to general procedure 12 and purified by preparative TLC (2:2:1 EtOAc:DCM:MeOH) followed by trituration in Et_2O and pentane. ^{1}H NMR (500 MHz, CD_2Cl_2) δ 8.65 (s, 1H), 7.85 (t, *J* = 6.0 Hz, 2H), 7.77 (d, *J* = 8.3 Hz, 2H), 7.59 (s, 1H), 7.47 – 7.41 (m, 3H), 7.13 (s, 1H), 7.06 – 7.02 (m, 1H), 6.97 – 6.92 (m, 2H), 6.88 (s, 1H), 6.83 (s, 1H), 4.68 (t, *J* = 7.8 Hz, 1H), 4.58 (d, *J* = 9.0 Hz, 1H), 4.56 – 4.53 (m, 1H), 4.51 (d, *J* = 7.1 Hz, 1H), 4.42 (d, *J* = 12.4 Hz, 1H), 4.35 (s, 1H), 4.31 (d, *J* = 5.8 Hz, 1H), 4.07 – 3.96 (m, 4H), 3.85 (d, *J* = 11.0 Hz, 1H), 3.80 (s, 3H), 3.76 (s, 3H), 3.64 (dd, *J* = 11.1, 4.1 Hz, 1H), 3.59 – 3.46 (m, 2H), 2.65 – 2.51 (m, 4H), 2.49 (s, 2H), 2.43 – 2.35 (m, 1H), 2.10 – 2.01 (m, 1H), 1.81 (p, *J* = 6.5 Hz, 2H), 1.63 – 1.57 (m, 2H), 1.54 – 1.46 (m, 2H), 1.42 – 1.33 (m, 6H), 1.33 – 1.23 (m, 2H), 0.95 (s, 9H). ^{13}C NMR (126 MHz, CD_2Cl_2) δ 171.26, 171.10, 169.81 (d, *J* = 20.4 Hz), 157.25, 150.42, 148.94, 145.98, 138.79, 136.77, 136.01, 133.24, 132.49, 132.22, 132.11, 131.77, 129.39, 129.00, 128.22, 126.96, 126.48, 121.53, 118.83, 116.95, 116.38, 112.85, 112.31, 78.74 (d, *J* = 232.4 Hz), 71.24, 70.40, 68.50, 66.07, 65.13, 59.28, 57.59, 56.94, 45.90, 39.12, 39.07, 37.02, 36.15, 29.93, 29.39, 29.36, 26.47, 26.39, 26.16, 25.70, 16.37, 15.50, 13.86 (d, *J* = 10.5 Hz), 13.67 (d, *J* = 10.0 Hz). ^{19}F NMR (471 MHz, CD_2Cl_2) δ -198.19. HRMS (ESI): m/z calcd for $C_{55}H_{69}FN_{11}O_6S$ $[M + H]^+$: 1030.51336; found 1030.51278.

Compound 41

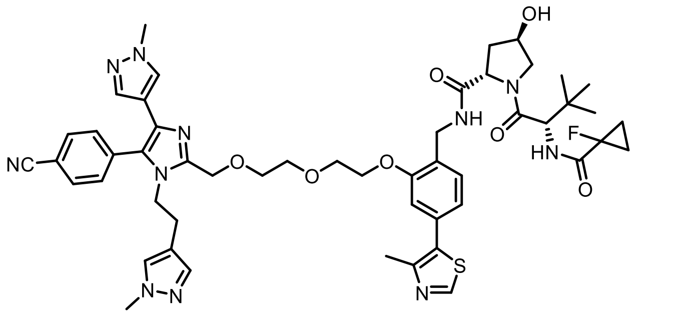

41 (8.3 mg, 41%, white solid) was prepared from BAZ2 'imidazole' warhead-tosyl intermediate **S9** (12.8 mg, 0.0203 mmol, 1.0 equiv) and **VHL phenol**[28] according to general procedure 12 and purified by preparative TLC (2:2:1 EtOAc:DCM:MeOH) followed by trituration in Et_2O and pentane. ^{1}H NMR (500 MHz, CD_2Cl_2) δ 8.66 (s, 1H), 7.75 (d, *J* = 7.8 Hz, 2H), 7.40 – 7.31 (m, 5H), 7.19 (s, 1H), 7.05 – 6.96 (m, 2H), 6.95 – 6.83 (m, 3H), 4.60 (d, *J* = 9.0 Hz, 1H), 4.56 – 4.40 (m, 6H), 4.22 – 4.10 (m, 2H), 3.99 (tt, *J* = 14.2, 7.0 Hz, 2H),

3.93 – 3.80 (m, 4H), 3.79 – 3.73 (m, 6H), 3.71 (s, 3H), 3.64 (d, *J* = 10.9 Hz, 1H), 2.59 (t, *J* = 7.3 Hz, 2H), 2.48 (s, 3H), 2.20 – 2.13 (m, 1H), 2.08 – 2.01 (m, 1H), 1.32 – 1.18 (m, 4H), 0.96 (s, 9H). ^{13}C NMR (126 MHz, CD_2Cl_2) δ 171.31, 170.70, 169.60 (d, *J* = 19.9 Hz), 157.39, 150.53, 149.06, 145.38, 138.87, 136.93, 135.81, 133.19, 132.82, 131.95, 131.92, 131.81, 130.33, 128.94, 127.83, 127.54, 126.72, 122.26, 118.79, 116.84, 116.30, 113.28, 112.93, 78.73 (d, *J* = 230.1 Hz), 71.03, 70.40, 70.29, 70.02, 68.59, 65.37, 59.41, 57.57, 57.06, 45.99, 39.52, 39.16, 39.02, 37.76, 36.33, 26.50, 25.86, 16.39, 13.84 (d, *J* = 10.5 Hz), 13.55 (d, *J* = 10.5 Hz). ^{19}F NMR (471 MHz, CD_2Cl_2) δ -197.88. HRMS (ESI): m/z calcd for $C_{51}H_{61}FN_{11}O_7S$ [M + H]$^+$: 990.44679; found 990.44721.

dBAZ2B

Synthetic route and characterization previously disclosed in Palaferri *et al.*[2]

Compound 42

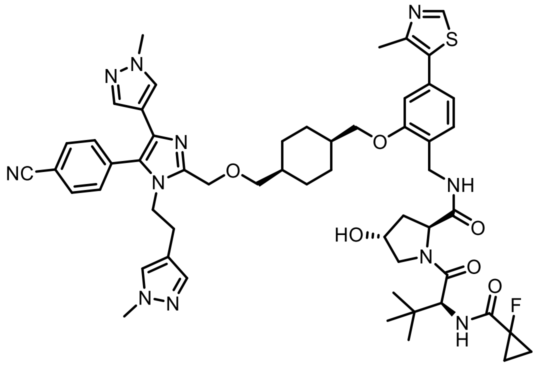

42 (4.5 mg, 27%, white solid) was prepared from BAZ2 'imidazole' warhead-tosyl intermediate **S10** (11 mg, 0.0165 mmol, 1.0 equiv) and **VHL phenol**[28] according to general procedure 12 and purified by preparative TLC (2:2:1 EtOAc:DCM:MeOH) followed by trituration in Et_2O and pentane. ^{1}H NMR (500 MHz, CD_2Cl_2) δ 8.66 (s, 1H), 7.77 (s, 1H), 7.76 (s, 1H), 7.41 (s, 1H), 7.39 (s, 1H), 7.37 (s, 1H), 7.35 – 7.30 (m, 2H), 7.15 (s, 1H), 6.99 – 6.94 (m, 2H), 6.93 (s, 1H), 6.89 (s, 1H), 6.88 (s, 1H), 4.68 (dd, *J* = 8.0, 6.0 Hz, 1H), 4.64 – 4.54 (m, 3H), 4.51 (d, *J* = 12.2 Hz, 2H), 4.28 (dd, *J* = 14.2, 4.4 Hz, 1H), 4.01 (t, *J* = 7.4 Hz, 2H), 3.89 (dd, *J* = 5.5, 2.0 Hz, 2H), 3.81 – 3.76 (m, 1H), 3.75 (s, 3H), 3.74 (s, 3H), 3.71 – 3.63 (m, 2H), 3.58 – 3.52 (m, 1H), 2.62 (t, *J* = 7.6 Hz, 2H), 2.56 – 2.51 (m, 1H), 2.50 (s, 3H), 1.98 (ddd, *J* = 12.7, 8.2, 4.6 Hz, 1H), 1.91 (s, 1H), 1.65 – 1.57 (m, 4H), 1.33 – 1.23 (m, 7H), 1.24 – 1.18 (m, 2H), 0.89 (s, 9H). ^{13}C NMR (126 MHz, CD_2Cl_2) δ 171.43, 170.52, 169.87 (d, *J* = 20.4 Hz), 157.45, 150.51, 149.02, 145.76, 138.80, 136.85, 136.02, 133.20, 132.96, 132.13, 131.85, 130.32, 128.90, 127.83, 126.77, 126.53, 121.67, 118.83, 116.93, 116.40, 112.88, 112.60, 104.60, 78.72 (d, *J* = 231.9 Hz), 73.83, 72.16, 70.20, 65.53, 59.10, 57.46, 56.14, 45.93, 39.13, 39.07, 38.83, 36.59, 36.45, 35.84, 35.27, 26.79, 26.56, 26.42, 25.94, 25.64, 25.45, 16.39, 13.86 (d, *J* = 10.0 Hz), 13.70 (d, *J* = 10.5 Hz). ^{19}F NMR (377 MHz, CD_2Cl_2) δ -198.02. HRMS (ESI): m/z calcd for $C_{55}H_{67}FN_{11}O_6S$ [M + H]$^+$: 1028.49750; found 1028.49804.

Compound 43

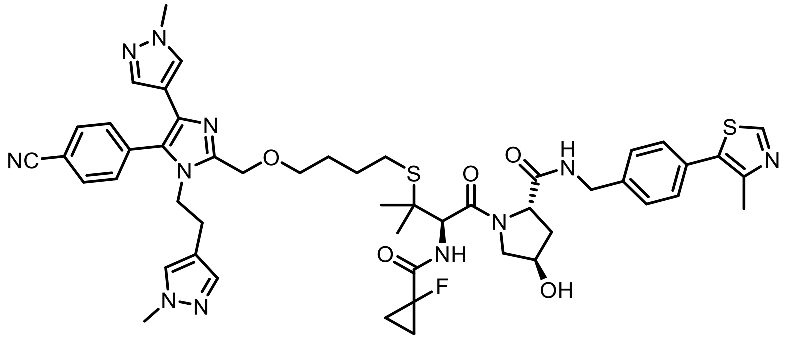

43 (6.8 mg, 33%, white solid) was prepared from BAZ2 'imidazole' warhead-tosyl intermediate **S7** (13 mg, 0.0212 mmol, 1.0 equiv) and **VHL thiol**[29] according to general procedure 13 and purified by preparative TLC (2:2:1 EtOAc:DCM:MeOH) followed by trituration in Et_2O and pentane. ^1H NMR (500 MHz, CD_2Cl_2) δ 8.64 (s, 1H), 7.77 (d, *J* = 8.0 Hz, 2H), 7.42 (d, *J* = 8.0 Hz, 2H), 7.40 – 7.34 (m, 3H), 7.34 – 7.30 (m, 3H), 7.19 (s, 1H), 7.15 – 7.09 (m, 1H), 7.00 (s, 1H), 6.86 (s, 1H), 4.93 (d, *J* = 8.8 Hz, 1H), 4.70 (dd, *J* = 8.8, 4.7 Hz, 1H), 4.57 (p, *J* = 5.6 Hz, 1H), 4.52 (d, *J* = 12.1 Hz, 1H), 4.45 (s, 1H), 4.41 (dd, *J* = 7.3, 2.3 Hz, 2H), 3.97 (h, *J* = 7.3 Hz, 2H), 3.86 (dd, *J* = 10.4, 5.5 Hz, 1H), 3.78 (s, 3H), 3.75 (s, 3H), 3.74 – 3.71 (m, 1H), 3.60 – 3.51 (m, 2H), 2.68 – 2.51 (m, 4H), 2.48 (s, 3H), 2.41 (dt, *J* = 12.9, 5.3 Hz, 1H), 2.20 – 2.13 (m, 1H), 1.73 – 1.62 (m, 2H), 1.56 – 1.51 (m, 2H), 1.34 – 1.23 (m, 10H). ^{13}C NMR (126 MHz, CD_2Cl_2) δ 171.13, 170.24, 169.93 (d, *J* = 20.4 Hz), 150.48, 149.00, 145.26, 138.77, 138.75, 136.88, 135.84, 133.20, 131.90, 131.82, 131.33, 129.77, 128.90, 128.49, 128.35, 127.69, 126.66, 118.80, 116.90, 116.33, 113.00, 78.62 (d, *J* = 232.4 Hz), 70.62, 69.24, 65.26, 59.69, 55.96, 55.53, 48.33, 46.02, 43.14, 39.19, 39.08, 37.08, 29.25, 28.71, 26.32, 26.07, 26.02, 24.71, 16.32, 13.99 (d, *J* = 10.5 Hz), 13.89 (d, *J* = 10.5 Hz). ^{19}F NMR (377 MHz, CD_2Cl_2) δ -198.26. HRMS (ESI): m/z calcd for $C_{50}H_{59}FN_{11}O_5S_2$ $[M + H]^+$: 976.41035; found 976.41040.

Compound 44

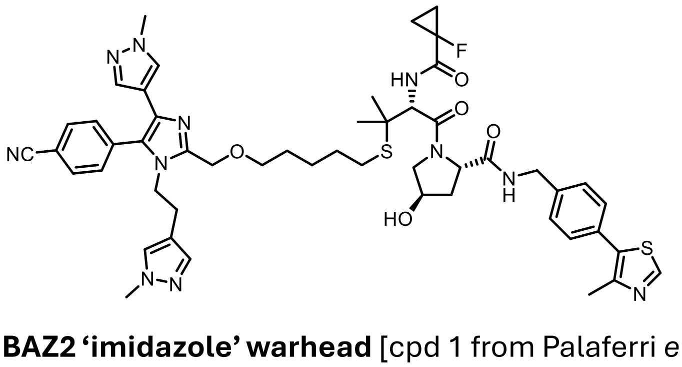

BAZ2 'imidazole' warhead [cpd 1 from Palaferri *et al.*][2] (9.2 mg, 0.0239 mmol, 1.0 equiv) and NaH 60 % dispersion in mineral oil (1.2 mg, 0.03 mmol, 1.3 equiv) were suspended in dry DMF (0.2 mL) and the resulting suspension stirred at 0 °C for 1 hour. Then, a solution of ditosyl linker pentane-1,5-diyl bis(4-methylbenzenesulfonate)[39] (30 mg, 0.072 mmol, 3.0 equiv) was added and the reaction mixture stirred for 3 hours and allowed to warm up to 20 °C. After this time, the reaction was quenched with water, extracted with DCM and the organic layer washed with brine, dried over anhydrous $MgSO_4$, filtered and the solvent removed under reduced pressure to afford the corresponding BAZ2 'imidazole' warhead-

tosyl intermediate which was used in the next step without further purification. BAZ2 'imidazole' warhead-tosyl intermediate obtained above (15 mg, ~0.0239 mmol, 1.0 equiv), **VHL thiol**[29] (17.2 mg, 0.0323 mmol, 1.35 equiv) were dissolved in dry THF (0.25 mL). DBU (0.005 mL, 0.036 mmol, 1.5 equiv) was added and the reaction mixture stirred at 22 °C for 15 hours. After this time, the organic solvent was removed under reduced pressure and the resulting crude product was purified by preparative TLC (1:2:2 MeOH:DCM:EtOAc) followed by trituration with Et_2O and pentane to afford **44** (9 mg, 38% over 2 steps, white solid). ^{1}H NMR (500 MHz, CD_2Cl_2) δ 8.65 (s, 1H), 7.77 (d, *J* = 8.1 Hz, 2H), 7.43 – 7.36 (m, 5H), 7.34 – 7.30 (m, 3H), 7.21 (s, 1H), 7.15 – 7.10 (m, 1H), 6.99 (s, 1H), 6.86 (s, 1H), 4.88 (d, *J* = 8.7 Hz, 1H), 4.69 (dd, *J* = 8.7, 5.5 Hz, 1H), 4.56 (q, *J* = 5.1 Hz, 1H), 4.49 (s, 2H), 4.42 (d, *J* = 5.9 Hz, 2H), 3.98 (t, *J* = 7.7 Hz, 2H), 3.85 (dd, *J* = 10.5, 5.1 Hz, 1H), 3.78 (s, 3H), 3.75 (s, 3H), 3.50 (q, *J* = 6.8 Hz, 2H), 2.62 (t, *J* = 7.4 Hz, 2H), 2.52 (t, *J* = 6.6 Hz, 2H), 2.49 (s, 3H), 2.41 (dt, *J* = 11.6, 5.4 Hz, 1H), 2.18 – 2.10 (m, 1H), 1.53 – 1.36 (m, 7H), 1.33 – 1.27 (m, 10H). ^{13}C NMR (126 MHz, CD_2Cl_2) δ 171.11, 170.31, 170.07 (d, *J* = 20.4 Hz), 150.50, 149.01, 145.47, 138.81, 138.78, 136.94, 135.97, 133.19, 131.96, 131.87, 131.85, 131.37, 129.77, 128.94, 128.35, 127.65, 126.61, 118.82, 116.97, 116.52, 112.89, 78.65 (d, *J* = 232.4 Hz), 70.84, 69.58, 65.42, 59.61, 56.28, 55.87, 48.31, 45.95, 43.18, 39.17, 39.07, 37.11, 29.46, 29.17, 28.76, 26.23, 26.15, 26.00, 24.82, 16.34, 14.02 (d, *J* = 8.0 Hz), 13.94 (d, *J* = 8.0 Hz). ^{19}F NMR (377 MHz, CD_2Cl_2) δ -198.23. HRMS (ESI): m/z calcd for $C_{51}H_{61}FN_{11}O_5S_2$ [M + H]$^+$: 990.42814; found 990.42814.

Compound 45

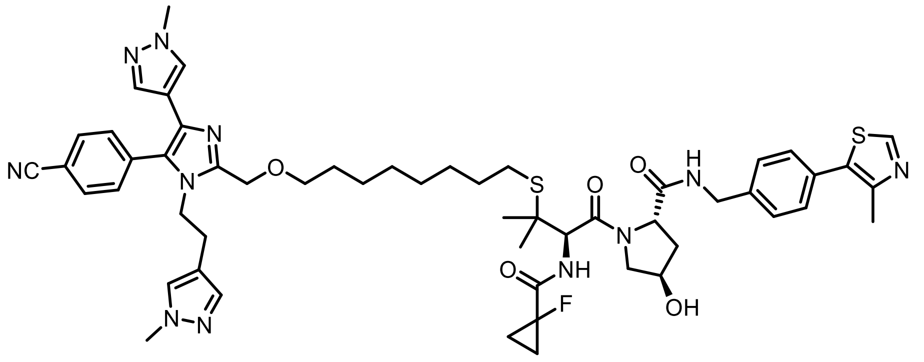

45 (5.6 mg, 20%, white solid) was prepared from BAZ2 'imidazole' warhead-tosyl intermediate **S8** (18 mg, 0.0269 mmol, 1.0 equiv) and **VHL thiol**[29] according to general procedure 13 and purified by preparative TLC (1:7:7 MeOH:DCM:EtOAC). ^{1}H NMR (500 MHz, CD_2Cl_2) δ 8.65 (s, 1H), 7.80 – 7.74 (m, 2H), 7.42 – 7.35 (m, 5H), 7.35 – 7.30 (m, 3H), 7.21 (s, 1H), 7.17 (dd, *J* = 8.5, 3.4 Hz, 1H), 6.97 (s, 1H), 6.87 (s, 1H), 4.84 (d, *J* = 7.8 Hz, 1H), 4.69 (t, *J* = 7.7 Hz, 1H), 4.55 – 4.48 (m, 3H), 4.43 (dd, *J* = 6.0, 2.4 Hz, 2H), 4.00 (dd, *J* = 8.7, 6.4 Hz, 2H), 3.90 (dt, *J* = 11.0, 2.1 Hz, 1H), 3.82 – 3.76 (m, 4H), 3.75 (s, 3H), 3.52 (t, *J* = 6.5 Hz, 2H), 2.65 – 2.59 (m, 2H), 2.54 – 2.42 (m, 5H), 2.38 – 2.30 (m, 1H), 2.24 – 2.15 (m, 1H), 1.62 – 1.54 (m, 2H), 1.47 – 1.37 (m, 2H), 1.34 – 1.22 (m, 18H), (-O*H* not detected due to exchange). ^{13}C NMR (126 MHz, CD_2Cl_2) δ 171.23, 170.26 (d, *J* = 20.4 Hz), 170.24, 150.51, 148.97, 145.62, 138.79, 138.79, 136.92, 136.03, 133.17, 131.90, 131.85, 131.82, 131.34, 129.74, 128.87, 128.29, 127.64, 126.58, 118.84, 116.97, 116.60, 112.83, 78.63 (d, *J* = 232.4 Hz), 71.23, 70.08, 65.39, 59.67, 56.77, 56.12, 48.40, 45.94, 43.13, 39.15, 39.07, 37.60, 29.93, 29.56, 29.46, 29.29, 29.26, 28.58, 26.35, 26.22, 25.97, 24.88, 16.34, 14.04

(d, J = 7.5 Hz), 13.96 (d, J = 8.0 Hz). HRMS (ESI): m/z calcd for $C_{54}H_{67}N_{11}O_5S_2F$ $[M + H]^+$: 1032.47466; found 1032.47474.

Compound 46

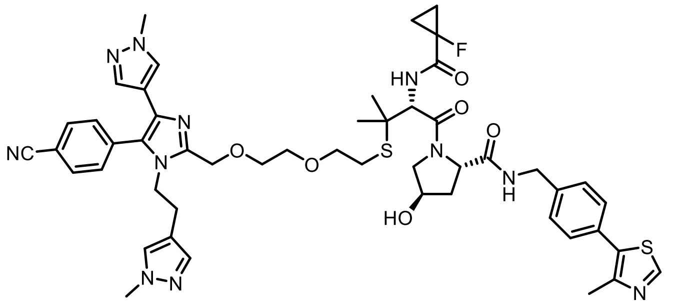

46 (8 mg, 37%, white solid) was prepared from BAZ2 'imidazole' warhead-tosyl intermediate **S9** (13.7 mg, 0.0294 mmol, 1.0 equiv) and **VHL thiol**[29] according to general procedure 13 and purified by preparative TLC (2:2:1 EtOAc:DCM:MeOH) followed by trituration in Et_2O and pentane. ^1H NMR (500 MHz, CD_2Cl_2) δ 8.65 (s, 1H), 7.77 (d, J = 8.0 Hz, 2H), 7.42 – 7.37 (m, 5H), 7.34 (d, J = 7.8 Hz, 3H), 7.19 (s, 1H), 7.15 – 7.09 (m, 1H), 6.99 (s, 1H), 6.88 (s, 1H), 4.91 (d, J = 8.7 Hz, 1H), 4.67 (dd, J = 8.7, 5.0 Hz, 1H), 4.57 (dt, J = 9.2, 4.6 Hz, 1H), 4.52 (d, J = 2.7 Hz, 2H), 4.48 (dd, J = 15.2, 6.3 Hz, 1H), 4.36 (dd, J = 15.0, 5.8 Hz, 1H), 4.03 – 3.94 (m, 3H), 3.78 (s, 3H), 3.75 (s, 3H), 3.70 – 3.54 (m, 6H), 3.47 – 3.41 (m, 1H), 2.80 – 2.69 (m, 2H), 2.66 – 2.59 (m, 2H), 2.49 (s, 3H), 2.39 (dt, J = 12.9, 5.3 Hz, 1H), 2.17 – 2.13 (m, 1H), 1.34 – 1.25 (m, 10H). ^{13}C NMR (126 MHz, CD_2Cl_2) δ 171.32, 170.14, 169.96 (d, J = 20.4 Hz), 150.49, 148.99, 145.10, 138.86, 138.83, 136.93, 135.84, 133.20, 132.00, 131.88, 131.31, 129.74, 129.02, 128.70, 128.33, 127.73, 126.66, 118.80, 116.92, 116.33, 112.97, 78.64 (d, J = 232.9 Hz), 70.80, 70.32, 70.25, 69.32, 65.50, 59.80, 56.10, 55.79, 48.32, 46.02, 43.16, 39.19, 39.08, 37.20, 29.28, 26.24, 25.99, 24.84, 16.34, 14.00 (d, J = 10.0 Hz), 13.92 (d, J = 10.0 Hz). ^{19}F NMR (377 MHz, CD_2Cl_2) δ -198.20. HRMS (ESI): m/z calcd for $C_{50}H_{59}FN_{11}O_6S_2$ $[M + H]^+$: 992.40613; found 992.40627.

dBAZ2

Synthetic route and characterization previously disclosed in Palaferri *et al.*[2]

Compound 47

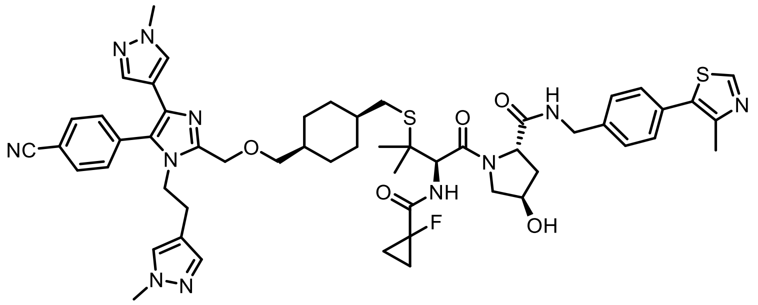

47 (6.2 mg, 26%, white solid) was prepared from BAZ2 'imidazole' warhead-tosyl intermediate **S10** (15.5 mg, 0.0232 mmol, 1.0 equiv) and **VHL thiol**[29] according to general procedure 13 with a slight modification, using 2.0 equivalents of DBU instead of 1.5 equivalents. The crude was purified by preparative TLC (2:2:1 EtOAc:DCM:MeOH) followed by trituration in Et_2O and pentane. ^1H NMR (500 MHz, CD_2Cl_2) δ 8.65 (s, 1H), 7.77 (d, *J* = 8.3 Hz, 2H), 7.44 – 7.35 (m, 3H), 7.35 – 7.30 (m, 3H), 7.23 (s, 1H), 7.14 (dd, *J* = 8.6, 3.3 Hz, 1H), 6.99 (s, 1H), 6.85 (s, 1H), 4.87 (d, *J* = 7.8 Hz, 1H), 4.68 (dd, *J* = 8.6, 5.8 Hz, 1H), 4.54 (p, *J* = 4.7 Hz, 1H), 4.50 (s, 2H), 4.44 (d, *J* = 6.2 Hz, 1H), 4.37 (dd, *J* = 15.2, 5.8 Hz, 1H), 4.00 (t, *J* = 7.5 Hz, 2H), 3.89 (dd, *J* = 10.7, 4.9 Hz, 1H), 3.80 (dd, *J* = 10.1, 3.8 Hz, 1H), 3.78 (s, 3H), 3.75 (s, 3H), 3.49 – 3.39 (m, 2H), 2.62 (dd, *J* = 8.3, 6.1 Hz, 2H), 2.50 (d, *J* = 15.9 Hz, 2H), 2.49 (s, 3H), 2.37 (dt, *J* = 13.1, 5.5 Hz, 1H), 2.18 – 2.13 (m, 1H), 1.83 – 1.73 (m, 2H), 1.52 – 1.36 (m, 7H), 1.35 – 1.27 (m, 10H), 0.96 – 0.81 (m, 2H). ^{13}C NMR (126 MHz, CD_2Cl_2) δ 170.76, 169.87, 169.73 (d, *J* = 20.4 Hz), 150.09, 148.59, 145.20, 138.38, 138.34, 136.53, 135.52, 132.79, 131.49, 131.41, 130.92, 129.36, 128.48, 127.83, 127.26, 126.20, 118.41, 116.54, 116.06, 112.48, 78.23 (d, *J* = 232.4 Hz), 73.58, 69.23, 65.38, 59.25, 55.94, 55.52, 47.75, 45.55, 42.76, 38.75, 38.66, 36.89, 35.24, 35.02, 32.96, 28.32, 27.98, 25.73, 25.59, 25.49, 25.43, 24.58, 15.91, 13.62 (d, *J* = 7.0 Hz), 13.54 (d, *J* = 6.5 Hz). HRMS (ESI): m/z calcd for $C_{54}H_{65}FN_{11}O_5S_2$ $[M + H]^+$: 1030.45901; found 1030.45995.

^{1}H, ^{13}C and ^{19}F NMR spectra

***Compound 2**. ^{1}H NMR (400 MHz, CD_2Cl_2) and ^{13}C NMR (126 MHz, CD_2Cl_2).*

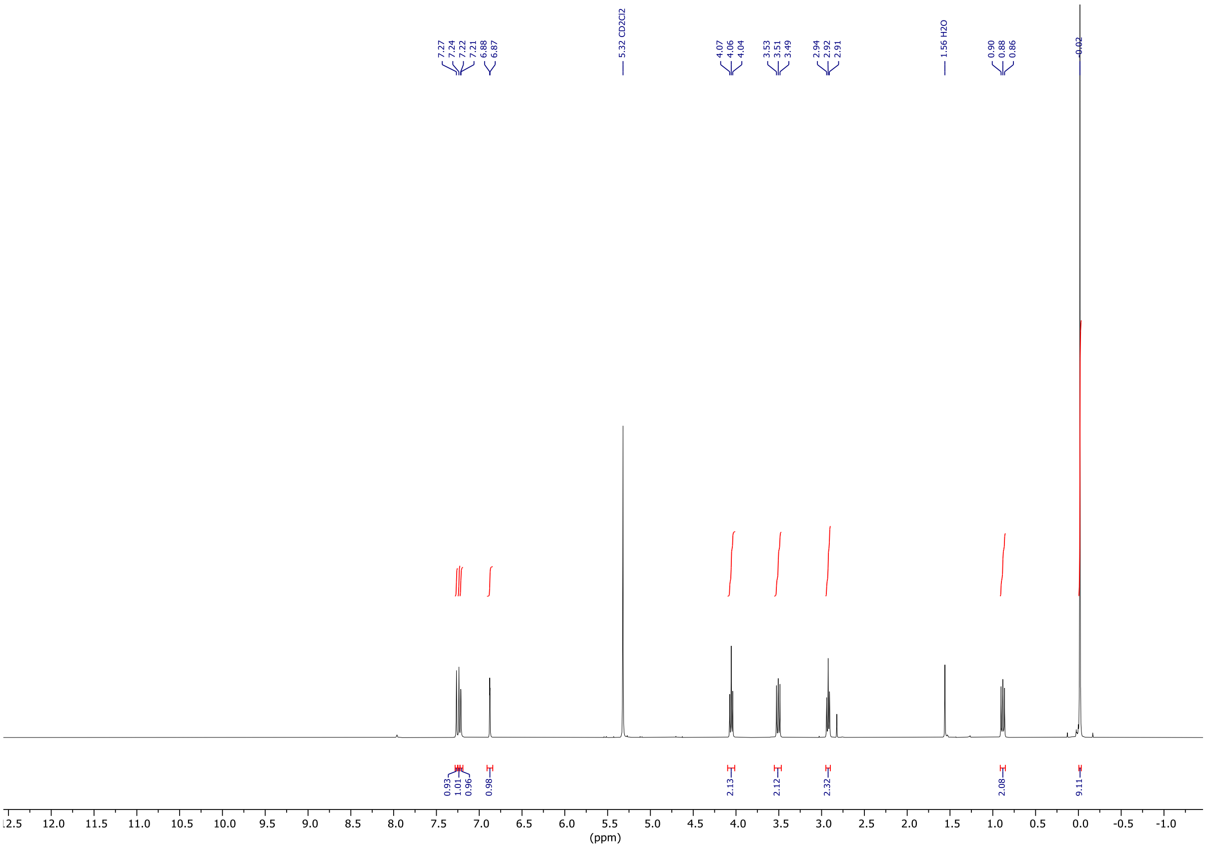

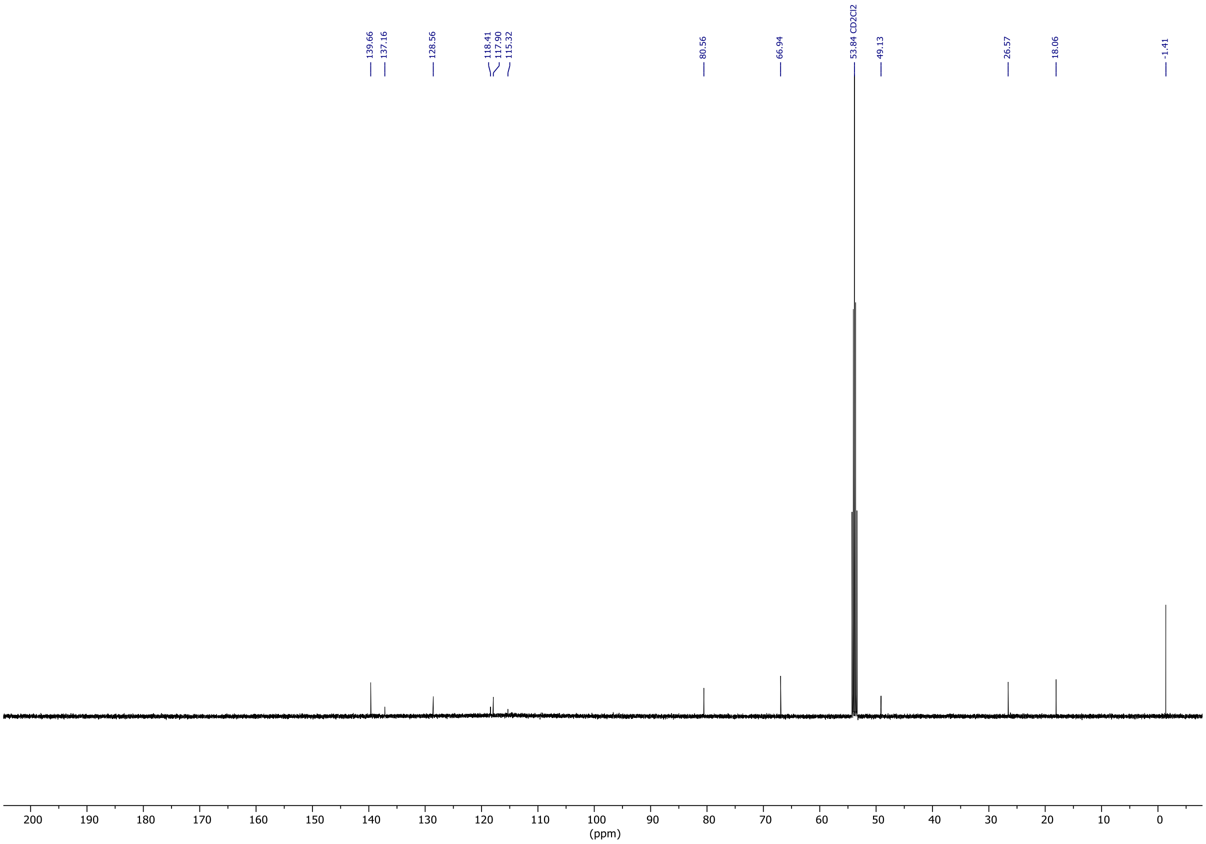

Compound 3. *^{1}H NMR (400 MHz, CD_2Cl_2) and ^{13}C NMR (126 MHz, CD_2Cl_2).*

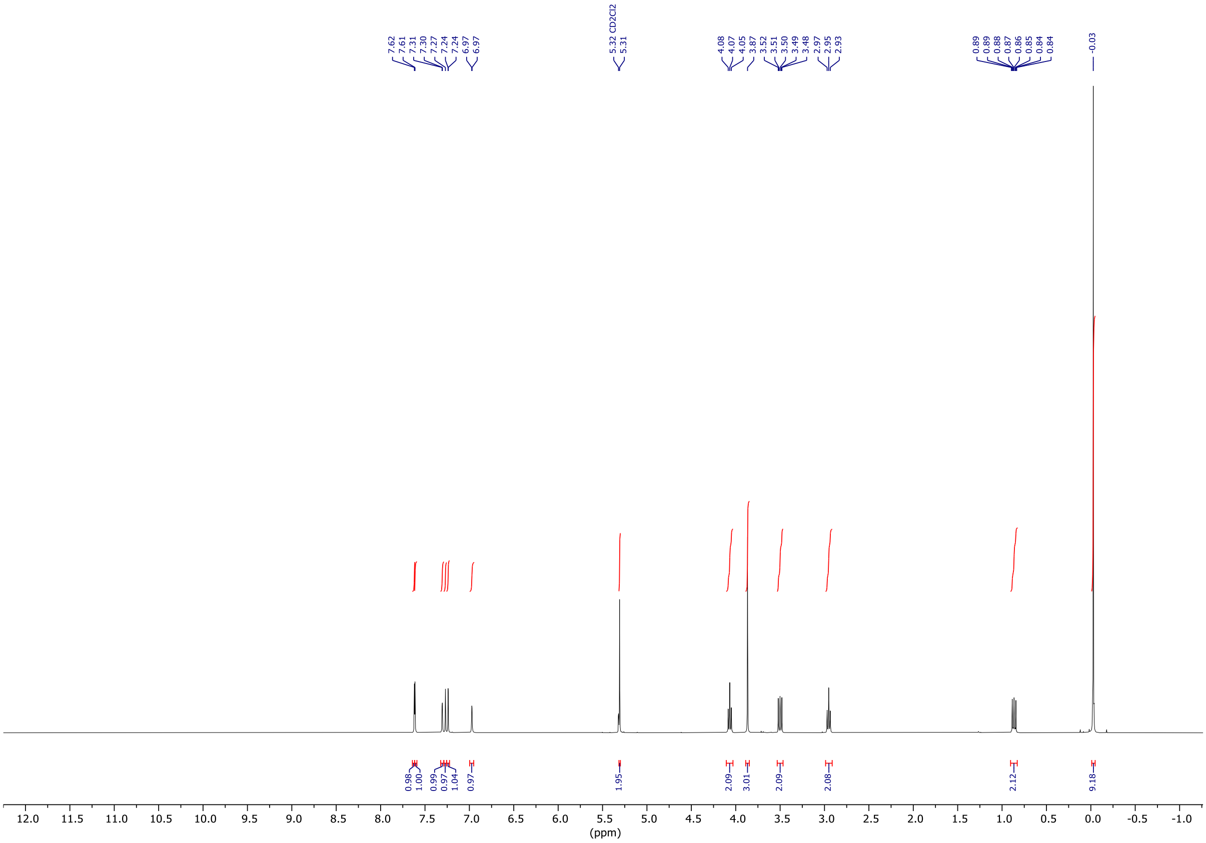

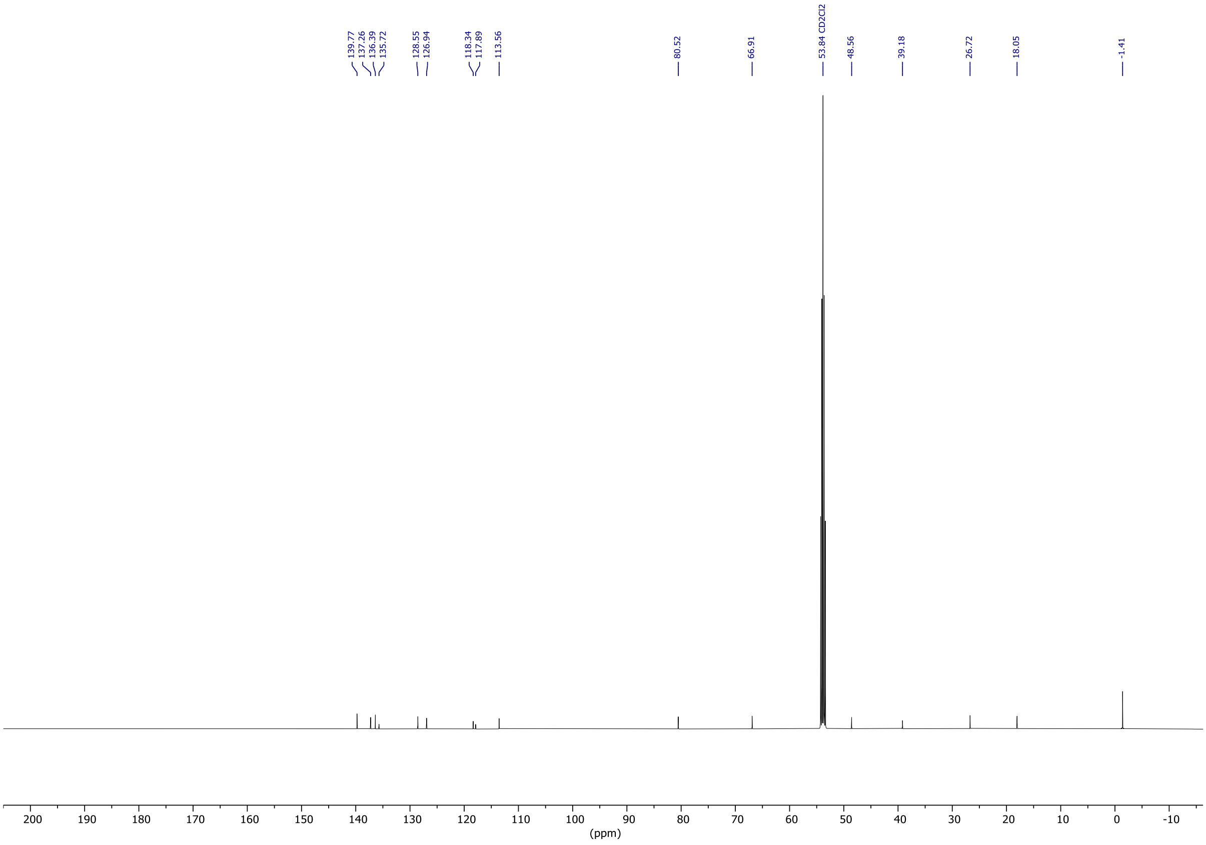

Compound 4. *^{1}H NMR (500 MHz, CD_2Cl_2) and ^{13}C NMR (126 MHz, CD_2Cl_2).*

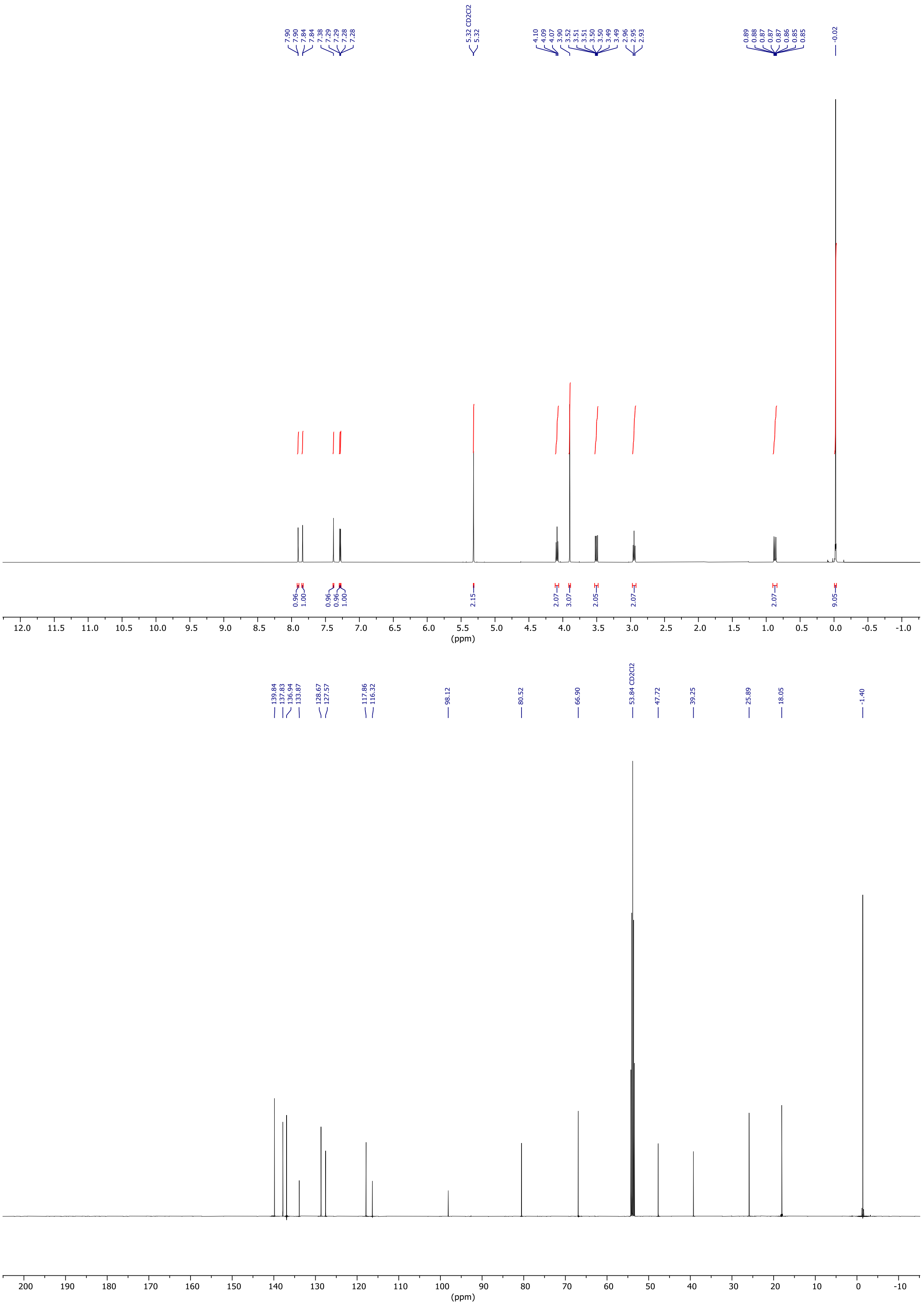

Compound 5. *^{1}H NMR (500 MHz, CD_2Cl_2) and ^{13}C NMR (126 MHz, CD_2Cl_2).*

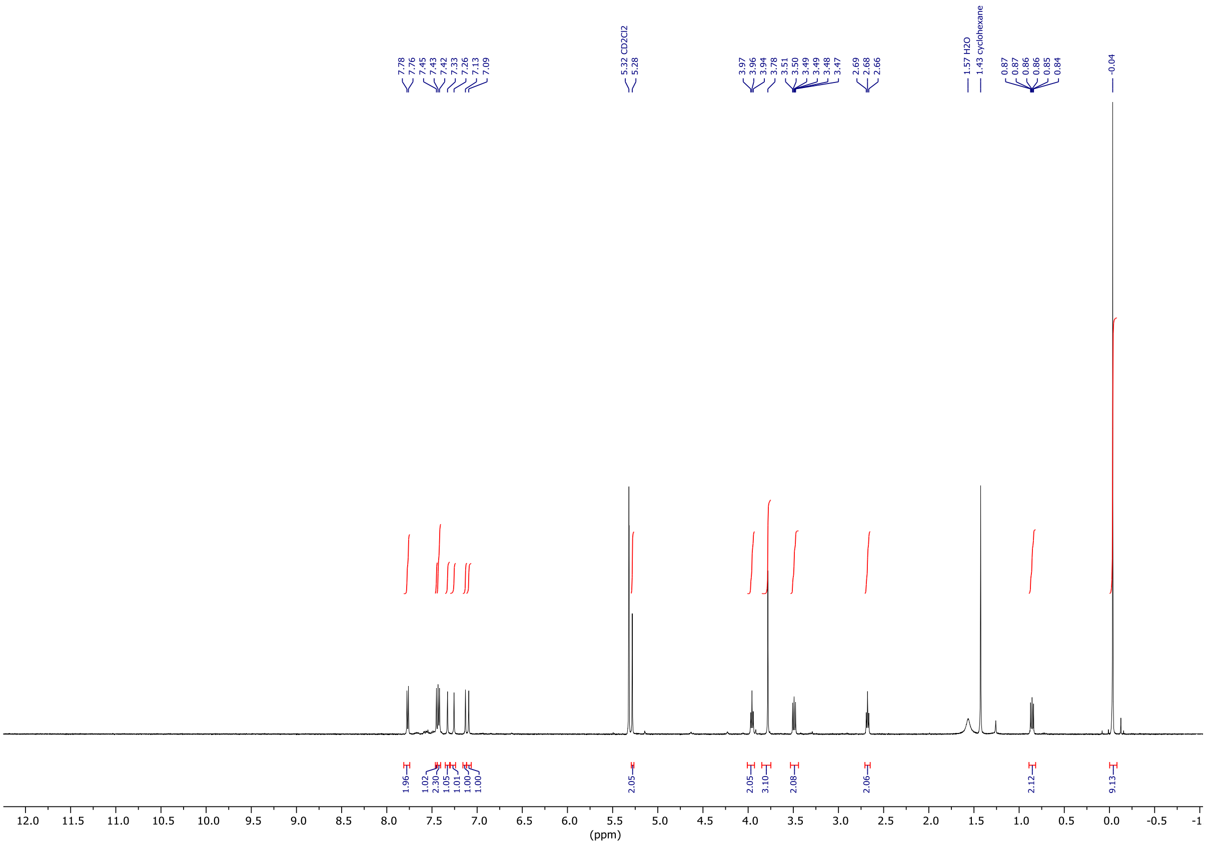

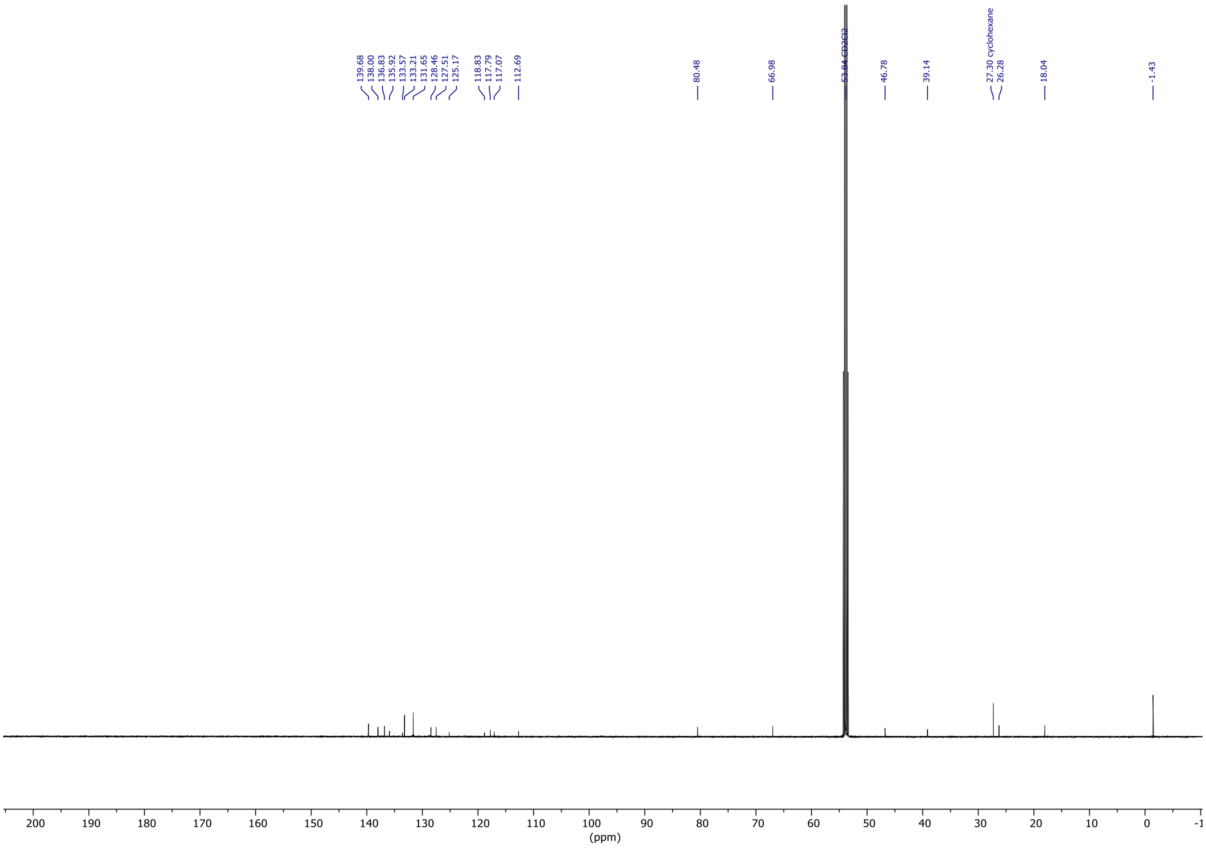

BAZ2 'pyrazole' warhead. *^{1}H NMR (500 MHz, CD_2Cl_2) and ^{13}C NMR (126 MHz, CD_2Cl_2).*

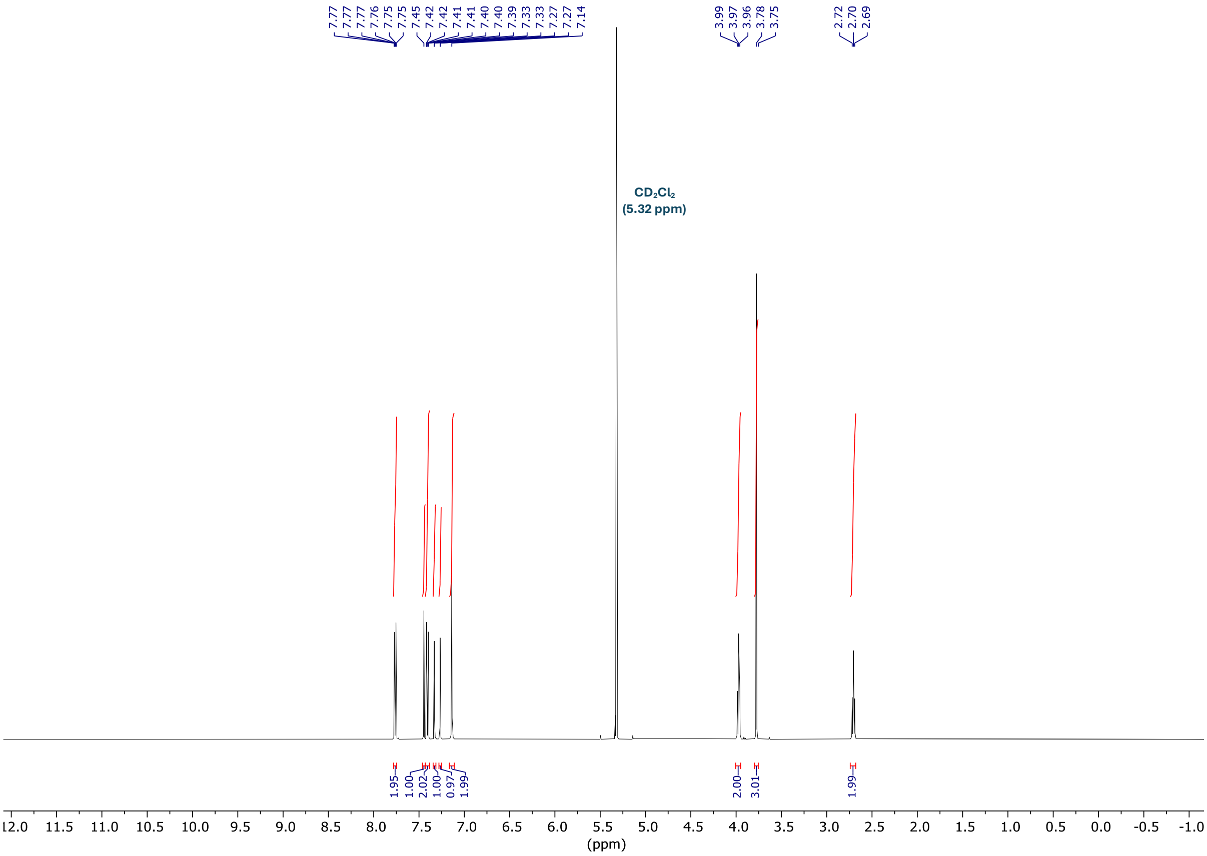

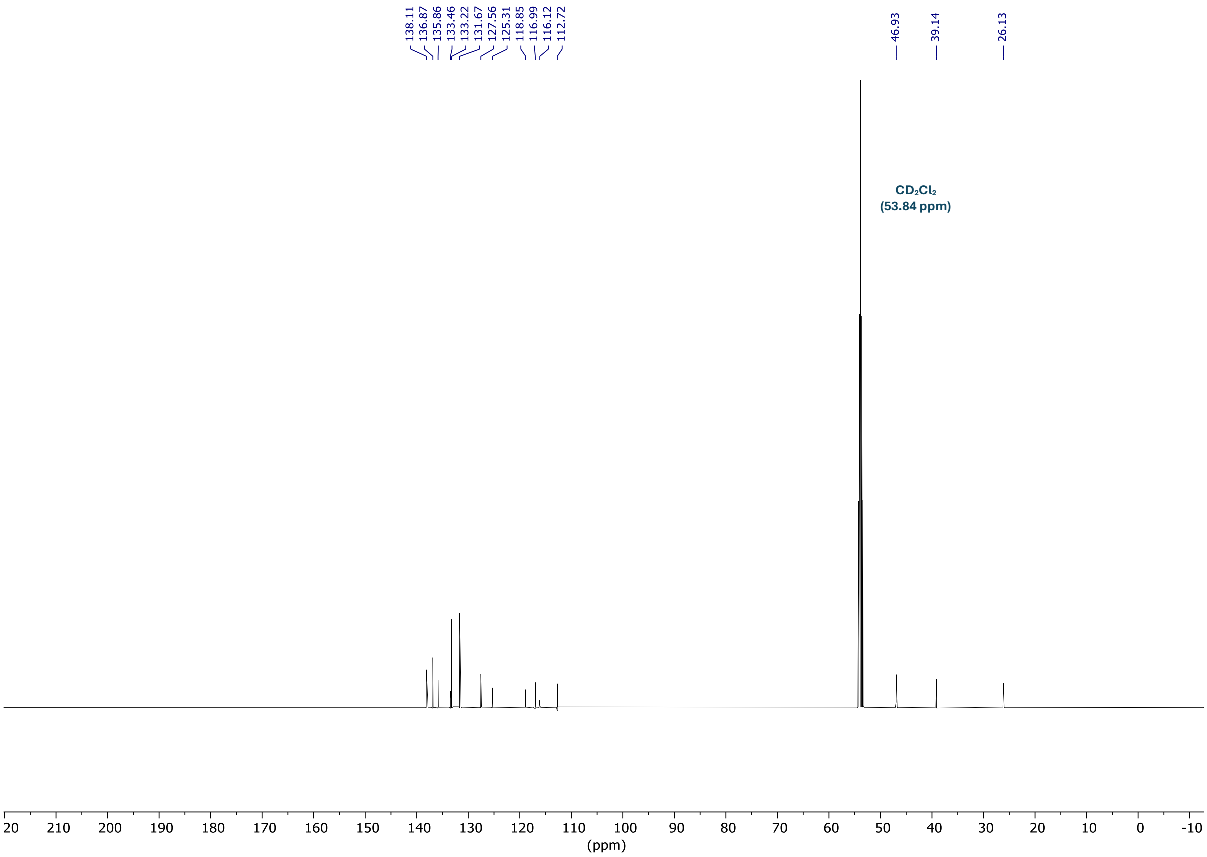

Compound S1. *^{1}H NMR (400 MHz, CD_2Cl_2) and ^{13}C NMR (101 MHz, CD_2Cl_2).*

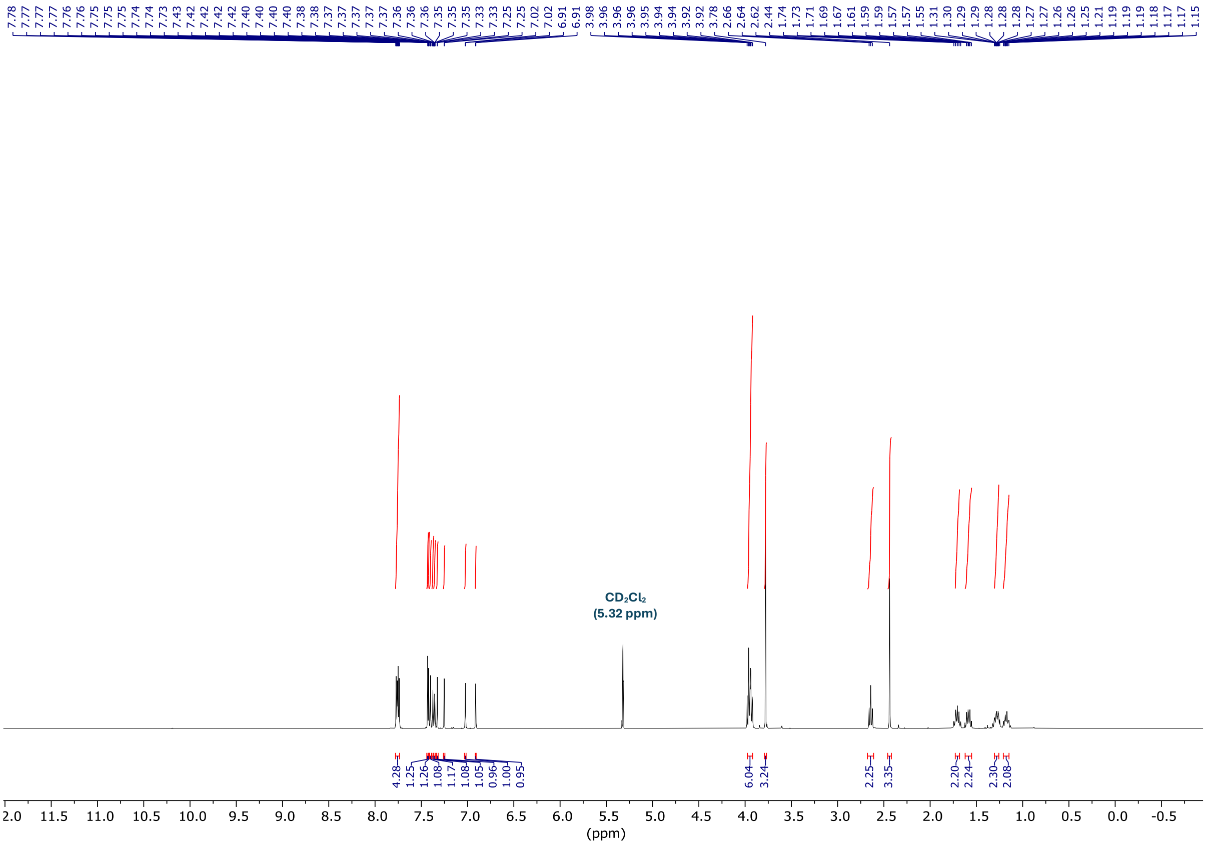

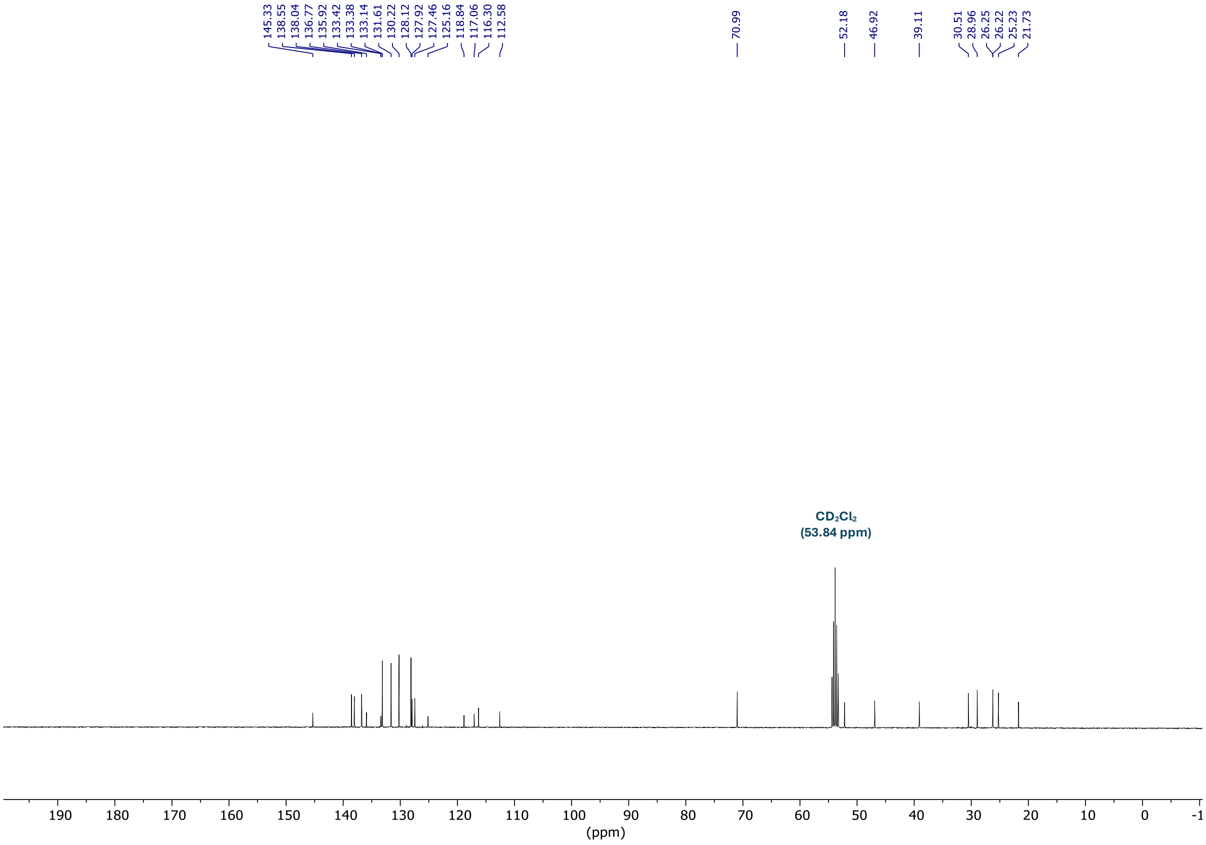

Compound S2. *^{1}H NMR (400 MHz, CD_2Cl_2) and ^{13}C NMR (101 MHz, CD_2Cl_2)*

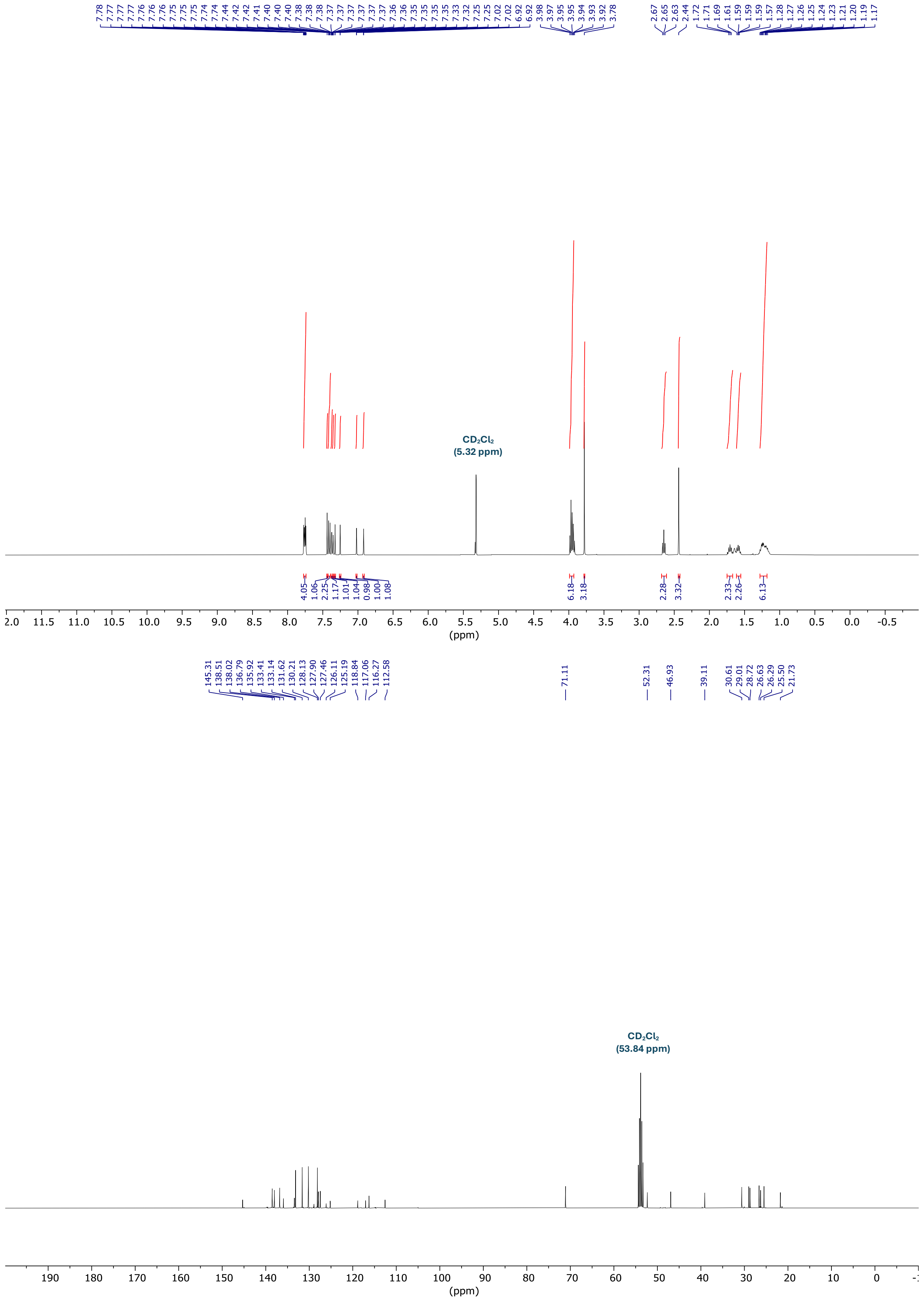

Compound S3. *^{1}H NMR (400 MHz, CD_2Cl_2) and ^{13}C NMR (126 MHz, CD_2Cl_2).*

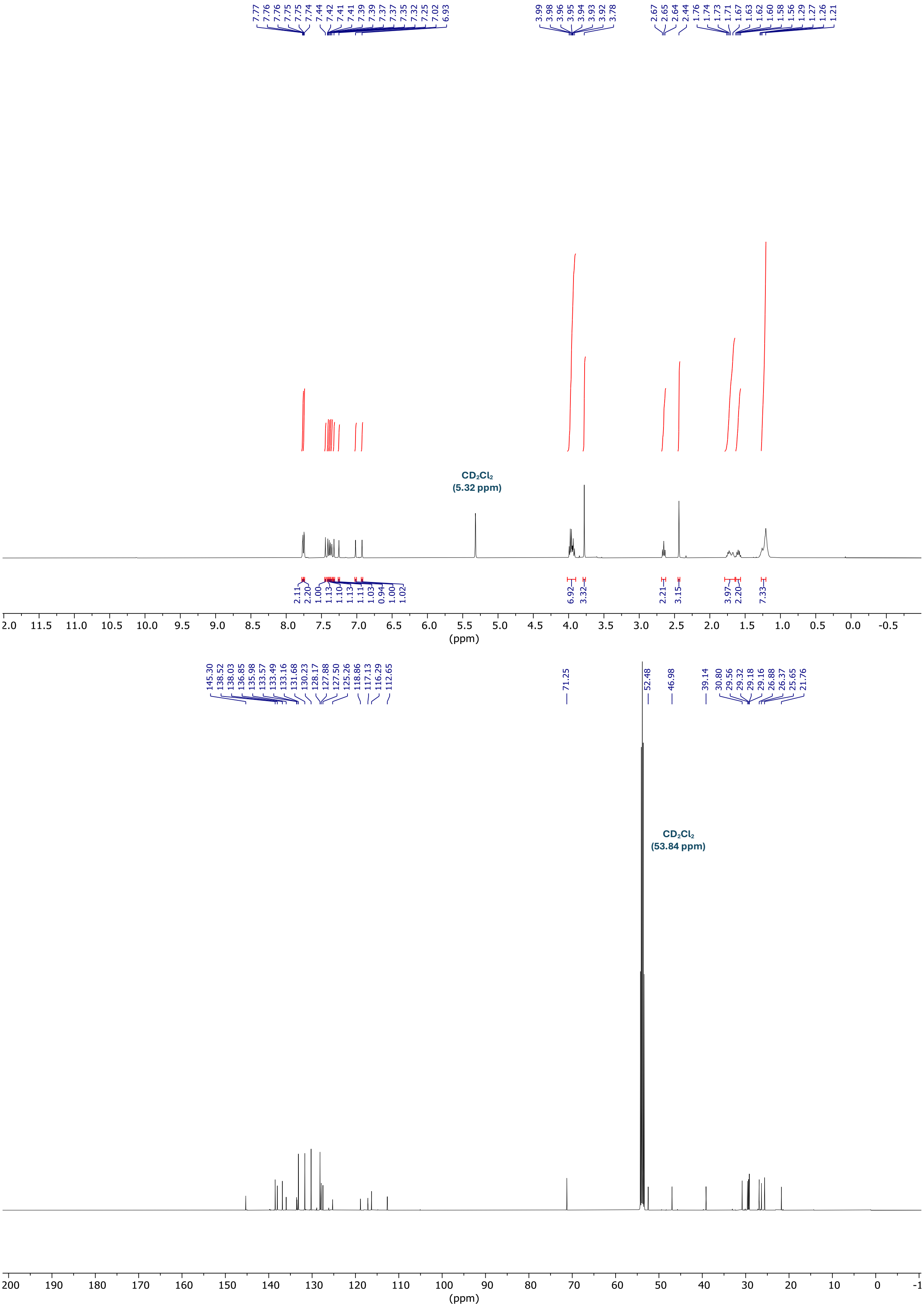

Compound S4. *^{1}H NMR (400 MHz, CD_2Cl_2) and ^{13}C NMR (126 MHz, CD_2Cl_2).*

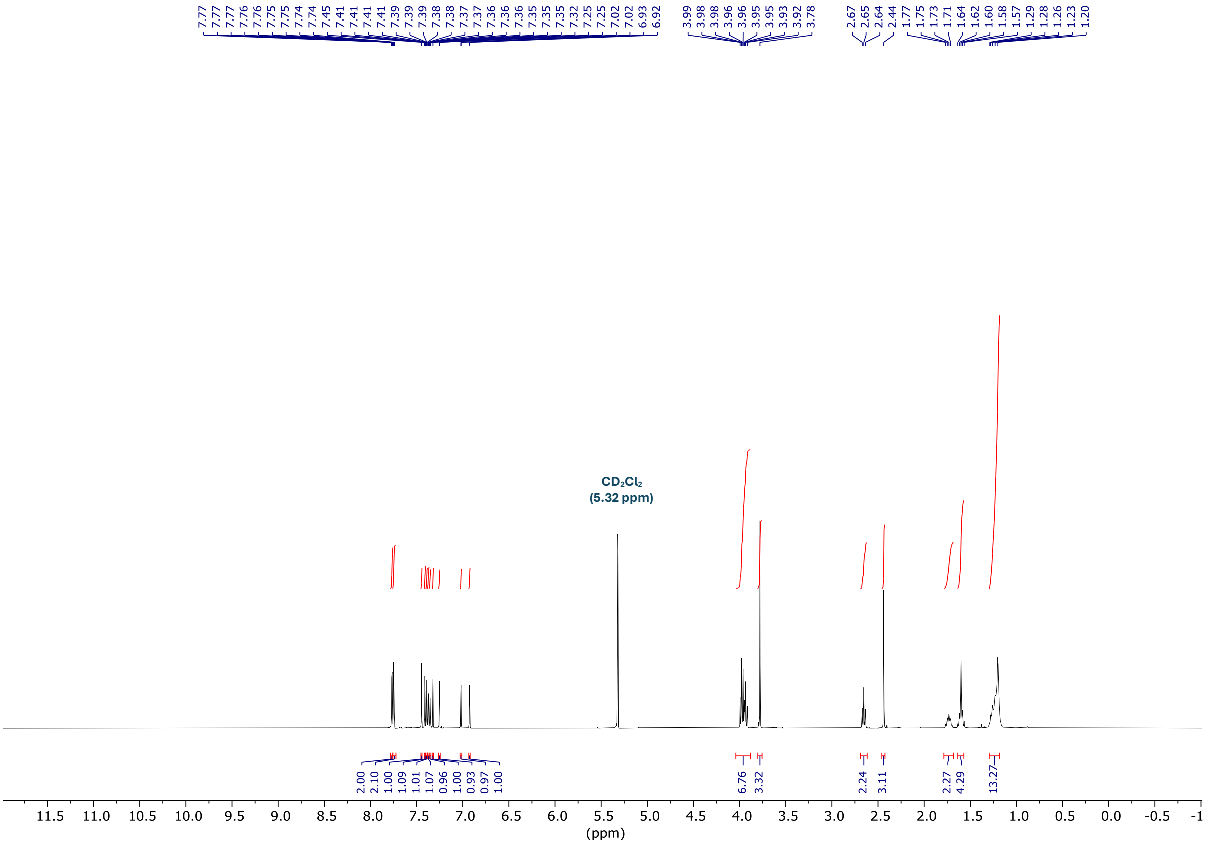

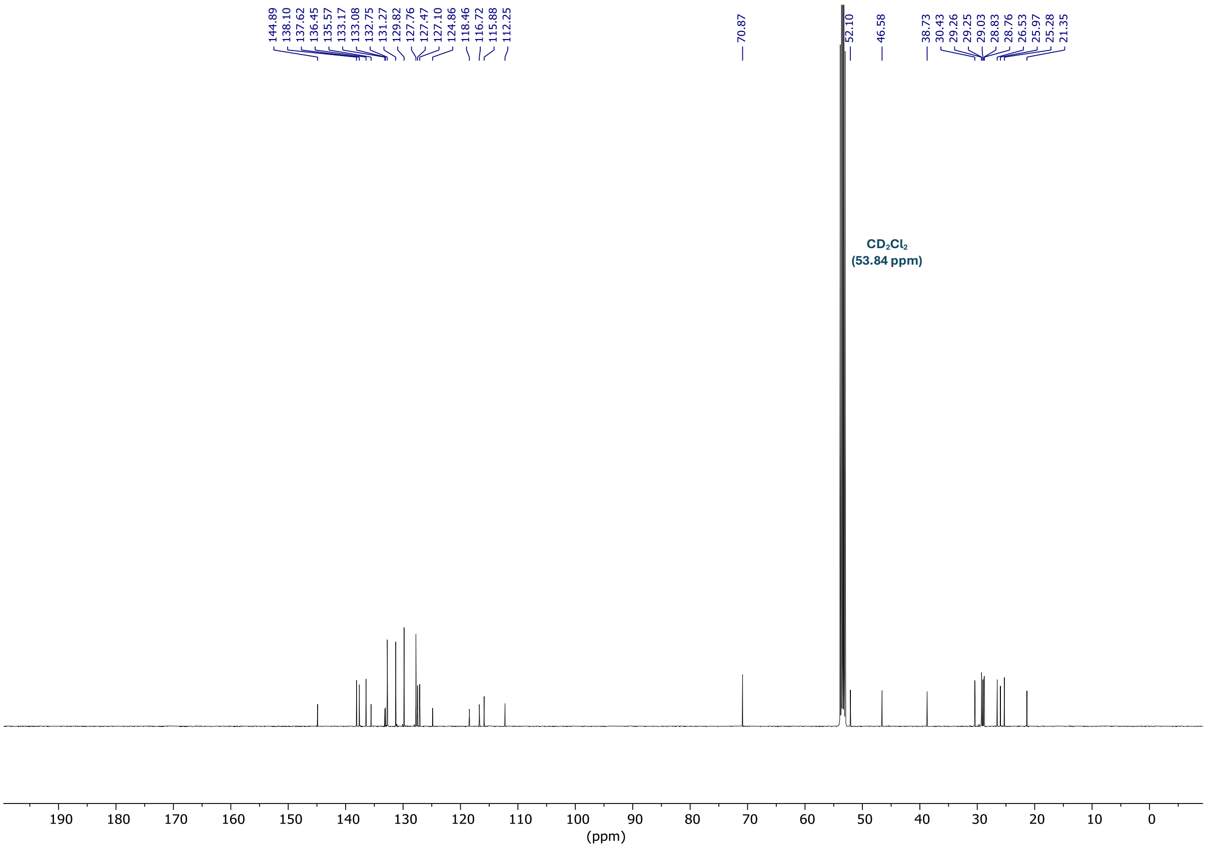

Compound S5. *^{1}H NMR (500 MHz, CD_2Cl_2) and ^{13}C NMR (126 MHz, CD_2Cl_2).*

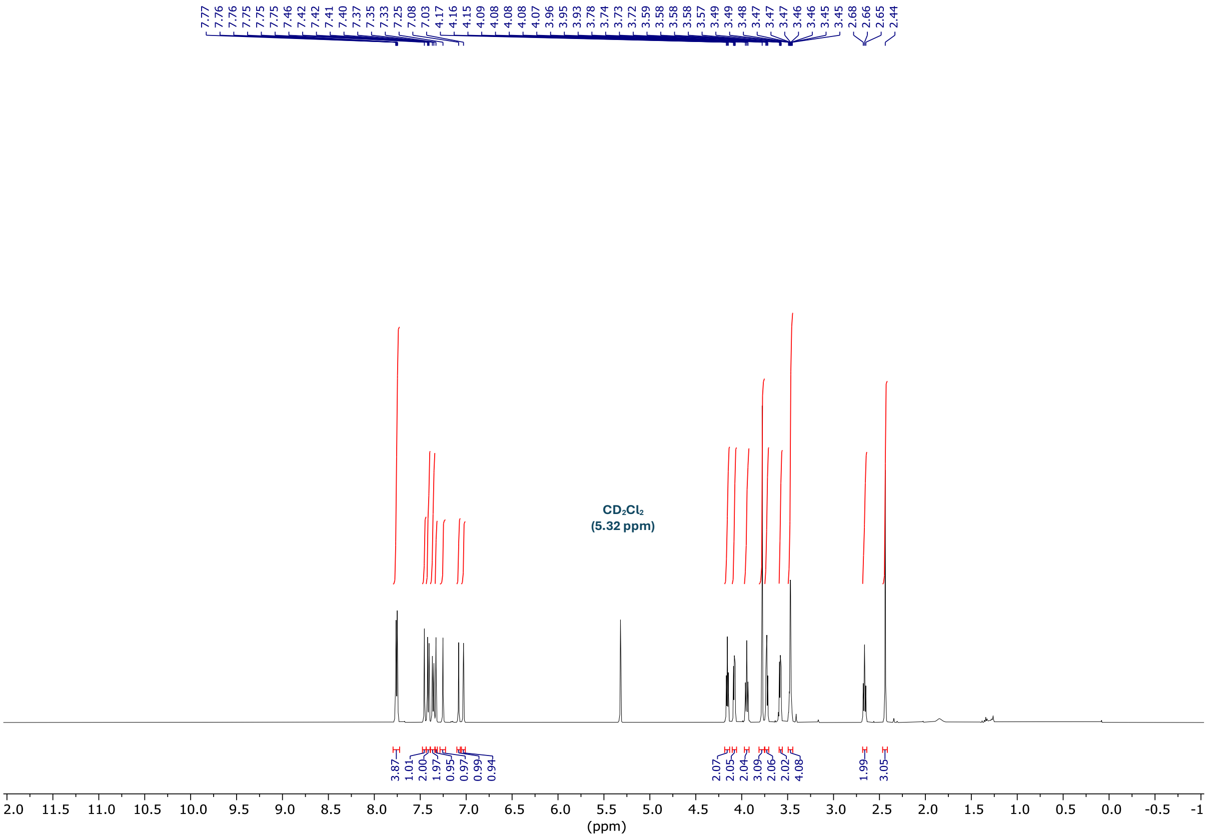

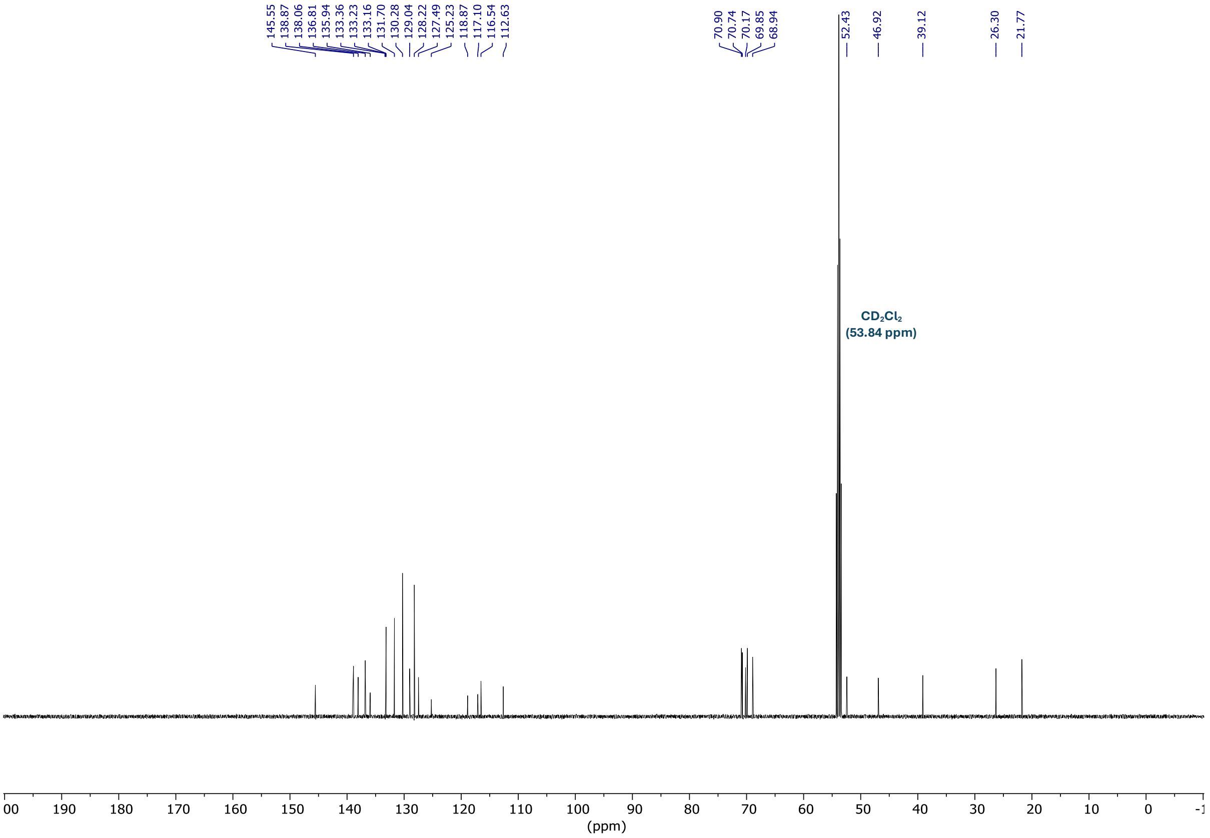

Compound S6. ^{1}H NMR (500 MHz, CD_2Cl_2) and ^{13}C NMR (126 MHz, CD_2Cl_2).

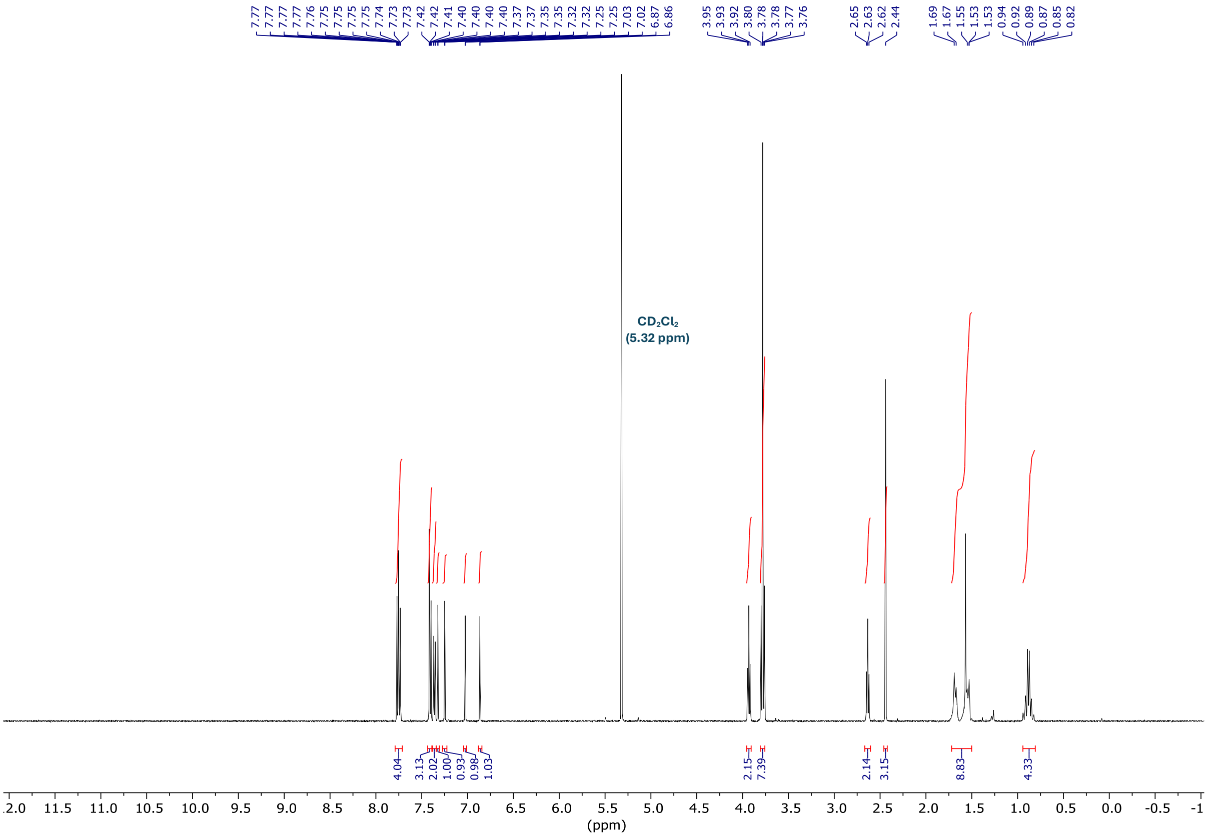

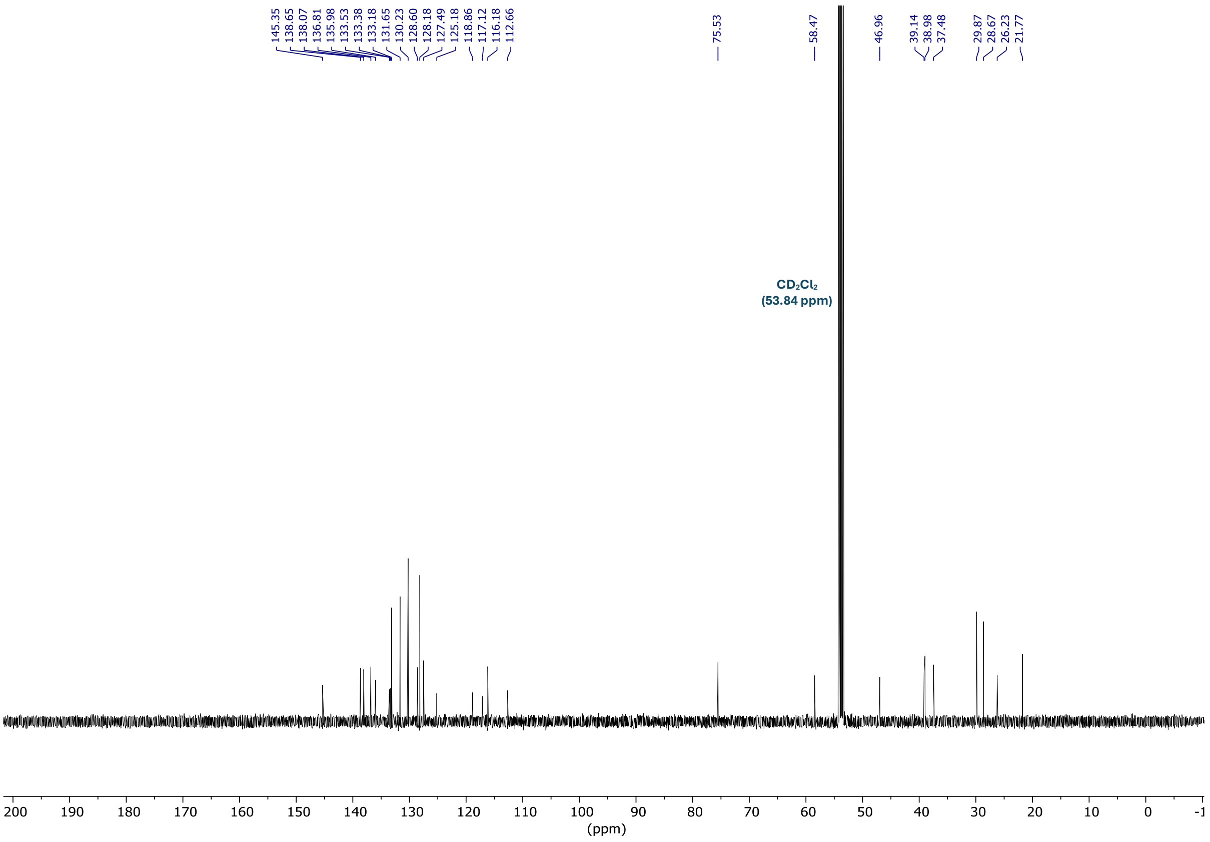

Compound S7. *^{1}H NMR (400 MHz, CD_2Cl_2) and ^{13}C NMR (126 MHz, CD_2Cl_2).*

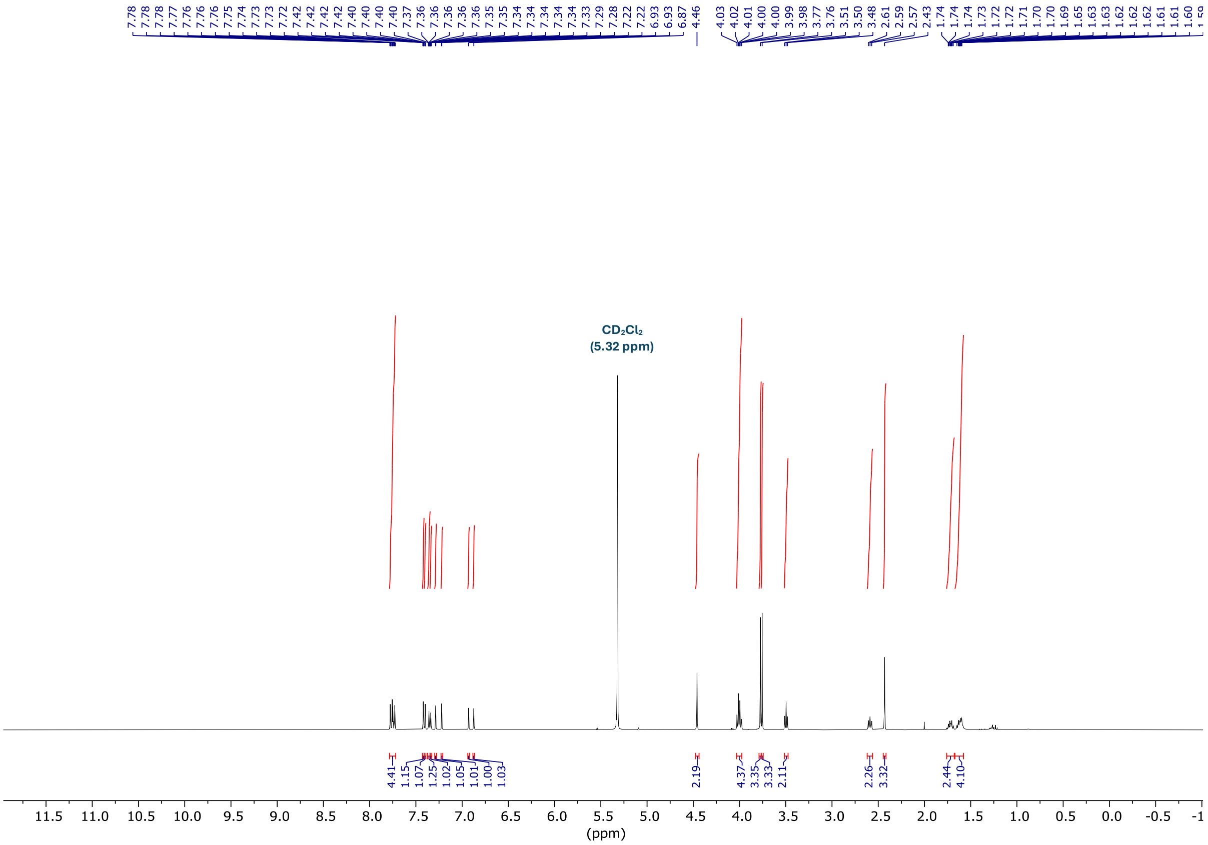

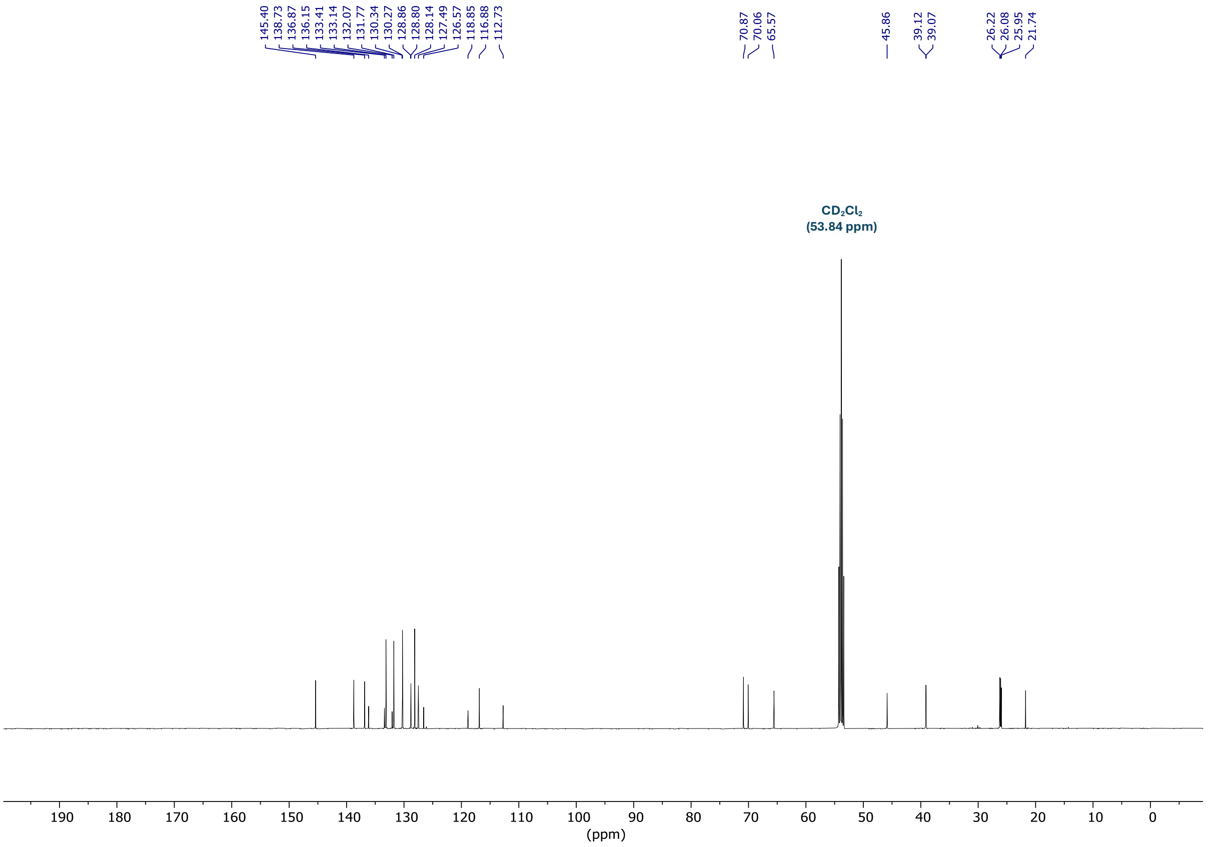

Compound S8. *^{1}H NMR (500 MHz, CD_2Cl_2) and ^{13}C NMR (126 MHz, CD_2Cl_2).*

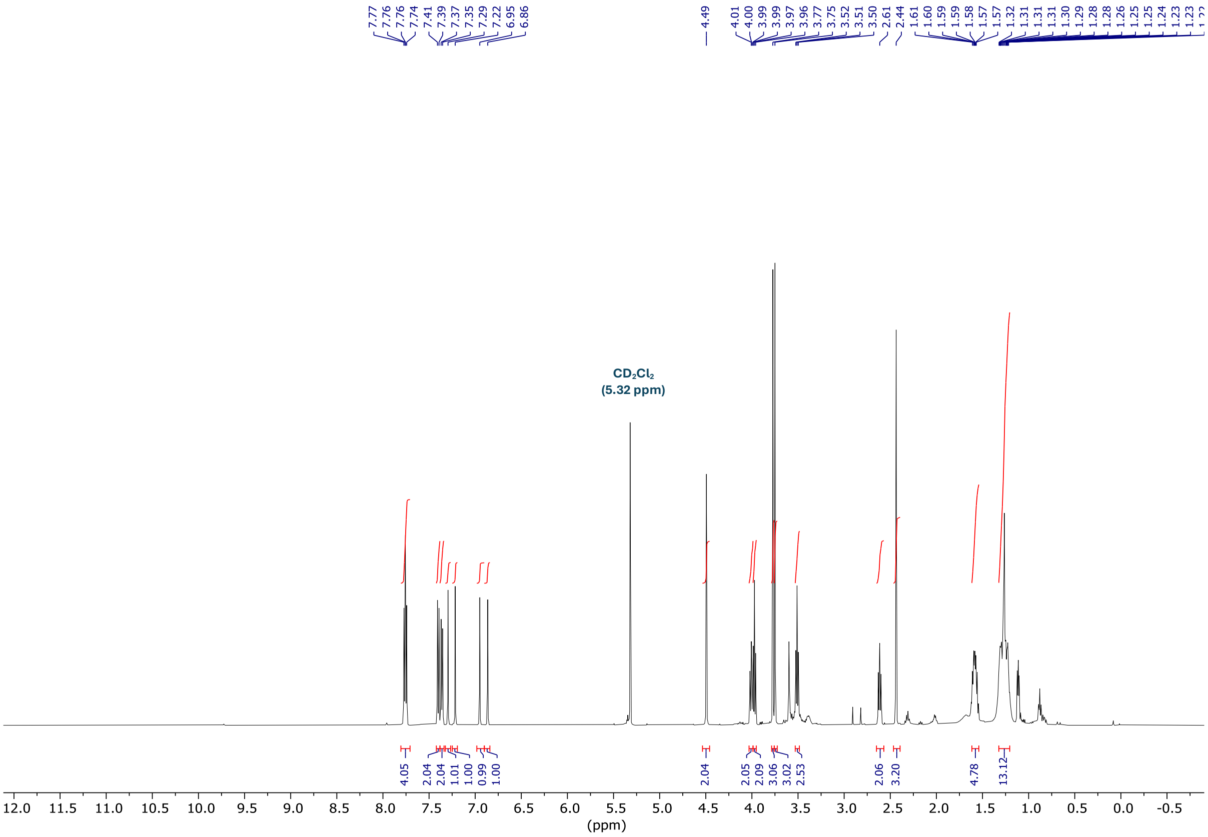

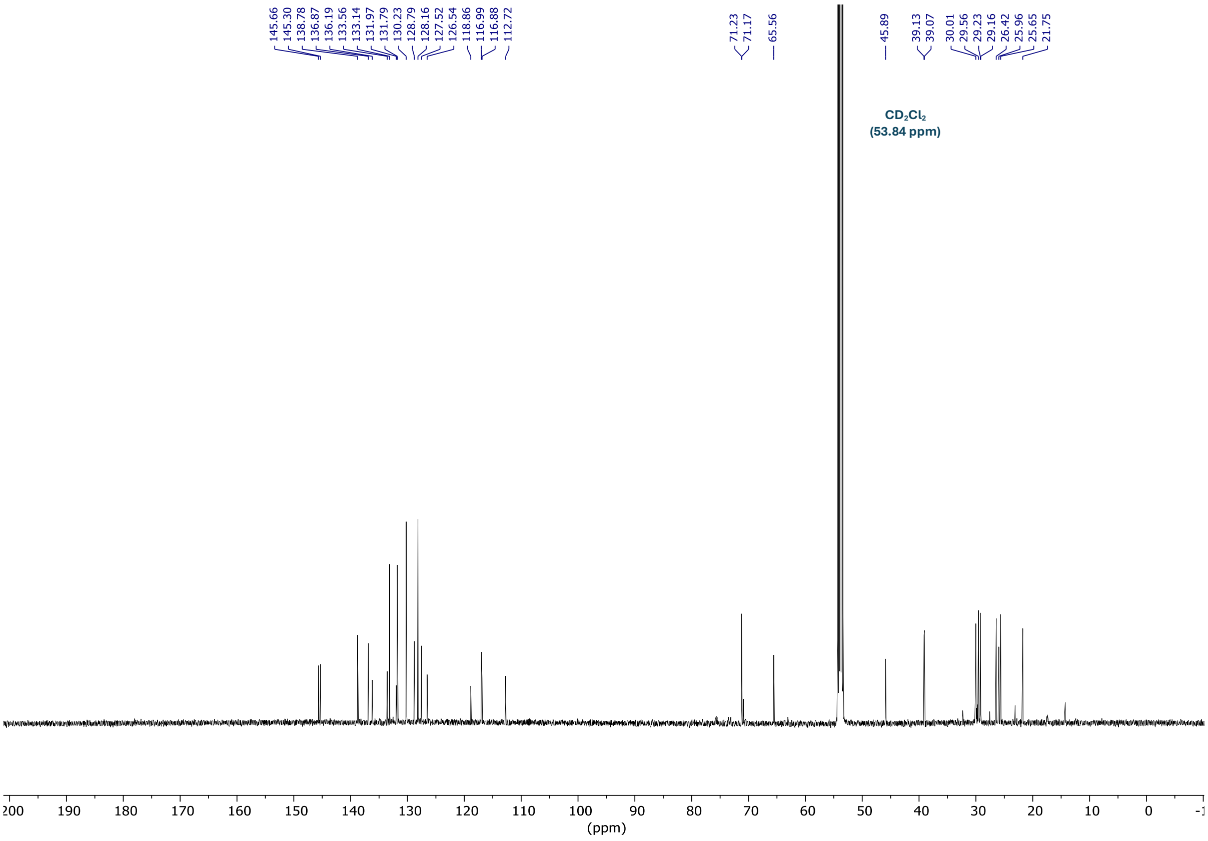

Compound S9. *^{1}H NMR (400 MHz, CD_2Cl_2) and ^{13}C NMR (126 MHz, CD_2Cl_2).*

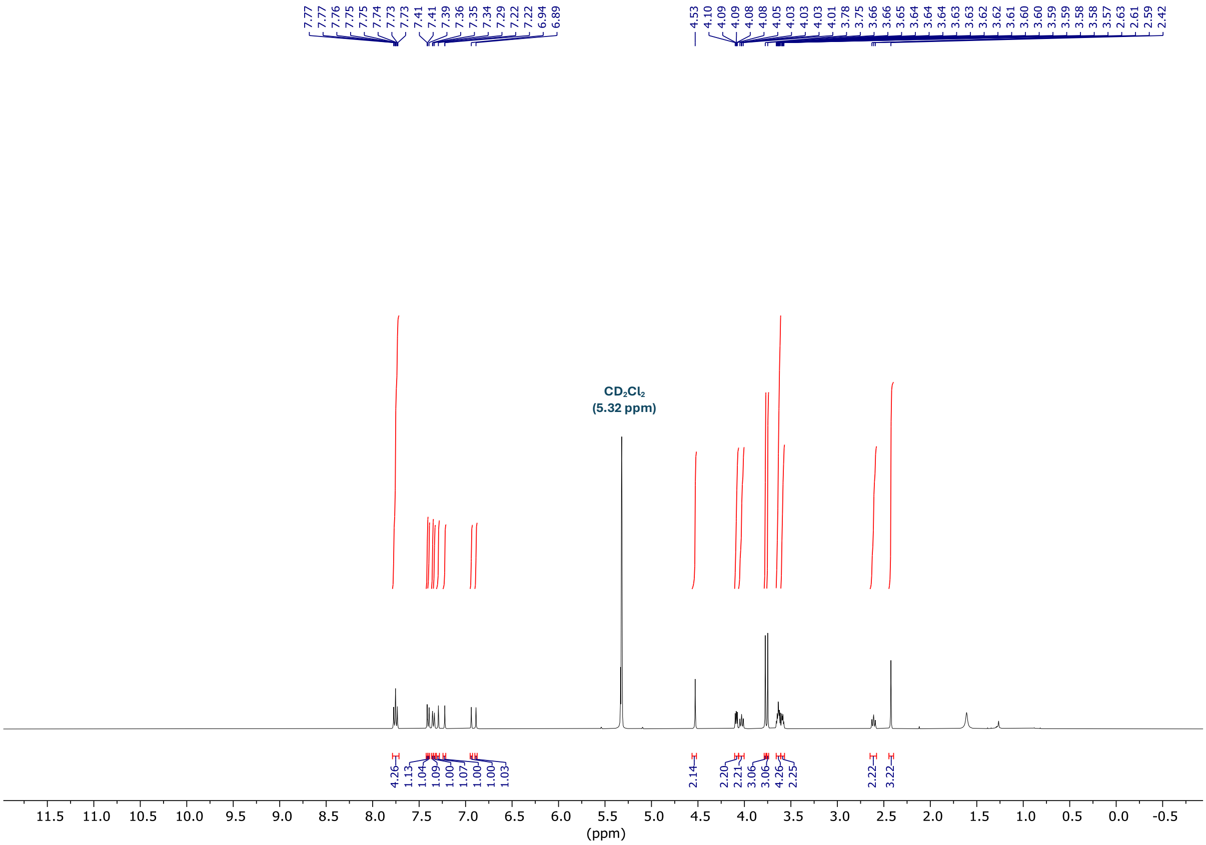

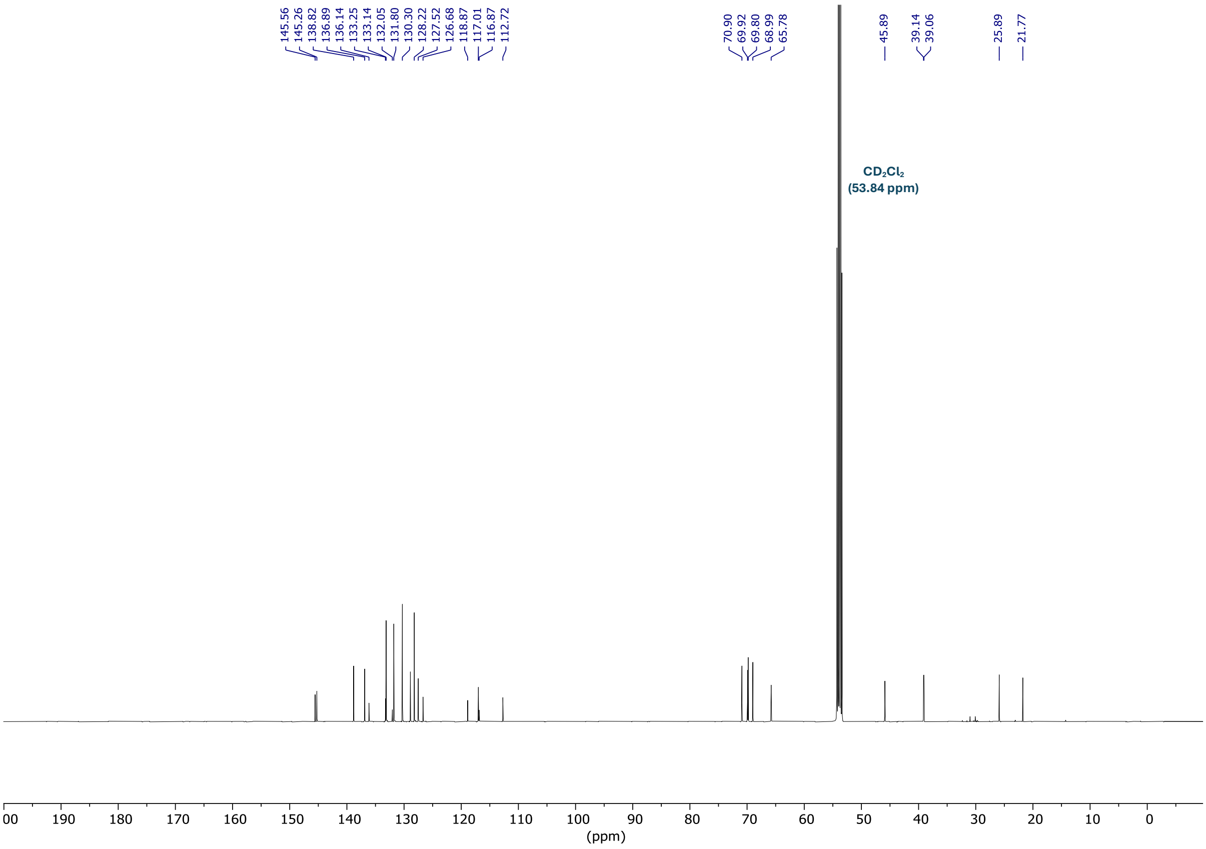

Compound S10. *^{1}H NMR (400 MHz, CD_2Cl_2) and ^{13}C NMR (126 MHz, CD_2Cl_2).*

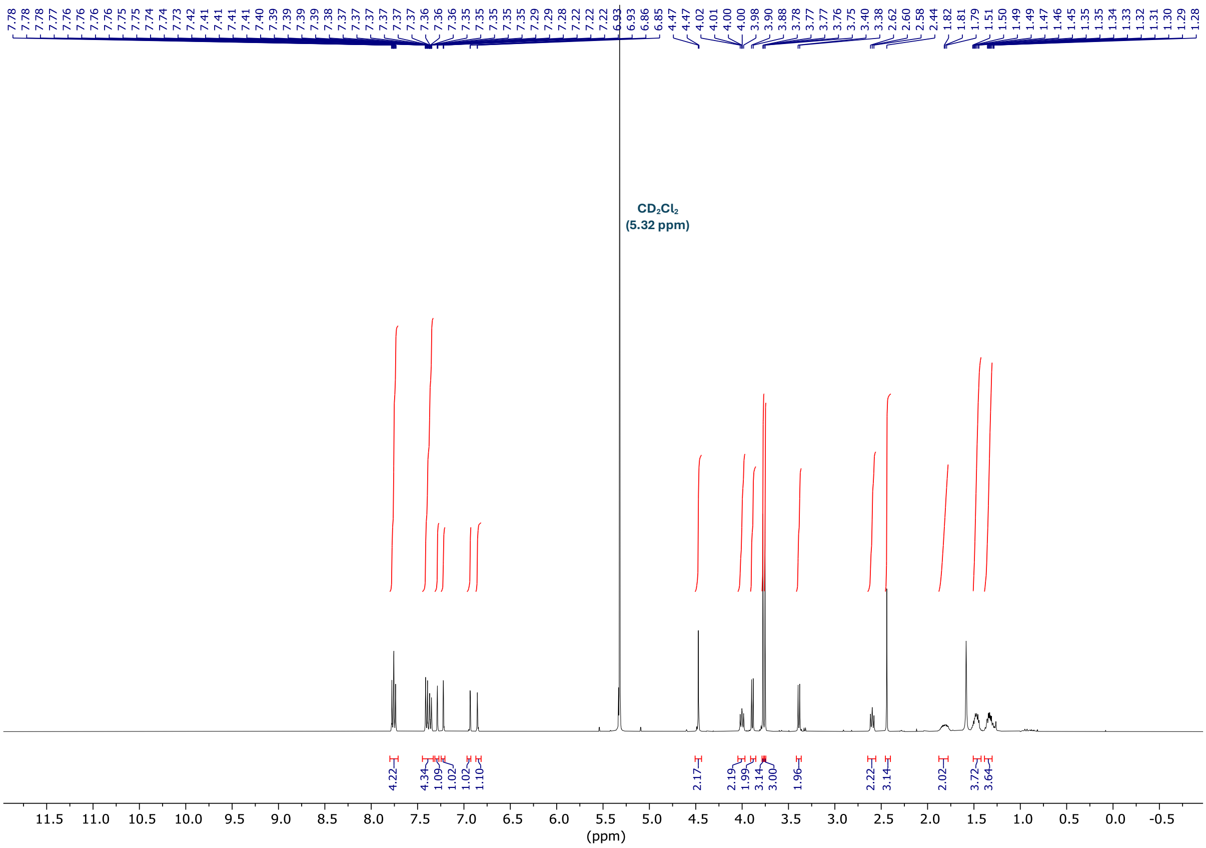

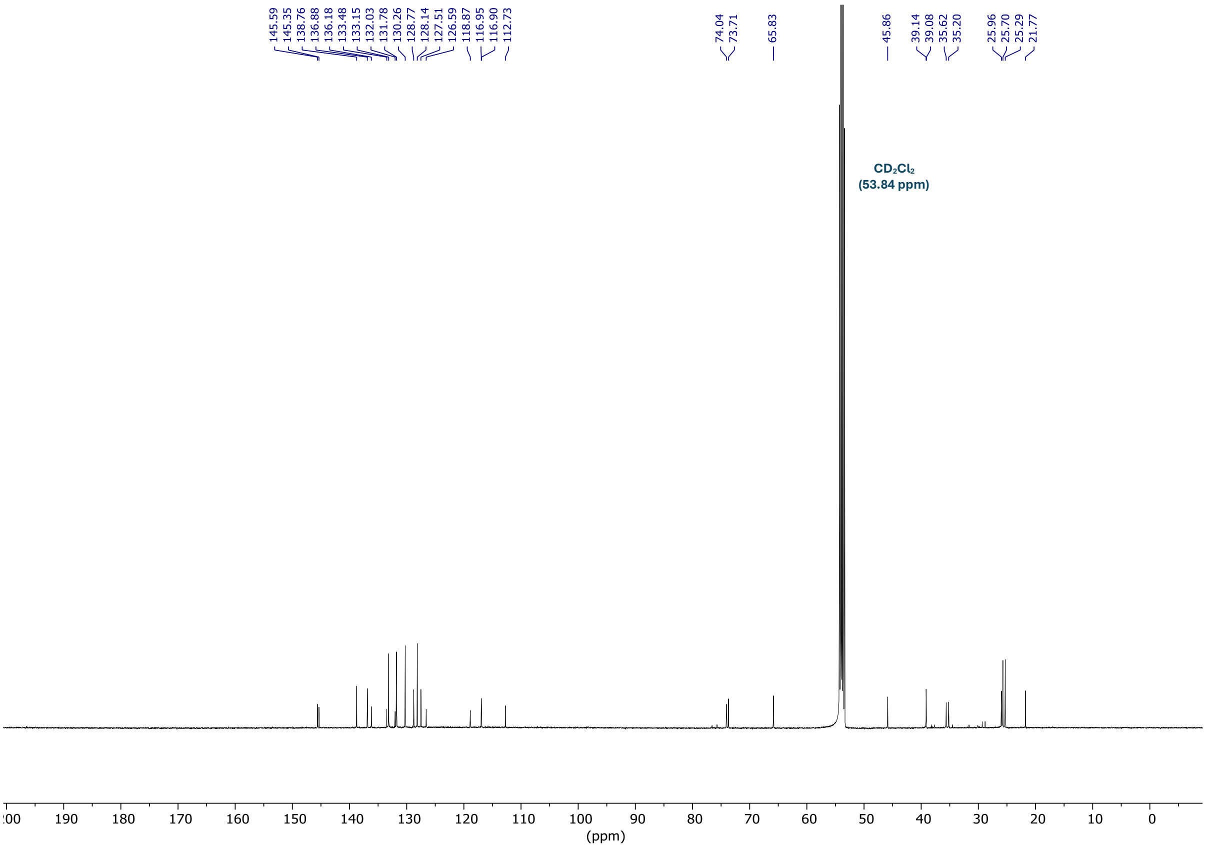

Compound S11. *^{1}H NMR (500 MHz, CD_2Cl_2), ^{13}C NMR (126 MHz, CD_2Cl_2).*

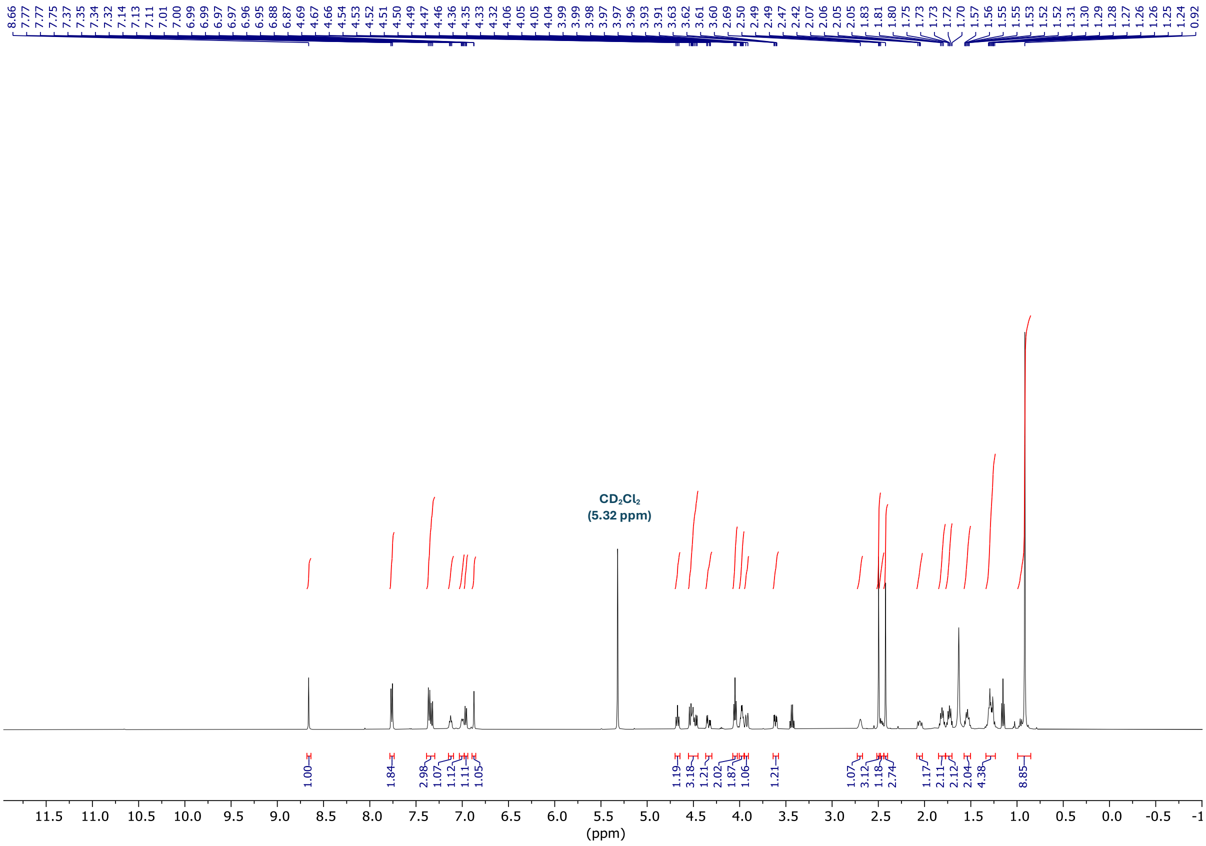

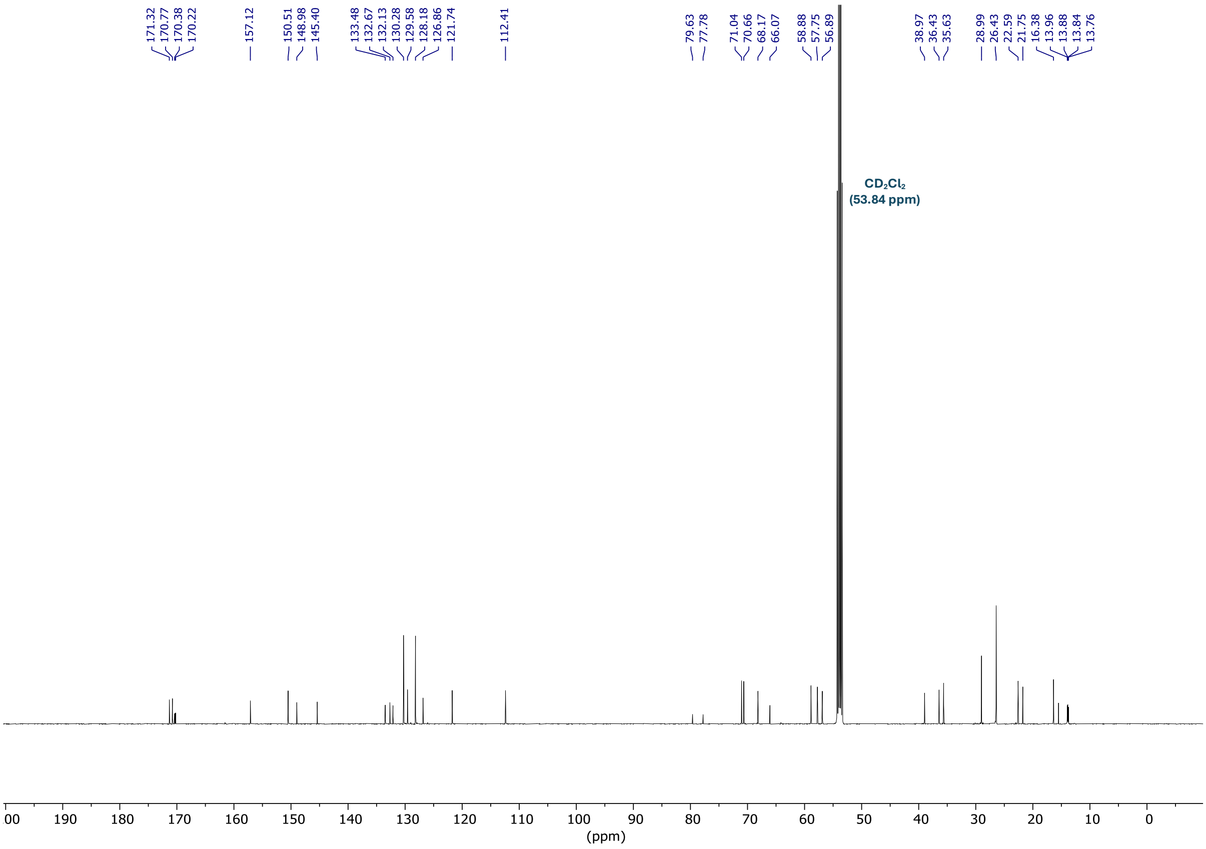

Compound S12. *^{1}H NMR (500 MHz, CD_2Cl_2), ^{13}C NMR (126 MHz, CD_2Cl_2).*

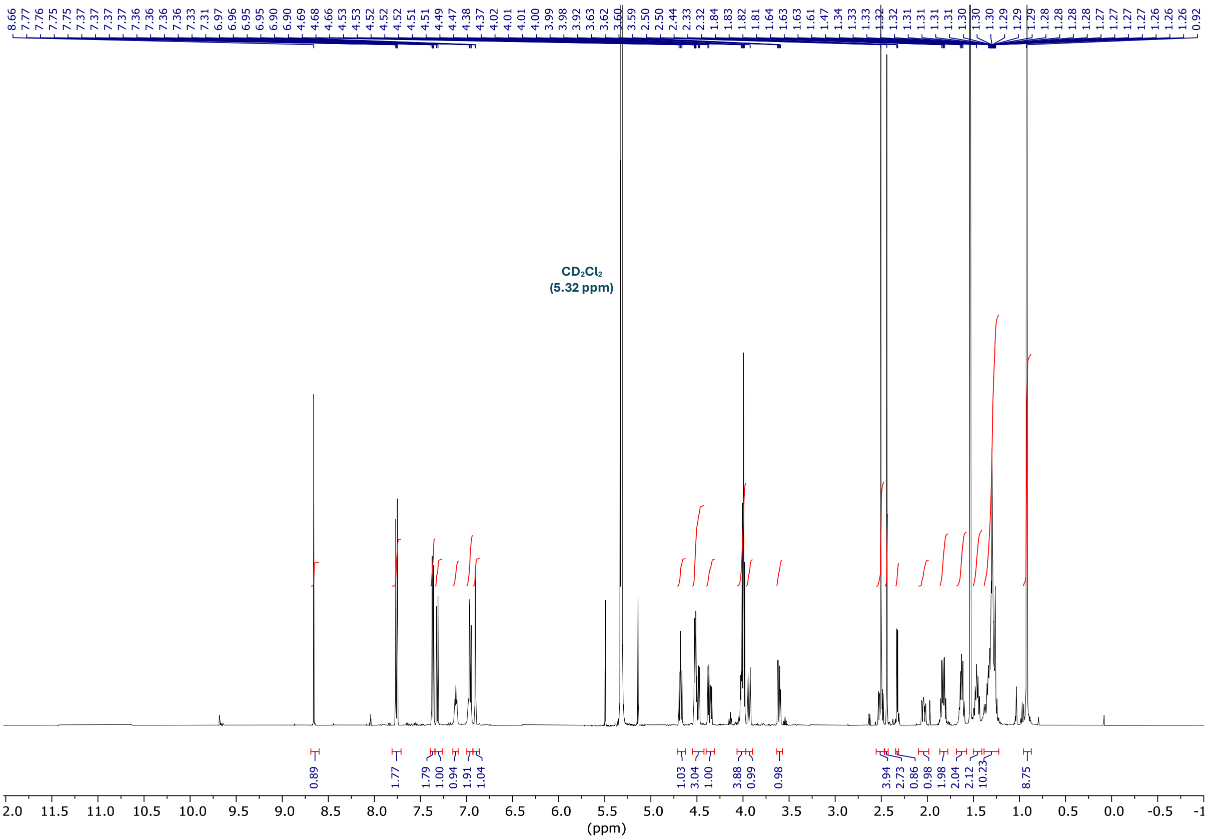

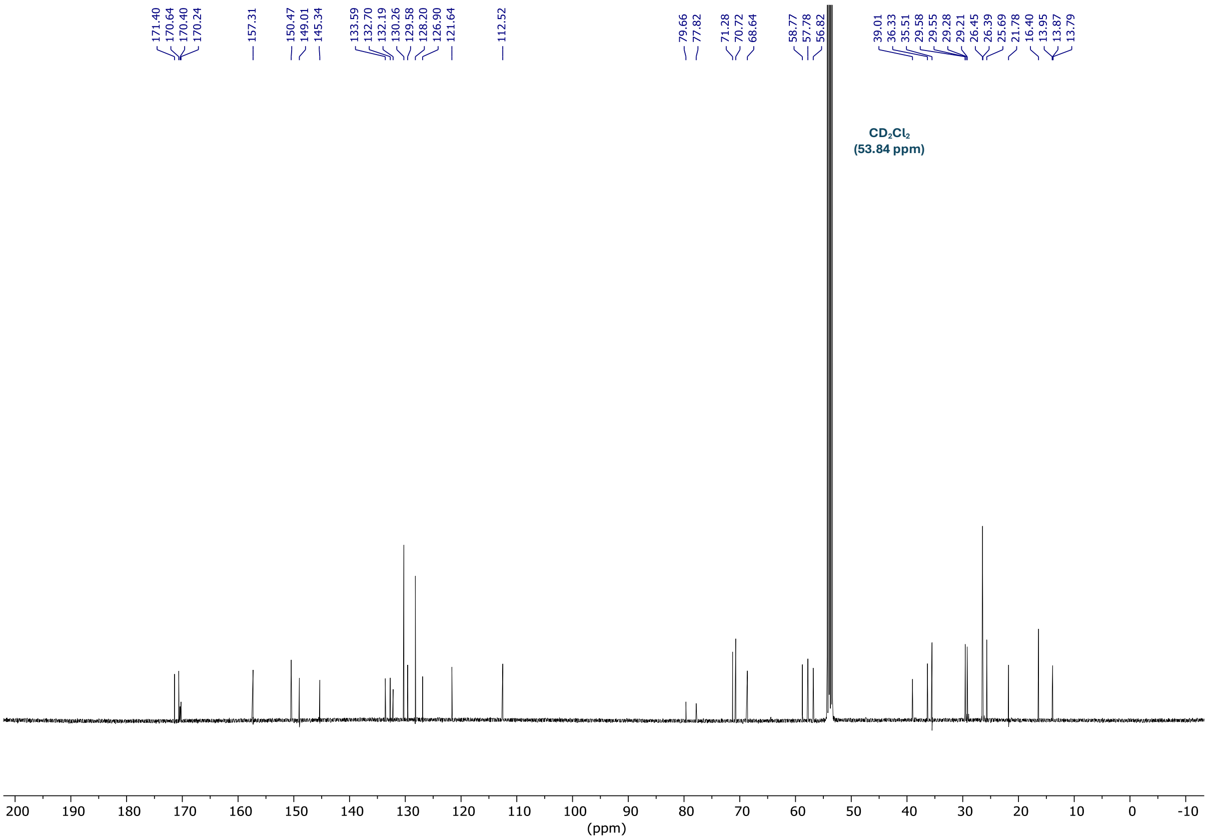

Compound S13. *^{1}H NMR (500 MHz, CD_2Cl_2) and ^{13}C NMR (126 MHz, CD_2Cl_2).*

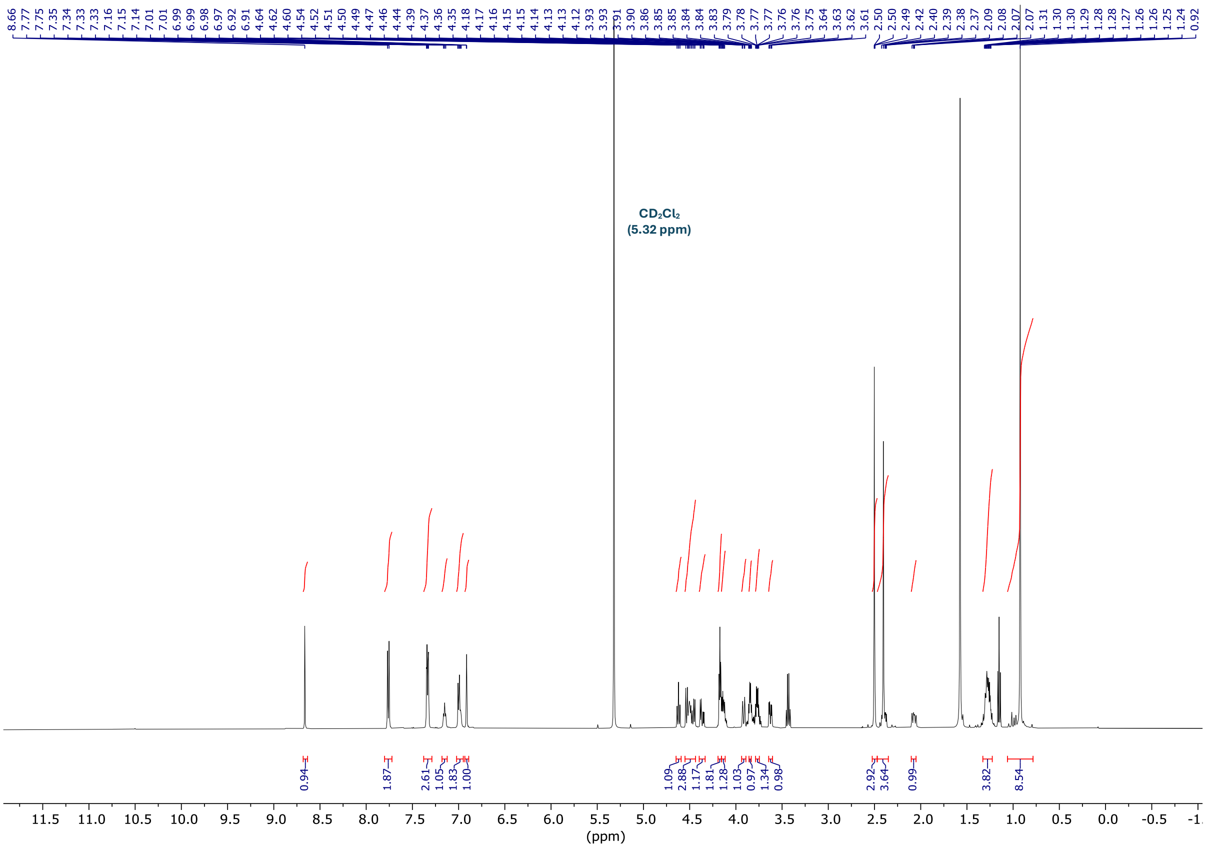

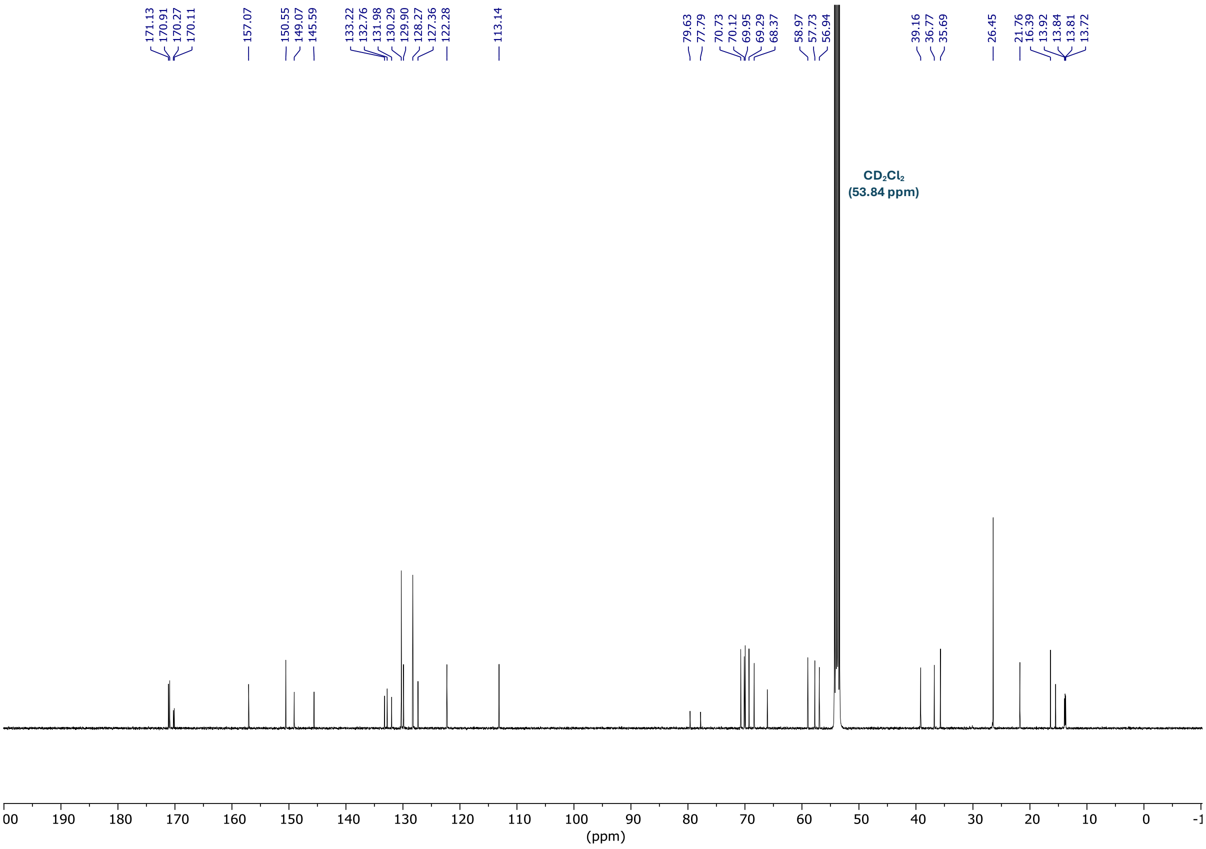

Compound S14. *^{1}H NMR (500 MHz, CD_2Cl_2) and ^{13}C NMR (126 MHz, CD_2Cl_2).*

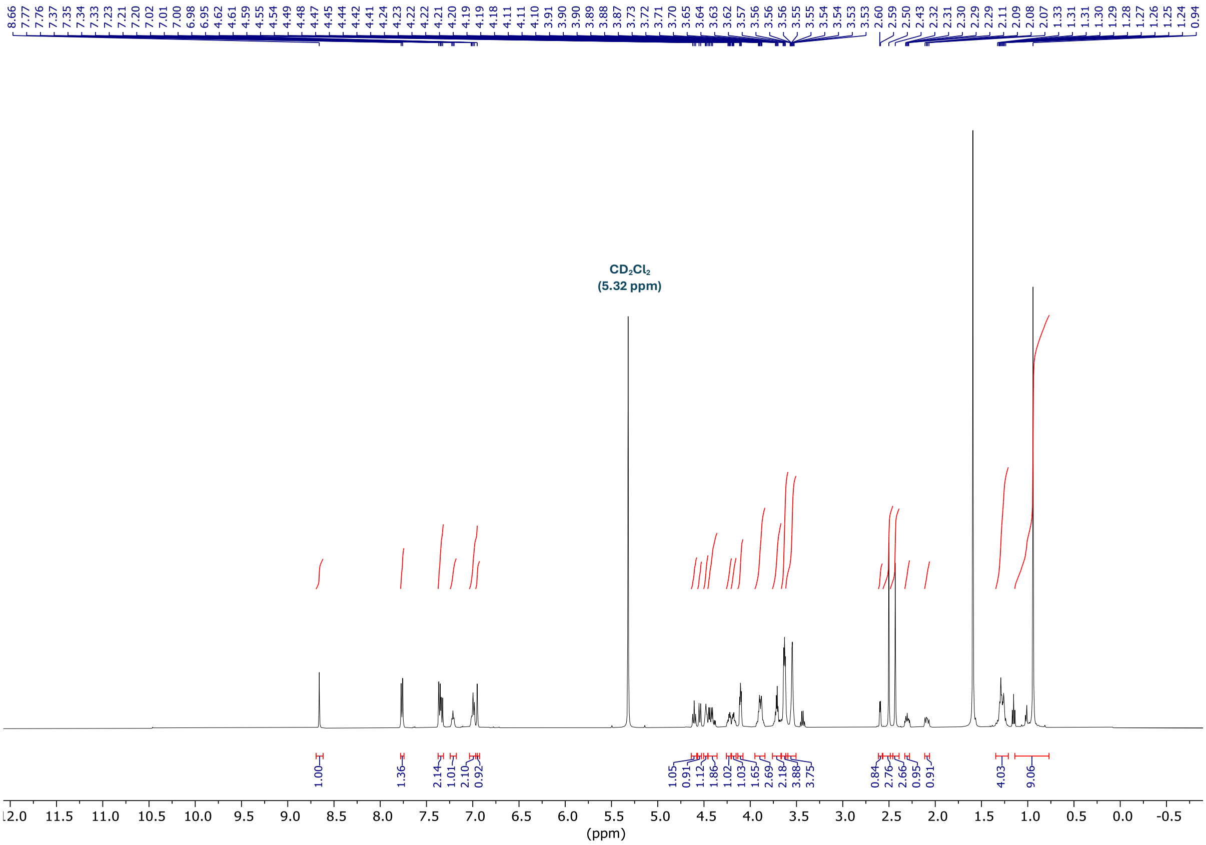

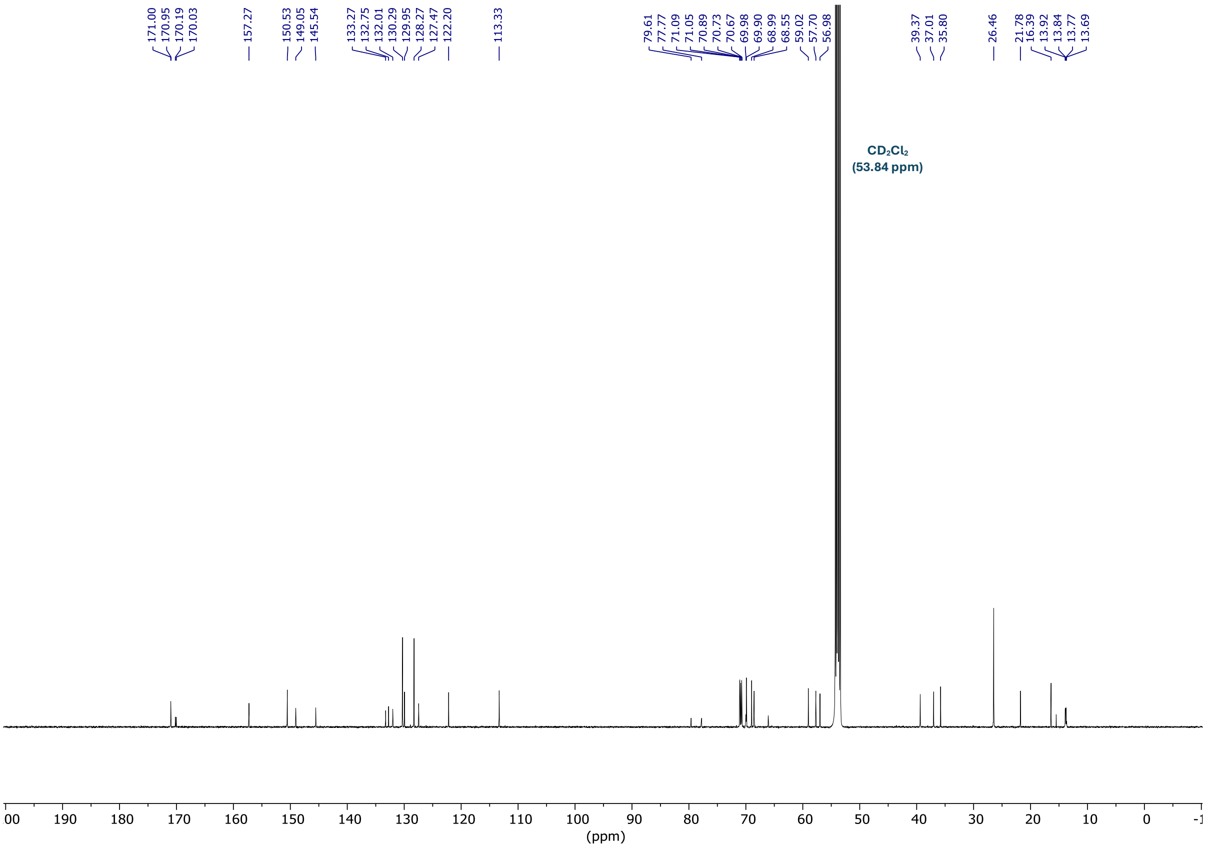

Compound S15. *^{1}H NMR (500 MHz, CD_2Cl_2) and ^{13}C NMR (126 MHz, CD_2Cl_2)*

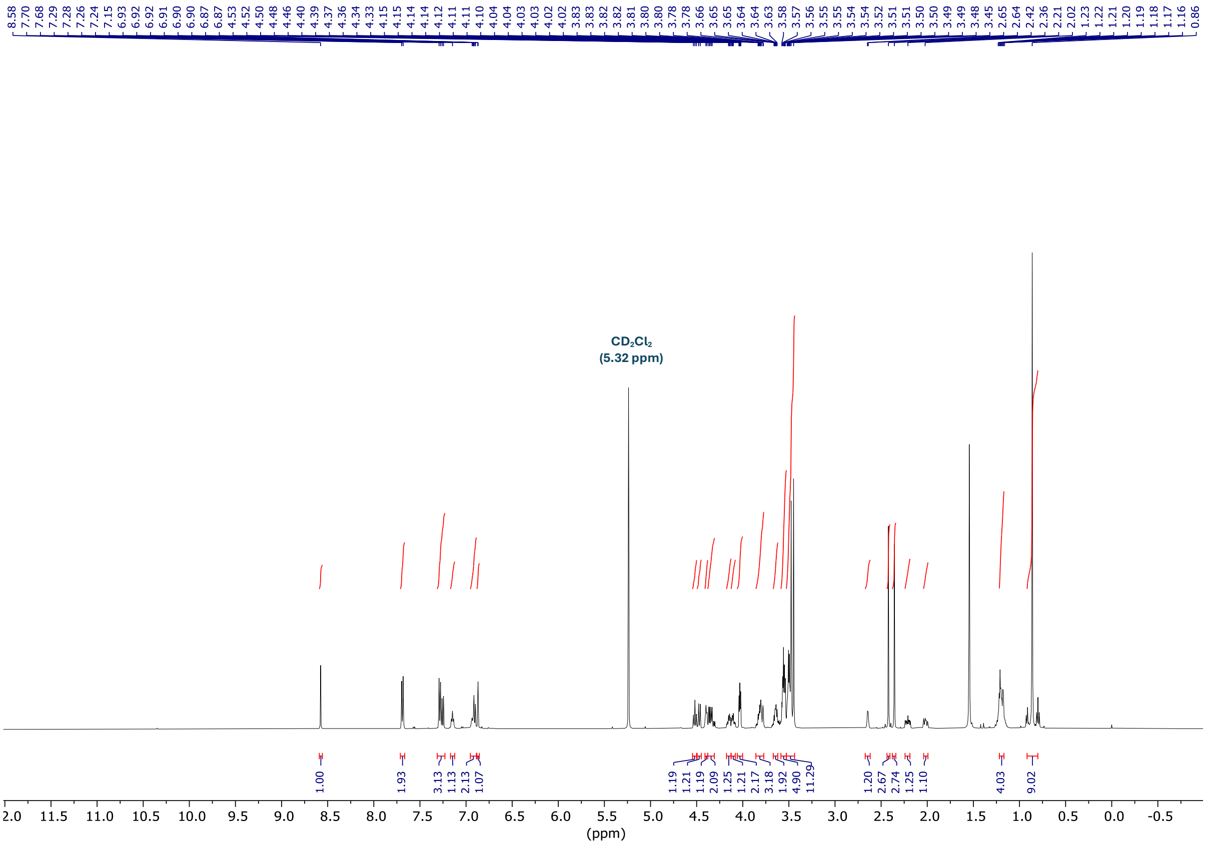

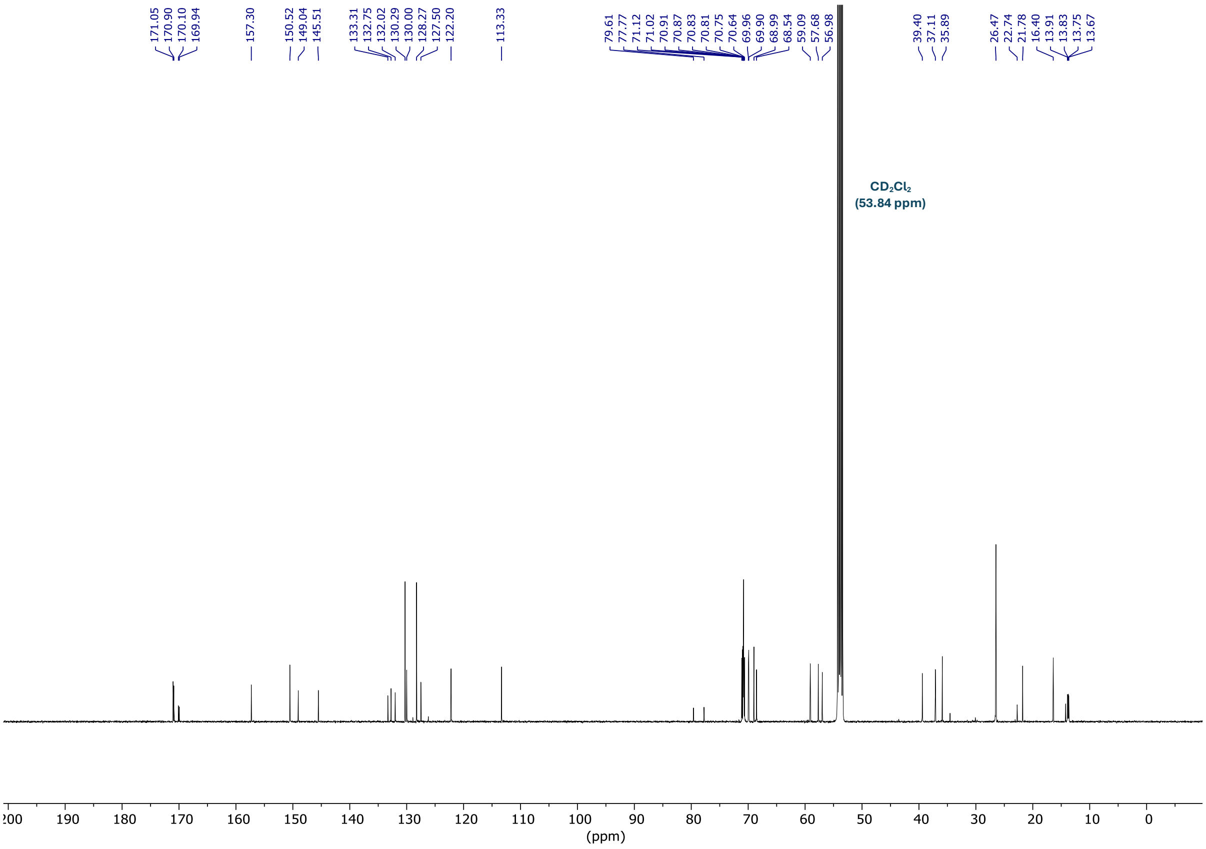

Compound S16. *^{1}H NMR (400 MHz, CD_2Cl_2) and ^{13}C NMR (126 MHz, CD_2Cl_2)*

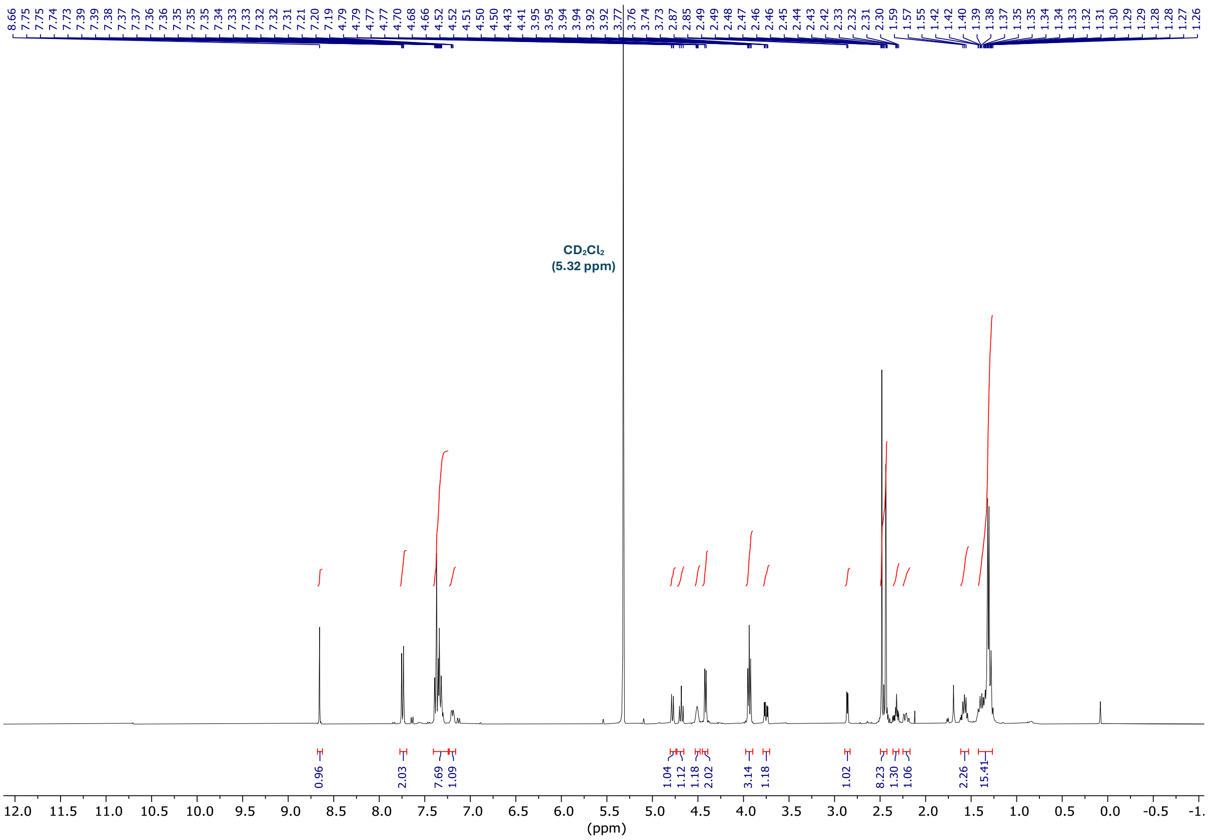

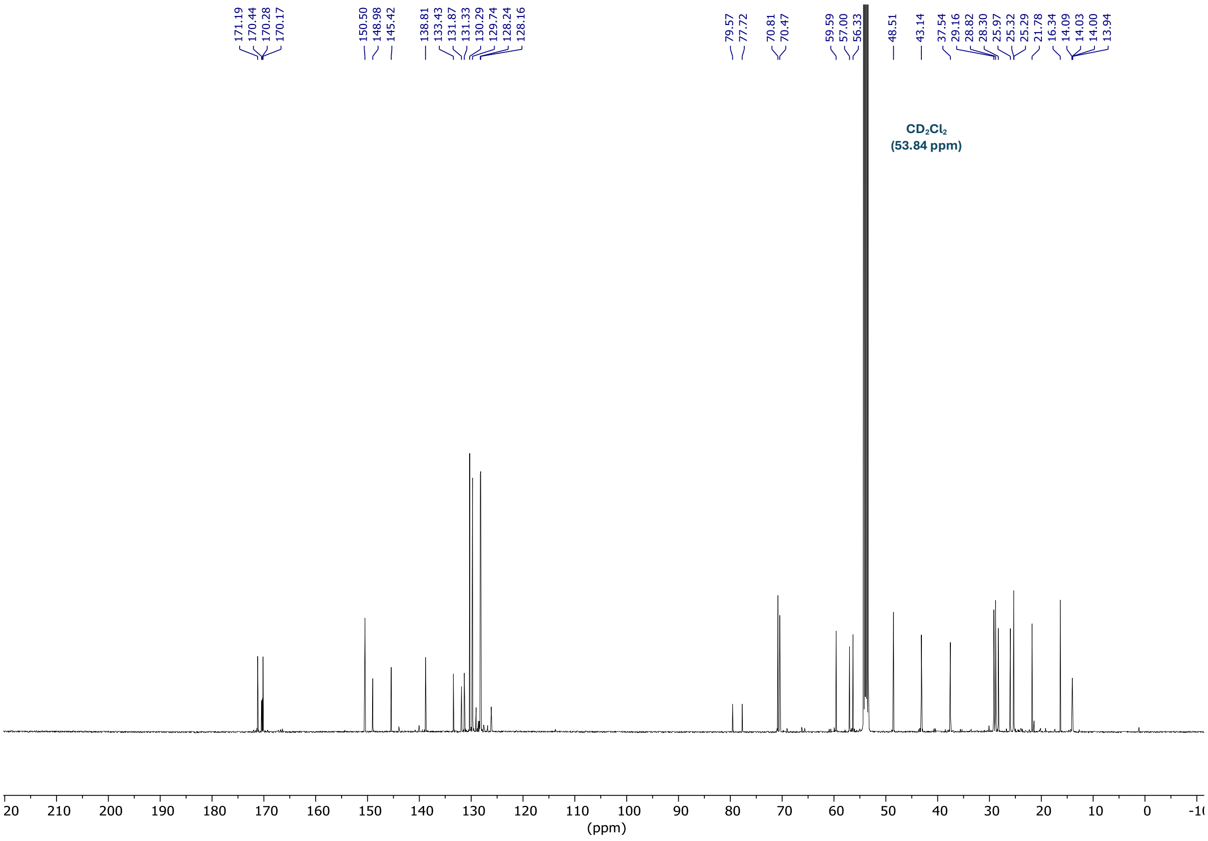

Compound S17. ^{1}H NMR (500 MHz, CD_2Cl_2) and ^{13}C NMR (126 MHz, CD_2Cl_2)

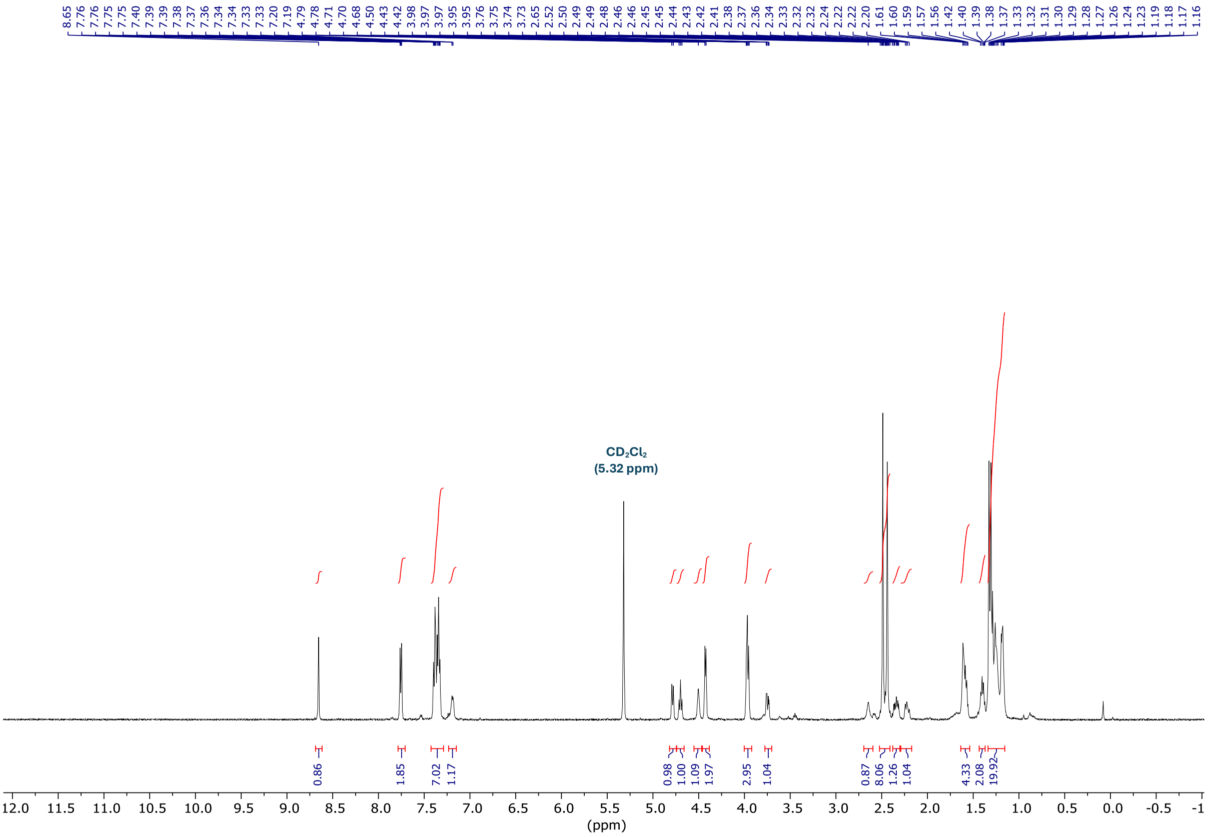

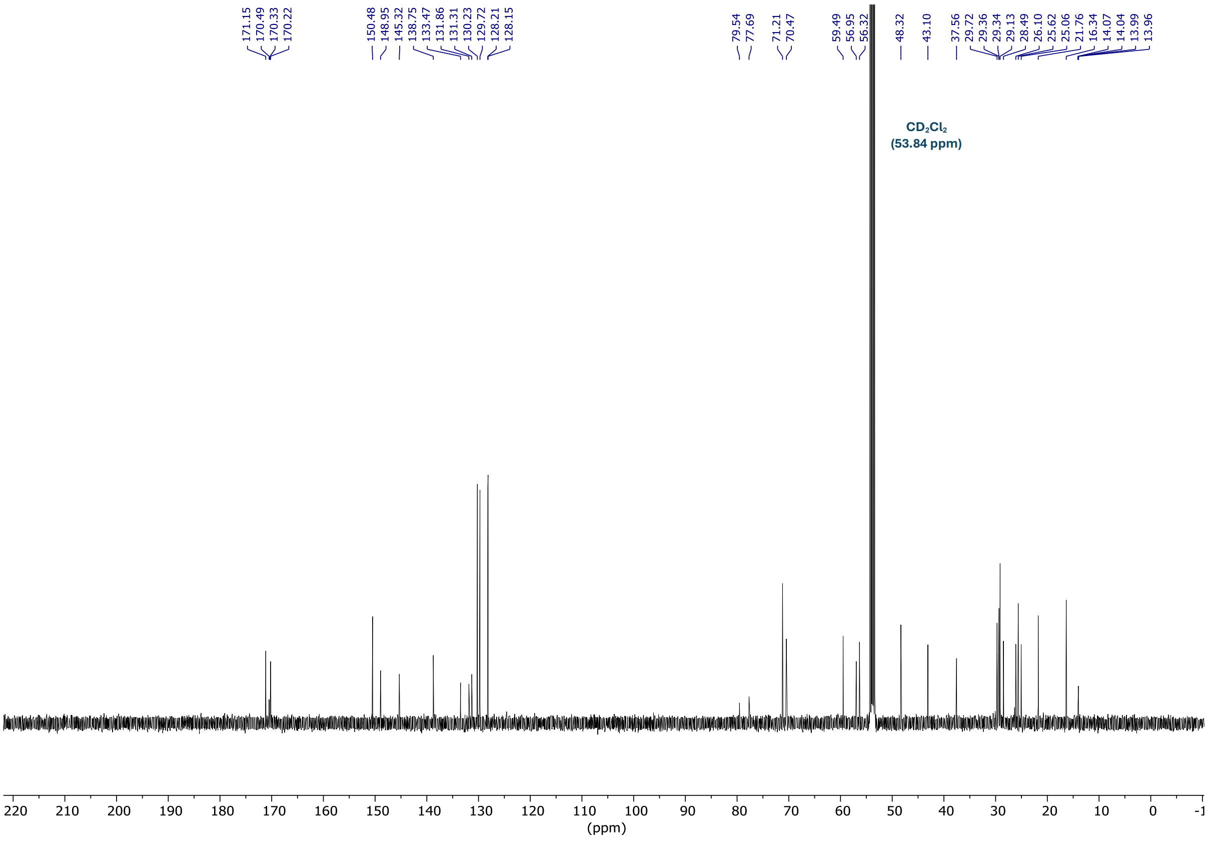

Compound S18. *^{1}H NMR (400 MHz, CD_2Cl_2) and ^{13}C NMR (126 MHz, CD_2Cl_2)*

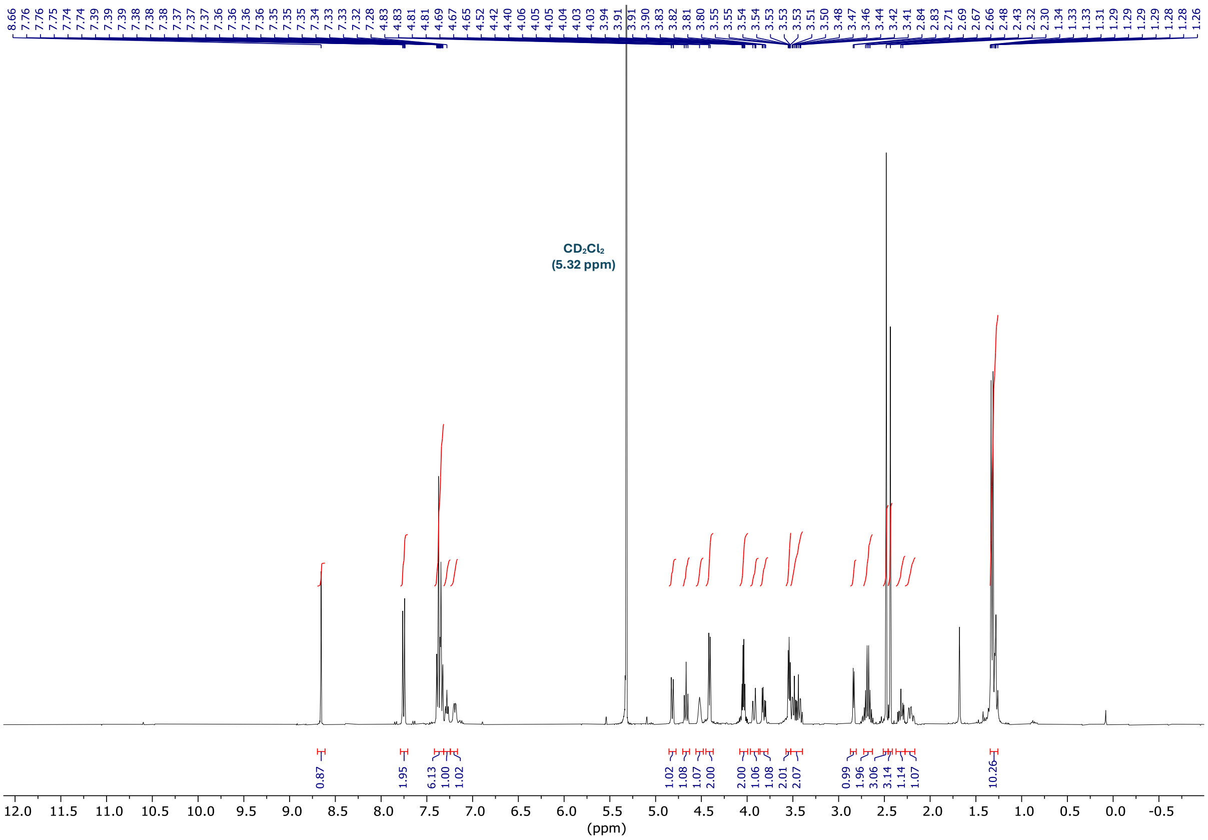

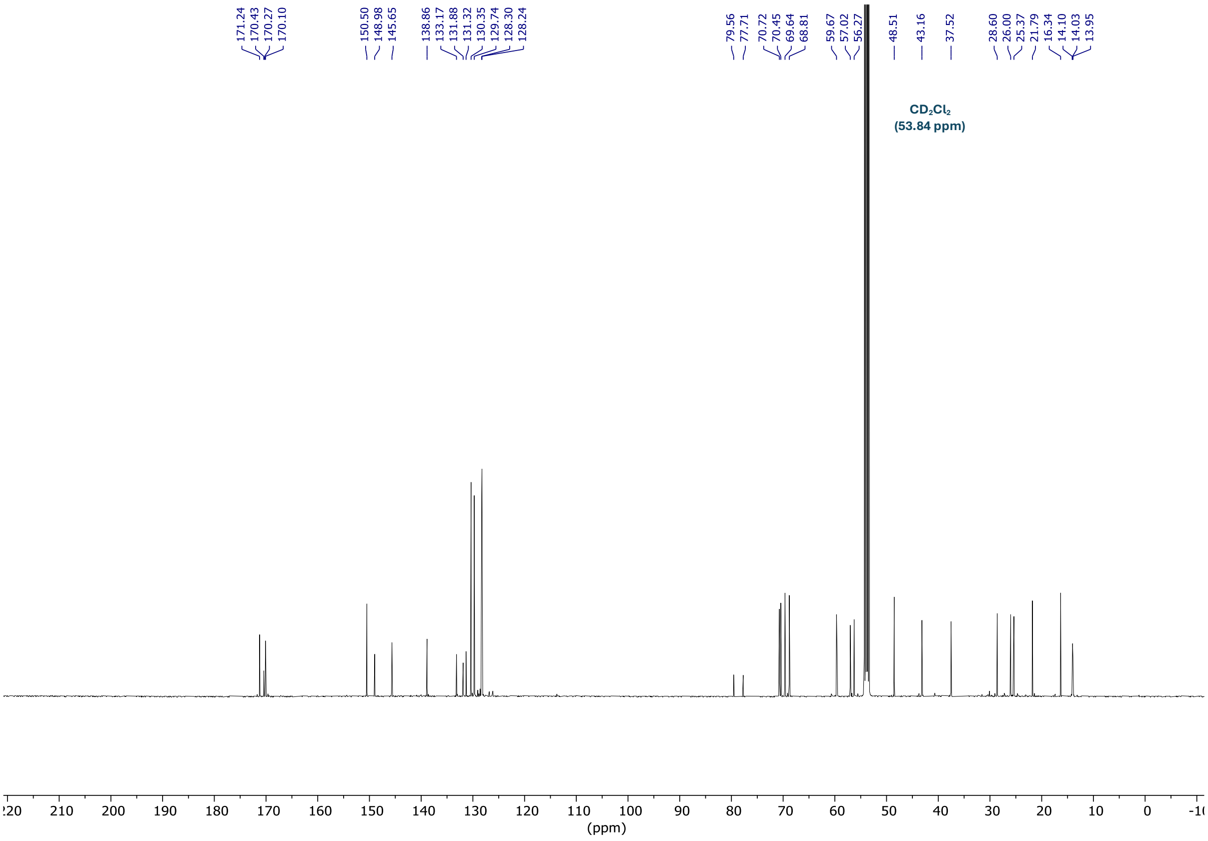

Compound S19. *^{1}H NMR (400 MHz, CD_2Cl_2) and ^{13}C NMR (126 MHz, CD_2Cl_2)*

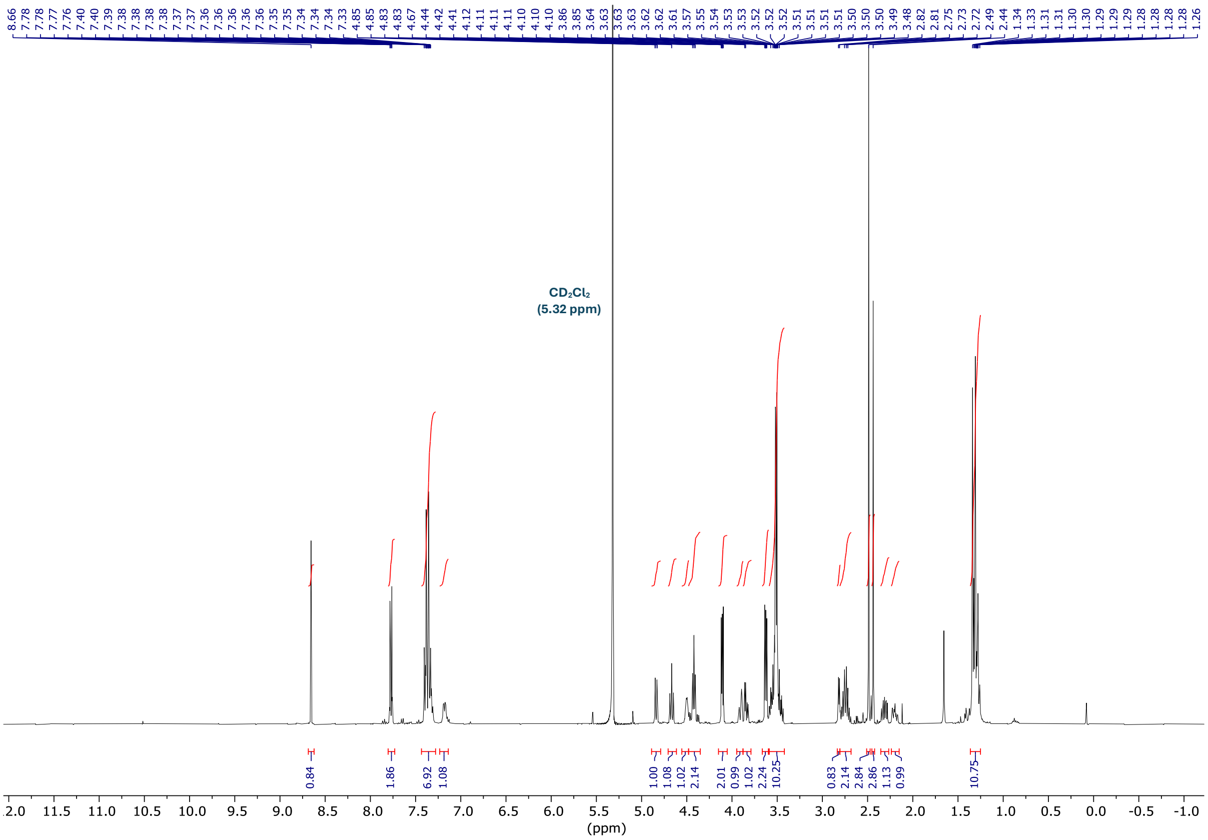

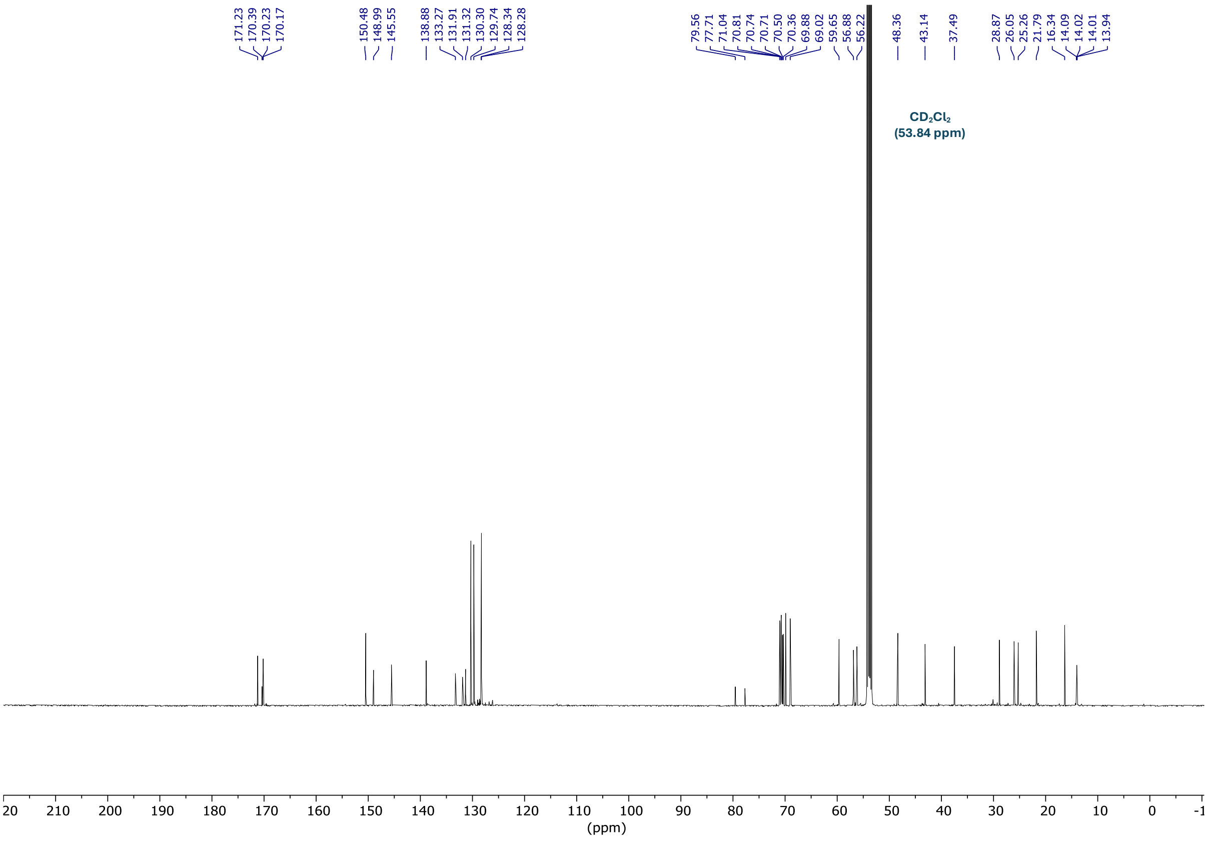

Compound S20. *^{1}H NMR (500 MHz, CD_2Cl_2) and ^{13}C NMR (126 MHz, CD_2Cl_2)*

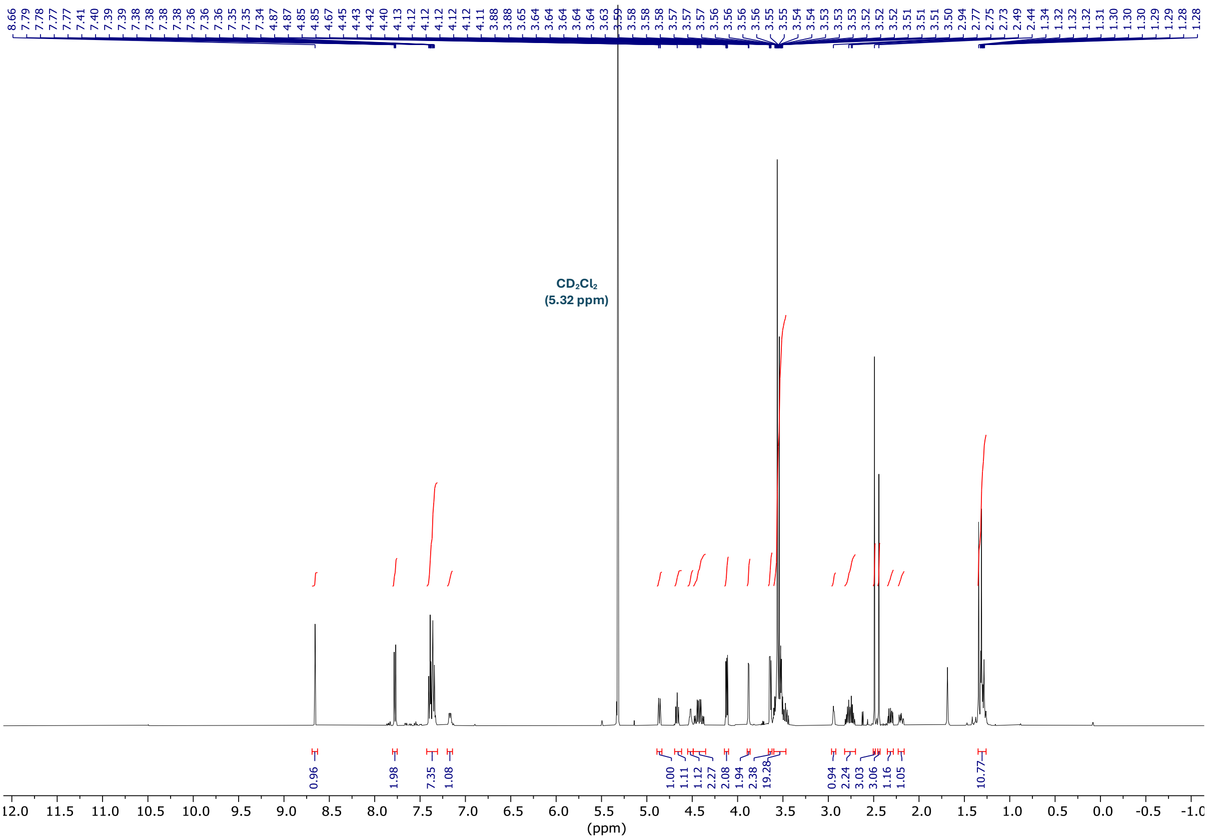

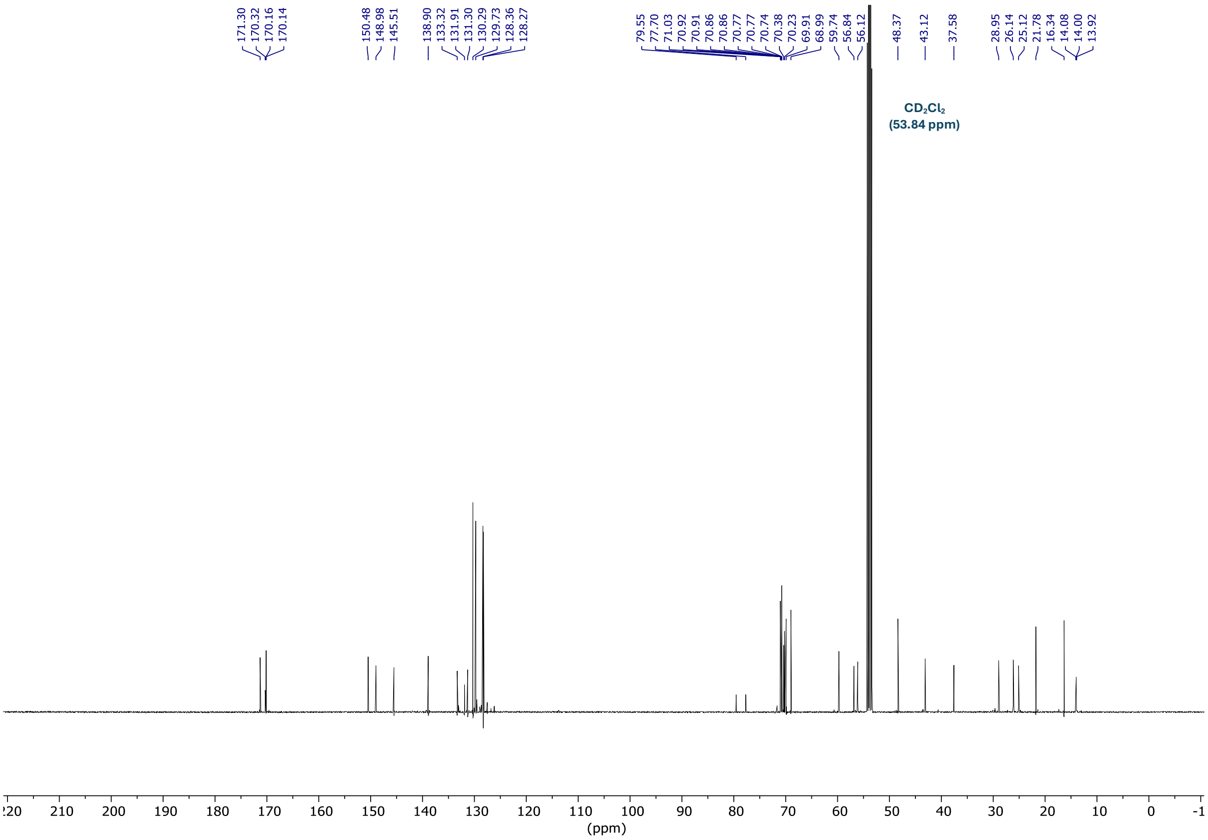

Compound S21. *^{1}H NMR (400 MHz, CD_2Cl_2) and ^{13}C NMR (126 MHz, CD_2Cl_2).*

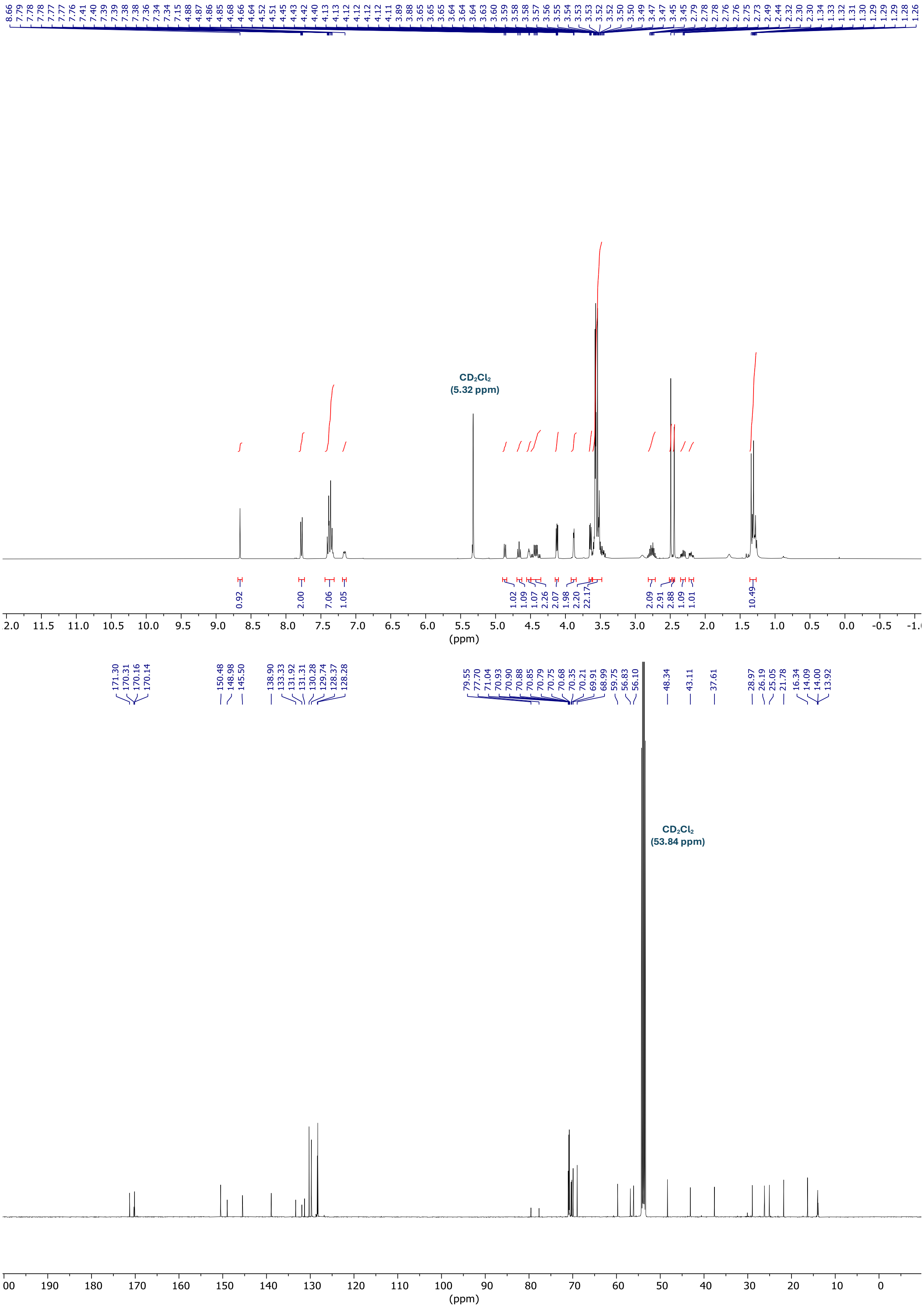

Compound 6. *^{1}H NMR (500 MHz, CD_2Cl_2), ^{13}C NMR (126 MHz, CD_2Cl_2) and ^{19}F NMR (471 MHz, CD_2Cl_2).*

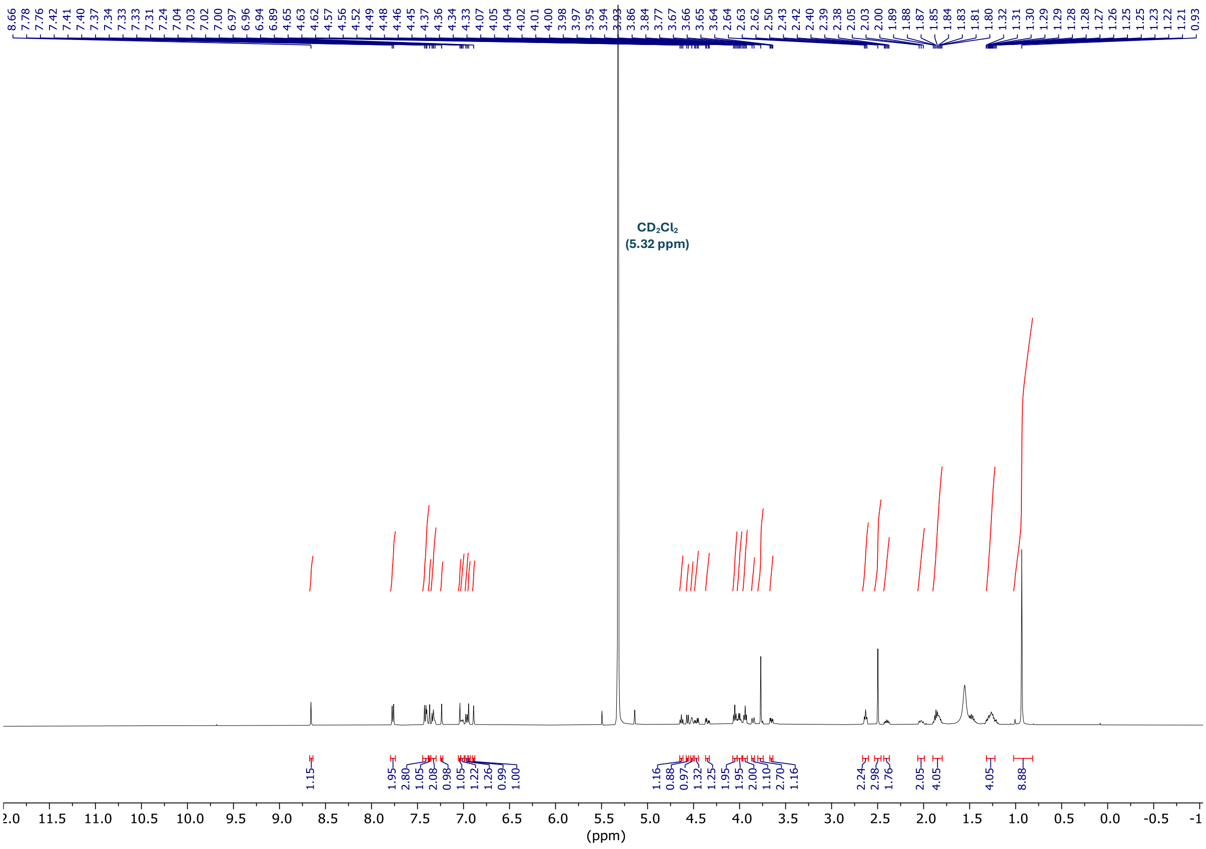

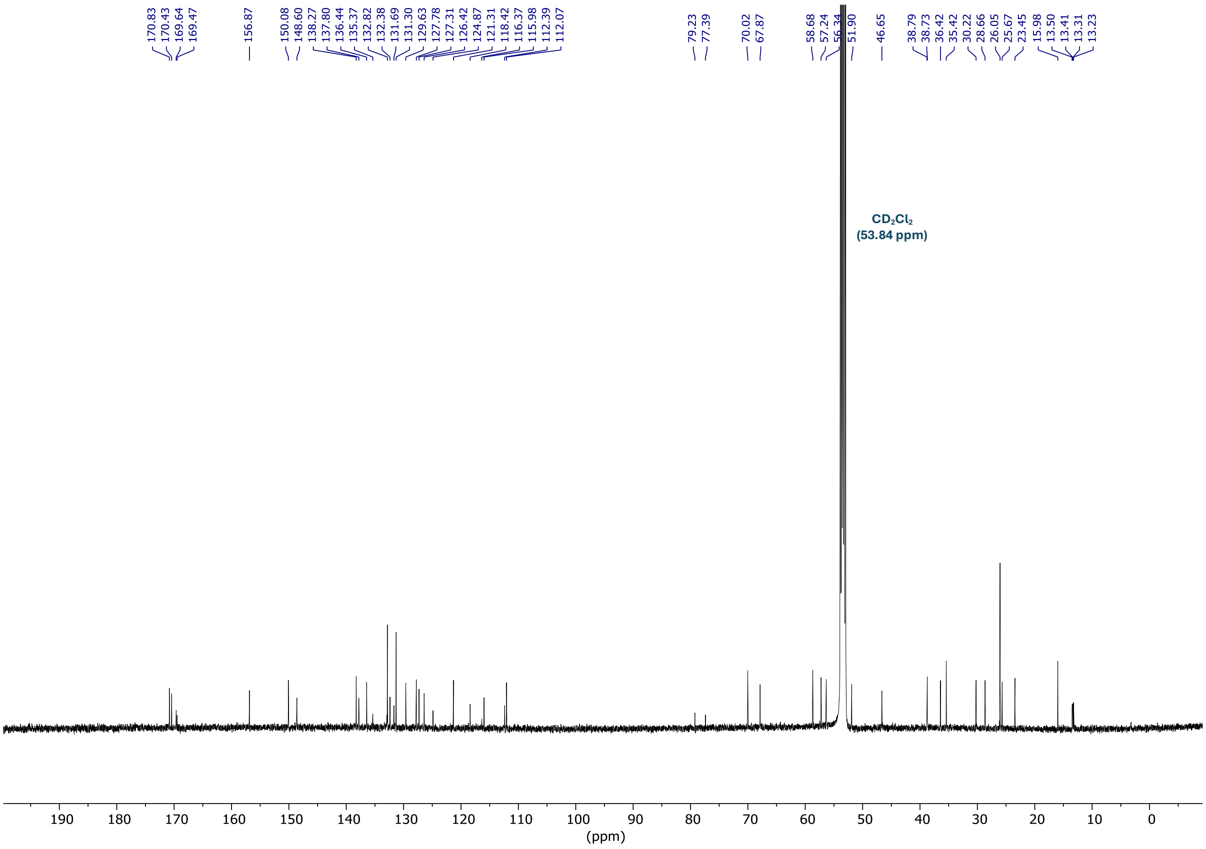

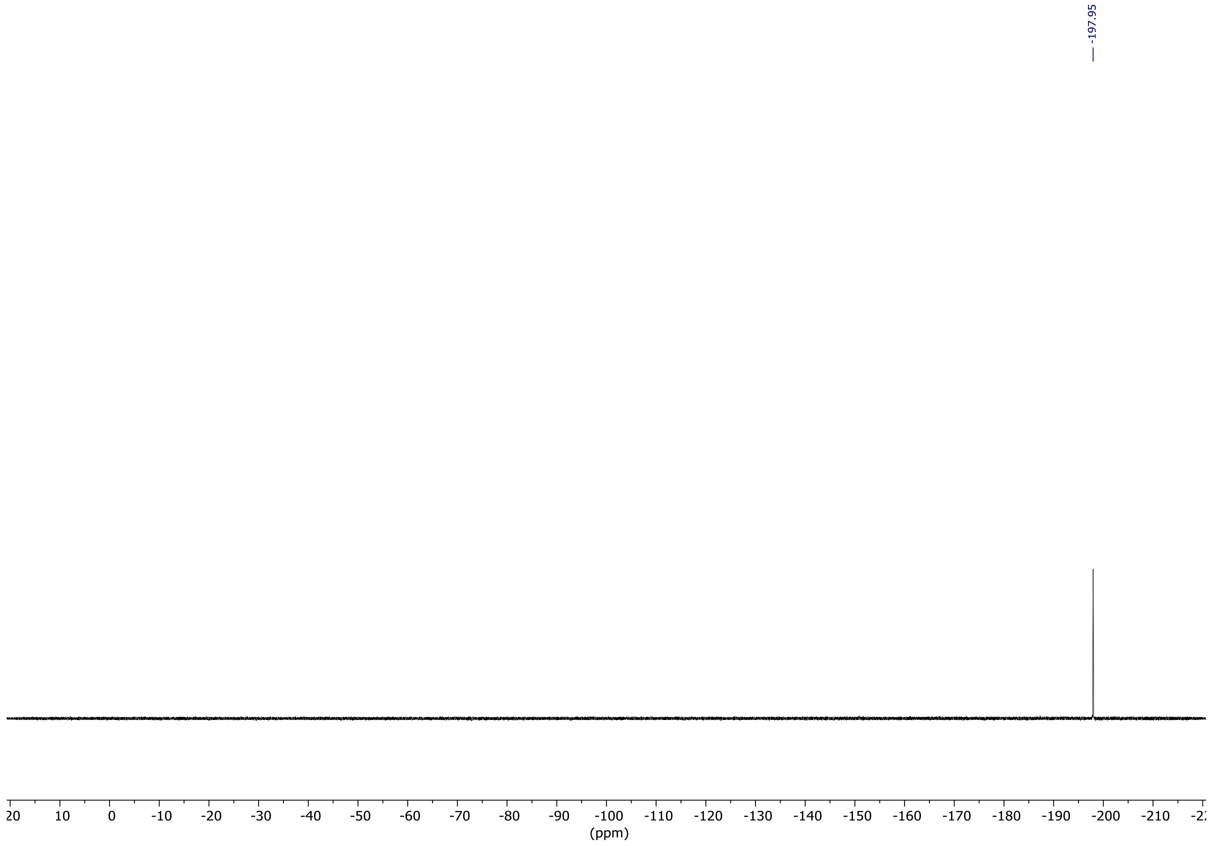
-197.95
20
10
0
-10
-20
-30
-40
-50
-60
-70
-80
-90
-100
-110
-120
-130
-140
-150
-160
-170
-180
-190
-200
-210
(ppm)

Compound 7. *^{1}H NMR (500 MHz, CD_2Cl_2), ^{13}C NMR (126 MHz, CD_2Cl_2) and ^{19}F NMR (377 MHz, CD_2Cl_2)*

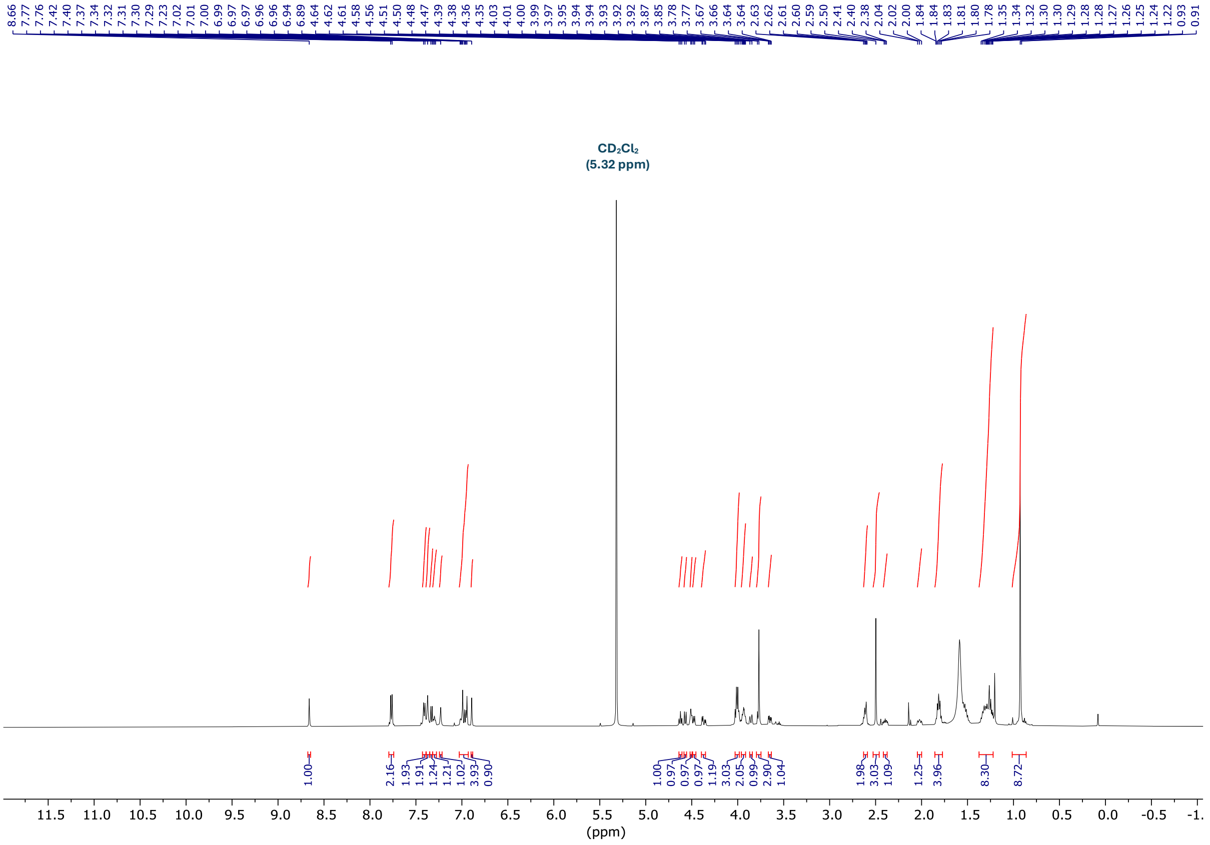

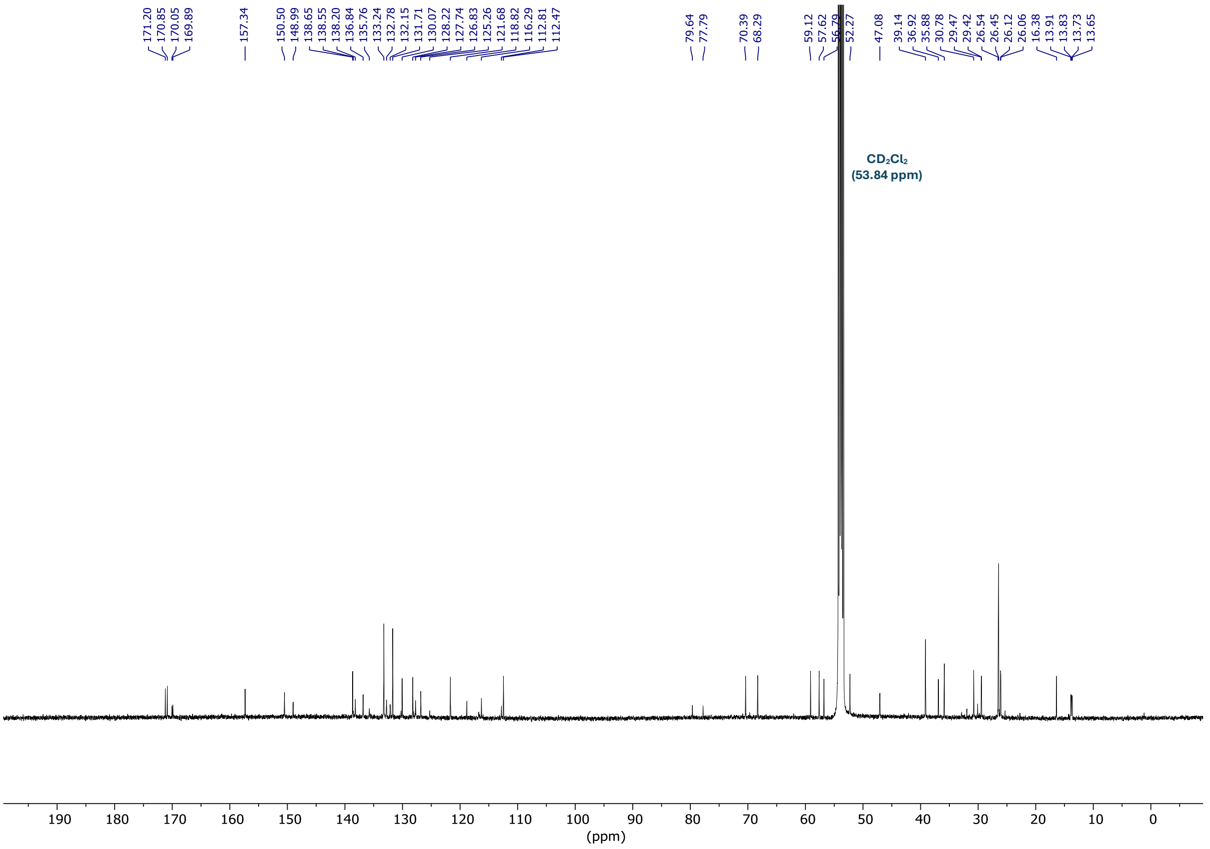

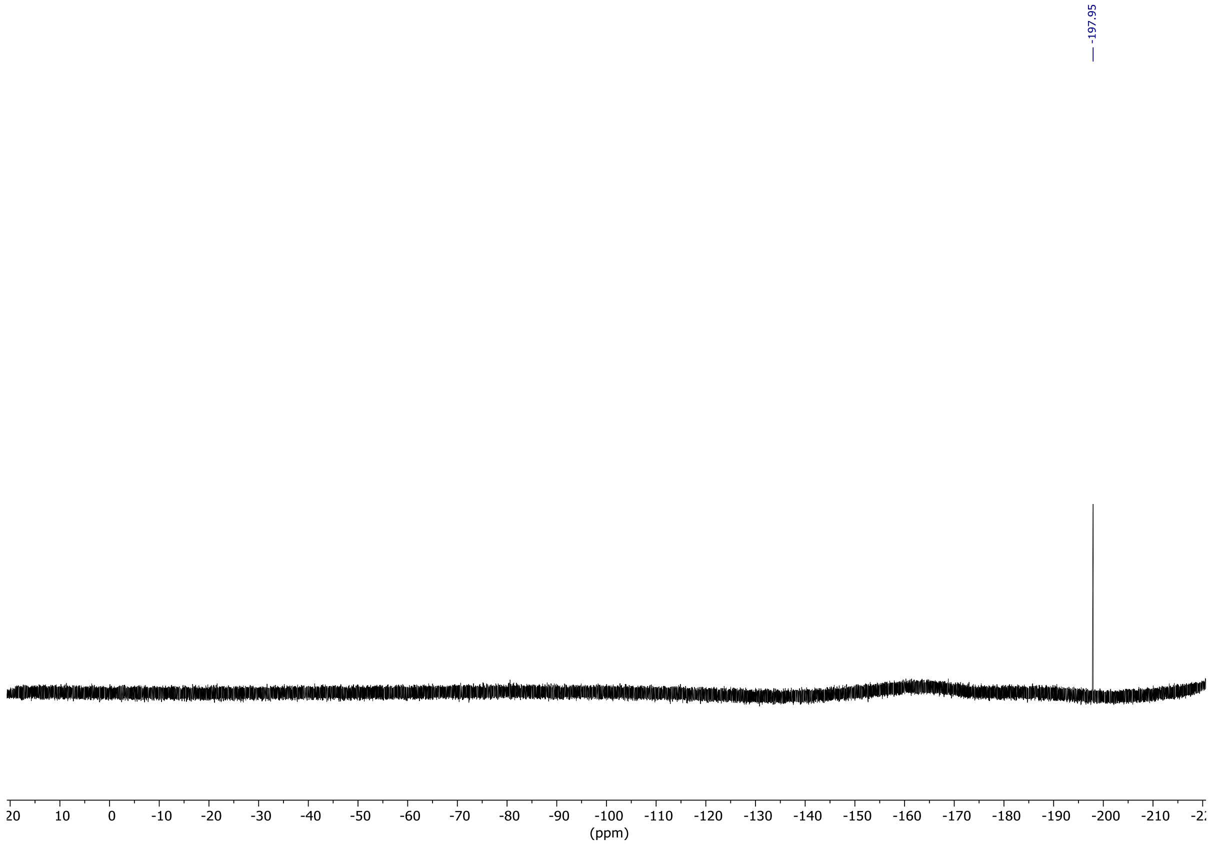

-197.95
20 10 0 -10 -20 -30 -40 -50 -60 -70 -80 -90 -100 -110 -120 -130 -140 -150 -160 -170 -180 -190 -200 -210
(ppm)

Compound 8. *^{1}H NMR (500 MHz, CD_2Cl_2), ^{13}C NMR (126 MHz, CD_2Cl_2) and ^{19}F NMR (471 MHz, CD_2Cl_2).*

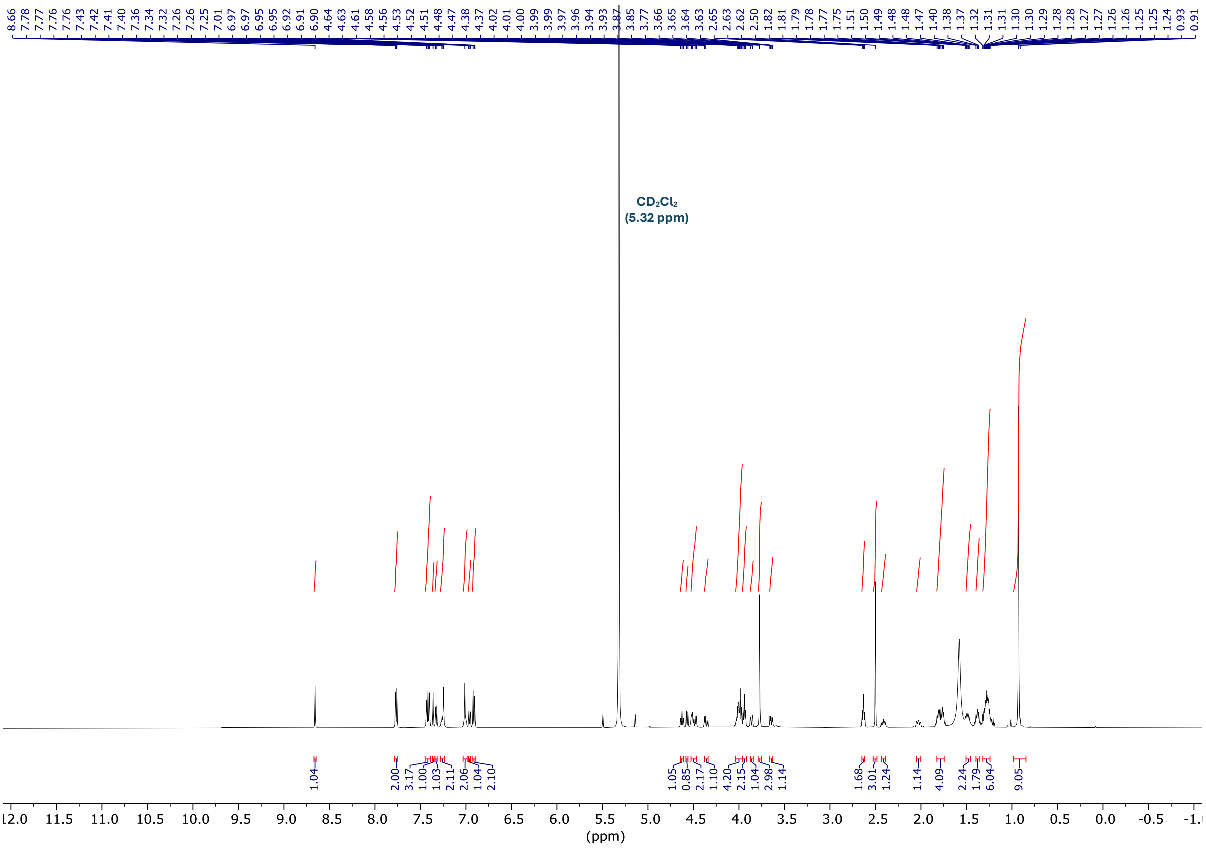

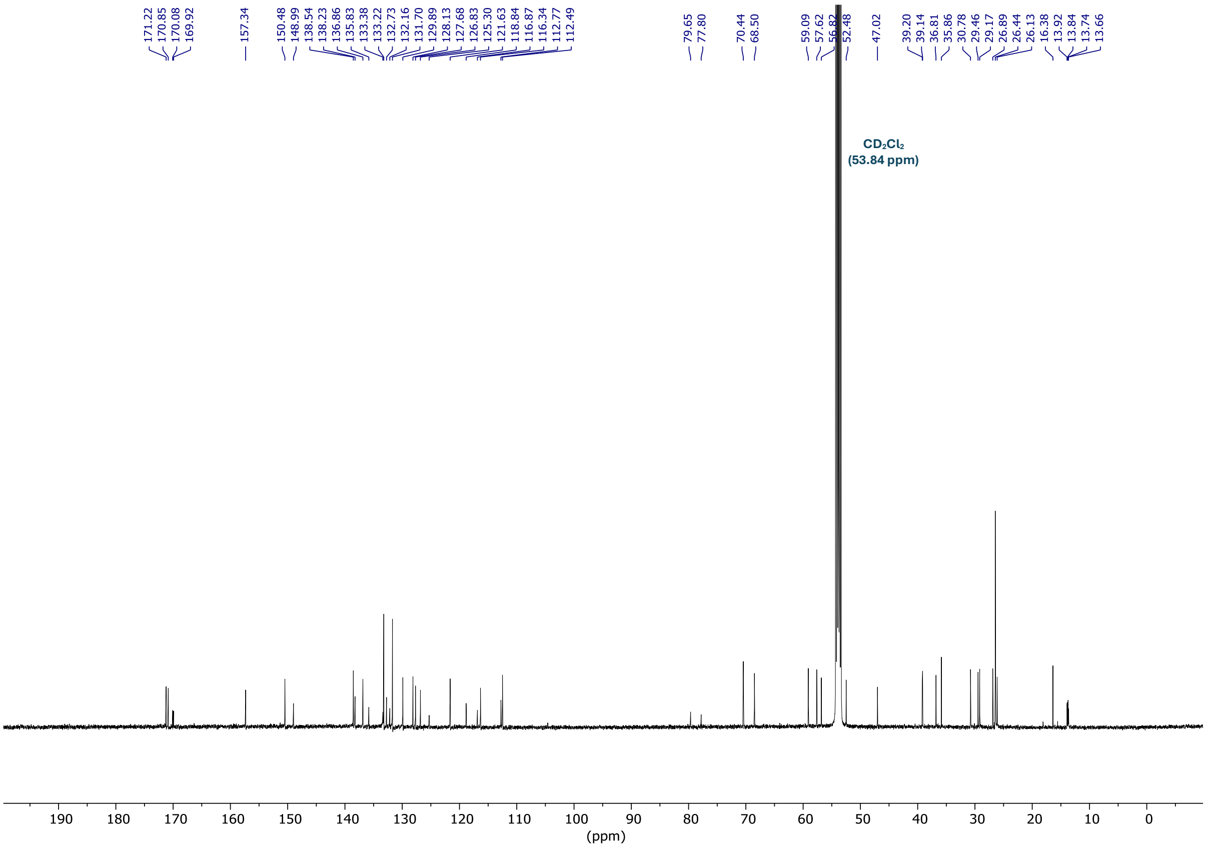

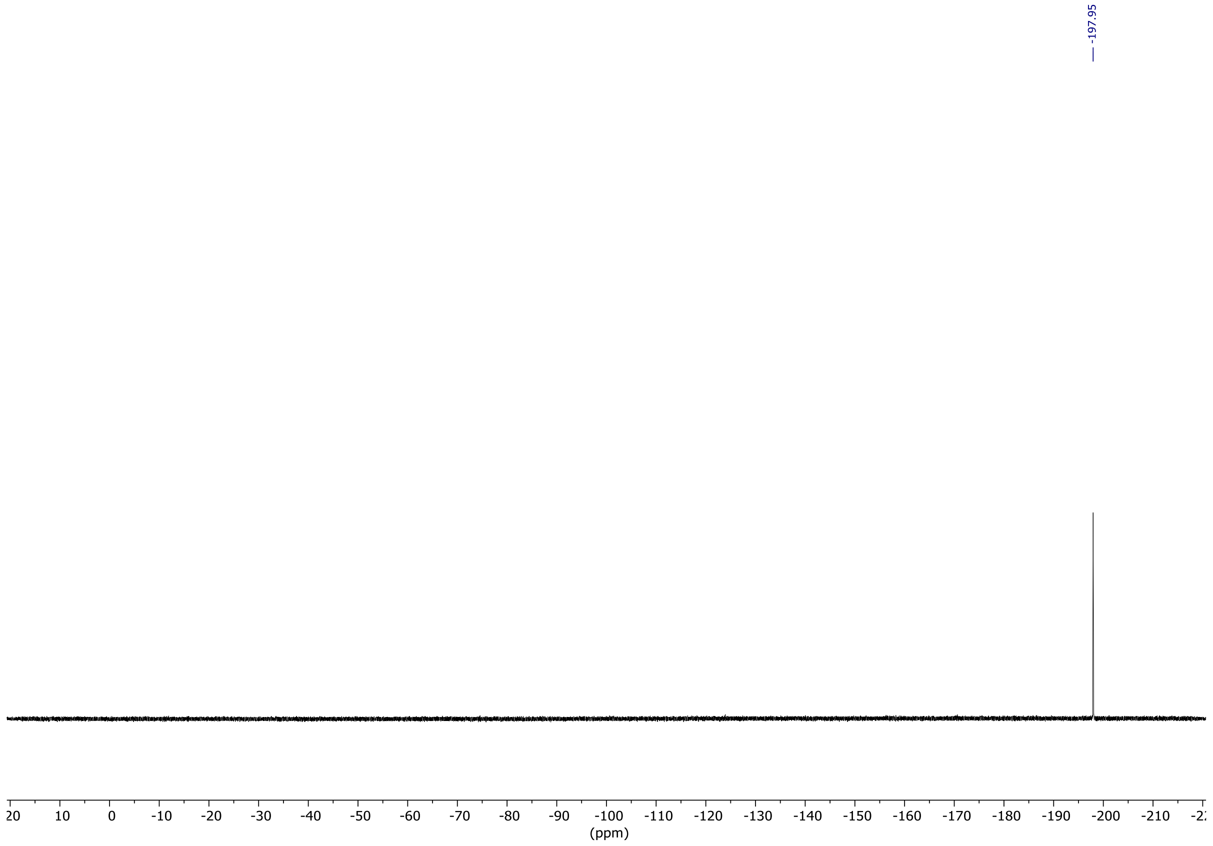
-197.95
20
10
0
-10
-20
-30
-40
-50
-60
-70
-80
-90
-100
-110
-120
-130
-140
-150
-160
-170
-180
-190
-200
-210
(ppm)

Compound 9. *^{1}H NMR (500 MHz, CD_2Cl_2) and ^{13}C NMR (126 MHz, CD_2Cl_2)*

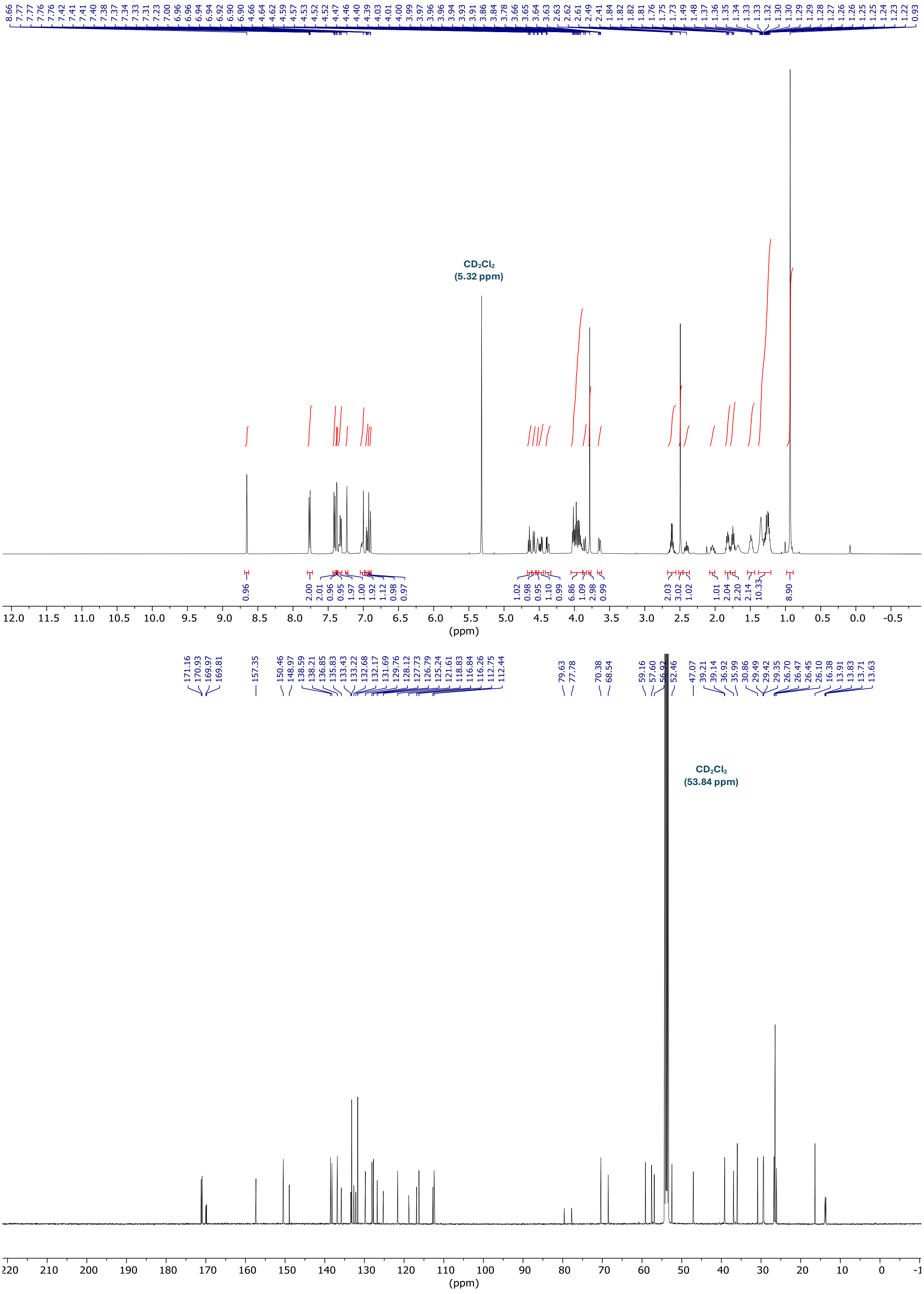

Compound 10. *^{1}H NMR (500 MHz, CD_2Cl_2), ^{13}C NMR (126 MHz, CD_2Cl_2) and ^{19}F NMR (377 MHz, CD_2Cl_2)*

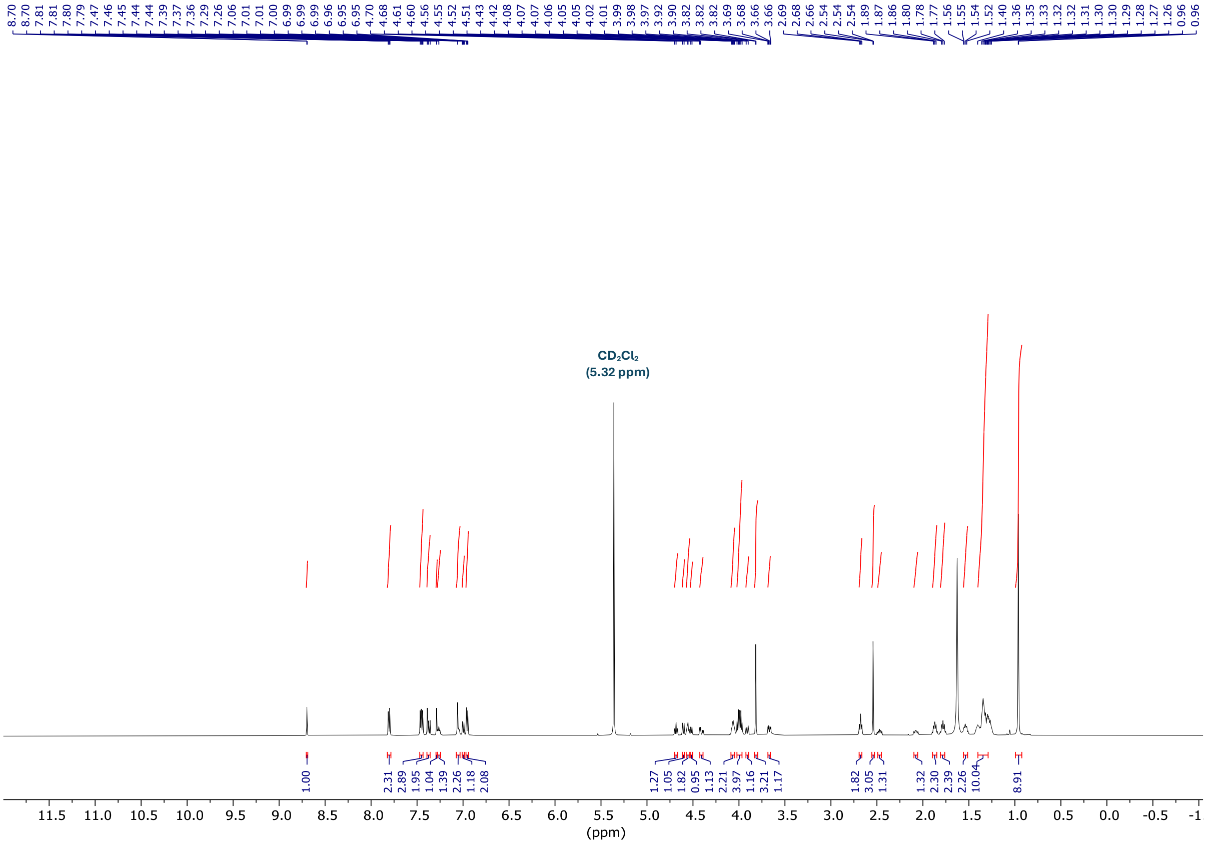

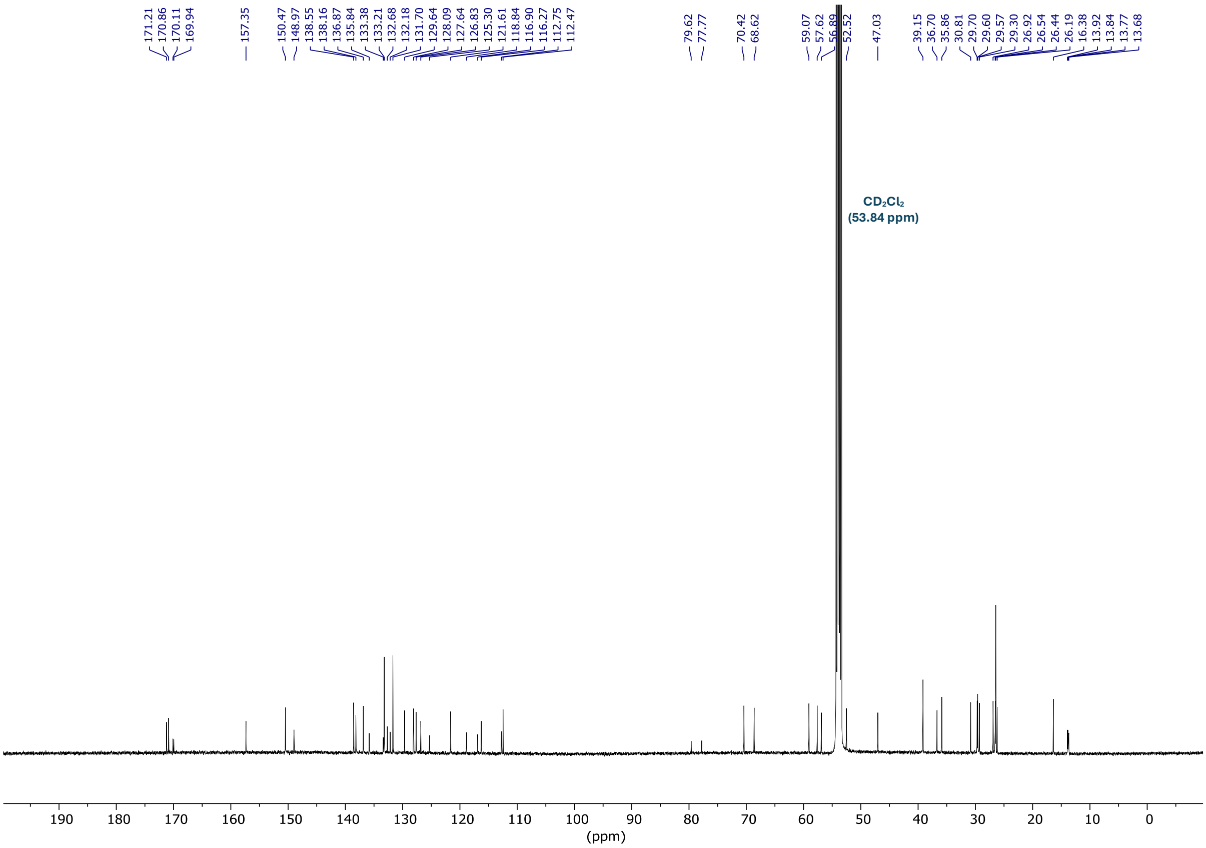

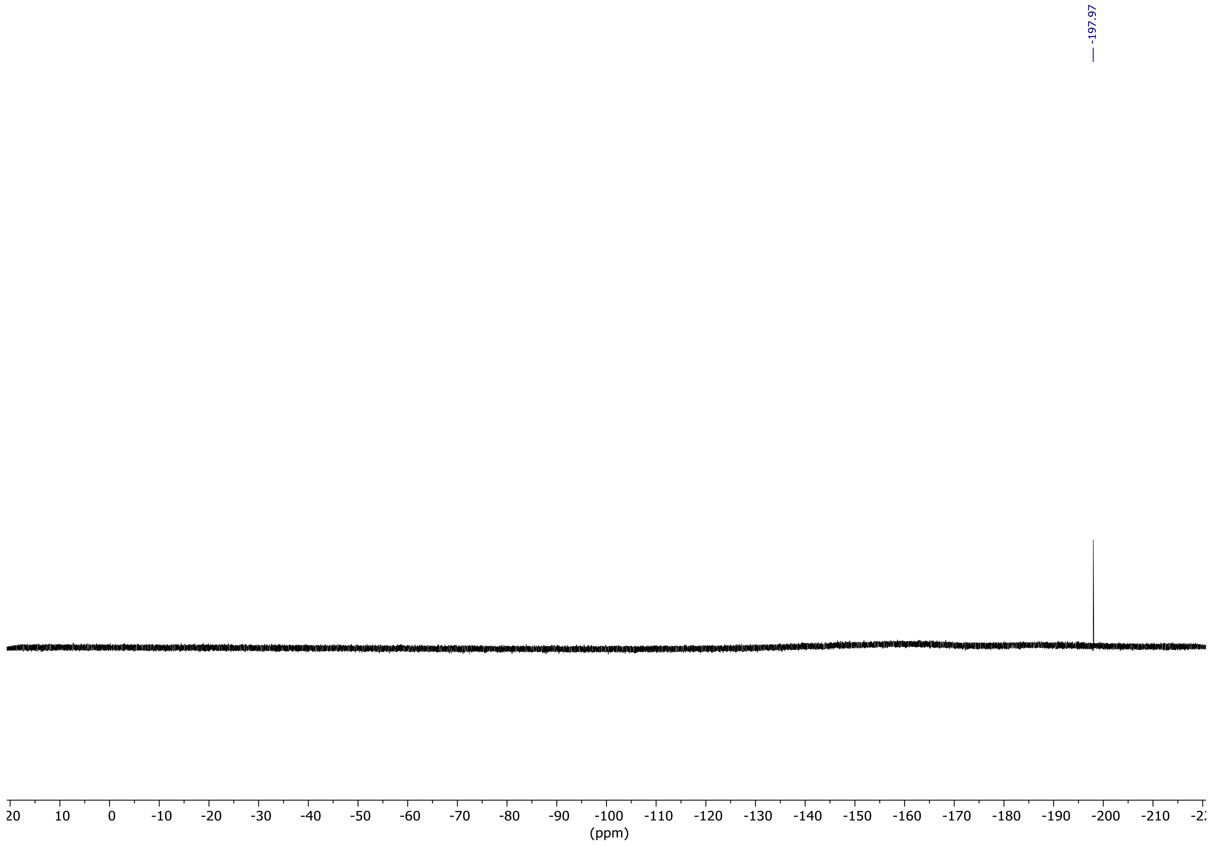
-197.97
20 10 0 -10 -20 -30 -40 -50 -60 -70 -80 -90 -100 -110 -120 -130 -140 -150 -160 -170 -180 -190 -200 -210
(ppm)

Compound 11. *^{1}H NMR (500 MHz, CD_2Cl_2), ^{13}C NMR (126 MHz, CD_2Cl_2) and ^{19}F NMR (471 MHz, CD_2Cl_2)*

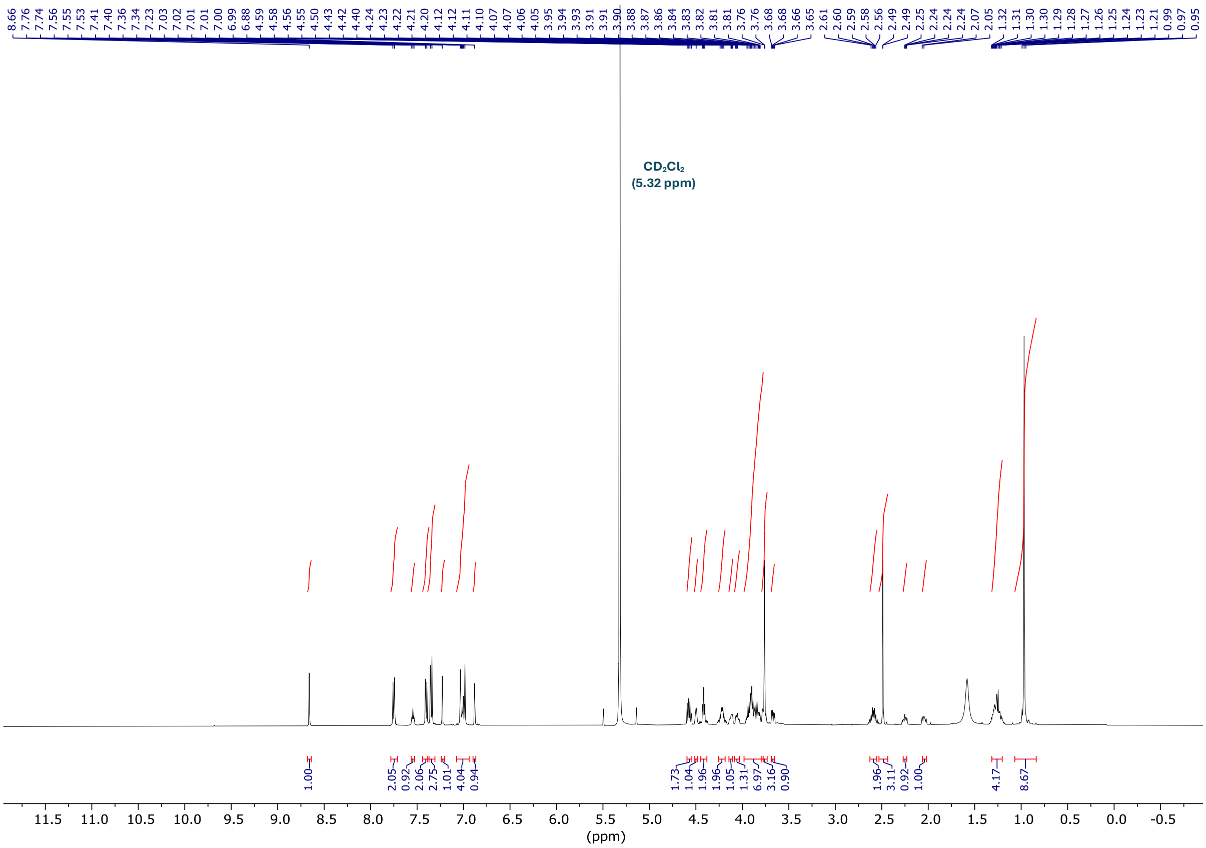

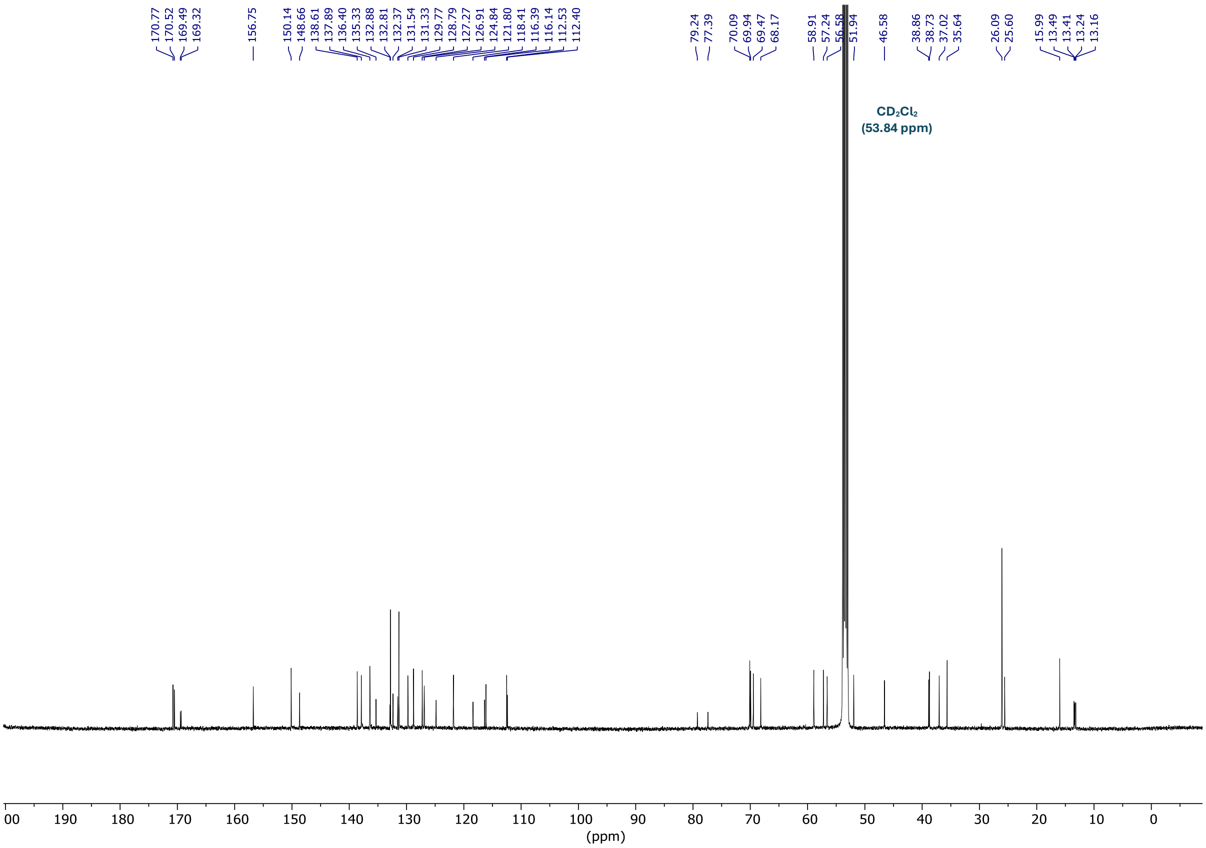

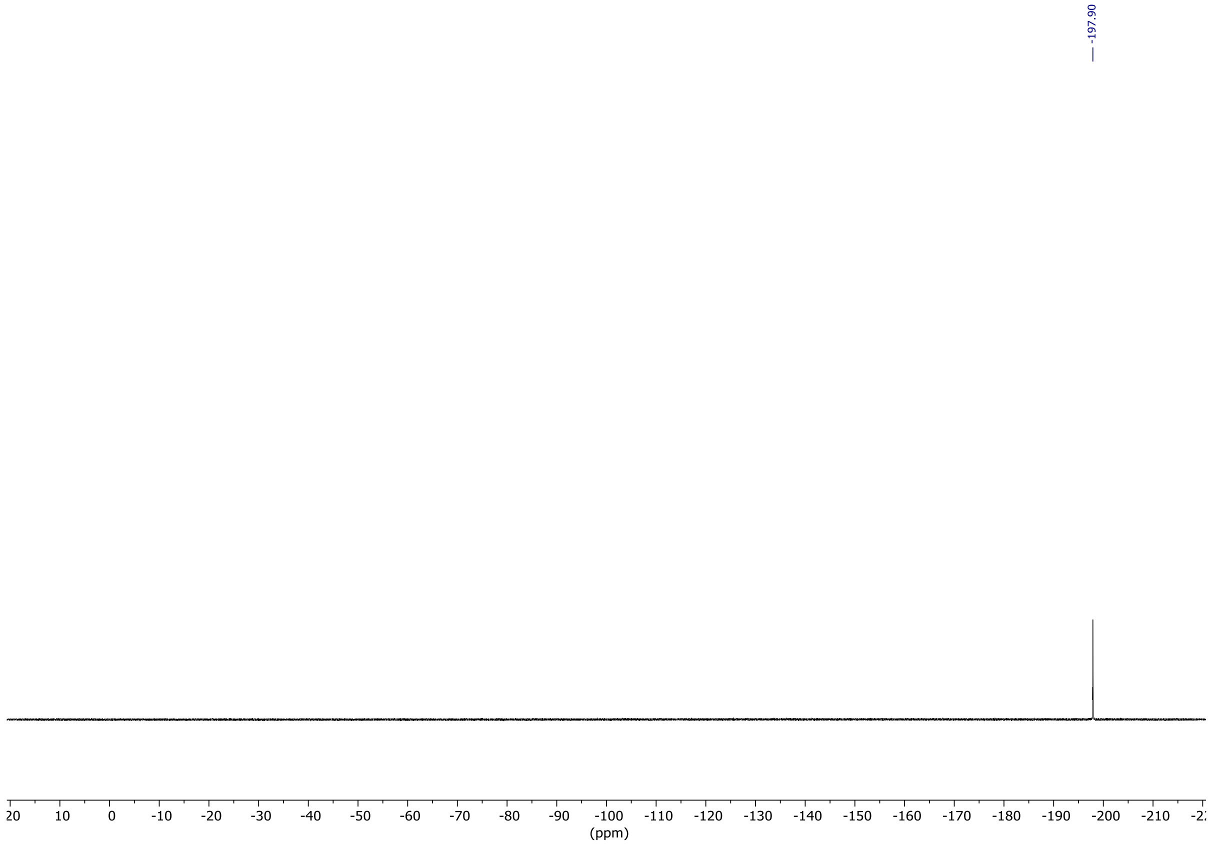
-197.90
20
10
0
-10
-20
-30
-40
-50
-60
-70
-80
-90
-100
-110
-120
-130
-140
-150
-160
-170
-180
-190
-200
-210
(ppm)

Compound 12. *^{1}H NMR (400 MHz, CD_2Cl_2), ^{13}C NMR (126 MHz, CD_2Cl_2) and ^{19}F NMR (377 MHz, CD_2Cl_2)*

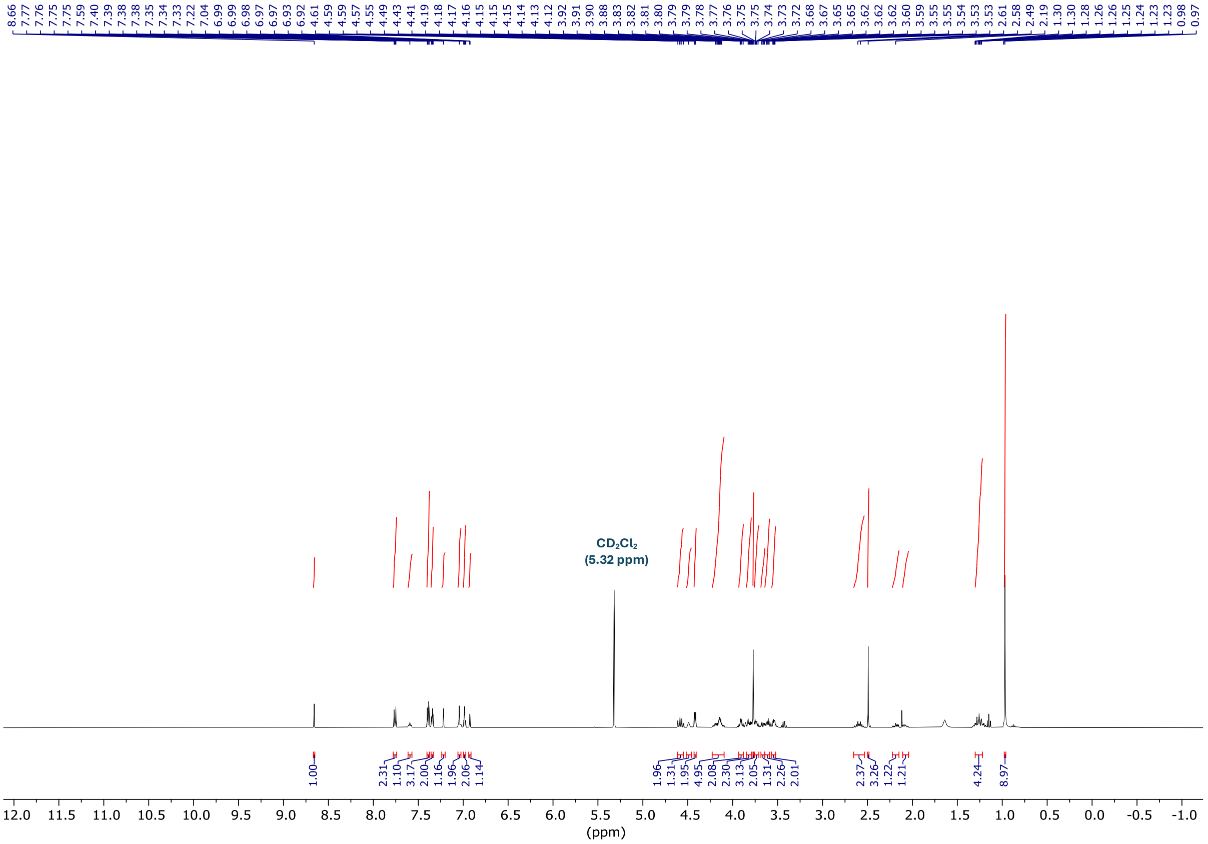

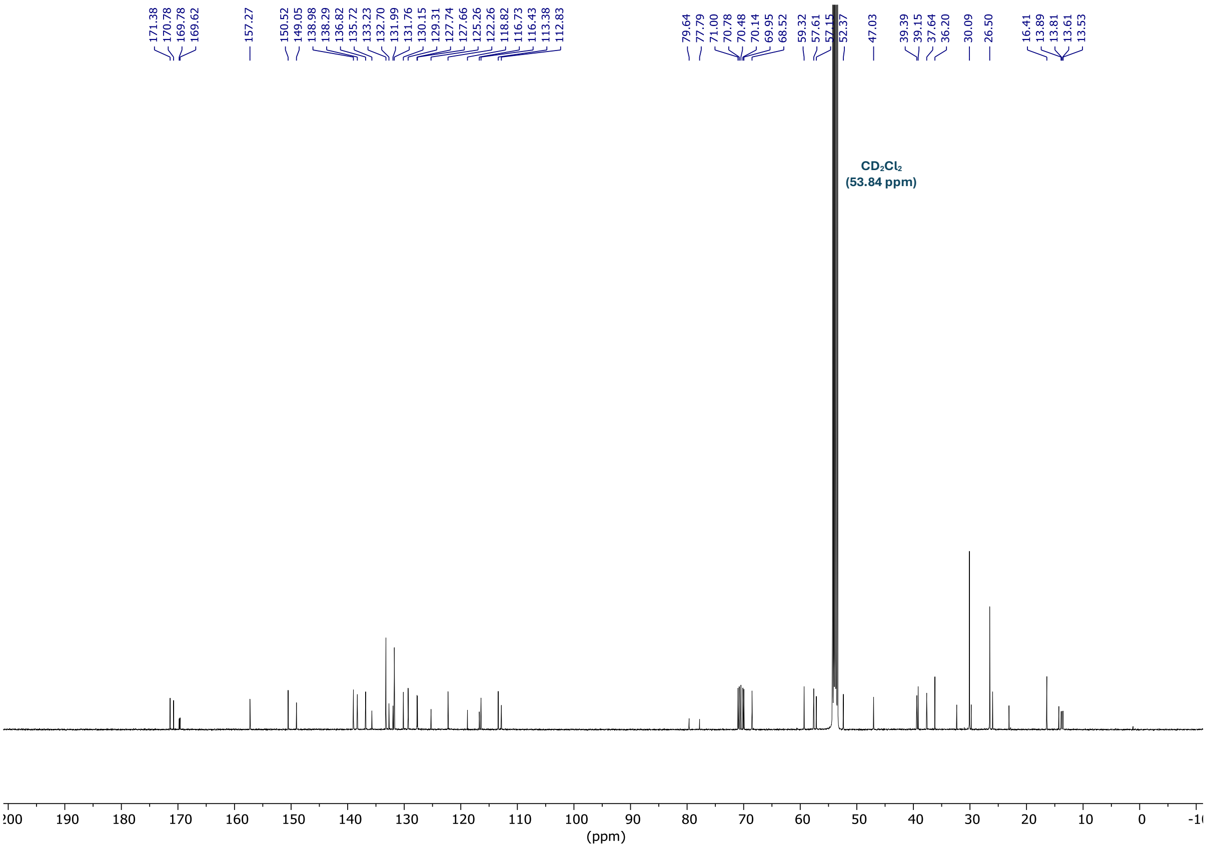

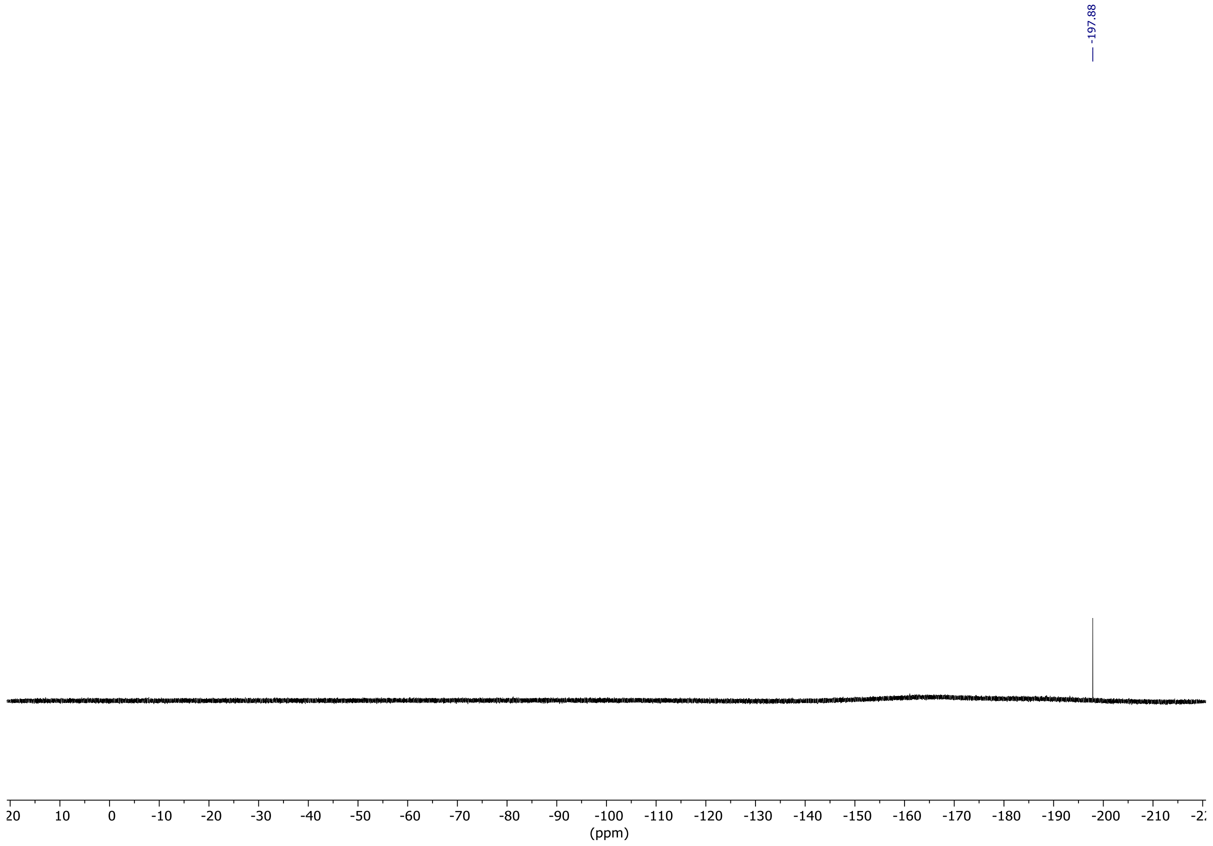
-197.88
20 10 0 -10 -20 -30 -40 -50 -60 -70 -80 -90 -100 -110 -120 -130 -140 -150 -160 -170 -180 -190 -200 -210
(ppm)

Compound 13. *^{1}H NMR (500 MHz, CD_2Cl_2), ^{13}C NMR (126 MHz, CD_2Cl_2) and ^{19}F NMR (471 MHz, CD_2Cl_2)*

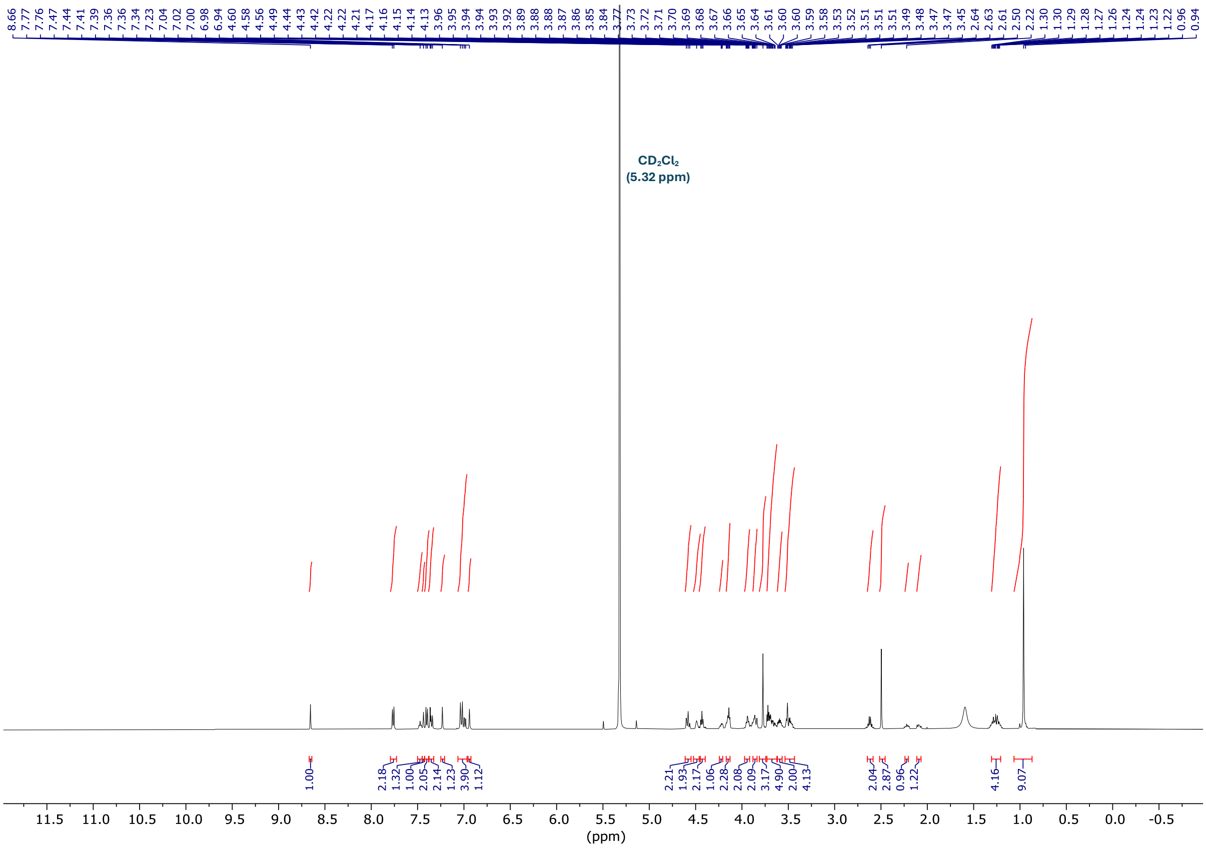

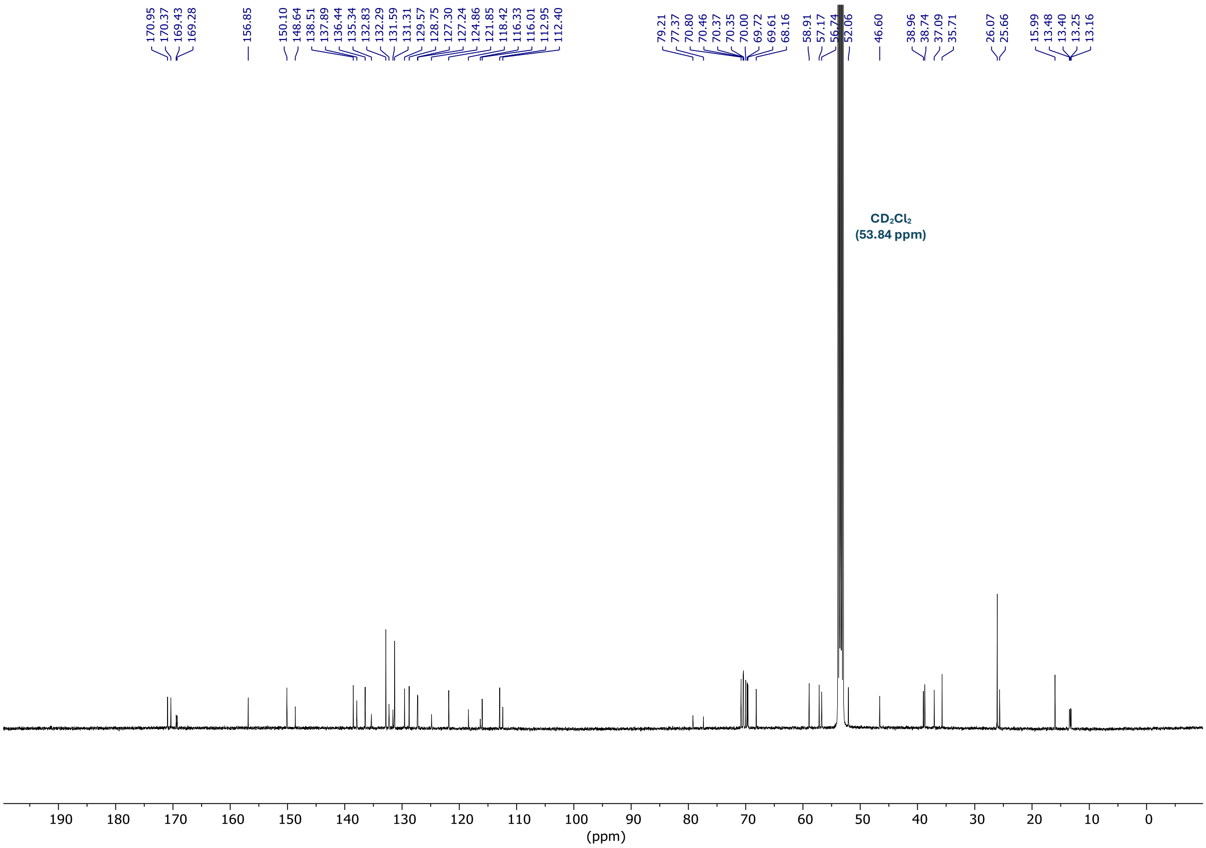

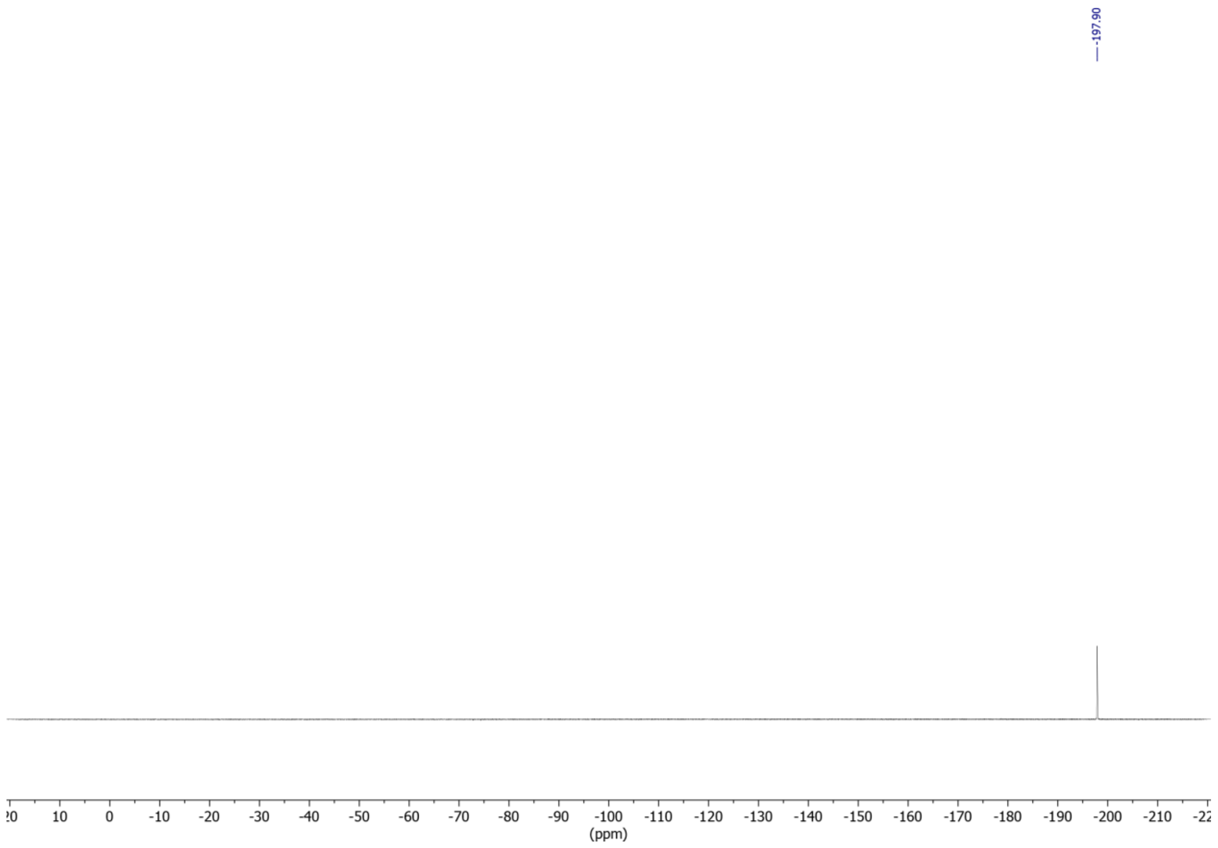
-197.90
20
10
0
-10
-20
-30
-40
-50
-60
-70
-80
-90
-100
-110
-120
-130
-140
-150
-160
-170
-180
-190
-200
-210
-22
(ppm)

Compound 14. *^{1}H NMR (500 MHz, CD_2Cl_2), ^{13}C NMR (126 MHz, CD_2Cl_2) and ^{19}F NMR (377 MHz, CD_2Cl_2)*

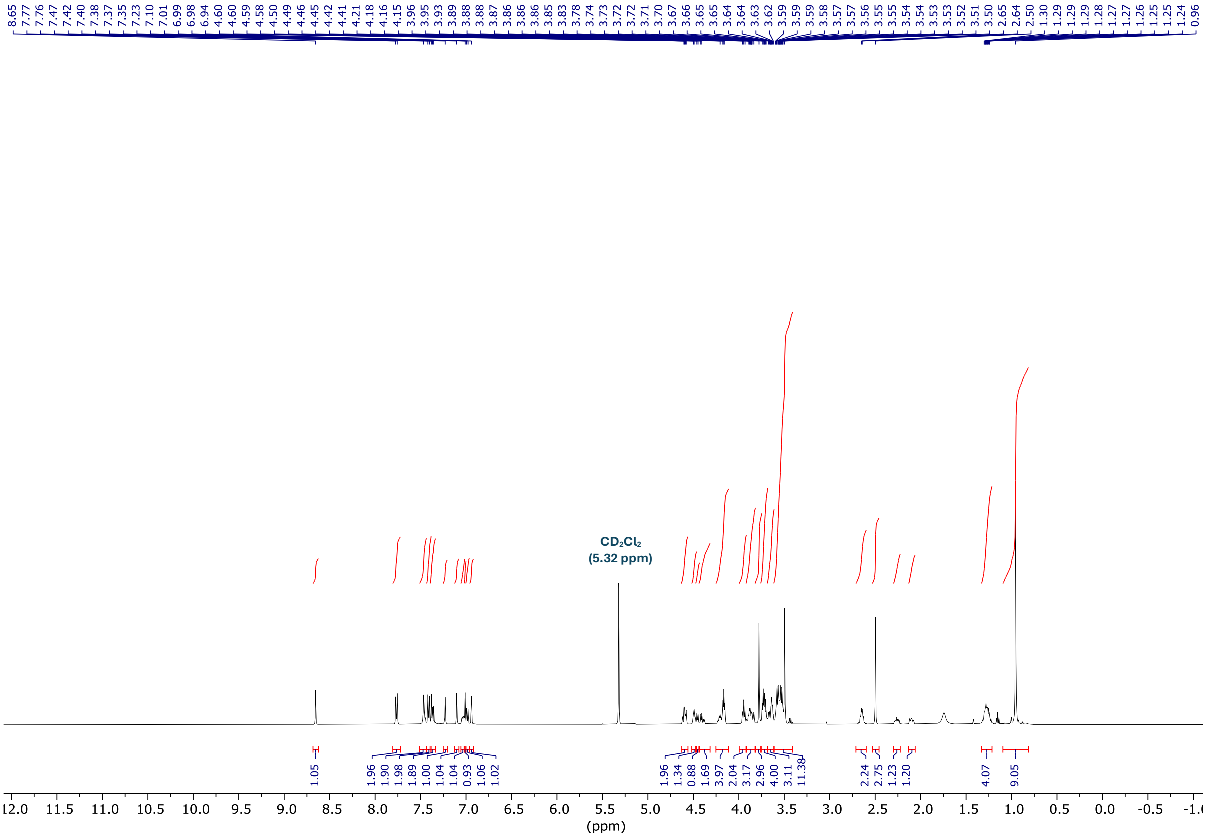

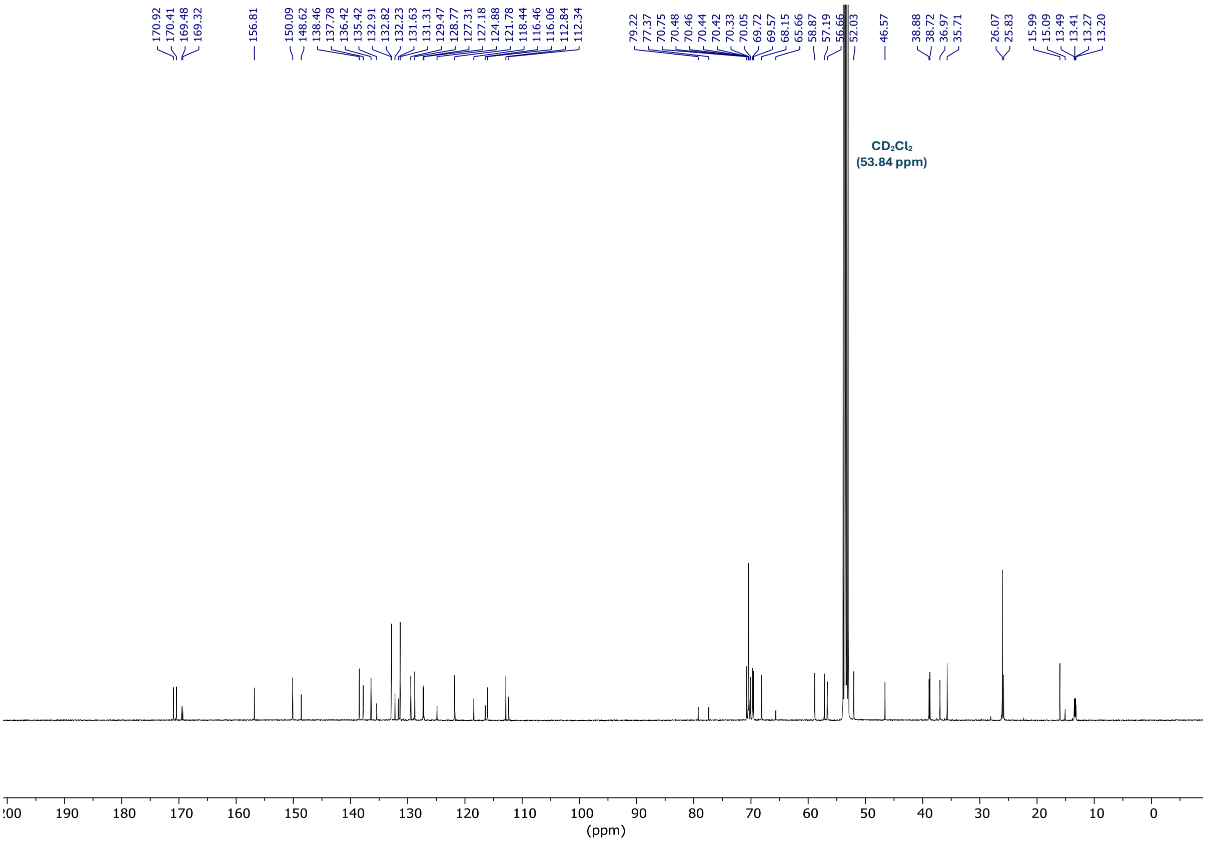

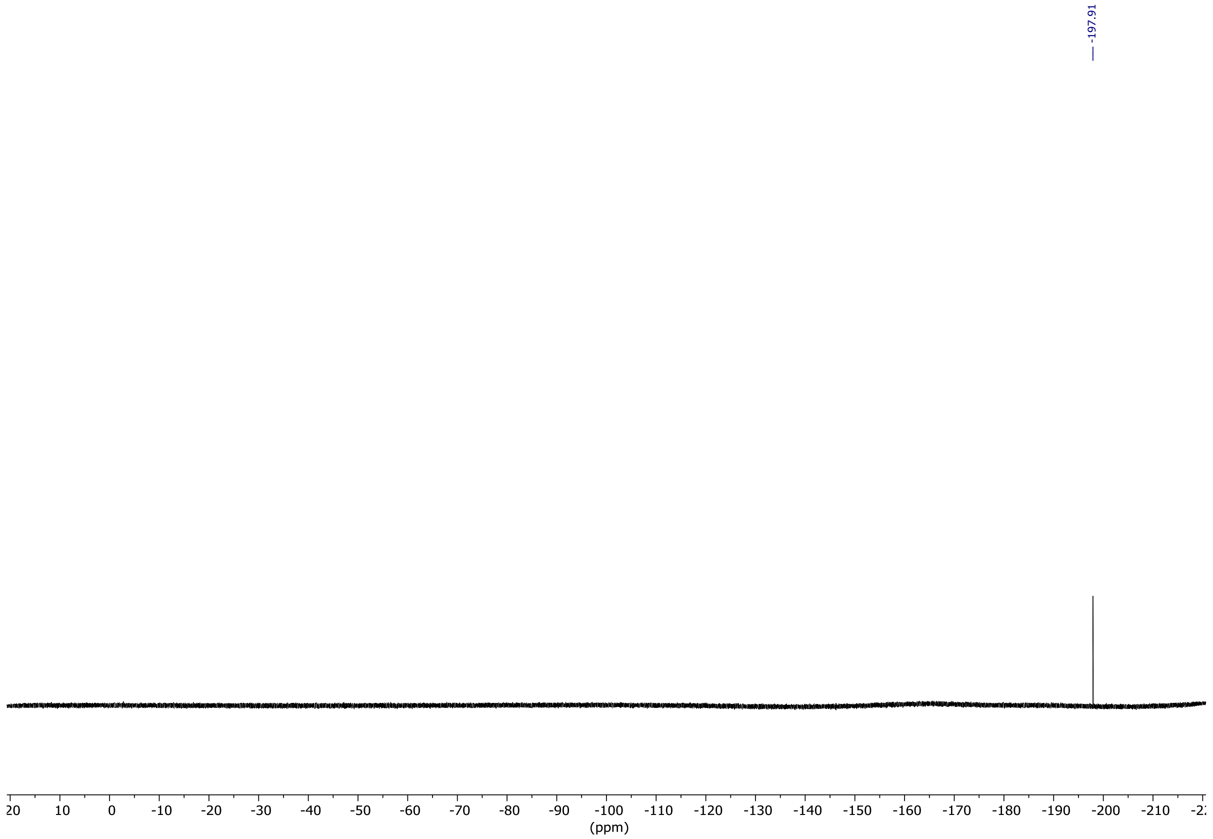
-197.91
20 10 0 -10 -20 -30 -40 -50 -60 -70 -80 -90 -100 -110 -120 -130 -140 -150 -160 -170 -180 -190 -200 -210
(ppm)

Compound 15. *^{1}H NMR (500 MHz, CD_2Cl_2) and ^{13}C NMR (126 MHz, CD_2Cl_2)*

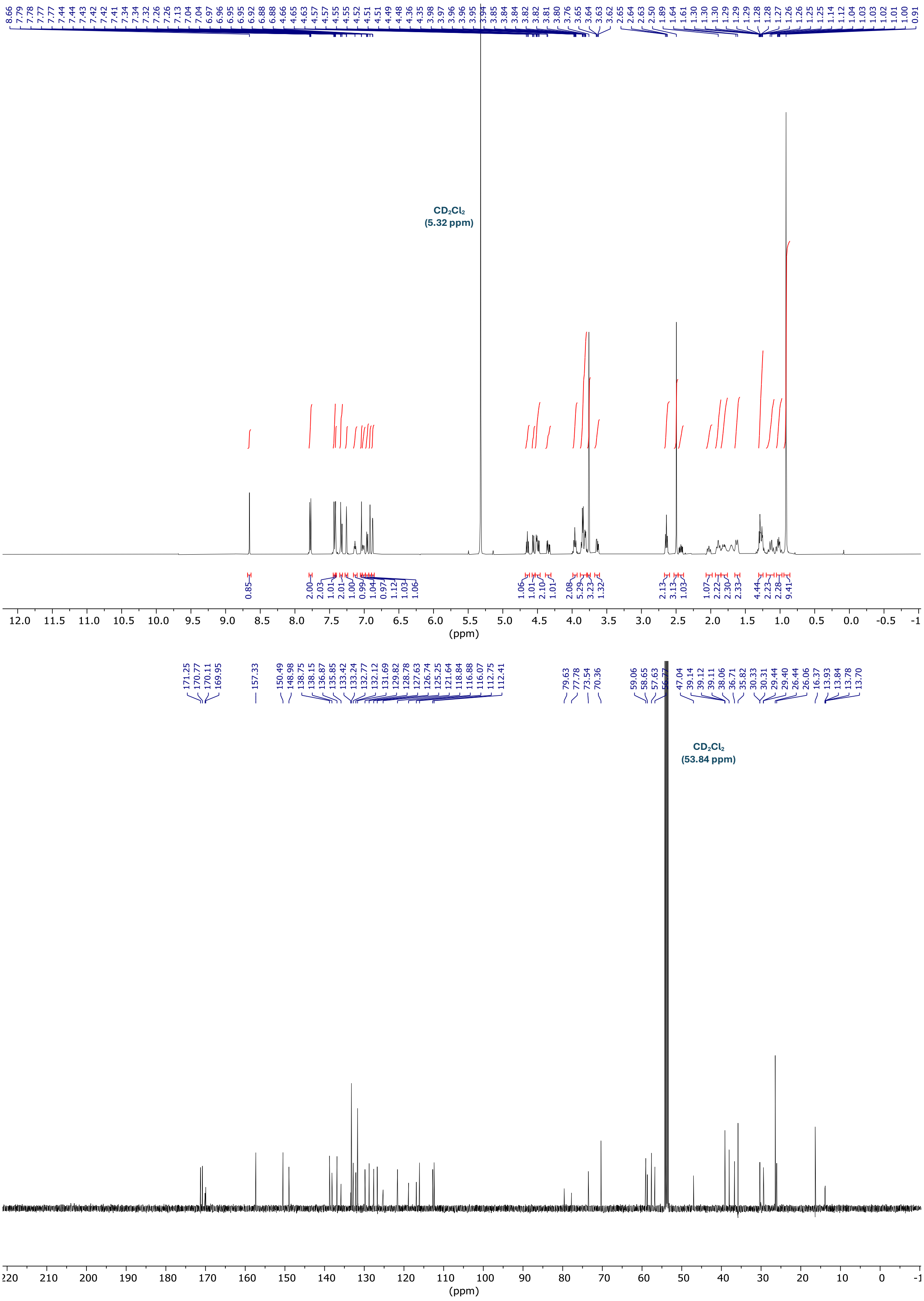

Compound 16. *^{1}H NMR (500 MHz, CD_2Cl_2), ^{13}C NMR (126 MHz, CD_2Cl_2) and ^{19}F NMR (376 MHz, CD_2Cl_2)*

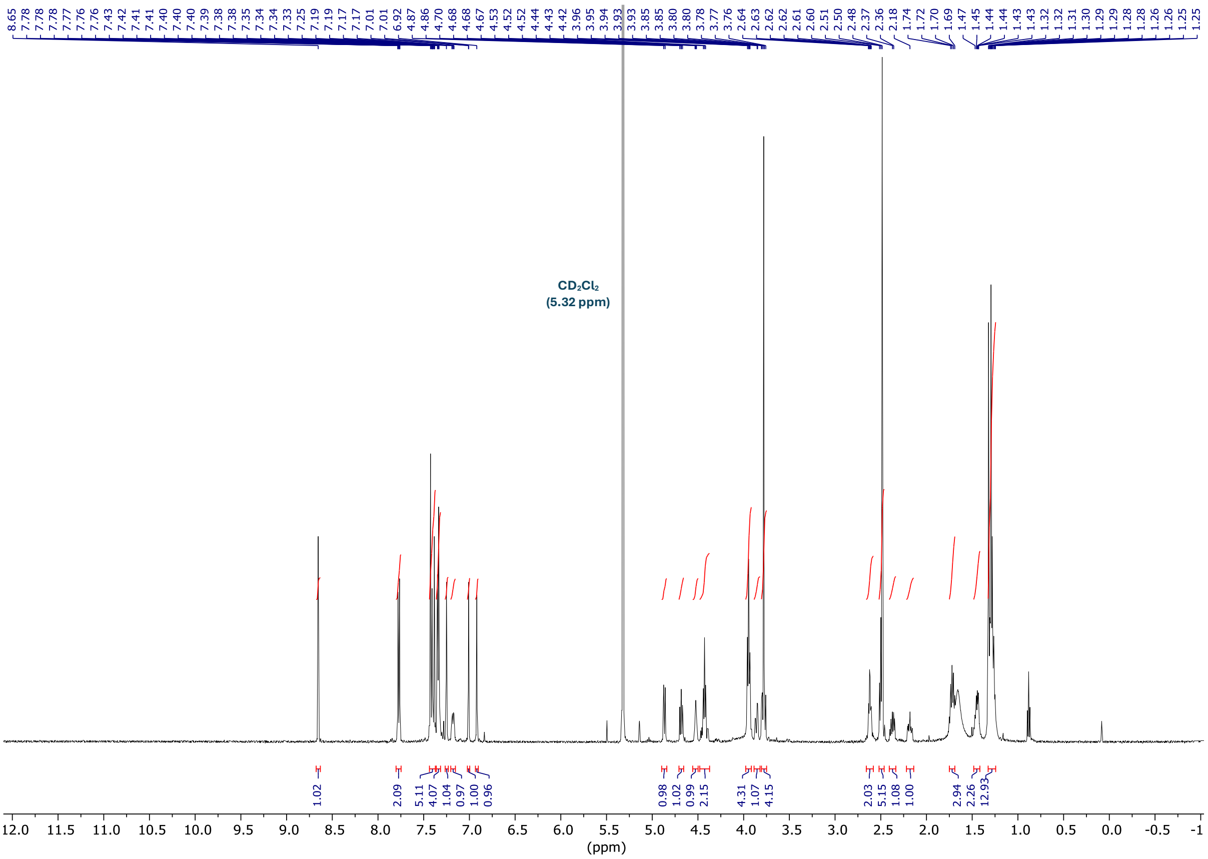

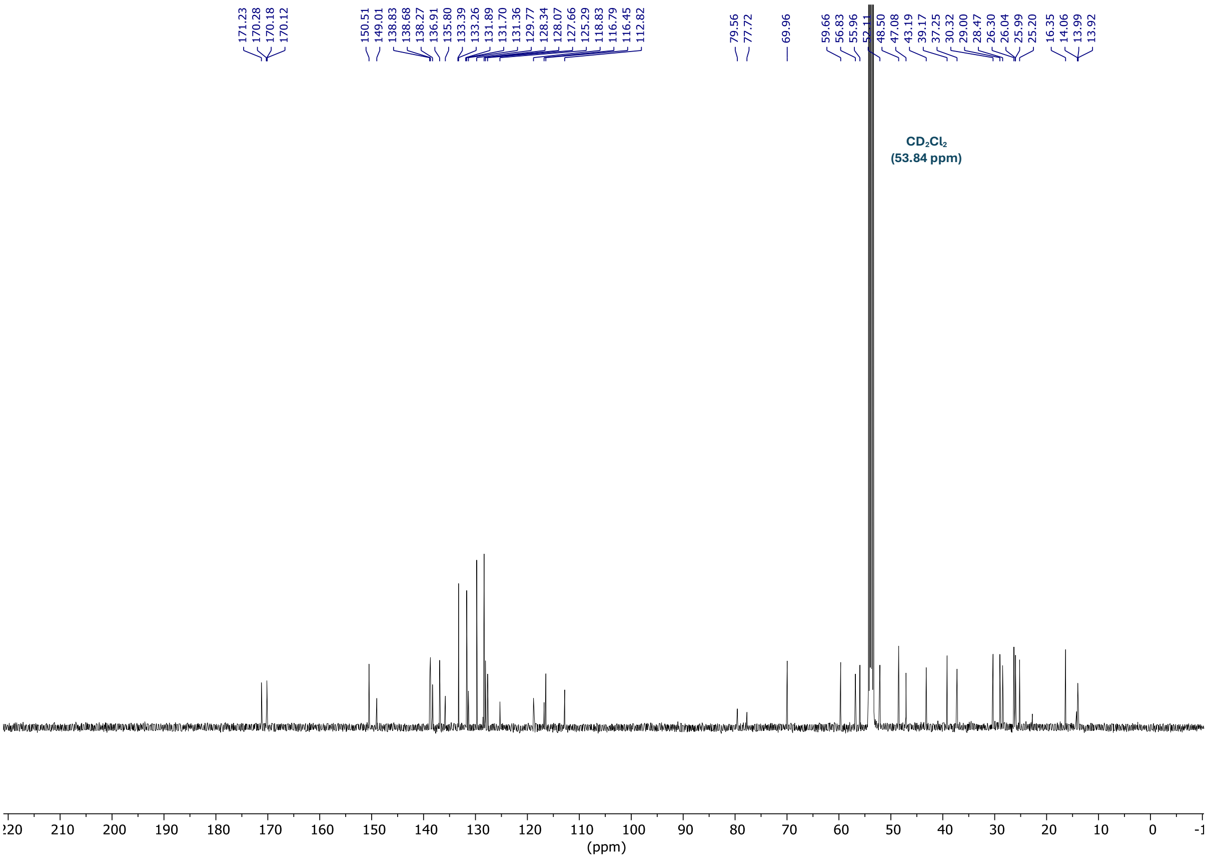

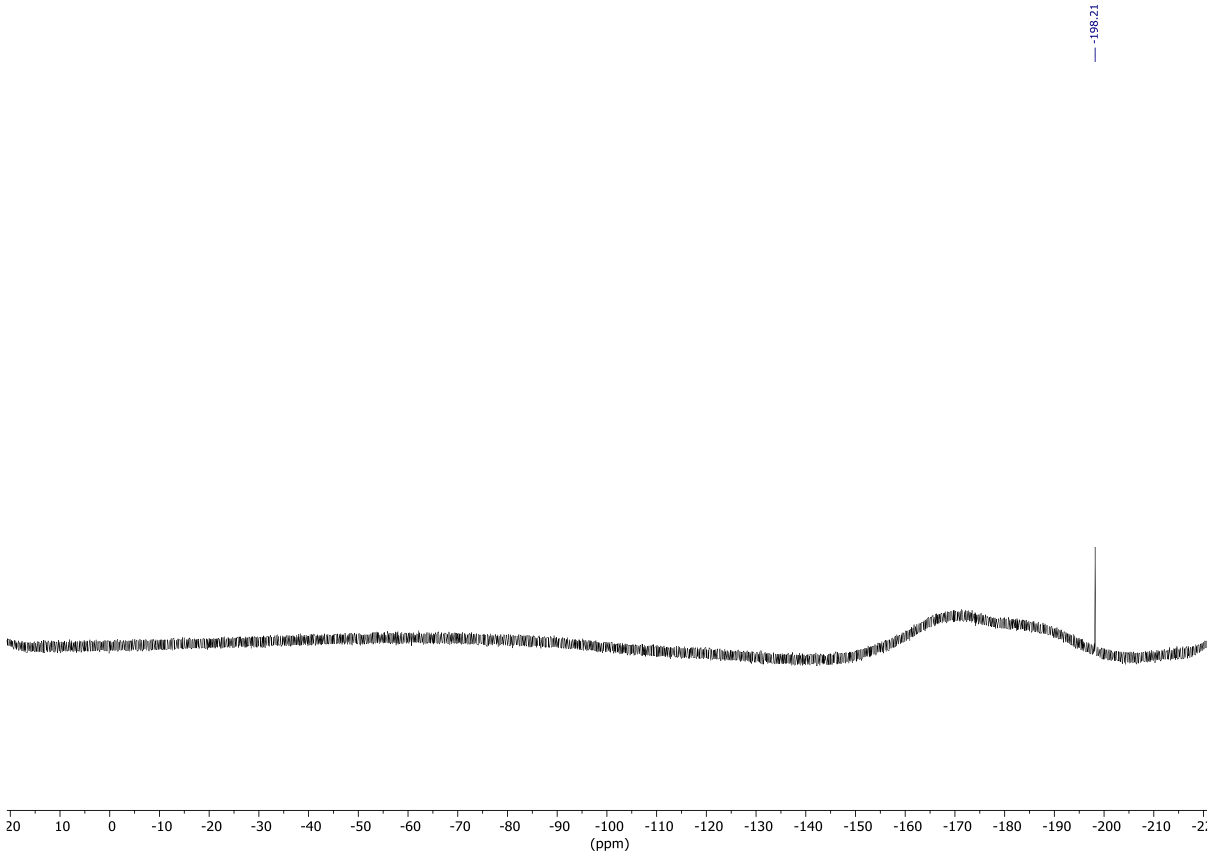

-198.21
20
10
0
-10
-20
-30
-40
-50
-60
-70
-80
-90
-100
-110
-120
-130
-140
-150
-160
-170
-180
-190
-200
-210
(ppm)

Compound 17. *^{1}H NMR (500 MHz, CD_2Cl_2), ^{13}C NMR (126 MHz, CD_2Cl_2) and ^{19}F NMR (377 MHz, CD_2Cl_2)*

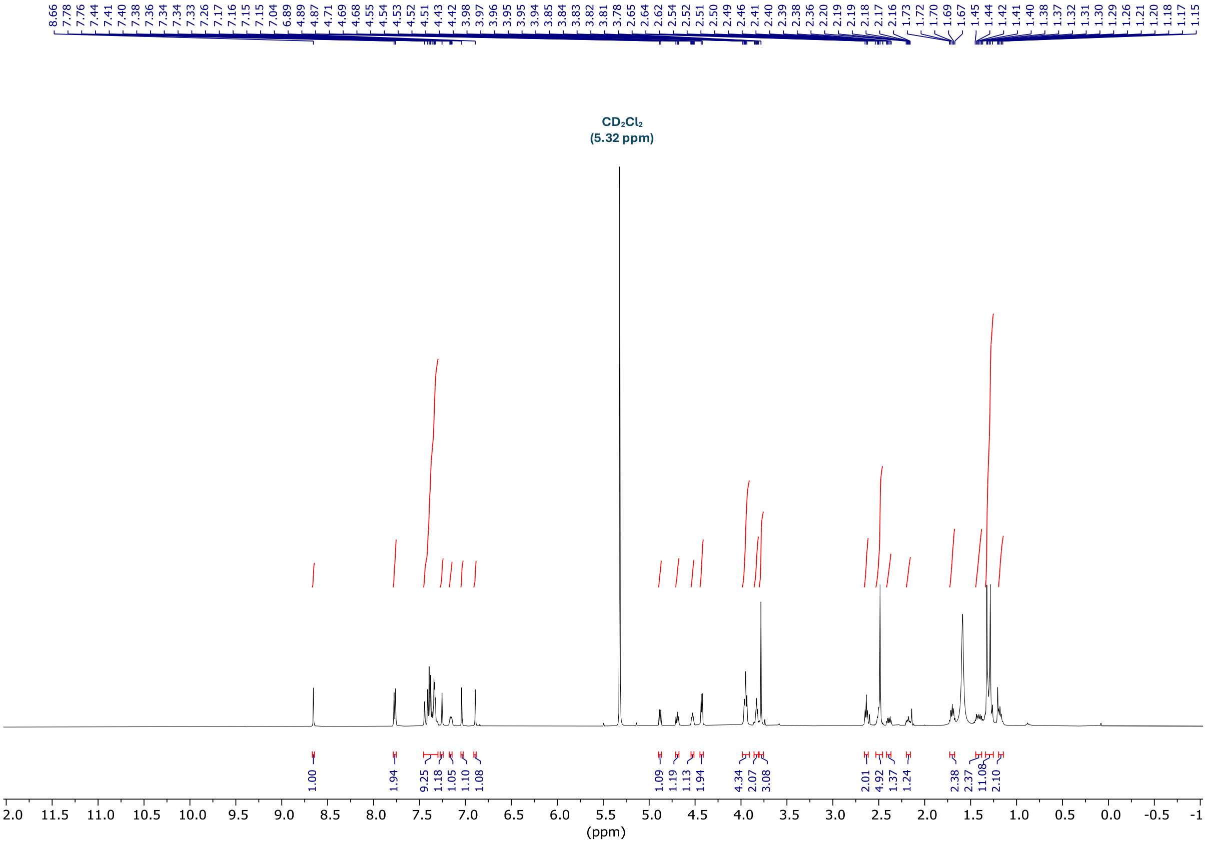

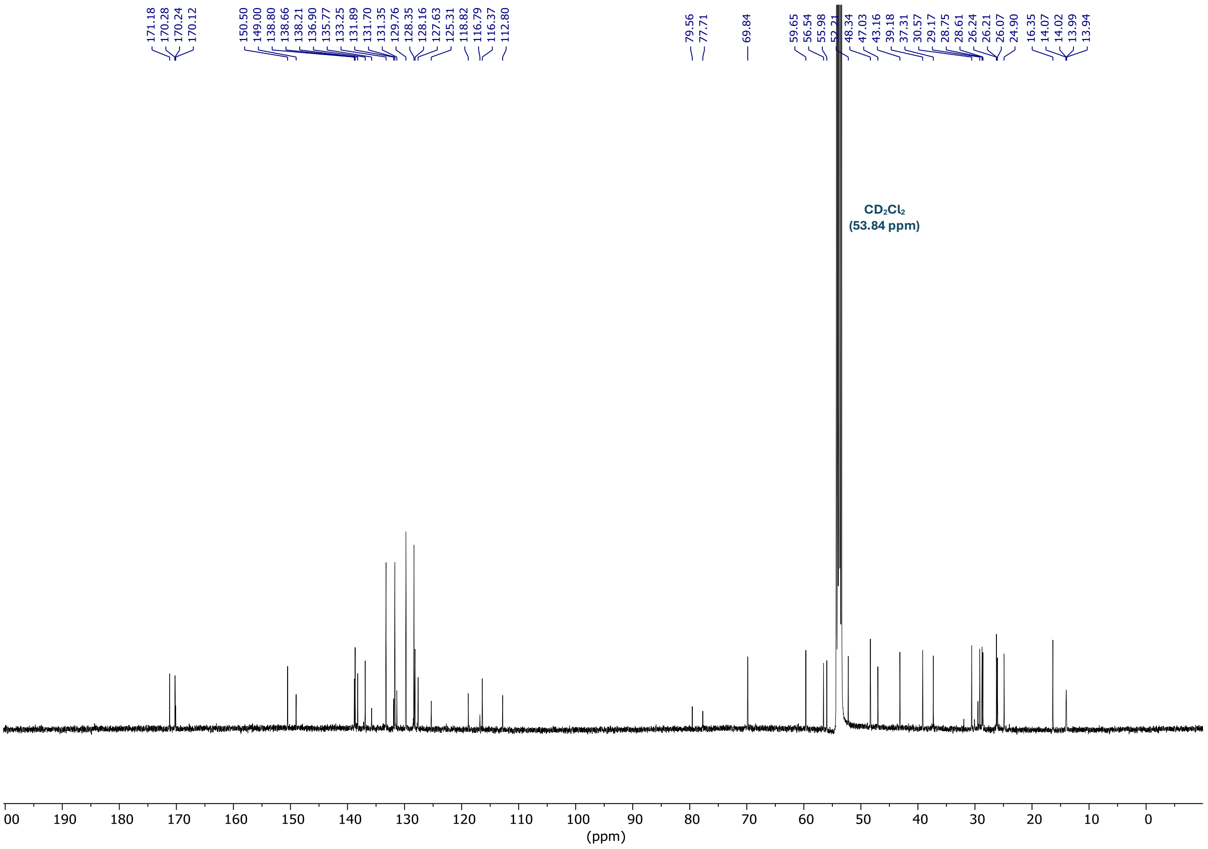

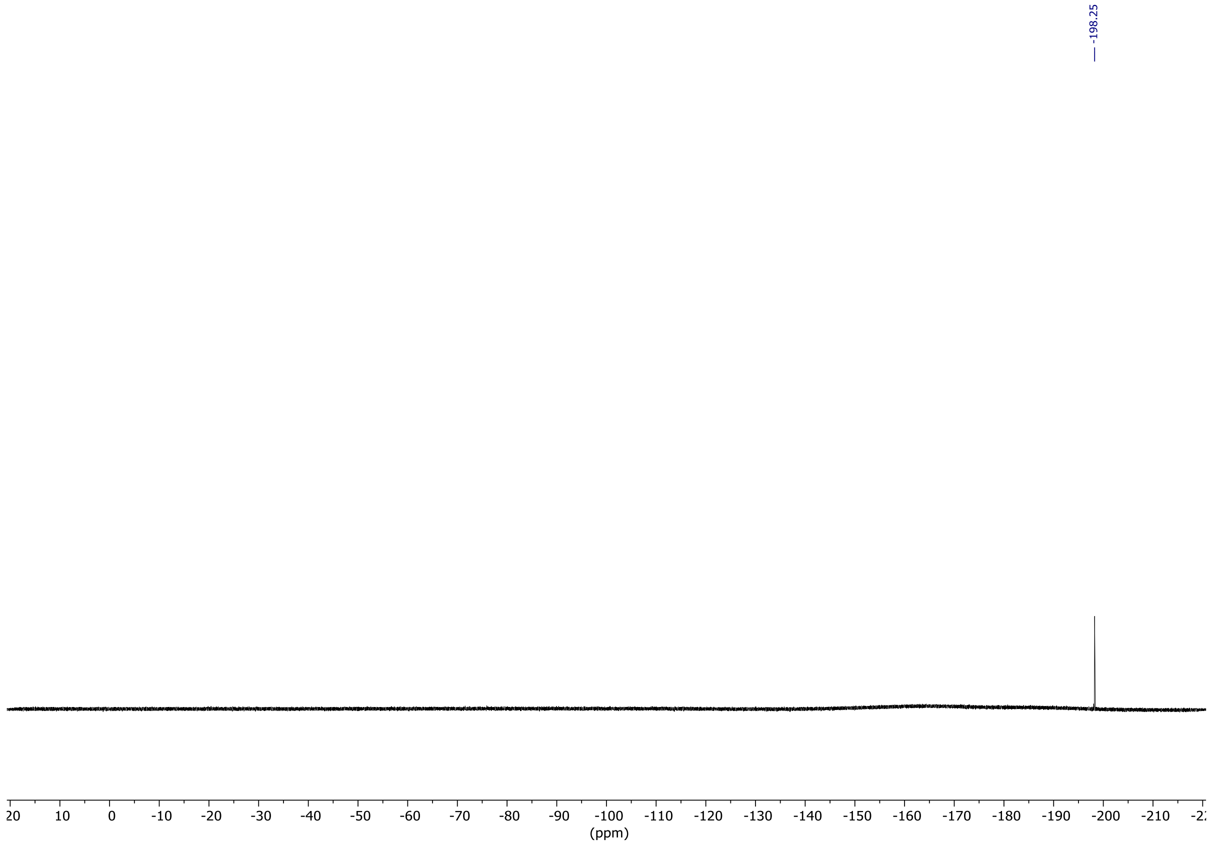
-198.25
20
10
0
-10
-20
-30
-40
-50
-60
-70
-80
-90
-100
-110
-120
-130
-140
-150
-160
-170
-180
-190
-200
-210
(ppm)

Compound 18. *^{1}H NMR (500 MHz, CD_2Cl_2), ^{13}C NMR (126 MHz, CD_2Cl_2) and ^{19}F NMR (377 MHz, CD_2Cl_2)*

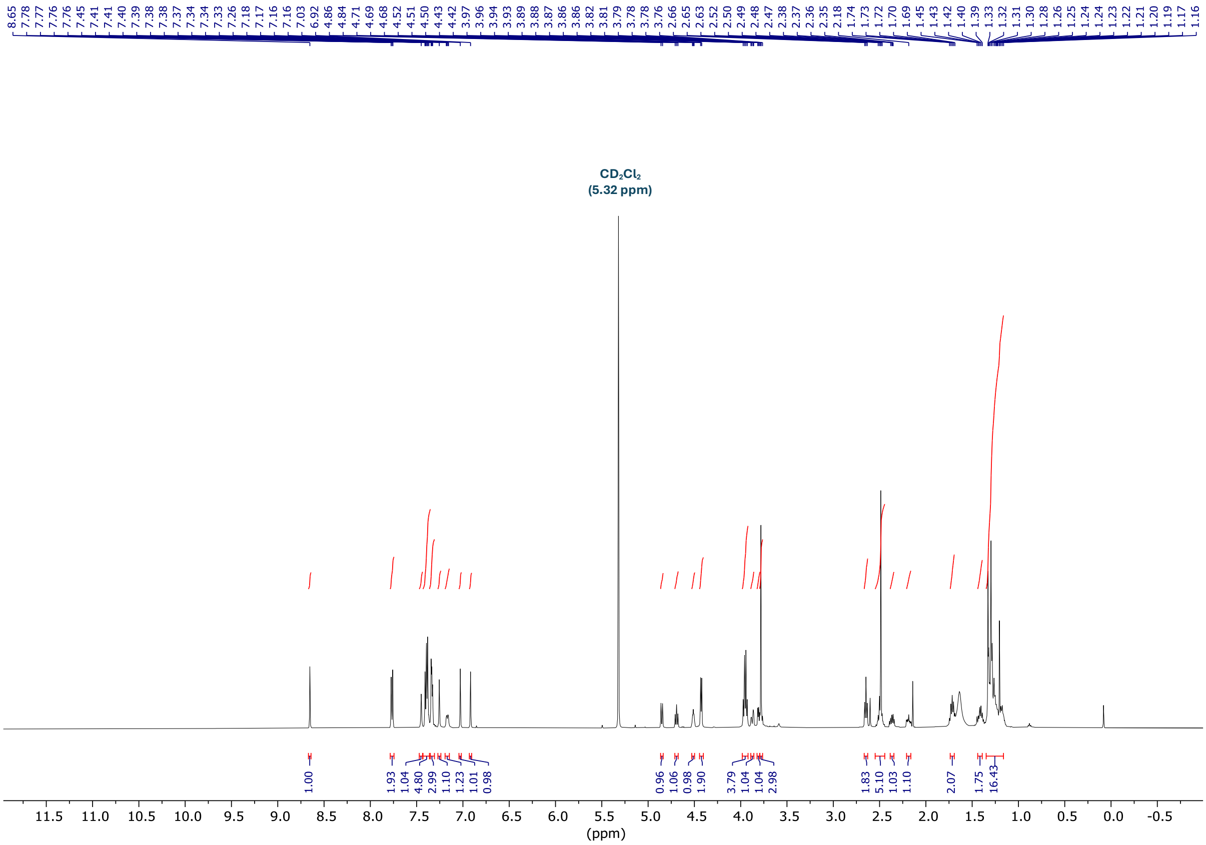

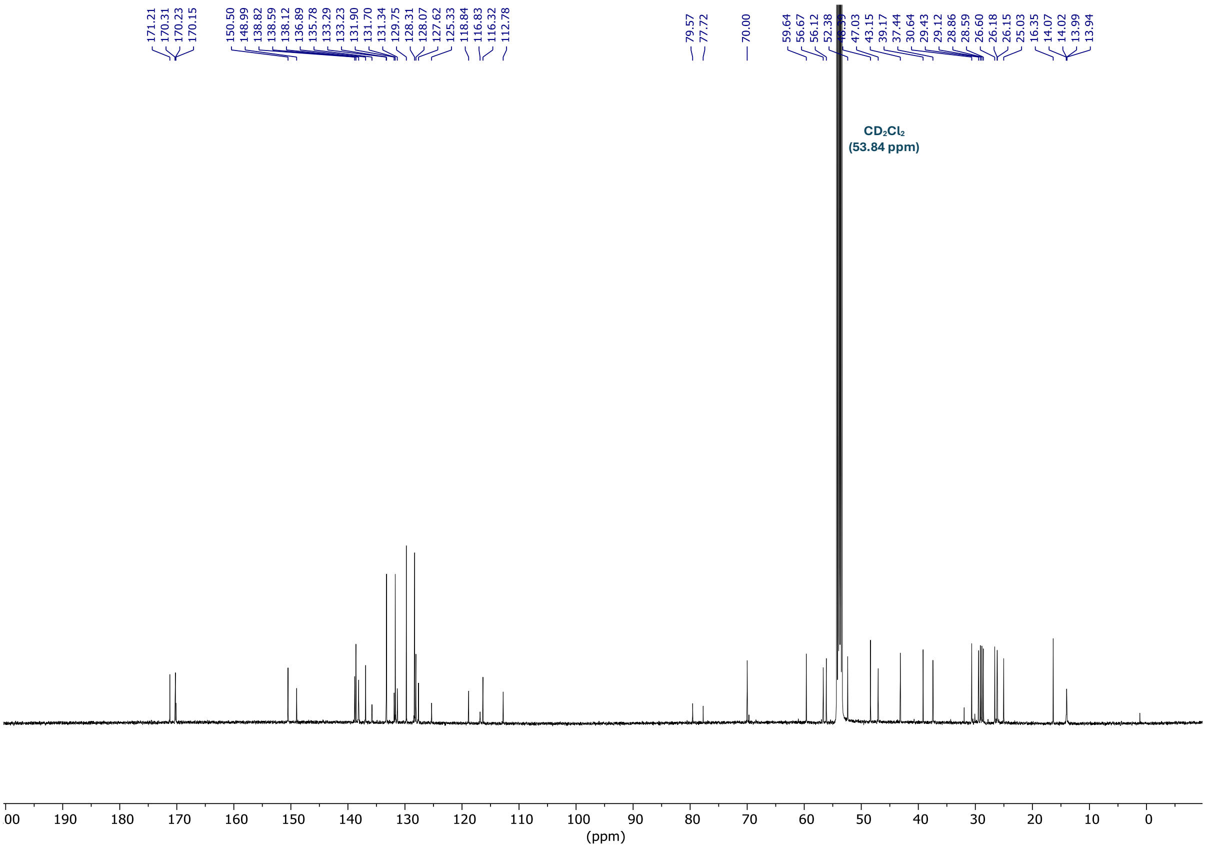

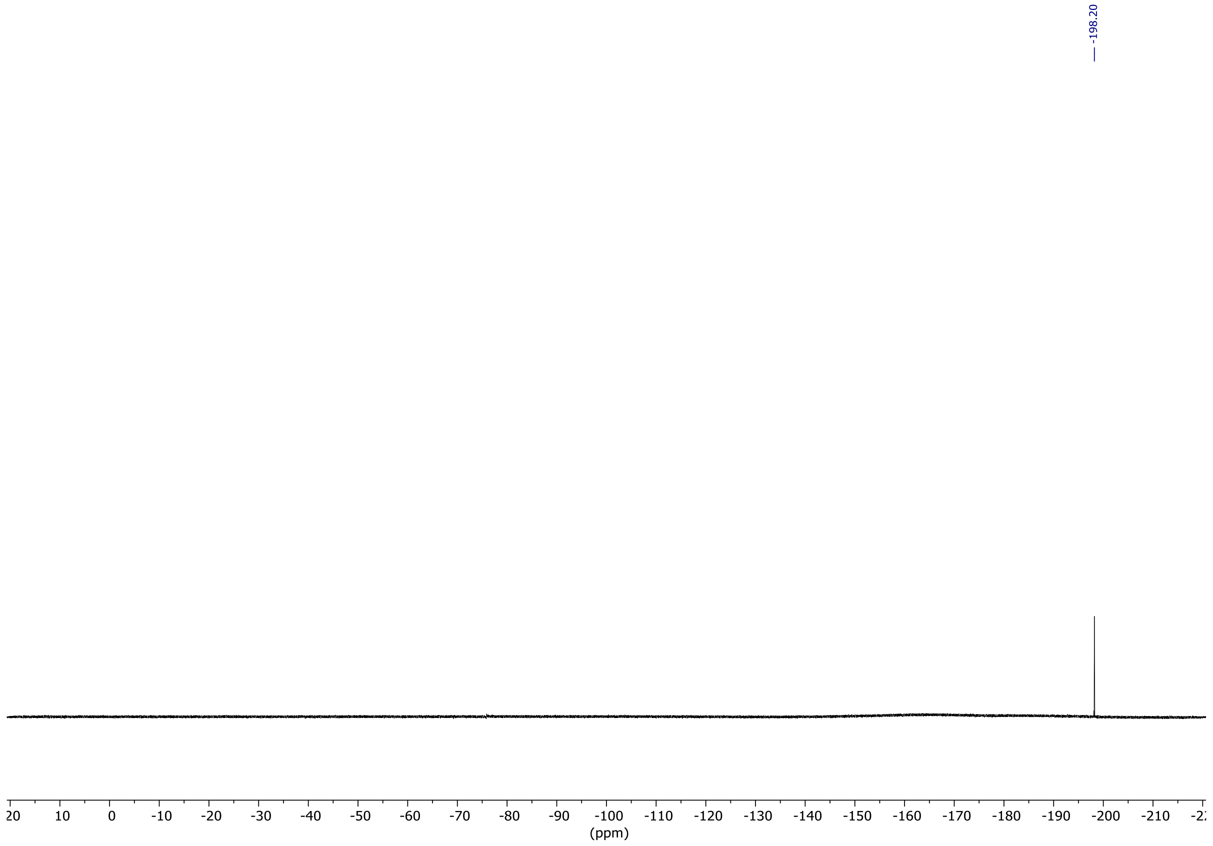
-198.20
(ppm)

Compound 19. *^{1}H NMR (500 MHz, CD_2Cl_2) and ^{13}C NMR (126 MHz, CD_2Cl_2)*

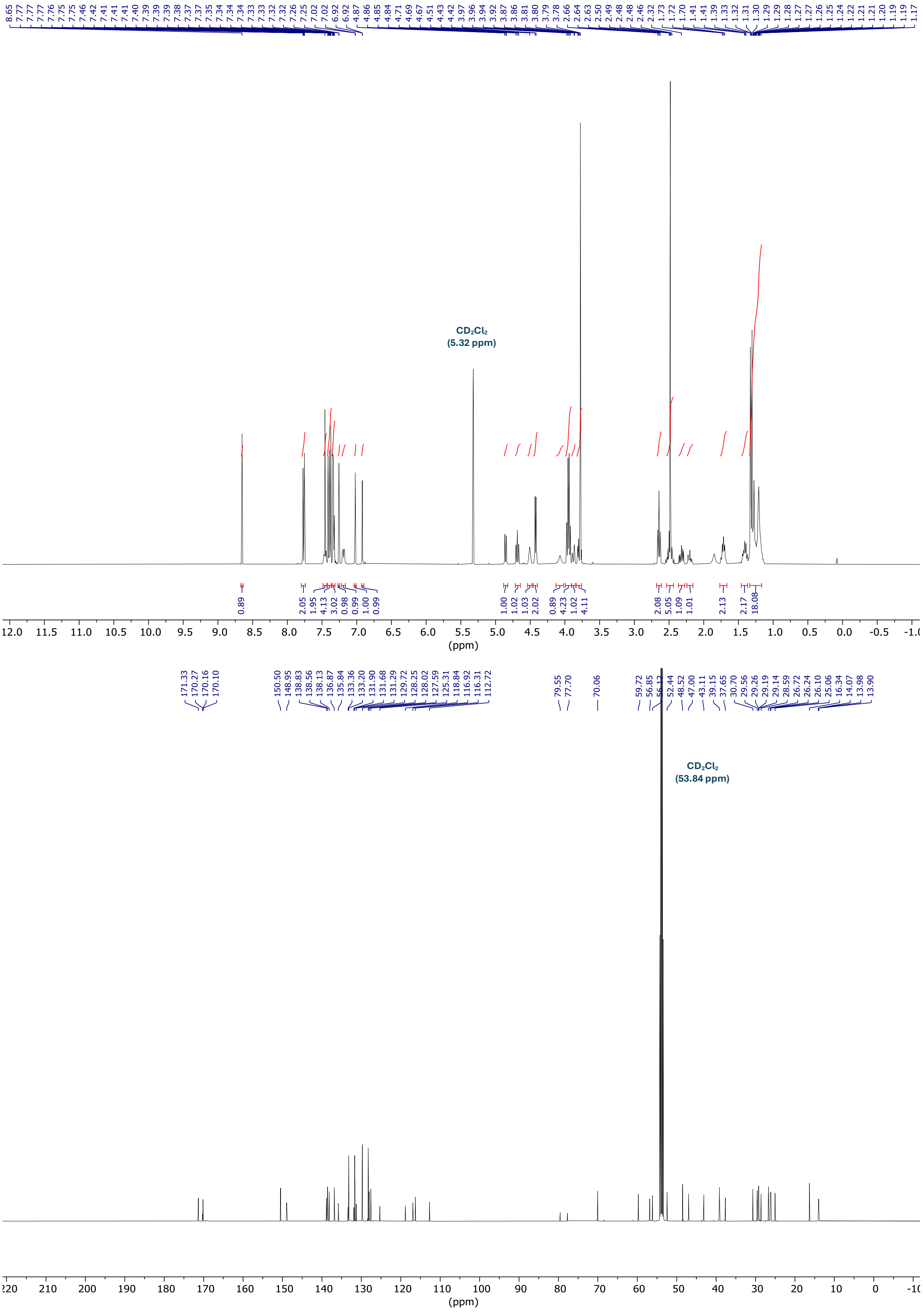

Compound 20. *^{1}H NMR (500 MHz, CD_2Cl_2), ^{13}C NMR (126 MHz, CD_2Cl_2) and ^{19}F NMR (377 MHz, CD_2Cl_2)*

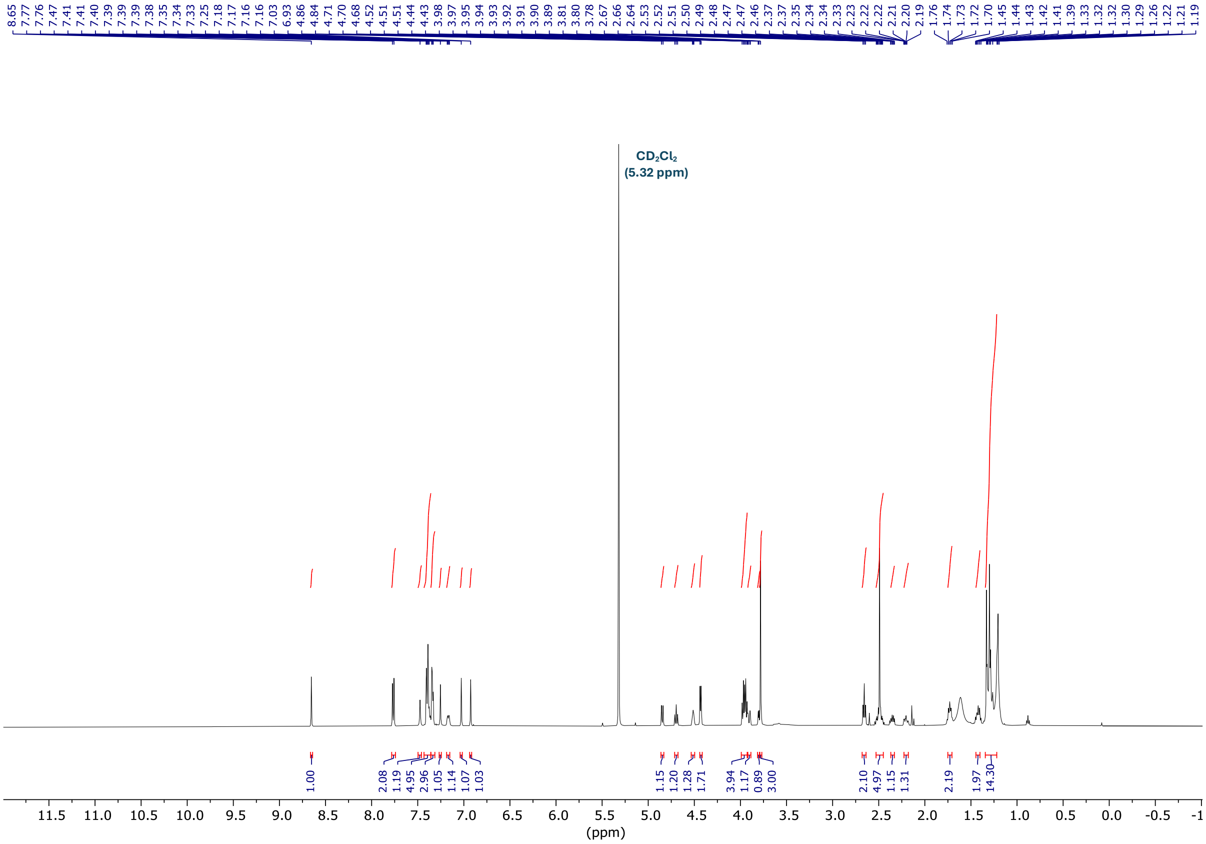

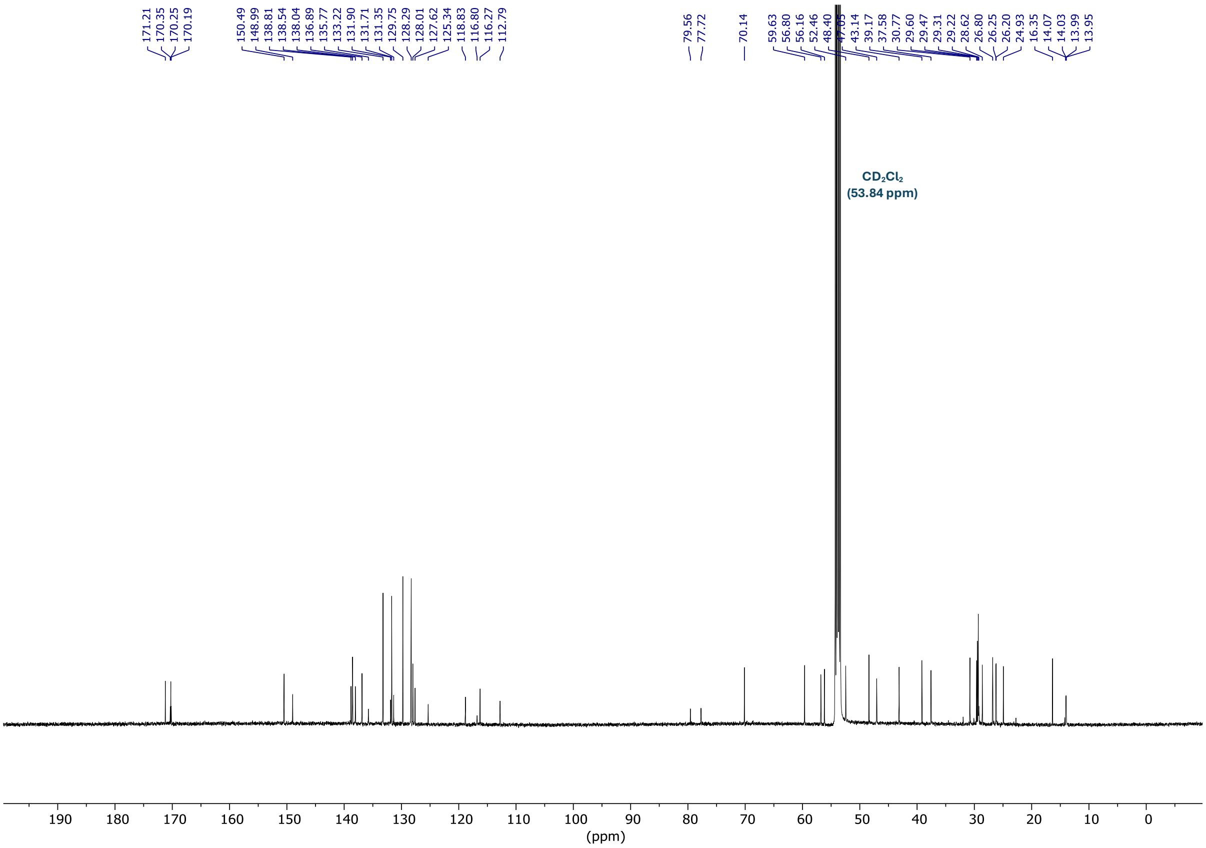

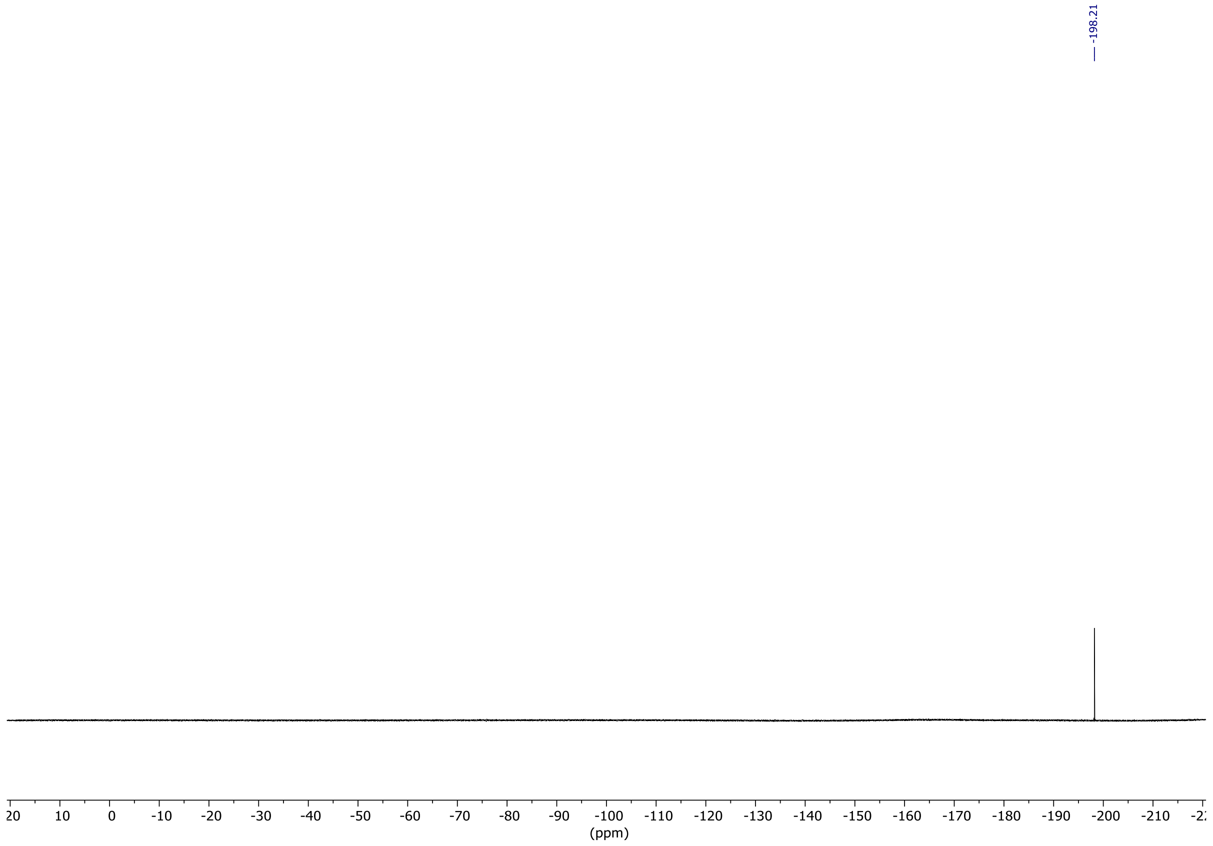
-198.21
20 10 0 -10 -20 -30 -40 -50 -60 -70 -80 -90 -100 -110 -120 -130 -140 -150 -160 -170 -180 -190 -200 -210
(ppm)

Compound 21. *^1H NMR (500 MHz, CD_2Cl_2), ^{13}C NMR (126 MHz, CD_2Cl_2) and ^{19}F NMR (376 MHz, CD_2Cl_2)*

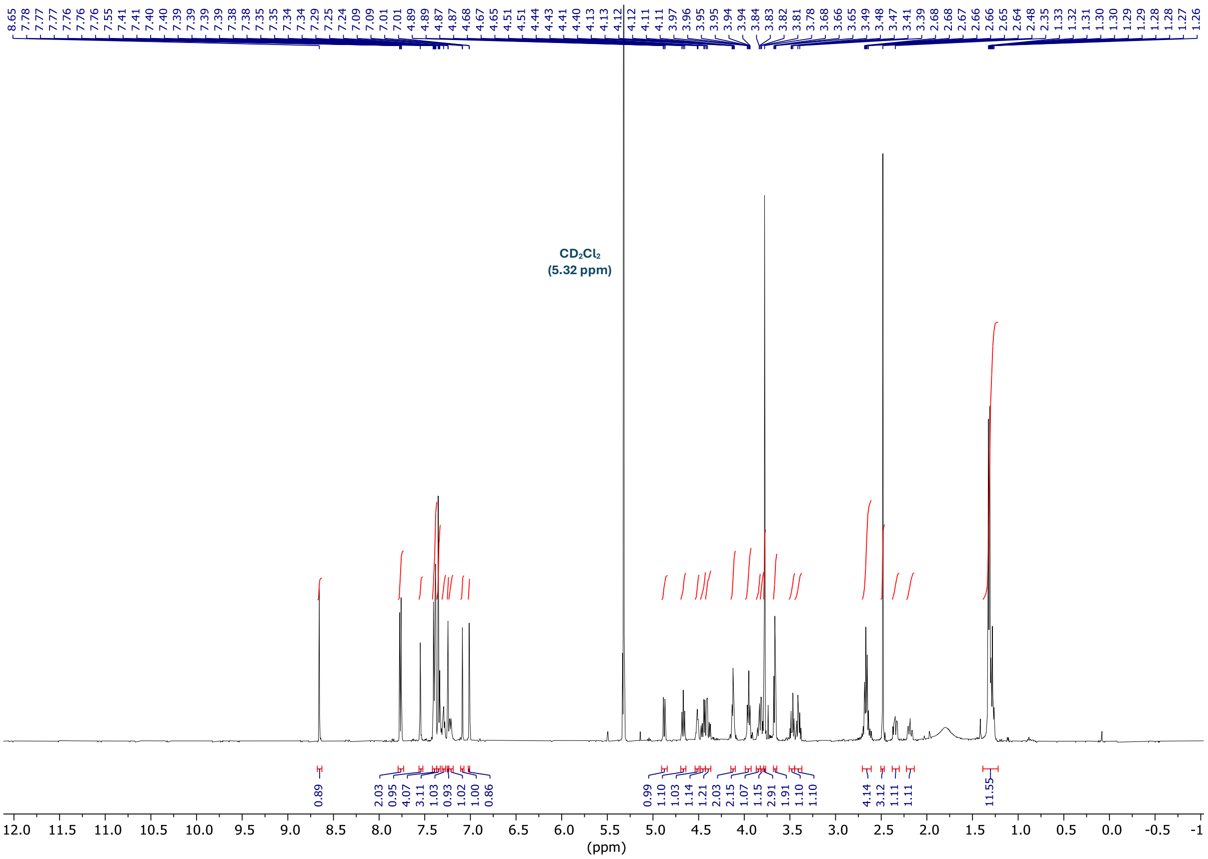

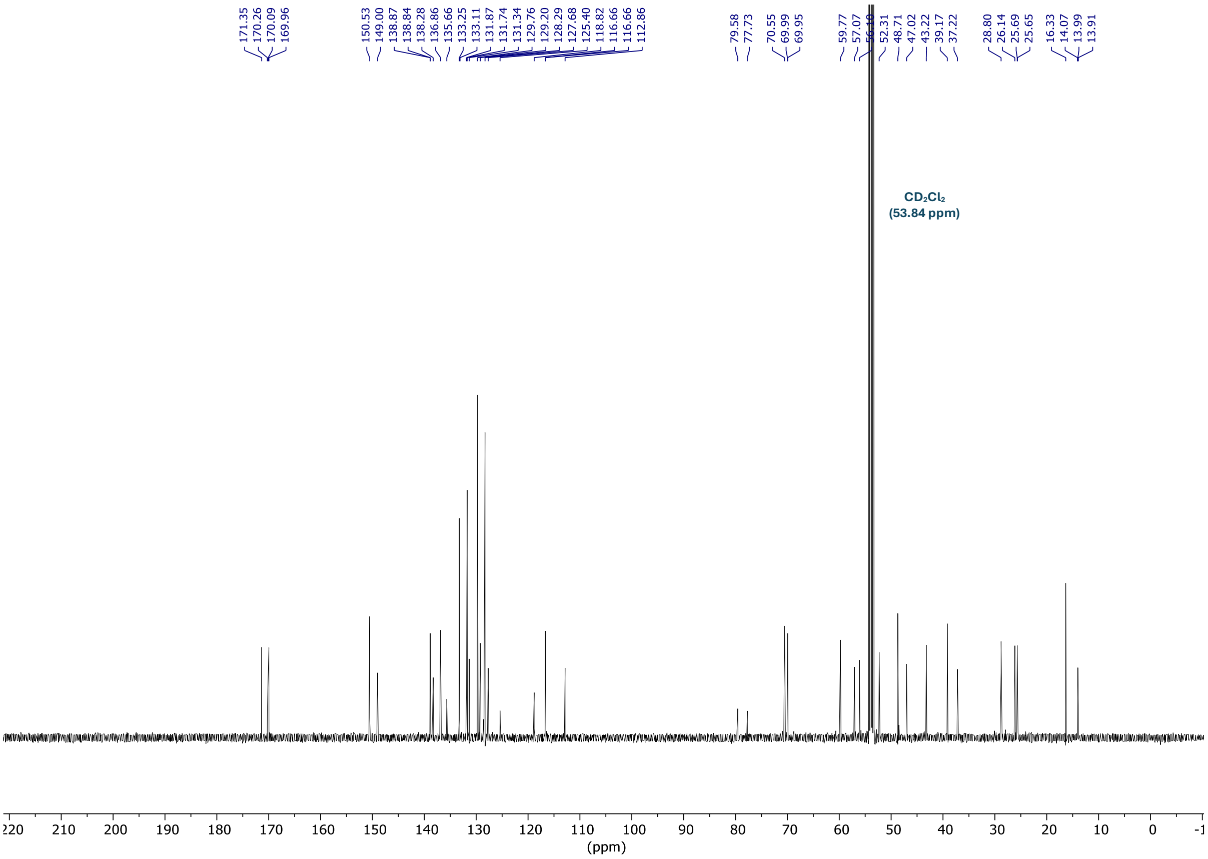

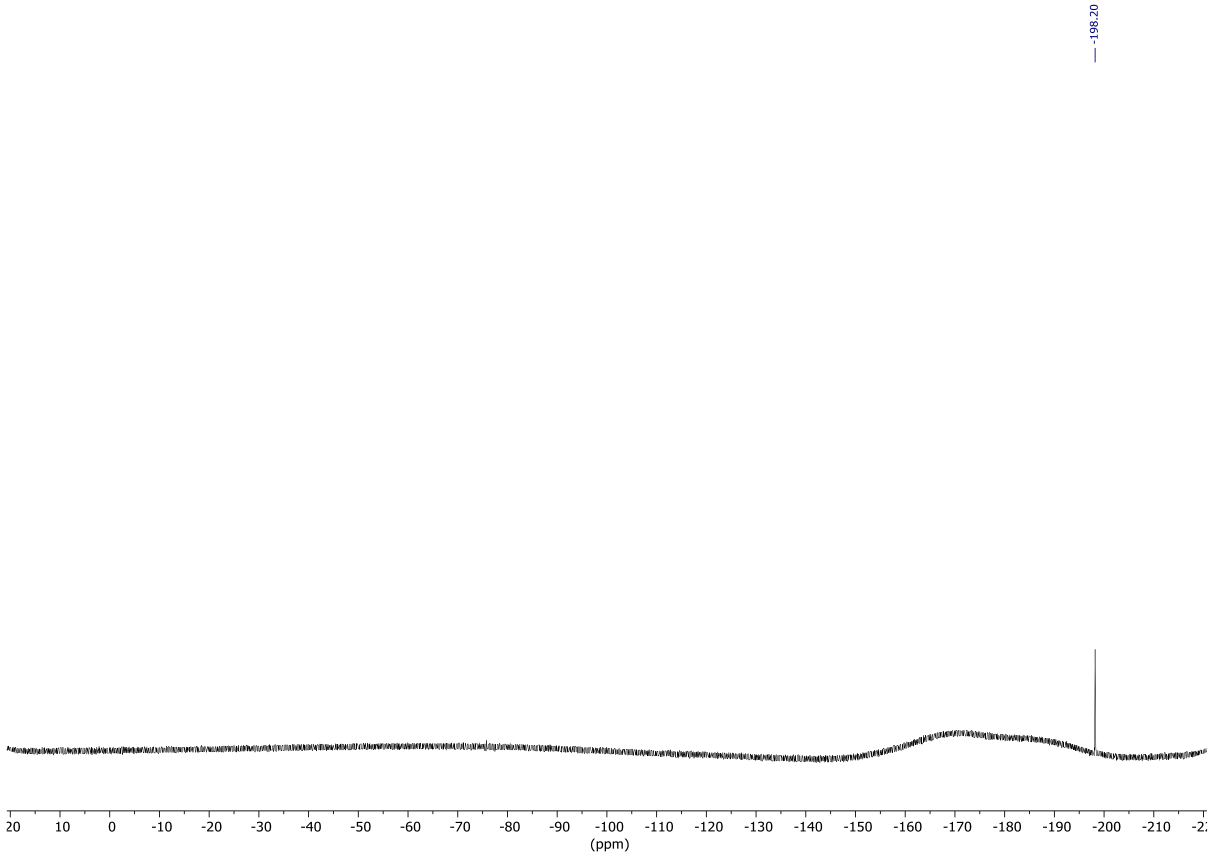
-198.20
20 10 0 -10 -20 -30 -40 -50 -60 -70 -80 -90 -100 -110 -120 -130 -140 -150 -160 -170 -180 -190 -200 -210
(ppm)

Compound 22. *^{1}H NMR (500 MHz, CD_2Cl_2) and ^{13}C NMR (126 MHz, CD_2Cl_2)*

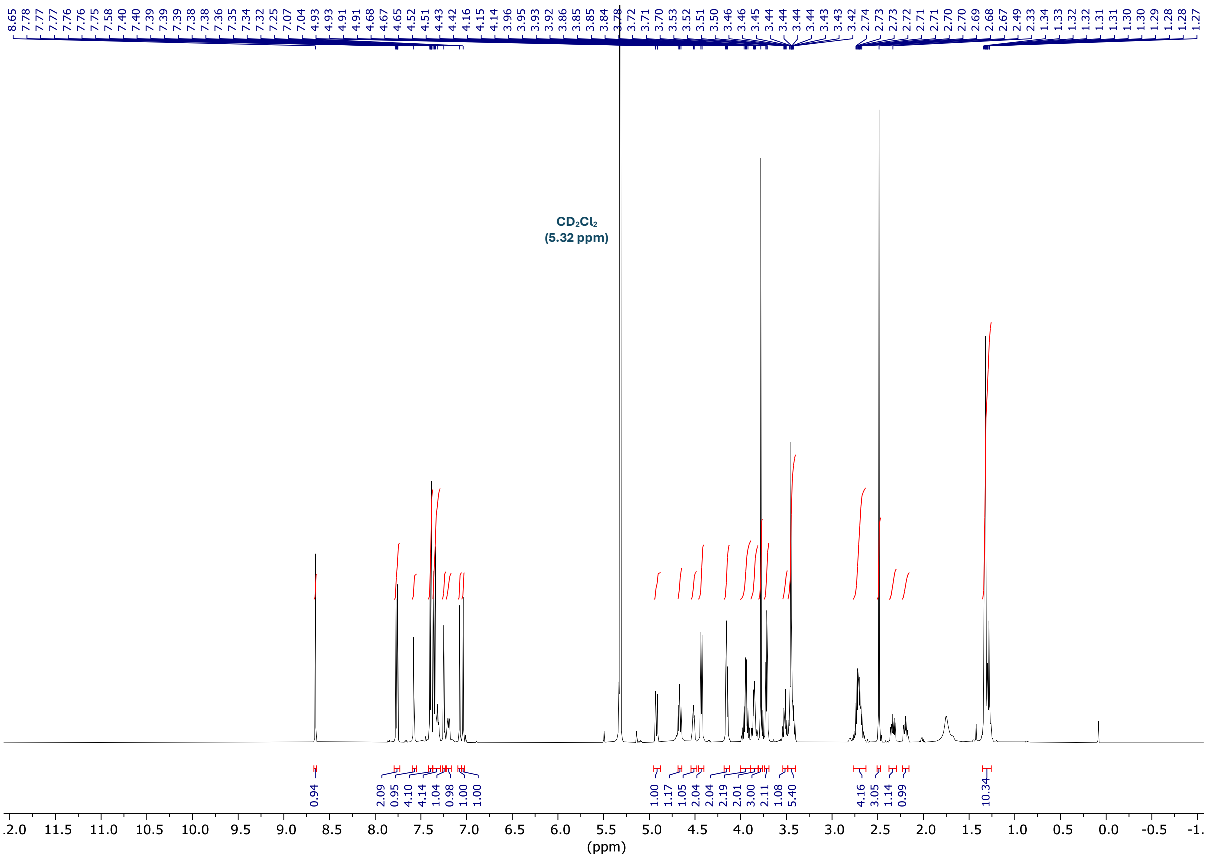

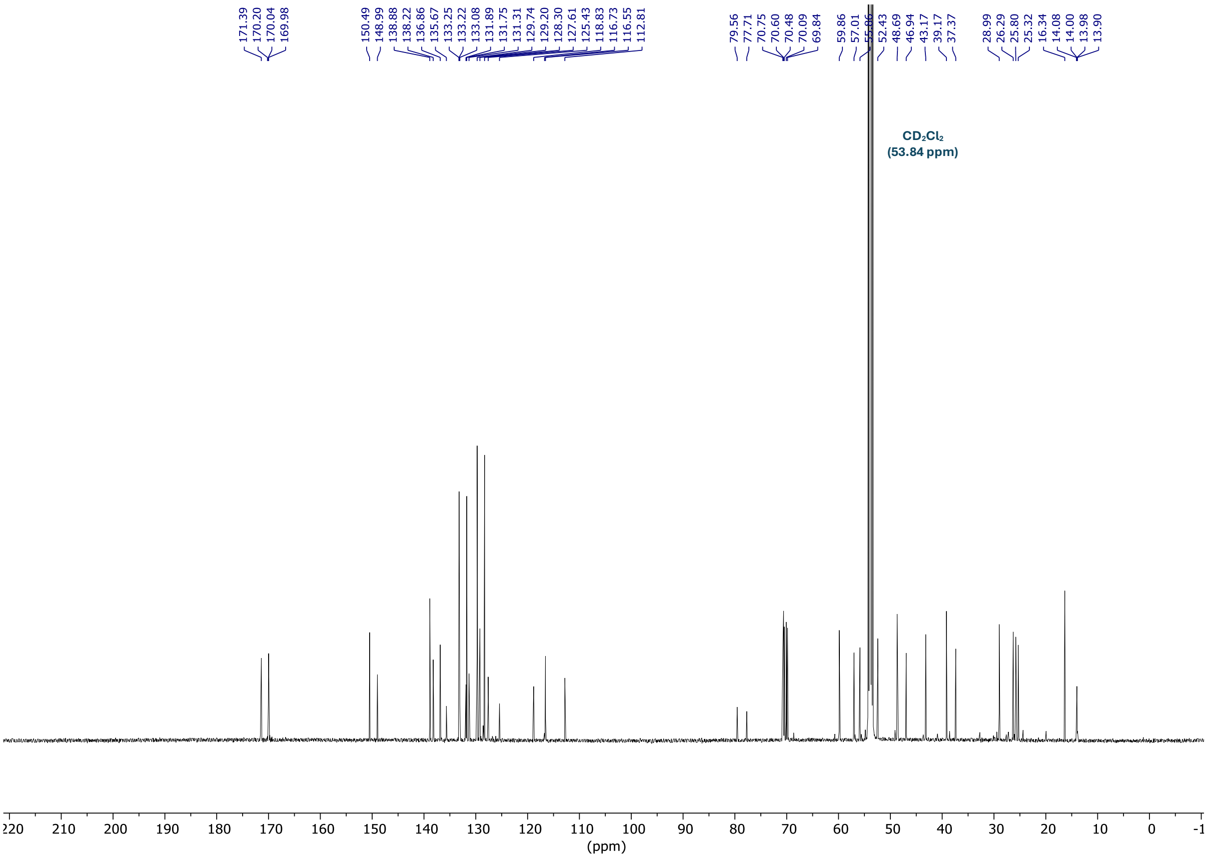

Compound 23. *¹H NMR (500 MHz, CD_2Cl_2), ¹³C NMR (126 MHz, CD_2Cl_2) and ¹⁹F NMR (376 MHz, CD_2Cl_2).*

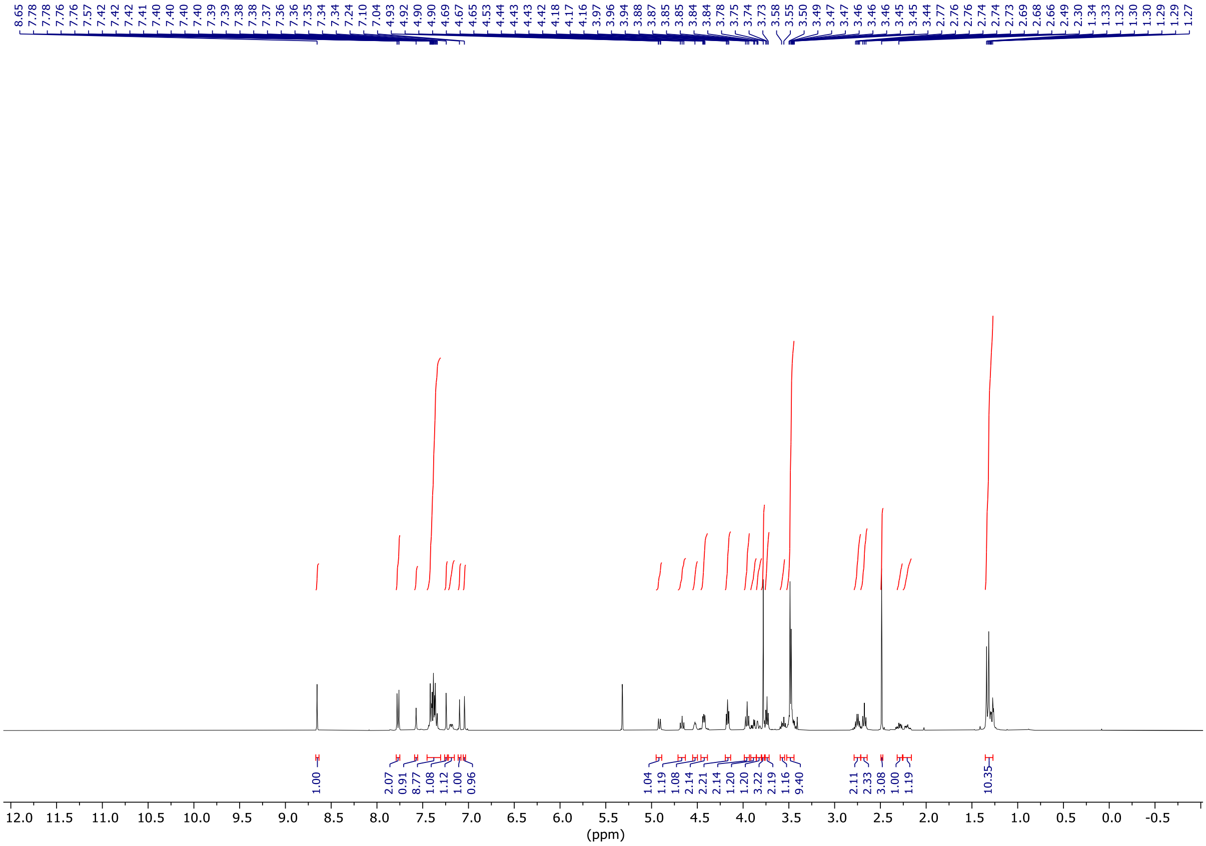

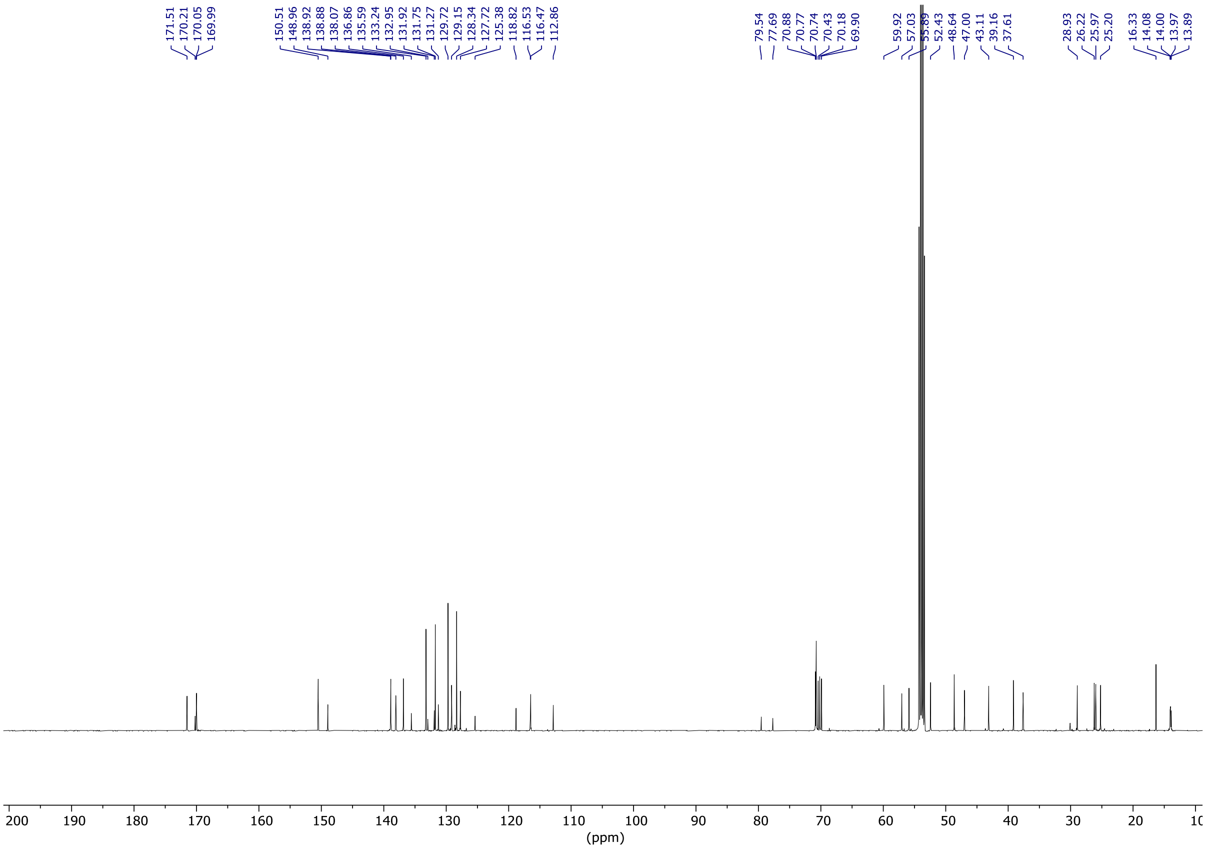

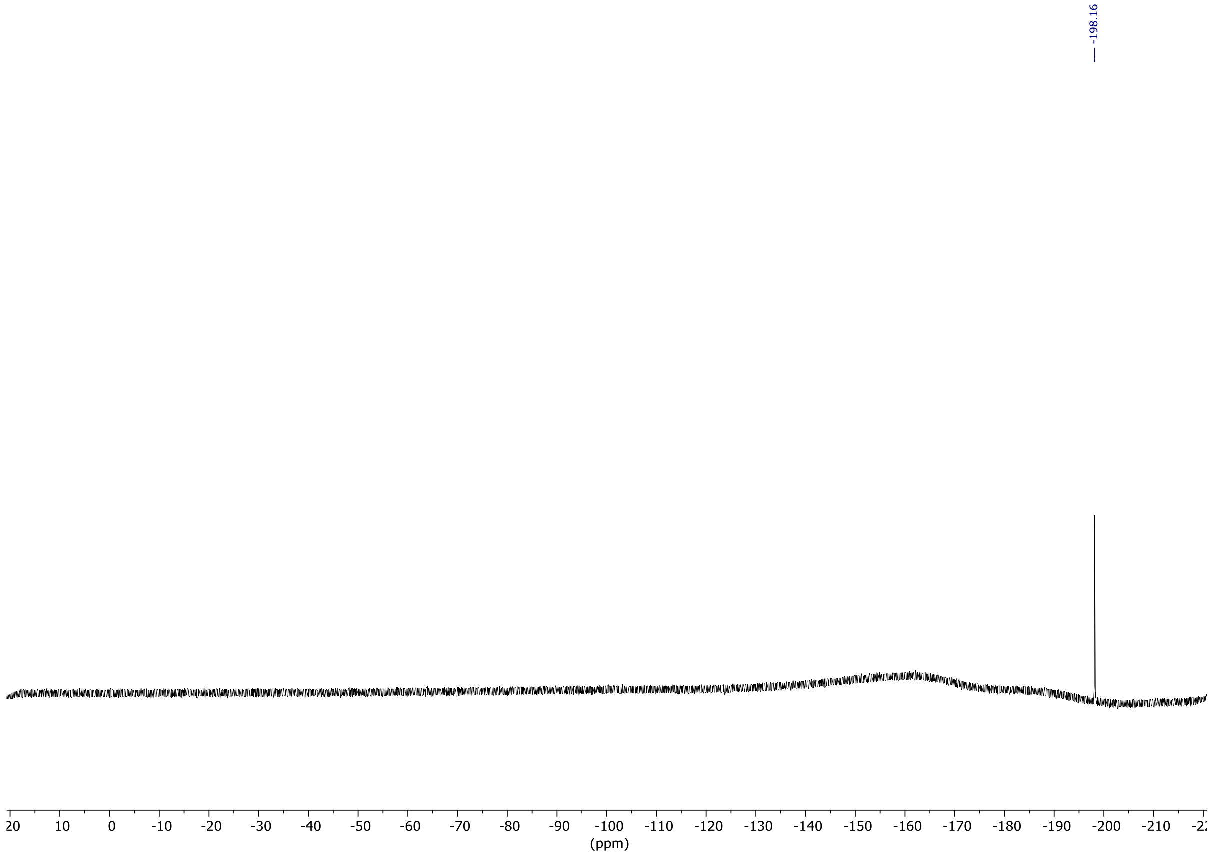

-198.16
20
10
0
-10
-20
-30
-40
-50
-60
-70
-80
-90
-100
-110
-120
-130
-140
-150
-160
-170
-180
-190
-200
-210
(ppm)

Compound 24. *^{1}H NMR (500 MHz, CD_2Cl_2), ^{13}C NMR (126 MHz, CD_2Cl_2) and ^{19}F NMR (376 MHz, CD_2Cl_2)*

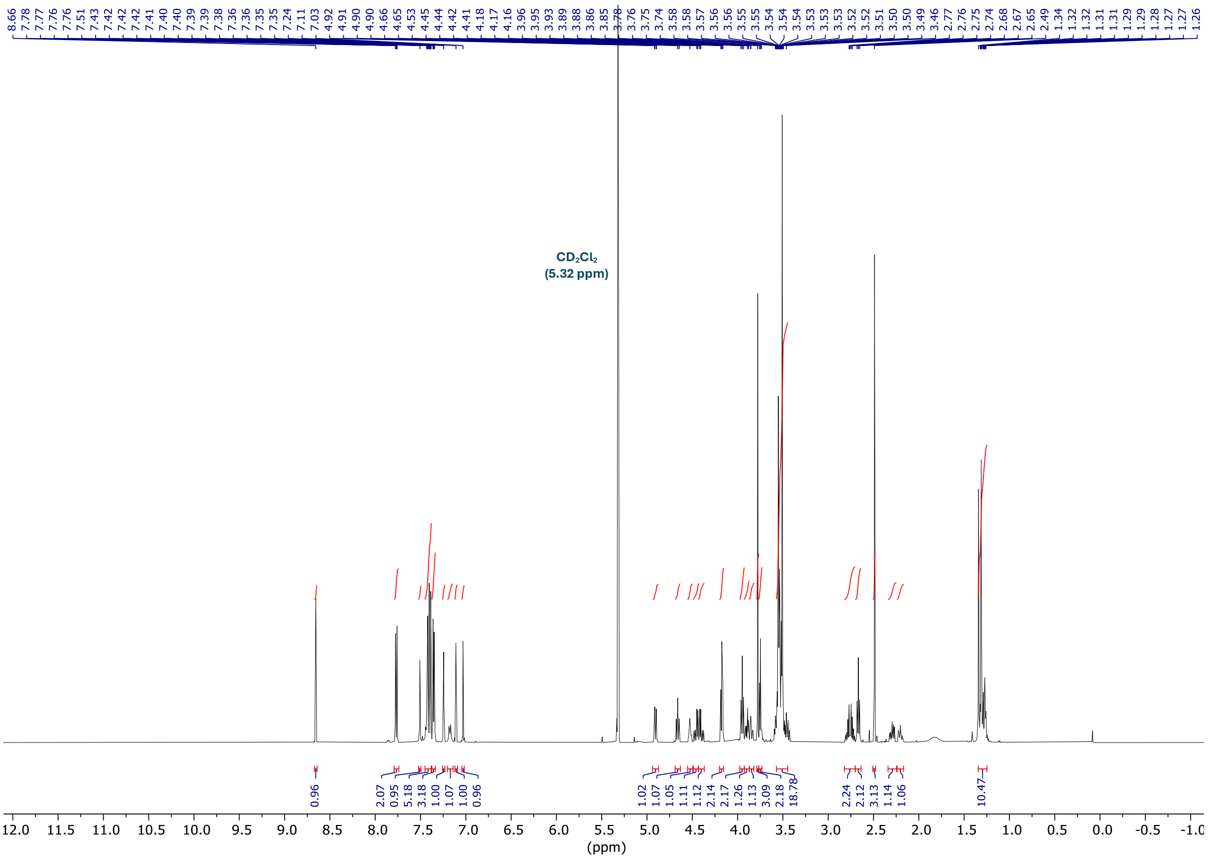

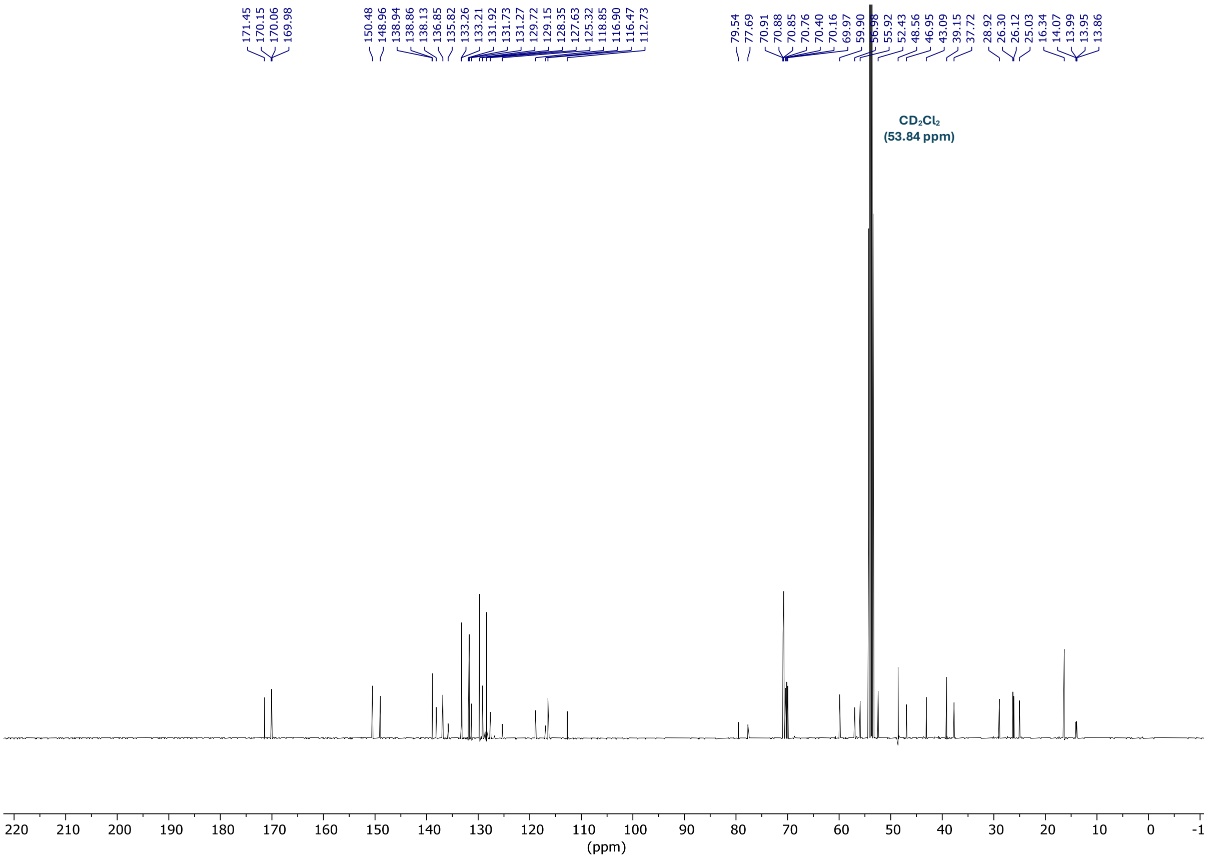

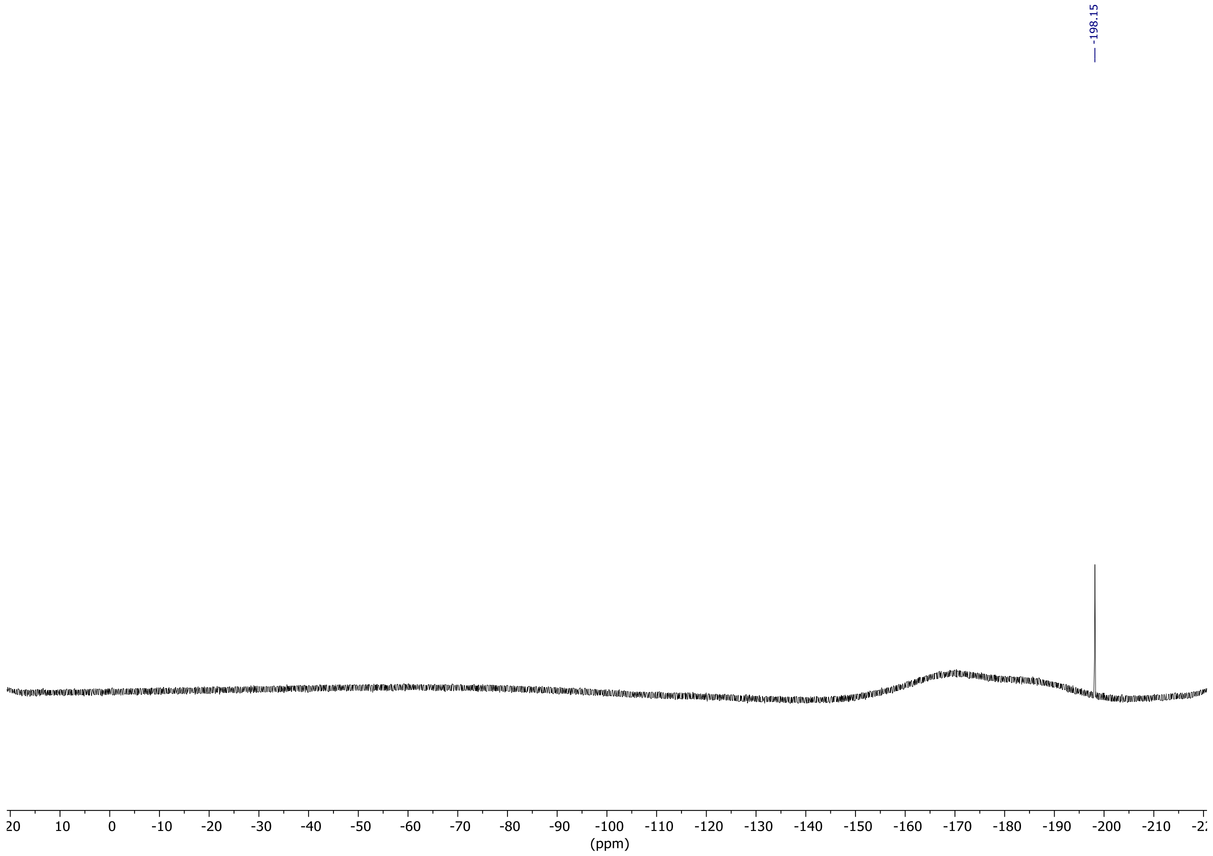
-198.15
20
10
0
-10
-20
-30
-40
-50
-60
-70
-80
-90
-100
-110
-120
-130
-140
-150
-160
-170
-180
-190
-200
-210
(ppm)

Compound 25. *^{1}H NMR (500 MHz, CD_2Cl_2), ^{13}C NMR (126 MHz, CD_2Cl_2) and ^{19}F NMR (377 MHz, CD_2Cl_2)*

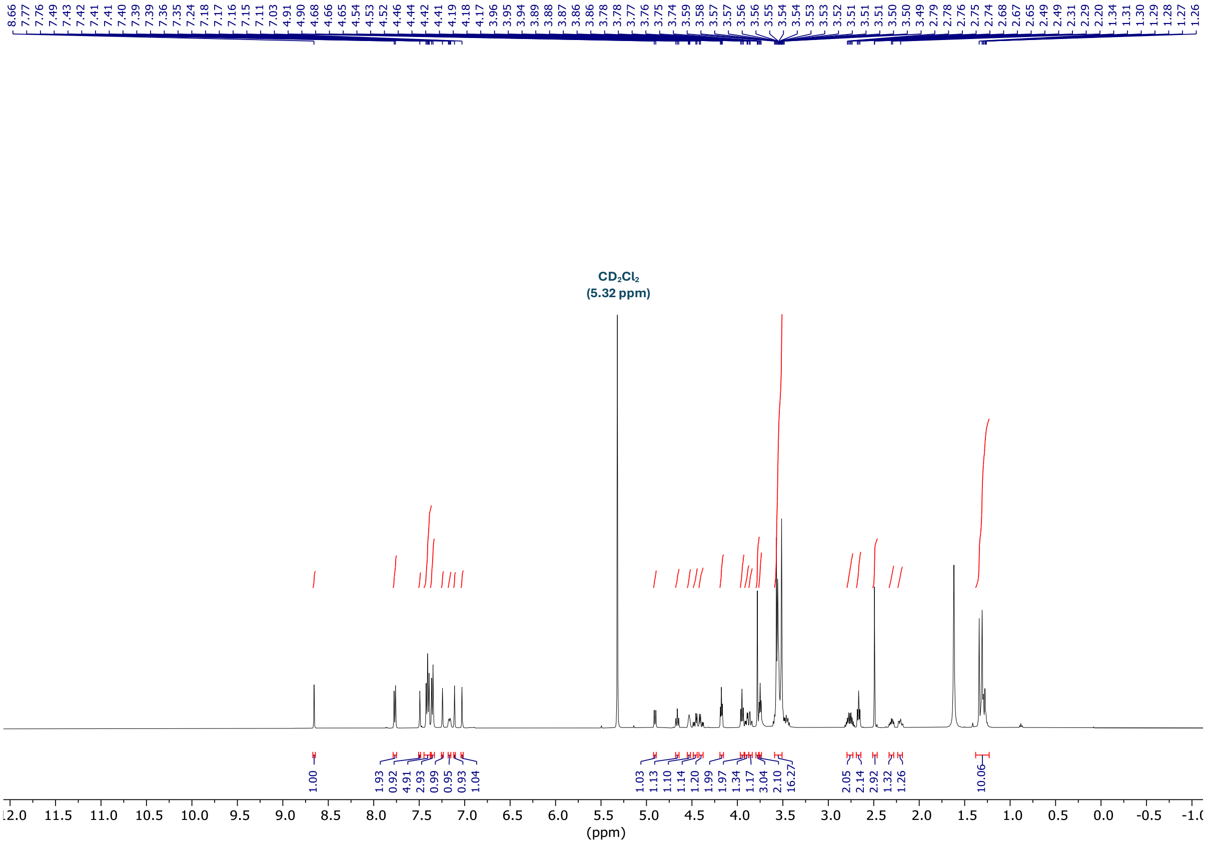

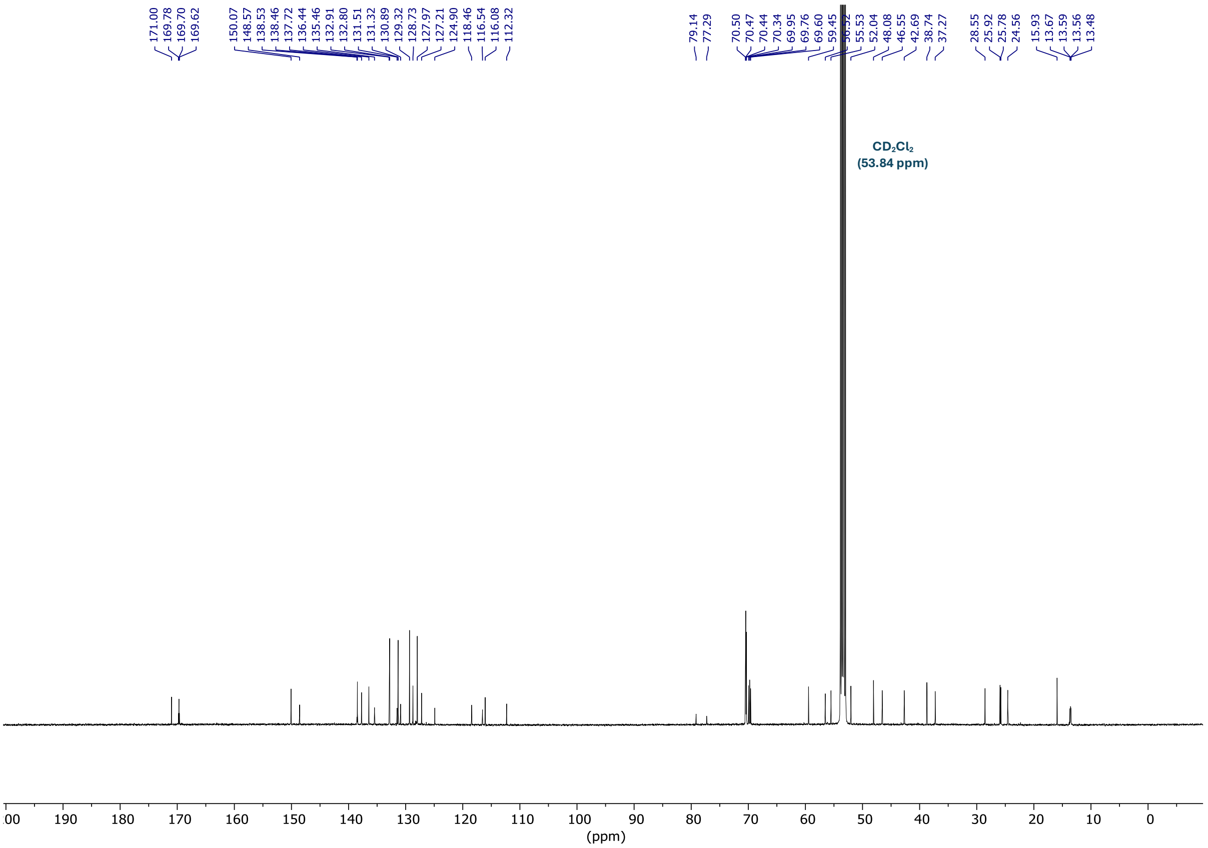

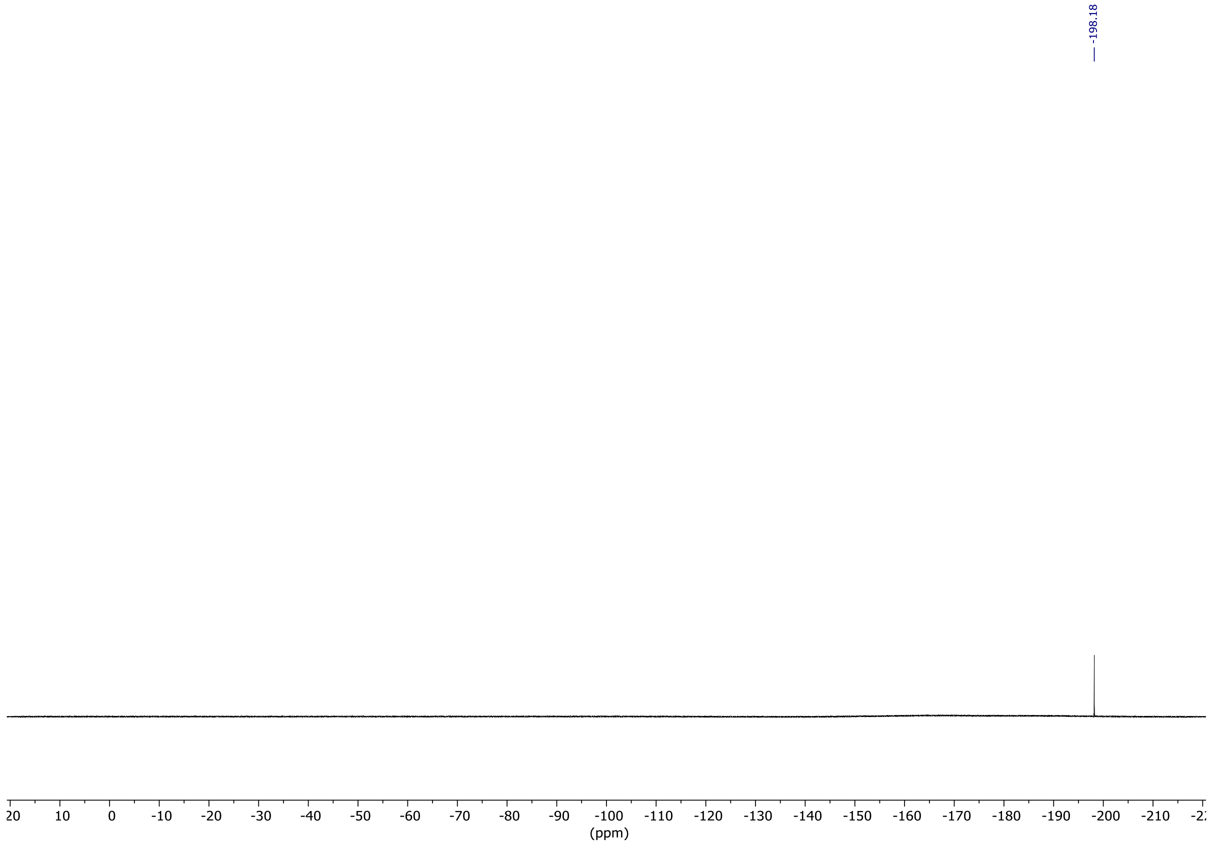

-198.18
(ppm)

Compound 26. *1H NMR (500 MHz, CD2Cl2) and 13C NMR (126 MHz, CD2Cl2).*

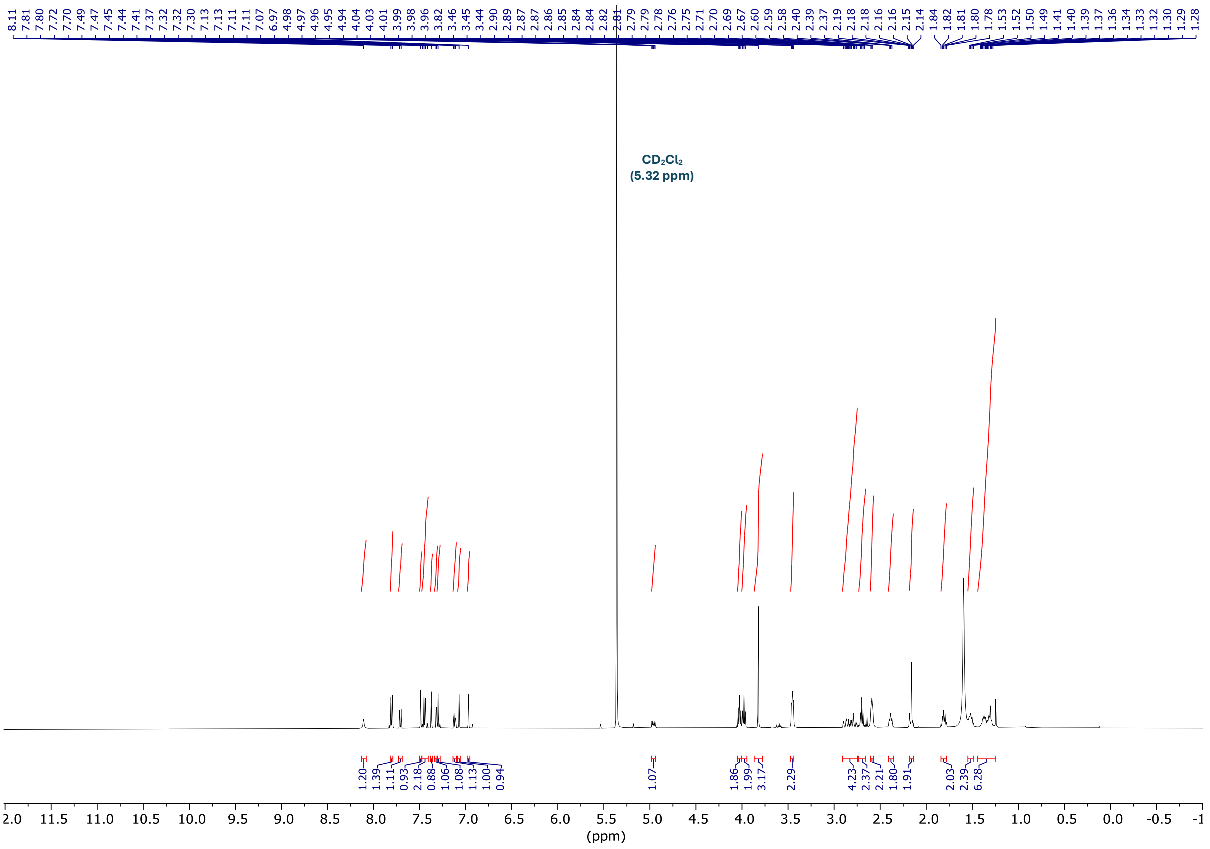

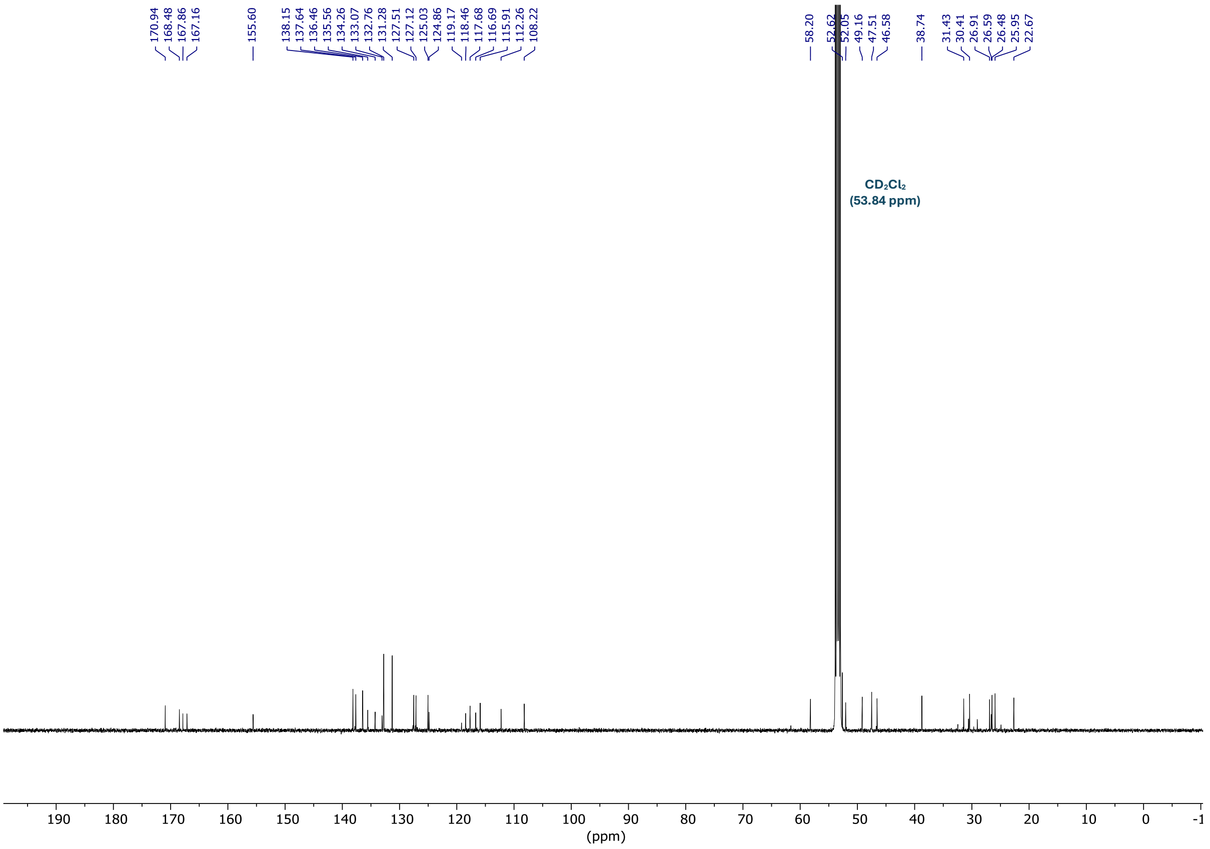

Compound 27. *¹H NMR (500 MHz, CD_2Cl_2) and ¹³C NMR (126 MHz, CD_2Cl_2).*

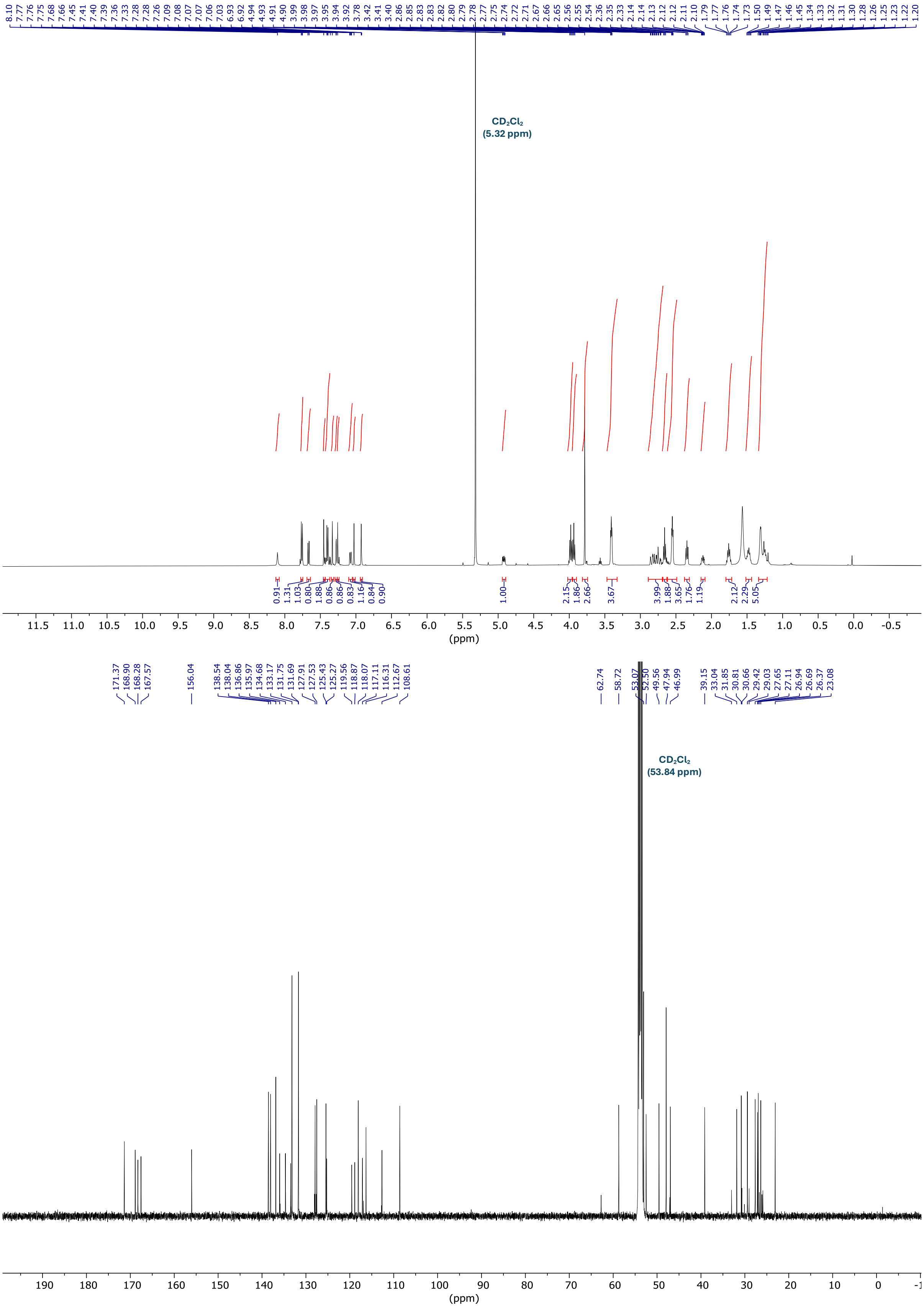

Compound 28. *¹H NMR (500 MHz, CD_2Cl_2) and ¹³C NMR (126 MHz, CD_2Cl_2).*

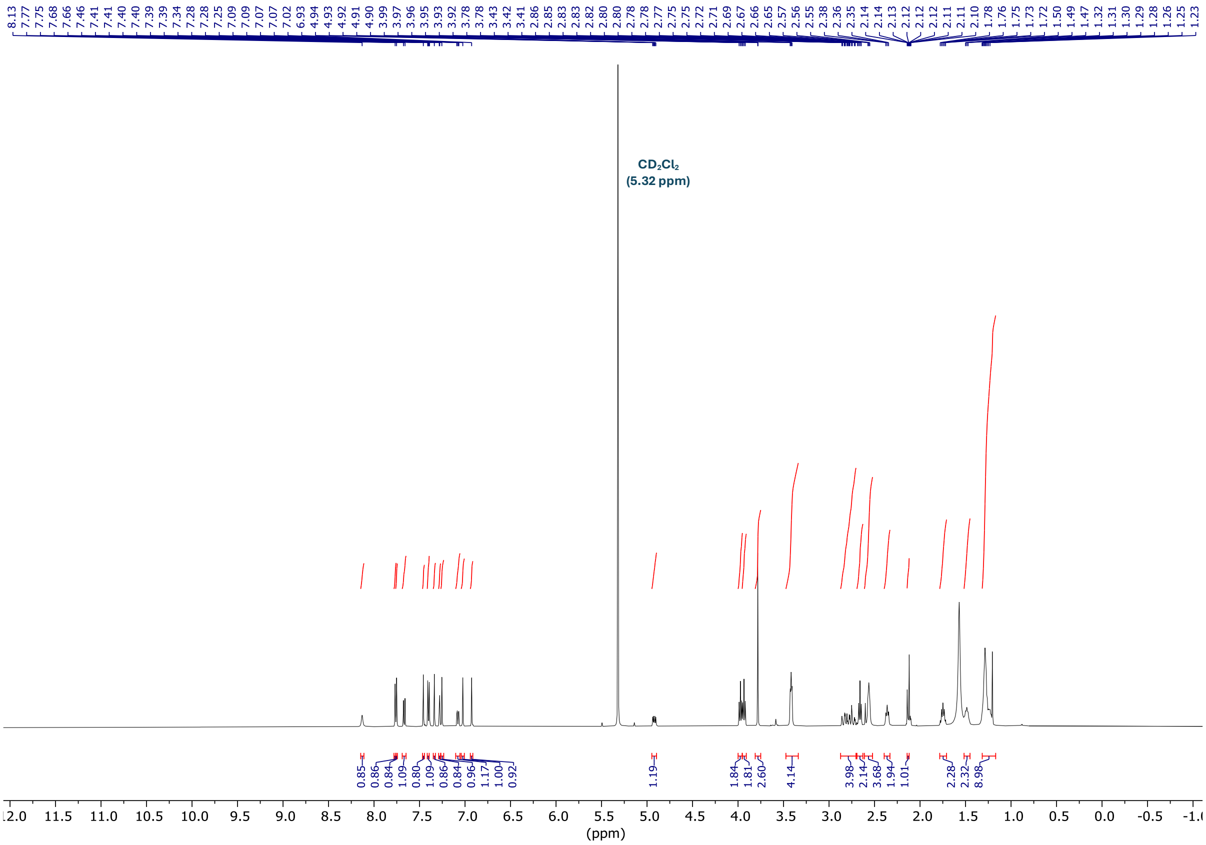

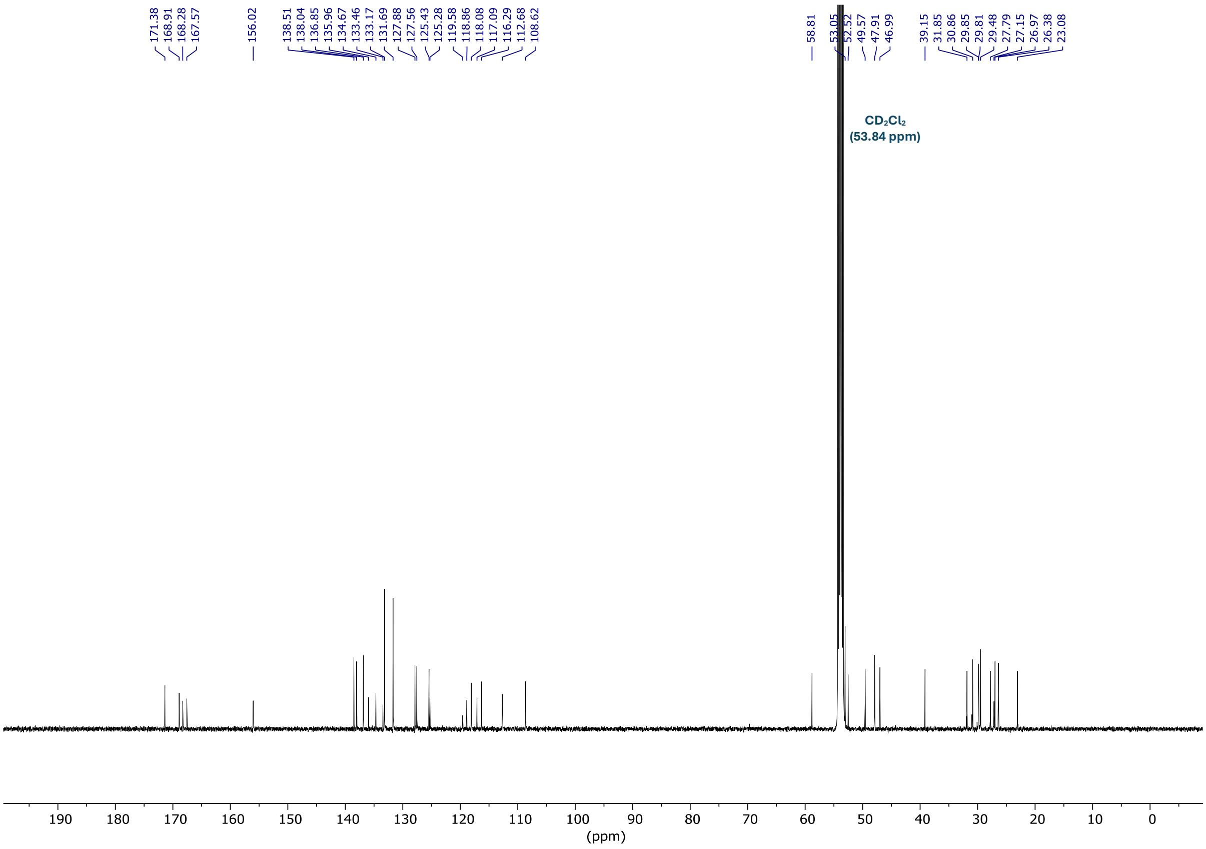

Compound 29. *^{1}H NMR (500 MHz, CD_2Cl_2) and ^{13}C NMR (126 MHz, CD_2Cl_2).*

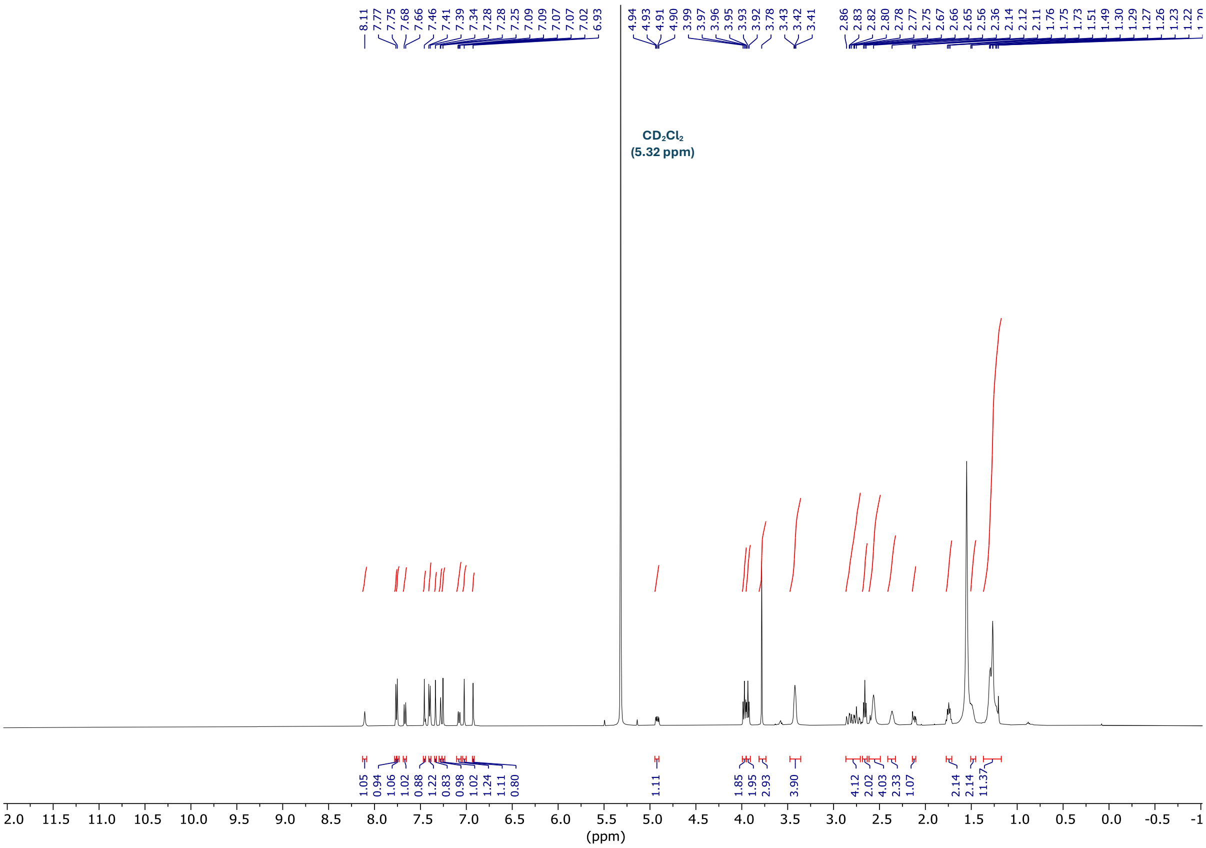

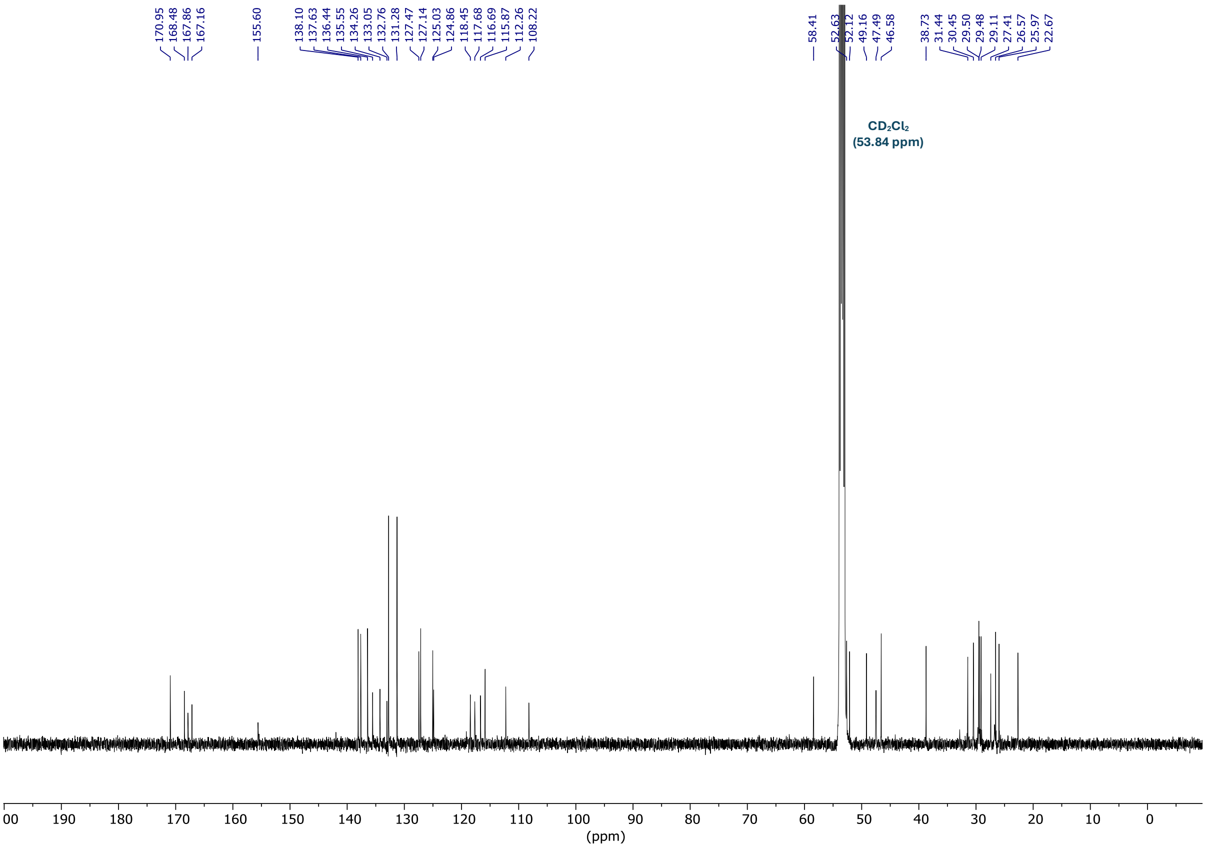

Compound 30. *^{1}H NMR (500 MHz, CD_2Cl_2) and ^{13}C NMR (126 MHz, CD_2Cl_2)*

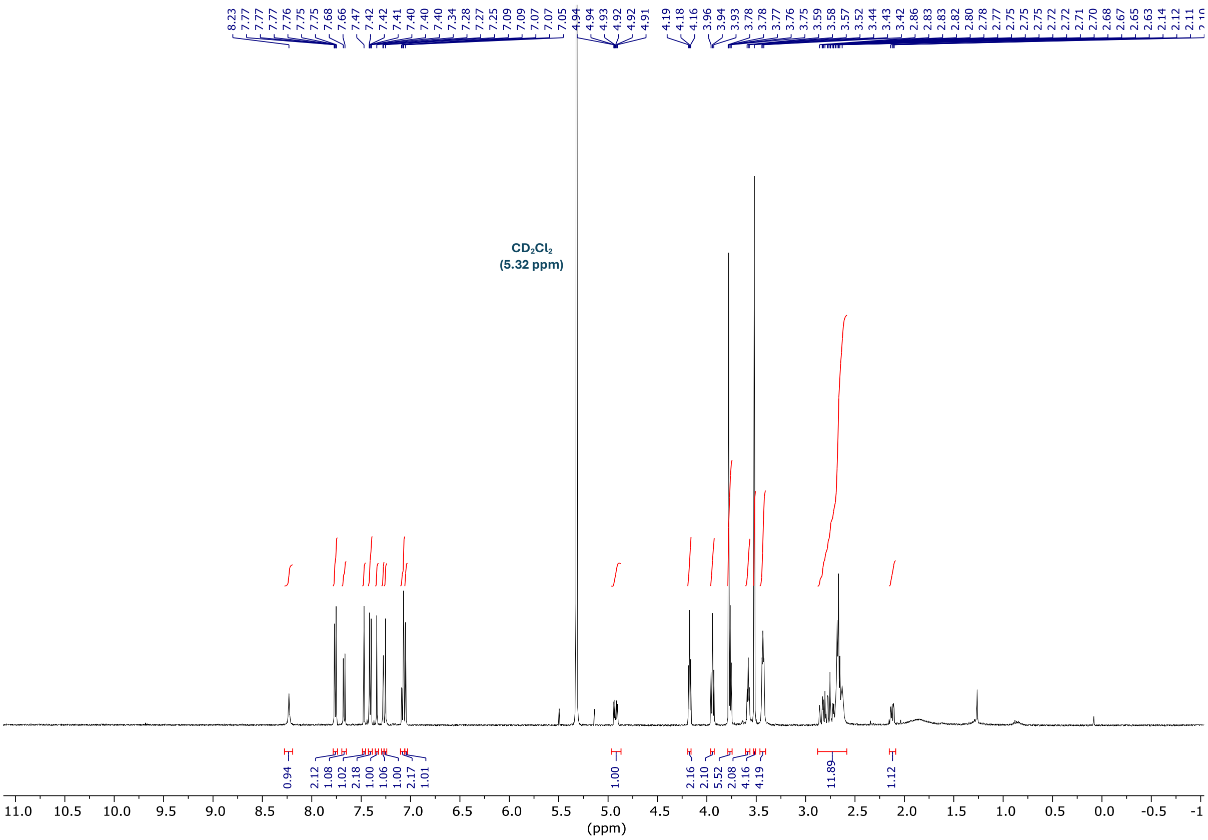

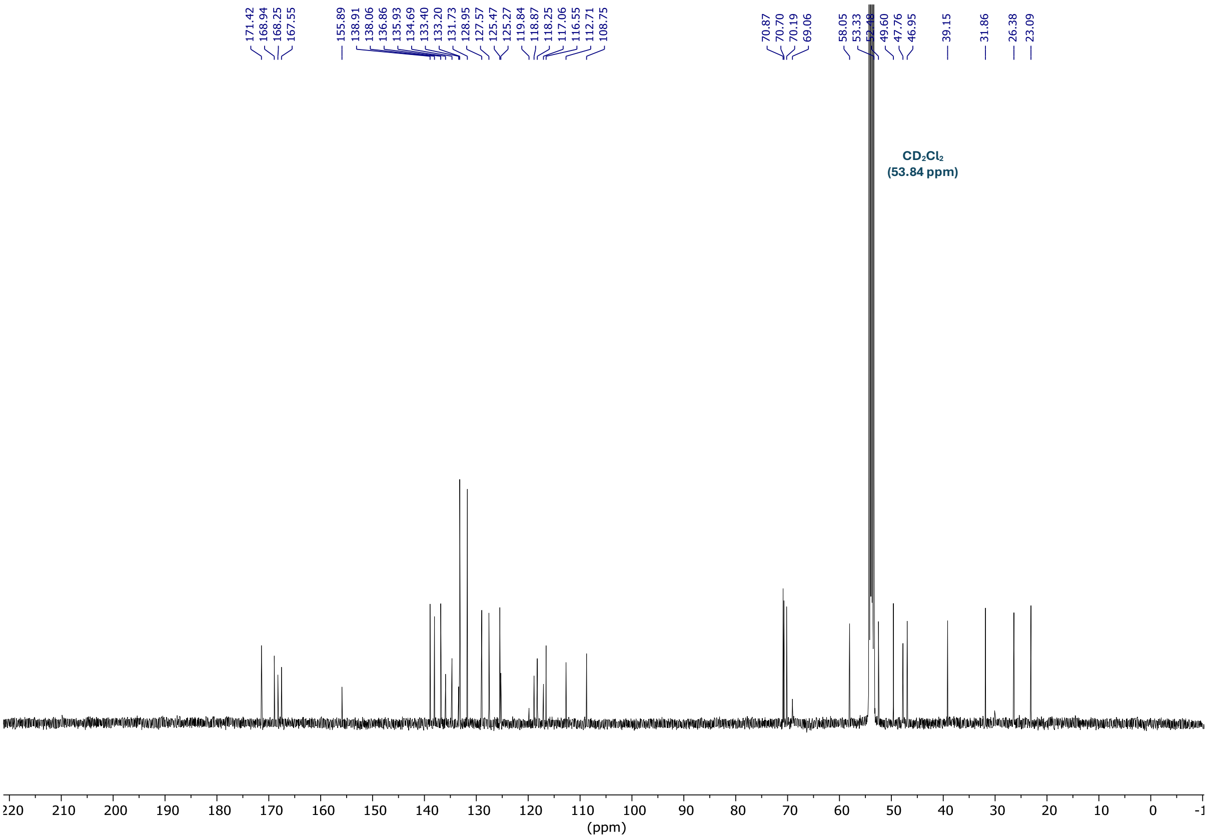

Compound 31. *^{1}H NMR (500 MHz, CD_2Cl_2) and ^{13}C NMR (126 MHz, CD_2Cl_2)*

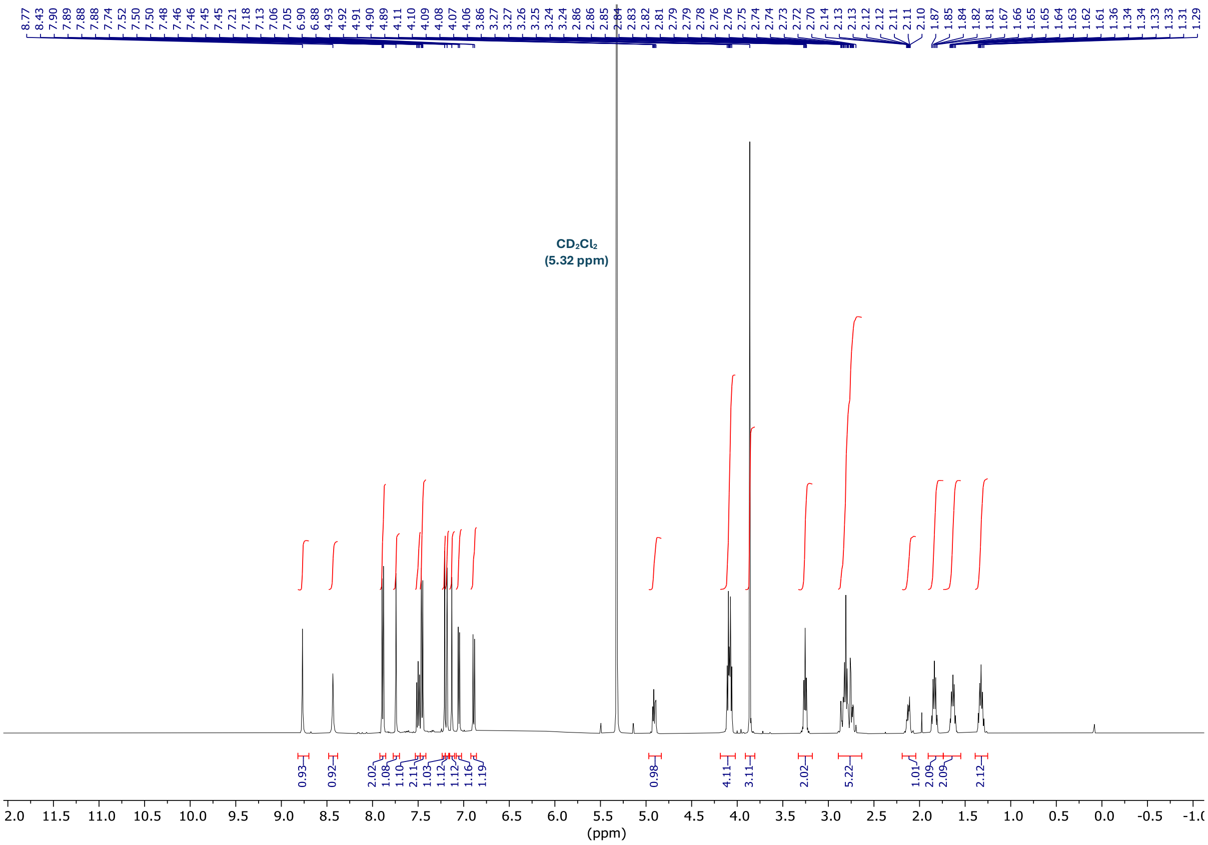

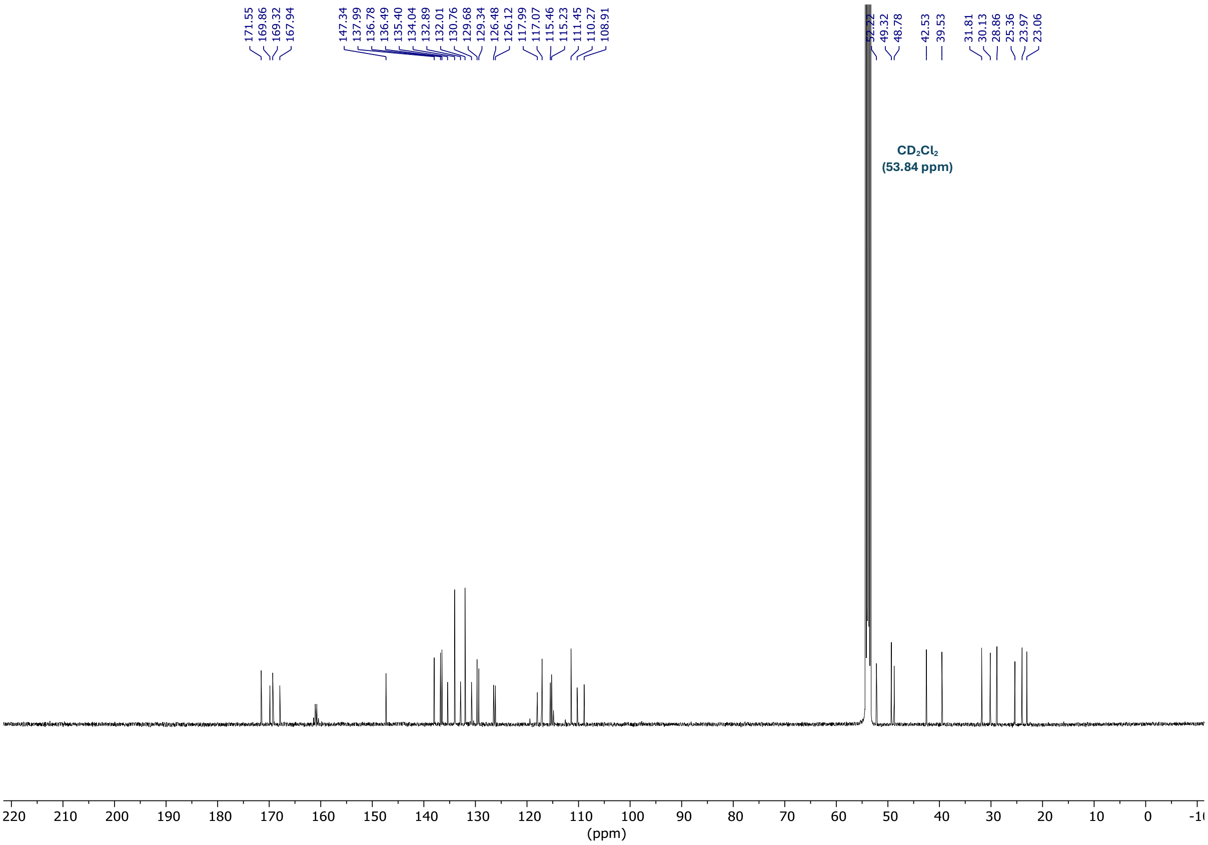

Compound 32. *^{1}H NMR (500 MHz, CD_2Cl_2) and ^{13}C NMR (126 MHz, CD_2Cl_2)*

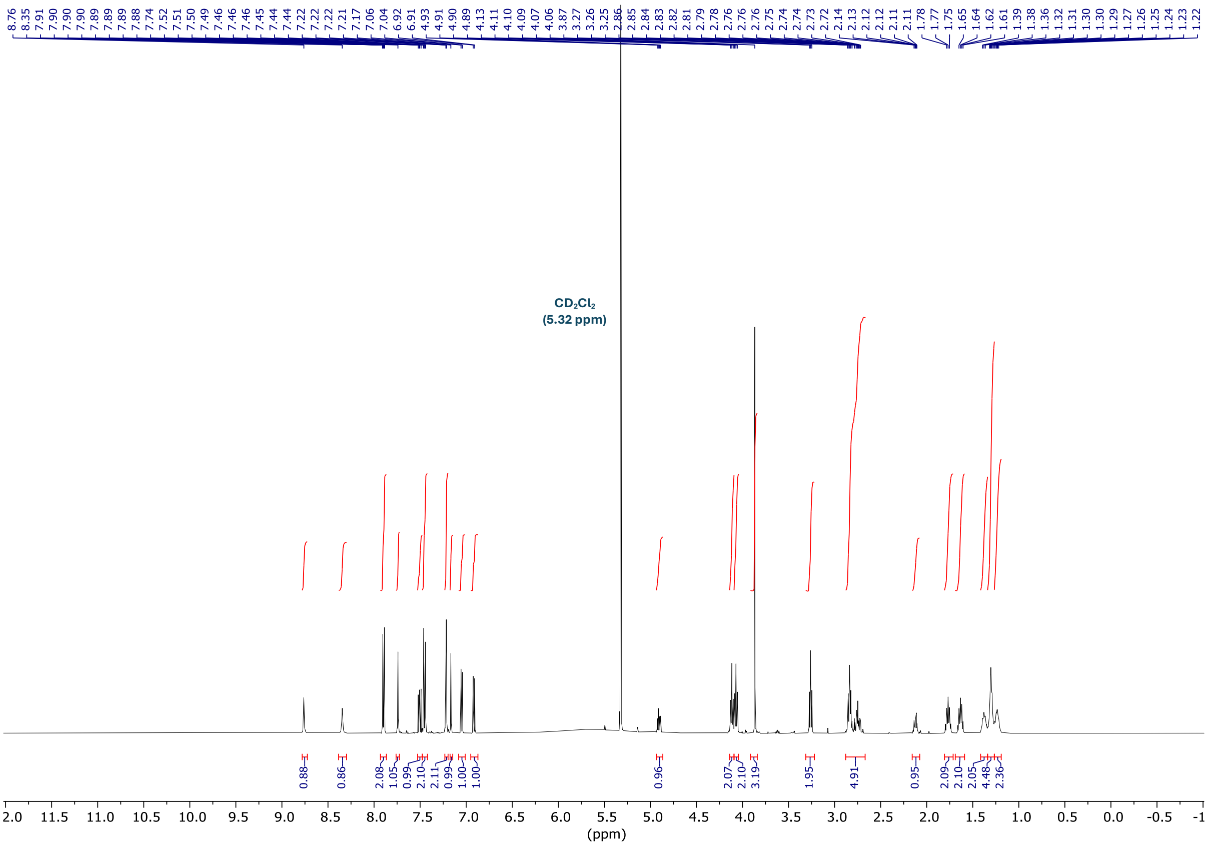

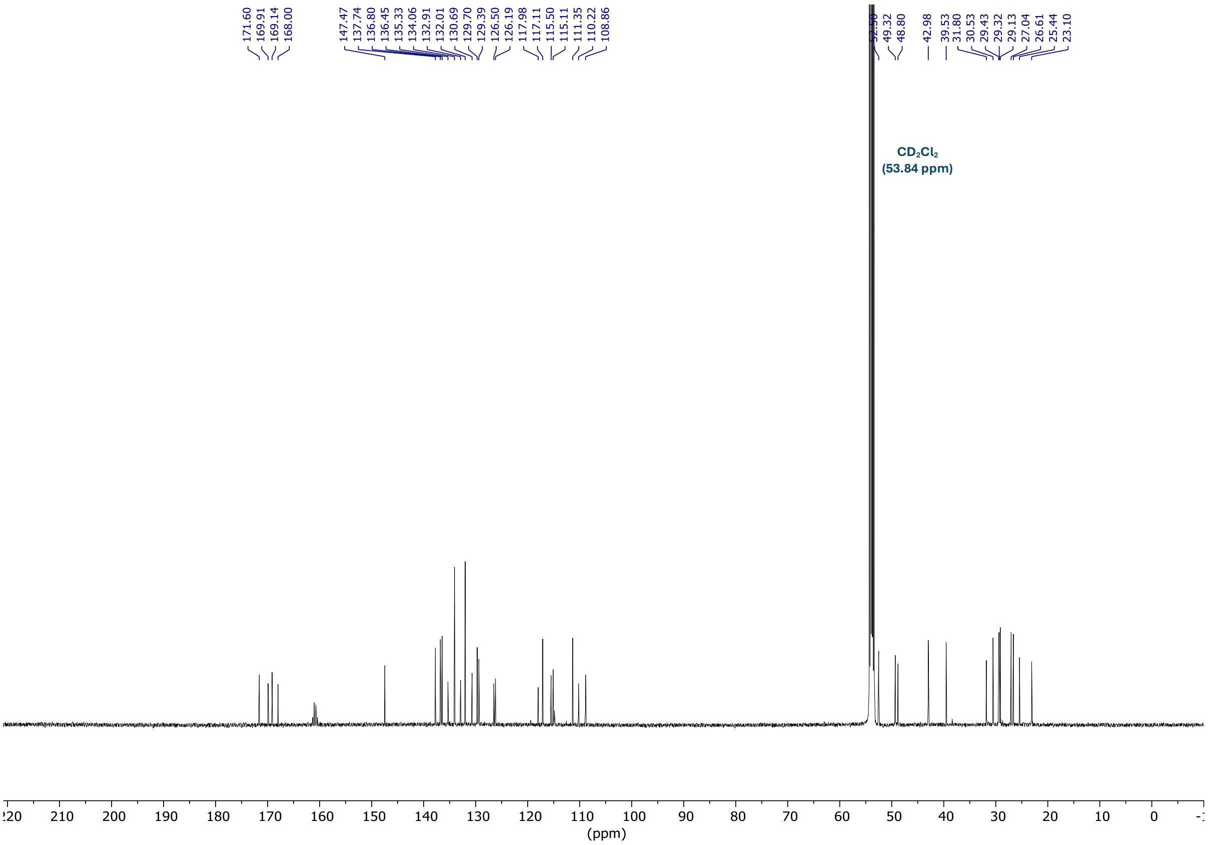

Compound 33. *^{1}H NMR (500 MHz, CD_2Cl_2) and ^{13}C NMR (126 MHz, CD_2Cl_2)*

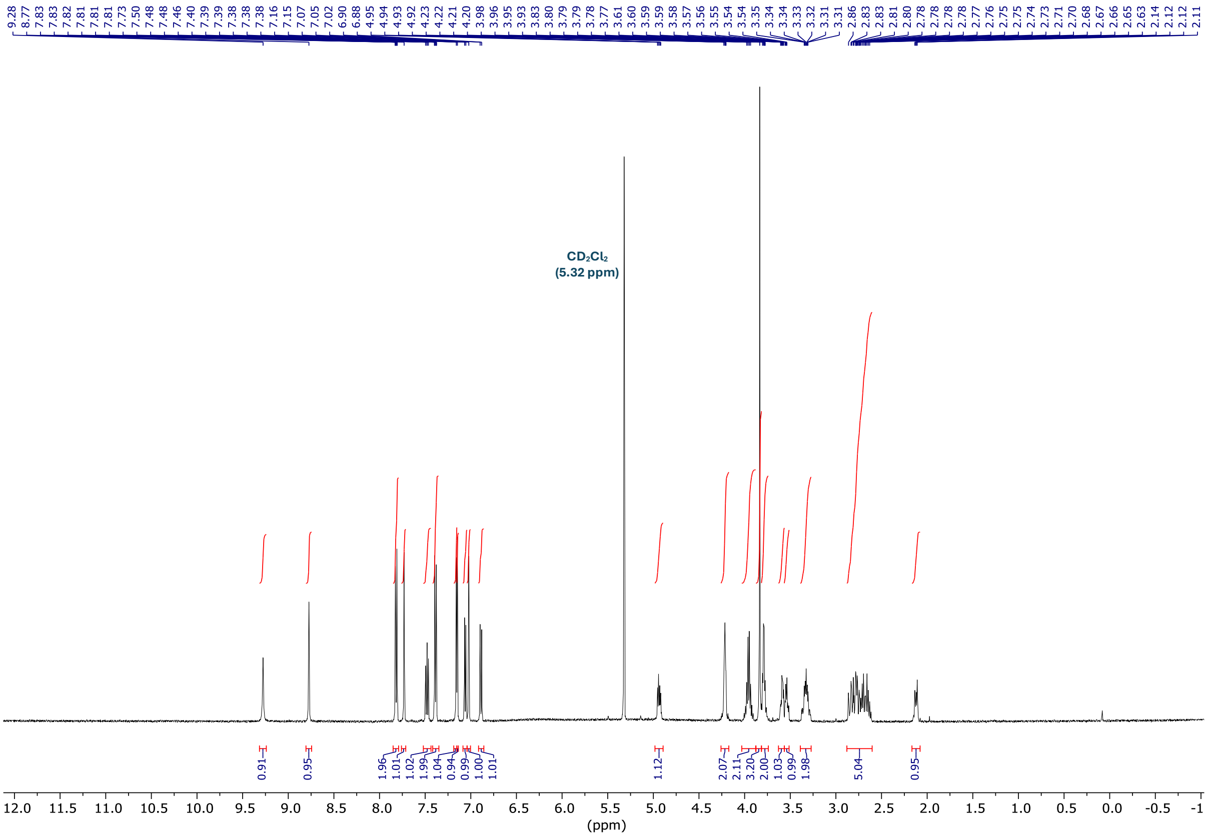

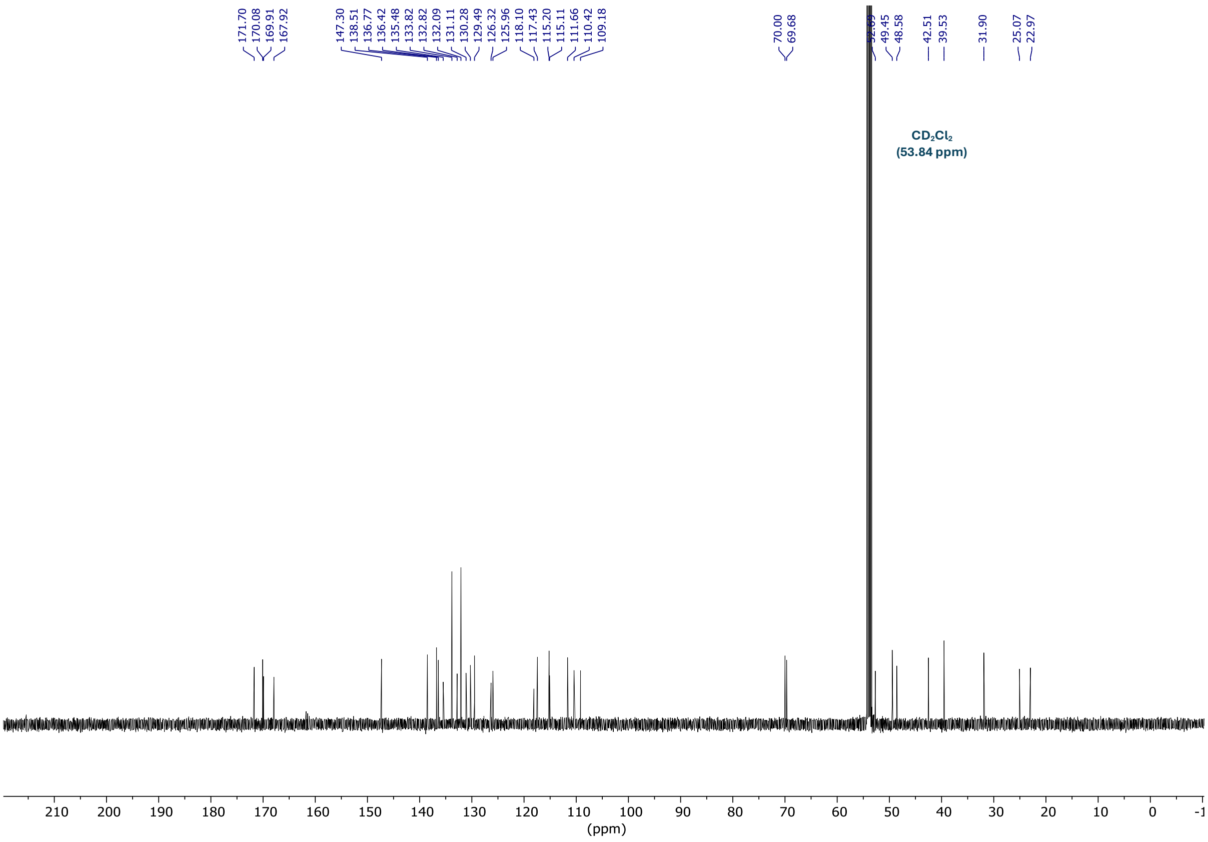

Compound 34. *^{1}H NMR (500 MHz, CD_2Cl_2) and ^{13}C NMR (126 MHz, CD_2Cl_2)*

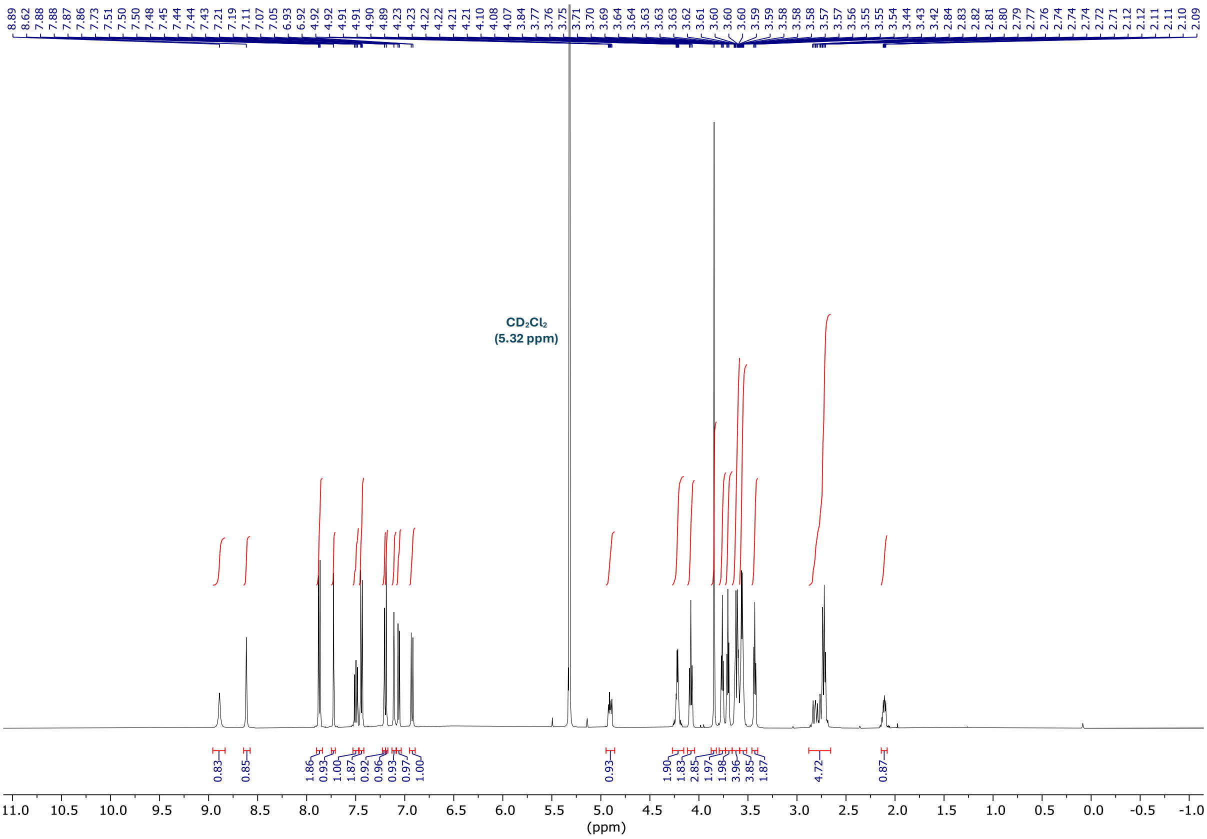

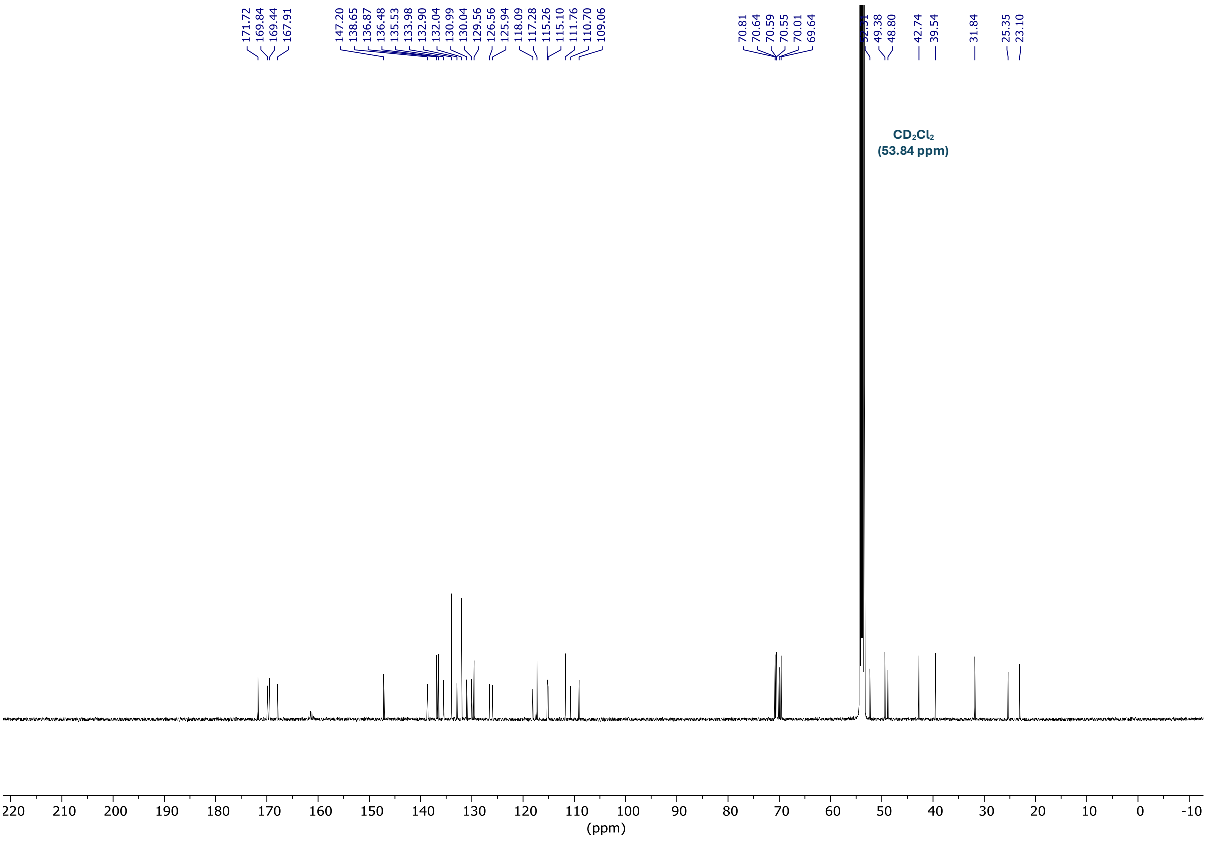

Compound 35. *^1H NMR (400 MHz, CD_2Cl_2) and ^{13}C NMR (126 MHz, CD_2Cl_2)*

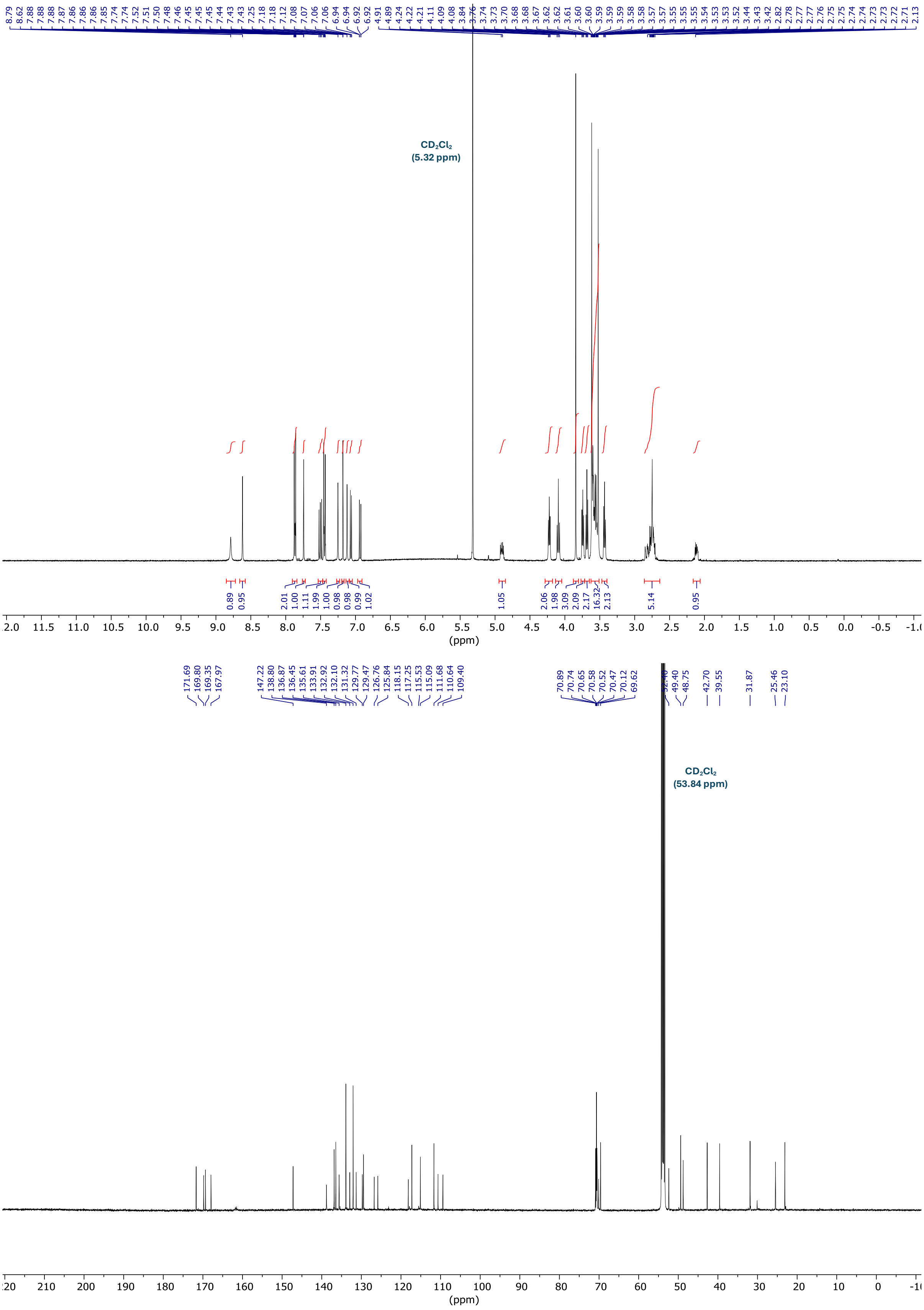

Compound 36. *^{1}H NMR (500 MHz, CD_2Cl_2) and ^{13}C NMR (126 MHz, CD_2Cl_2).*

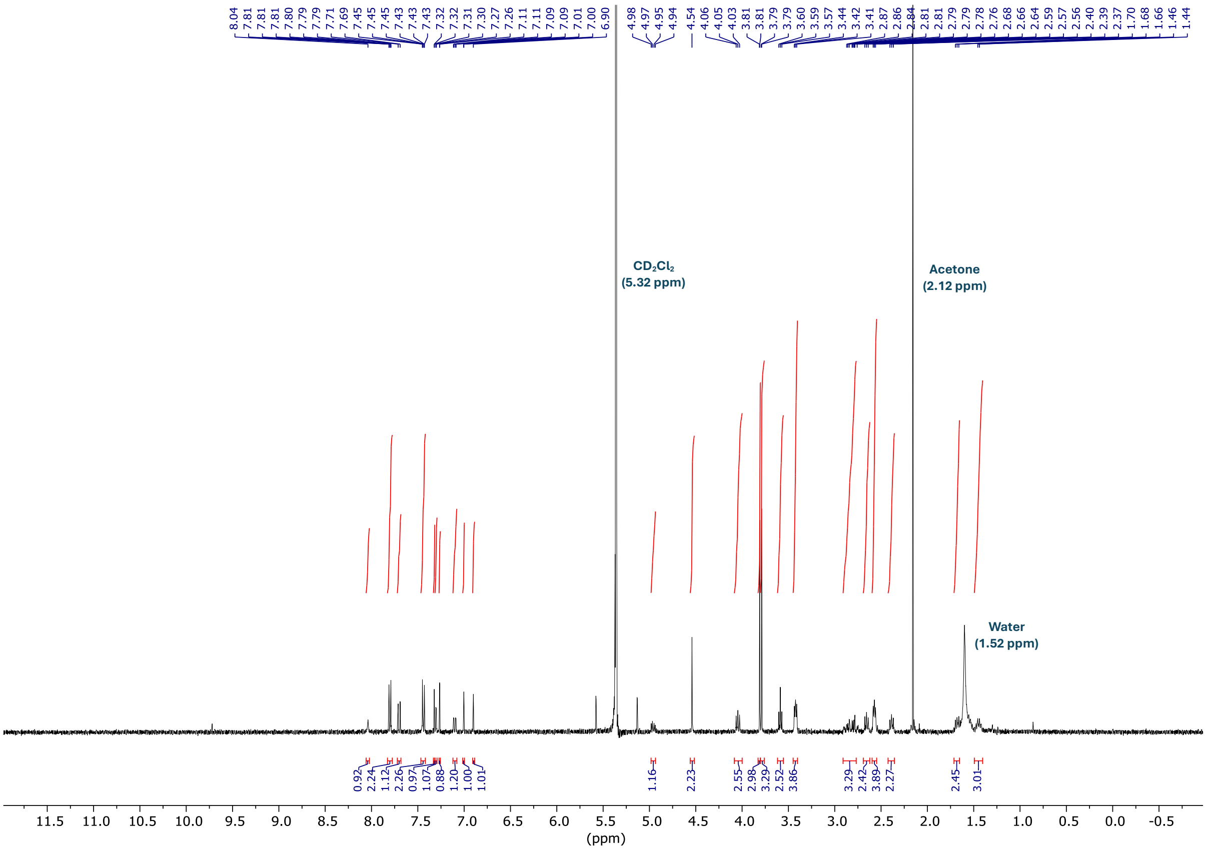

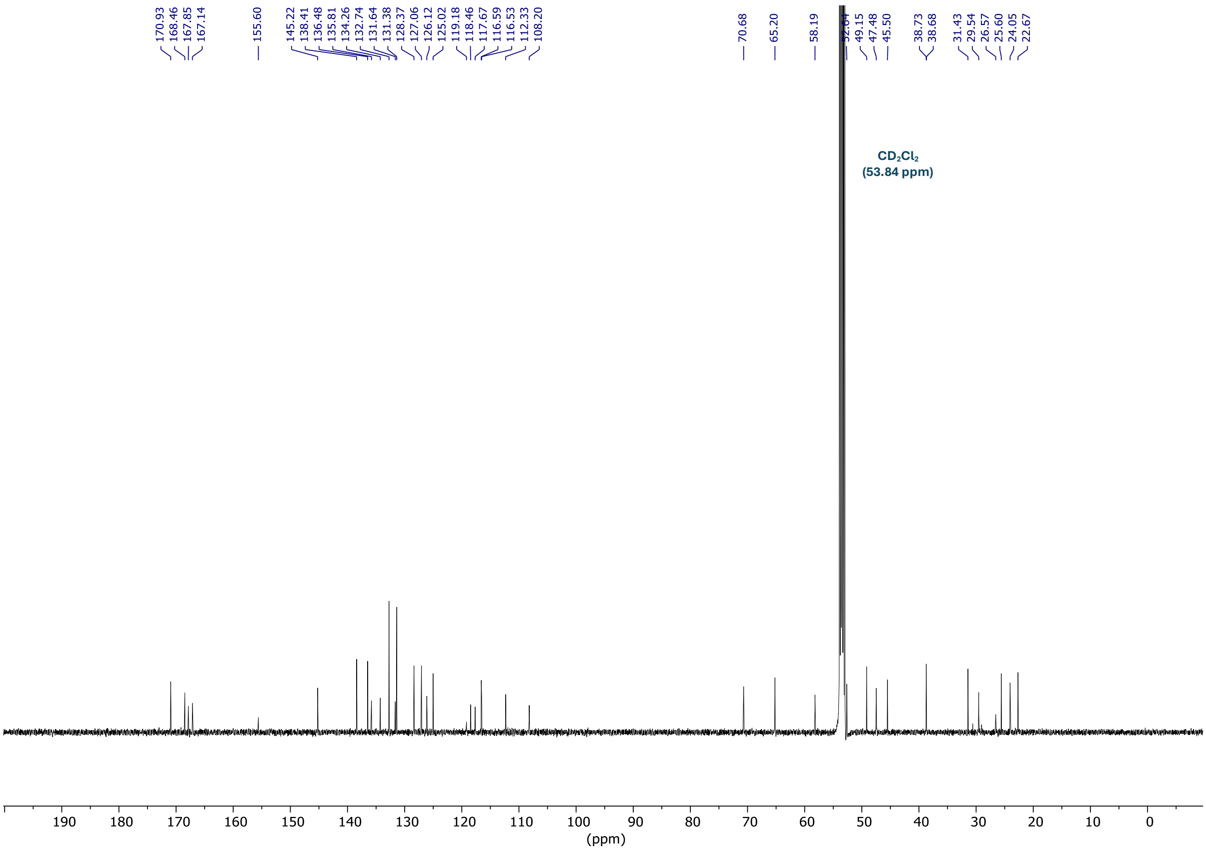

Compound 37. *^{1}H NMR (400 MHz, CD_2Cl_2) and ^{13}C NMR (126 MHz, CD_2Cl_2).*

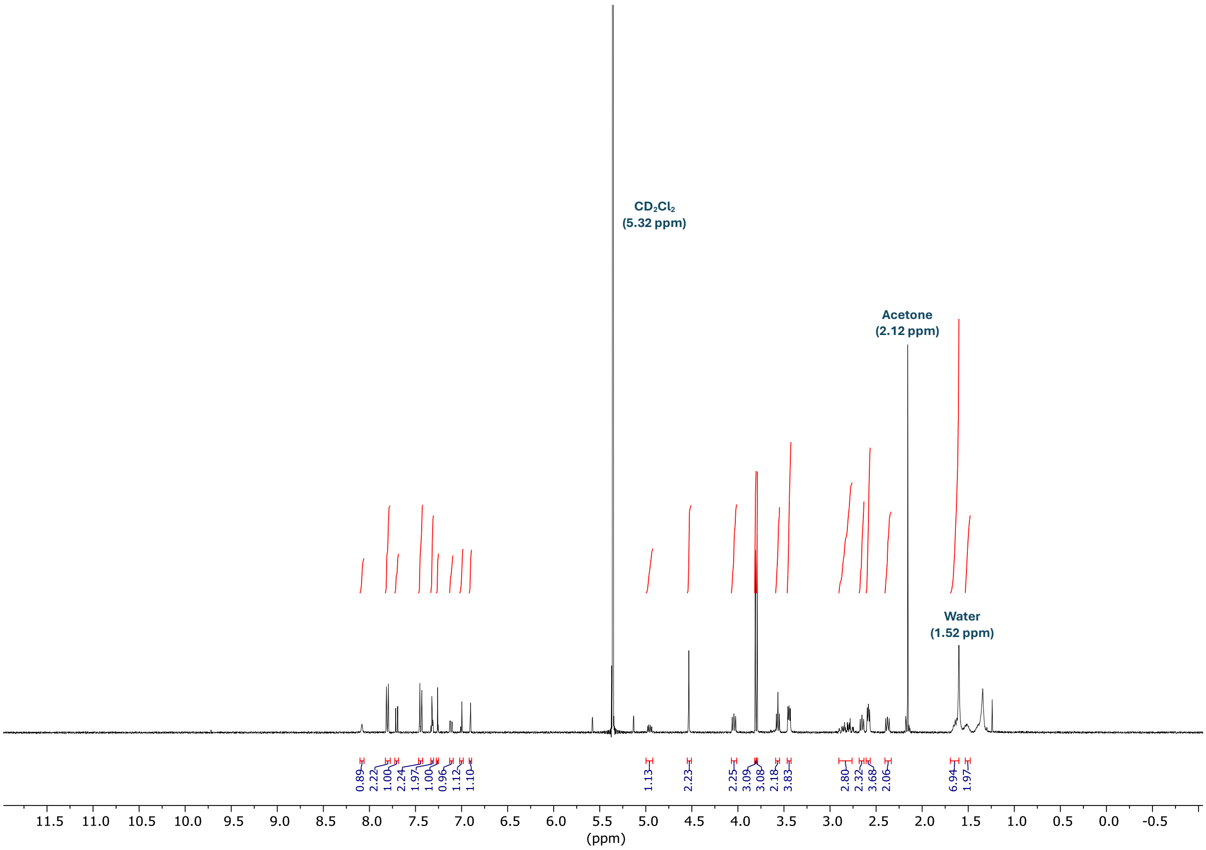

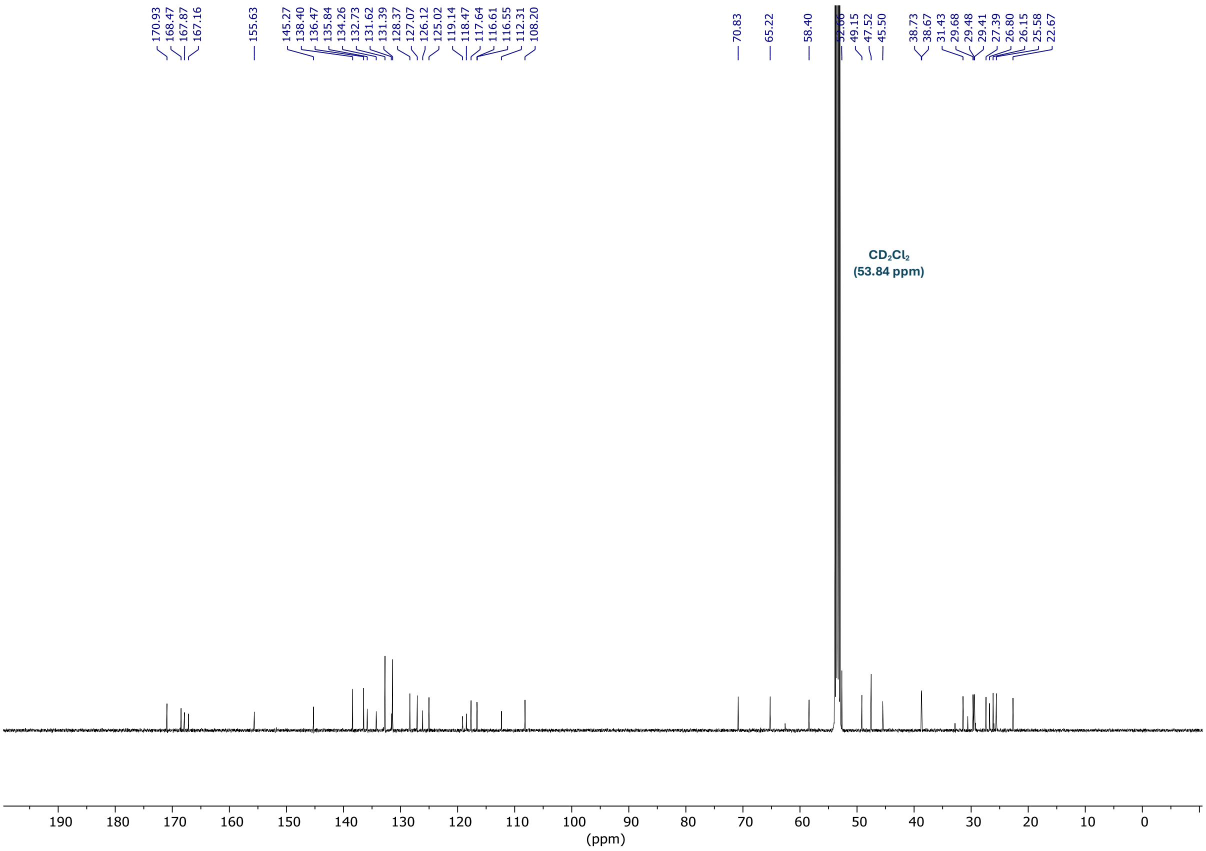

Compound 38. *^1H NMR (500 MHz, CD_2Cl_2) and ^{13}C NMR (126 MHz, CD_2Cl_2).*

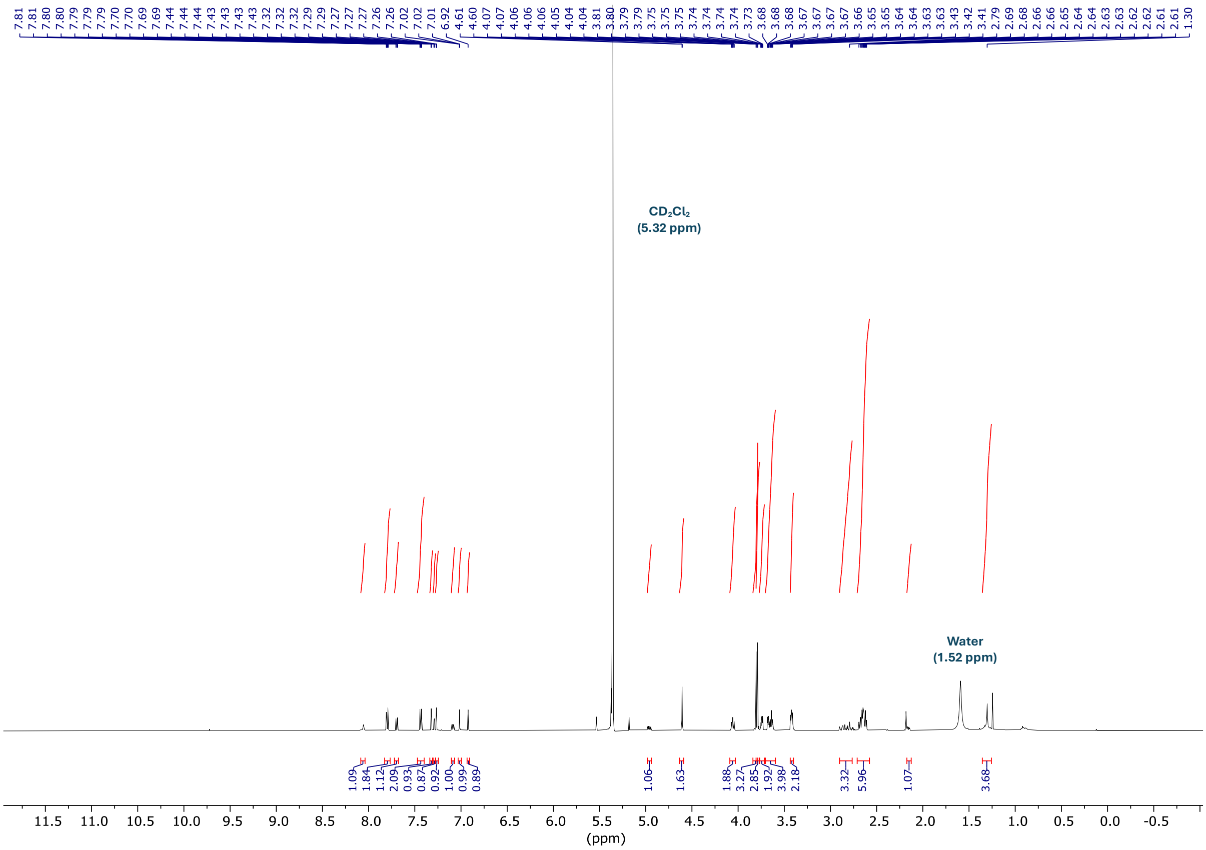

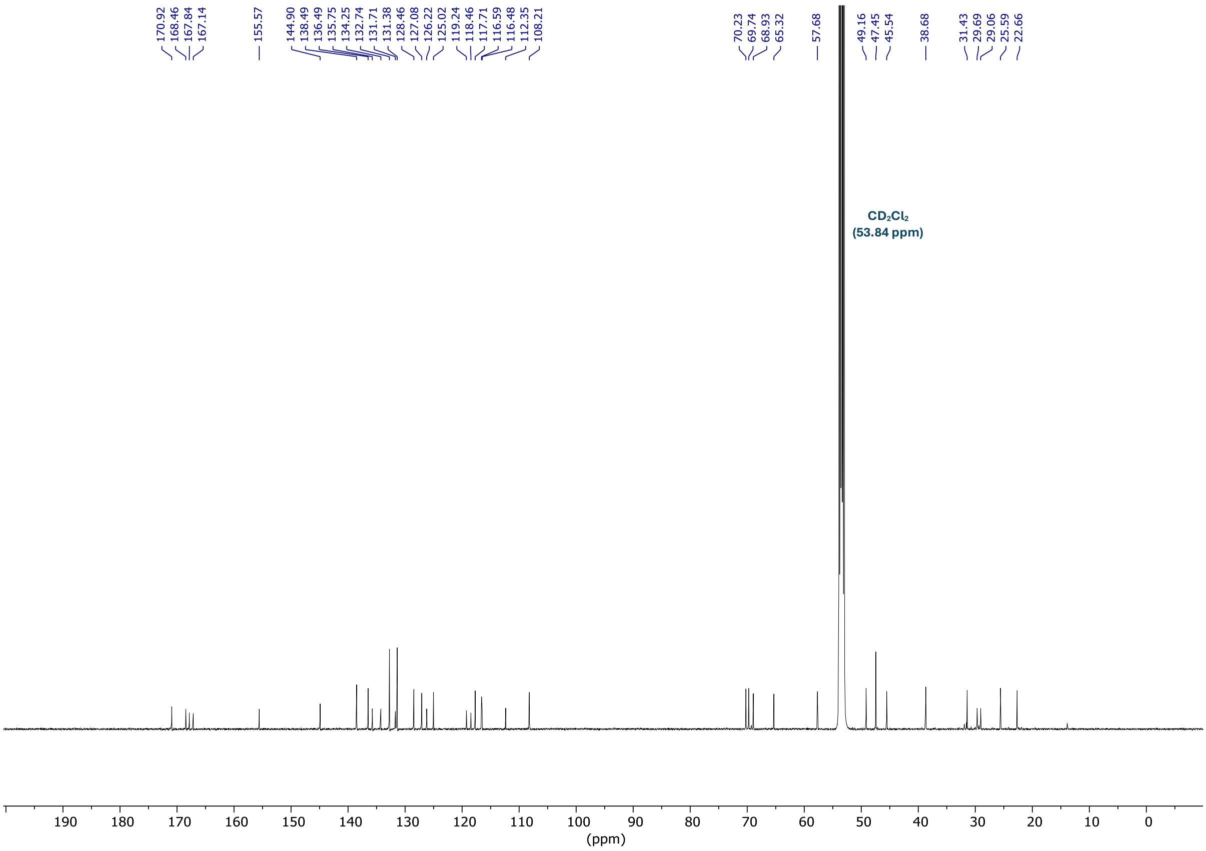

Compound 39. *^1H NMR (500 MHz, CD_2Cl_2), ^{13}C NMR (126 MHz, CD_2Cl_2) and ^{19}F NMR (377 MHz, CD_2Cl_2).*

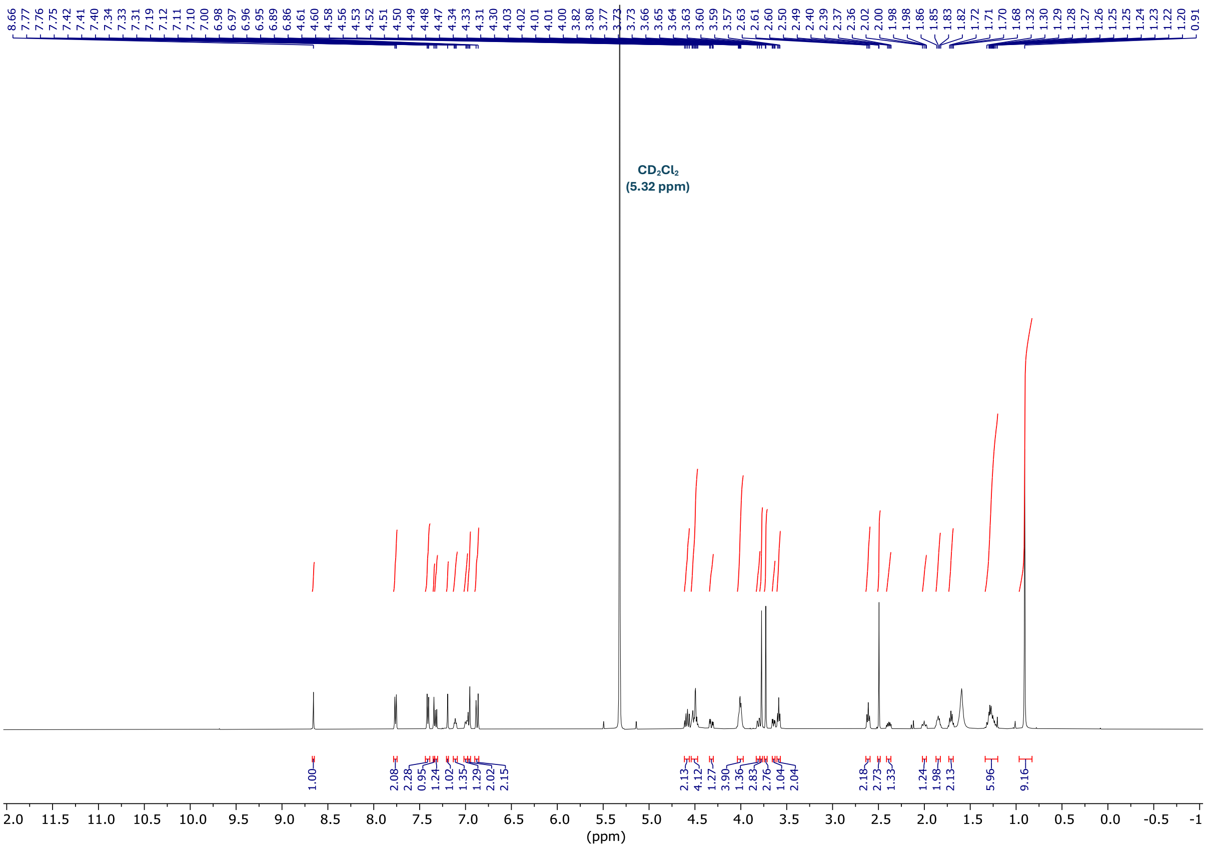

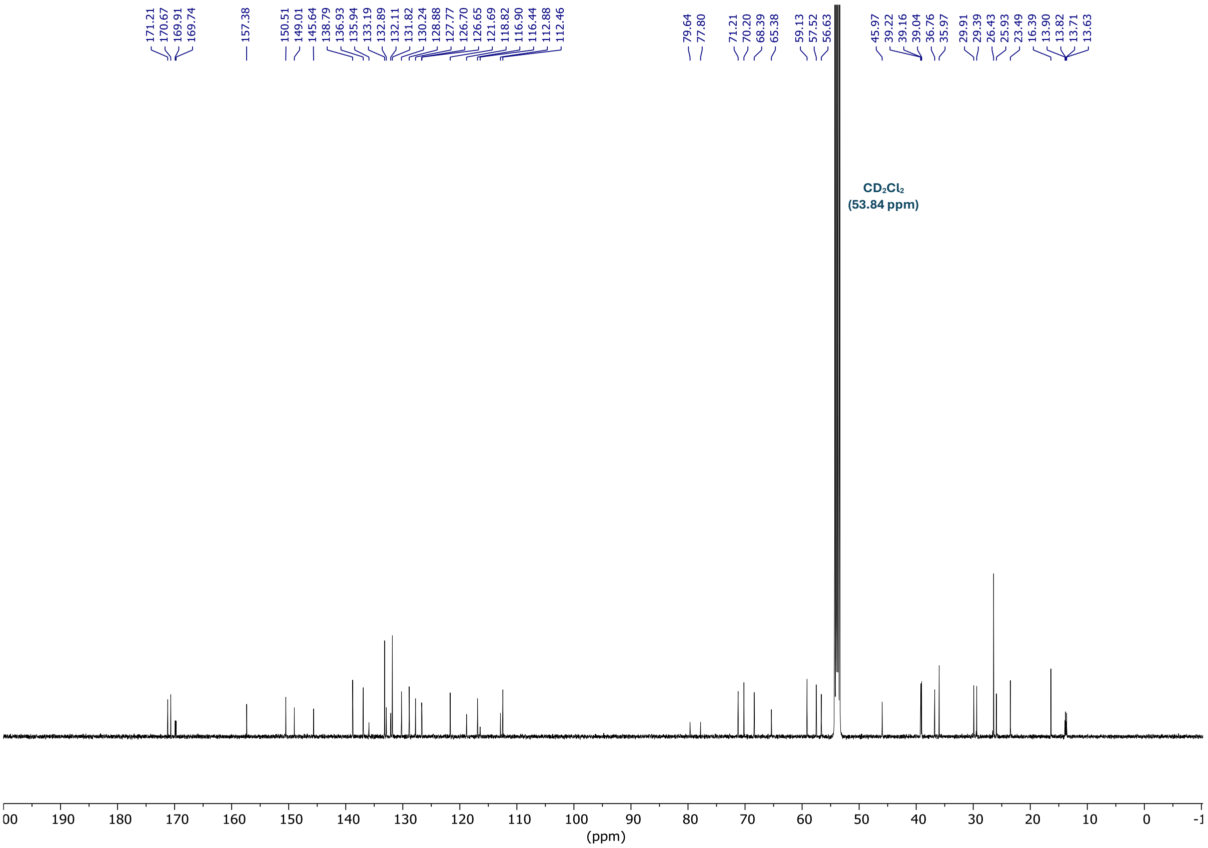

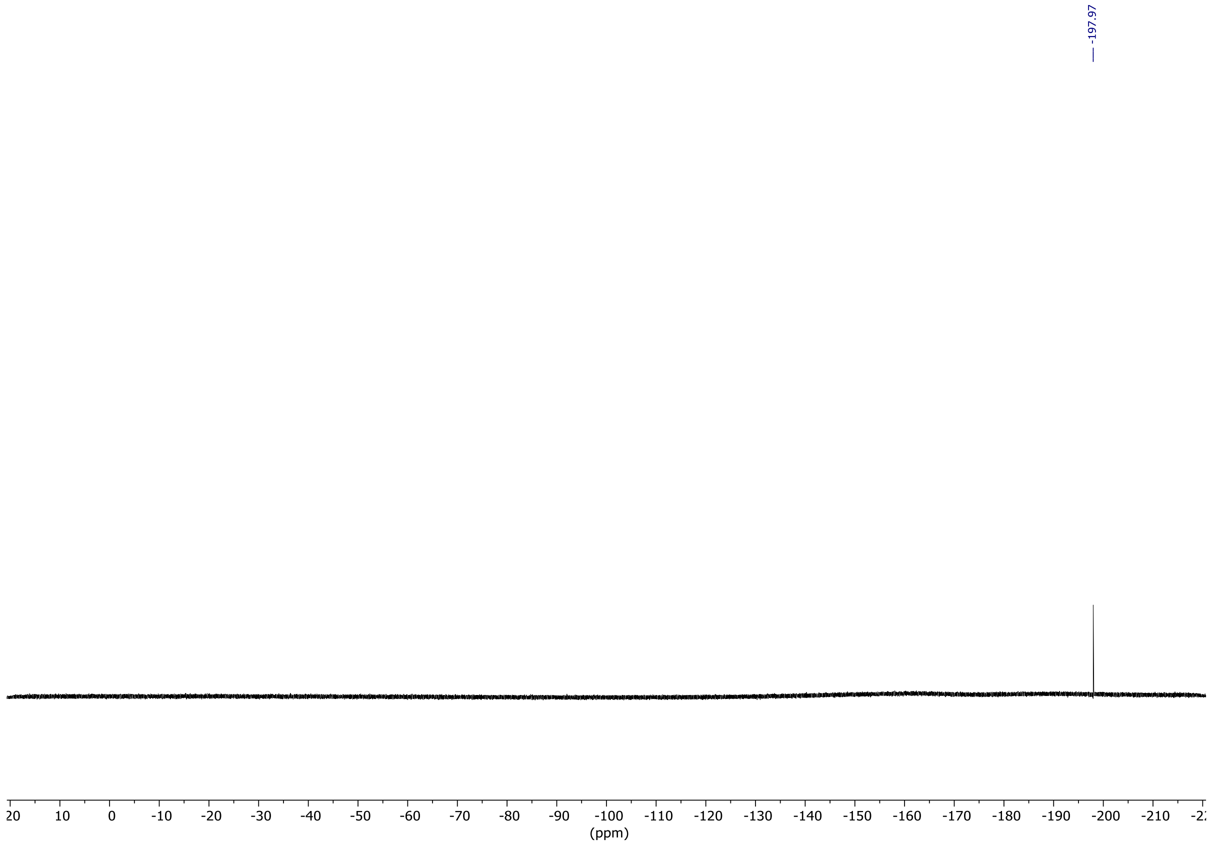

-197.97
20 10 0 -10 -20 -30 -40 -50 -60 -70 -80 -90 -100 -110 -120 -130 -140 -150 -160 -170 -180 -190 -200 -210
(ppm)

Compound 40. *^{1}H NMR (500 MHz, CD_2Cl_2), ^{13}C NMR (126 MHz, CD_2Cl_2) and ^{19}F NMR (471 MHz, CD_2Cl_2).*

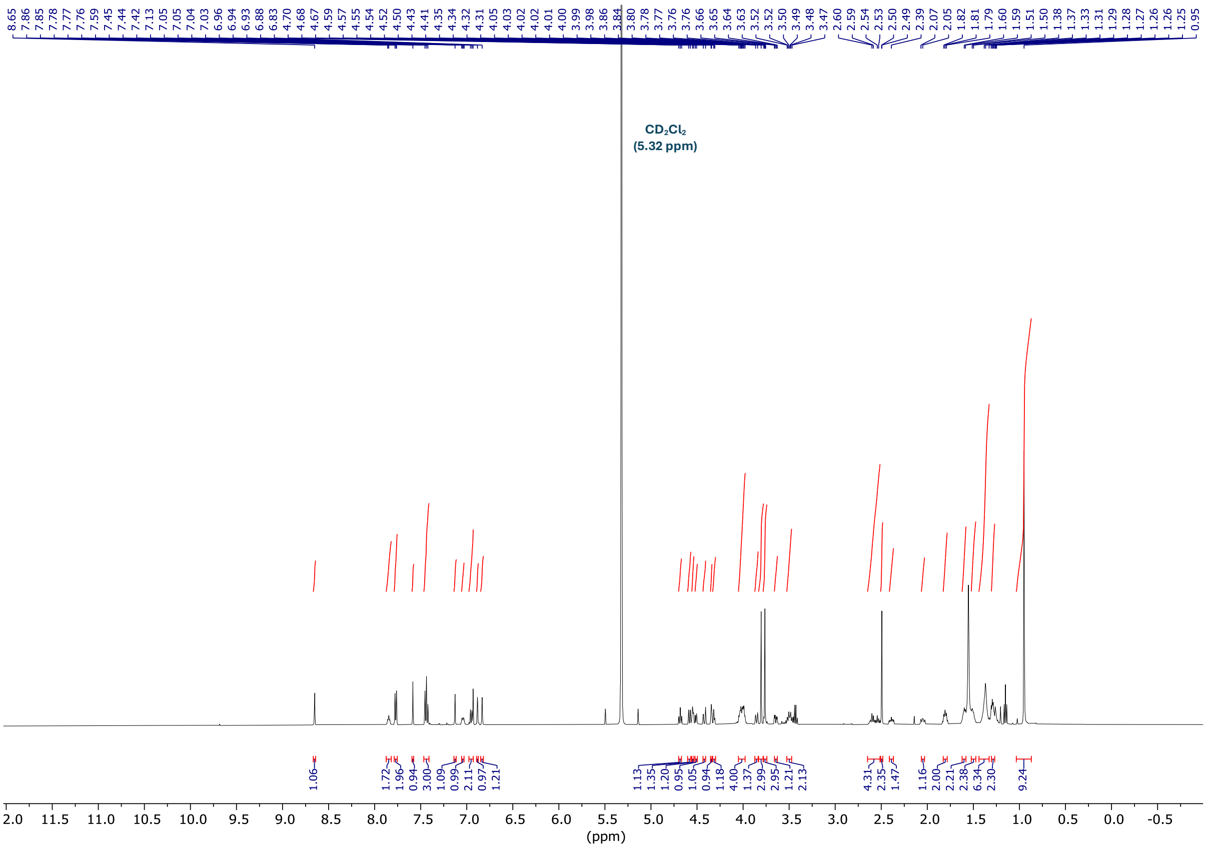

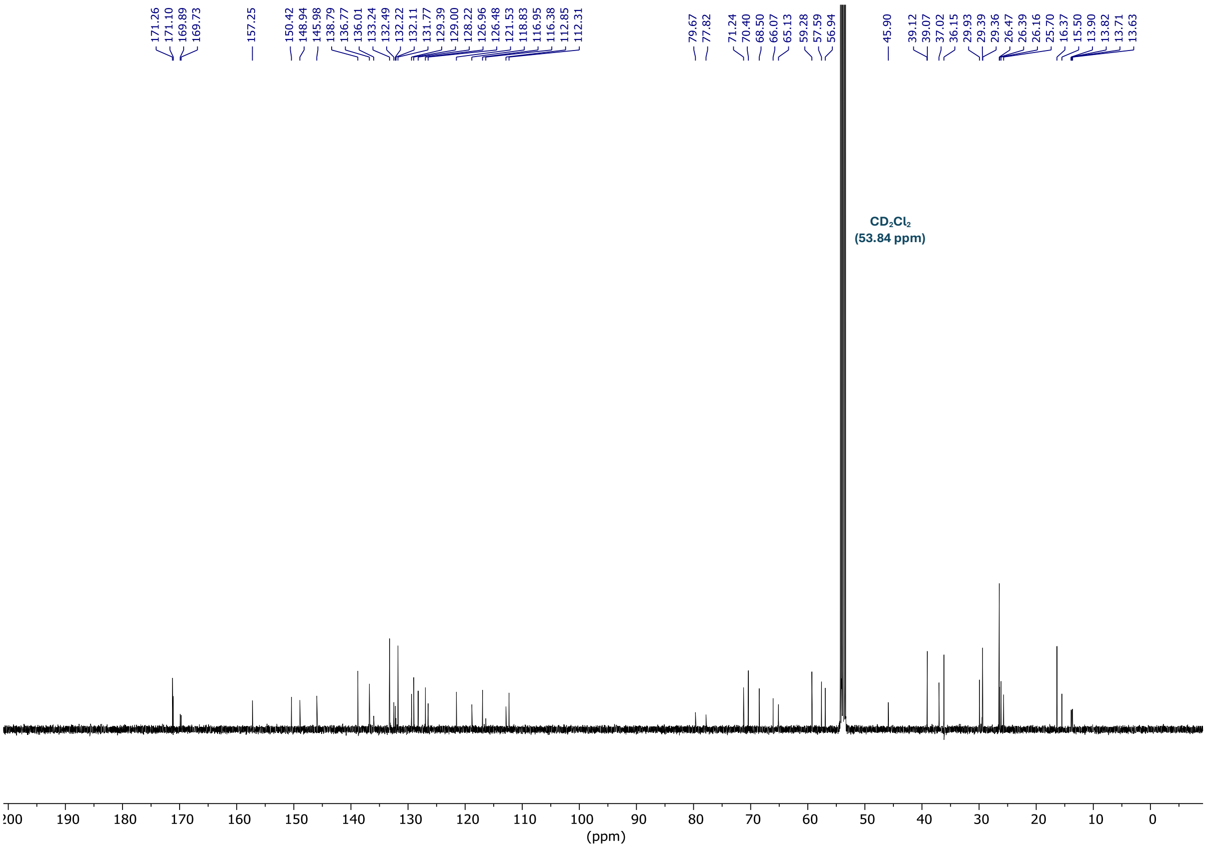

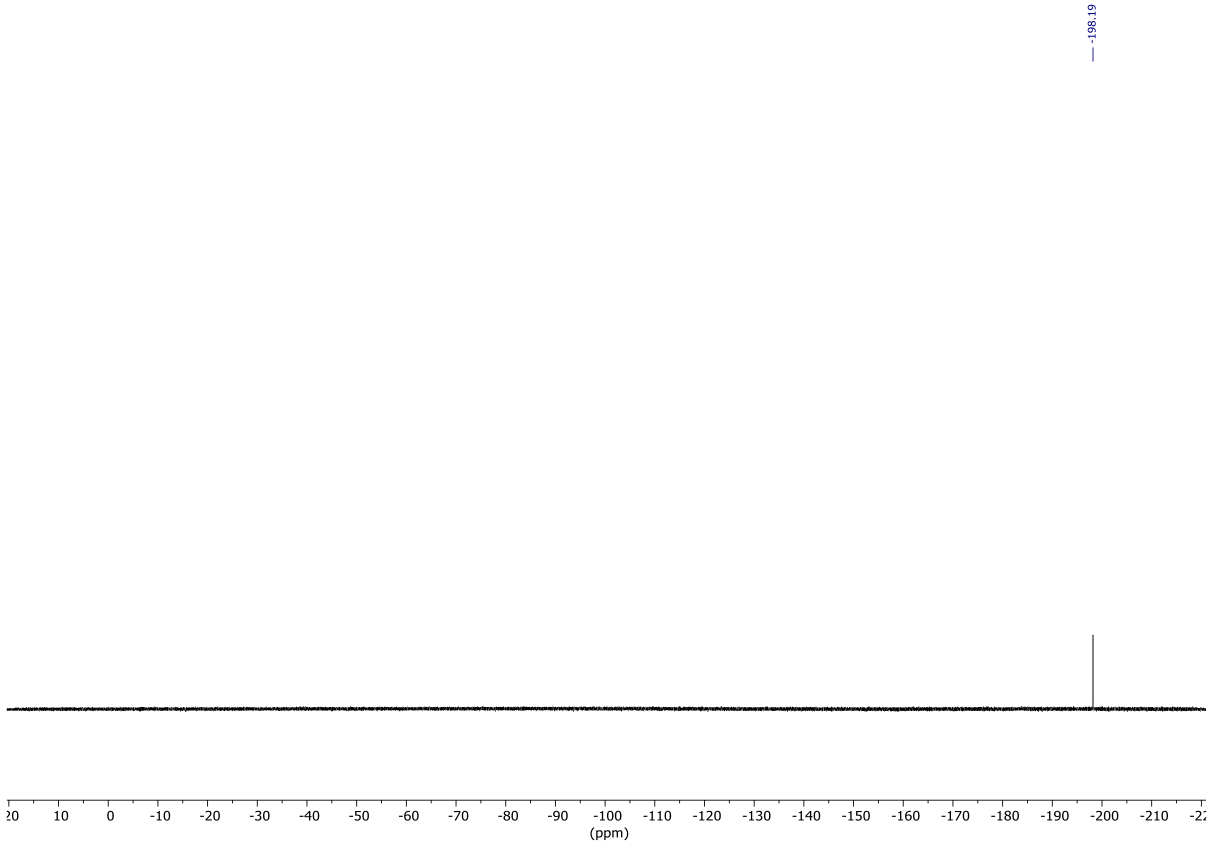

-198.19
20
10
0
-10
-20
-30
-40
-50
-60
-70
-80
-90
-100
-110
-120
-130
-140
-150
-160
-170
-180
-190
-200
-210
(ppm)

Compound 41. *^{1}H NMR (500 MHz, CD_2Cl_2), ^{13}C NMR (126 MHz, CD_2Cl_2) and ^{19}F NMR (471 MHz, CD_2Cl_2).*

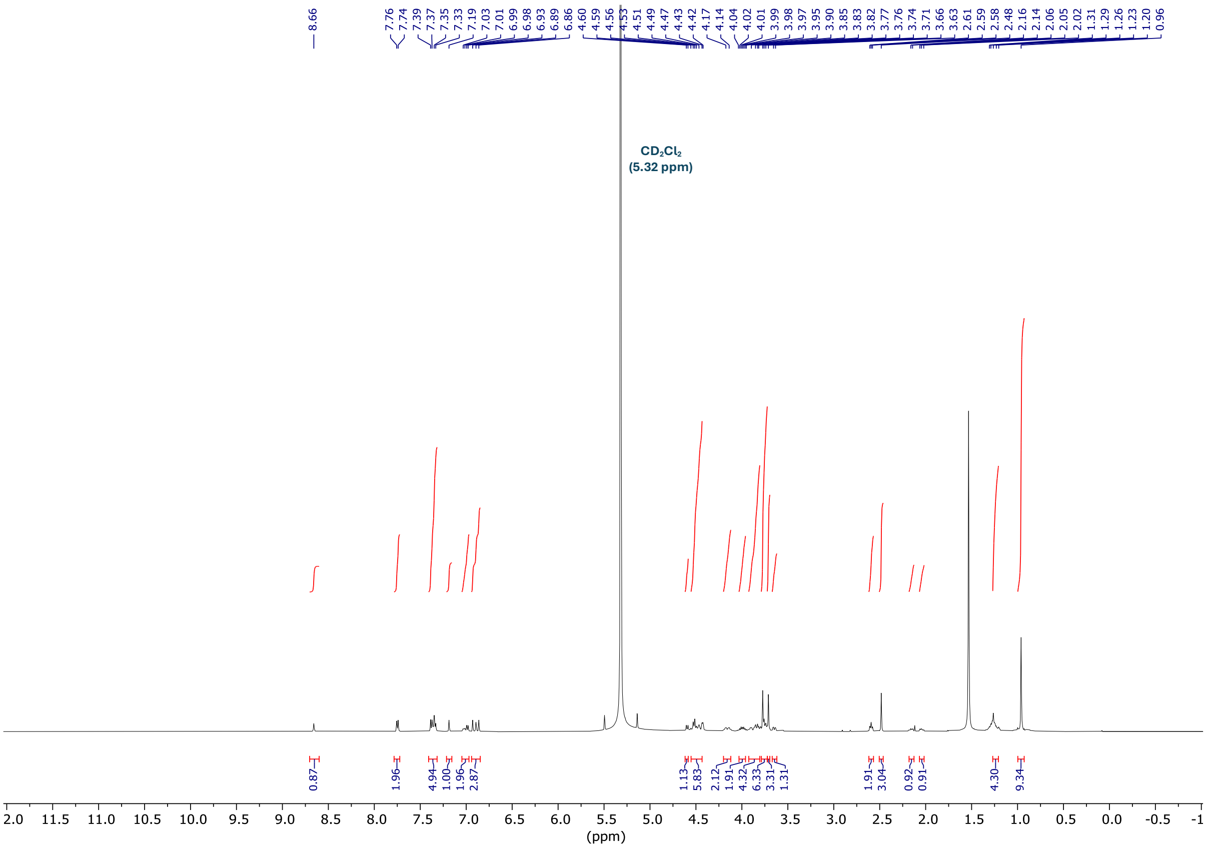

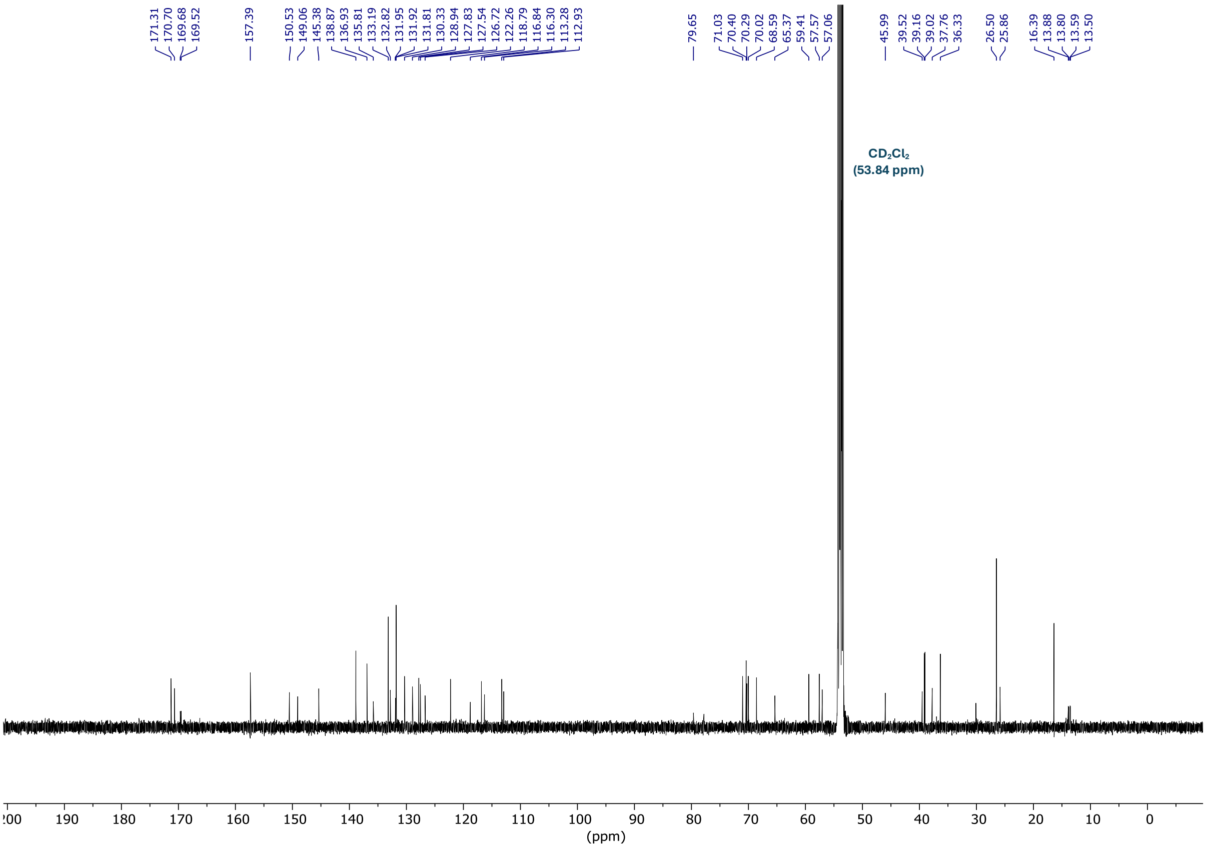

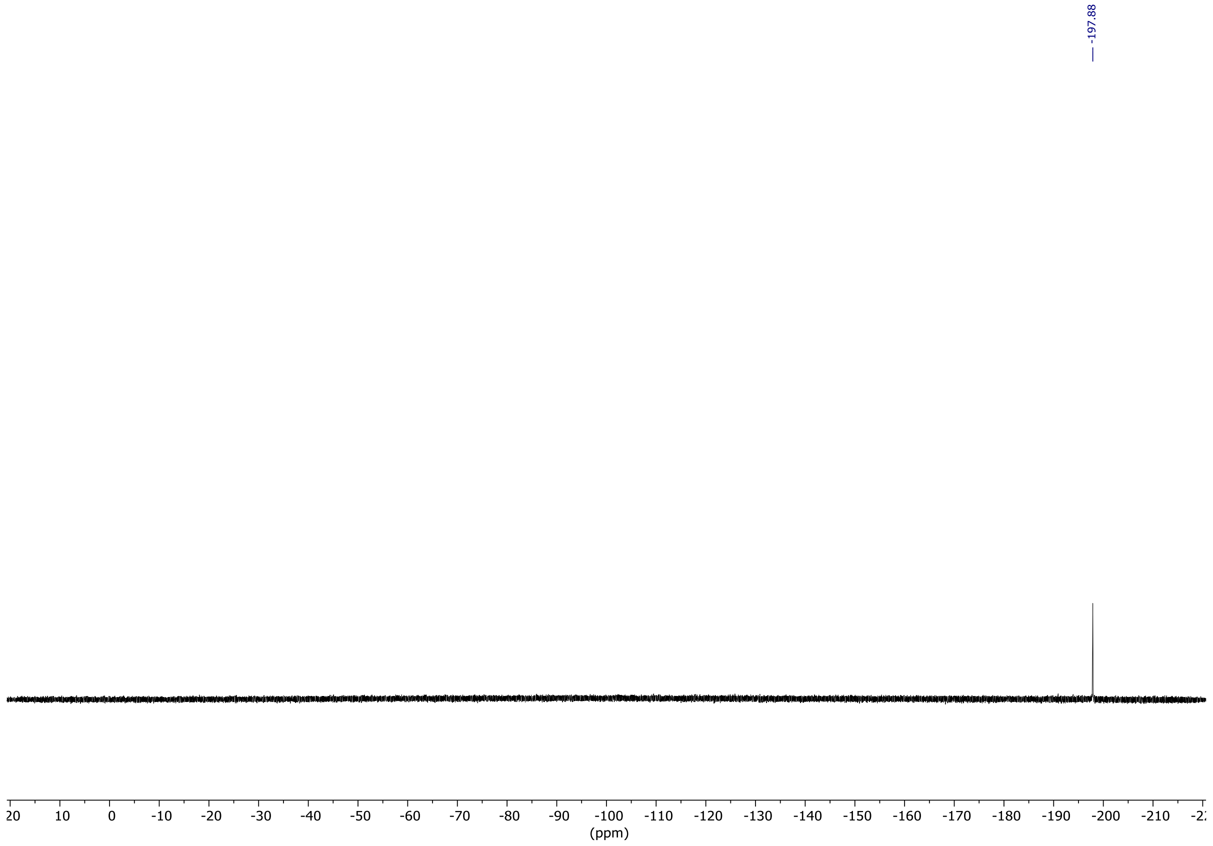
-197.88
20
10
0
-10
-20
-30
-40
-50
-60
-70
-80
-90
-100
-110
-120
-130
-140
-150
-160
-170
-180
-190
-200
-210
(ppm)

Compound 42. *^{1}H NMR (500 MHz, CD_2Cl_2), ^{13}C NMR (126 MHz, CD_2Cl_2) and ^{19}F NMR (377 MHz, CD_2Cl_2).*

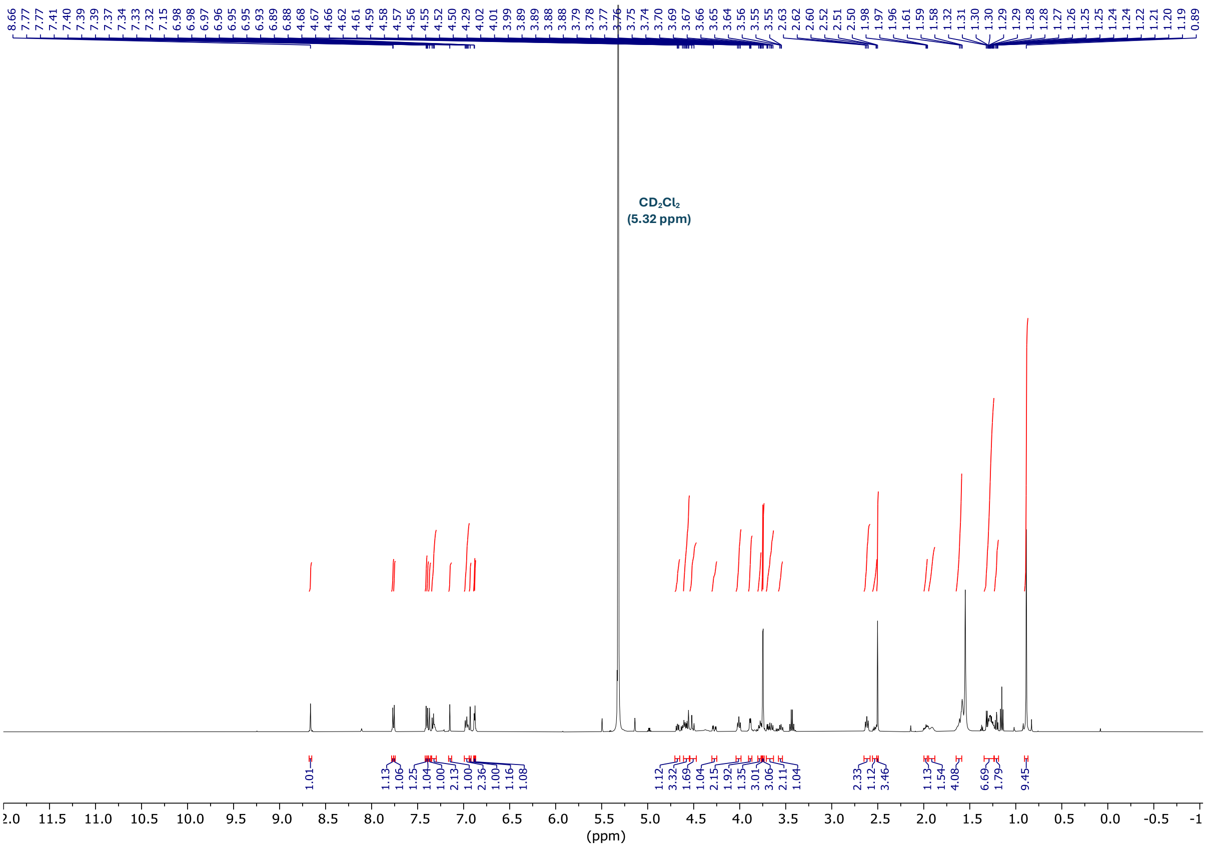

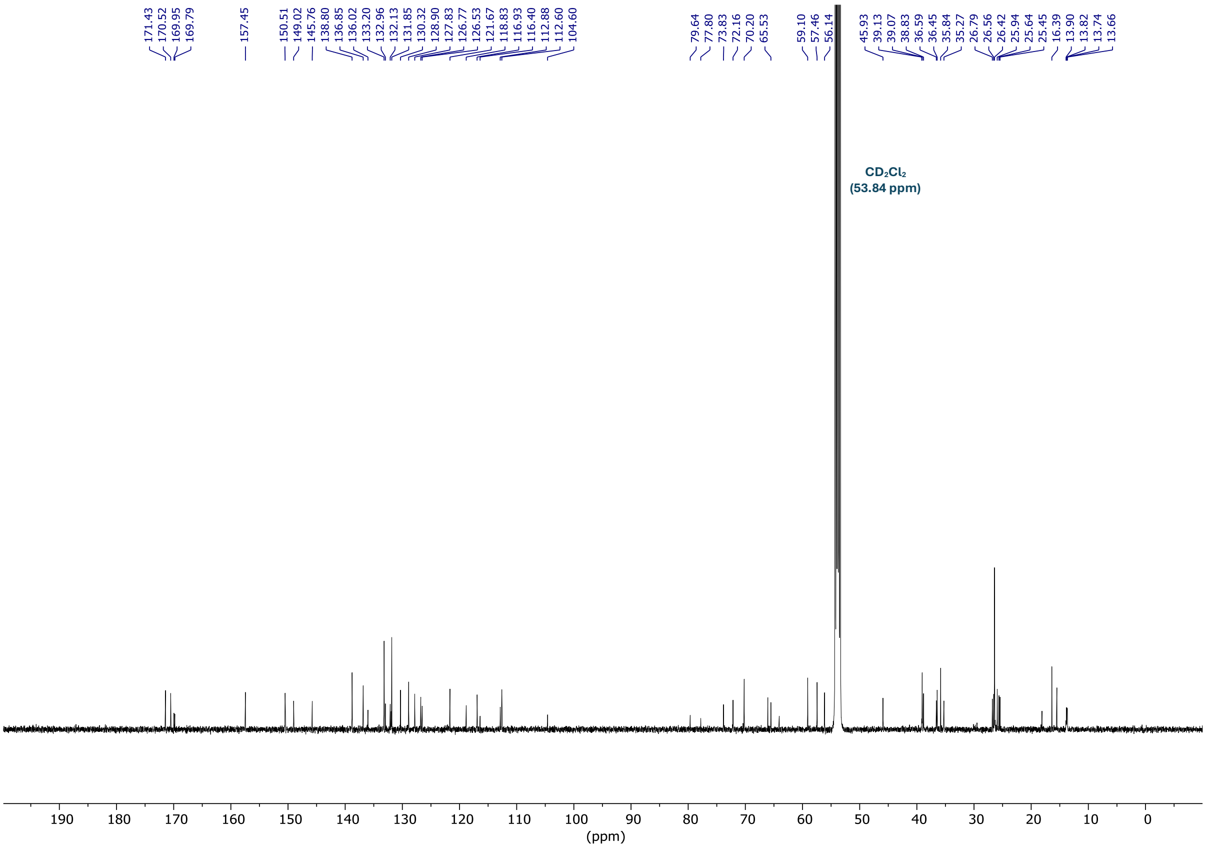

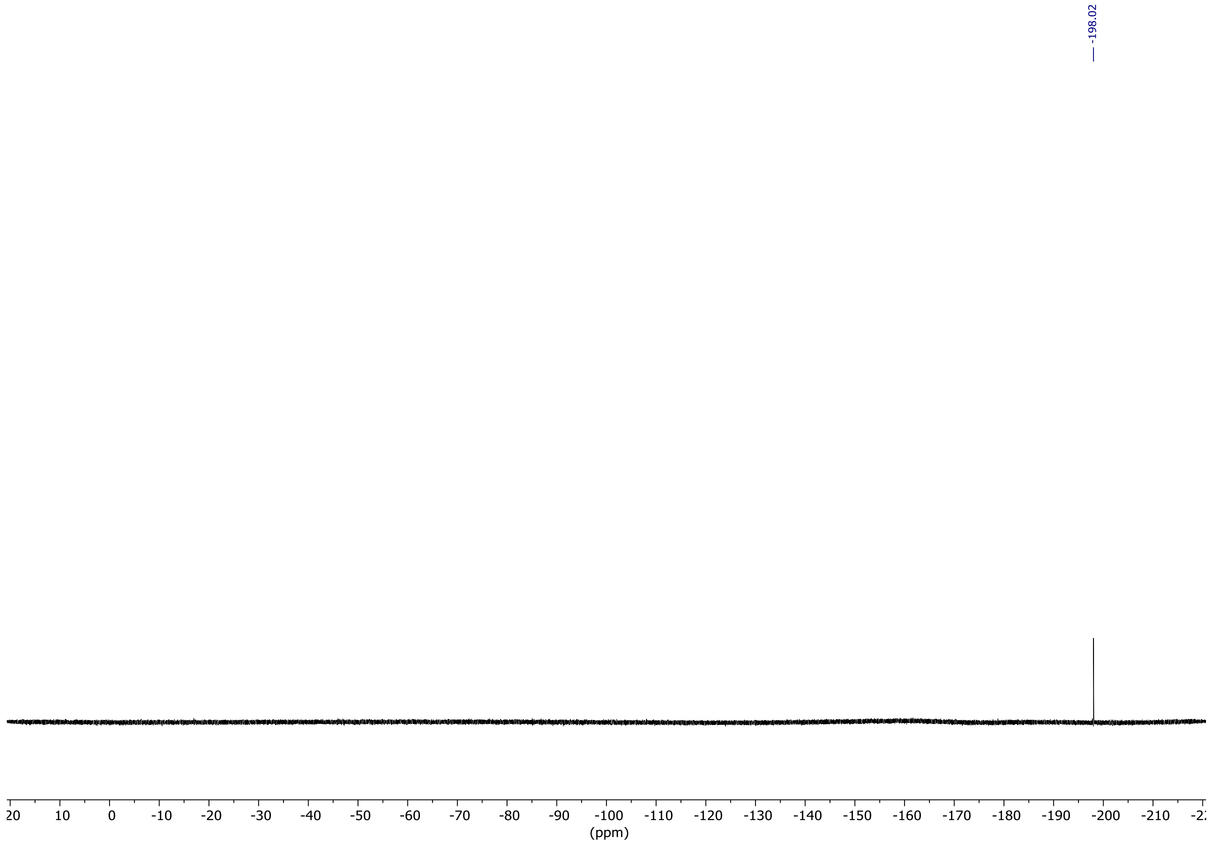
-198.02
20
10
0
-10
-20
-30
-40
-50
-60
-70
-80
-90
-100
-110
-120
-130
-140
-150
-160
-170
-180
-190
-200
-210
(ppm)

Compound 43. *^{1}H NMR (500 MHz, CD_2Cl_2), ^{13}C NMR (126 MHz, CD_2Cl_2) and ^{19}F NMR (377 MHz, CD_2Cl_2).*

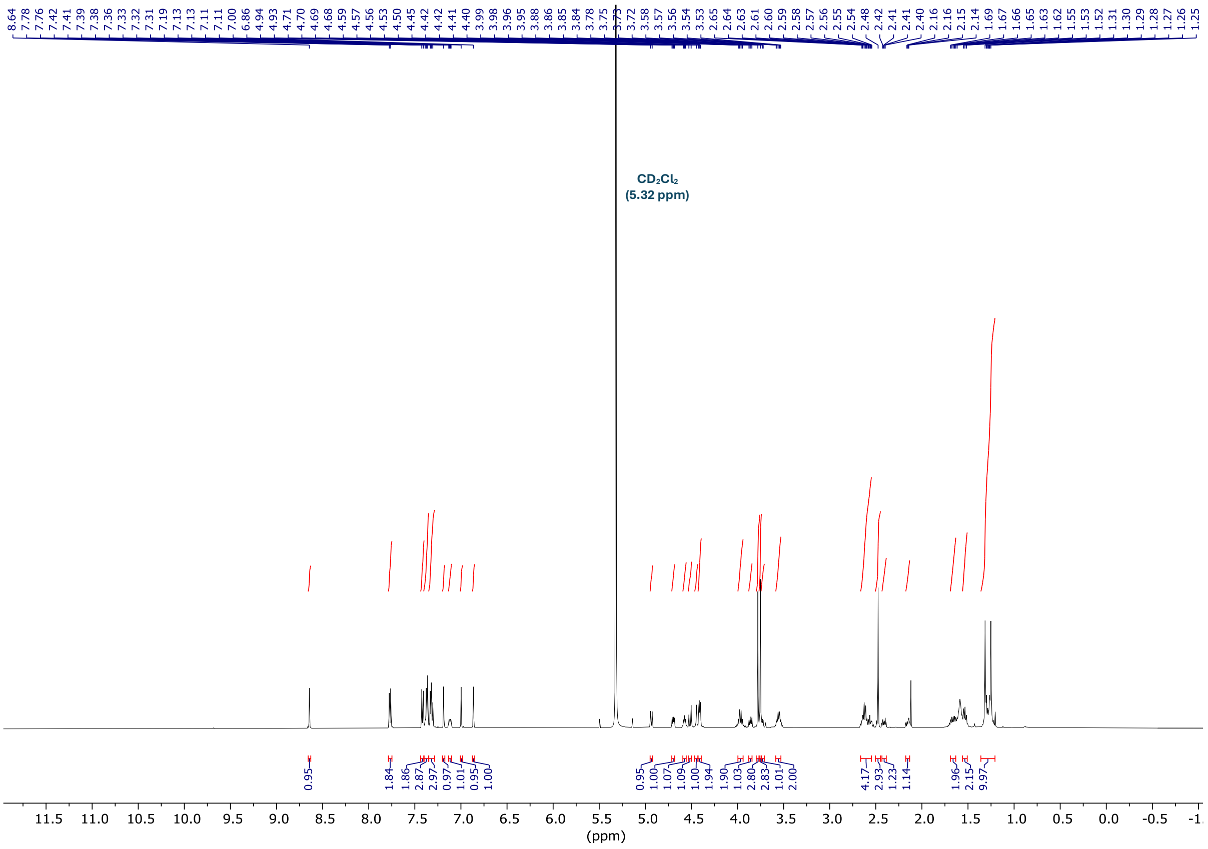

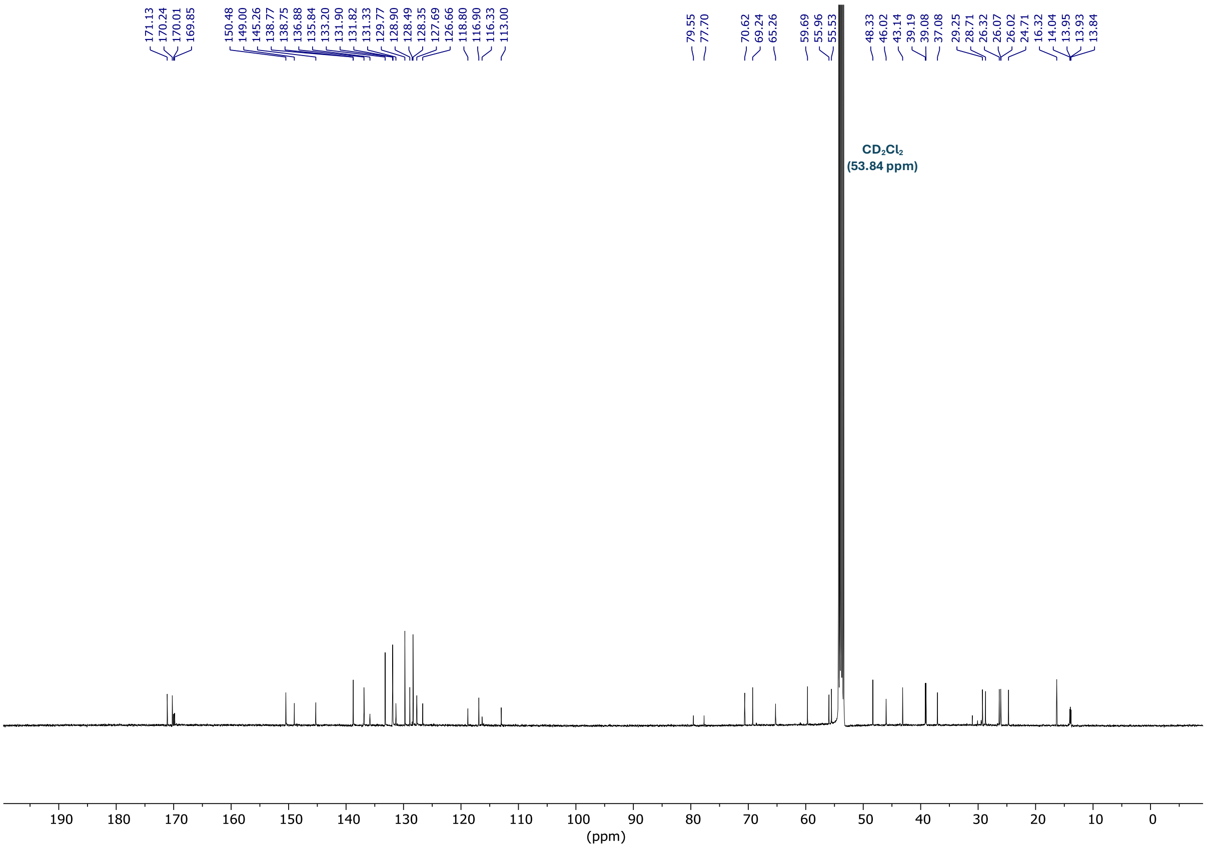

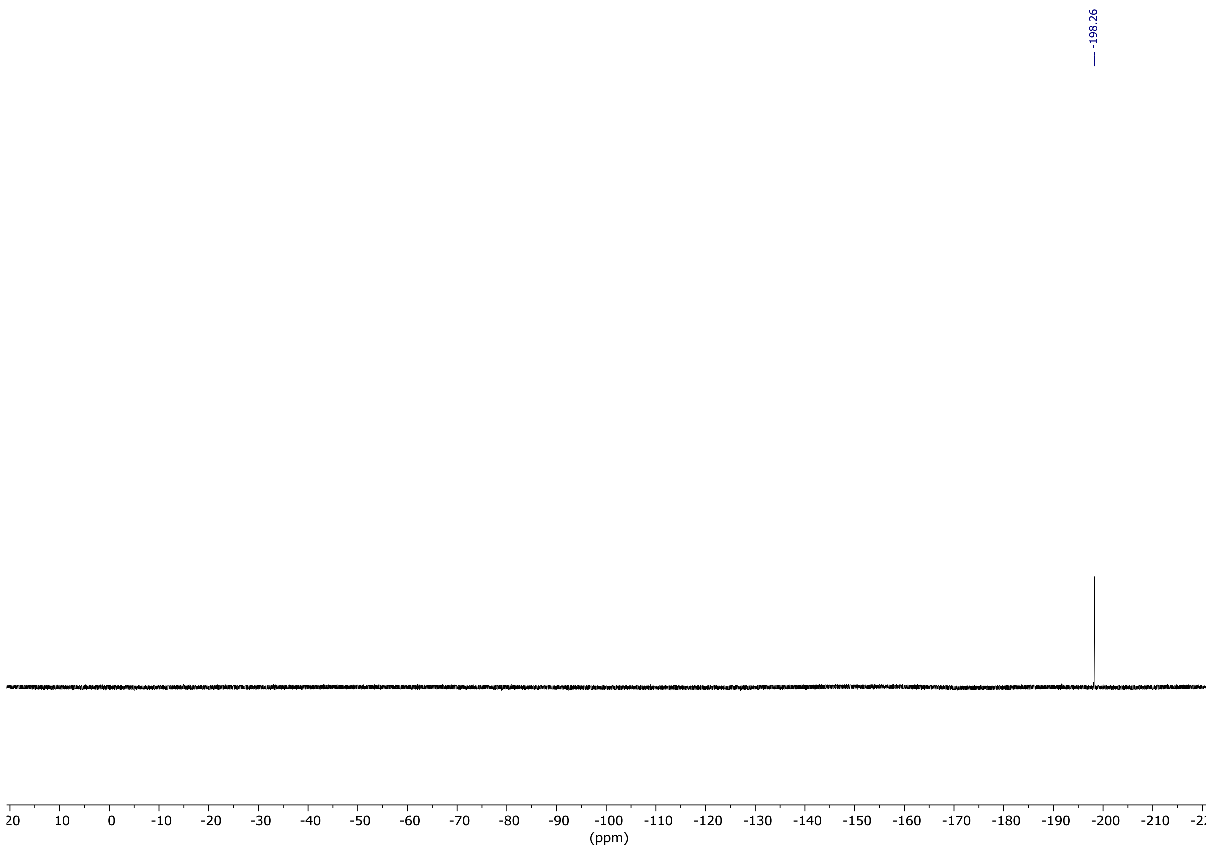
-198.26
20
10
0
-10
-20
-30
-40
-50
-60
-70
-80
-90
-100
-110
-120
-130
-140
-150
-160
-170
-180
-190
-200
-210
(ppm)

Compound 44. *^{1}H NMR (500 MHz, CD_2Cl_2), ^{13}C NMR (126 MHz, CD_2Cl_2) and ^{19}F NMR (377 MHz, CD_2Cl_2).*

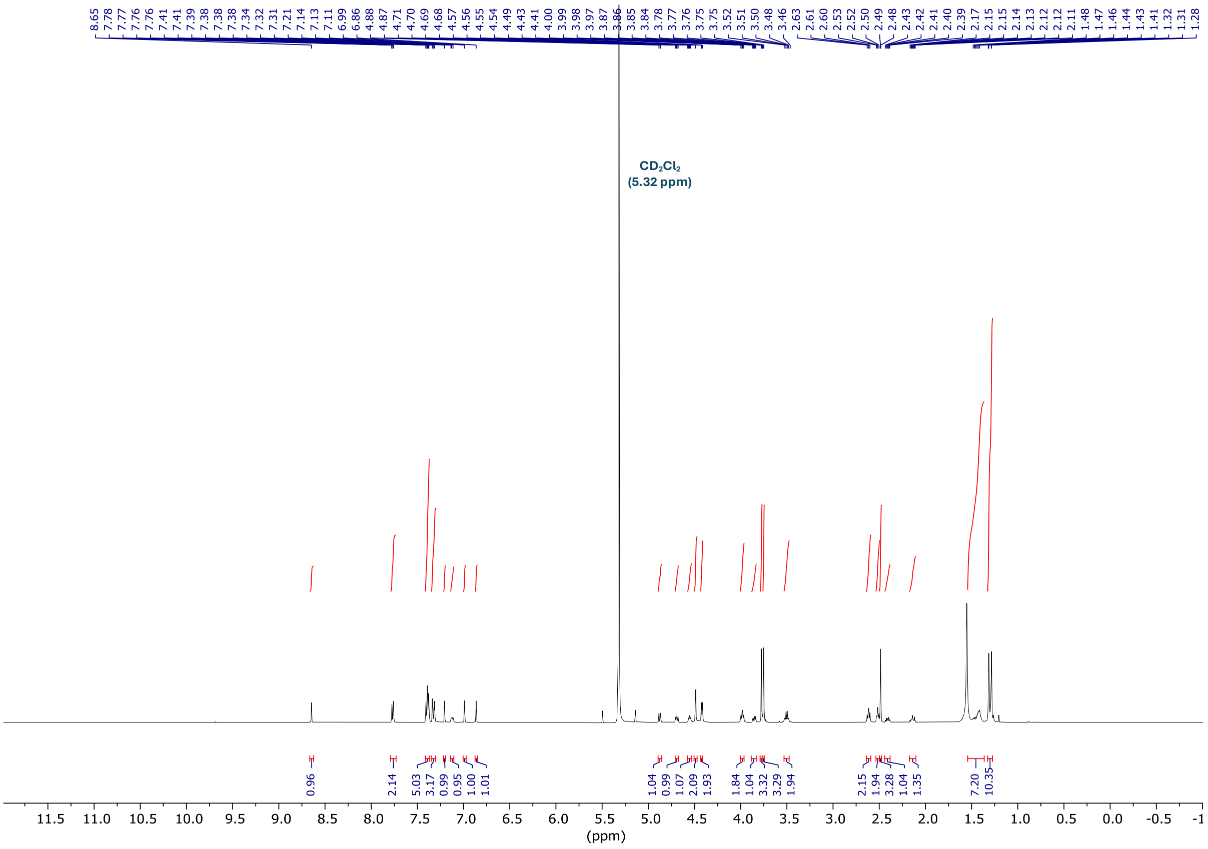

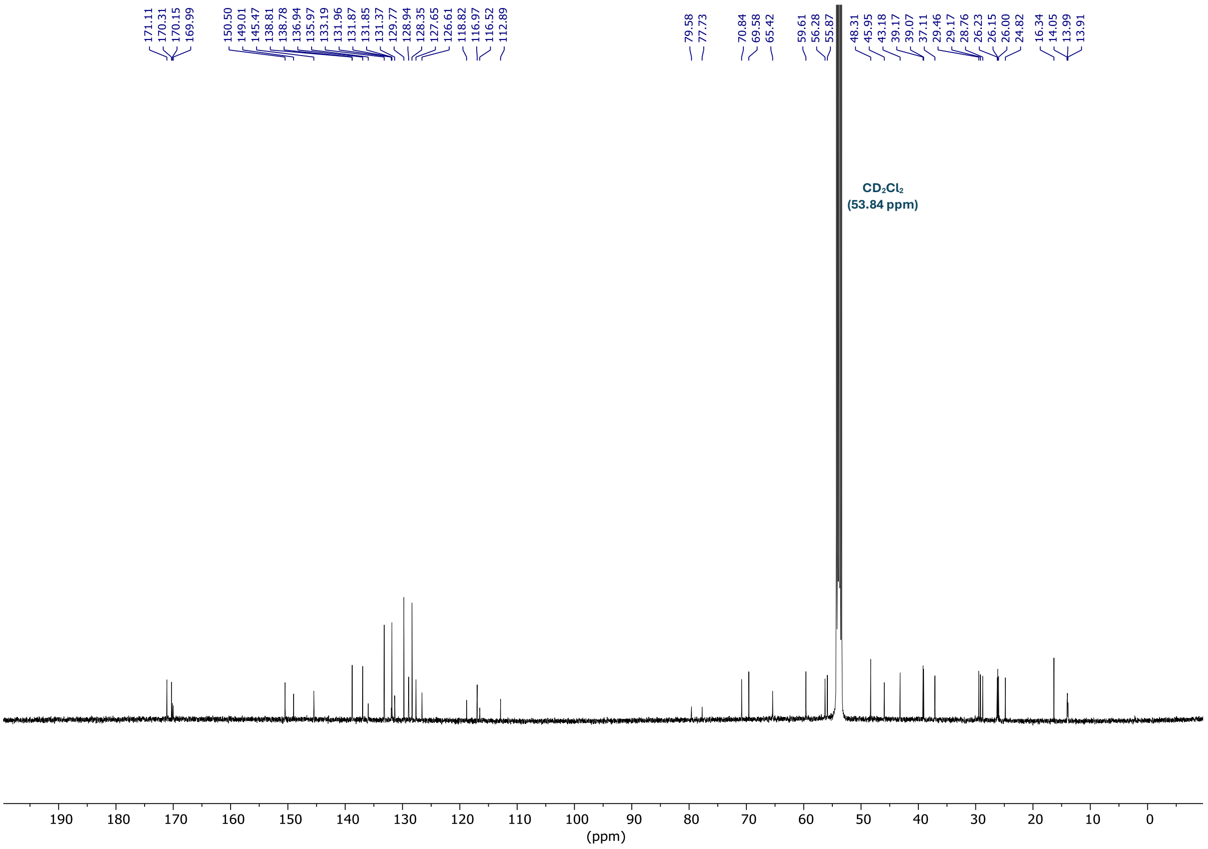

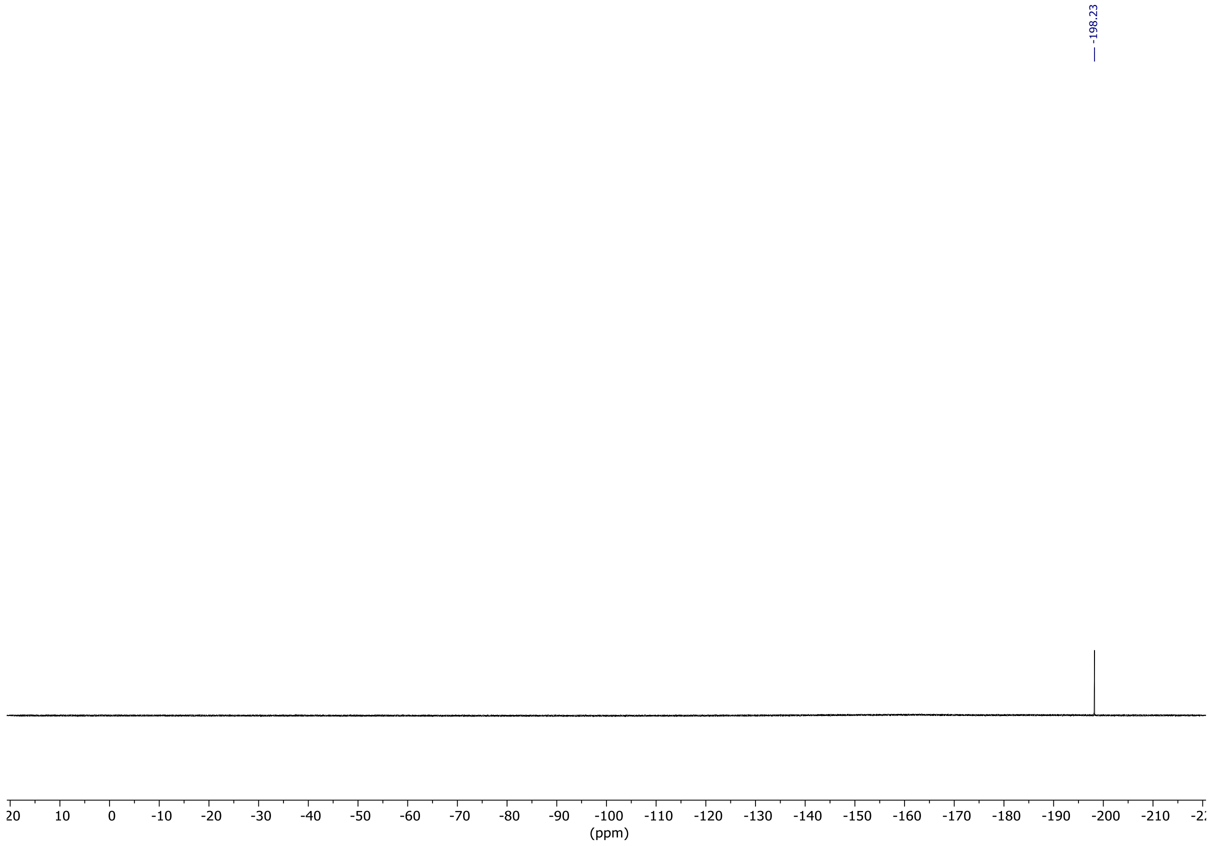
-198.23
20 10 0 -10 -20 -30 -40 -50 -60 -70 -80 -90 -100 -110 -120 -130 -140 -150 -160 -170 -180 -190 -200 -210
(ppm)

Compound 45. *^{1}H NMR (500 MHz, CD_2Cl_2) and ^{13}C NMR (126 MHz, CD_2Cl_2)*

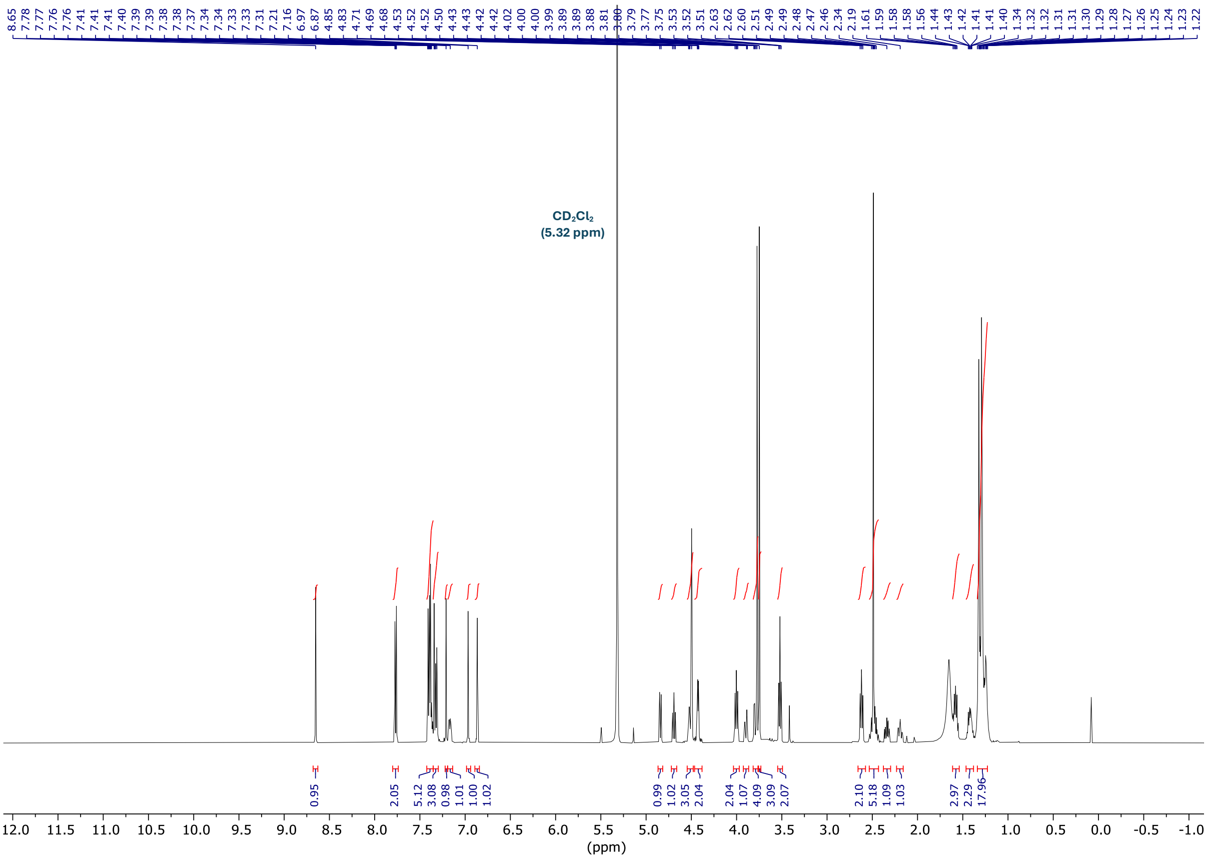

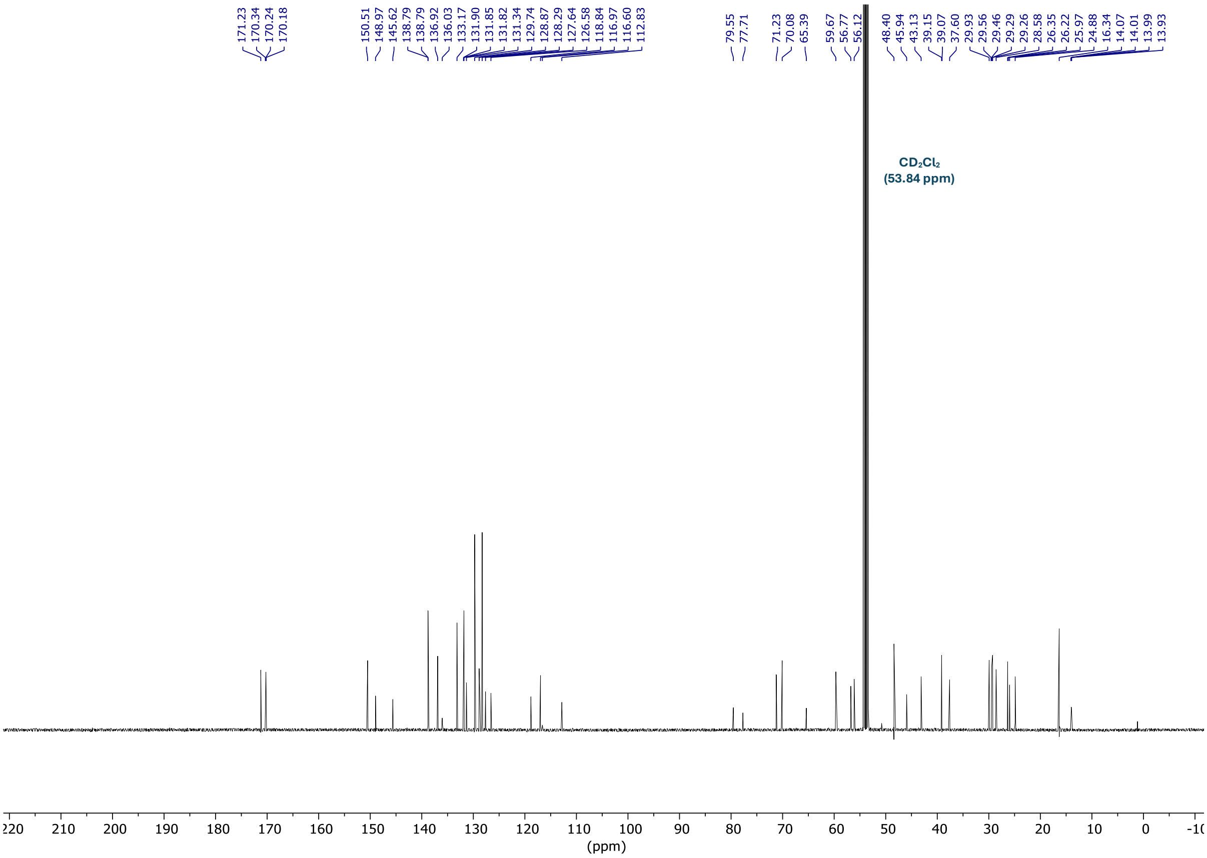

Compound 46. *^{1}H NMR (500 MHz, CD_2Cl_2), ^{13}C NMR (126 MHz, CD_2Cl_2) and ^{19}F NMR (377 MHz, CD_2Cl_2).*

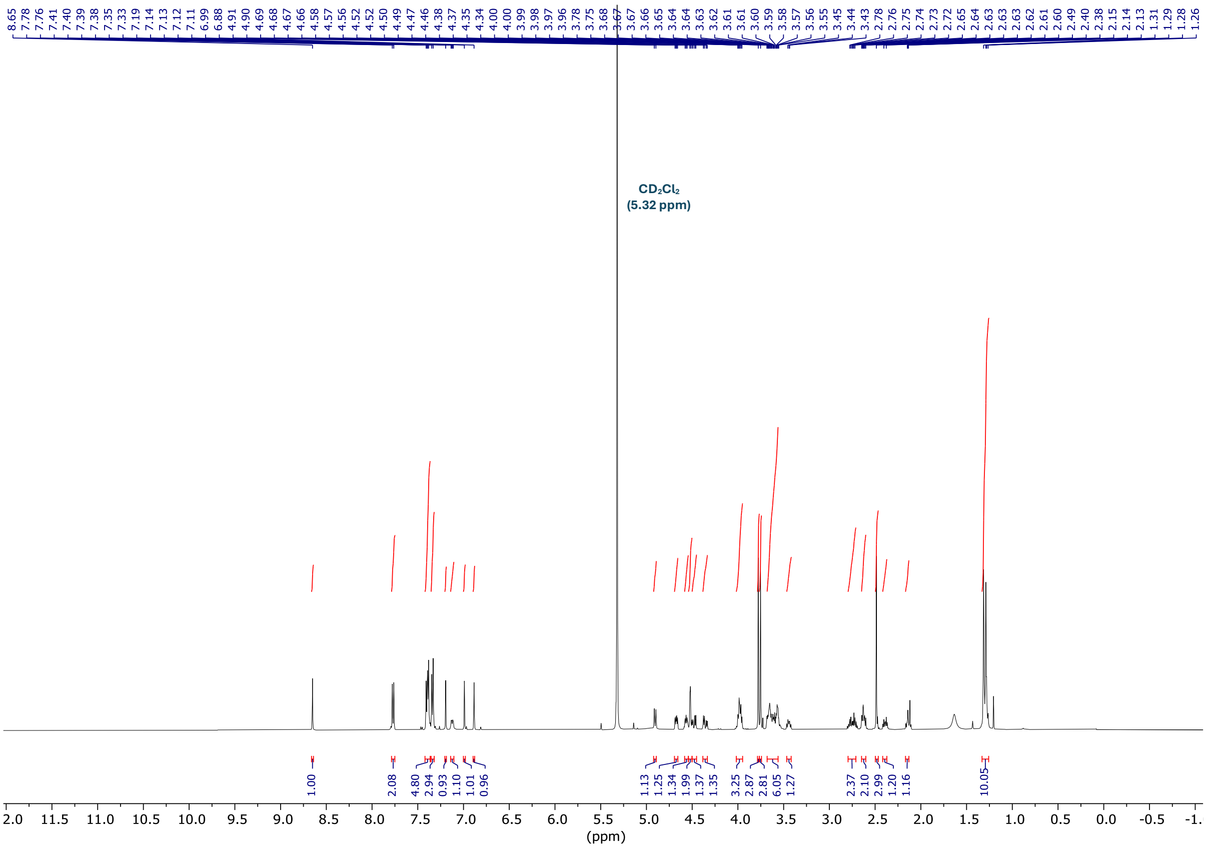

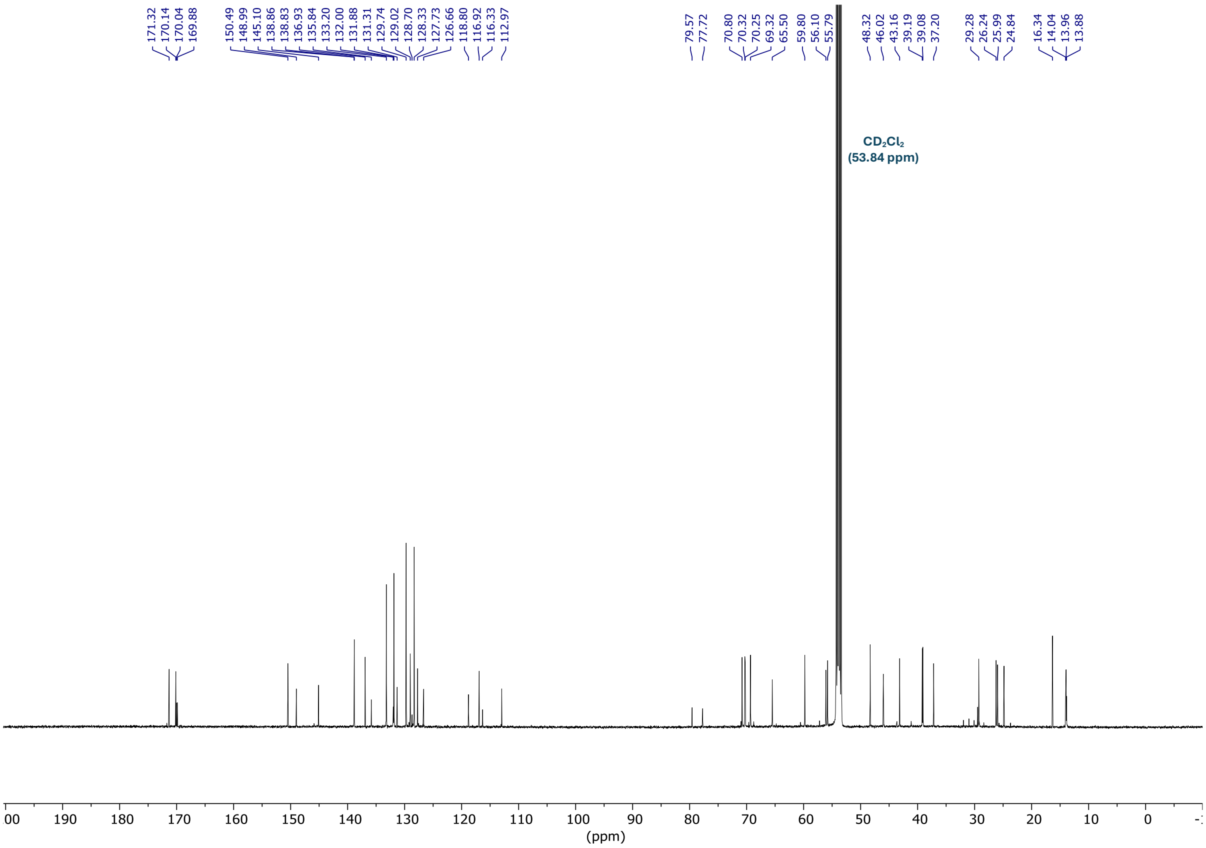

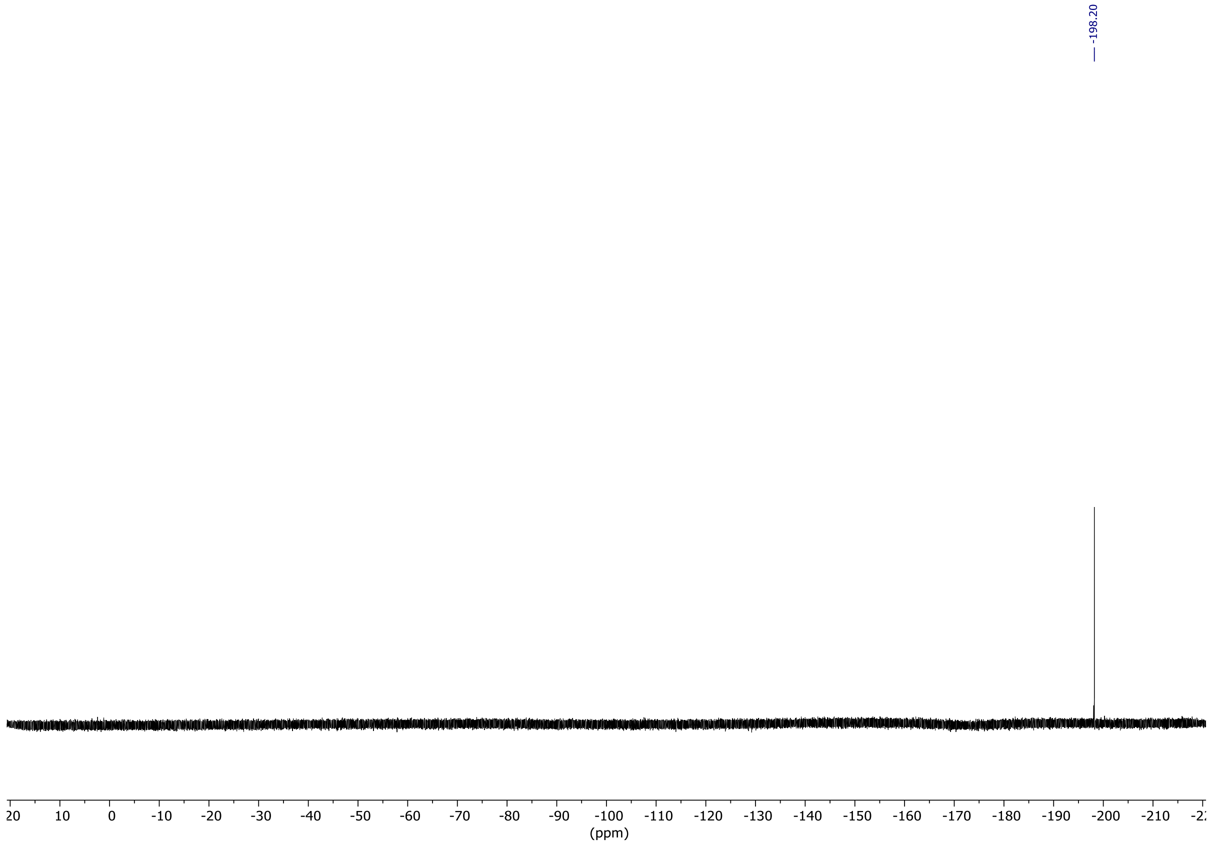
-198.20
20
10
0
-10
-20
-30
-40
-50
-60
-70
-80
-90
-100
-110
-120
-130
-140
-150
-160
-170
-180
-190
-200
-210
(ppm)

Compound 47. *^{1}H NMR (500 MHz, CD_2Cl_2) and ^{13}C NMR (126 MHz, CD_2Cl_2)*

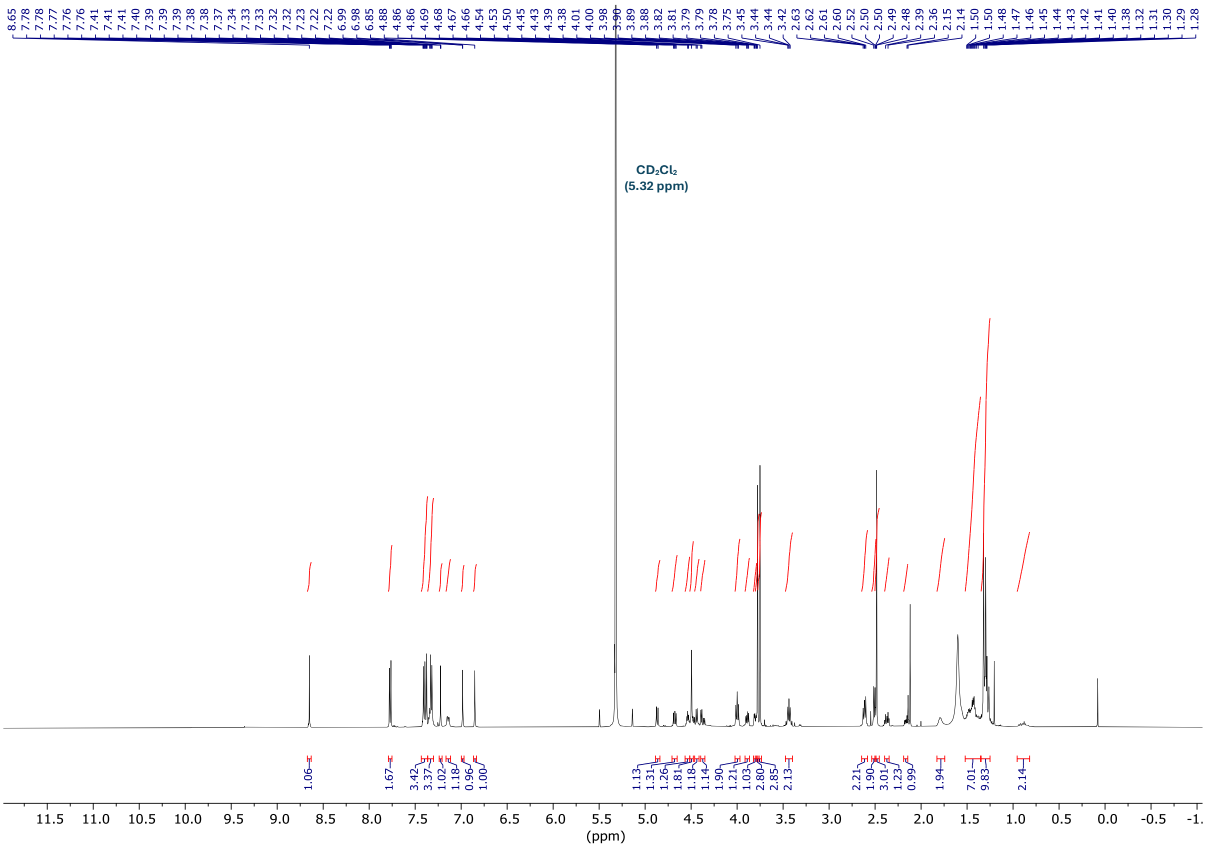

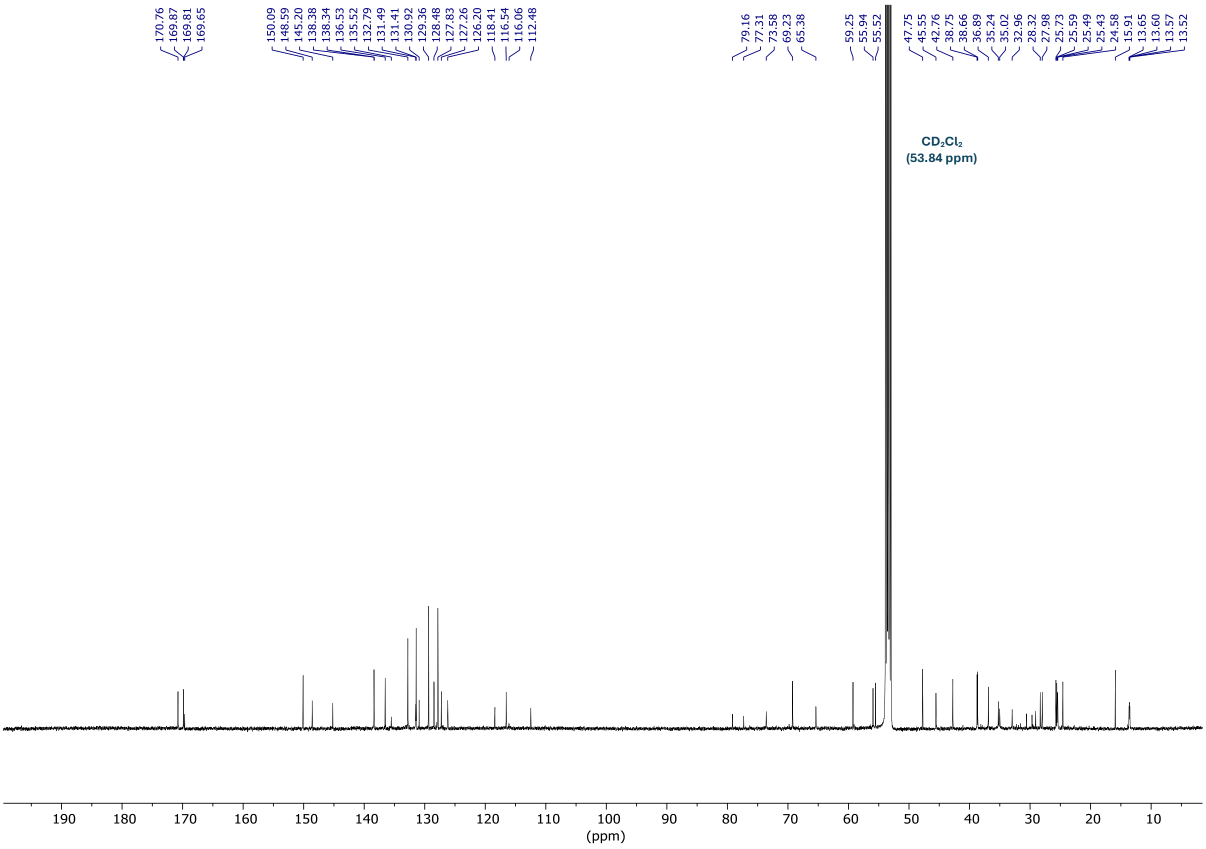

LCMS Spectra

Compound 6. *LC-MS*

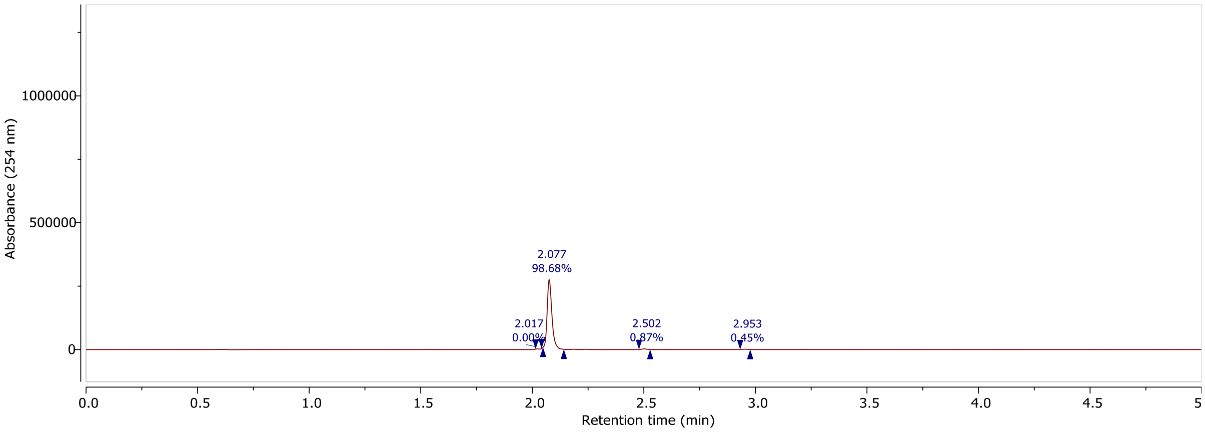

	RT	Area	Height	Total Height %	Total Area %	Start time	End time
1	2.017	27.800	37.400	0.01	0.00	2.015	2.048
2	2.077	4060562.000	272613.300	98.50	98.68	2.042	2.142
3	2.502	35707.500	2684.400	0.97	0.87	2.478	2.528
4	2.953	18589.000	1423.259	0.51	0.45	2.932	2.977

Compound 7. *LC-MS*

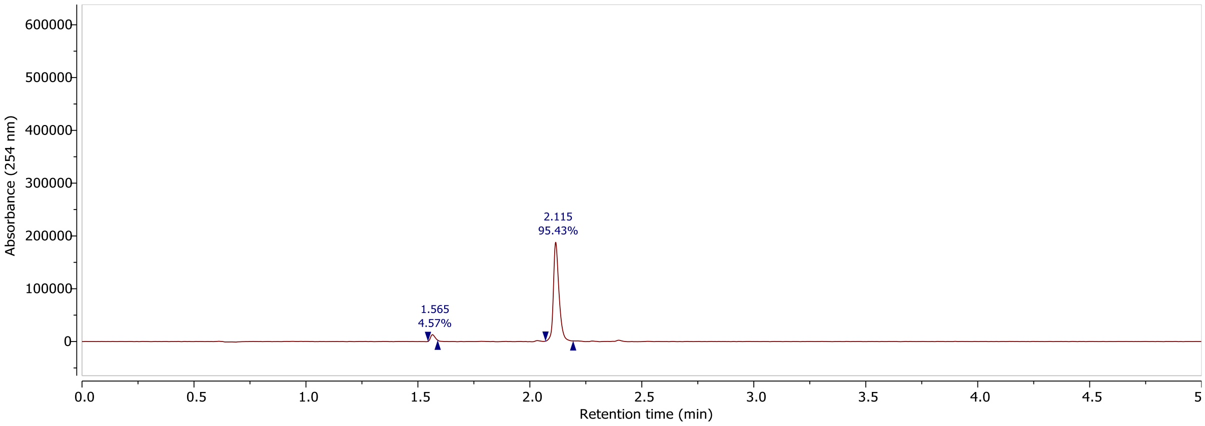

	RT	Area	Height	Total Height %	Total Area %	Start time	End time
1	1.565	153881.000	11846.000	5.94	4.57	1.545	1.588
2	2.115	3215011.500	187484.635	94.06	95.43	2.070	2.193

Compound 8. *LC-MS*

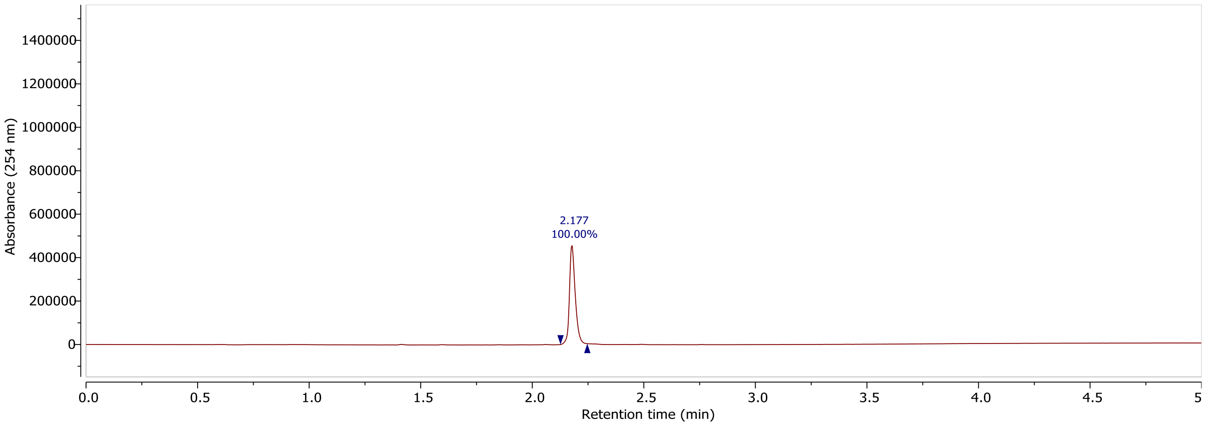

	RT	Area	Height	Total Height %	Total Area %	Start time	End time
1	2.177	8183585.500	455674.750	100.00	100.00	2.127	2.247

Compound 9. *LC-MS*

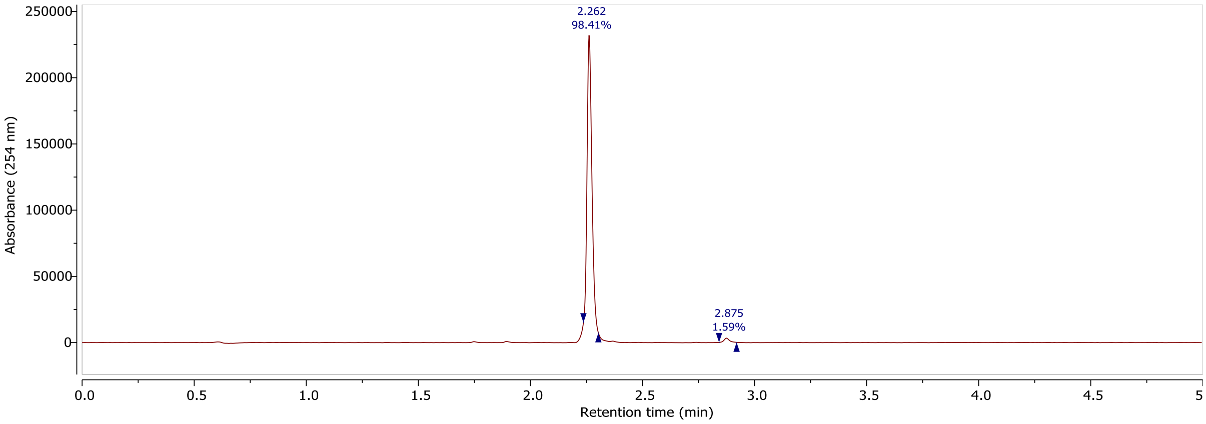

	RT	Height	Area	Total Height %	Total Area %	Start time	End time
1	2.262	219860.250	3243792.000	98.56	98.41	2.237	2.303
2	2.875	3212.979	52480.000	1.44	1.59	2.842	2.920

Compound 10. *LC-MS*

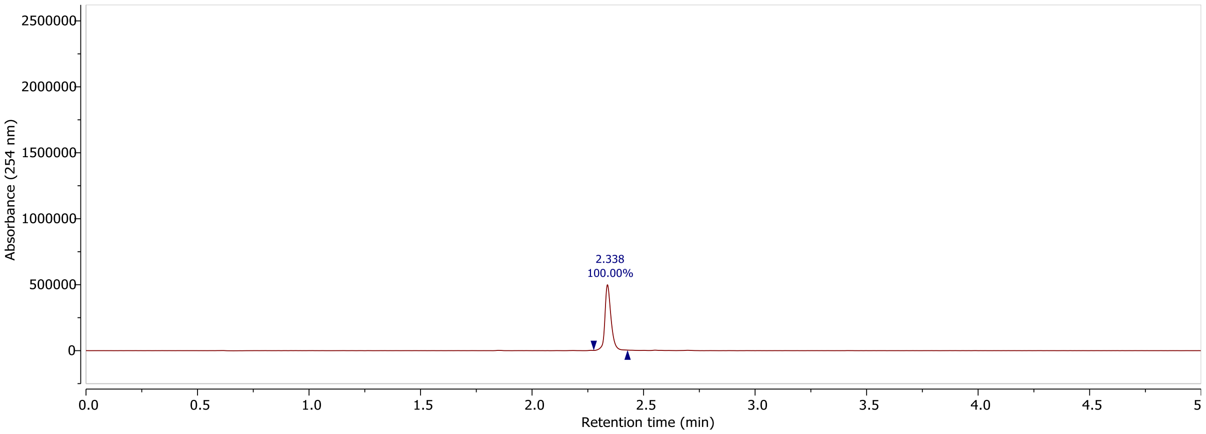

	RT	Area	Height	Total Height %	Total Area %	Start time	End time
1	2.338	9332675.418	497824.275	100.00	100.00	2.277	2.428

Compound 11. *LC-MS*

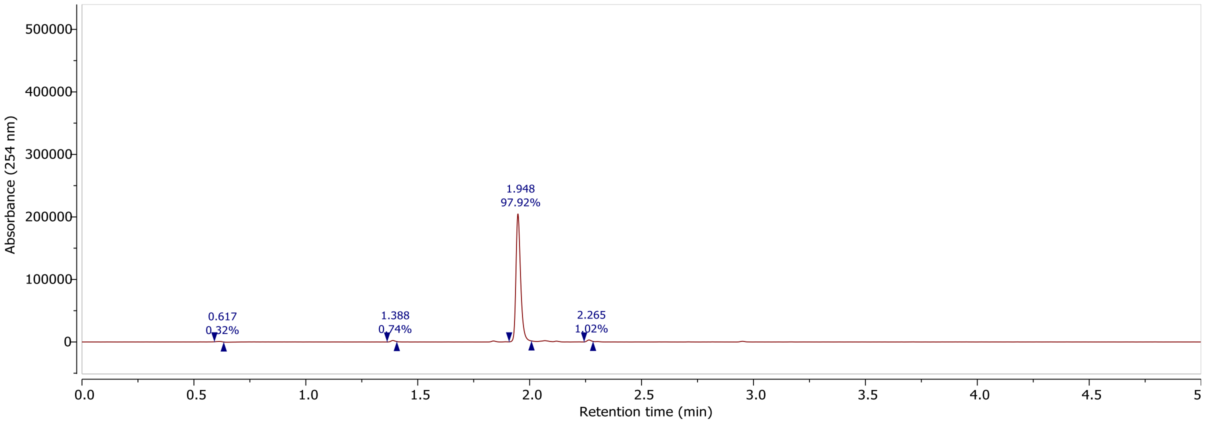

	RT	Area	Height	Total Height %	Total Area %	Start time	End time
1	0.617	9674.000	639.000	0.30	0.32	0.592	0.633
2	1.388	22181.269	2030.077	0.97	0.74	1.363	1.407
3	1.948	2946183.000	204228.200	97.45	97.92	1.908	2.008
4	2.265	30671.500	2675.958	1.28	1.02	2.243	2.283

Compound 12. *LC-MS*

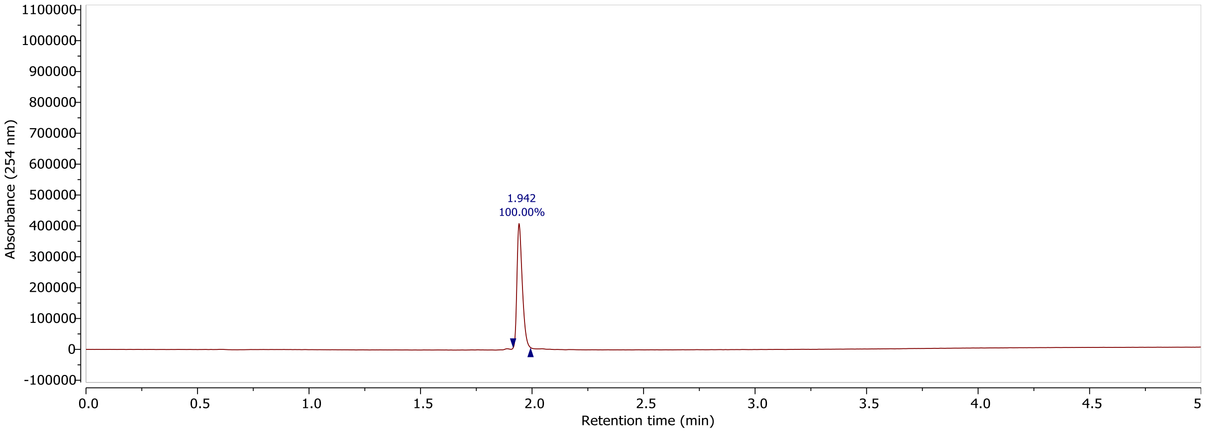

	RT	Area	Height	Total Height %	Total Area %	Start time	End time
1	1.942	1321289411.836	74261392.359	100.00	100.00	1.900	2.007

Compound 13. *LC-MS*

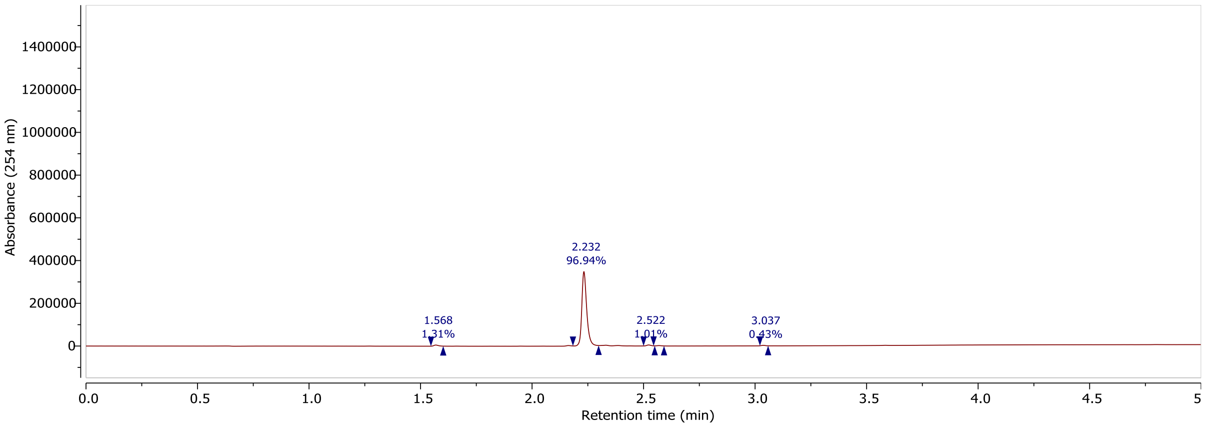

	RT	Area	Height	Total Height %	Total Area %	Start time	End time
1	1.568	70815.000	5127.424	1.42	1.31	1.547	1.602
2	2.232	5254668.043	346922.652	96.39	96.94	2.183	2.298
3	2.522	54805.500	4352.767	1.21	1.01	2.500	2.550
4	2.568	16699.643	1434.000	0.40	0.31	2.545	2.592
5	3.037	23456.500	2070.136	0.58	0.43	3.022	3.058

Compound 14. *LC-MS*

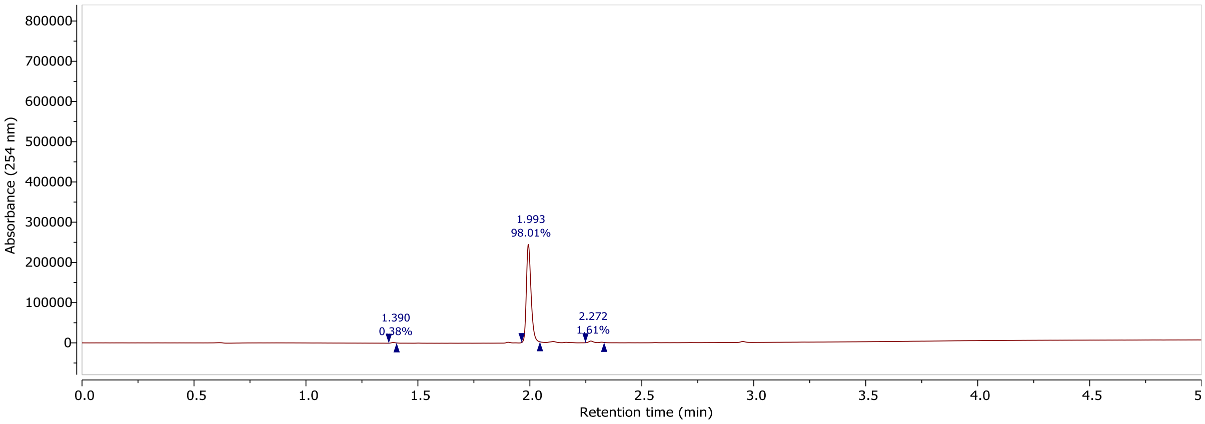

	RT	Area	Height	Total Height %	Total Area %	Start time	End time
1	1.390	13523.000	1256.571	0.50	0.38	1.370	1.405
2	1.993	3506662.000	244151.388	97.91	98.01	1.963	2.045
3	2.272	57521.320	3947.240	1.58	1.61	2.248	2.332

Compound 15. *LC-MS*

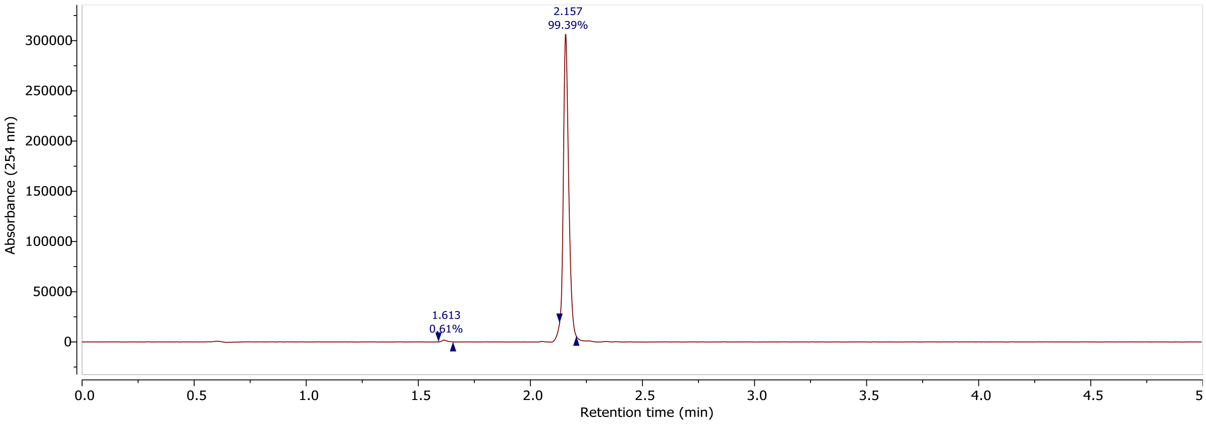

	RT	Height	Area	Total Height %	Total Area %	Start time	End time
1	1.613	1827.718	28496.000	0.62	0.61	1.590	1.655
2	2.157	292338.311	4631123.000	99.38	99.39	2.130	2.205

Compound 16. *LC-MS*

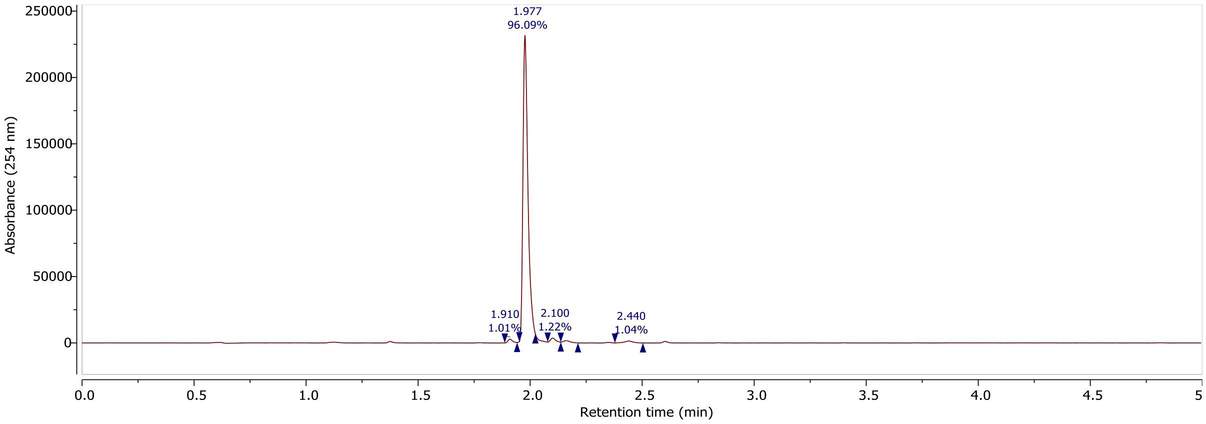

	RT	Height	Area	Total Height %	Total Area %	Start time	End time
1	1.910	2727.545	36574.000	1.15	1.01	1.887	1.942
2	1.977	228800.465	3475768.000	96.43	96.09	1.952	2.023
3	2.100	3035.086	44224.000	1.28	1.22	2.078	2.137
4	2.163	1250.348	23180.500	0.53	0.64	2.137	2.213
5	2.440	1456.941	37452.779	0.61	1.04	2.378	2.503

Compound 17. *LC-MS*

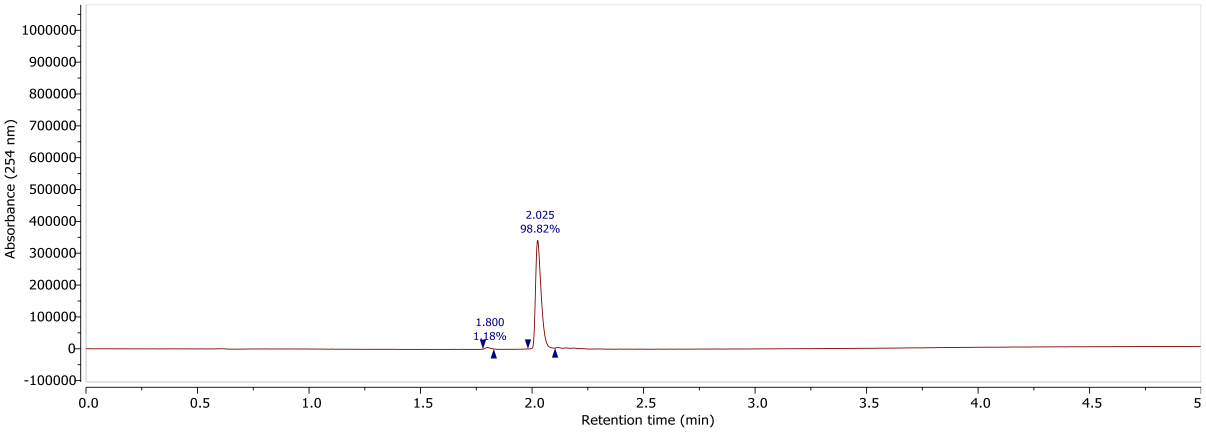

	RT	Area	Height	Total Height %	Total Area %	Start time	End time
1	1.800	70273.000	4915.103	1.42	1.18	1.780	1.828
2	2.025	5877467.178	340615.411	98.58	98.82	1.982	2.103

Compound 18. *LC-MS*

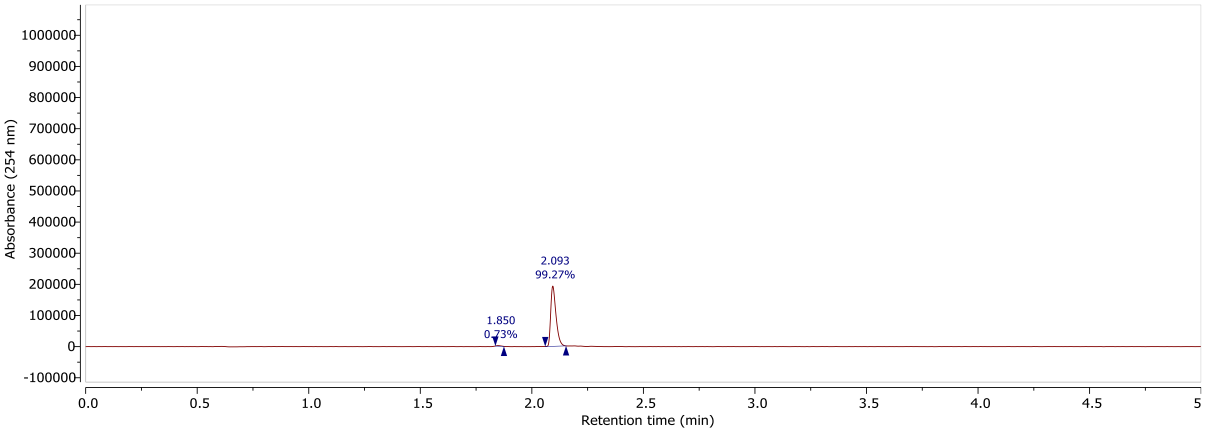

	RT	Area	Height	Total Height %	Total Area %	Start time	End time
1	1.850	23215.000	1726.696	0.88	0.73	1.837	1.875
2	2.093	3176648.268	193713.286	99.12	99.27	2.060	2.153

Compound 19. *LC-MS*

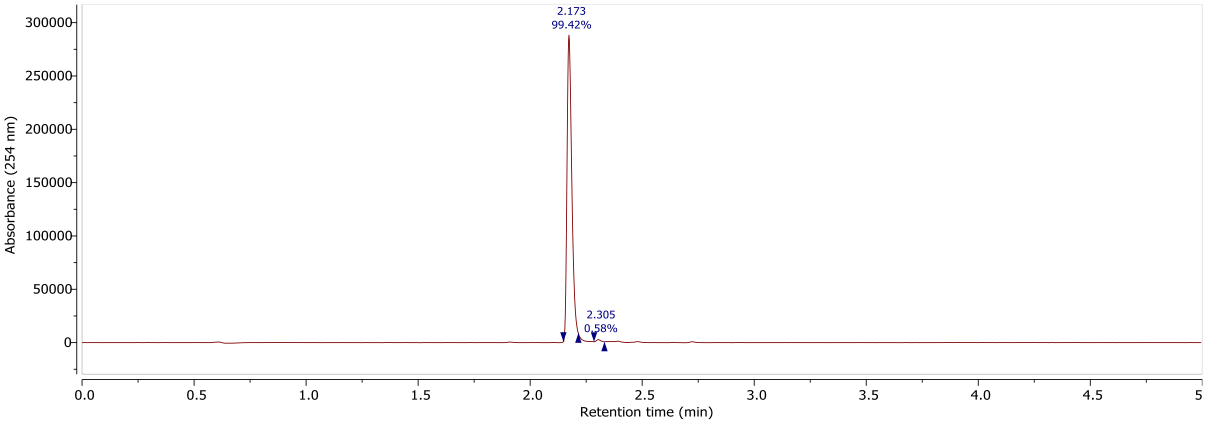

	RT	Height	Area	Total Height %	Total Area %	Start time	End time
1	2.173	284672.000	4162725.000	99.30	99.42	2.148	2.215
2	2.305	2014.000	24265.500	0.70	0.58	2.285	2.332

Compound 20. *LC-MS*

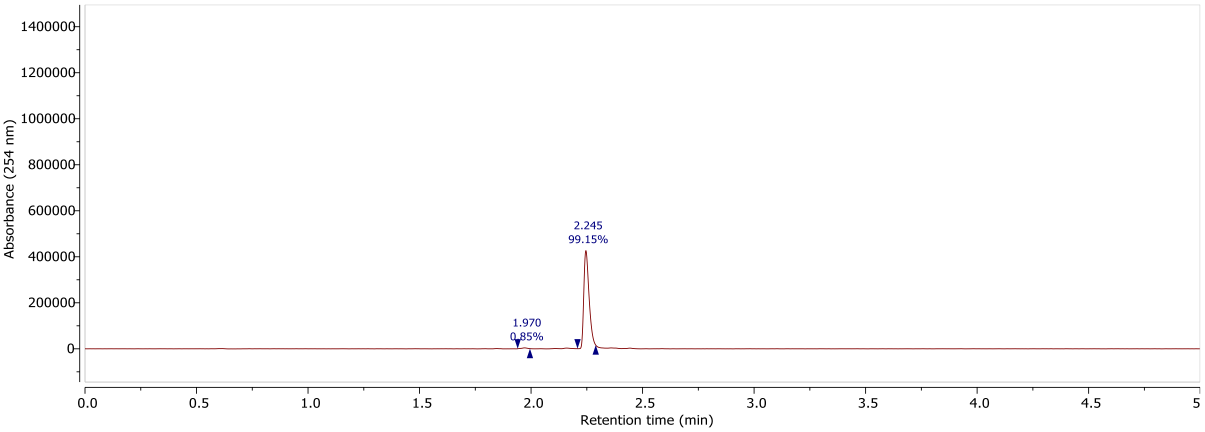

	RT	Area	Height	Total Height %	Total Area %	Start time	End time
1	1.970	58236.000	3484.091	0.82	0.85	1.940	1.995
2	2.245	6770822.612	418872.204	99.18	99.15	2.208	2.290

Compound 21. *LC-MS*

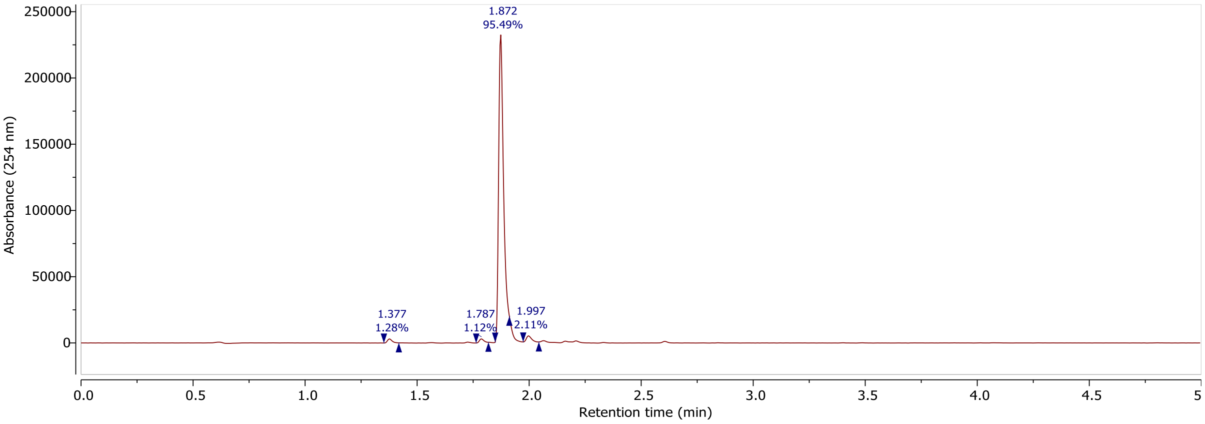

	RT	Height	Area	Total Height %	Total Area %	Start time	End time
1	1.377	3042.750	43699.000	1.29	1.28	1.352	1.418
2	1.787	2897.394	38292.000	1.23	1.12	1.763	1.818
3	1.872	224667.947	3260161.500	95.52	95.49	1.848	1.912
4	1.997	4591.333	72089.000	1.95	2.11	1.973	2.043

Compound 22. *LC-MS*

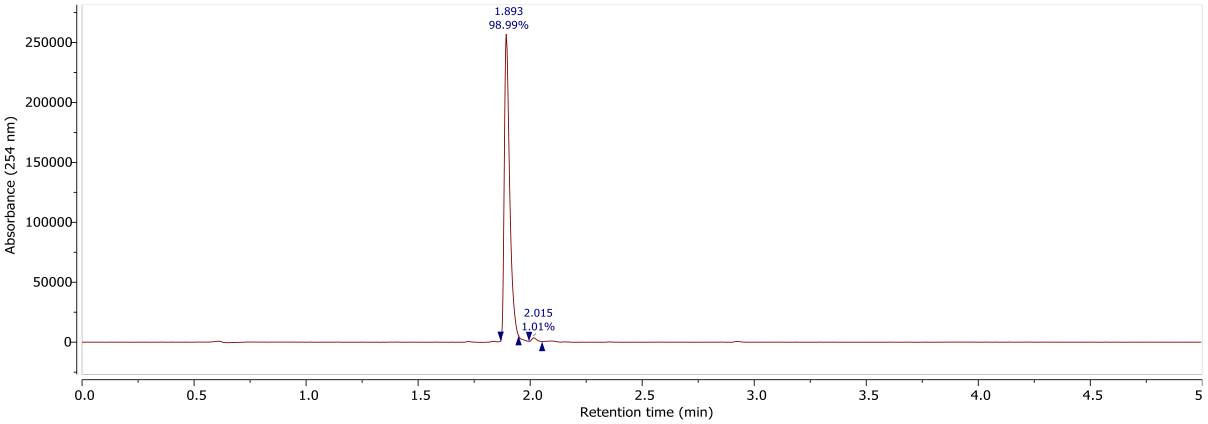

	RT	Height	Area	Total Height %	Total Area %	Start time	End time
1	1.893	254715.250	4207768.000	98.85	98.99	1.868	1.948
2	2.015	2975.057	43106.000	1.15	1.01	1.995	2.053

Compound 23. *LC-MS*

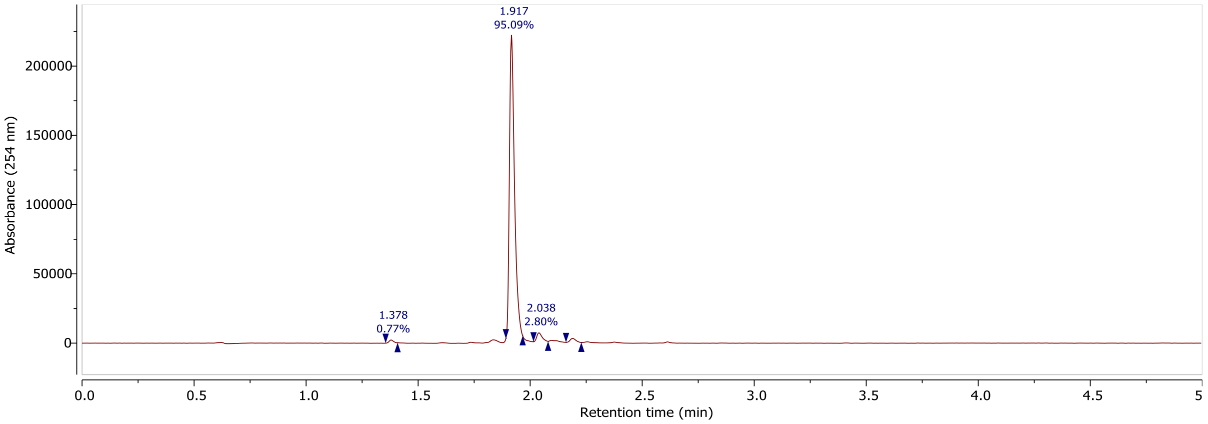

	RT	Height	Area	Total Height %	Total Area %	Start time	End time
1	1.378	2169.000	28104.000	0.94	0.77	1.355	1.408
2	1.917	218535.000	3448359.000	95.07	95.09	1.892	1.967
3	2.038	6308.949	101417.000	2.74	2.80	2.015	2.080
4	2.190	2849.049	48462.000	1.24	1.34	2.160	2.228

Compound 24. *LC-MS*

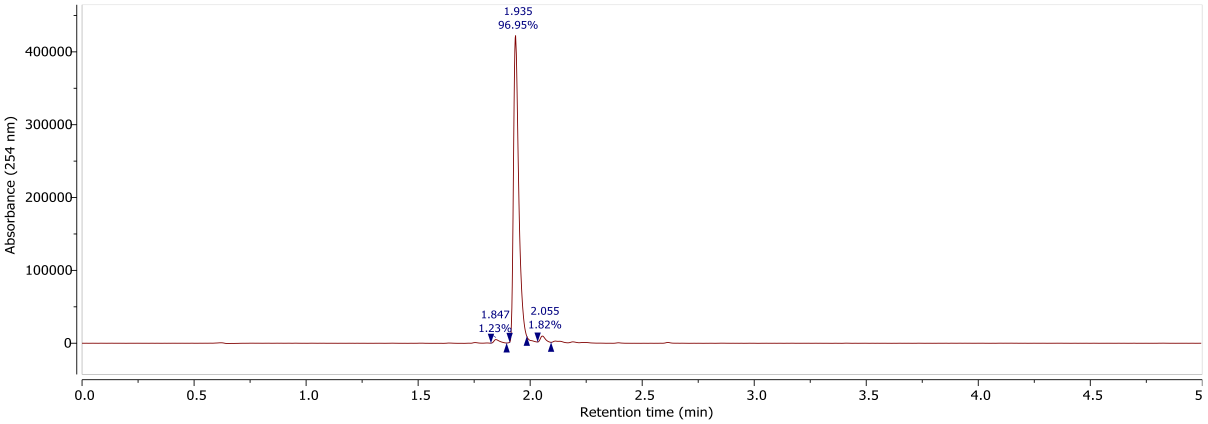

	RT	Height	Area	Total Height %	Total Area %	Start time	End time
1	1.847	4932.167	85067.500	1.14	1.23	1.825	1.895
2	1.935	418483.957	6692944.000	96.89	96.95	1.908	1.985
3	2.055	8505.722	125837.000	1.97	1.82	2.033	2.093

Compound 25. *LC-MS*

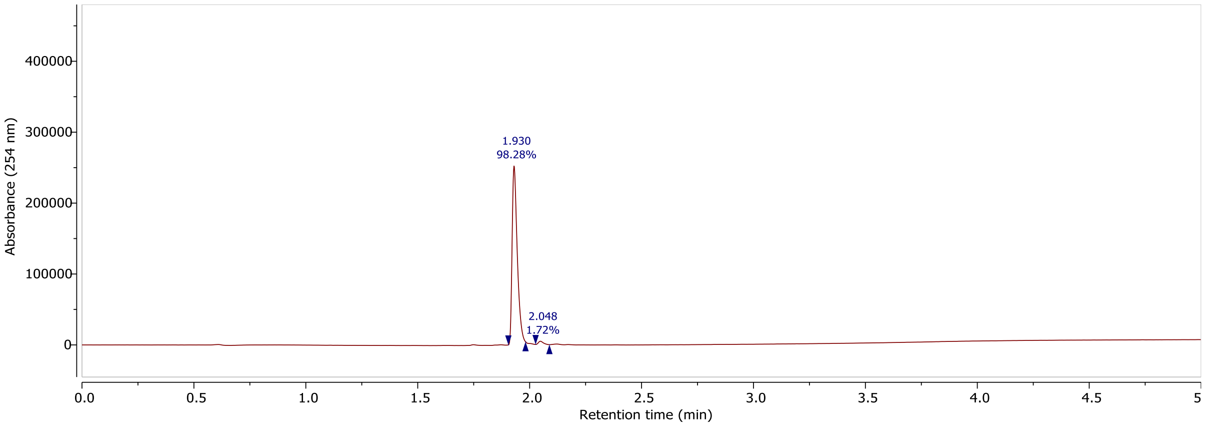

	RT	Area	Height	Total Height %	Total Area %	Start time	End time
1	1.930	3999405.500	251111.848	98.18	98.28	1.905	1.982
2	2.048	69957.000	4665.054	1.82	1.72	2.027	2.088

Compound 26. *LC-MS*

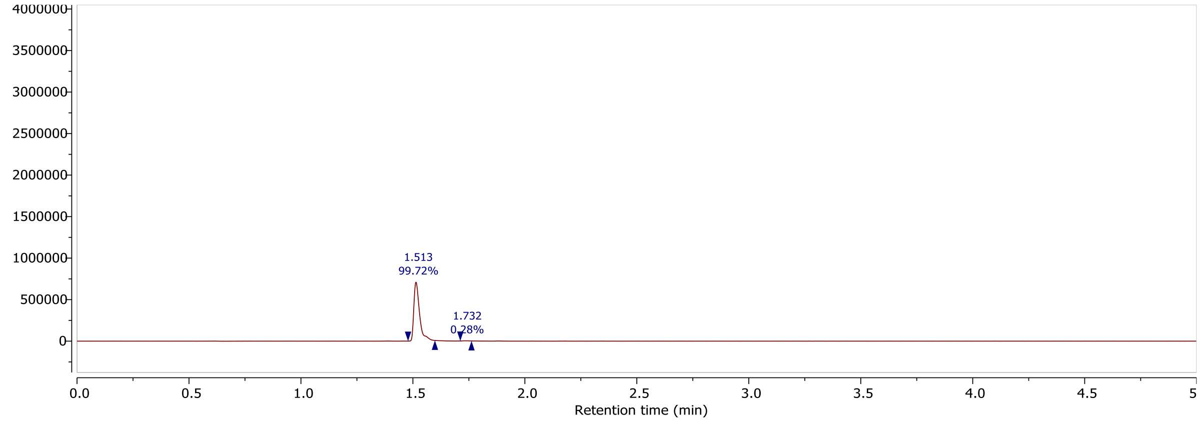

	RT	Area	Height	Total Height %	Total Area %	Start time	End time
1	1.513	12436857.278	708716.083	99.71	99.72	1.478	1.598
2	1.732	35000.000	2036.600	0.29	0.28	1.712	1.762

Compound 27. *LC-MS*

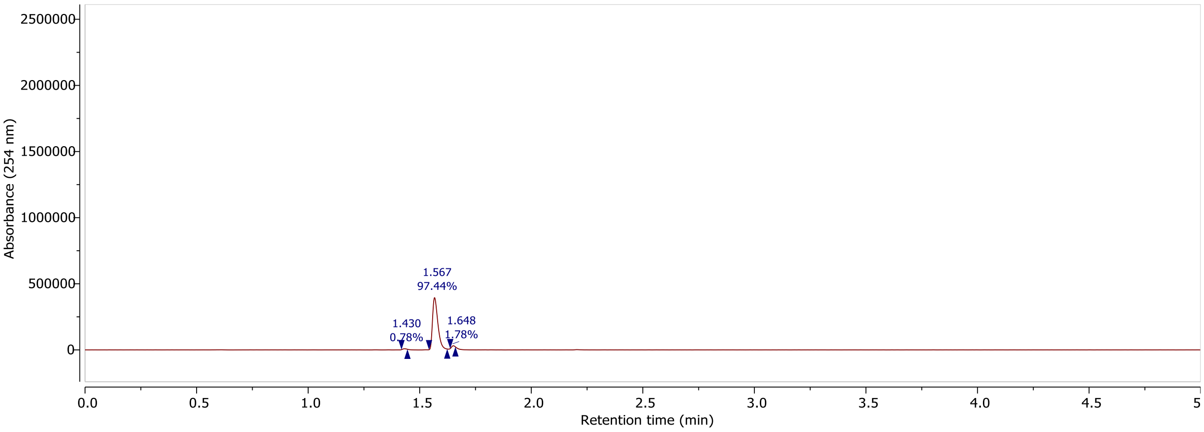

	RT	Area	Height	Total Height %	Total Area %	Start time	End time
1	1.430	51636.000	5368.250	1.30	0.78	1.418	1.445
2	1.567	6432120.000	393004.878	95.25	97.44	1.542	1.623
3	1.648	117225.000	14217.000	3.45	1.78	1.637	1.660

Compound 28. *LC-MS*

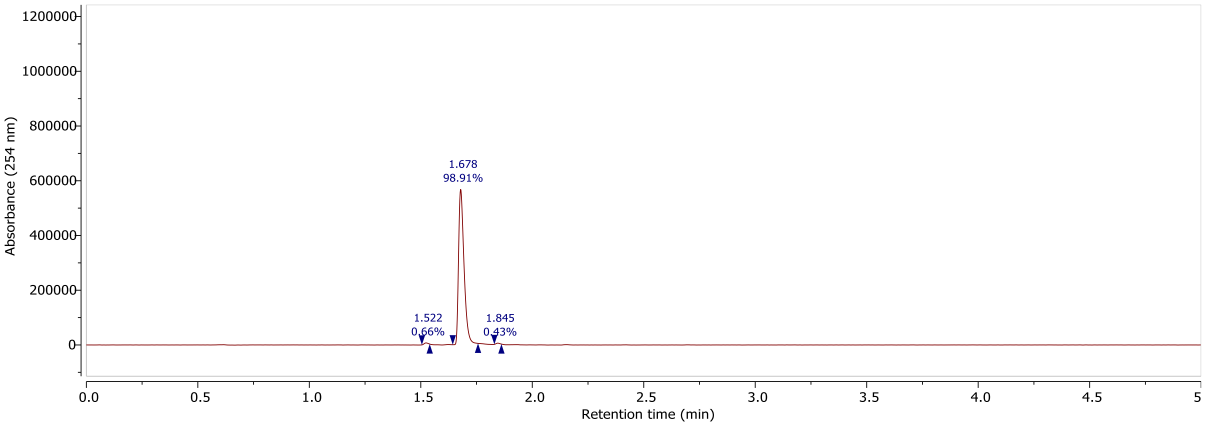

	RT	Area	Height	Total Height %	Total Area %	Start time	End time
1	1.522	63937.000	5639.333	0.98	0.66	1.505	1.540
2	1.678	9566843.147	566541.912	98.33	98.91	1.643	1.757
3	1.845	41645.000	3992.789	0.69	0.43	1.830	1.862

Compound 29. *LC-MS*

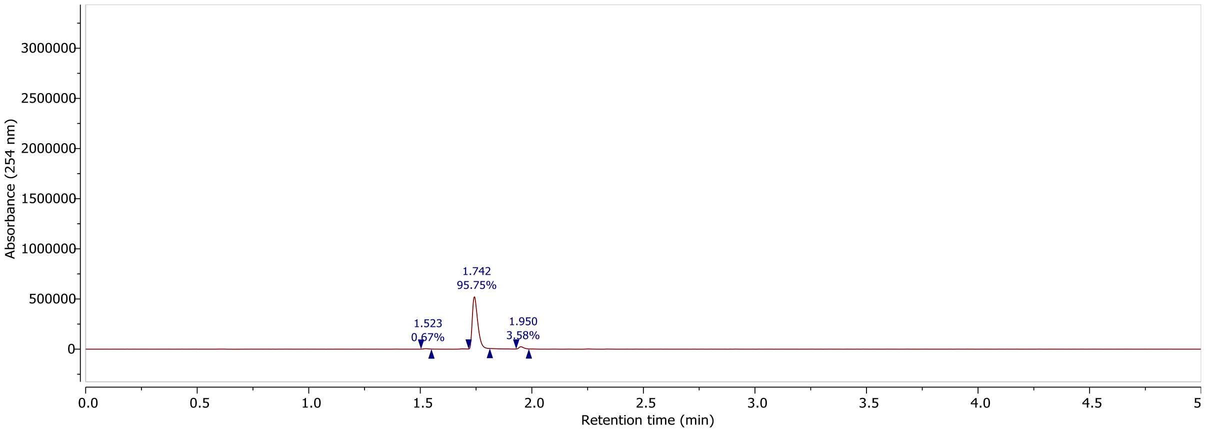

	RT	Area	Height	Total Height %	Total Area %	Start time	End time
1	1.523	61837.000	4436.571	0.81	0.67	1.503	1.550
2	1.742	8812257.000	522112.895	95.06	95.75	1.717	1.812
3	1.950	329218.500	22704.529	4.13	3.58	1.930	1.987

Compound 30. *LC-MS*

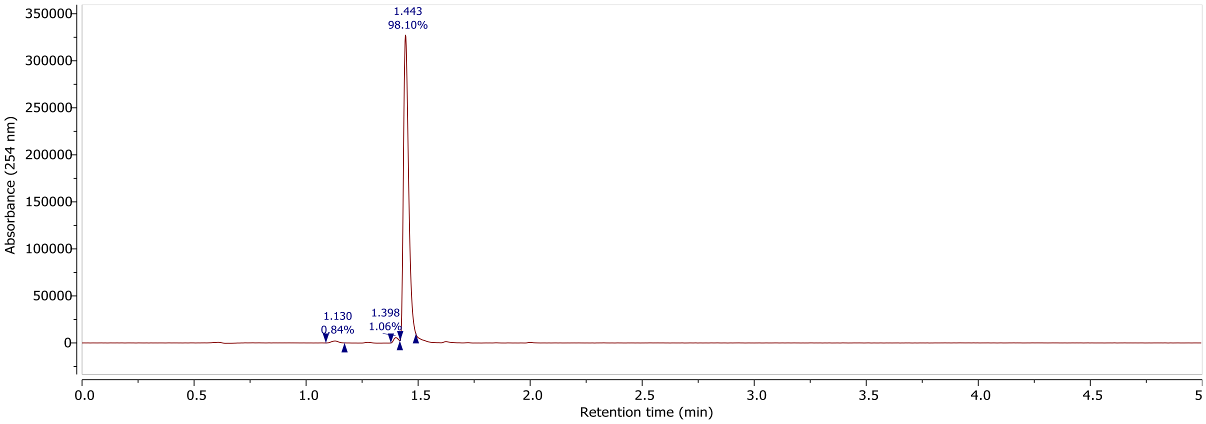

	RT	Height	Area	Total Height %	Total Area %	Start time	End time
1	1.130	2049.000	43406.000	0.62	0.84	1.088	1.172
2	1.398	4419.000	55222.000	1.34	1.06	1.378	1.418
3	1.443	322633.000	5093521.500	98.03	98.10	1.420	1.490

Compound 31. *LC-MS*

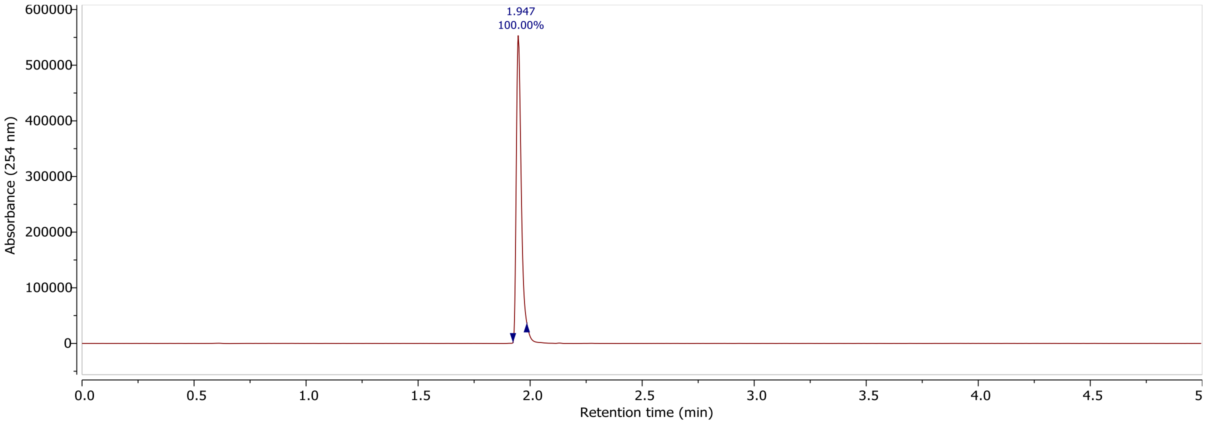

	RT	Height	Area	Total Height %	Total Area %	Start time	End time
1	1.947	538594.108	7823343.000	100.00	100.00	1.923	1.985

Compound 32. *LC-MS*

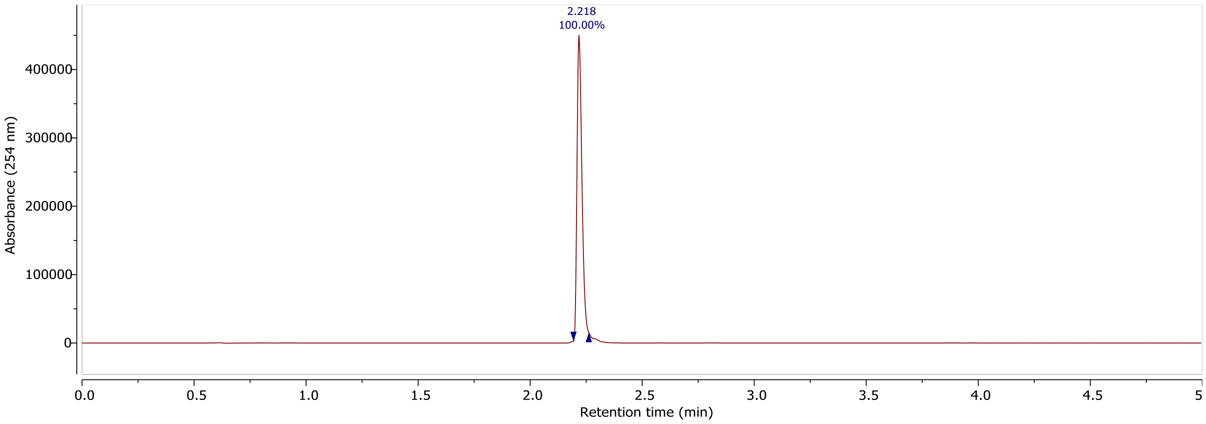

	RT	Height	Area	Total Height %	Total Area %	Start time	End time
1	2.218	442947.390	6688056.000	100.00	100.00	2.193	2.262

Compound 33. *LC-MS*

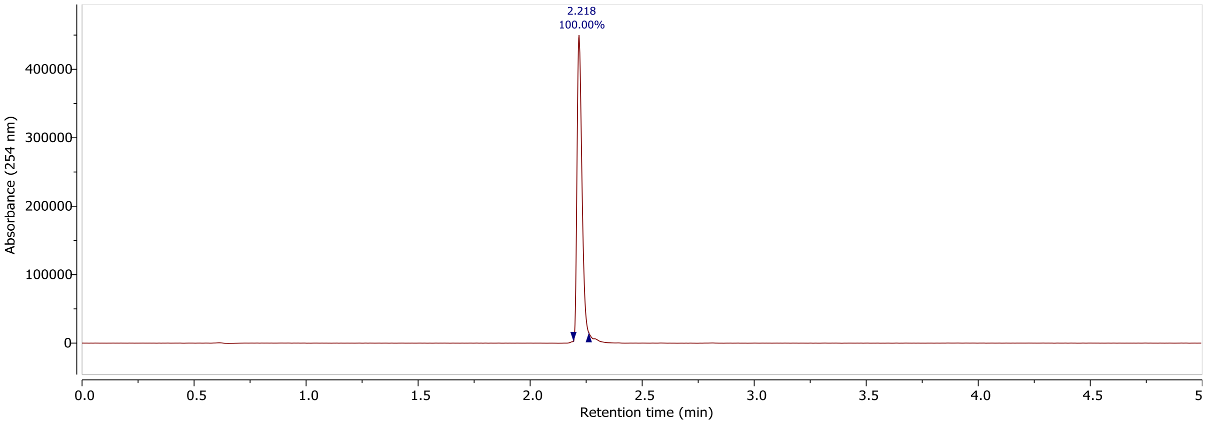

	RT	Height	Area	Total Height %	Total Area %	Start time	End time
1	1.790	2190101.478	38042511.000	100.00	100.00	1.762	1.838

Compound 34. *LC-MS*

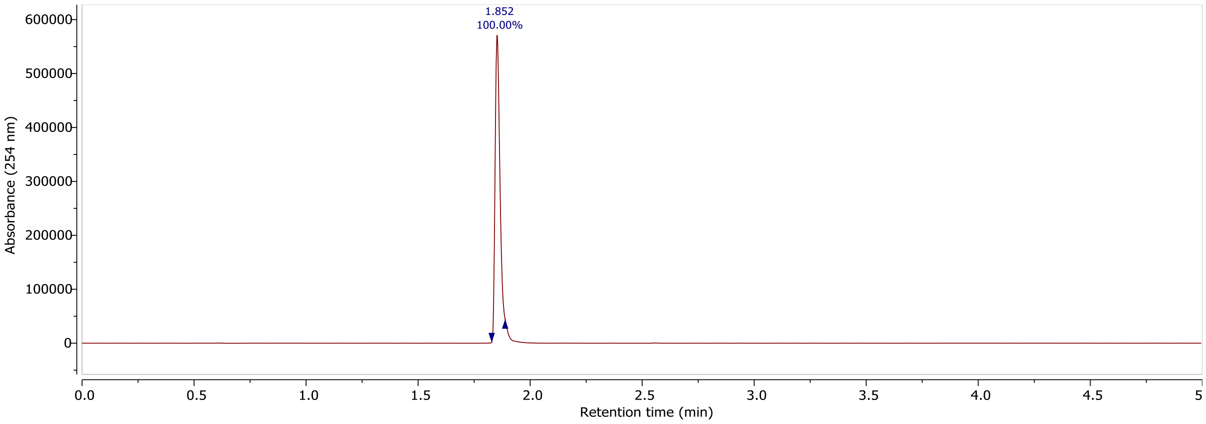

	RT	Height	Area	Total Height %	Total Area %	Start time	End time
1	1.852	552979.889	7961389.000	100.00	100.00	1.828	1.888

Compound 35. *LC-MS*

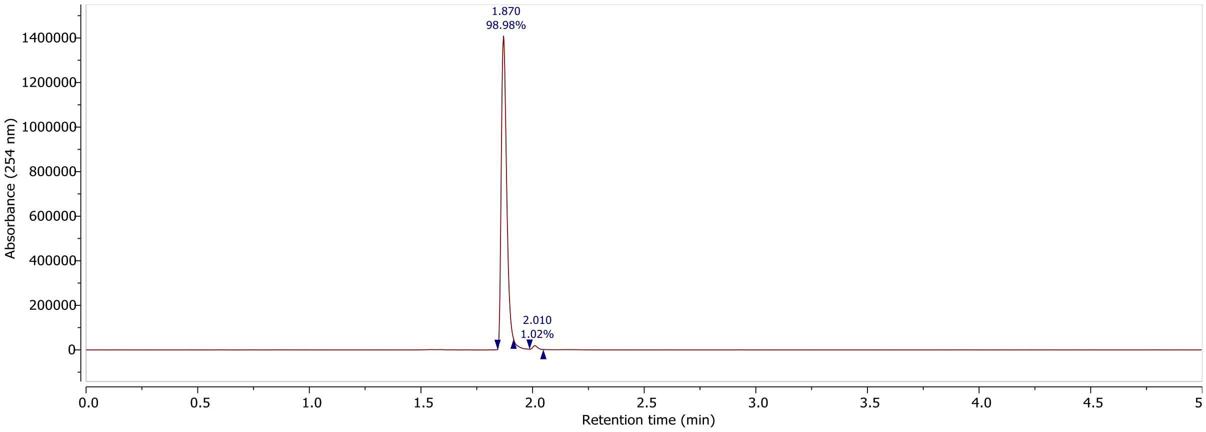

	RT	Height	Area	Total Height %	Total Area %	Start time	End time
1	1.870	1390290.372	22825342.000	98.80	98.98	1.843	1.915
2	2.010	16953.649	234796.000	1.20	1.02	1.987	2.048

Compound 36. *LC-MS*

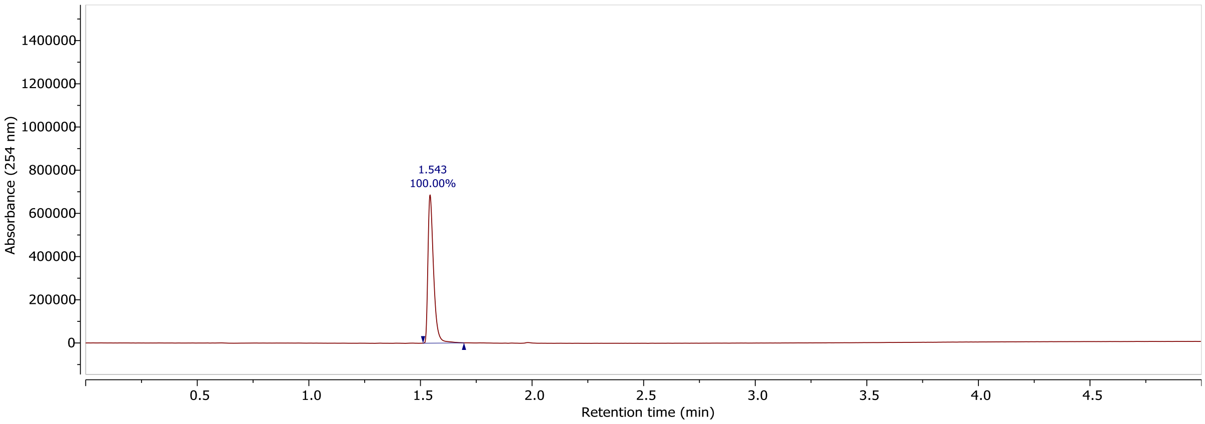

	RT	Height	Area	Total Height %	Total Area %	Start time	End time
1	1.543	687299.400	12058395.000	100.00	100.00	1.512	1.695

Compound 37. *LC-MS*

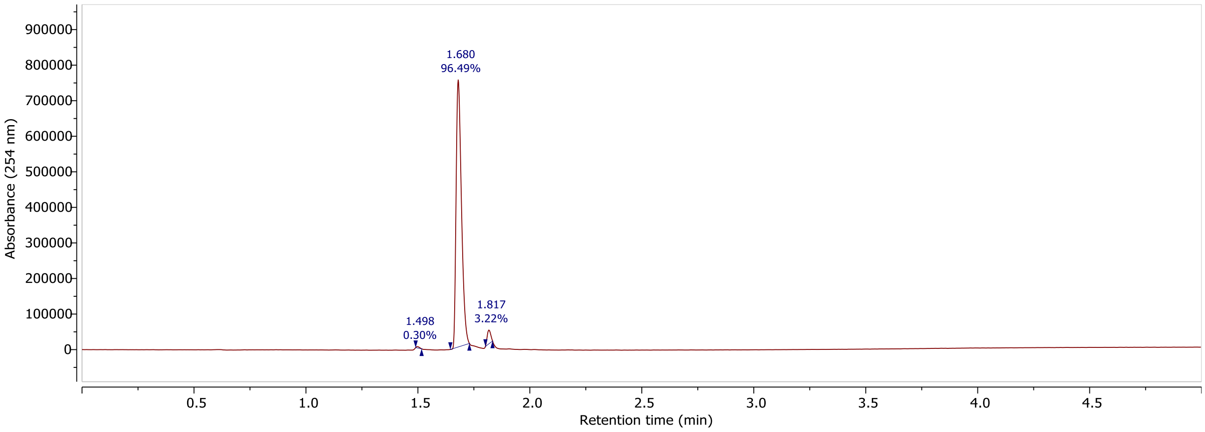

	RT	Height	Area	Total Height %	Total Area %	Start time	End time
1	1.498	4055.813	38393.500	0.51	0.30	1.490	1.517
2	1.680	751688.176	12508982.706	94.56	96.49	1.645	1.730
3	1.817	39181.000	416989.000	4.93	3.22	1.802	1.833

Compound 38. *LC-MS*

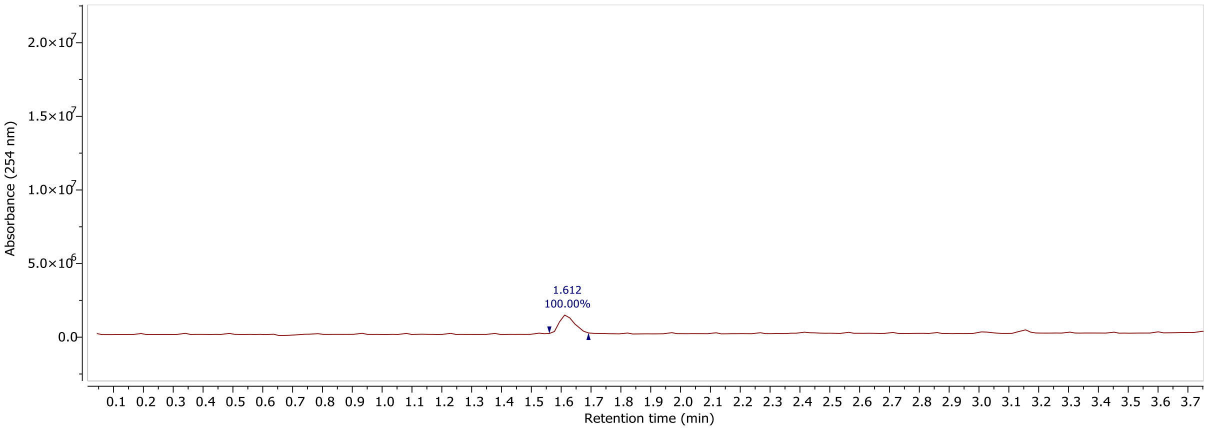

	RT	Area	Height	Total Height %	Total Area %	Start time	End time
1	1.612	3944788.469	1236774.567	100.00	100.00	1.560	1.692

Compound 39. *LC-MS*

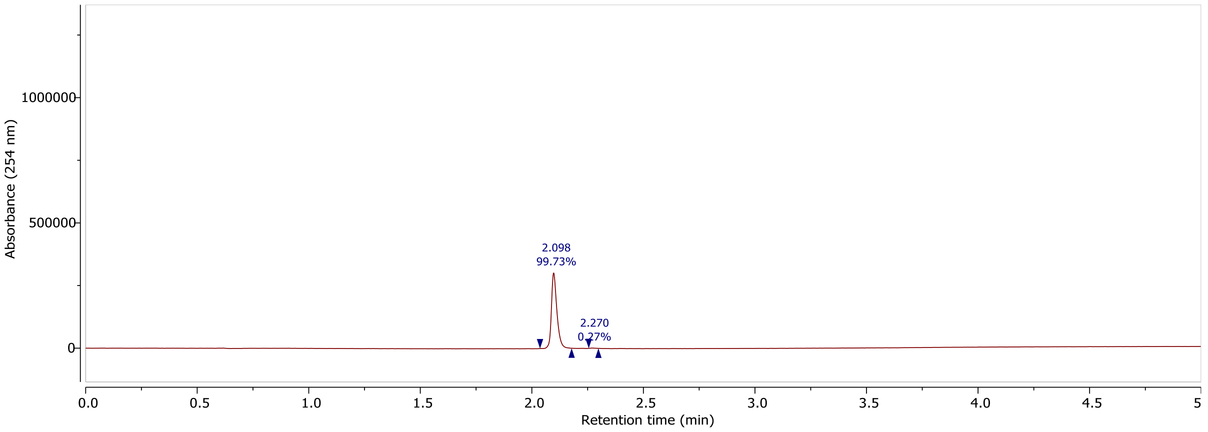

	RT	Area	Height	Total Height %	Total Area %	Start time	End time
1	2.098	5145246.000	301536.612	99.65	99.73	2.037	2.178
2	2.270	13949.500	1046.115	0.35	0.27	2.255	2.298

Compound 40. *LC-MS*

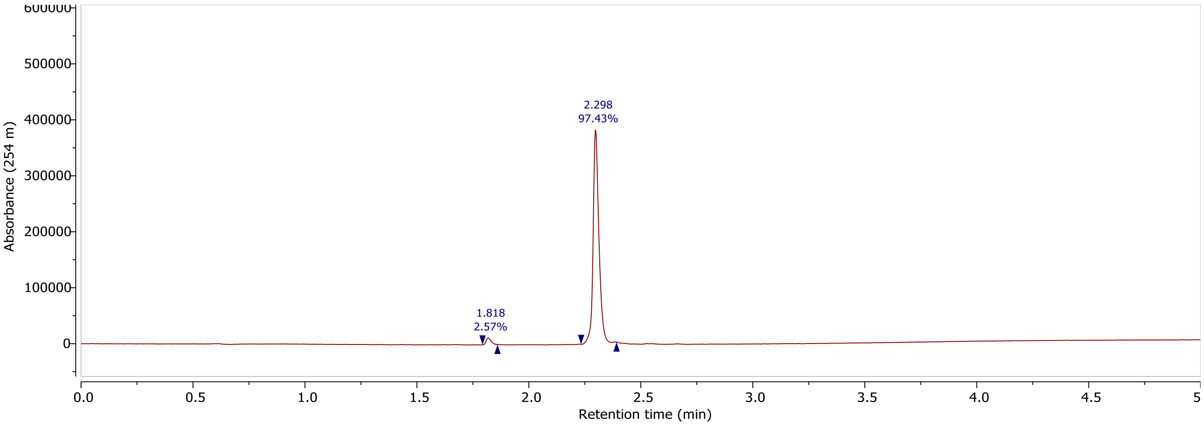

	RT	Area	Height	Total Height %	Total Area %	Start time	End time
1	1.818	179756.500	12078.125	3.07	2.57	1.793	1.860
2	2.298	6824302.500	381626.000	96.93	97.43	2.233	2.392

Compound 41. *LC-MS*

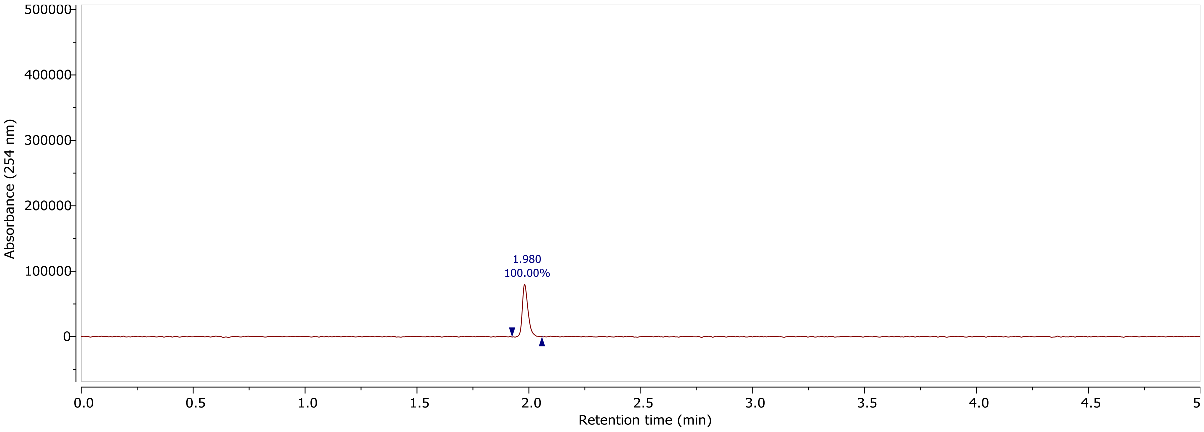

	RT	Area	Height	Total Height %	Total Area %	Start time	End time
1	1.980	1425347.532	80230.888	100.00	100.00	1.925	2.058

Compound 42. *LC-MS*

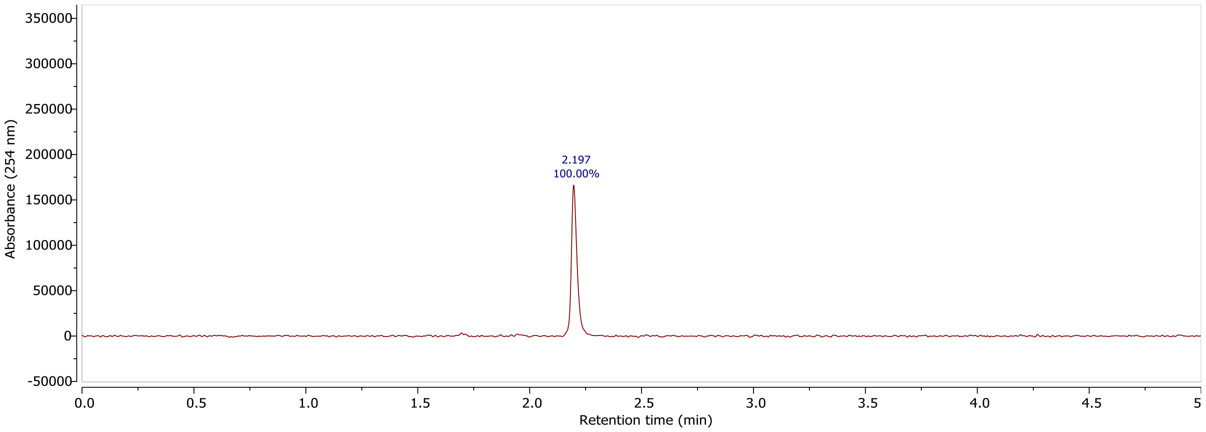

	RT	Area	Height	Total Height %	Total Area %	Start time	End time
1	2.197	2946255.681	166012.759	100.00	100.00	2.135	2.273

Compound 43. *LC-MS*

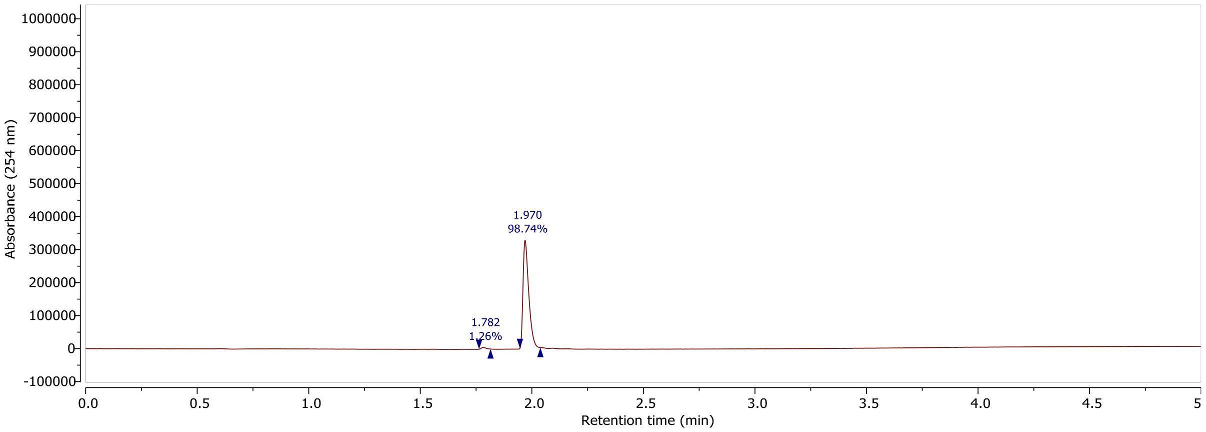

	RT	Area	Height	Total Height %	Total Area %	Start time	End time
1	1.782	74397.000	5041.742	1.52	1.26	1.763	1.815
2	1.970	5824443.000	327549.109	98.48	98.74	1.947	2.038

Compound 44. *LC-MS*

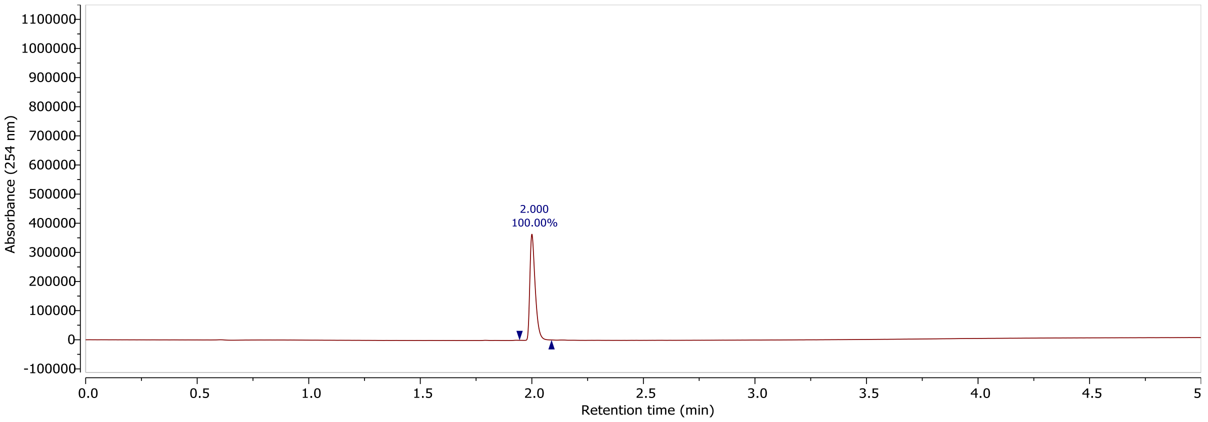

	RT	Area	Height	Total Height %	Total Area %	Start time	End time
1	2.000	6028281.465	364472.558	100.00	100.00	1.945	2.088

Compound 45. *LC-MS*

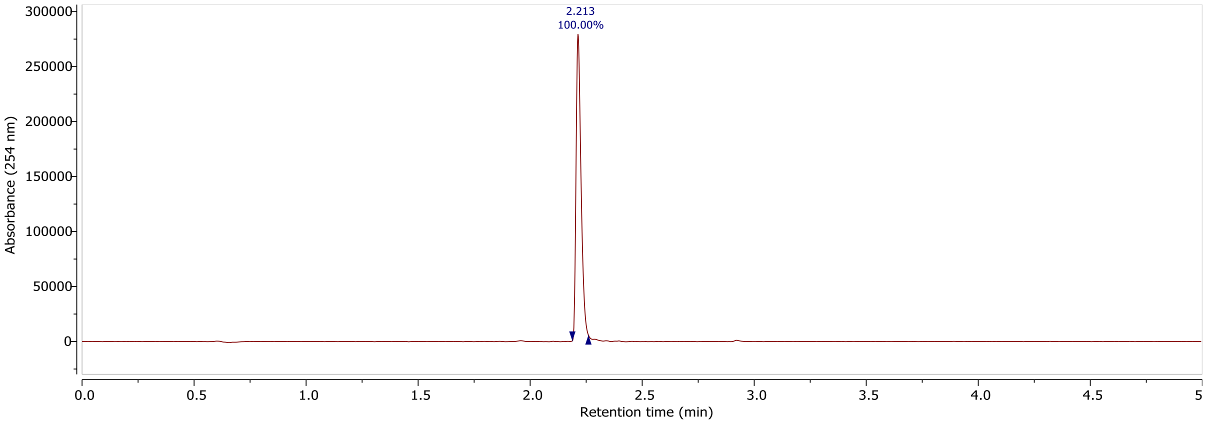

	RT	Height	Area	Total Height %	Total Area %	Start time	End time
1	2.213	277264.907	4273263.000	100.00	100.00	2.188	2.260

Compound 46. *LC-MS*

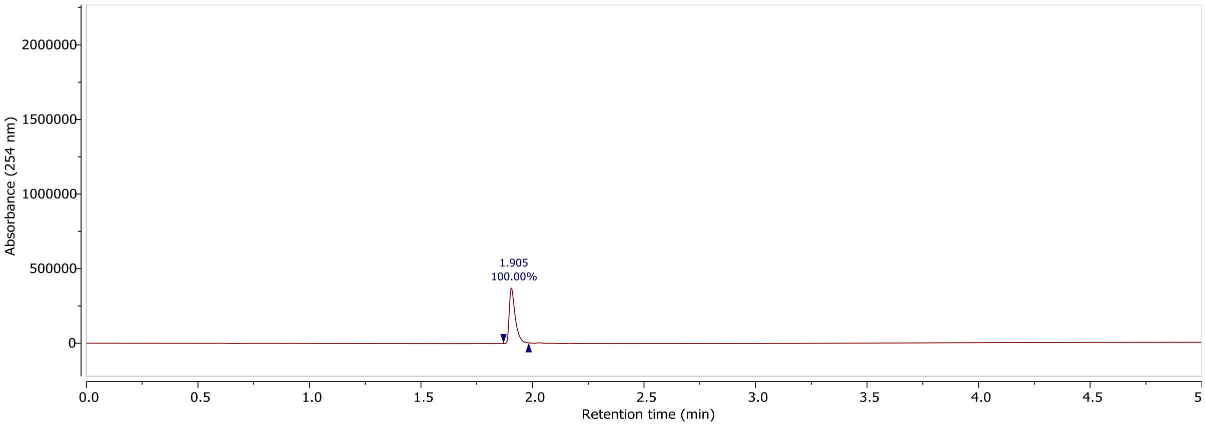

	RT	Area	Height	Total Height %	Total Area %	Start time	End time
1	1.905	6816251.294	371739.353	100.00	100.00	1.870	1.983

Compound 47. *LC-MS*

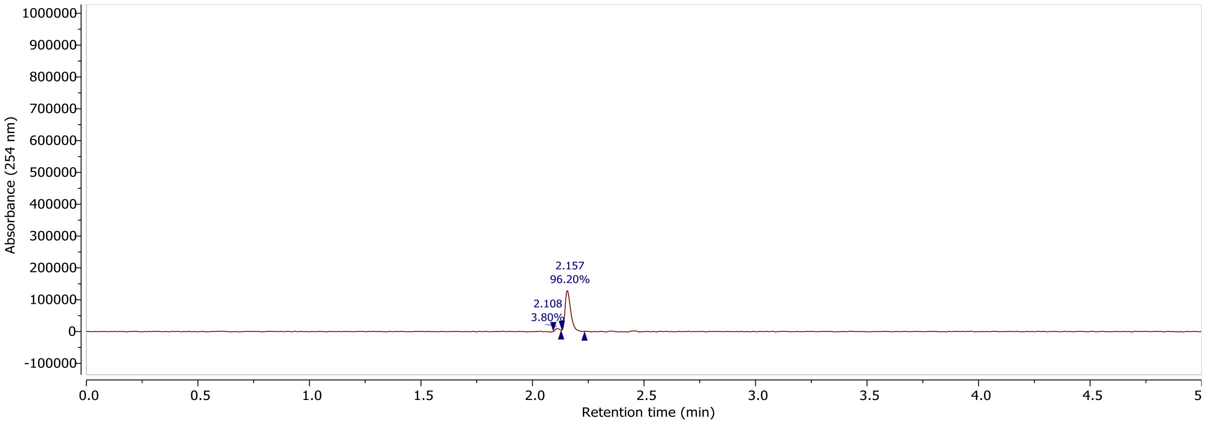

	RT	Area	Height	Total Height %	Total Area %	Start time	End time
1	2.108	83816.000	7376.286	5.55	3.80	2.093	2.128
2	2.157	2123643.984	125582.607	94.45	96.20	2.132	2.233

References

(1) Crowe, C.; Nakasone, M. A.; Chandler, S.; Craigon, C.; Sathe, G.; Tatham, M. H.; Makukhin, N.; Hay, R. T.; Ciulli, A. Mechanism of Degrader-Targeted Protein Ubiquitinability. *Sci. Adv.* **2024**, *10* (41). https://doi.org/10.1126/sciadv.ado6492.

(2) Palaferri, L.; Cheng-Sánchez, I.; Gosselé, K.; Zielinska, D.; Nevado, C. Discovery of First-in-Class BAZ2A/B and BAZ2B-Selective Degraders. *ACS Med. Chem. Lett.* **2025**, *16* (2), 234–240. https://doi.org/10.1021/acsmedchemlett.4c00334.

(3) Buckley, D. L.; Van Molle, I.; Gareiss, P. C.; Tae, H. S.; Michel, J.; Noblin, D. J.; Jorgensen, W. L.; Ciulli, A.; Crews, C. M. Targeting the von Hippel-Lindau E3 Ubiquitin Ligase Using Small Molecules to Disrupt the VHL/HIF-1α Interaction. *J. Am. Chem. Soc.* **2012**, *134* (10). https://doi.org/10.1021/ja209924v.

(4) Tallant, C.; Valentini, E.; Fedorov, O.; Overvoorde, L.; Ferguson, F. M.; Filippakopoulos, P.; Svergun, D. I.; Knapp, S.; Ciulli, A. Molecular Basis of Histone Tail Recognition by Human TIP5 PHD Finger and Bromodomain of the Chromatin Remodeling Complex NoRC. *Structure* **2015**, *23* (1), 80–92. https://doi.org/https://doi.org/10.1016/j.str.2014.10.017.

(5) Winter, G. *Xia2* : An Expert System for Macromolecular Crystallography Data Reduction. *J. Appl. Crystallogr.* **2010**, *43* (1), 186–190. https://doi.org/10.1107/S0021889809045701.

(6) Winter, G.; Waterman, D. G.; Parkhurst, J. M.; Brewster, A. S.; Gildea, R. J.; Gerstel, M.; Fuentes-Montero, L.; Vollmar, M.; Michels-Clark, T.; Young, I. D.; Sauter, N. K.; Evans, G. *DIALS* : Implementation and Evaluation of a New Integration Package. *Acta Crystallogr. D Struct. Biol.* **2018**, *74* (2), 85–97. https://doi.org/10.1107/S2059798317017235.

(7) Beilsten-Edmands, J.; Winter, G.; Gildea, R.; Parkhurst, J.; Waterman, D.; Evans, G. Scaling Diffraction Data in the *DIALS* Software Package: Algorithms and New Approaches for Multi-Crystal Scaling. *Acta Crystallogr. D Struct. Biol.* **2020**, *76* (4), 385–399. https://doi.org/10.1107/S2059798320003198.

(8) Winn, M. D.; Ballard, C. C.; Cowtan, K. D.; Dodson, E. J.; Emsley, P.; Evans, P. R.; Keegan, R. M.; Krissinel, E. B.; Leslie, A. G. W.; McCoy, A.; McNicholas, S. J.; Murshudov, G. N.; Pannu, N. S.; Potterton, E. A.; Powell, H. R.; Read, R. J.; Vagin, A.; Wilson, K. S. Overview of the *CCP* 4 Suite and Current Developments. *Acta Crystallogr. D Biol. Crystallogr.* **2011**, *67* (4), 235–242. https://doi.org/10.1107/S0907444910045749.

(9) Evans, P. R.; Murshudov, G. N. How Good Are My Data and What Is the Resolution? *Acta Crystallogr. D Biol. Crystallogr.* **2013**, *69* (7), 1204–1214. https://doi.org/10.1107/S0907444913000061.

(10) McCoy, A. J.; Grosse-Kunstleve, R. W.; Adams, P. D.; Winn, M. D.; Storoni, L. C.; Read, R. J. *Phaser* Crystallographic Software. *J. Appl. Crystallogr.* **2007**, *40* (4), 658–674. https://doi.org/10.1107/S0021889807021206.

(11) Drouin, L.; McGrath, S.; Vidler, L. R.; Chaikuad, A.; Monteiro, O.; Tallant, C.; Philpott, M.; Rogers, C.; Fedorov, O.; Liu, M.; Akhtar, W.; Hayes, A.; Raynaud, F.; Müller, S.; Knapp, S.; Hoelder, S. Structure Enabled Design of BaZ2-ICR, a Chemical Probe Targeting the

Bromodomains of BAZ2A and BAZ2B. *J. Med. Chem.* **2015**, *58* (5), 2553–2559. https://doi.org/10.1021/jm501963e.

(12) Scott, T.; Smethurst, C. A. P.; Westermaier, Y.; Mayer, M.; Greb, P.; Kousek, R.; Biberger, T.; Bader, G.; Jandova, Z.; Schmalhorst, P. S.; Fuchs, J. E.; Magarkar, A.; Hoenke, C.; Gerstberger, T.; Combs, S. A.; Pape, R.; Phul, S.; Kothiwale, S.; Bergner, A.; Alekseevich, A. D.; Barmettler, W.; Bogusz, K.; Boykov, A.; Cai, A.; Canfield, J. M.; Chen, A. M.; Clayton, K.; Coral, A. R.; Dickel, M.; Feldmann, H.; Gao, L.; George, T. J.; Gildea, K. M.; Hansen, G. T.; Higgins, A. J.; Innes, R. B. M.; Kestemont, B.; Lubow, A.; McIlvaine, G. V.; McNaughton, I. J.; Partinico, C.; Pit, V.; Sen, A.; Simon, M.; Simon, V.; Spearing, R.; Tastan, B.; Vincent, S.; Wei, L.; Weinberg, J.; Wells, K. D.; Wheeler, D. C.; Yeginer, U.; Zaccanelli, S. J.; Zambrano-Bukonja, C.; Waterson, A. G.; Weinstabl, H.; McConnell, D. B.; Meiler, J.; Böttcher, J.; Moretti, R. Drugit: Crowd-Sourcing Molecular Design of Non-Peptidic VHL Binders. *Nat. Commun.* **2025**, *16* (1), 3548. https://doi.org/10.1038/s41467-025-58406-0.

(13) Moriarty, N. W.; Grosse-Kunstleve, R. W.; Adams, P. D. *Electronic Ligand Builder and Optimization Workbench* (*ELBOW*): A Tool for Ligand Coordinate and Restraint Generation. *Acta Crystallogr. D Biol. Crystallogr.* **2009**, *65* (10), 1074–1080. https://doi.org/10.1107/S0907444909029436.

(14) Murshudov, G. N.; Skubák, P.; Lebedev, A. A.; Pannu, N. S.; Steiner, R. A.; Nicholls, R. A.; Winn, M. D.; Long, F.; Vagin, A. A. *REFMAC* 5 for the Refinement of Macromolecular Crystal Structures. *Acta Crystallogr. D Biol. Crystallogr.* **2011**, *67* (4), 355–367. https://doi.org/10.1107/S0907444911001314.

(15) Emsley, P.; Cowtan, K. *Coot* : Model-Building Tools for Molecular Graphics. *Acta Crystallogr. D Biol. Crystallogr.* **2004**, *60* (12), 2126–2132. https://doi.org/10.1107/S0907444904019158.

(16) Malý, M.; Diederichs, K.; Dohnálek, J.; Kolenko, P. Paired Refinement under the Control of *PAIREF*. *IUCrJ* **2020**, *7* (4), 681–692. https://doi.org/10.1107/S2052252520005916.

(17) Gu, L.; Frommel, S. C.; Oakes, C. C.; Simon, R.; Grupp, K.; Gerig, C. Y.; Bär, D.; Robinson, M. D.; Baer, C.; Weiss, M.; Gu, Z.; Schapira, M.; Kuner, R.; Sültmann, H.; Provenzano, M.; Yaspo, M. L.; Brors, B.; Korbel, J.; Schlomm, T.; Sauter, G.; Eils, R.; Plass, C.; Santoro, R. BAZ2A (TIP5) Is Involved in Epigenetic Alterations in Prostate Cancer and Its Overexpression Predicts Disease Recurrence. *Nat. Genet.* **2015**, *47* (1), 22–30. https://doi.org/10.1038/ng.3165.

(18) Peña-Hernández, R.; Aprigliano, R.; Carina Frommel, S.; Pietrzak, K.; Steiger, S.; Roganowicz, M.; Lerra, L.; Bizzarro, J.; Santoro, R. BAZ2A-mediated Repression via H3K14ac-marked Enhancers Promotes Prostate Cancer Stem Cells. *EMBO Rep.* **2021**, *22* (11), e53014. https://doi.org/10.15252/embr.202153014.

(19) Hughes, C. S.; Foehr, S.; Garfield, D. A.; Furlong, E. E.; Steinmetz, L. M.; Krijgsveld, J. Ultrasensitive Proteome Analysis Using Paramagnetic Bead Technology. *Mol. Syst. Biol.* **2014**, *10* (10), 757. https://doi.org/10.15252/msb.20145625.

(20) Leutert, M.; Rodríguez-Mias, R. A.; Fukuda, N. K.; Villén, J. R2-P2 Rapid-robotic Phosphoproteomics Enables Multidimensional Cell Signaling Studies. *Mol. Syst. Biol.* **2019**, *15* (12), e9021. https://doi.org/10.15252/msb.20199021.

(21) Panse, C.; Trachsel, C.; Türker, C. Bridging Data Management Platforms and Visualization Tools to Enable Ad-Hoc and Smart Analytics in Life Sciences. *J. Integr. Bioinform.* **2022**, *19* (4), 20220031. https://doi.org/10.1515/jib-2022-0031.

(22) Djomehri, S. I.; Gonzalez, M. E.; da Veiga Leprevost, F.; Tekula, S. R.; Chang, H.-Y.; White, M. J.; Cimino-Mathews, A.; Burman, B.; Basrur, V.; Argani, P.; Nesvizhskii, A. I.; Kleer, C. G. Quantitative Proteomic Landscape of Metaplastic Breast Carcinoma Pathological Subtypes and Their Relationship to Triple-Negative Tumors. *Nat. Commun.* **2020**, *11* (1), 1723. https://doi.org/10.1038/s41467-020-15283-z.

(23) Wolski, W. E.; Nanni, P.; Grossmann, J.; d'Errico, M.; Schlapbach, R.; Panse, C. *Prolfqua* : A Comprehensive *R* -Package for Proteomics Differential Expression Analysis. *J. Proteome Res.* **2023**, *22* (4), 1092–1104. https://doi.org/10.1021/acs.jproteome.2c00441.

(24) Huber, W.; von Heydebreck, A.; Sültmann, H.; Poustka, A.; Vingron, M. Variance Stabilization Applied to Microarray Data Calibration and to the Quantification of Differential Expression. *Bioinformatics* **2002**, *18* (suppl_1), S96–S104. https://doi.org/10.1093/bioinformatics/18.suppl_1.S96.

(25) Smyth, G. K. Linear Models and Empirical Bayes Methods for Assessing Differential Expression in Microarray Experiments. *Stat. Appl. Genet. Mol. Biol.* **2004**, *3* (1), 1–25. https://doi.org/10.2202/1544-6115.1027.

(26) Benjamini, Y.; Hochberg, Y. Controlling the False Discovery Rate: A Practical and Powerful Approach to Multiple Testing. *Journal of the Royal Statistical Society. Series B (Methodological)* **1995**, *57* (1), 289–300.

(27) Bertomeu, T.; Coulombe-Huntington, J.; Chatr-aryamontri, A.; Bourdages, K. G.; Coyaud, E.; Raught, B.; Xia, Y.; Tyers, M. A High-Resolution Genome-Wide CRISPR/Cas9 Viability Screen Reveals Structural Features and Contextual Diversity of the Human Cell-Essential Proteome. *Mol. Cell. Biol.* **2018**, *38* (1), e00302-17. https://doi.org/10.1128/MCB.00302-17.

(28) Zoppi, V.; Hughes, S. J.; Maniaci, C.; Testa, A.; Gmaschitz, T.; Wieshofer, C.; Koegl, M.; Riching, K. M.; Daniels, D. L.; Spallarossa, A.; Ciulli, A. Iterative Design and Optimization of Initially Inactive Proteolysis Targeting Chimeras (PROTACs) Identify VZ185 as a Potent, Fast, and Selective von Hippel–Lindau (VHL) Based Dual Degrader Probe of BRD9 and BRD7. *J. Med. Chem.* **2019**, *62* (2), 699–726. https://doi.org/10.1021/acs.jmedchem.8b01413.

(29) Liu, X.; Kalogeropulou, A. F.; Domingos, S.; Makukhin, N.; Nirujogi, R. S.; Singh, F.; Shpiro, N.; Saalfrank, A.; Sammler, E.; Ganley, I. G.; Moreira, R.; Alessi, D. R.; Ciulli, A. Discovery of XL01126: A Potent, Fast, Cooperative, Selective, Orally Bioavailable, and Blood–Brain Barrier Penetrant PROTAC Degrader of Leucine-Rich Repeat Kinase 2. *J. Am. Chem. Soc.* **2022**, *144* (37), 16930–16952. https://doi.org/10.1021/jacs.2c05499.

(30) Cheng, J.; Li, Y.; Wang, X.; Dong, G.; Sheng, C. Discovery of Novel PDEδ Degraders for the Treatment of KRAS Mutant Colorectal Cancer. *J. Med. Chem.* **2020**, *63* (14), 7892–7905. https://doi.org/10.1021/acs.jmedchem.0c00929.

(31) Fan, J.; Zeng, F.; Wu, S.; Wang, X. Polymer Micelle with PH-Triggered Hydrophobic–Hydrophilic Transition and De-Cross-Linking Process in the Core and Its Application for Targeted Anticancer Drug Delivery. *Biomacromolecules* **2012**, *13* (12), 4126–4137. https://doi.org/10.1021/bm301424r.

(32) Chen, X.; Wang, L.; Li, C.; Xiao, J.; Ding, H.; Liu, X.; Zhang, X.; He, W.; Yang, H. Light-Controllable Reflection Wavelength of Blue Phase Liquid Crystals Doped with Azobenzene-Dimers. *Chem. Comm.* **2013**, *49* (86), 10097. https://doi.org/10.1039/c3cc46117c.

(33) Luscombe, C. K.; Proemmel, S.; Huck, W. T. S.; Holmes, A. B.; Fukushima, H. Synthesis of Supercritical Carbon Dioxide Soluble Perfluorinated Dendrons for Surface Modification. *J. Org. Chem.* **2007**, *72* (15), 5505–5513. https://doi.org/10.1021/jo070293f.

(34) Lee, E. J.; Duggirala, K. B.; Lee, Y.; Yun, M. R.; Jang, J.; Cyriac, R.; Jung, M. E.; Choi, G.; Chae, C. H.; Cho, B. C.; Lee, K. Novel Allosteric Glutaminase 1 Inhibitors with Macrocyclic Structure Activity Relationship Analysis. *Bioorg. Med. Chem. Lett.* **2022**, *75*, 128956. https://doi.org/10.1016/j.bmcl.2022.128956.

(35) Wang, T.; Wang, J.; Chen, L.; Zhang, X.; Mou, T.; An, X.; Zhang, J.; Zhang, X.; Deuther-Conrad, W.; Huang, Y.; Jia, H. Development of a Highly Specific ^{18}F-Labeled Radioligand for Imaging of the Sigma-2 Receptor in Brain Tumors. *J. Med. Chem.* **2023**, *66* (18), 12840–12857. https://doi.org/10.1021/acs.jmedchem.3c00735.

(36) Nguyen, T. B.; Castanet, A.-S.; Nguyen, T.-H.; Nguyen, K. P. P.; Bardeau, J.-F.; Gibaud, A.; Mortier, J. Synthesis of Model Long-Chain ω-Alkenyltrichlorosilanes and Triethoxysilanes for the Formation of Self-Assembled Monolayers. *Tetrahedron* **2006**, *62* (4), 647–651. https://doi.org/10.1016/j.tet.2005.10.003.

(37) Bonger, K. M.; van den Berg, R. J. B. H. N.; Heitman, L. H.; IJzerman, A. P.; Oosterom, J.; Timmers, C. M.; Overkleeft, H. S.; van der Marel, G. A. Synthesis and Evaluation of Homo-Bivalent GnRHR Ligands. *Bioorg. Med. Chem.* **2007**, *15* (14), 4841–4856. https://doi.org/10.1016/j.bmc.2007.04.065.

(38) Bernard, C.; Loic, F.; Alain, P.; Claudine, S.-L.-G.; Gerard, V. Compounds Bearing Sulphamoyl and Amidino Radicals, Their Preparation Process and Pharmaceutical Compositions Containing Them. US 5714497 A, 1998.

(39) Das, M.; Du, Y.; Mortensen, J. S.; Hariharan, P.; Lee, H. S.; Byrne, B.; Loland, C. J.; Guan, L.; Kobilka, B. K.; Chae, P. S. Rationally Engineered Tandem Facial Amphiphiles for Improved Membrane Protein Stabilization Efficacy. *ChemBioChem* **2018**, *19* (20), 2225–2232. https://doi.org/10.1002/cbic.201800388.

(40) Maitra, A.; Lake, W. R.; Mohamed, A.; Edington, S. C.; Das, P.; Thompson, B. C.; Hammes-Schiffer, S.; Johnson, M.; Dawlaty, J. M. Measuring the Electric Fields of Ions Captured in Crown Ethers. *J. Phys. Chem. Lett.* **2024**, *15* (29), 7458–7465. https://doi.org/10.1021/acs.jpclett.4c01303.

(41) Sekiguchi, H.; Muranaka, K.; Osada, A.; Ichikawa, S.; Matsuda, A. Efficient Synthesis of Hsp90 Inhibitor Dimers as Potential Antitumor Agents. *Bioorg. Med. Chem.* **2010**, *18* (15), 5732–5737. https://doi.org/10.1016/j.bmc.2010.05.075.

(42) Shi, Y.; Duan, Y.-H.; Ji, Y.-Y.; Wang, Z.-L.; Wu, Y.-R.; Gunosewoyo, H.; Xie, X.-Y.; Chen, J.-Z.; Yang, F.; Li, J.; Tang, J.; Xie, X.; Yu, L.-F. Amidoalkylindoles as Potent and Selective Cannabinoid Type 2 Receptor Agonists with in Vivo Efficacy in a Mouse Model of Multiple Sclerosis. *J. Med. Chem.* **2017**, *60* (16), 7067–7083. https://doi.org/10.1021/acs.jmedchem.7b00724.

(43) Hwang, J. Y.; Ha, J. Du; Cho, S. Y.; Kim, P.; Yun, C. S. H. J.; Park, S. G.; Park, B. C.; Kim, J. H.; Kim, S. Target Protein EED Degradation-Inducing Degrader, Preparation Method Thereof, and Pharmaceutical Composition for Preventing or Treating Diseases Related to EED, EZH2, or PRC2, Comprising Same As Active Ingredient. WO 2020/162725 A1, 2020.

(44) Mori, R.; Kato, A.; Komenoi, K.; Kurasaki, H.; Iijima, T.; Kawagoshi, M.; Kiran, Y. B.; Takeda, S.; Sakai, N.; Konakahara, T. Synthesis and in Vitro Antitumor Activity of Novel 2-Alkyl-5-Methoxycarbonyl-11-Methyl-6H-Pyrido[4,3-b]Carbazol-2-Ium and 2-Alkylellipticin-2-Ium Chloride Derivatives. *Eur. J. Med. Chem.* **2014**, *82*, 16–35. https://doi.org/10.1016/j.ejmech.2014.05.032.

(45) Zhang, X.; Wu, X.; Jiang, S.; Gao, J.; Yao, Z.; Deng, J.; Zhang, L.; Yu, Z. Photo-Accelerated “Click” Reaction between Diarylsydnones and Ring-Strained Alkynes for Bioorthogonal Ligation. *Chem. Comm.* **2019**, *55* (50), 7187–7190. https://doi.org/10.1039/C9CC02882J.

(46) de Feijter, I.; Goor, O.; Hendrikse, S.; Comellas-Aragonès, M.; Söntjens, S.; Zaccaria, S.; Fransen, P.; Peeters, J.; Milroy, L.-G.; Dankers, P. Solid-Phase-Based Synthesis of Ureidopyrimidinone–Peptide Conjugates for Supramolecular Biomaterials. *Synlett* **2015**, *26* (19), 2707–2713. https://doi.org/10.1055/s-0035-1560520.
